# Pd-catalyzed stereoselective tandem ring-opening amination/cyclization of vinyl γ-lactones: access to caprolactam diversity†

**DOI:** 10.1039/d0sc03647a

**Published:** 2020-08-06

**Authors:** Jianing Xie, Xuetong Li, Arjan W. Kleij

**Affiliations:** Institute of Chemical Research of Catalonia (ICIQ), The Barcelona Institute of Science and Technology Av. Països Catalans 16 43007–Tarragona Spain akleij@iciq.es; Catalan Institute of Research and Advanced Studies (ICREA) Pg. Lluís Companys 23 08010 Barcelona Spain

## Abstract

A stereoselective amination/cyclization cascade process has been developed that allows for the preparation of a series of unsaturated and substituted caprolactam derivatives in good yields. This conceptually novel protocol takes advantage of the easy access and modular character of vinyl γ-lactones that can be prepared from simple precursors. Activation of the lactone substrate in the presence of a suitable Pd precursor and newly developed phosphoramidite ligand offers a stereocontrolled ring-opening/allylic amination manifold under ambient conditions. The intermediate (*E*)-configured ε-amino acid can be cyclized using a suitable dehydrating agent in an efficient one-pot, two-step sequence. This overall highly chemo-, stereo- and regio-selective transformation streamlines the production of a wide variety of modifiable and valuable caprolactam building blocks in an operationally attractive way.

## Abstract 2

A Pd-mediated stereoselective tandem process has been developed towards a large variety of functional caprolactam synthons.

## Graphical abstract

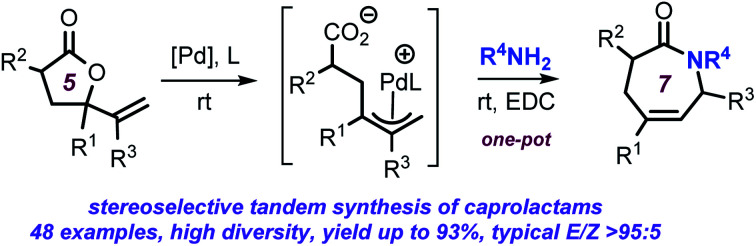

## Introduction

Lactam derivatives play an important role in the development of new pharmaceuticals^1^ and are useful as a starting point for synthetic polyamides,^2^ with caprolactam being the most prominent monomer known to date.^3^ Classic synthesis of lactams is achieved using methods such as the Beckmann rearrangement of oximes,^4^ the Schmidt reaction between an azide and a carbonyl compound,^5^ and through cyclization of amino acids or similar precursors.^6^ Apart from the easy availability of stoichiometric methodologies, catalytic formation of lactams has materialized as a more sustainable alternative within the synthetic community fueled by the need for a higher atom and resource economy.^7^ Efficient methodologies have been developed by Fujita, Yamaguchi *et al.* who reported on a Rh-catalyzed oxidative *N*-heterocyclization of amino alcohols giving primarily access to five- and six-membered lactams,^8^ whereas Hong and coworkers showed that direct lactam synthesis can be achieved using lactones and amines as reagents under Ir-catalysis.^9^ Other approaches that rely on the use of prefunctionalized substrates have also been recently disclosed.^10^

**Scheme 1:**
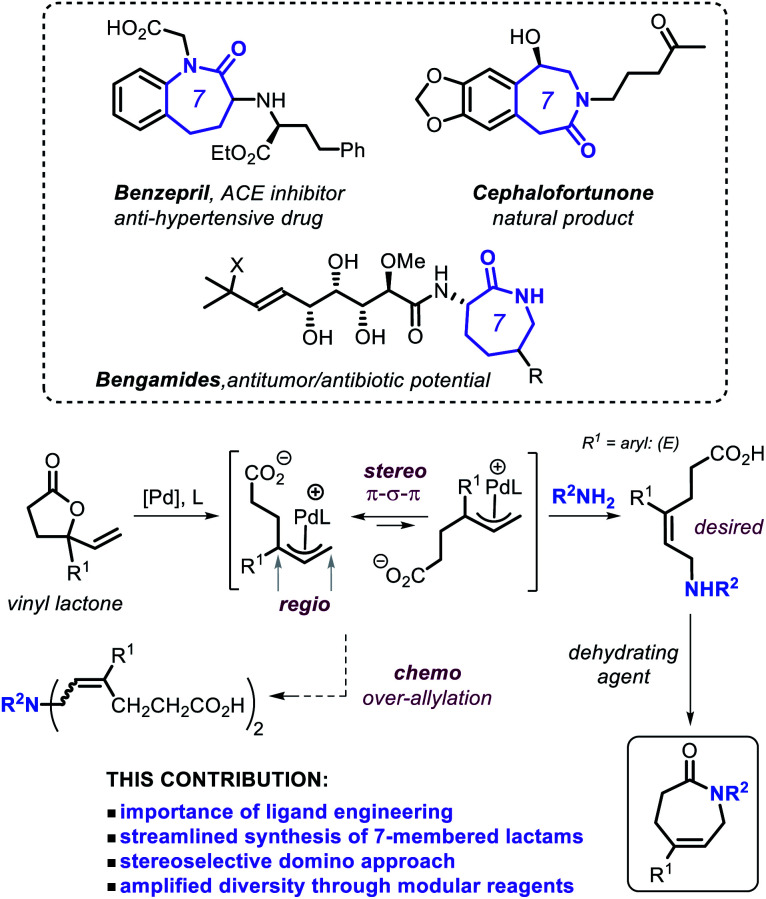
Approach towards the stereoselective domino synthesis of caprolactams.

Caprolactams represent key motifs of many natural products and other biologically active compounds (Scheme 1, top),^11^ and often serve as versatile synthetic intermediates for bicyclic amino compounds and azepane ring systems.^12^ Despite the recent progress noted for the catalytic synthesis of the more common five- and six-membered lactams,^4*b*,8–10,13^ the scope of functional and modular seven-membered analogues remains surprisingly limited. Therefore, designing new synthetic procedures that can streamline the preparation of such caprolactam building blocks can create new incentives for their use as synthons in drug-development programs and the creation of new polyamide monomers. With this challenge in mind, we decided to contemplate on combining our expertise in the area of allylic substitution chemistry^14^ and merging this with vinyl γ-lactones as modular substrates as outlined in Scheme 1.

Non-strained, stable γ-lactones have so far only sporadically emerged as versatile and readily accessible substrates under suitable catalytic conditions.^15^ More specifically, the use of vinyl γ-lactones (Scheme 1)^16^ may offer a tangible way to prepare ε-amino acid intermediates through allylic amination under Pd catalysis. In the overall manifold, proper ligand engineering^17^ is crucial to control simultaneously the chemo-, regio- and stereo-selectivity under ambient conditions in order to generate the (*E*)-configured ε-amino acid intermediate (Scheme 1) *en route* to the caprolactam target.^18^ Here we present a general and practically attractive approach for the preparation of a library of functional caprolactams through a novel and formal tandem ring-opening allylic amination/cyclization process, which is controlled by a newly developed, readily accessible phosphoramidite-based Pd catalyst.

## Results and discussion

At the onset of the screening studies, we considered to use previously reported successful phosphorus ligands^14,17^ to examine their efficacy to generate Pd-catalysts derived from Pd_2_(dba)_3_·CHCl_3_ (see the ESI†). We were pleased to find that the presence of phosphoramidite ligand **L1** proved to be highly beneficial for the ring-opening allylic amination step under ambient conditions. Furthermore, the *in situ* rapid cyclization of the intermediate (*E*)-ε-amino acid towards the lactam target using the well-known reagent EDC^19^ was combined in a one-pot two step sequence. Our screening then focused on producing the intermediate ε-amino acids **1** in the most efficient way from lactone **A** using phosphoramidite **L1** (Table 1, entries 1–6) as to favor the formation of the desired lactam **3**.^20^ The type of solvent has a significant effect on the outcome of the tandem process. The use of both DCM and MeOH gave access to moderate yields of **3** while minimizing the formation of the double allylated byproduct **2**. The presence of highly polar solvents such as DMSO and DMF or THF, however, resulted in unproductive processes, whereas the utilization of ACN lowered the chemo-selectivity of the process. It is important to note that in the absence of **L1** (entry 7), there was no formation of any product pointing at the crucial role of the ligand.

**Table 1:** Screening and optimization studies towards the stereoselective conversion of substrate **A** into caprolactam **3** using aniline as reagent^a^

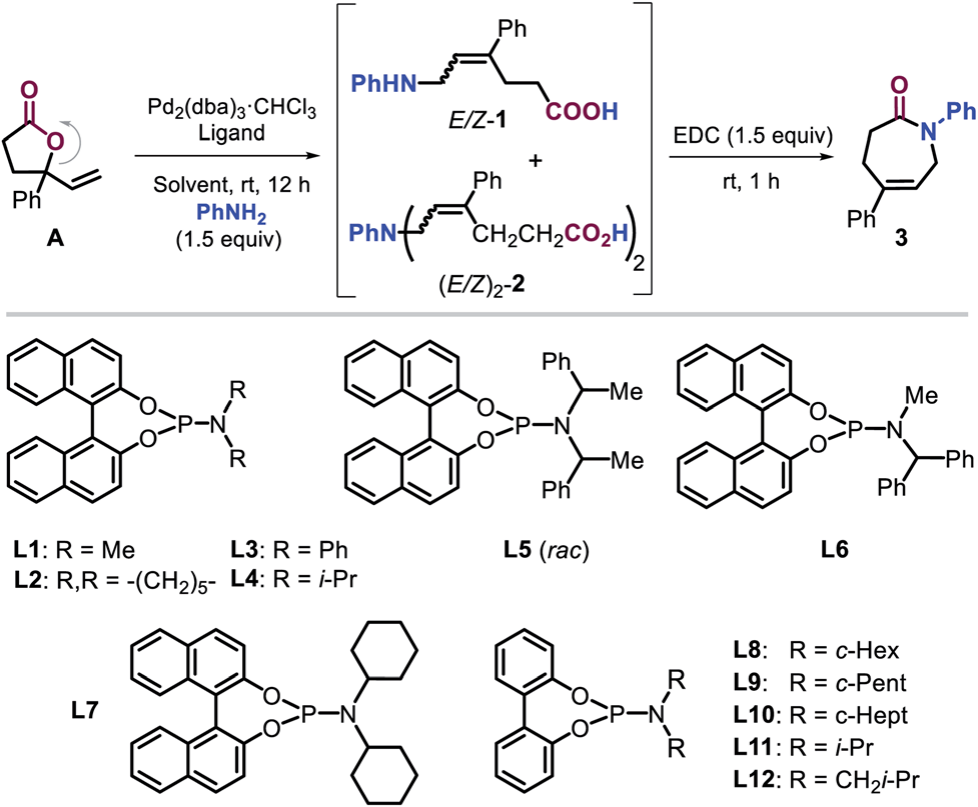						
Entry	**L**	Solvent	Yield^b^ of **1** (%)	*E* : *Z*-**1**^c^	**1**/**2**^c^	Yield^b^ of **3** (%)
1	**L1**	DCM	83	82 : 18	16 : 1	66
2	**L1**	THF	0	—	—	—
3	**L1**	ACN	67	89 : 11	5 : 1	59
4	**L1**	MeOH	78	82 : 18	17 : 1	64
5	**L1**	DMF	0	—	—	—
6	**L1**	DMSO	0	—	—	—
7	—	DCM	0	—	—	—
8	**L2**	DCM	85	77 : 23	16 : 1	65
9	**L3**	DCM	82	61 : 39	>20 : 1	53
10	**L4**	DCM	<1	—	—	—
11	**L5**	DCM	0	—	—	—
12	**L6**	DCM	0	—	—	—
13	**L7**	DCM	79	96 : 4	10 : 1	76
14	**L8**	DCM	80	97 : 3	10 : 1	77
15	**L9**	DCM	79	94 : 6	13 : 1	74
16	**L10**	DCM	<1	—	—	—
17	**L11**	DCM	0	—	—	—
18	**L12**	DCM	82	91 : 9	15 : 1	75
19^d^^,^^e^	**L8**	DCM	88	93 : 7	>20 : 1	82
20^d^^,^^e^^,^^f^	**L8**	DCM	86	98 : 2	>20 : 1	84

a Lactone **A** (0.20 mmol), PhNH_2_ (0.30 mmol, 1.5 equiv.), solvent (0.20 mL), Pd_2_(dba)_3_·CHCl_3_ (2.0 mol%), **L** (8.0 mol%), rt, 12 h; then EDC (0.30 mmol), 1 h.

b Determined by ^1^H NMR analysis in CDCl_3_ using CH_2_Br_2_ as an internal standard.

c Determined by ^1^H NMR analysis.

d PhNH_2_ (2.0 equiv.).

e Pd_2_(dba)_3_·CHCl_3_ (3.0 mol%), **L1** (12.0 mol%).

f Solvent (0.30 mL).

A variety of phosphoramidite ligands **L2–L12** (entries 8–18) were then scrutinized under the conditions of entry 1, illustrating the critical influence of the *N*-substitution and biaryl backbone on the chemo-, regio- and stereoselectivity of this catalytic process. For instance, too bulky *N*-substituents (**L4–L6**, entries 10–12) do not permit the formation of **3**, whereas ligands equipped with piperidine and phenyl (**L2** and **L3**) provided good yields of intermediate **1** with high chemoselectivity and moderate *E*/*Z* ratios (entries 8 and 9). To our delight, the introduction of cyclohexyl groups (**L7**, entry 13) further increased the *E*-stereoselective formation of **1** and thus the yield of **3** while retaining good chemo-selectivity. Interestingly, a binaphthyl ligand backbone was not essential to achieve high selectivity, which became clear from studying the biphenyl based ligands **L8–L12** and the steric effect of their *N*-substituents (entries 15–18). Cyclopentyl and i-Bu substituted ligands **L9** and **L12** provided productive and rather selective catalysts for the formation of **3** though with slightly lower yields and stereoselectivity as compared to **L8**. The reaction did not proceed with ligands comprising of cycloheptyl or i-Pr groups (**L10** and **L11**). These results indicate that steric modulation of the *N*-substituent of the ligand is key to the success of the formation of caprolactam **3**. From all ligands studied, the novel ligand **L8** (entry 14) provided the best combination of yield (for **1** and **3**) and selectivity features. A further increase in the yield of **3** in the presence of **L8** was finally obtained by increasing the amount of catalyst, aniline and solvent (entries 19 and 20) providing the caprolactam product in 84% yield and with high stereo- (*E*/*Z* = 98 : 2) and chemo-selectivity (**1** : **2** > 20 : 1).

**Scheme 2:**
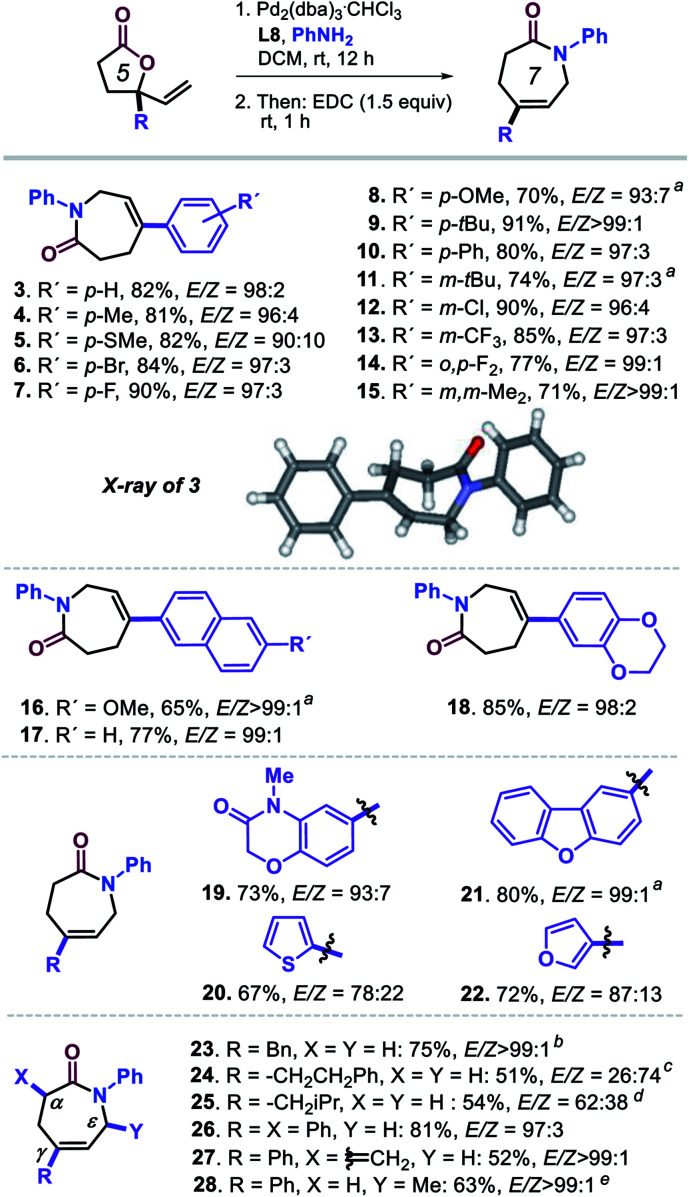
Scope in vinyl-substituted γ-lactone substrates. All reactions were performed under the optimized conditions (Table 1, entry 20). Isolated yields are reported, and the reported *E*/*Z* ratios for the intermediate ε-amino acids were determined by ^1^H NMR; the inset shows the molecular structure determined for **3** by X-ray analysis, see footnote 21. ^*a*^Using DCM/EtOH (1 : 1 v/v) as solvent. ^*b*^**L1** (12.0 mol%), MeOH (0.30 mL), 24 h. ^*c*^Using **L1** (12.0 mol%), MeOH, 24 h, 30 °C; note that the same stereoisomer is formed though with a different *E*/*Z* priority assignment, and thus in this case the *Z* isomer is required towards lactam formation. ^*d*^Using P(OPh)_3_ (12.0 mol%), MeOH, 24 h. ^*e*^**L1** (12.0 mol%), MeOH, 60 °C.

With the optimized conditions of entry 20 in Table 1, we then set out to examine the scope of this new transformation (Scheme 2, compounds **3–28**) focusing first on variation of the vinyl lactone reagent. Lactones with *para*-substituted aryl substituents were productive substrates giving the caprolactam derivatives **3–10** in high isolated yields (>80% in most cases) and with excellent stereo- (typically with *E*/*Z* ratios >95 : 5), regio- and chemo-selectivity control. The presence of *meta*-, *ortho*, *para*-di- or 3,5-di-substituted aryl groups (**11–15**; 71–90%, *E*/*Z* ≥ 96 : 4) did not have any significant effect on the outcome of the tandem process, and the desired products could be easily formed and isolated. Larger aromatic surfaces such as those present in lactam products **16** and **17** are also tolerated, while this new protocol furthermore allows the introduction of heteroaromatic fragments (**18–22**) in the final products.

In the case of **20** and **22** bearing a 2-thienyl and 3-furyl group, respectively, the observed stereoselectivity was significantly lower which may be ascribed to the potential of these groups to interact with the Pd-catalyst while affecting the π–σ–π equilibrium as depicted in Scheme 1. Finally, introducing other γ-substituents in the lactam product such as different alkyl substituents (**23–25**; 54–75%),^22^ and the introduction of additional α-positioned groups (**26**, 81%; **27**, 52%) were also feasible preserving excellent stereoselectivity. The methylidene substituted product **27** could act as potential Michael acceptor for late-stage modification towards the formation of biologically active compounds. The preparation of **28** (63%) additionally demonstrates that also lactones with substituted vinyl groups are productive substrates.^23^

**Scheme 3:**
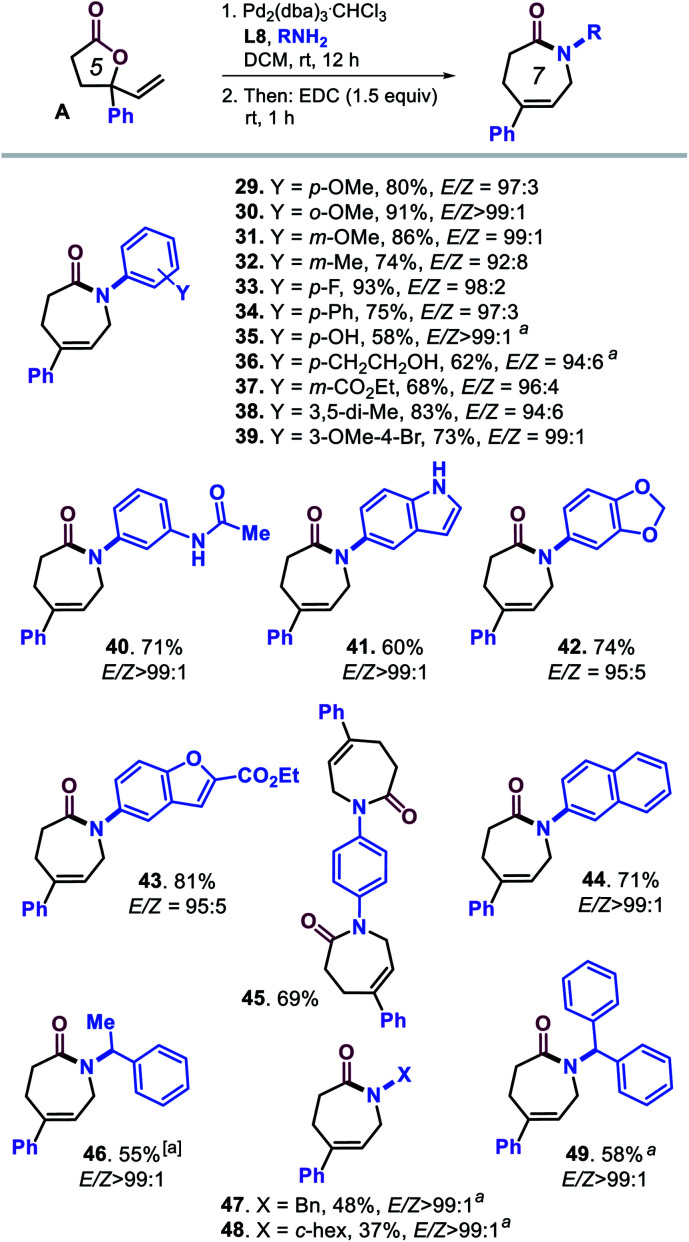
Scope in amine reagents. All reactions were performed under the optimized conditions (Table 1, entry 20). Isolated yields are reported, and the *E*/*Z* ratios for the intermediate ε-amino acids were determined by ^1^H NMR, though in the preparation **45**, the *E*/*Z* selectivity could not be determined accurately. ^*a*^Using DCM/EtOH (1 : 1 v/v) as solvent.

Our attention was then on further widening the scope of caprolactam products by combining vinyl lactone **A** and various amine reaction partners (Scheme 3, compounds **29–49**). A range of substituted anilines could be coupled to ring-opened lactone **A** under the optimized conditions providing overall good to excellent yields of caprolactams **29–40** under high stereocontrol. The use of larger (hetero)aromatic amines (**41–44**) gave access to lactam products with *N*-indole, *N*-1,3-benzodioxole, *N*-benzofuran and *N*-naphthyl groups. The di-lactam derivative **45** could be prepared in appreciable yield (69%) despite the more challenging nature of this formal tandem bis allylic amination/cyclization process.

Lastly, we tested several, more nucleophilic aliphatic amines to challenge the chemo-selectivity of our developed protocol. As may be expected, substantially lower yields for **46–49** were obtained (37–58%) though under excellent stereocontrol (*E*/*Z* > 99 : 1) in a mixed solvent system consisting of DCM/EtOH. The ^1^H NMR analysis of the crude products indeed indicated that larger amounts of bis-allylated amines of type **2** had been formed.^24^

**Scheme 4:**
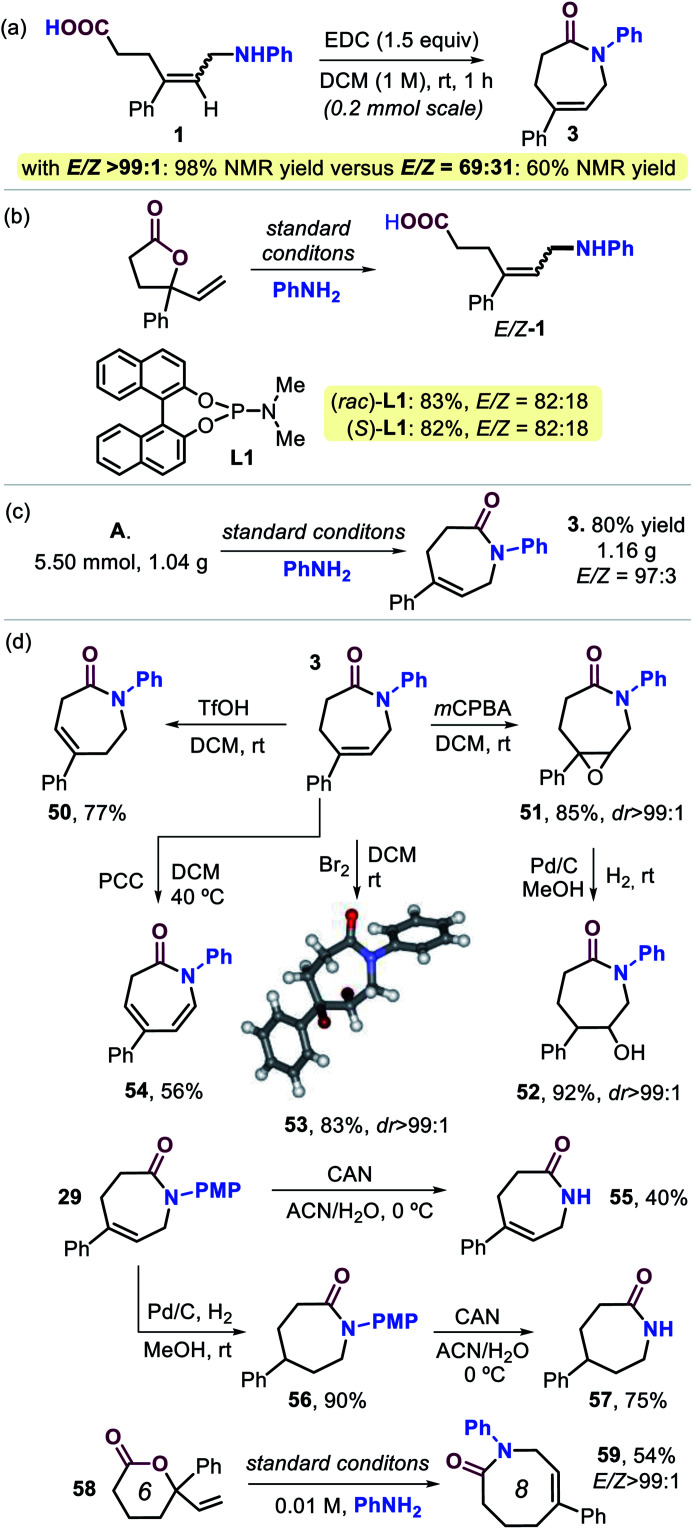
(a and b) Control experiments. (c) Gram-scale preparation of **3**. (d) Product diversification studies based on **3** and **29**. For details, see the ESI.†

In order to examine whether the stereoselectivity indeed plays an important role prior to the cyclization step (Scheme 1), we compared the yield of lactam **3** attained from a configurationally pure sample of **1** (*E*/*Z* > 99 : 1) and one being a 69 : 31 *E*/*Z* mixture (Scheme 4a). From the results, it may be concluded that the maximum yield unquestionably depends on the amount of *E*-**1** that is initially formed, and also molecular models strongly suggest that the (*Z*) isomer of **1** is unable to cyclize towards a lactam product. These results reinforce the assumption that stereocontrol in the first step is vital to the success of the overall manifold. The use of a simple ligand structure (*cf.*, **L8**) shows that the presence of a chiral ligand is not a requisite. This is demonstrated by the fact that both (*rac*)- and enantiopure (*S*)-**L1** give virtually the same stereocontrol (for **1**) and lead to a similar yield of **3** (Scheme 4b), thus being consistent with the results described in Table 1.

The practicality of this new procedure for the preparation of functional seven-membered lactams is illustrated by the scale-up of compound **3** (Scheme 4c), and product diversification studies using **3** and **29** were also initiated (Scheme 4d). After establishing that scaling up does not influence the stereo-outcome or yield of the product (*cf.*, Scheme 2 and **3**), several post-modifications were carried out. Triflic acid mediated double bond isomerization in **3** was feasible to produce **50** in good yield, whereas epoxidation afforded **51** with excellent diastereoselectivity. The latter compound could be reduced by using Pd-catalyzed hydrogenation leading to **52** in high yield. Dibromination of **3** gives **53** (83%, with its molecular structure confirmed by X-ray analysis, see Scheme 4),^21^ whereas pyridinium chlorochromate (PCC) assisted oxidation of **3** furnished the conjugated, bis-unsaturated caprolactam derivative **54**. In order to provide free lactams potentially useful as a new type of monomer for polyamide synthesis or pharmaceutical precursors, compound **29** was treated with cerium ammonium nitrate (CAN) to afford **55** in 40% yield. Alternatively, standard hydrogenation of **29** (**56**, 90%) followed by deprotection gave easy access to free lactam **57** in 75% yield. Finally, six-membered vinyl lactone **58** could be transformed into eight-membered lactam **59** (54%) with high stereocontrol, and this implies that the developed cascade process may be suitable for the preparation of an even wider range of larger ring lactams.^25^

## Conclusions

In summary, this work provides a new, efficient and practical approach for valuable caprolactam scaffolds that involves (after ring-opening of vinyl γ-lactones) a Pd-mediated stereo- and regio-selective allylic amination followed by a cyclization step affording the lactam target. This formal tandem process relies on the use of a new, easily prepared phosphoramidite ligand (**L8**) that allows to control the various process selectivity features and, as such, the yield of the final product under ambient conditions. The scale-up and diversification studies show that more functional scaffolds may be created that can be of great value to build up molecular complexity from this new library of readily accessible caprolactam building blocks.

## Conflicts of interest

There are no conflicts to declare.

## Supplementary Material

SC-011-D0SC03647A-s001

SC-011-D0SC03647A-s002
