# Supplementary material for: Pd-catalyzed stereoselective tandem ring-opening amination/cyclization of vinyl γ-lactones: access to caprolactam diversity
Source: Chem Sci. 2020 Aug 6;11(33):8839–45. doi: 10.1039/d0sc03647a (PMC8163440; doi:10.1039/d0sc03647a)
Supplement: SC-011-D0SC03647A-s001 [file SC-011-D0SC03647A-s001.pdf]

Electronic Supplementary Material (ESI) for Chemical Science.
This journal is © The Royal Society of Chemistry 2020

Electronic Supporting Information (ESI) for:

Pd-catalyzed stereoselective tandem ring-opening amination/cyclization of vinyl γ-lactones: Access to caprolactam diversity

Jianing Xie,[a] Xuetong Li[a] and Arjan W. Kleij*[a,b]

[a] Institute of Chemical Research of Catalonia (ICIQ), the Barcelona Institute of Science and Technology, Av. Països Catalans 16, 43007 - Tarragona, Spain

[b] Catalan Institute of Research and Advanced Studies (ICREA), Pg. Lluís Companys 23, 08010 Barcelona, Spain

E-mail: akleij@iciq.es

Contents:

Page S2: General comments
Page S2: Preparation of γ-oxobutanoic acids
Page S3: Procedure for the preparation of vinyl γ-lactones
Page S4: Procedure for the preparation of phosphoramidite ligands
Page S6: Table S1: Preliminary evaluation of ligands
Page S7: Table S2: Other ligands used to optimize the reaction conditions
Page S8: Table S3: Further optimization towards caprolactam **3**
Page S9: Procedure for the screening phase in the preparation of caprolactam **3**
Page S10: Typical procedure for the preparation of caprolactams
Page S11: Characterization data for non-reported phosphoramidite ligands
Page S32: IR, NMR spectra, HRMS data for vinyl γ-lactones
Page S87: Characterization data for the amino acid intermediate *E*-**1**
Page S90: IR, NMR spectra and HRMS data for caprolactam products
Page S188: Product diversification based on compounds **3** and **29**
Page S211: Control reactions
Page S219: Characterization of byproduct **2** from bis-allylation of aniline
Page S222: X-ray structure of **3** and **53**
Page S223: References

S2. General comments

The amine reagents and solvents were purchased from Aldrich or TCI, and used without further purification. Phosphoramidite ligands **L1**,[1a] **L2**,[1b] **L3**,[1c] **L4-L5**,[1d] **L6**,[1c] **L7-L12**,[1d] **L13**[1a] and **L14-L19**[1d] were prepared according to previously reported protocols. Other ligands and palladium precursors were purchased from Aldrich or TCI. ^{1}H NMR, ^{13}C{^{1}H} NMR, ^{31}P{^{1}H} NMR and ^{19}F{^{1}H} NMR spectra were recorded at room temperature on a Bruker AV-400 or AV-500 spectrometer and referenced to the residual deuterated solvent signals. All reported NMR values are given in parts per million (ppm). FT-IR measurements were carried out on a Bruker Optics FTIR Alpha spectrometer. Mass spectrometric analyses and X-ray diffraction studies were performed by the Research Support Group at ICIQ.

S2. Preparation of γ-oxobutanoic acids

The γ-oxobutanoic acids were synthesized following reported procedures if not commercially available. **H1-H9** were purchased from Aldrich or TCI, **H10-H14**,[2a] **H15-H18**,[2b] **H19**,[2c] **H20**,[2d] **H21**,[2e] **H22**,[2f] **H23**,[2g] **H24**[2h] and **H25**[2i] were prepared following previously reported procedures.

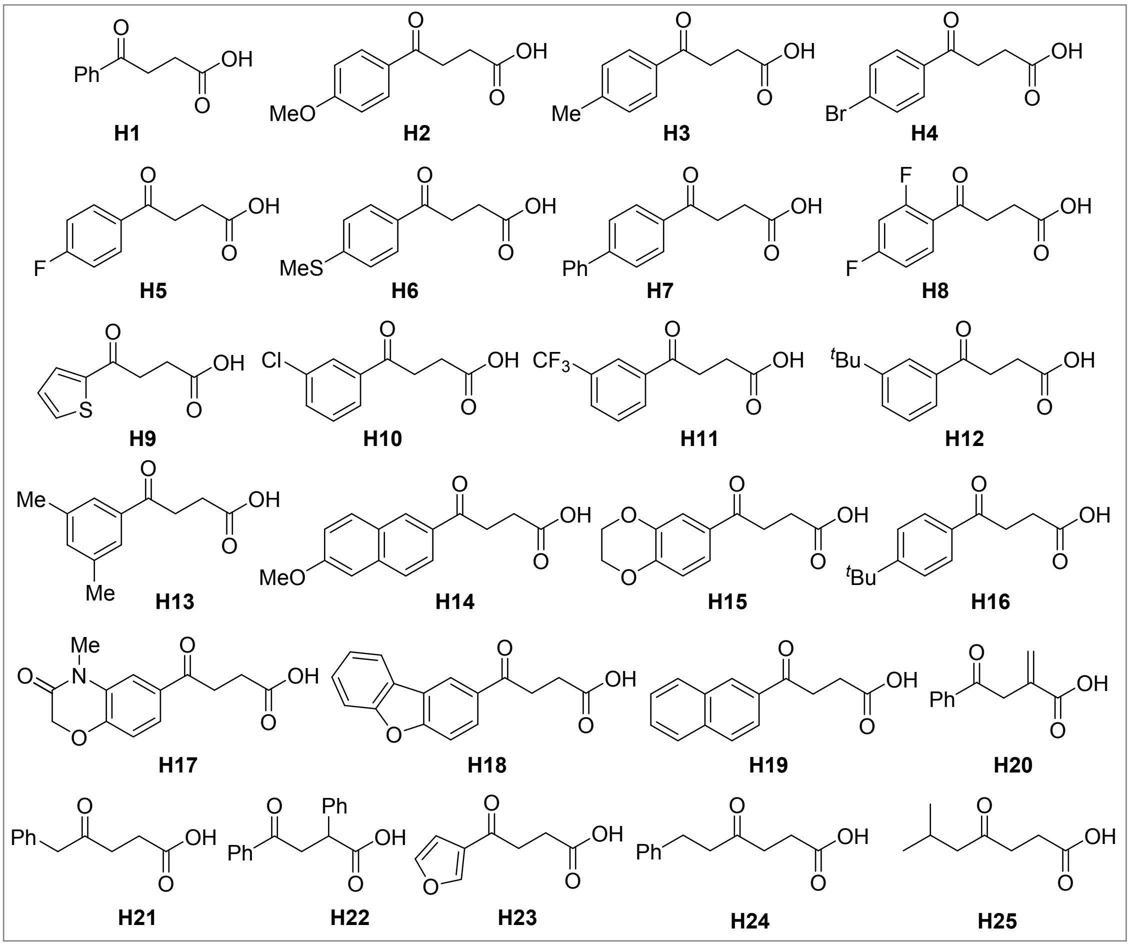

S3. Procedure for the preparation of vinyl γ-lactones

General Procedure A:

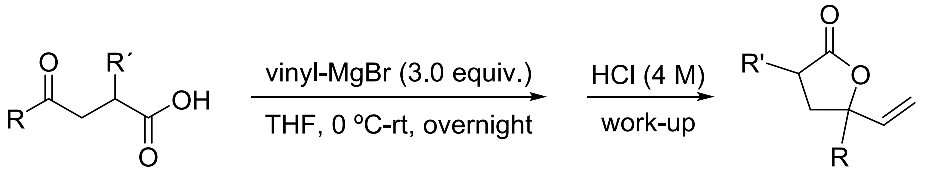

Under a N_2 atmosphere, to a separate flame-dried round-bottom flask equipped with a stirring bar was added the respective γ-oxobutanoic acid (10.0 mmol) and anhydrous THF (30 mL). The solution was cooled down to 0 ºC (ice/water), followed by dropwise addition of vinyl magnesium bromide in THF (1.0 M, 30.0 mL, 30.0 mmol). The reaction mixture was allowed to warm to room temperature and stirred for 16 h. The reaction mixture was quenched with saturated aqueous NH_4Cl, then treated with HCl (4 M) until the pH was 3. The organic components were extracted with EtOAc (3 × 20 mL). Hereafter, the combined organic layers were washed with brine, dried over anhydrous Na_2SO_4, filtered, and then concentrated under reduced pressure. The residue was purified by flash chromatography to afford the corresponding lactone.

General Procedure B:

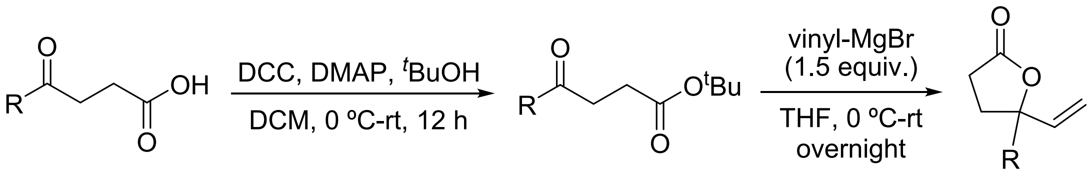

According to a previously reported procedure,[3] to a solution of γ-oxobutanoic acid (10.0 mmol, 1.0 equiv) and *tert*-butyl alcohol (20.0 mmol, 2.0 equiv) in DCM (20 mL), DMAP (3.0 mmol, 0.3 equiv) was added. The resultant solution was cooled down to 0 °C and *N,N'*-dicyclohexylcarbodiimide (DCC, 12.0 mmol, 1.2 equiv) was added. The reaction mixture was stirred at room temperature for 12 h. The urea byproduct was filtered off and the organic layer was concentrated under vacuum. The crude residue was purified by a rapid flash chromatographic purification to give the corresponding ester product.

Under a N_2 atmosphere, to a separate flame-dried round-bottom flask equipped with a stirring bar was added the respective γ-oxobutanoic ester (5.0 mmol) and anhydrous THF (15 mL). The solution was cooled down to 0 ºC (ice/water), followed by dropwise addition of vinyl magnesium bromide in THF (1.0 M, 7.5 mL, 7.5 mmol). The reaction mixture was allowed to warm to room temperature and stirred for 16 h. The reaction mixture was quenched with saturated aqueous NH_4Cl. The organic components were extracted with EtOAc (3 × 20 mL), and the combined organic layers were washed with brine, dried over anhydrous Na_2SO_4, filtered, and then concentrated under reduced pressure. The residue was purified by flash chromatography to afford the corresponding lactone.

The desired starting materials could be prepared by methods A or B in 30-70% isolated yields without further optimization.

S4. Procedure for the preparation of phosphoramidite ligands

The non-reported ligands **L8-L12**, **L16-L17**, **L19** were prepared according to a reported procedure with slight modifications.[2d]

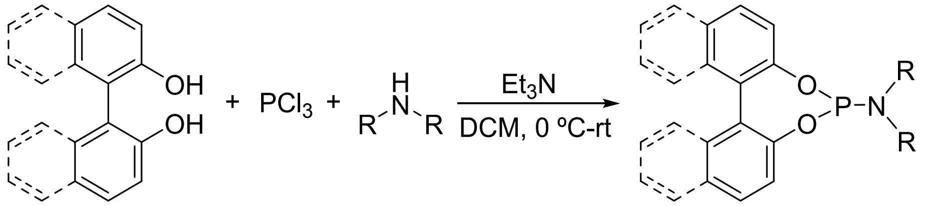

To an oven-dried round bottom flask, distilled PCl_3 (180.0 μL, 2.06 mmol) was added to a solution of anhydrous Et_3N (1.68 mL, 12.06 mmol) in DCM (15 mL) at 0 °C and the mixture stirred for 0.5 h at this temperature. Then, the respective amine (2.0 mmol) was added dropwise at 0 °C, and the reaction mixture stirred for 4 h at room temperature. The resultant solution was cooled to 0 °C, and then [1,1-biphenyl]-2,2-diol (372.4 mg, 2.0 mmol) or (±)-1,1′-binaphthalene-2,2′-diol (572.7 mg, 2.0 mmol) was added. The mixture was stirred for 16 h at room temperature, diluted with water and extracted with DCM (3 × 20 mL). The combined organic layers were dried over anhydrous Na_2SO_4, filtered, and then concentrated under reduced pressure. The residue was purified by flash chromatography to afford the respective phosphoramidite ligand. All purified ligands were fully characterized by NMR (^1H, ^{13}C, ^{31}P), IR and HRMS. *[Note that, in some cases, it proved to be crucial to isolate the pure ligand by chromatography under a N_2 atmosphere]*

Dicyclopentylamine and dicycloheptylamine, being the starting materials of **L9-L10** and **L16-L17**, were prepared following reported procedures.[4]

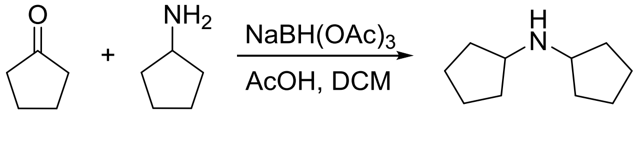

To a stirred solution of the cyclopentanone (420.6 mg, 5.0 mmol) and cyclopentylamine (425.8 mg, 5.0 mmol) in DCM (15 mL) were added sodium triacetoxy borohydride (1.4836 g, 7.0 mmol) and acetic acid (300.0 mg, 5.0 mmol). The reaction mixture was stirred for 12 h at room temperature, and hereafter, 1 N aqueous NaOH was added. The resultant mixture was extracted with ester (3 × 20 mL). The combined organic layers were dried over anhydrous Na_2SO_4, filtered, and then concentrated under reduced pressure. The crude product could be directly used without further purification.

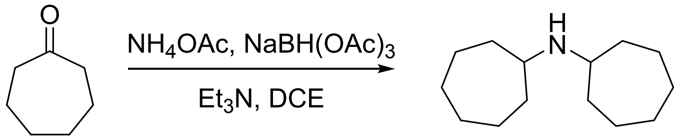

To a stirred solution of cycloheptanone (1.0 g, 8.9 mmol) and NH_4OAc (6.63 g, 86.0 mmol) in DCE (30 mL) were added sodium triacetoxy borohydride (2.65 g, 12.5 mmol) and Et_3N (2.5 mL, 17.9 mmol). The reaction mixture was stirred for 48 h at room temperature, followed by addition of saturated aqueous $NaHCO_3$. The resultant mixture was extracted with EtOAc (3 × 20 mL). The combined organic layers were washed with brine, dried over anhydrous Na_2SO_4, filtered, and then concentrated under reduced pressure. The residue was purified by flash chromatography to afford the desired amine as a yellow liquid.

S6. Table S1: Preliminary evaluation of ligands

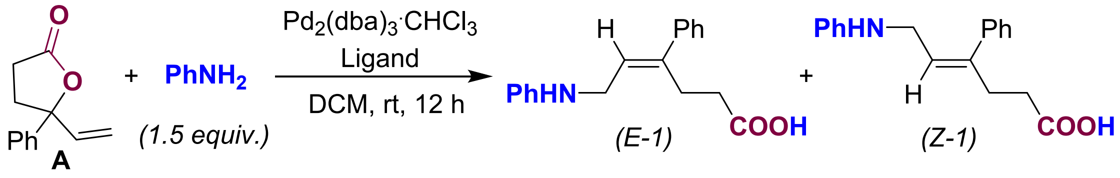

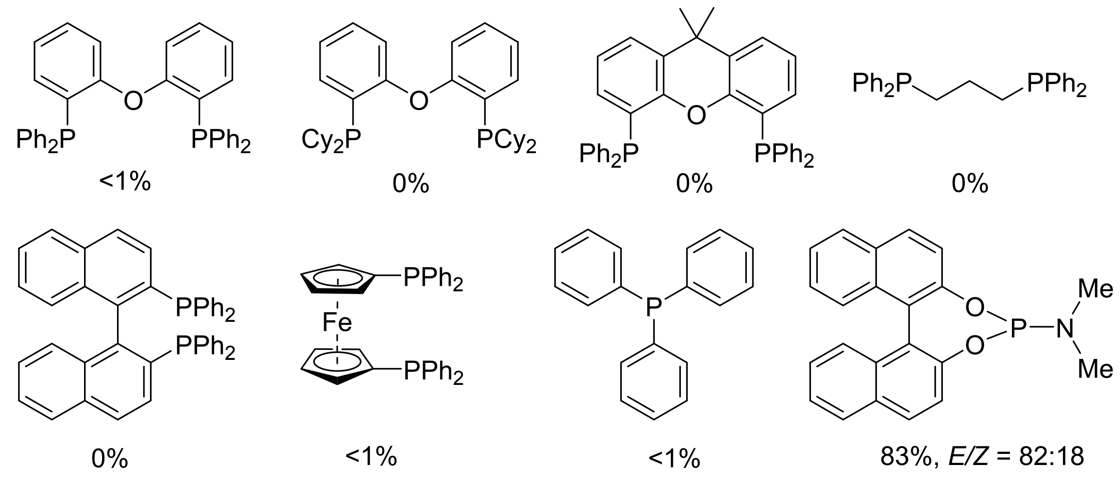

Reaction conditions: **A** (0.20 mmol), aniline (0.30 mmol, 1.5 equiv.), DCM (0.20 mL), $Pd_2(dba)_3{\cdot}CHCl_3$ (2.0 mol%), monodentate ligand (8.0 mol%) or bidentate ligand (4.0 mol %), rt, 12 h; the yields and *E/Z* ratios were determined by ^1H NMR analysis of the crude reaction mixture, using CH_2Br_2 (1.0 equiv.) as an internal standard.

S7. Table S2: Other ligands used to optimize the reaction conditions

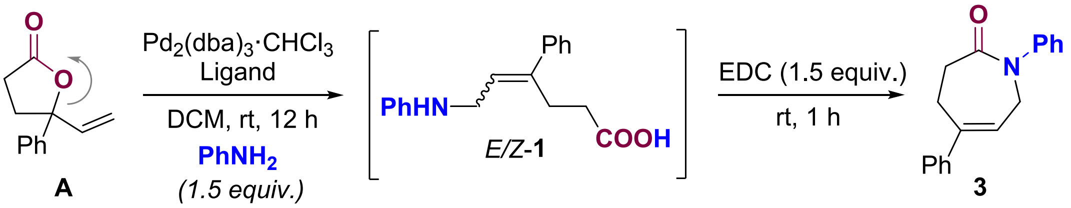

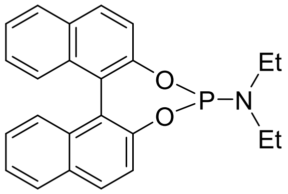

L13: 92%, *E/Z* = 79:21

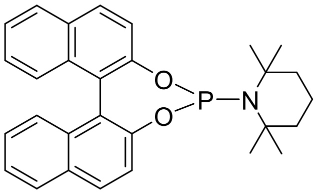

L14: 0%

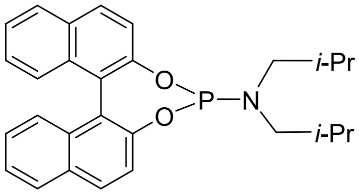

L15: 84%, *E/Z* = 87:13

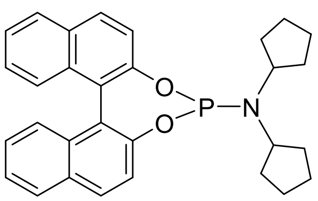

L16: 78%, *E/Z* = 94:6

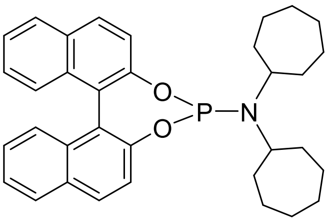

L17: <1%

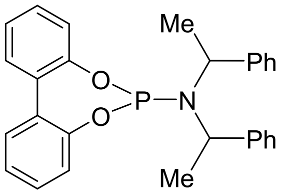

L18: <1%

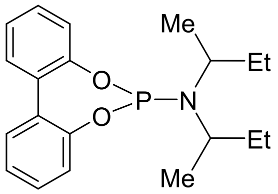

L19: <1%

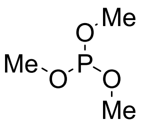

L20: <1%

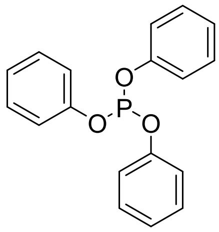

L21: 87%, *E/Z* = 80:20

Reaction conditons: **A** (0.20 mmol), aniline (0.30 mmol, 1.5 equiv.), DCM (0.20 mL), $Pd_2(dba)_3 \cdot CHCl_3$ (2.0 mol%), ligand (8.0 mol%), rt, 12 h, then EDC (0.30 mmol), 1 h; the yields and *E/Z* ratios were determined by ^1H NMR analysis of the crude reaction mixture, using CH_2Br_2 (1.0 equiv.) as an internal standard.

S8. Table S3: Further optimization towards caprolactam 3

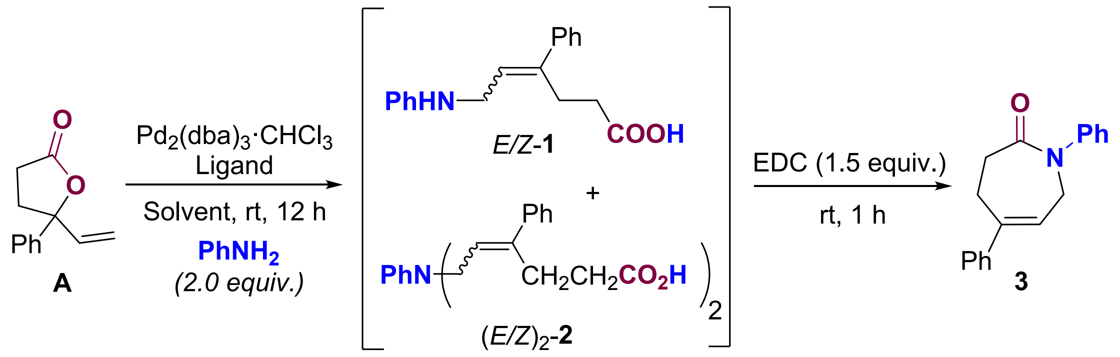

Entry[a]	Ligand	Solvent	*E*:*Z*-**1**[c]	**1**/**2**[c]	Yield of **3** [%][b]
1	**L8**	DCM	98:2	>20:1	84
2	**L8**	DCE	-	-	0
3	**L8**	$CHCl_3$	98:2	12:1	70
4	**L8**	MeOH	94:6	7:1	62
5	**L8**	EtOH	90:10	>20:1	76
6	**L8**	*i*-PrOH	96:4	7:1	70
7	**L8**	*t*-BuOH	98:2	7:1	68
8	**L8**	HFIP	92:8	7:1	52
9	**L8**	EtOH/DCM (1:1)	95:5	>20:1	84
10	**L8**	EtOH/DCM (2:1)	94:6	18:1	78

[a] Reaction conditons: **A** (0.20 mmol), aniline (0.40 mmol, 2.0 equiv.), solvent (0.30 mL), $Pd_2(dba)_3·CHCl_3$ (3.0 mol%), **L8** (12.0 mol%), rt, 12 h, then EDC (0.30 mmol), 1 h; [b] Determined by ^1H NMR analysis in $CDCl_3$ using CH_2Br_2 as an internal standard. [c] Determined by ^1H NMR analysis.

S9. Procedure for the screening phase in the preparation of caprolactam 3

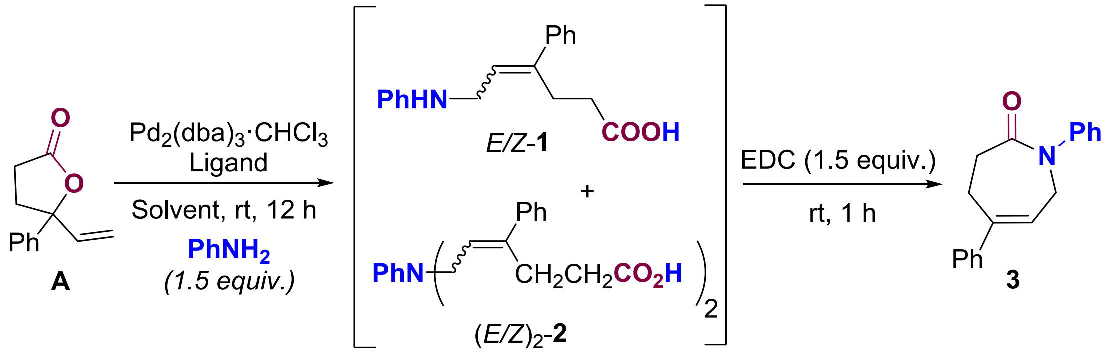

Vinyl γ-lactone **A** (37.6 mg, 0.20 mmol, 1.0 equiv) was combined with $Pd_2(dba)_3 \cdot CHCl_3$, the ligand, aniline and the solvent at room temperature under air. The internal standard CH_2Br_2 (1.0 equiv.) was added after the reaction mixture had been stirred at room temperature for 12 h, and then an aliquot of the mixture was taken for analysis allowing to determine the NMR yield of the amino acid intermediate, the *Z/E* ratio and the ratio **1**/**2** using signal integration. Hereafter, EDC (57.5 mg, 3.0 mmol, 1.5 equiv.) was added and the reaction mixture stirred for another 1 h, after which the yield of the targeted product **3** was determined by ^{1}H NMR spectroscopy.

S10. Typical procedure for the preparation of caprolactams

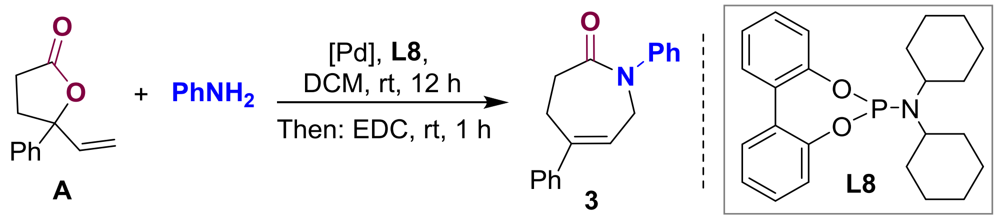

Representative case:
Vinyl γ-lactone **A** (37.6 mg, 0.20 mmol, 1.0 equiv) was combined with $Pd_2(dba)_3 \cdot CHCl_3$ (6.0 mg, 3.0 mol%), **L8** (9.6 mg, 12.0 mol%) and aniline (38.0 mg, 0.40 mmol) in DCM (0.30 mL) at room temperature under air. The reaction mixture was stirred at room temperature for 12 h, then an aliquot of the mixture was taken for NMR analysis, which provided the unsaturated amino acid intermediate with an *E/Z* ratio of 98:2. Then, EDC (57.5 mg, 3.0 mmol) was added and the reaction mixture stirred for another 1 h. The desired product was isolated by flash chromatography (43.2 mg, 82%, Hexane/EtOAc = 2:1, R_f = 0.25). Note that all purified caprolactam products were fully characterized by NMR (^{1}H, ^{13}C; ^{19}F where appropriate), IR and HRMS.

S11. Characterization data for non-reported phosphoramidite ligands

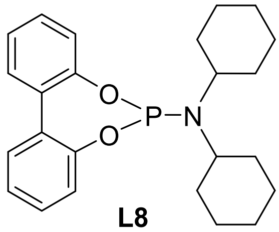

L8

White solid; Column conditions: Hexane : EA = 50 : 1, R_f = 0.30 (Note: EA stands for ethyl acetate)

^{1}H NMR spectrum ($CDCl_3$)

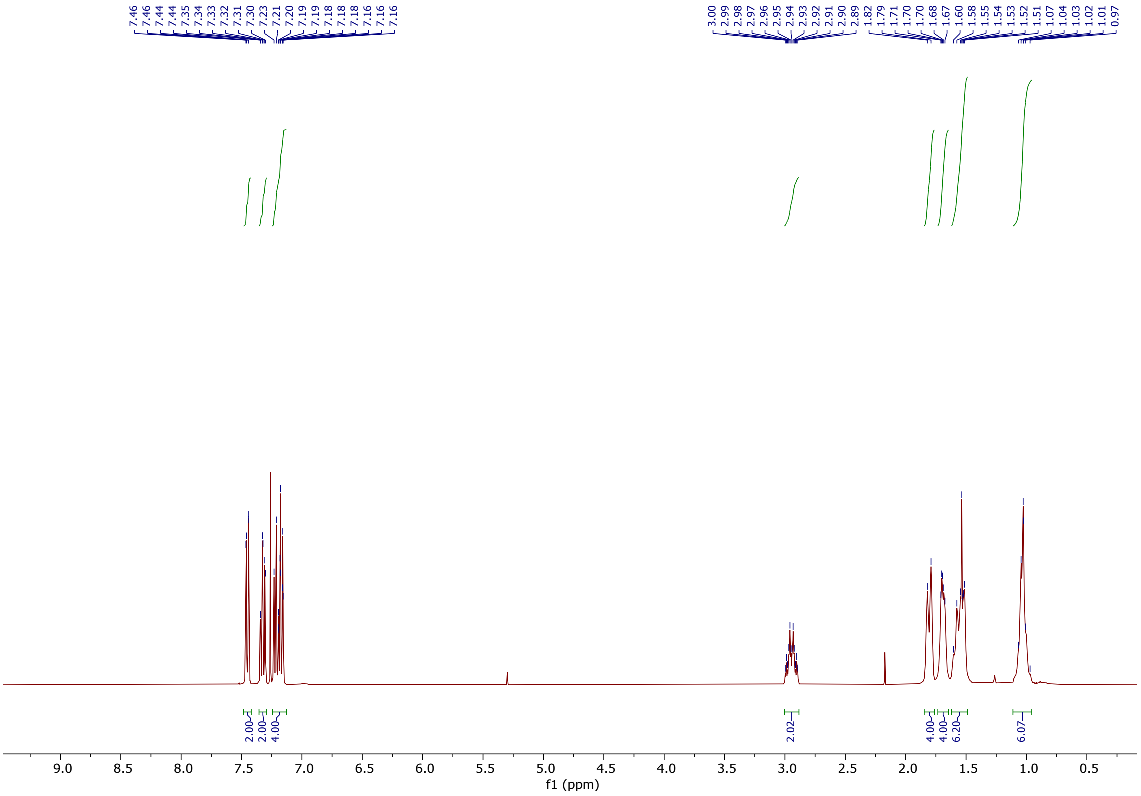

^{1}H NMR (400 MHz, $CDCl_3$) δ 7.45 (dd, J = 7.6, 1.7 Hz, 2H), 7.32 (td, J = 7.7, 1.7 Hz, 2H), 7.24 – 7.13 (m, 4H), 3.01 – 2.90 (m, 2H), 1.81 (d, J = 12.4 Hz, 4H), 1.73 – 1.65 (m, 4H), 1.62 – 1.49 (m, 6H), 1.07 – 0.96 (m, 6H).

^{13}C NMR spectrum ($CDCl_3$)

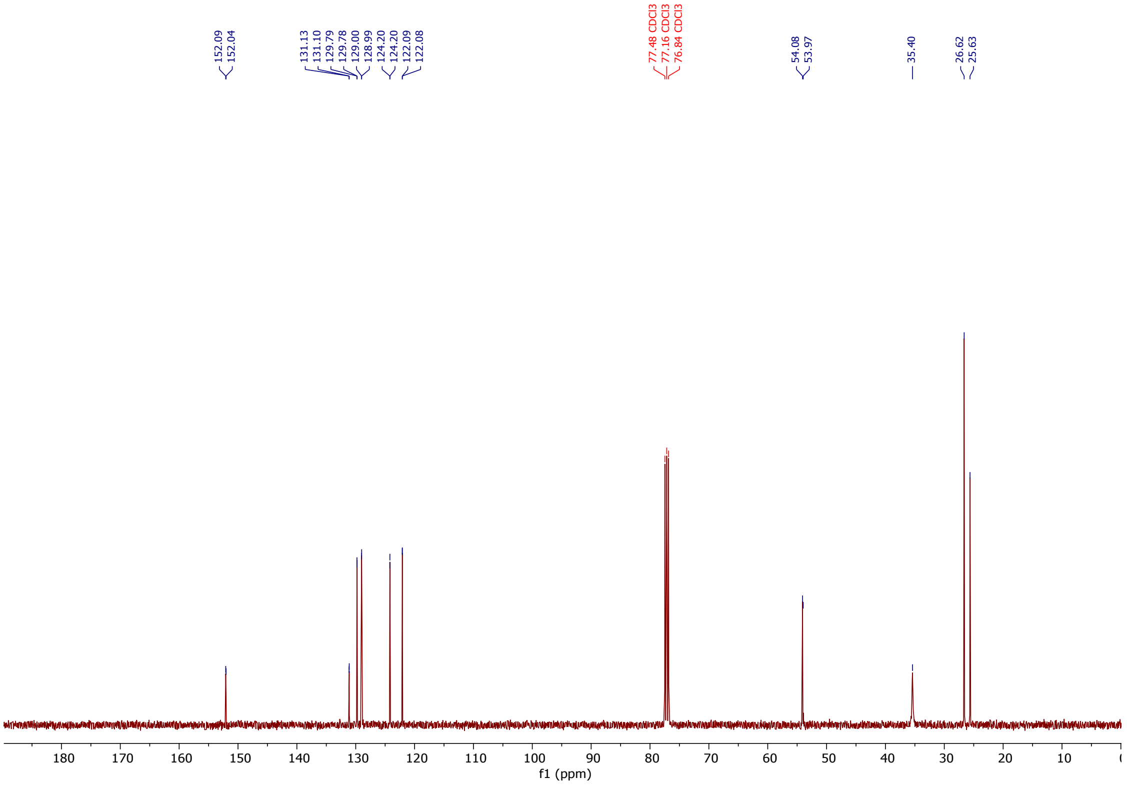

^{13}C NMR (101 MHz, $CDCl_3$) δ 152.09, 152.04, 131.13, 131.10, 129.79, 129.78, 129.00, 128.99, 124.20, 124.20, 122.09, 122.08, 54.08, 53.97, 35.40, 26.62, 25.63.

^{31}P NMR spectrum ($CDCl_3$)

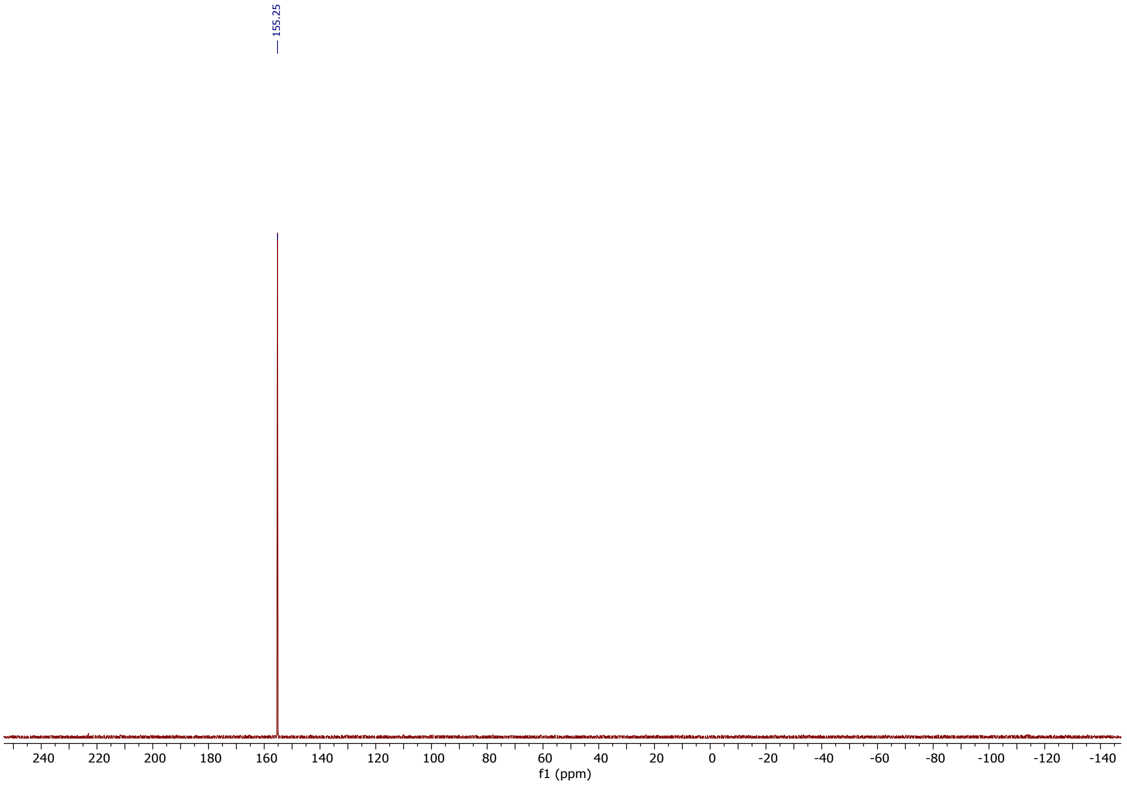

^{31}P NMR (162 MHz, $CDCl_3$) δ 155.25.

IR spectrum (neat)

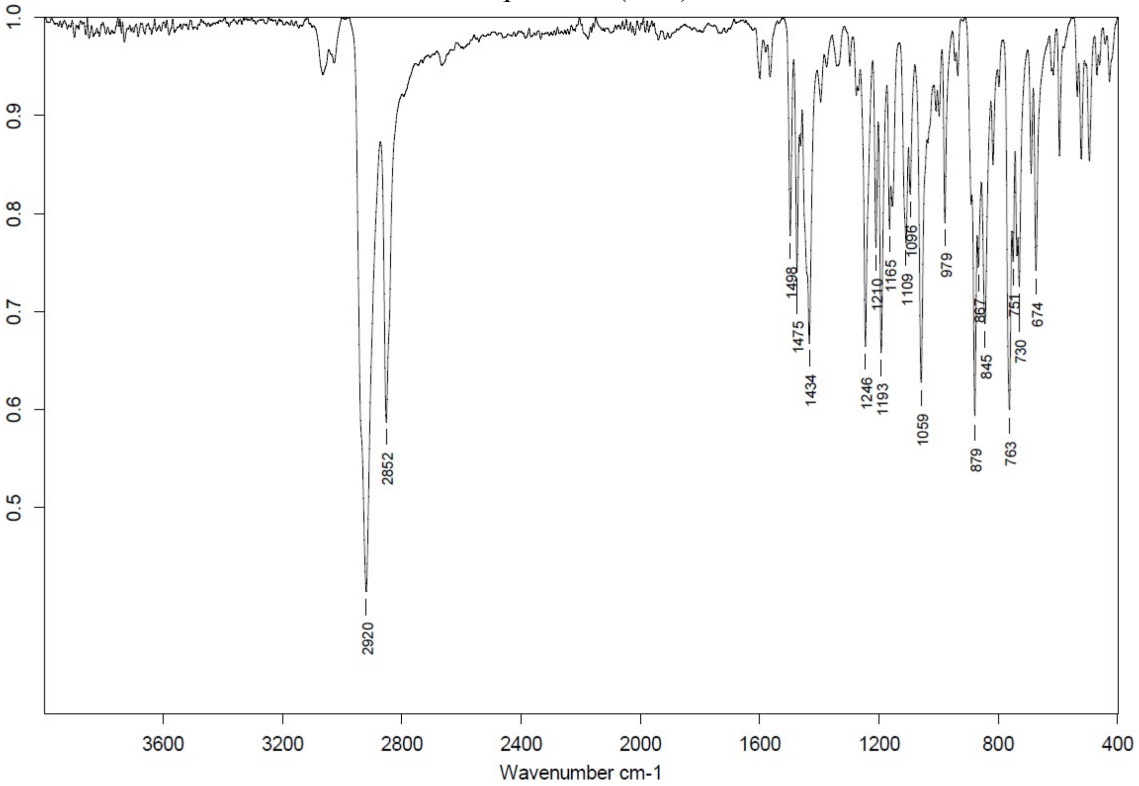

HRMS (ESI$^+$, MeOH): *m/z* calcd. 396.2087 (M + H)$^+$, found: 396.2090.

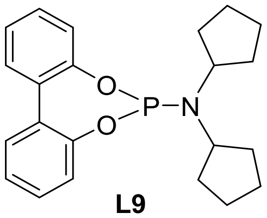

L9

White solid; Column conditions: Hexane : EA = 50 : 1, R_f = 0.30

^{1}H NMR spectrum ($CDCl_3$)

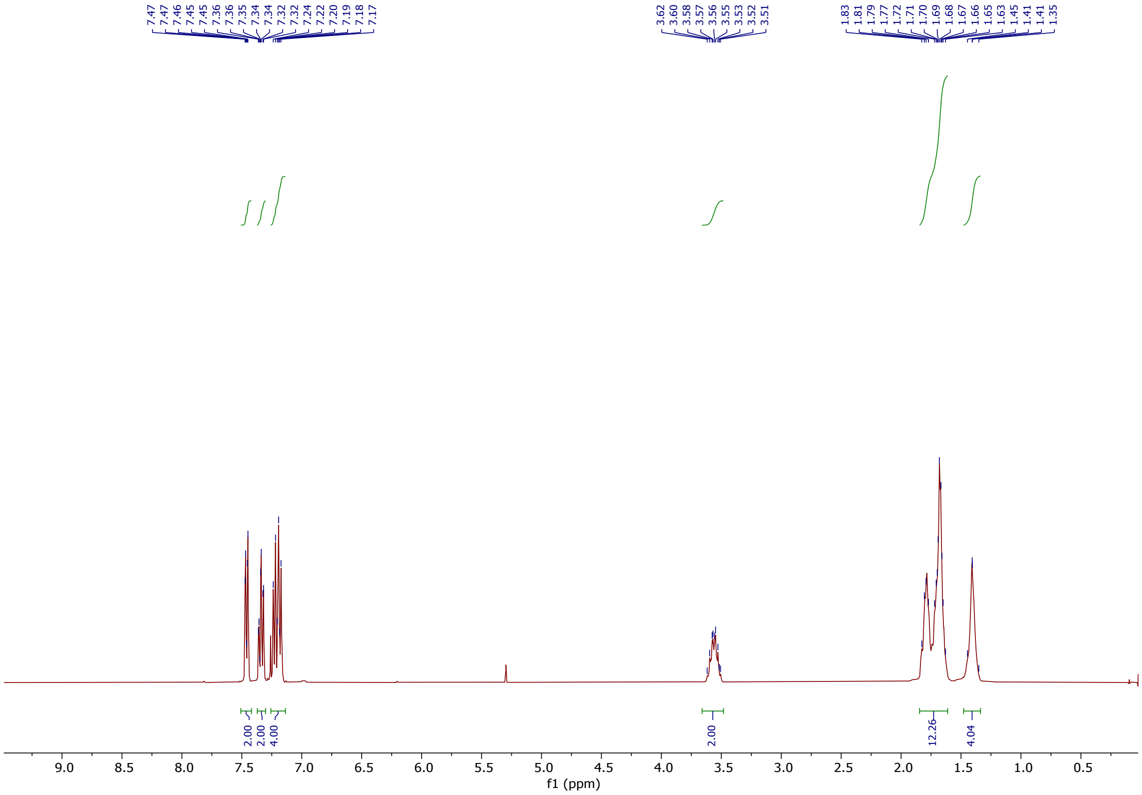

^{1}H NMR (400 MHz, $CDCl_3$) δ 7.50 – 7.42 (m, 2H), 7.35 – 7.30 (m, 2H), 7.26 – 7.14 (m, 4H), 3.66 – 3.48 (m, 2H), 1.85 – 1.61 (m, 12H), 1.48 – 1.34 (m, 4H).

^{13}C NMR spectrum ($CDCl_3$)

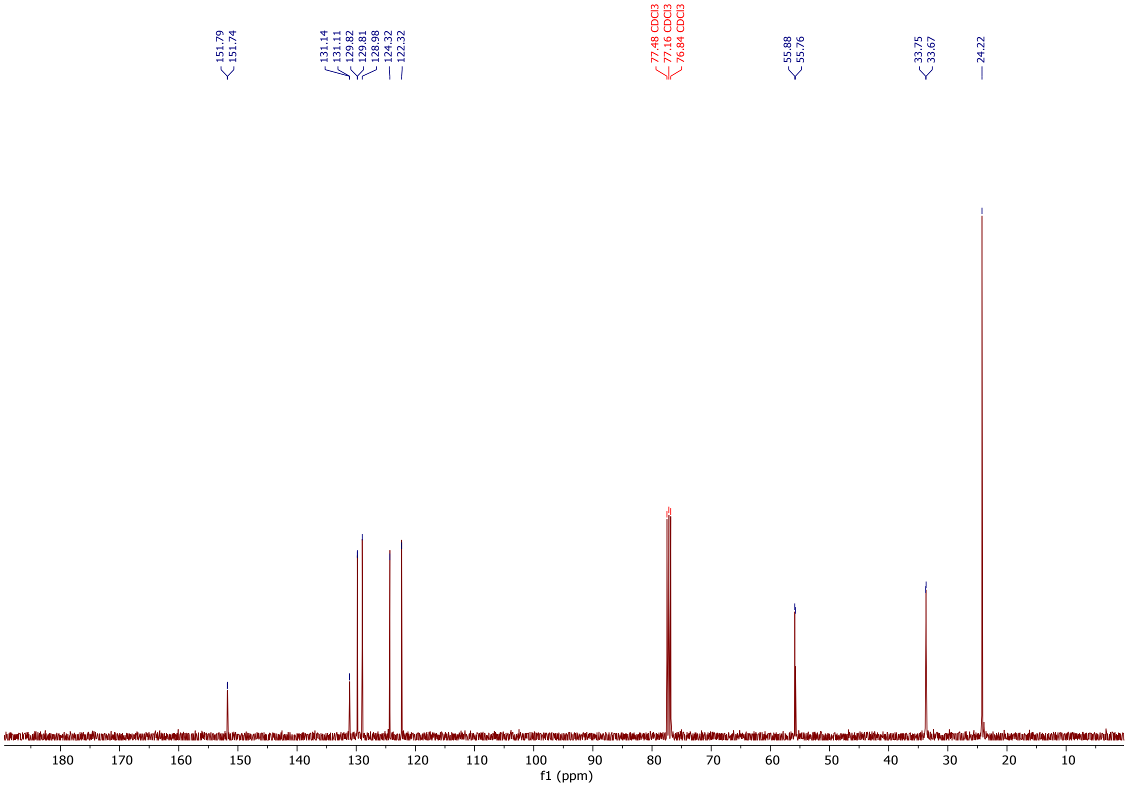

^{13}C NMR (101 MHz, $CDCl_3$) δ 151.79, 151.74, 131.14, 131.11, 129.82, 129.81, 128.98, 124.32, 122.32, 55.88, 55.76, 33.75, 33.67, 24.22.

^{31}P NMR spectrum ($CDCl_3$)

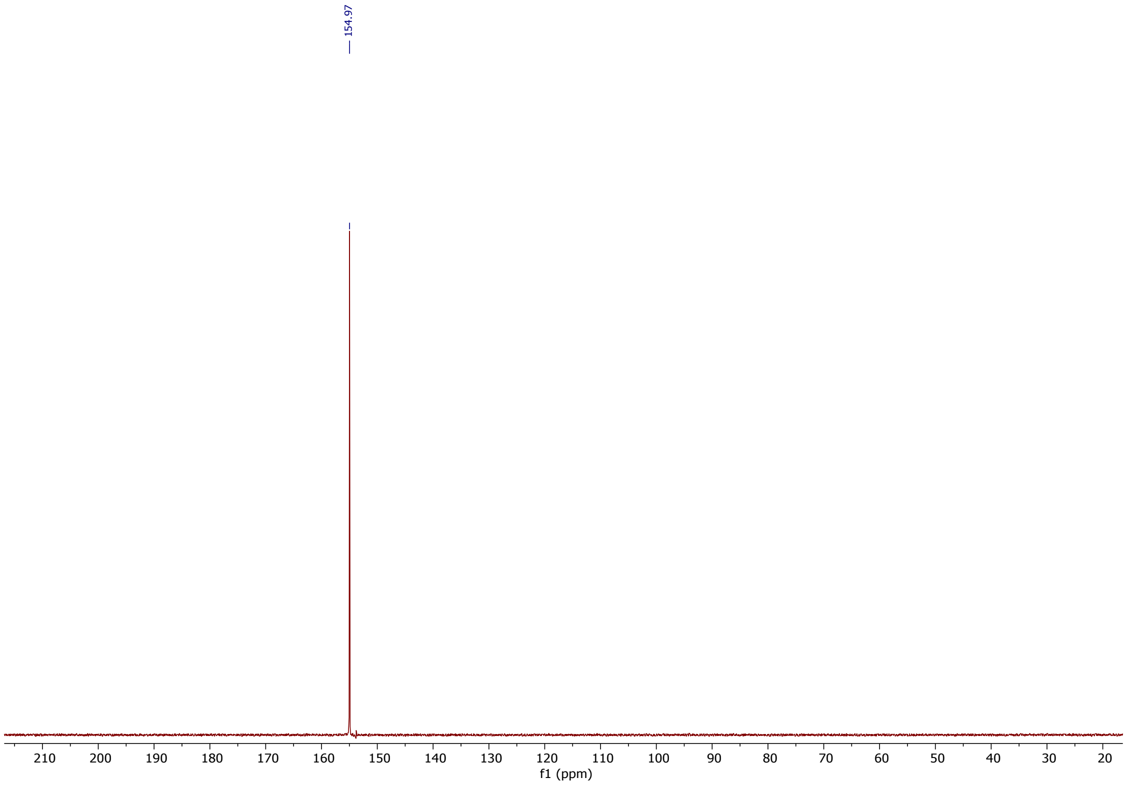

^{31}P NMR (162 MHz, $CDCl_3$) δ 154.97.

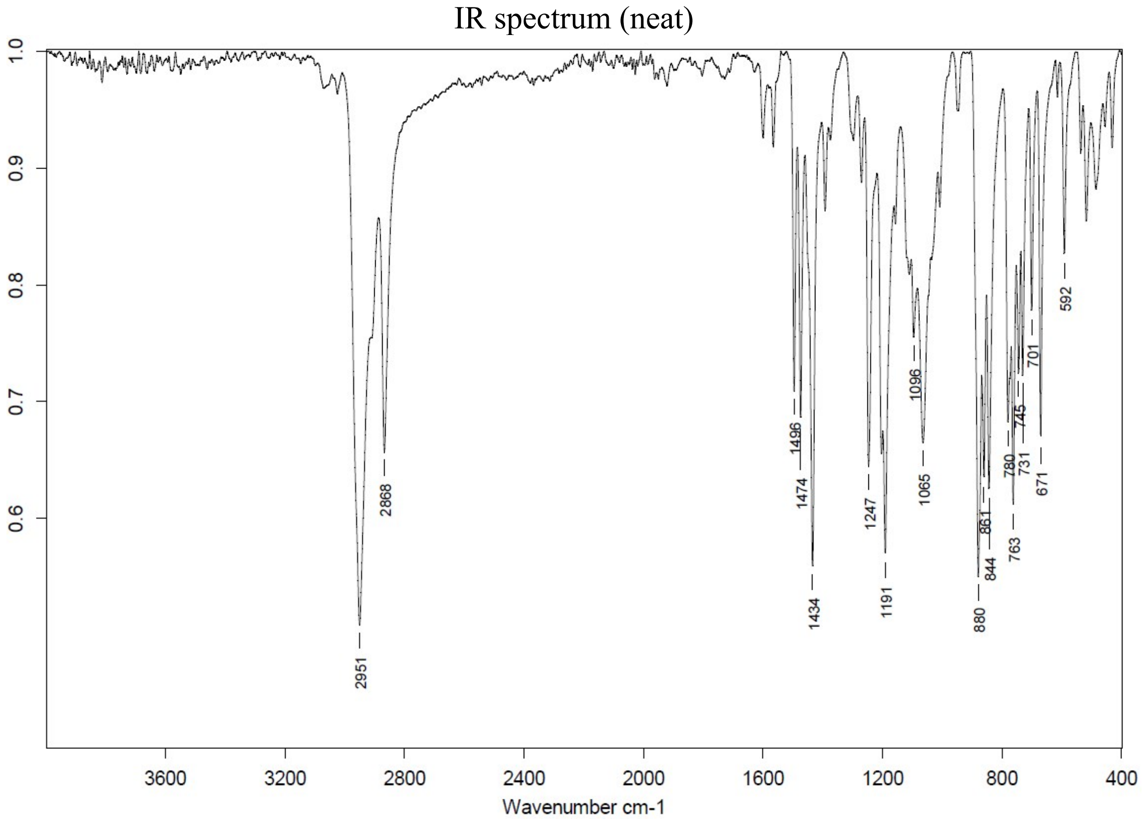

HRMS (ESI$^+$, MeOH): *m/z* calcd. 368.1774 (M + H)$^+$, found: 368.1778.

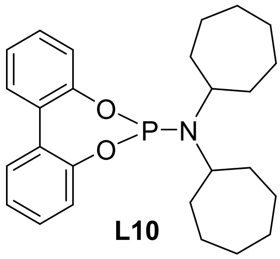

L10

White solid; Column conditions: Hexane : EA = 50 : 1, R_f = 0.30

^{1}H NMR spectrum ($CDCl_3$)

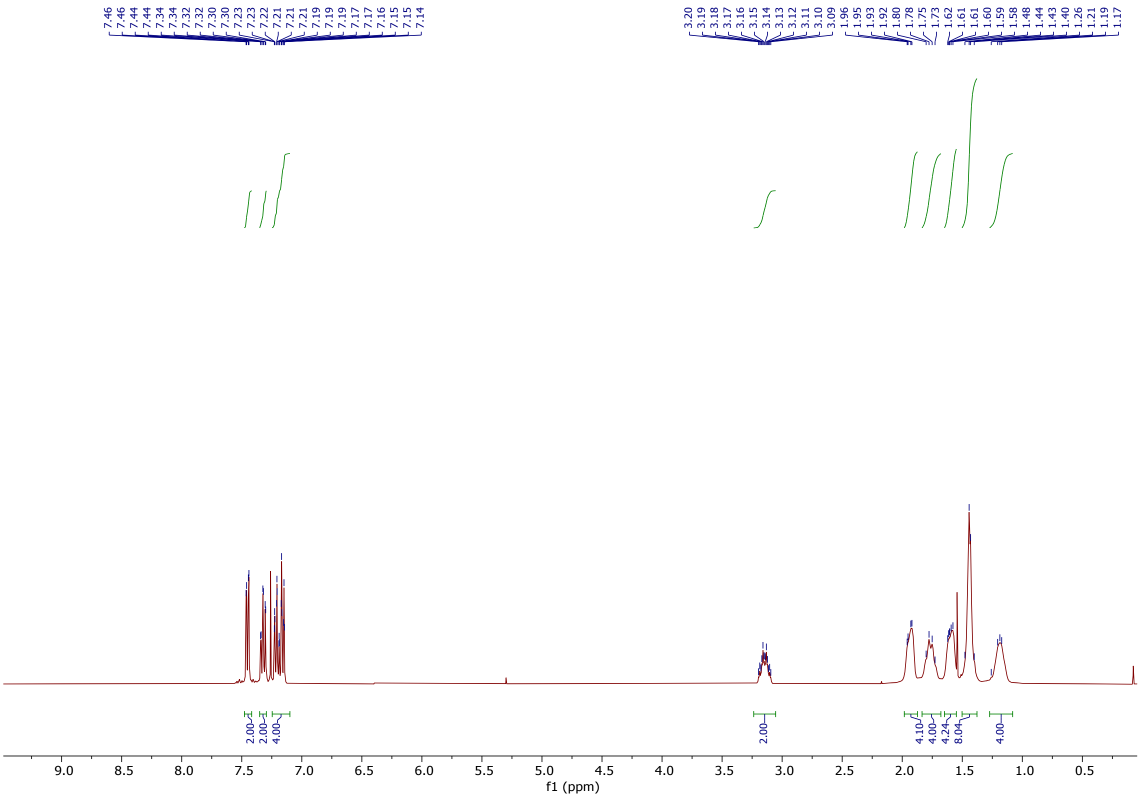

^{1}H NMR (400 MHz, $CDCl_3$) δ 7.45 (dd, *J* = 7.6, 1.8 Hz, 2H), 7.32 (td, *J* = 7.6, 1.7 Hz, 2H), 7.25 – 7.10 (m, 4H), 3.15 (qt, *J* = 11.1, 3.9 Hz, 2H), 1.98 – 1.87 (m, 4H), 1.83 – 1.68 (m, 4H), 1.65 – 1.55 (m, 4H), 1.50 – 1.37 (m, 8H), 1.27 – 1.08 (m, 4H).

^{13}C NMR spectrum ($CDCl_3$)

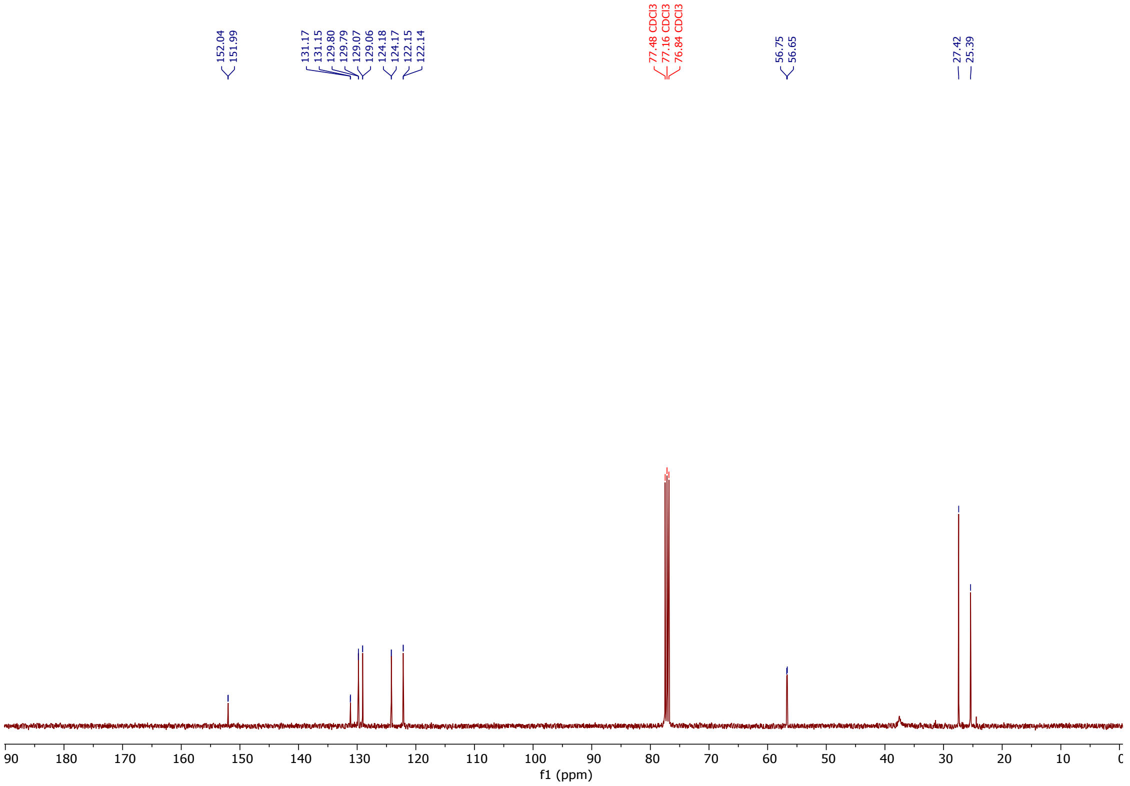

^{13}C NMR (101 MHz, $CDCl_3$) δ 152.04, 151.99, 131.17, 131.15, 129.80, 129.79, 129.07, 129.06, 124.18, 124.17, 122.15, 122.14, 56.75, 56.65, 27.42, 25.39.

^{31}P NMR spectrum ($CDCl_3$)

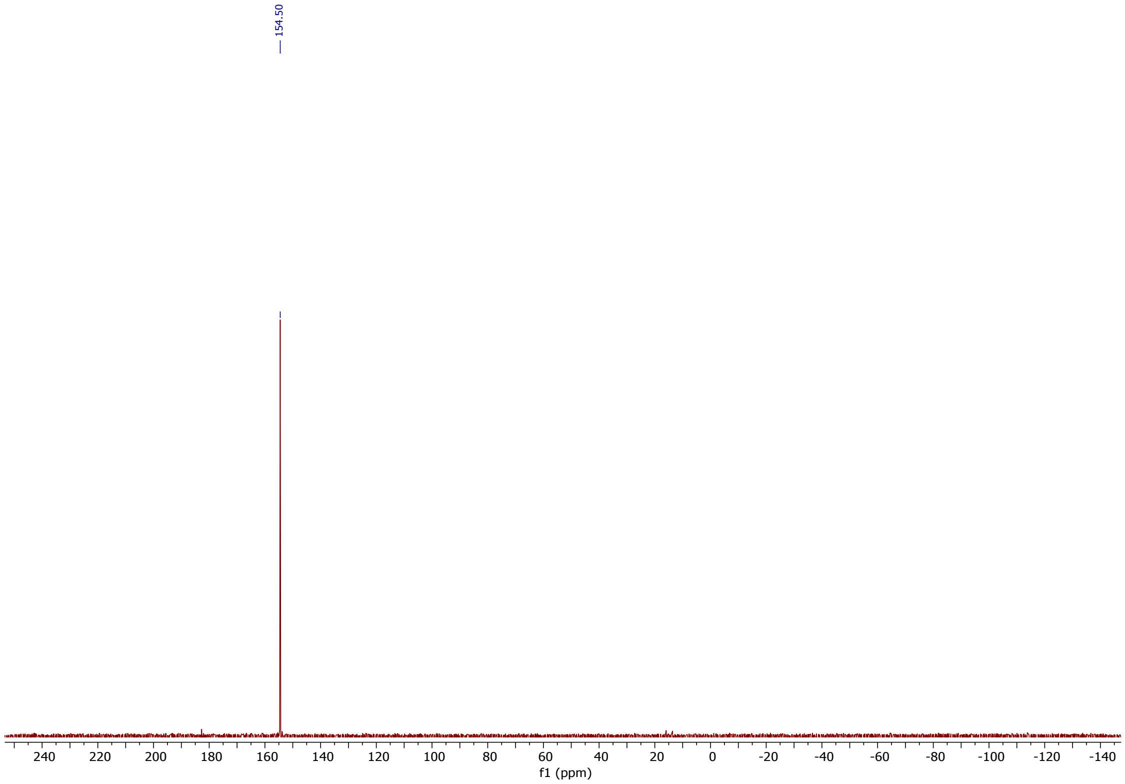

^{31}P NMR (162 MHz, $CDCl_3$) δ 154.50.

IR spectrum (neat)

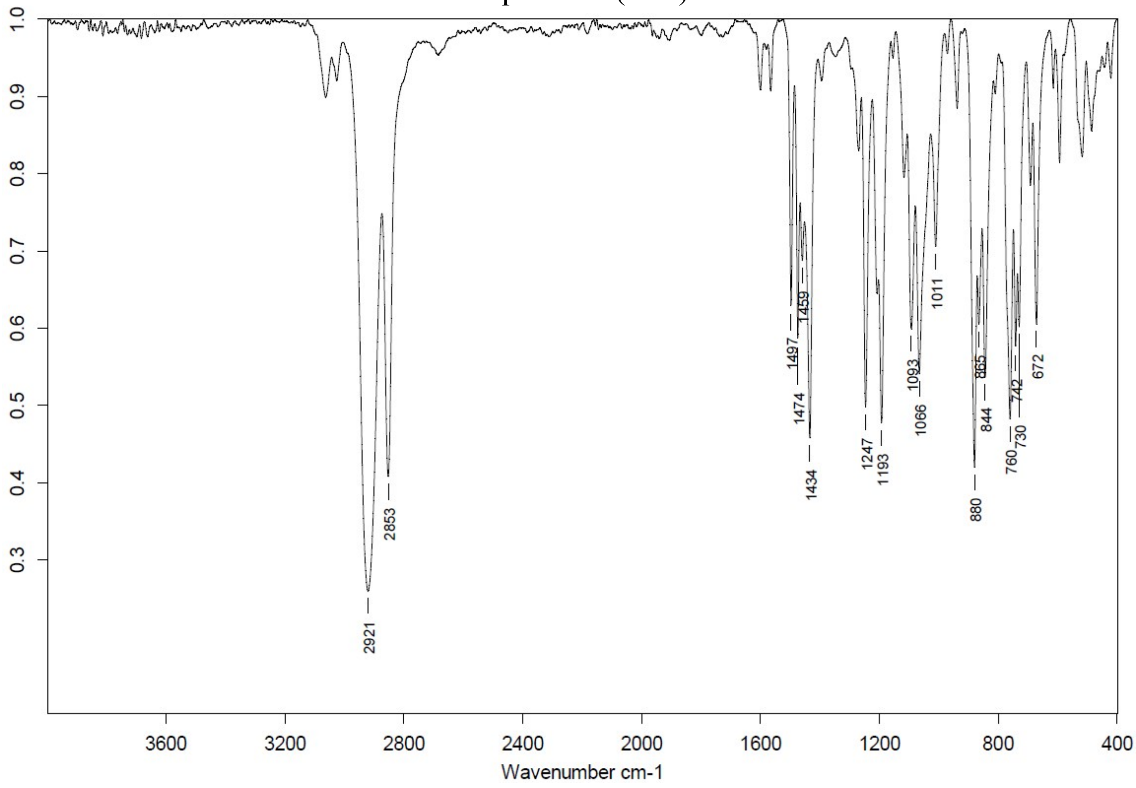

HRMS (ESI^{+}, MeOH): *m/z* calcd. 424.2400 $(M + H)^{+}$, found: 424.2421.

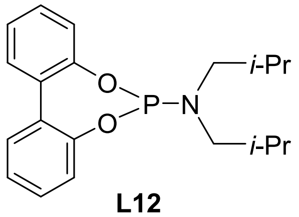

L12

White solid; Column conditions: Hexane : EA = 50 : 1, R_f = 0.33

^{1}H NMR spectrum ($CDCl_3$)

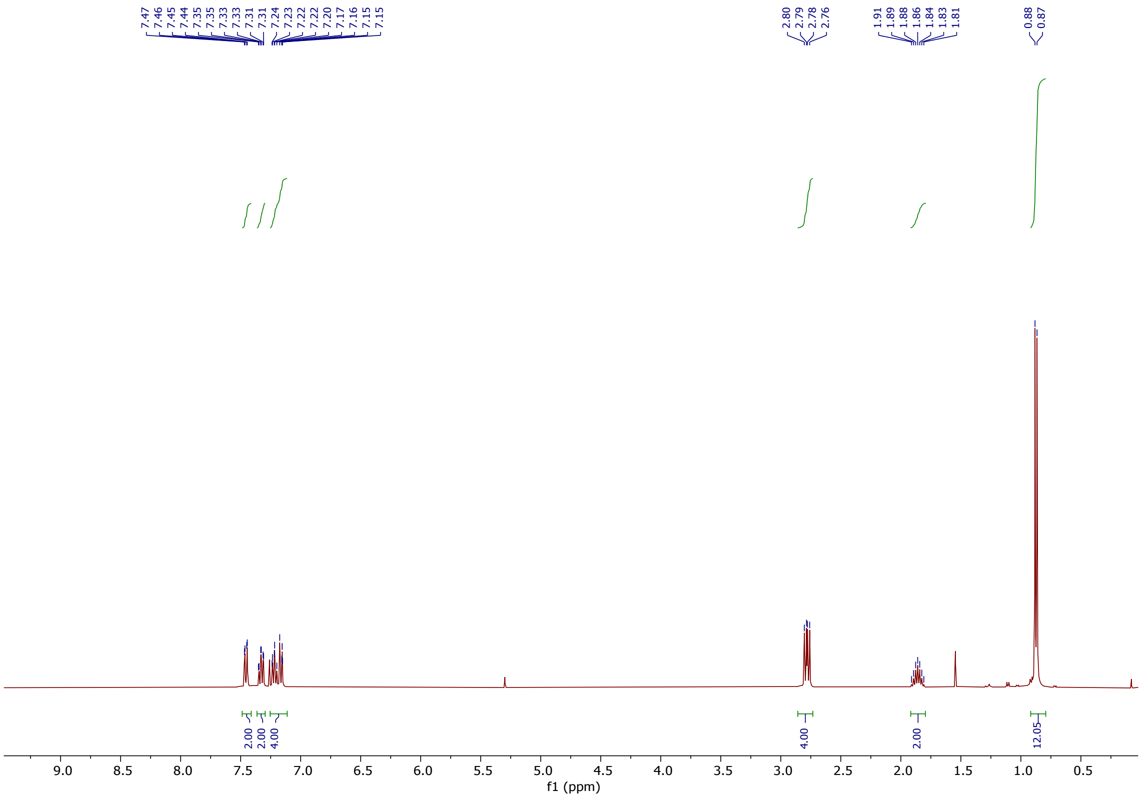

^{1}H NMR (400 MHz, $CDCl_3$) δ 7.46 (dd, J = 7.6, 1.7 Hz, 2H), 7.33 (td, J = 7.7, 1.7 Hz, 2H), 7.25 – 7.11 (m, 4H), 2.78 (dd, J = 10.5, 7.3 Hz, 4H), 1.86 (dp, J = 13.6, 6.8 Hz, 2H), 0.87 (d, J = 6.6 Hz, 12H).

^{13}NMR spectrum ($CDCl_3$)

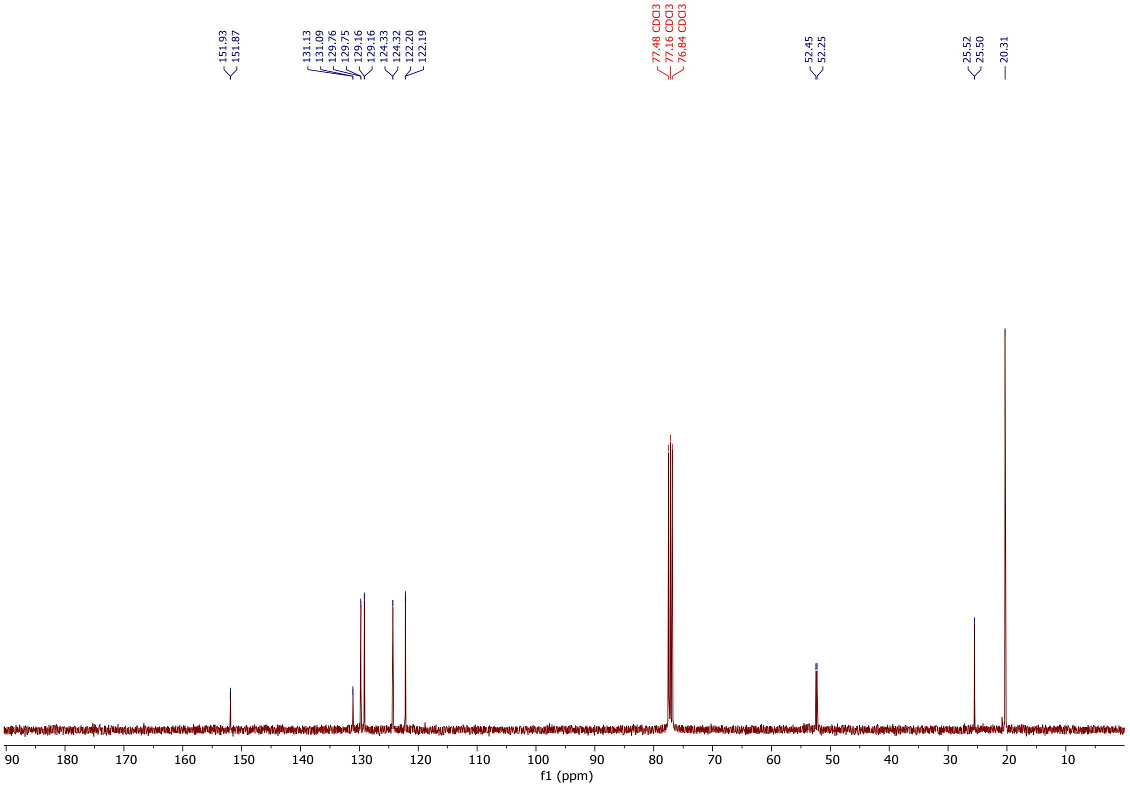

^{13}C NMR (101 MHz, $CDCl_3$) δ 151.93, 151.87, 131.13, 131.09, 129.76, 129.75, 129.16, 129.16, 124.33, 124.32, 122.20, 122.19, 52.45, 52.25, 25.52, 25.50, 20.31.

^{31}P NMR spectrum ($CDCl_3$)

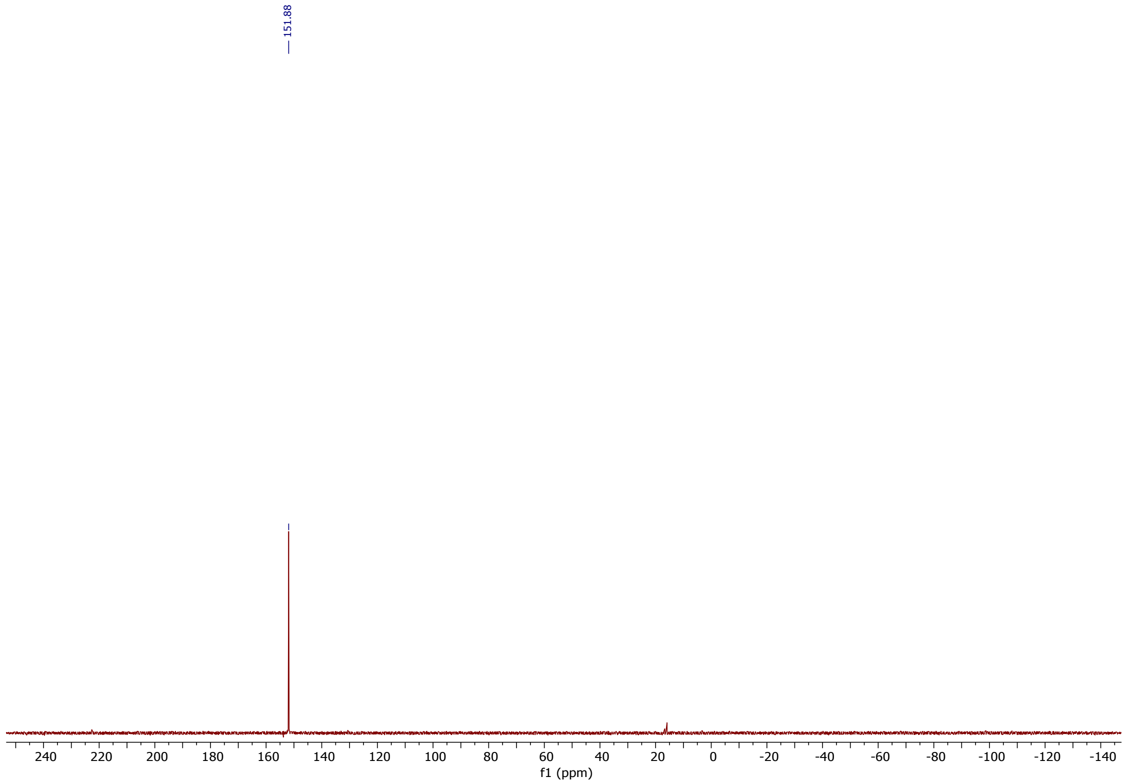

^{31}P NMR (162 MHz, $CDCl_3$) δ 151.88.

IR spectrum (neat)

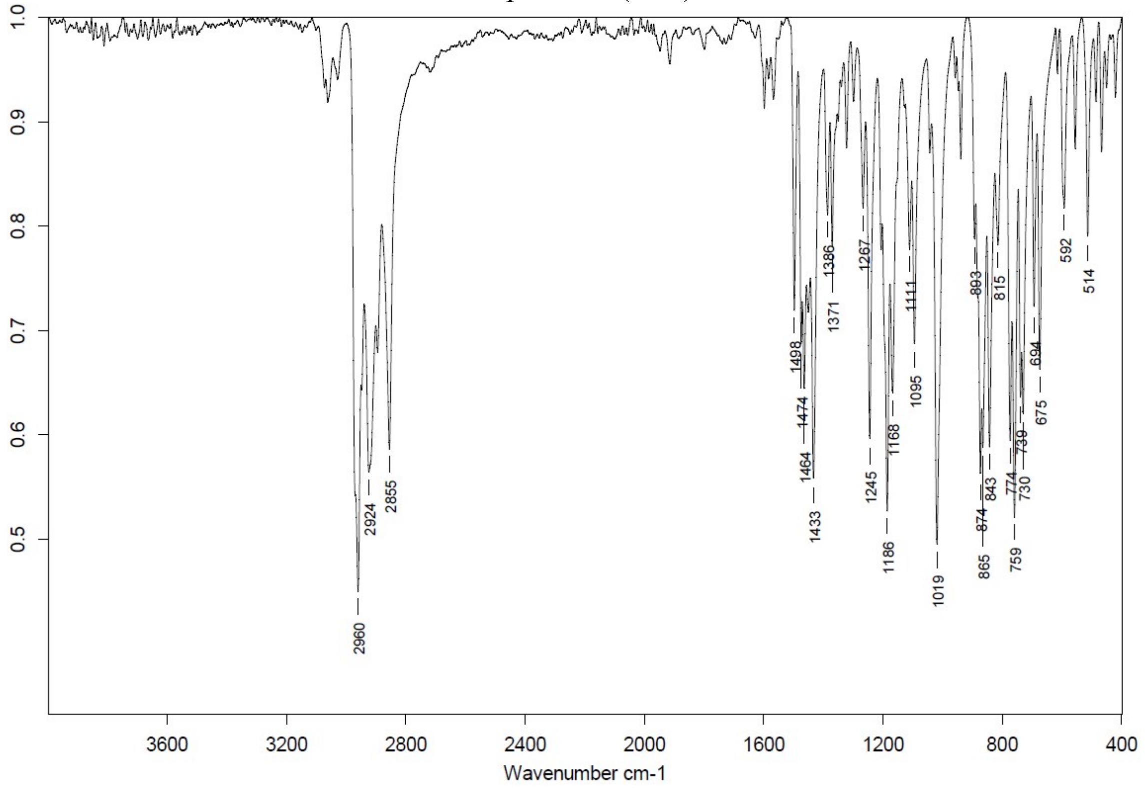

HRMS (ESI$^+$, MeOH): *m/z* calcd. 344.1774 $(M + H)^+$, found: 344.1776.

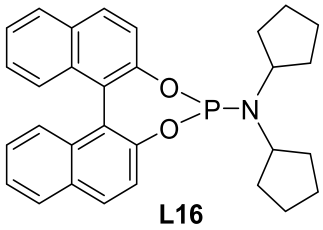

White solid; Column conditions: Hexane : EA = 50 : 1, R_f = 0.25

^{1}H NMR spectrum ($CDCl_3$)

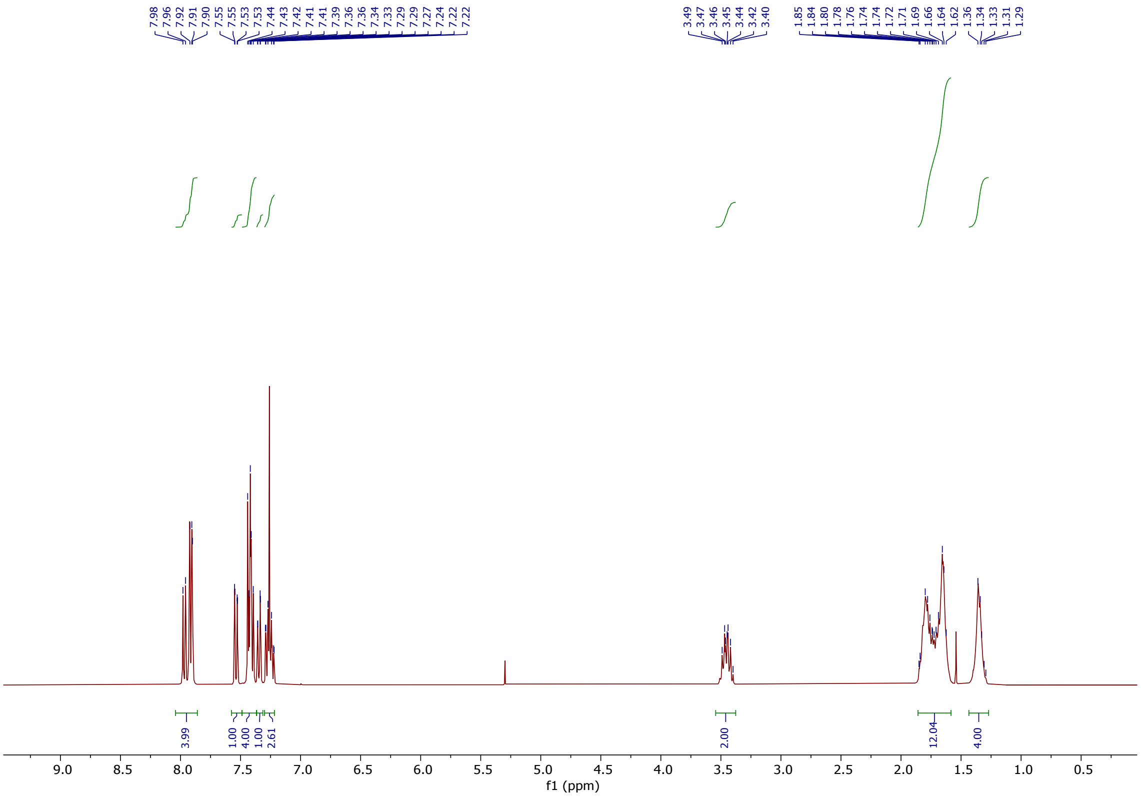

^{1}H NMR (400 MHz, $CDCl_3$) δ 8.04 – 7.86 (m, 4H), 7.54 (dd, J = 8.7, 1.1 Hz, 1H), 7.49 – 7.37 (m, 4H), 7.37 – 7.31 (m, 1H), 7.30 – 7.22 (m, 3H), 3.54 – 3.38 (m, 2H), 1.86 – 1.58 (m, 12H), 1.43 – 1.27 (m, 4H).

^{13}NMR spectrum ($CDCl_3$)

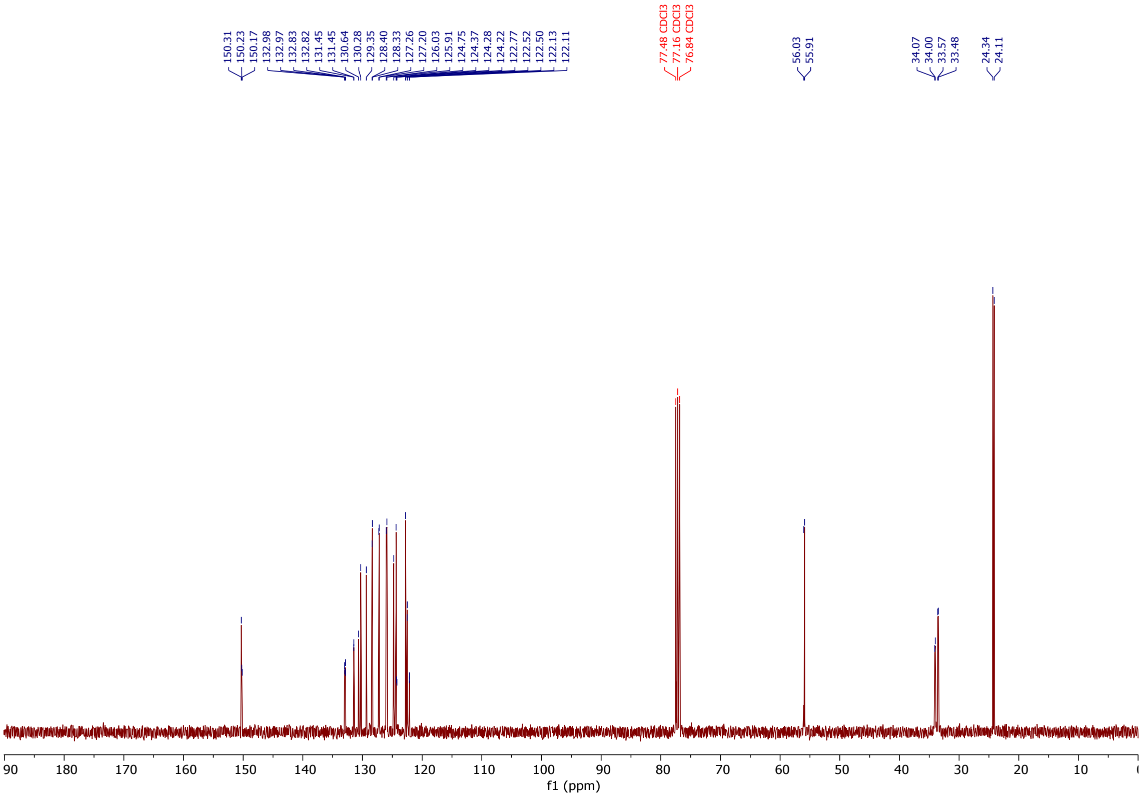

^{13}C NMR (101 MHz, $CDCl_3$) δ 150.31, 150.23, 150.17, 132.98, 132.97, 132.83, 132.82, 131.45, 131.45, 130.64, 130.28, 129.35, 128.40, 128.33, 127.26, 127.20, 126.03,125.91, 124.75, 124.37, 124.28, 124.22, 122.77, 122.52, 122.50, 122.13, 122.11, 56.03, 55.91, 34.07, 34.00, 33.57, 33.48, 24.34, 24.11.

^{31}P NMR spectrum ($CDCl_3$)

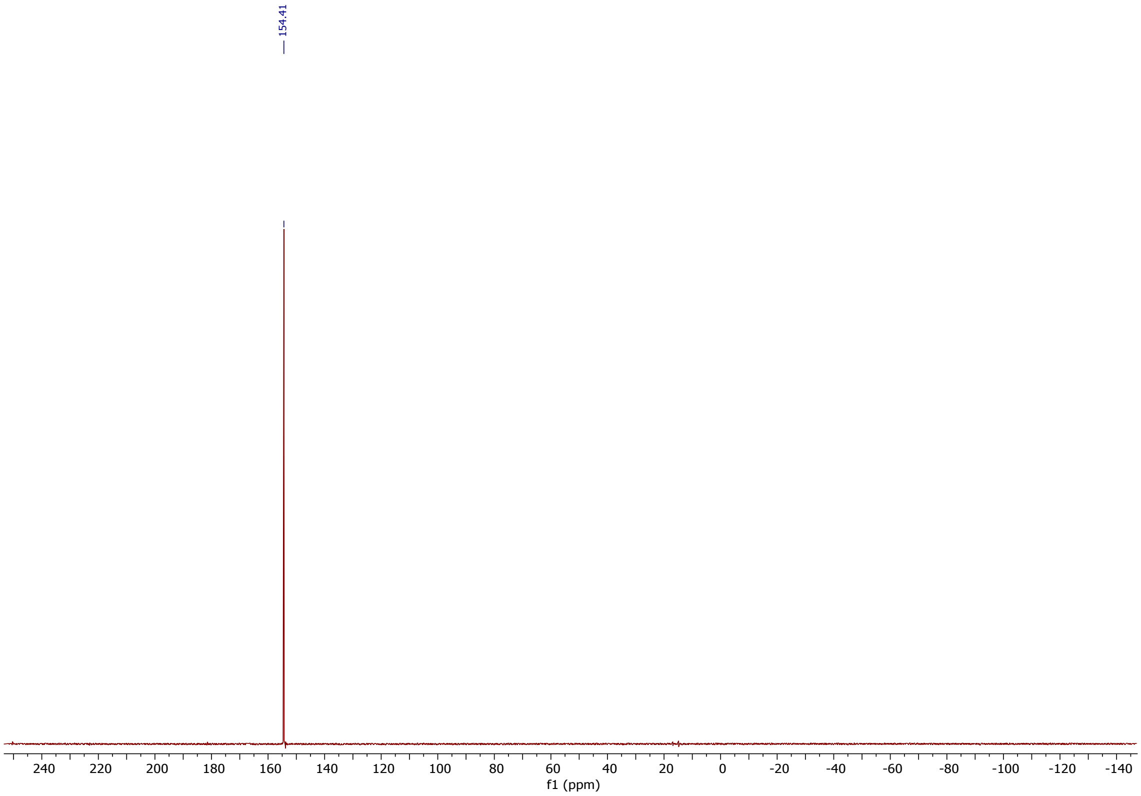

^{31}P NMR (162 MHz, $CDCl_3$) δ 154.41.

IR spectrum (neat)

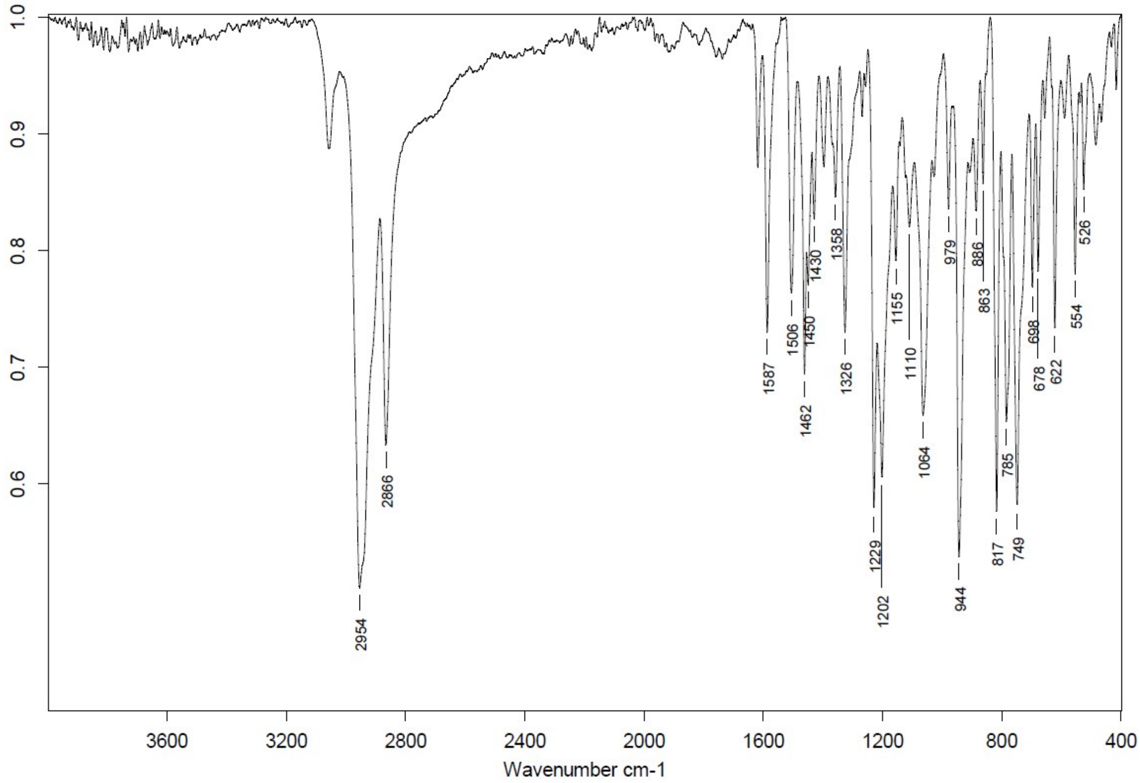

HRMS (ESI$^+$, MeOH): *m/z* calcd. 468.2087 $(M + H)^+$, found: 468.2087.

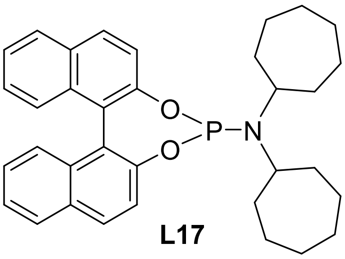

L17

White solid; Column conditions: Hexane : EA = 50 : 1, R_f = 0.25

^{1}H NMR spectrum ($CDCl_3$)

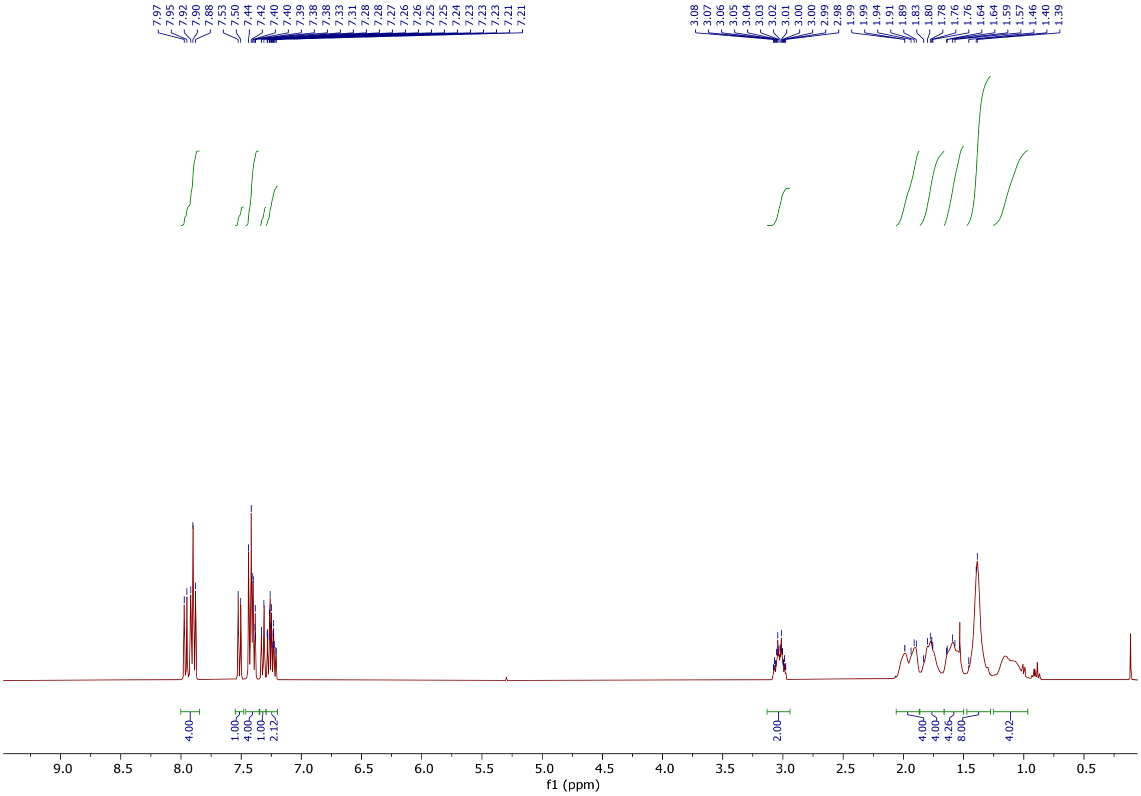

^{1}H NMR (400 MHz, $CDCl_3$) δ 8.00 – 7.84 (m, 4H), 7.51 (d, J = 8.8 Hz, 1H), 7.46 – 7.35 (m, 4H), 7.32 (d, J = 7.7 Hz, 1H), 7.29 – 7.19 (m, 2H), 3.03 (qt, J = 10.8, 3.8 Hz, 2H), 2.06 – 1.87 (m, 4H), 1.86 – 1.66 (m, 4H), 1.66 – 1.50 (m, 4H), 1.47 – 1.28 (m, 8H), 1.25 – 0.97 (m, 4H).

^{13}NMR spectrum ($CDCl_3$)

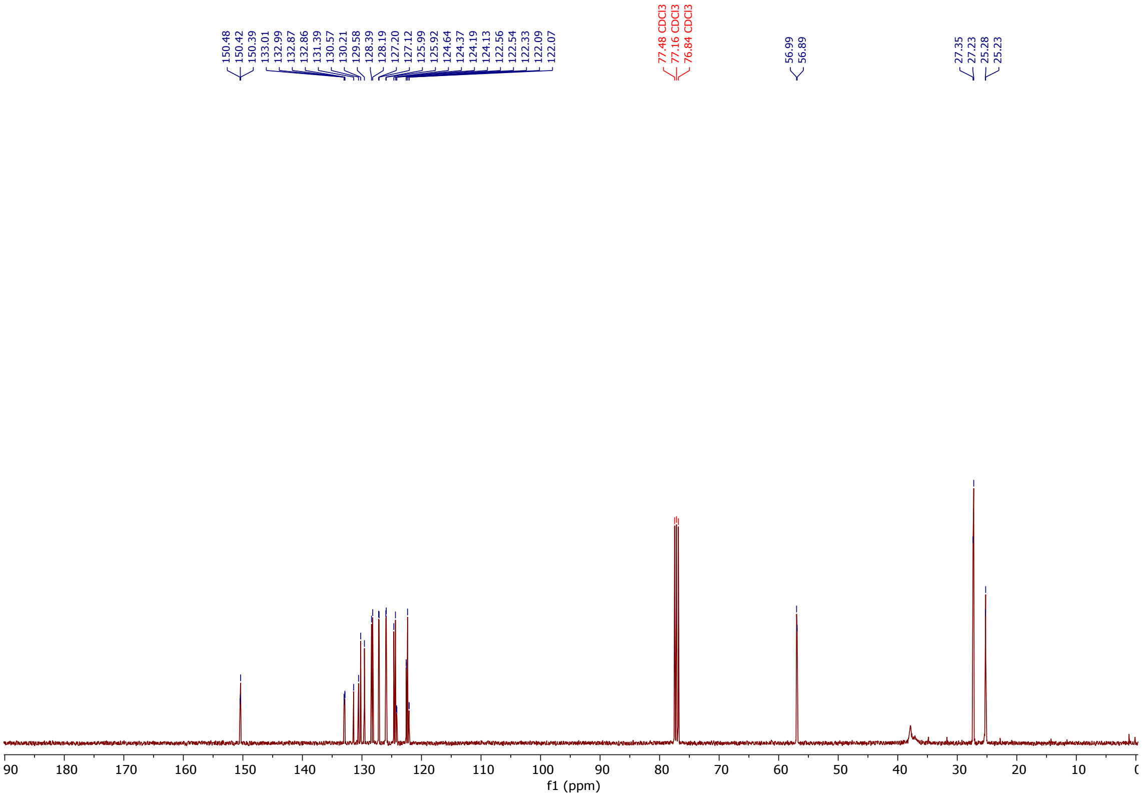

^{13}C NMR (101 MHz, $CDCl_3$) δ 150.48, 150.42, 150.39, 133.01, 132.99, 132.87, 132.86, 131.39, 130.57, 130.21, 129.58, 128.39, 128.19, 127.20, 127.12, 125.99, 125.92, 124.64, 124.37, 124.19, 124.13, 122.56, 122.54, 122.33, 122.09, 122.07, 56.99, 56.89, 27.35, 27.23, 25.28, 25.23.

^{31}P NMR spectrum ($CDCl_3$)

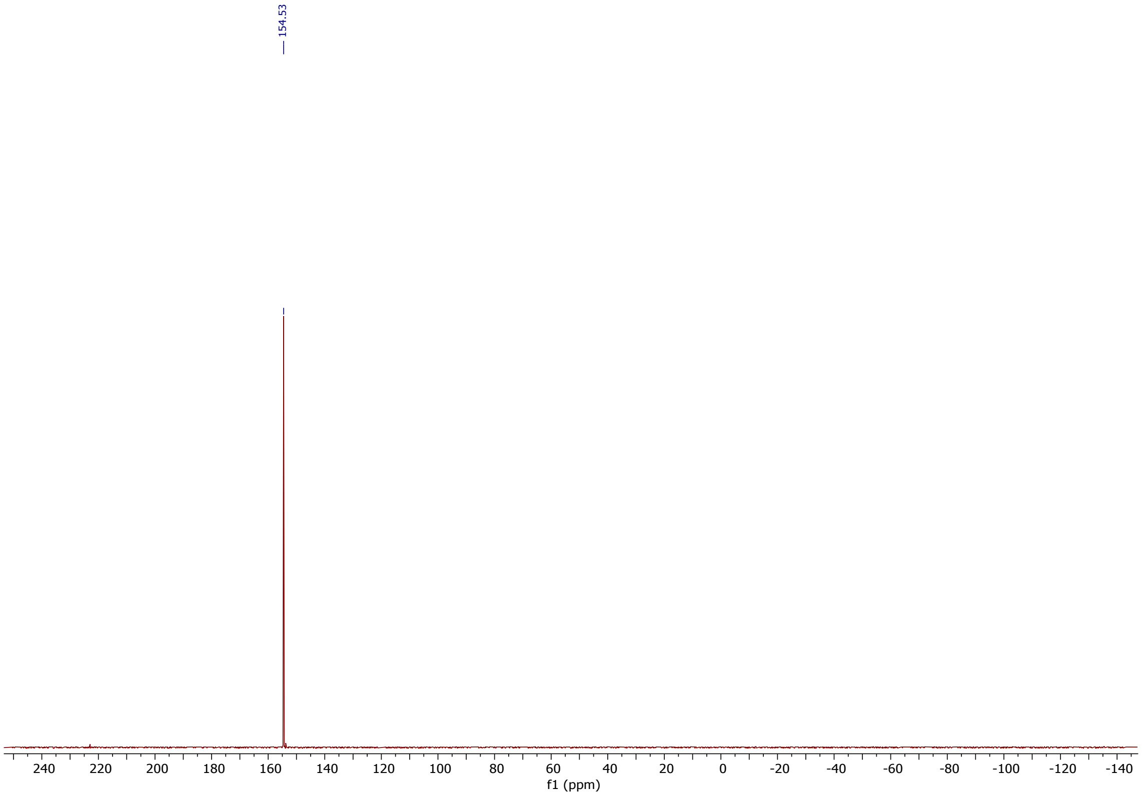

^{31}P NMR (162 MHz, $CDCl_3$) δ 154.53.

IR spectrum (neat)

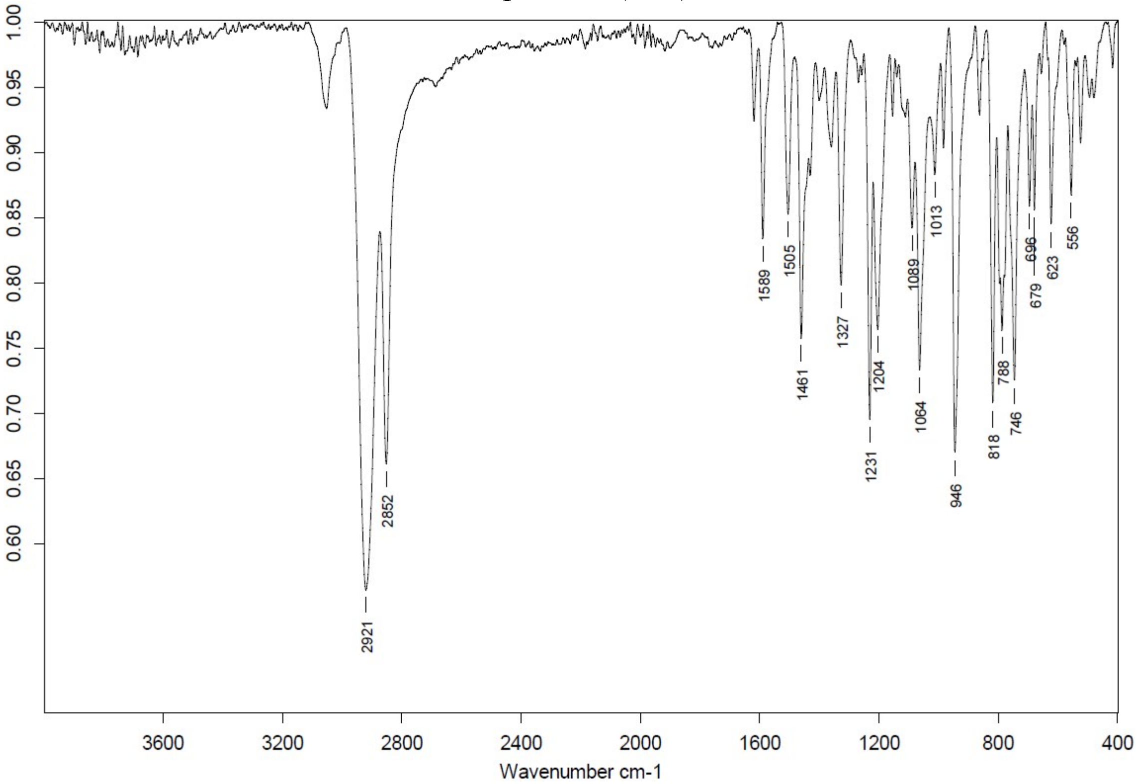

HRMS (ESI$^+$, MeOH): *m/z* calcd. 524.2713 (M + H)$^+$, found: 524.2720.

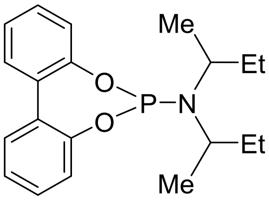

L19

Colorless oil; Column conditions: Hexane : EA = 50 : 1, R_f = 0.35

^{1}H NMR spectrum ($CDCl_3$)

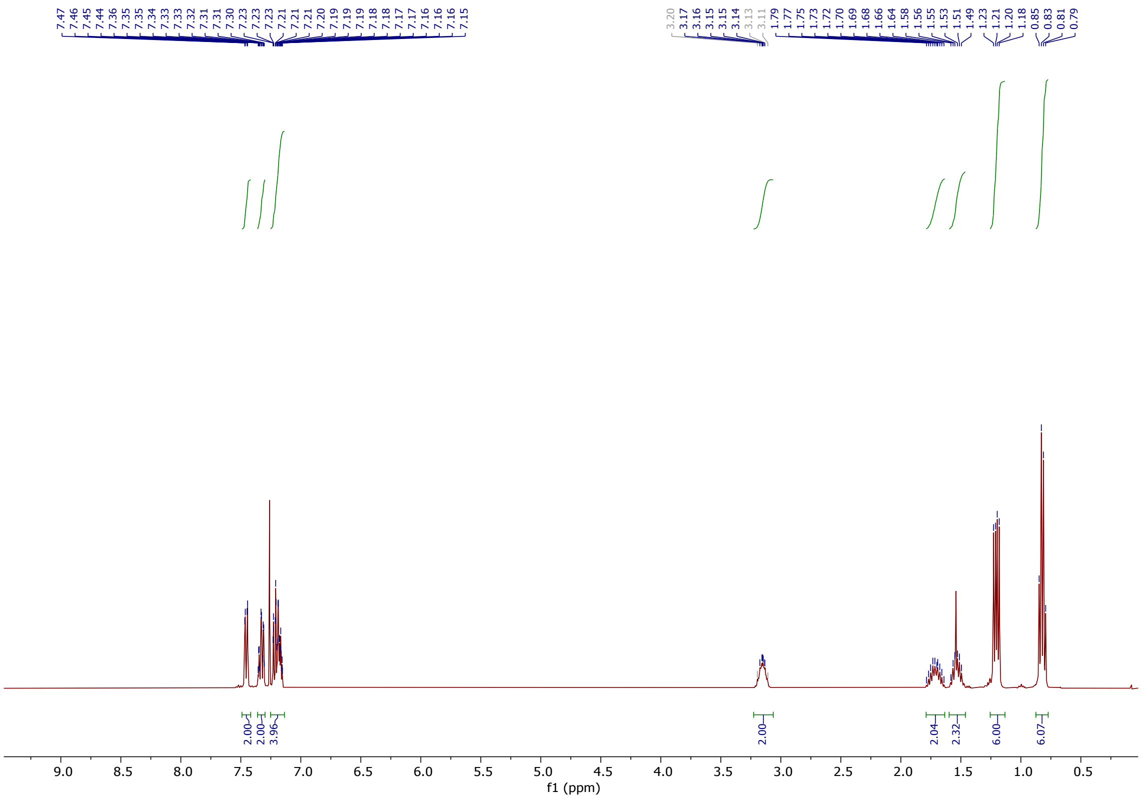

^{1}H NMR (400 MHz, $CDCl_3$) δ 7.46 (dd, J = 7.6, 1.6 Hz, 2H), 7.36 – 7.30 (m, 2H), 7.25 – 7.14 (m, 4H), 3.23 – 3.06 (m, 2H), 1.78 – 1.63 (m 2H), 1.60 – 1.46 (m, 2H), 1.22 – 1.29 (m, 6H), 0.87 – 0.74 (m, 6H).

^{13}NMR spectrum ($CDCl_3$)

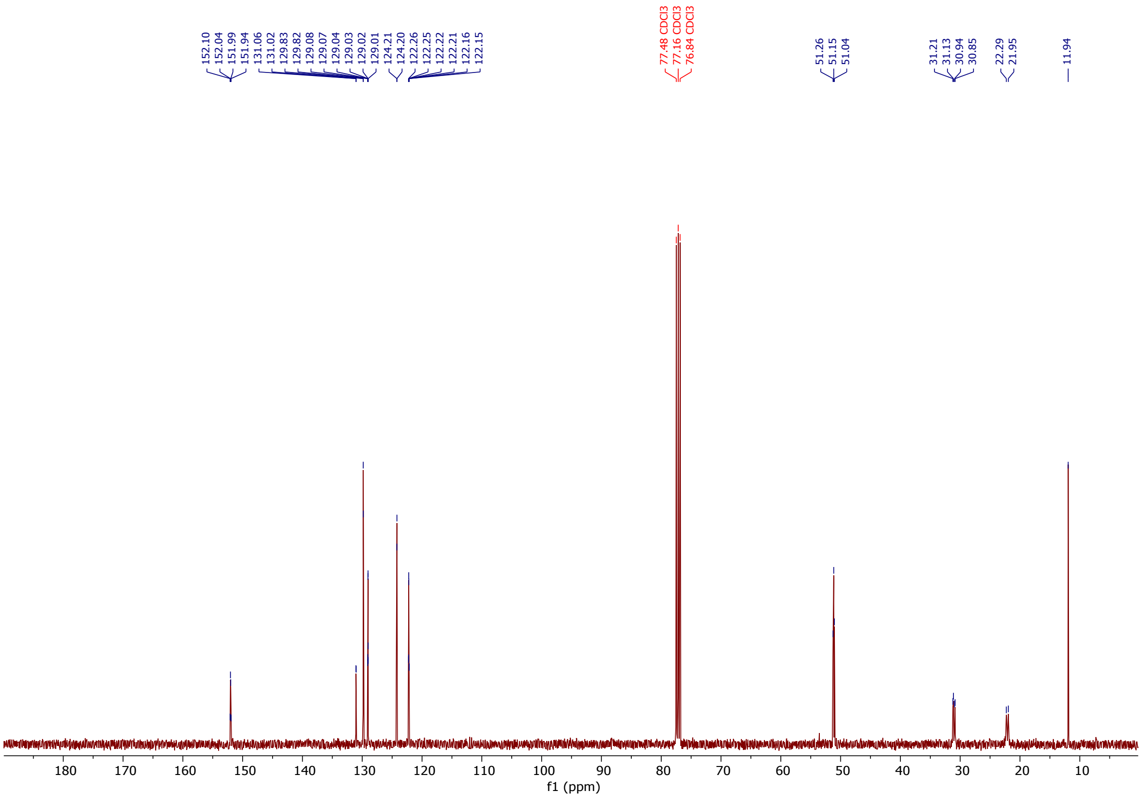

^{13}C NMR (101 MHz, $CDCl_3$) δ 152.10, 152.04, 151.99, 151.94, 131.06, 131.02, 129.83, 129.82, 129.08, 129.07, 129.04, 129.03, 129.02, 129.01, 124.21, 124.20, 122.26, 122.25, 122.22, 122.21, 122.16, 122.15, 51.26, 51.15, 51.04, 31.21, 31.13, 30.94, 30.85, 22.29, 21.95, 11.94.

^{31}P NMR spectrum ($CDCl_3$)

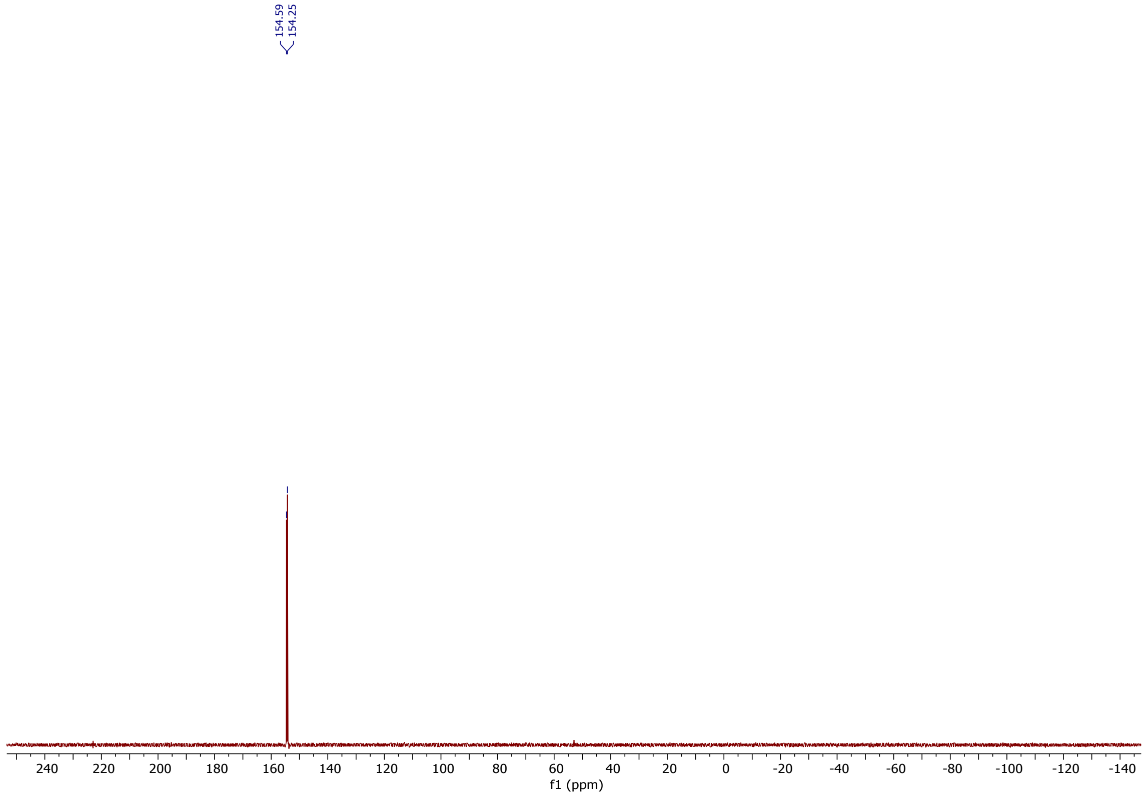

^{31}P NMR (162 MHz, $CDCl_3$) δ 154.59, 154.25.

IR spectrum (neat)

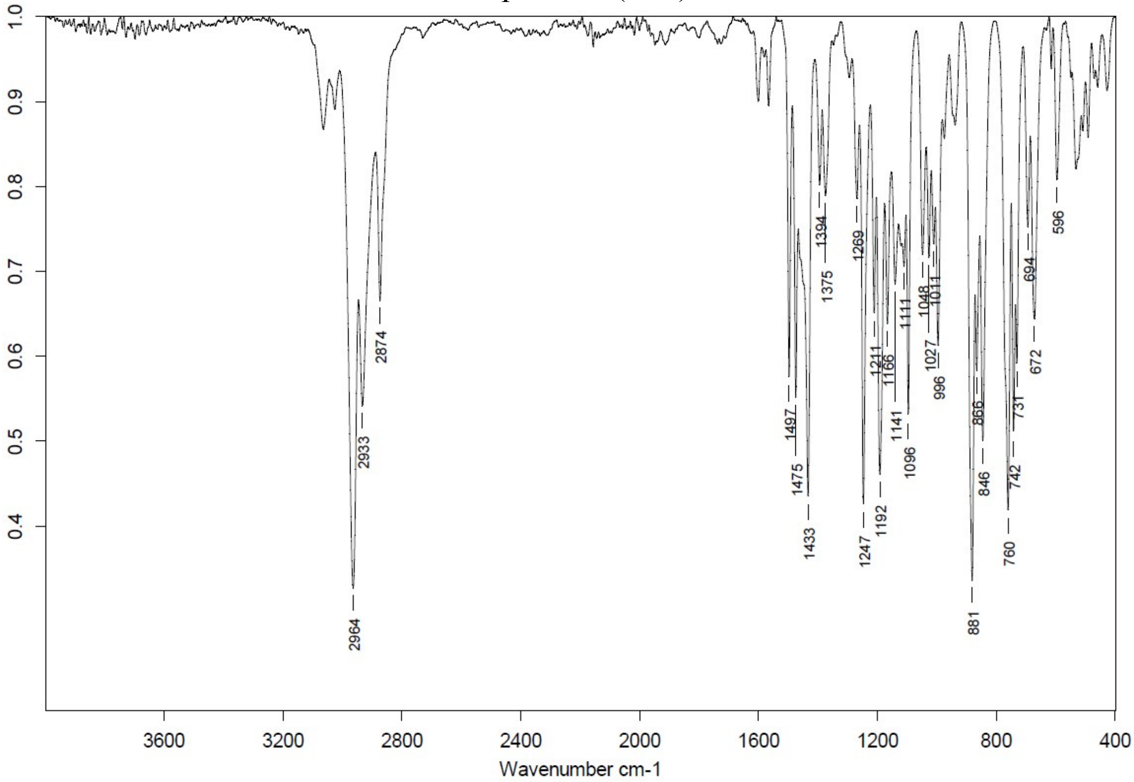

HRMS (ESI^+, MeOH): *m/z* calcd. 344.1774 $(M + H)^+$, found: 344.1773.

S32. IR, NMR spectra, HRMS data for vinyl γ-lactones

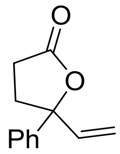

General procedure **A**: Colorless oil; Column conditions: Hexane : EA = 20 : 1, $R_f = 0.18$

^{1}H NMR spectrum ($CDCl_3$)

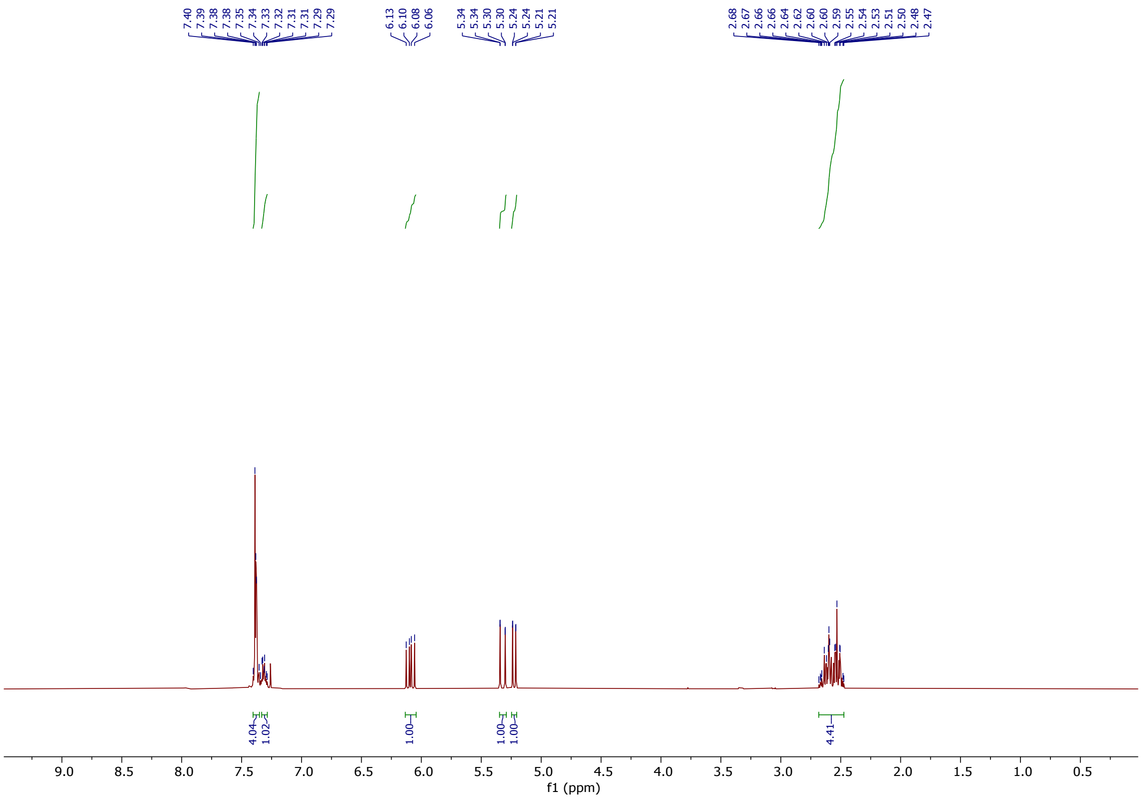

^{1}H NMR (400 MHz, $CDCl_3$) δ 7.40 – 7.35 (m, 4H), 7.33 – 7.29 (m, 1H), 6.09 (dd, J = 17.2, 10.7 Hz, 1H), 5.32 (dd, J = 17.1, 0.7 Hz, 1H), 5.23 (dd, J = 10.7, 0.7 Hz, 1H), 2.68 – 2.47 (m, 4H).

^{13}C NMR spectrum ($CDCl_3$)

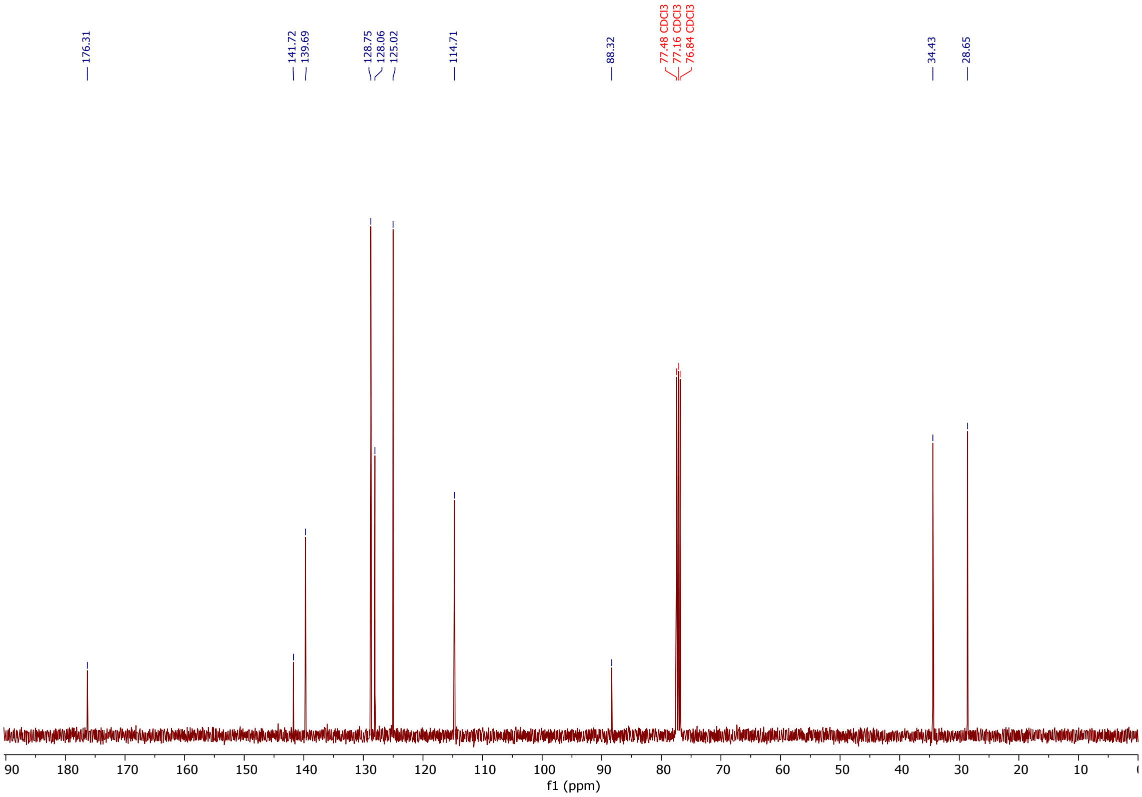

^{13}C NMR (101 MHz, $CDCl_3$) δ 176.31, 141.72, 139.69, 128.75, 128.06, 125.02, 114.71, 88.32, 34.43, 28.65.[5]

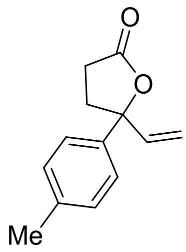

General procedure **A**: Colorless solid; Column conditions: Hexane : EA = 20 : 1, R_f = 0.18

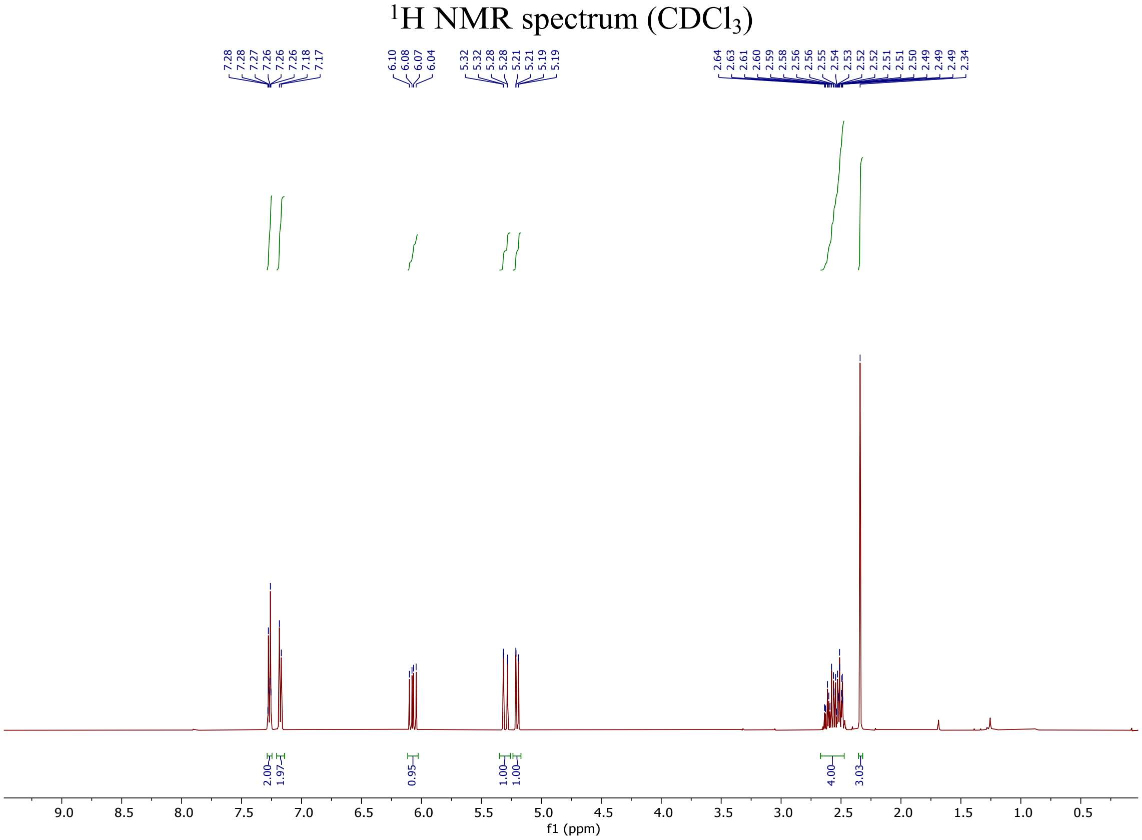

^{1}H NMR (500 MHz, $CDCl_3$) δ 7.29 – 7.25 (m, 2H), 7.18 (d, J = 7.8 Hz, 2H), 6.07 (dd, J = 17.1, 10.7 Hz, 1H), 5.30 (dd, J = 17.1, 0.8 Hz, 1H), 5.20 (dd, J = 10.7, 0.8 Hz, 1H), 2.67 – 2.47 (m, 4H), 2.34 (s, 3H).

^{13}C NMR spectrum ($CDCl_3$)

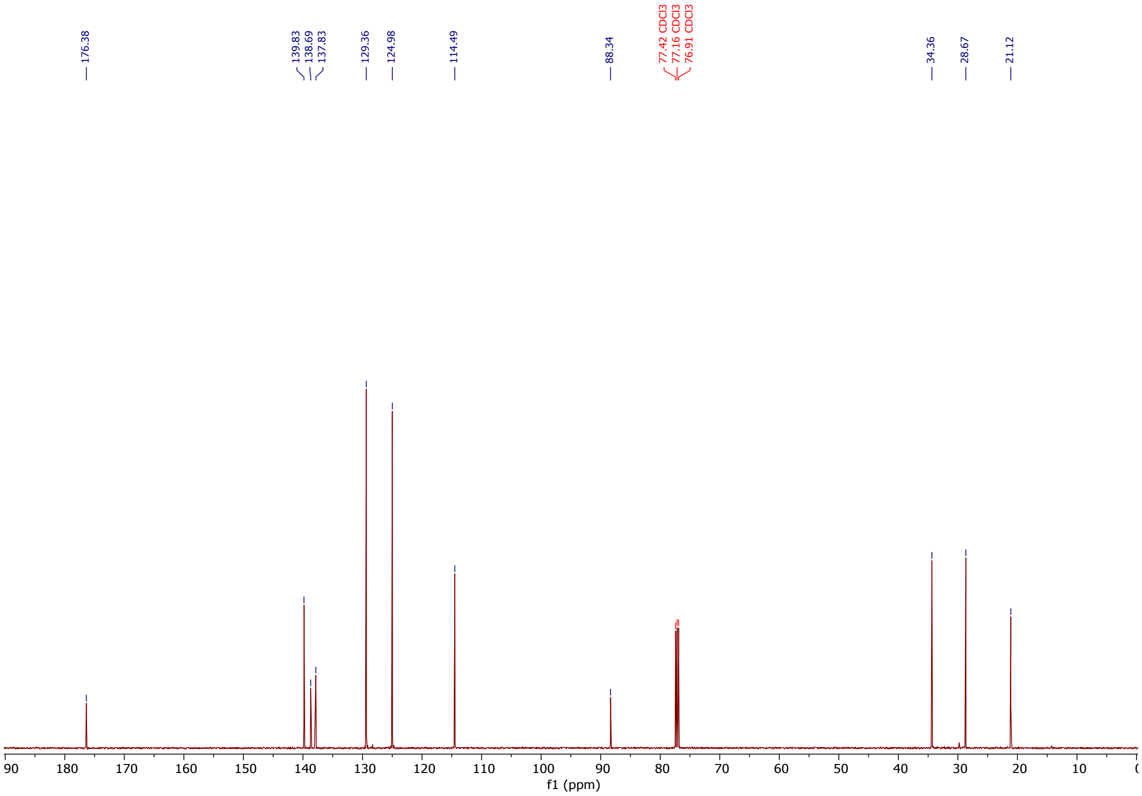

^{13}C NMR (126 MHz, $CDCl_3$) δ 176.38, 139.83, 138.69, 137.83, 129.36, 124.98, 114.49, 88.34, 34.36, 28.67, 21.12.

IR spectrum (neat)

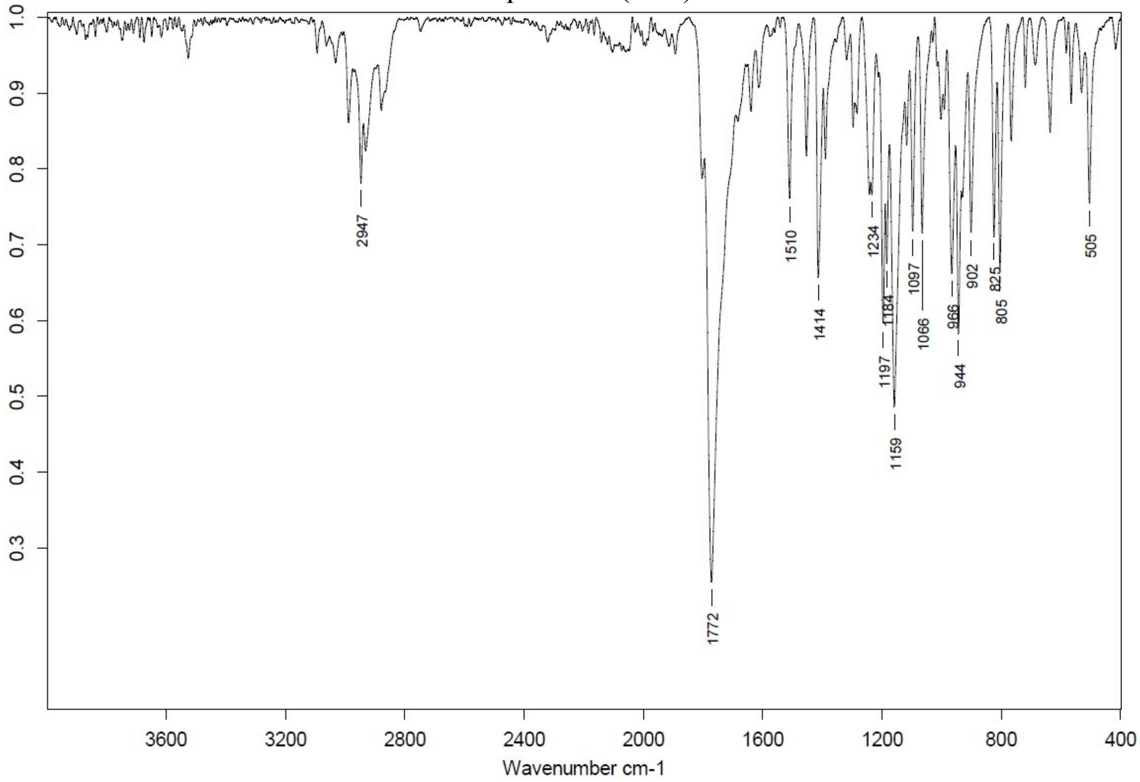

HRMS (ESI$^+$, MeOH): *m/z* calcd. 203.1067 $(M + H)^+$, found: 203.1068.

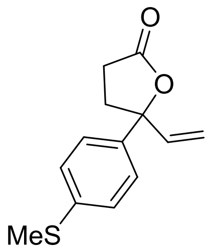

General procedure **A**: Yellow oil; Column conditions: Hexane : EA = 20 : 1, R_f = 0.15

^{1}H NMR spectrum ($CDCl_3$)

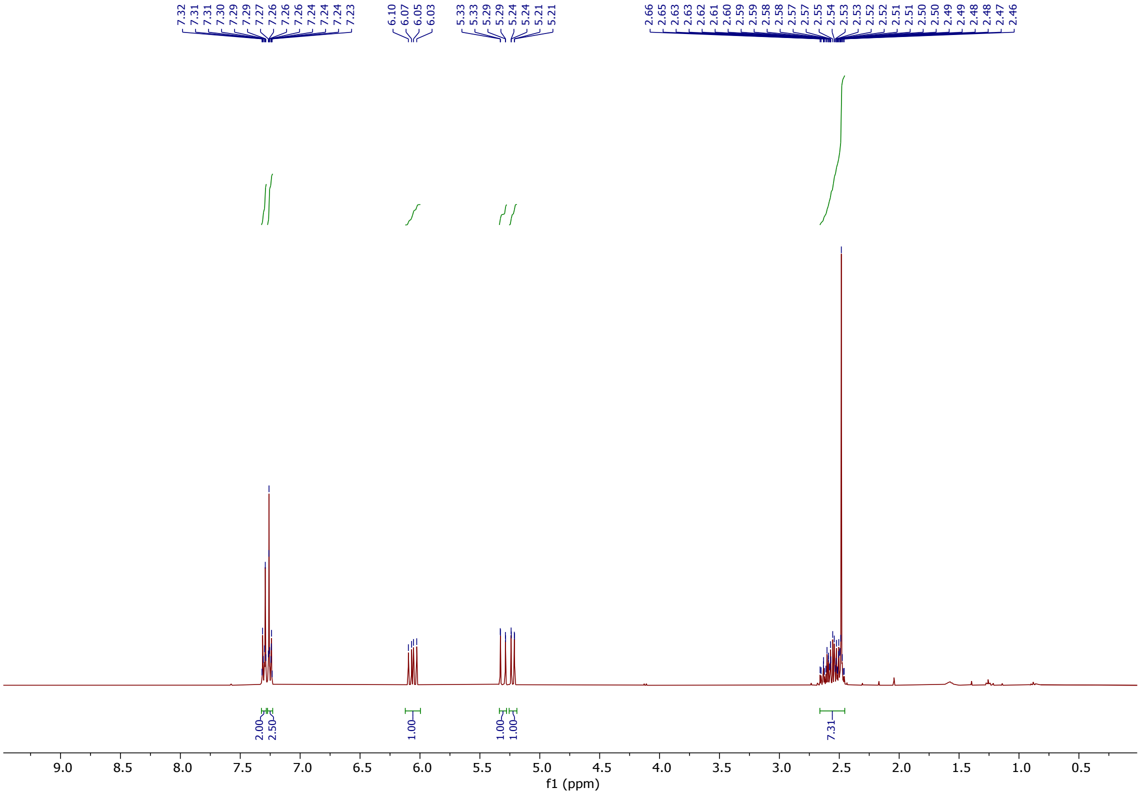

^{1}H NMR (400 MHz, $CDCl_3$) δ 7.32 – 7.28 (m, 2H), 7.27 – 7.23 (m, 3H), 6.06 (dd, J = 17.1, 10.7 Hz, 1H), 5.31 (dd, J = 17.2, 0.7 Hz, 1H), 5.22 (dd, J = 10.7, 0.7 Hz, 1H), 2.66 – 2.45 (m, 7H).

^{13}C NMR spectrum ($CDCl_3$)

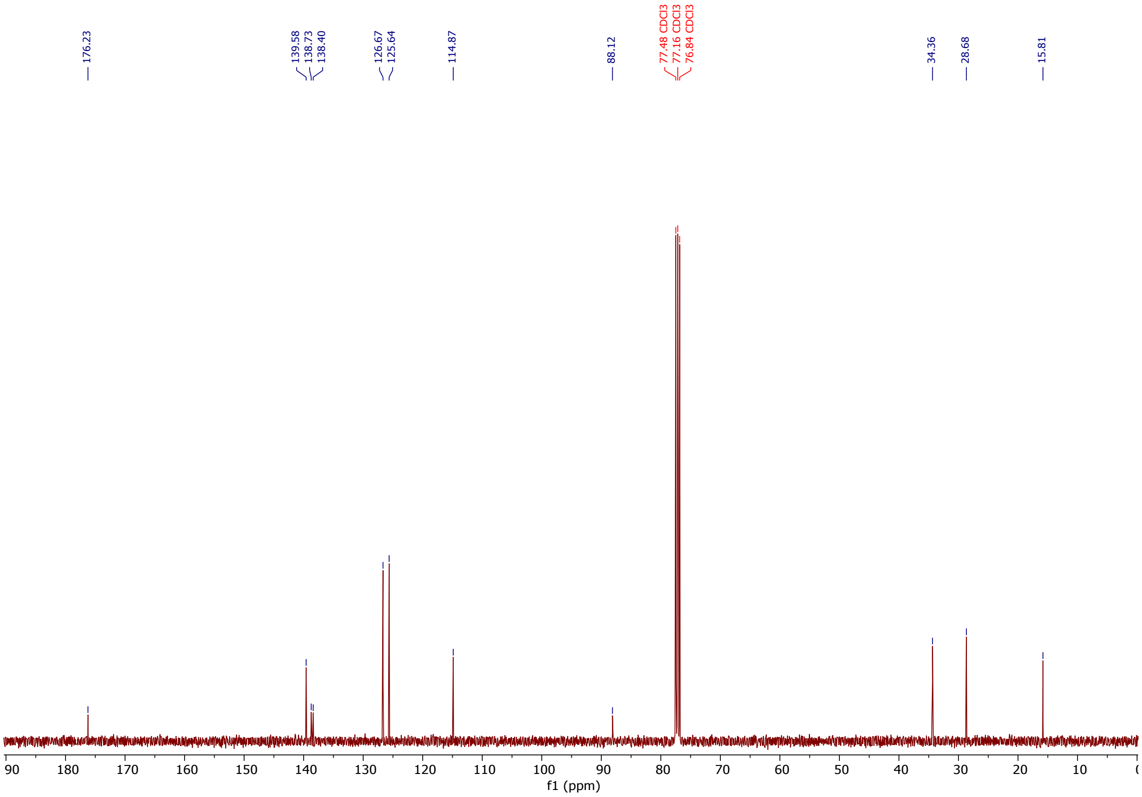

^{13}C NMR (101 MHz, $CDCl_3$) δ 176.23, 139.58, 138.73, 138.40, 126.67, 125.64, 114.87, 88.12, 34.36, 28.68, 15.81.

IR spectrum (neat)

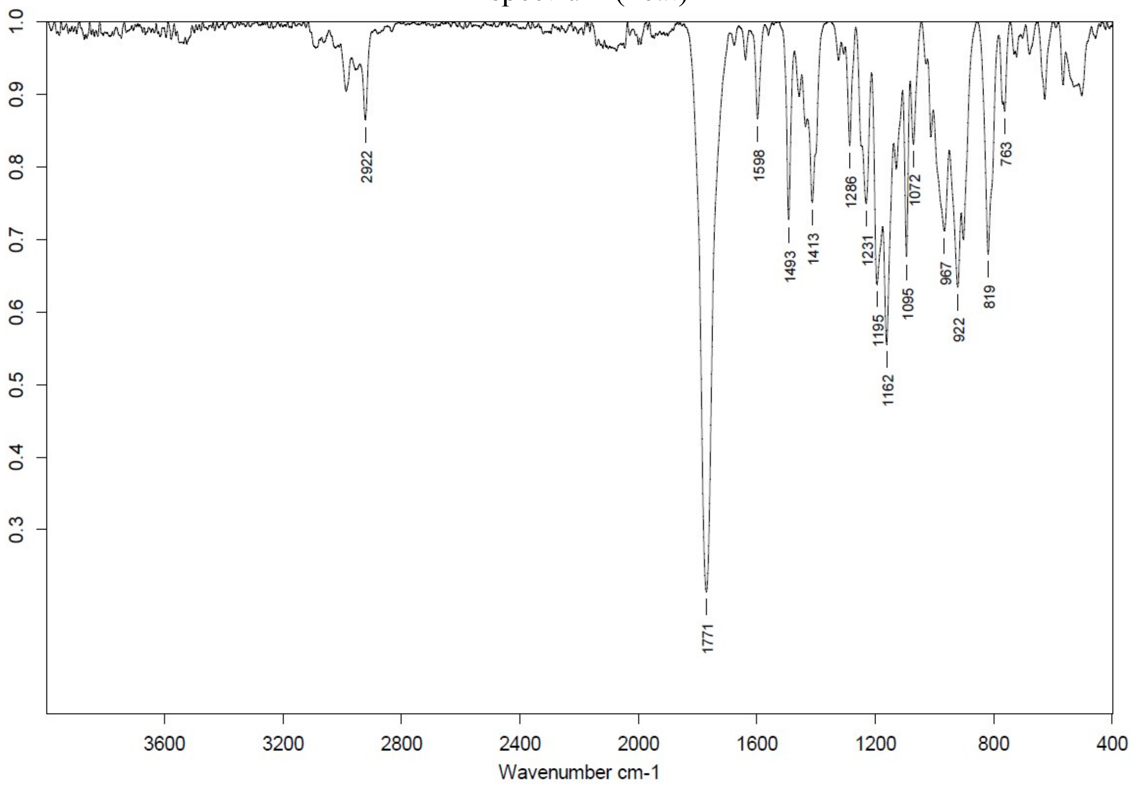

HRMS (ESI$^+$, MeOH): *m/z* calcd. 235.0787 $(M + H)^+$, found: 235.0790.

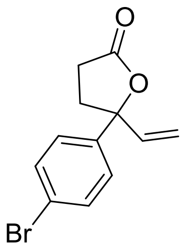

General procedure **A**: Yellow oil; Column conditions: Hexane : EA = 20 : 1, R_f = 0.15

^{1}H NMR spectrum ($CDCl_3$)

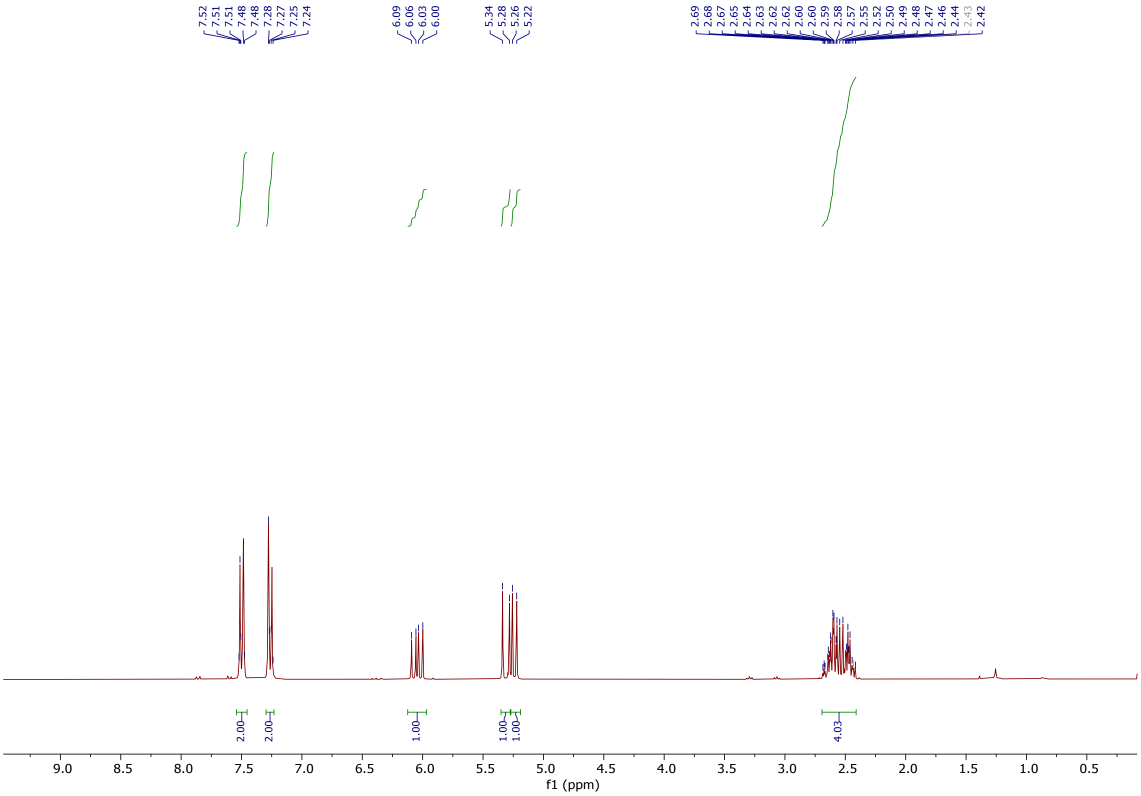

^{1}H NMR (300 MHz, $CDCl_3$) δ 7.54 – 7.45 (m, 2H), 7.30 – 7.23 (m, 2H), 6.04 (dd, J = 17.1, 10.7 Hz, 1H), 5.31 (d, J = 17.1 Hz, 1H), 5.24 (d, J = 10.7 Hz, 1H), 2.69 – 2.41 (m, 4H).

^{13}C NMR spectrum ($CDCl_3$)

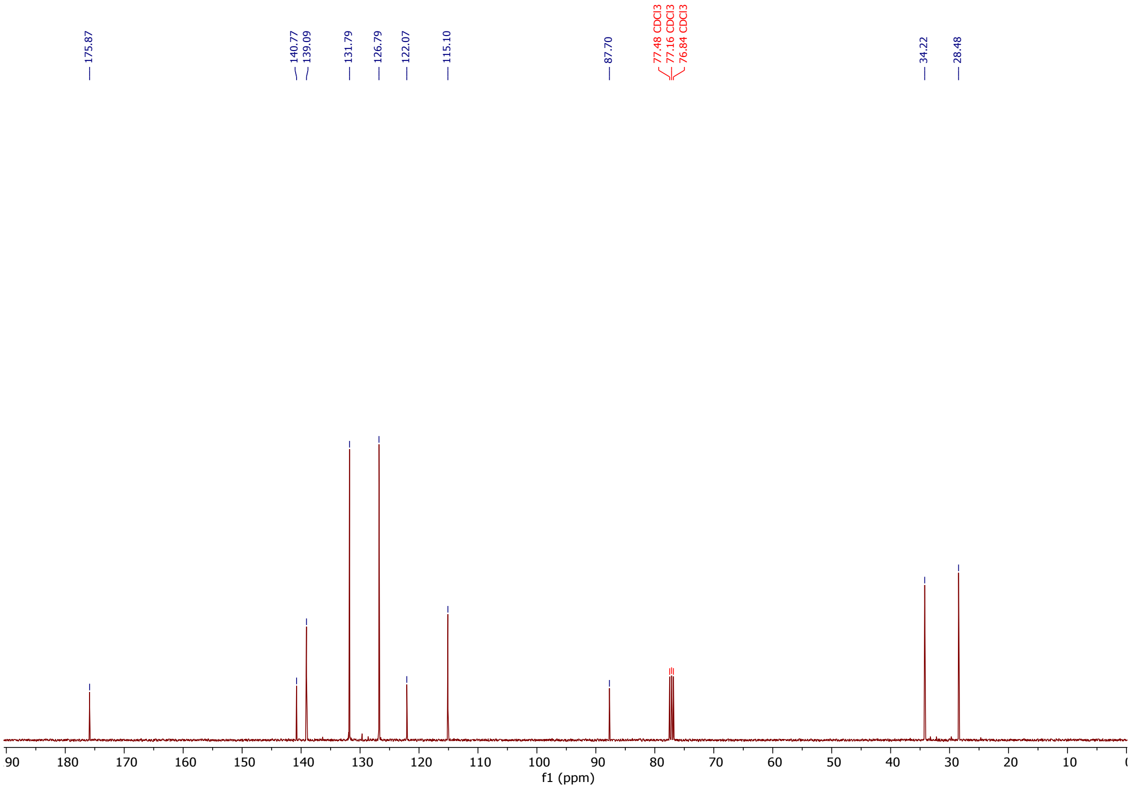

^{13}C NMR (101 MHz, $CDCl_3$) δ 175.87, 140.77, 139.09, 131.79, 126.79, 122.07, 115.10, 87.70, 34.22, 28.48.

IR spectrum (neat)

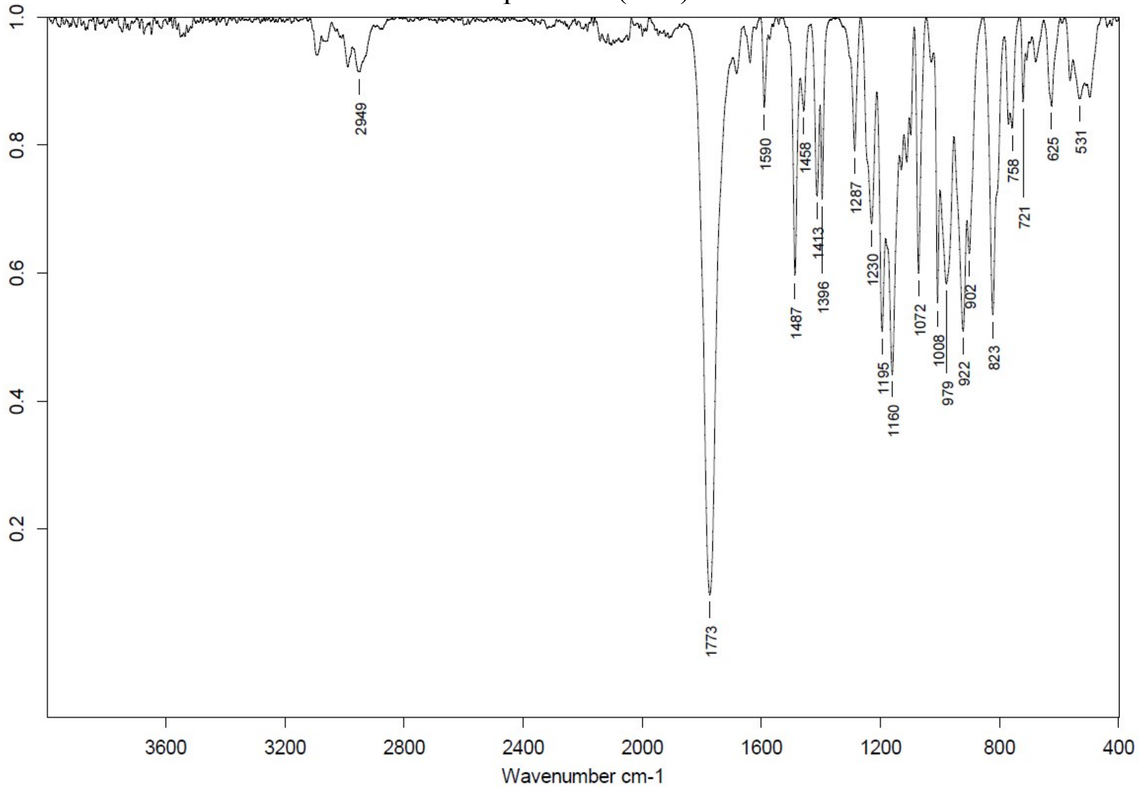

HRMS (ESI$^+$, MeOH): *m/z* calcd. 288.9835 $(M + Na)^+$, found: 288.9835.

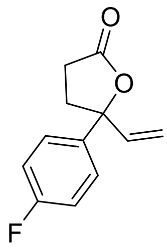

General procedure **A**: Yellow oil; Column conditions: Hexane : EA = 20 : 1, R_f = 0.18

^{1}H NMR spectrum ($CDCl_3$)

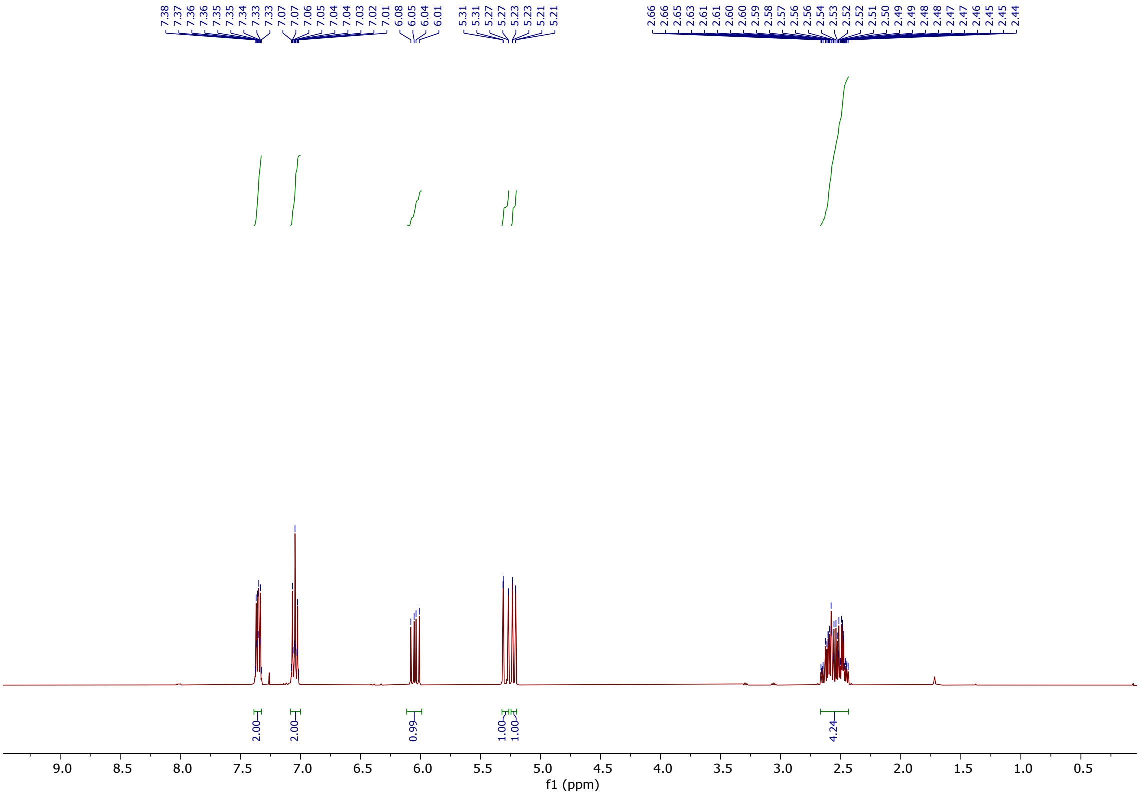

^{1}H NMR (400 MHz, $CDCl_3$) δ 7.39 – 7.32 (m, 2H), 7.08 – 7.00 (m, 2H), 6.04 (dd, J = 17.1, 10.7 Hz, 1H), 5.29 (dd, J = 17.2, 0.6 Hz, 1H), 5.22 (dd, J = 10.7, 0.6 Hz, 1H), 2.67 – 2.43 (m, 4H).

^{13}C NMR spectrum ($CDCl_3$)

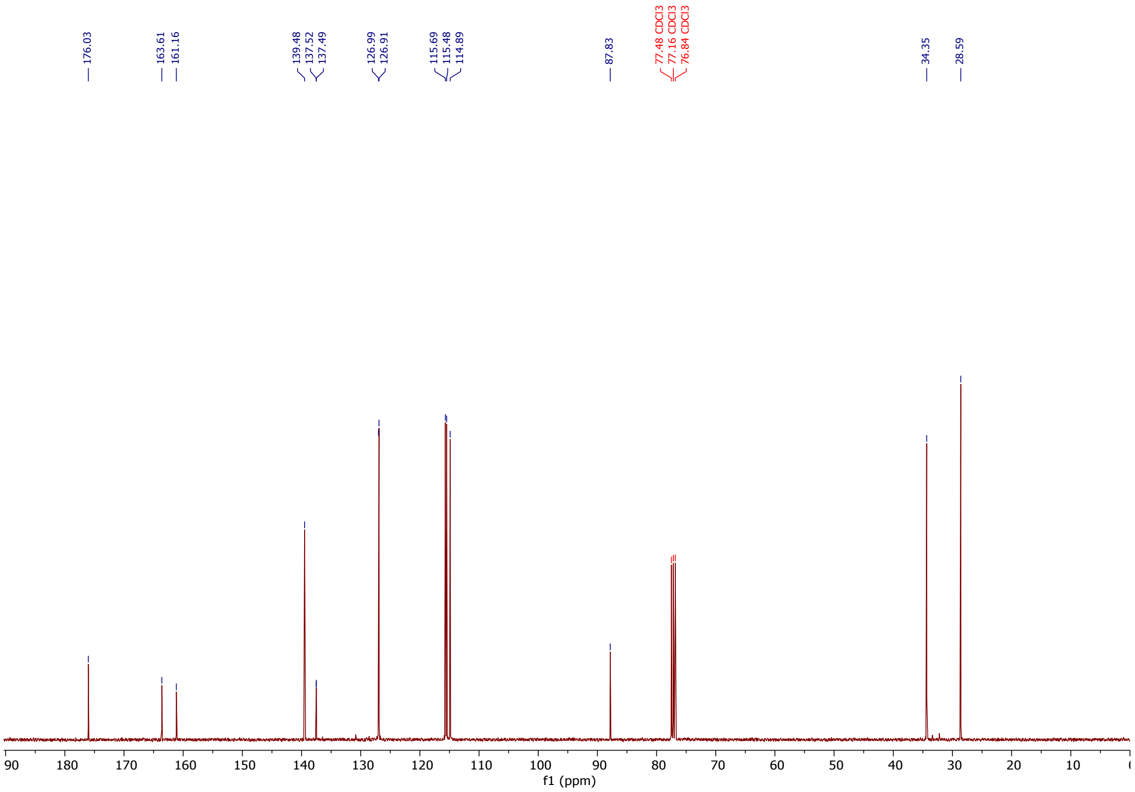

^{13}C NMR (101 MHz, $CDCl_3$) δ 176.03, 162.38 (d, J = 247.0 Hz), 139.48, 137.51 (d, J = 3.3 Hz), 126.95 (d, J = 8.4 Hz), 115.58 (d, J = 21.6 Hz), 114.89, 87.83, 34.35, 28.59.

^{19}F NMR spectrum ($CDCl_3$)

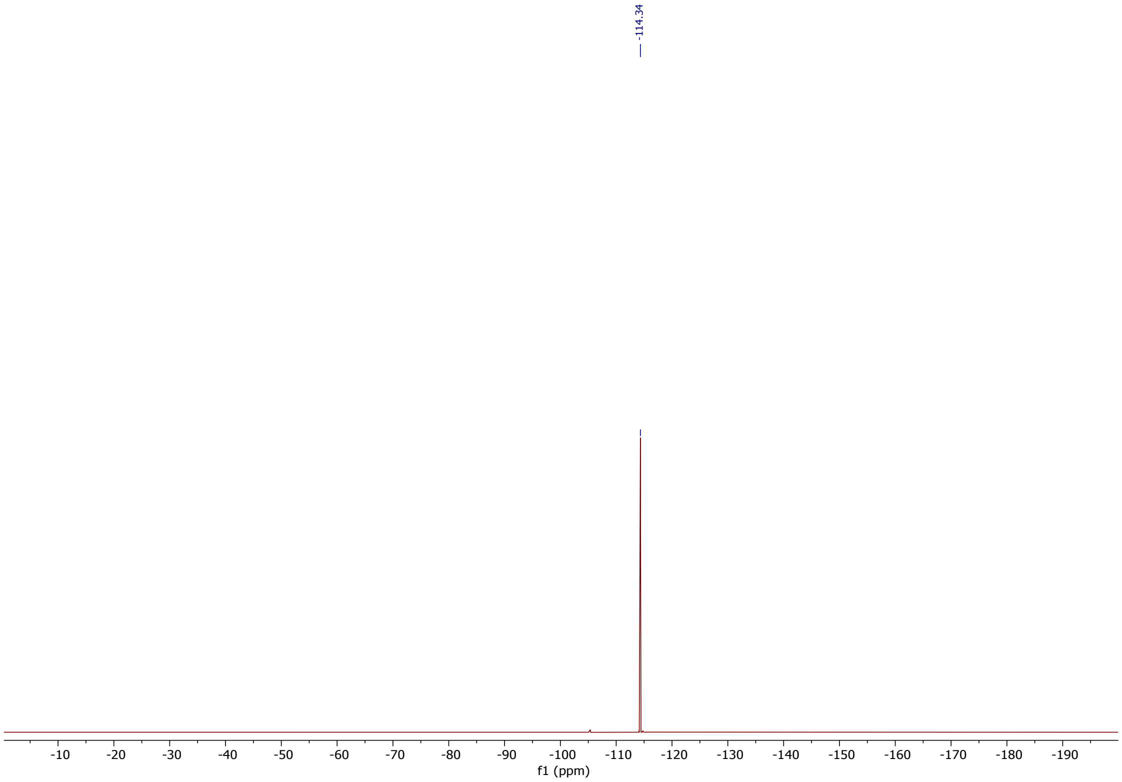

^{19}F NMR (376 MHz, $CDCl_3$) δ -114.34.

IR spectrum (neat)

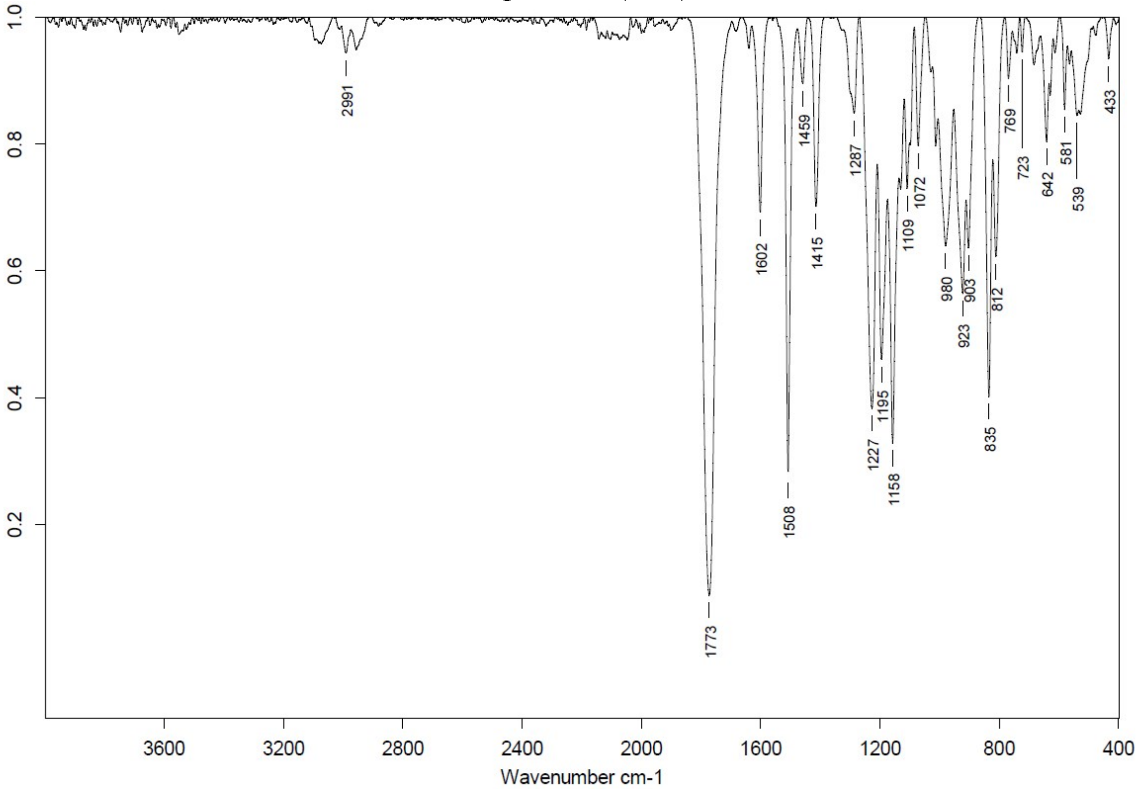

HRMS (ESI$^+$, MeOH): *m/z* calcd. 207.0816 $(M + H)^+$, found: 207.0818.

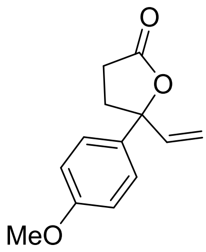

General procedure **A**: Yellow oil; Column conditions: Hexane : DCM = 1 : 1, R_f = 0.20

^{1}H NMR spectrum ($CDCl_3$)

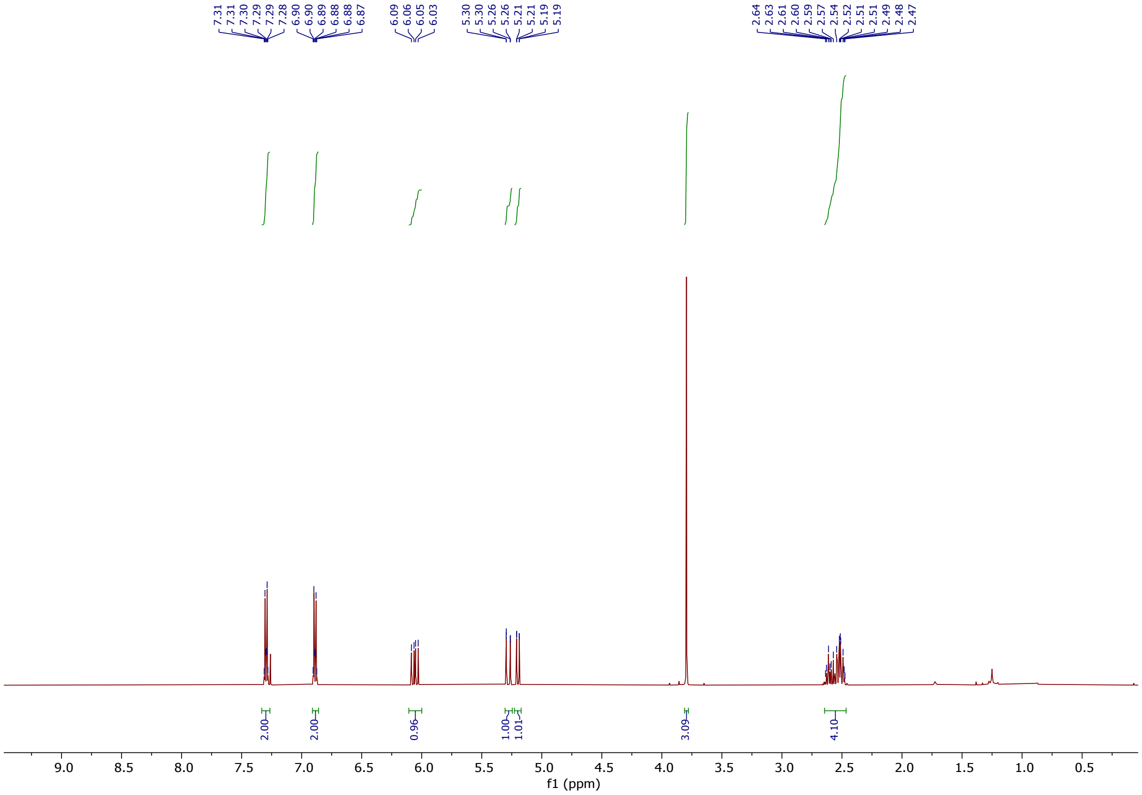

^{1}H NMR (500 MHz, $CDCl_3$) δ 7.33 – 7.26 (m, 2H), 6.91 – 6.86 (m, 2H), 6.06 (dd, J = 17.2, 10.7 Hz, 1H), 5.28 (dd, J = 17.2, 0.8 Hz, 1H), 5.20 (dd, J = 10.7, 0.7 Hz, 1H), 3.79 (s, 3H), 2.65 – 2.47 (m, 4H).

^{13}C NMR spectrum ($CDCl_3$)

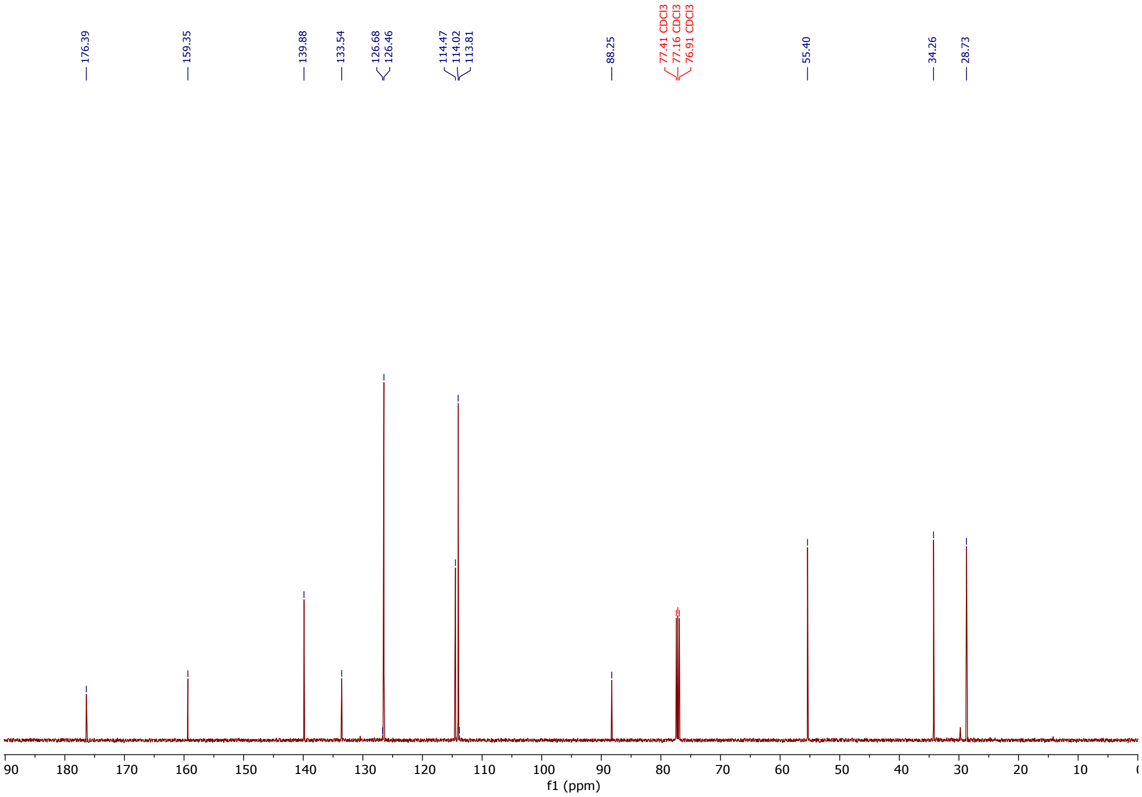

^{13}C NMR (126 MHz, $CDCl_3$) δ 176.39, 159.35, 139.88, 133.54, 126.68, 126.46, 114.47, 114.02, 113.81, 88.25, 55.40, 34.26, 28.73.[5]

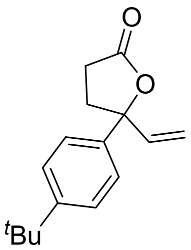

General procedure **A**: Slight yellow oil; Column conditions: Hexane : EA = 20 : 1, R_f = 0.20

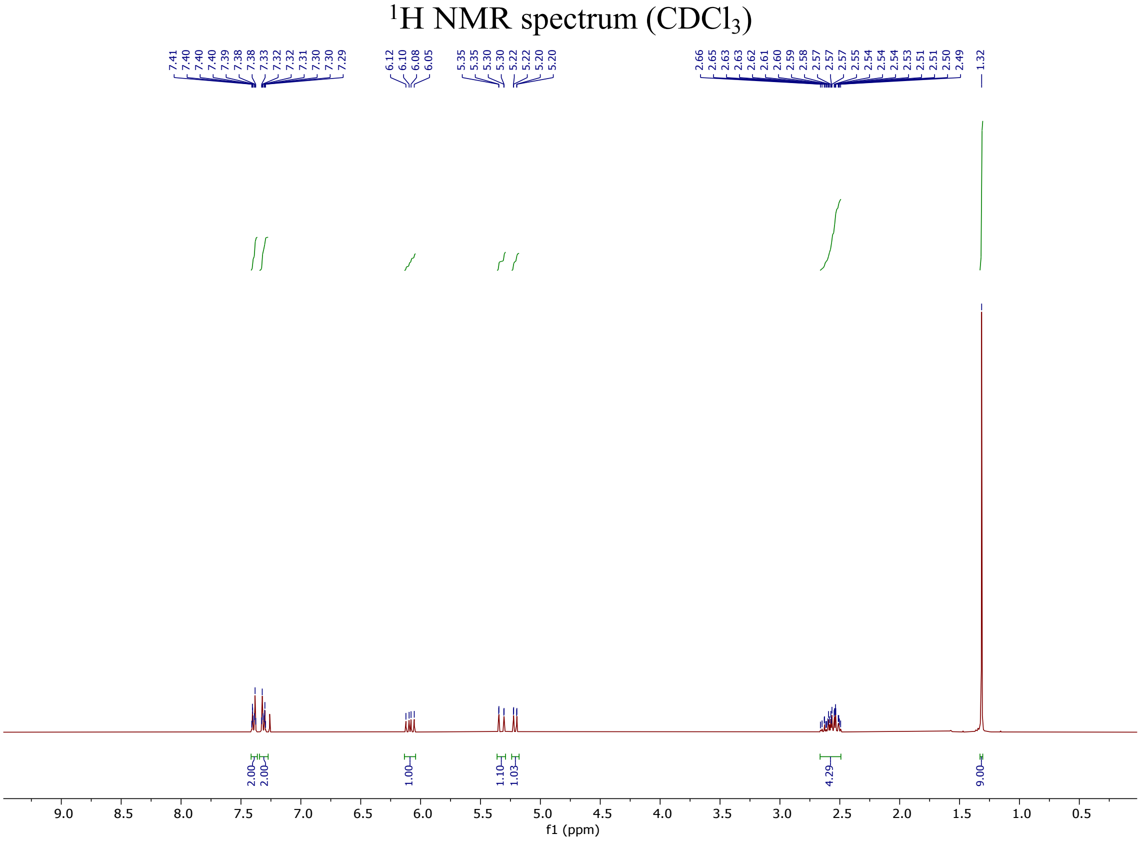

^{1}H NMR (400 MHz, $CDCl_3$) δ 7.42 – 7.36 (m, 2H), 7.35 – 7.27 (m, 2H), 6.09 (dd, J = 17.2, 10.7 Hz, 1H), 5.33 (dd, J = 17.2, 0.7 Hz, 1H), 5.21 (dd, J = 10.7, 0.7 Hz, 1H), 2.66 – 2.49 (m, 4H), 1.32 (s, 9H).

^{13}C NMR spectrum ($CDCl_3$)

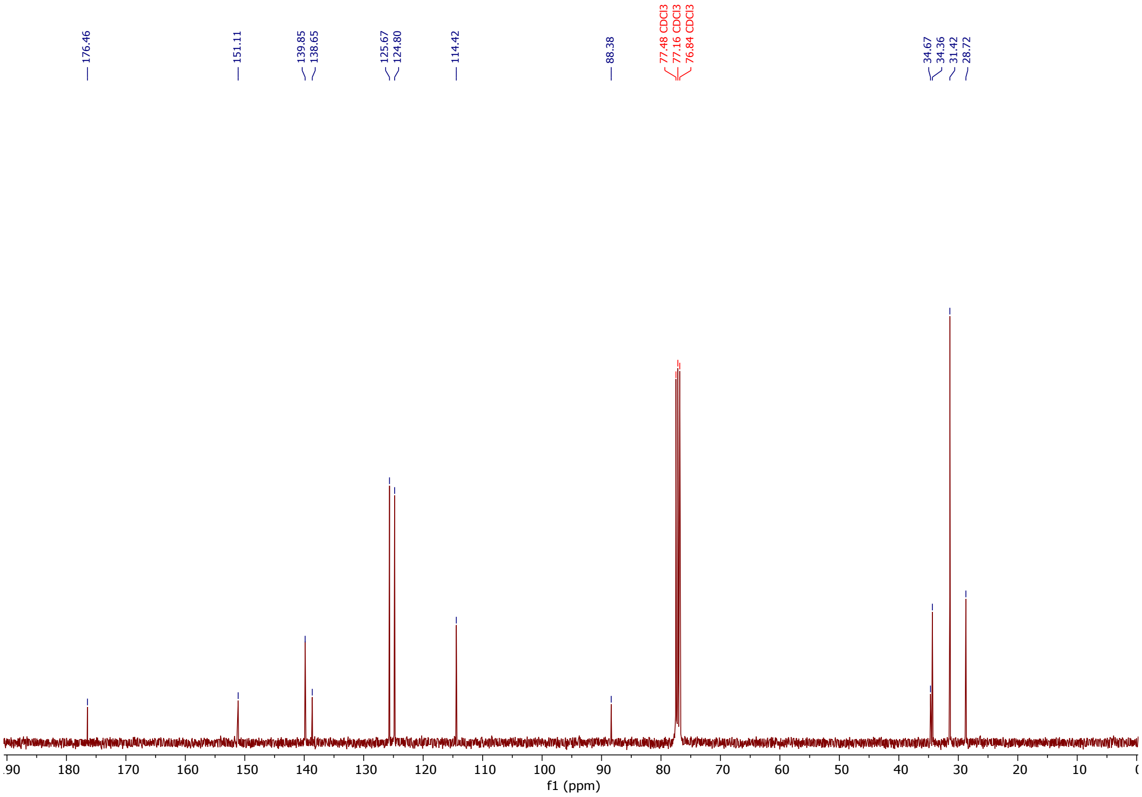

^{13}C NMR (101 MHz, $CDCl_3$) δ 176.46, 151.11, 139.85, 138.65, 125.67, 124.80, 114.42, 88.38, 34.67, 34.36, 31.42, 28.72.

IR spectrum (neat)

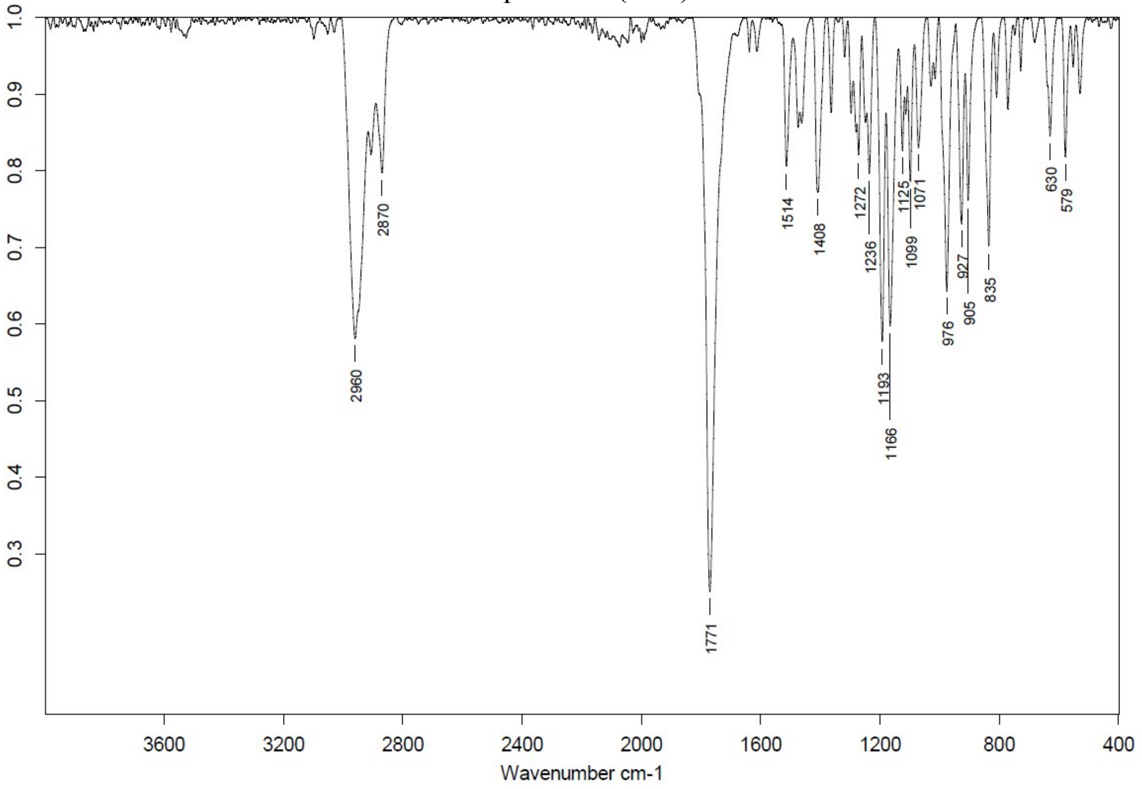

HRMS (ESI$^+$, MeOH): *m/z* calcd. 267.1356 (M + Na)$^+$, found: 267.1359.

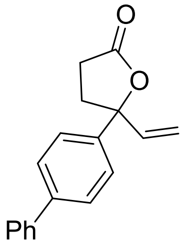

General procedure **A**: White solid; Column conditions: Hexane : EA = 20 : 1, R_f = 0.10

^{1}H NMR spectrum ($CDCl_3$)

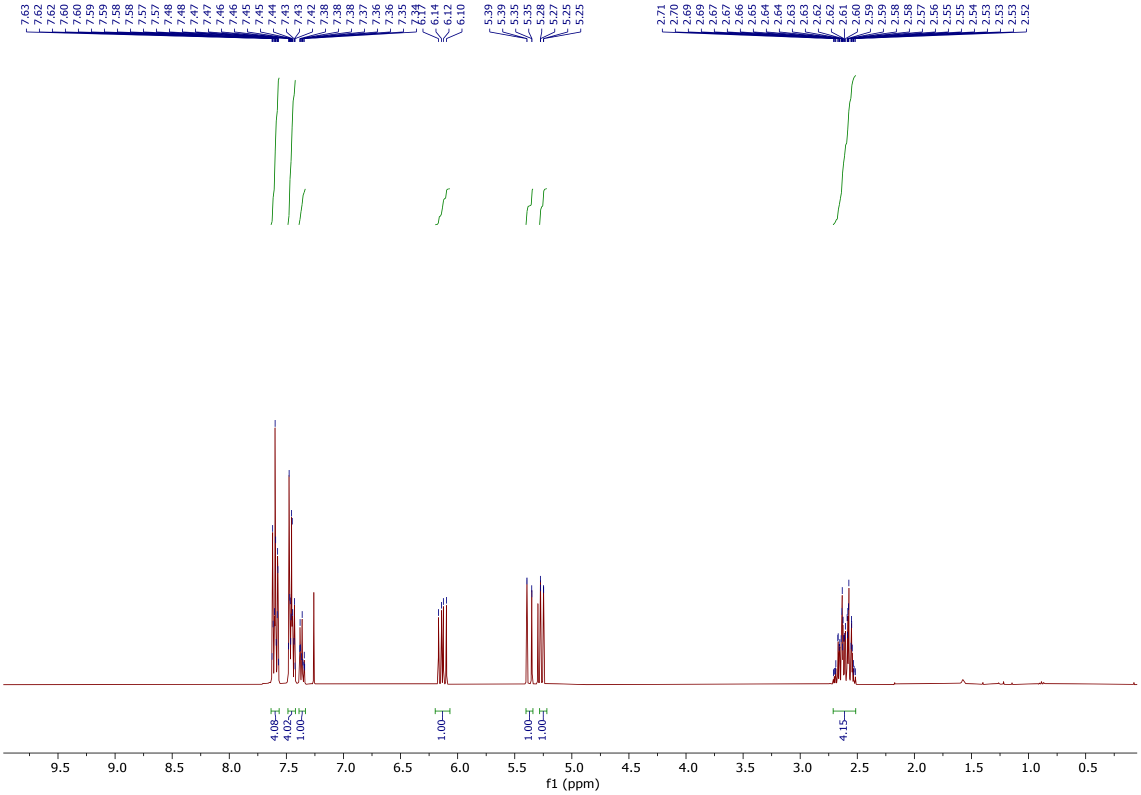

^{1}H NMR (400 MHz, $CDCl_3$) δ 7.63 – 7.56 (m, 4H), 7.49 – 7.42 (m, 4H), 7.39 – 7.33 (m, 1H), 6.13 (dd, J = 17.2, 10.7 Hz, 1H), 5.37 (dd, J = 17.2, 0.7 Hz, 1H), 5.26 (dd, J = 10.7, 0.7 Hz, 1H), 2.71 – 2.51 (m, 4H).

^{13}C NMR spectrum ($CDCl_3$)

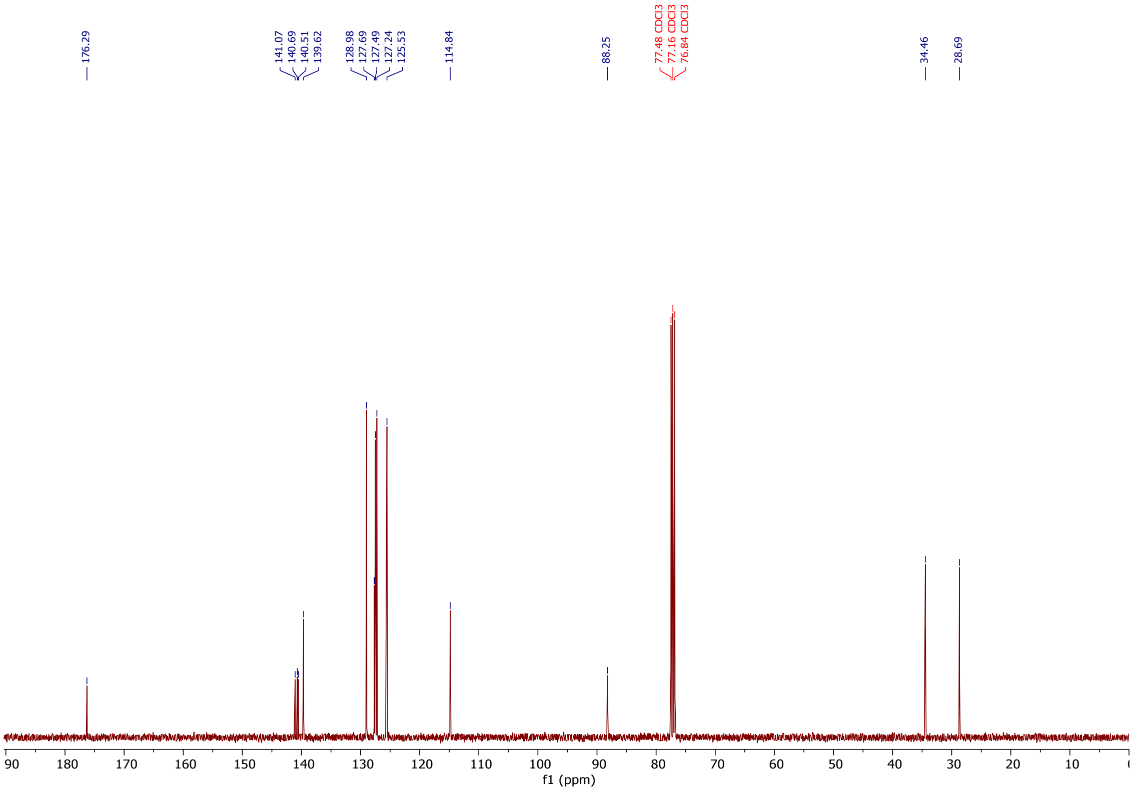

^{13}C NMR (101 MHz, $CDCl_3$) δ 176.29, 141.07, 140.69, 140.51, 139.62, 128.98, 127.69, 127.49, 127.24, 125.53, 114.84, 88.25, 34.46, 28.69.

IR spectrum (neat)

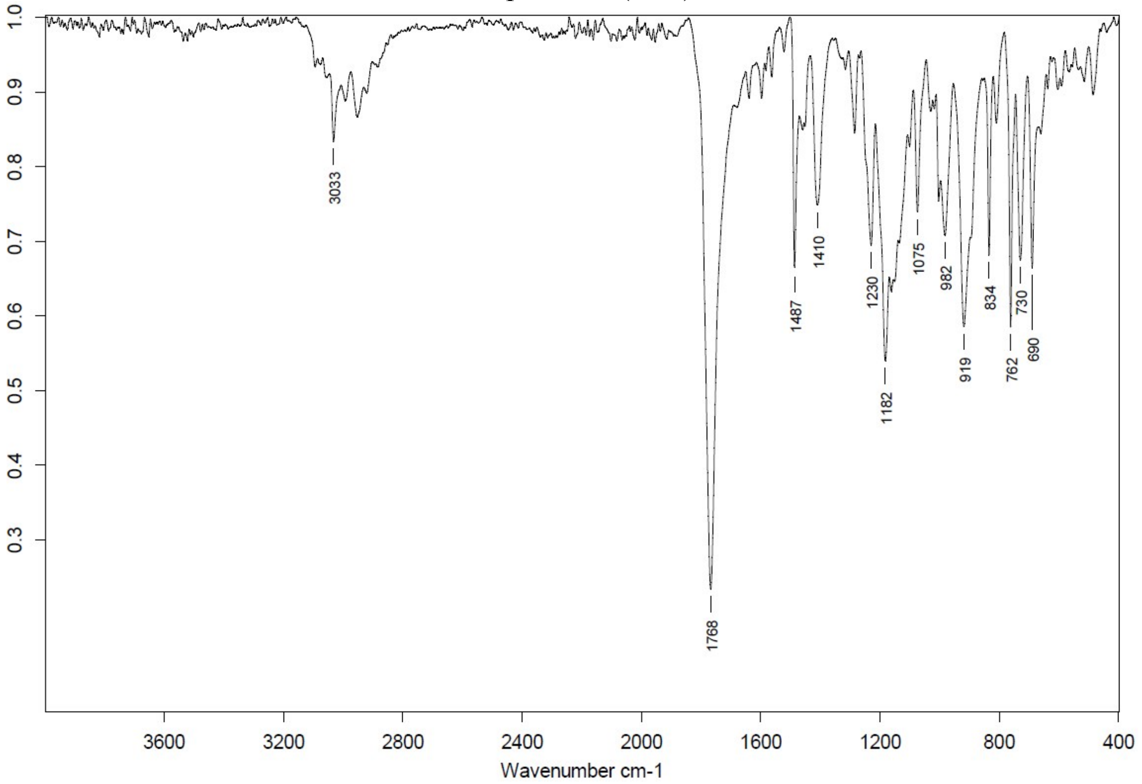

HRMS (ESI$^+$, MeOH): *m/z* calcd. 265.1223 $(M + H)^+$, found: 265.1226.

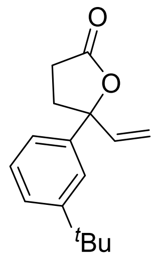

General procedure **A**: Colorless oil; Column conditions: Hexane : EA = 20 : 1, R_f = 0.20

^{1}H NMR spectrum ($CDCl_3$)

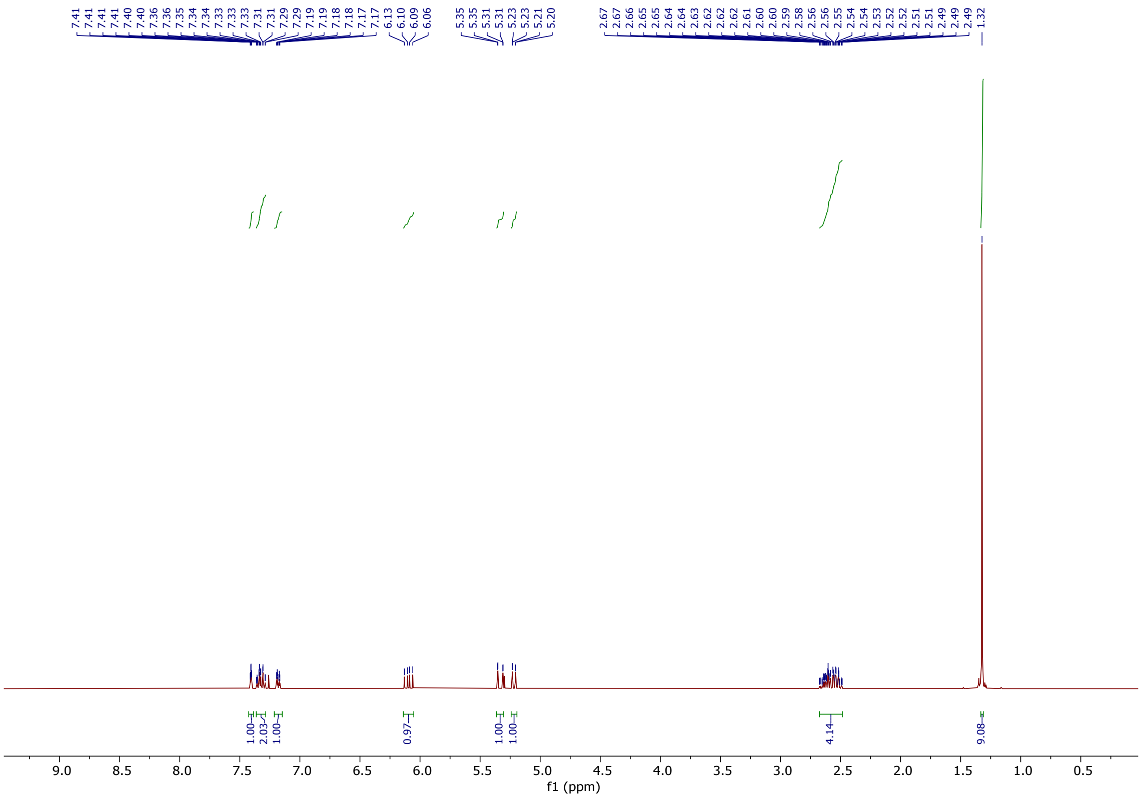

^{1}H NMR (400 MHz, $CDCl_3$) δ 7.42 – 7.38 (m, 1H), 7.36 – 7.28 (m, 2H), 7.21 – 7.13 (m, 1H), 6.09 (dd, J = 17.1, 10.7 Hz, 1H), 5.33 (dd, J = 17.2, 0.7 Hz, 1H), 5.22 (dd, J = 10.7, 0.7 Hz, 1H), 2.68 – 2.48 (m, 4H), 1.32 (s, 9H).

^{13}C NMR spectrum ($CDCl_3$)

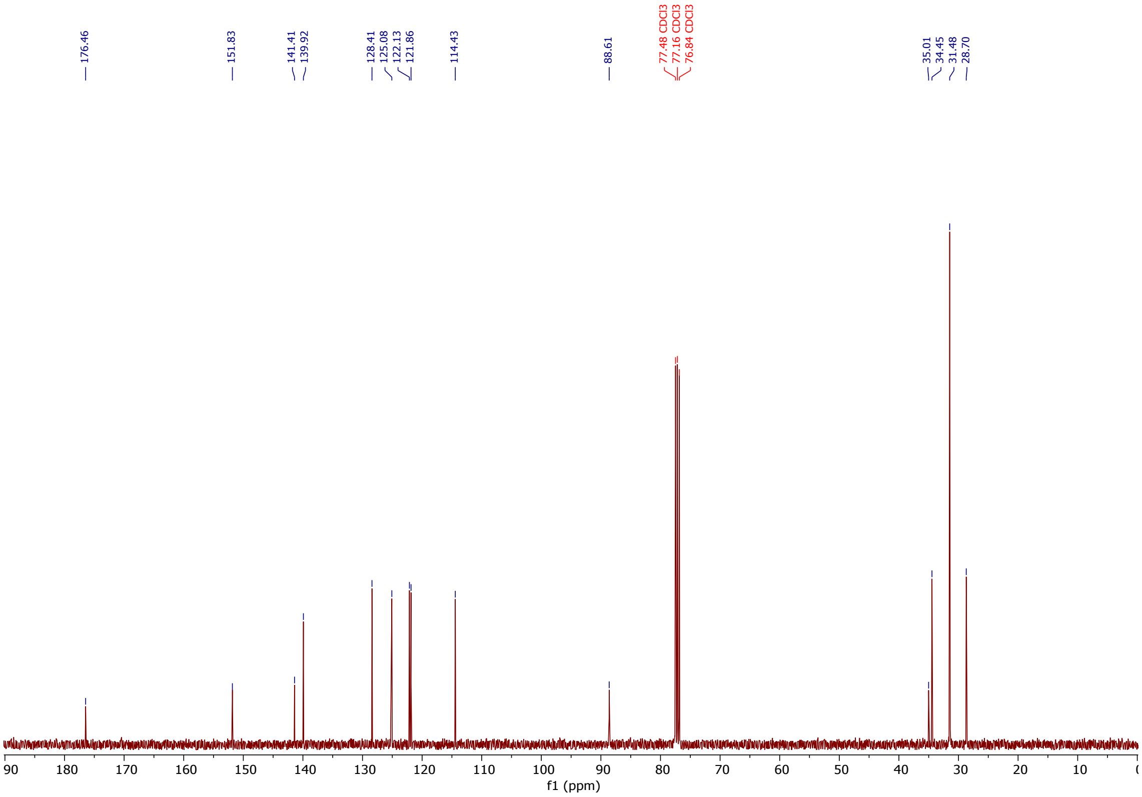

^{13}C NMR (101 MHz, $CDCl_3$) δ 176.46, 151.83, 141.41, 139.92, 128.41, 125.08, 122.13, 121.86, 114.43, 88.61, 35.01, 34.45, 31.48, 28.70.

IR spectrum (neat)

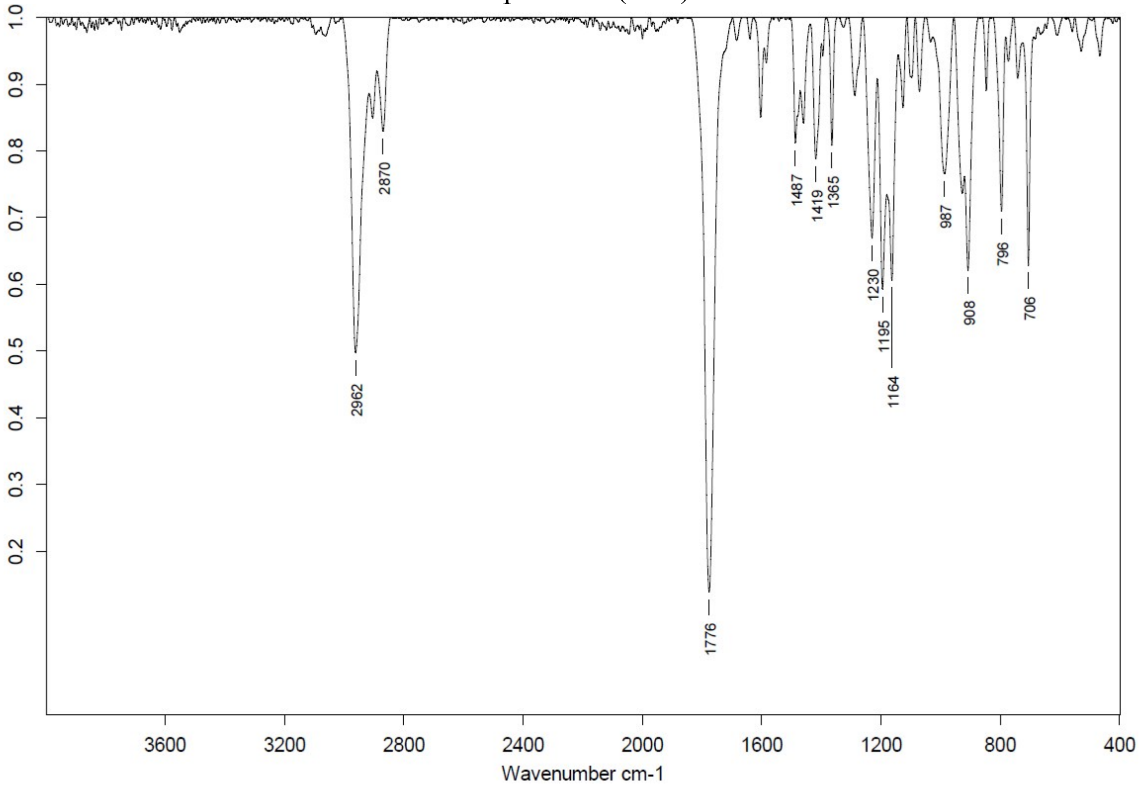

HRMS (ESI$^+$, MeOH): *m/z* calcd. 267.1356 (M + Na)$^+$, found: 267.1357.

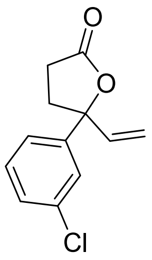

General procedure **A**: Yellow oil; Column conditions: Hexane : DCM = 1 : 1, $R_f = 0.18$

^{1}H NMR spectrum ($CDCl_3$)

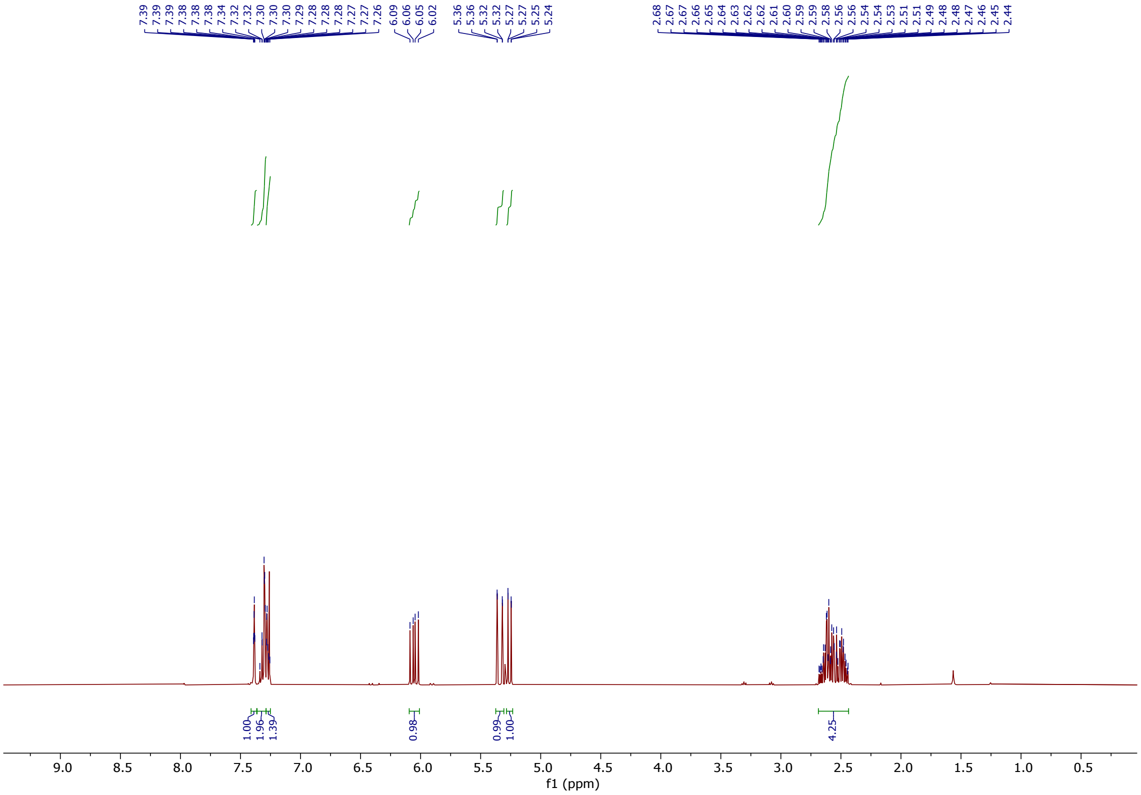

^{1}H NMR (400 MHz, $CDCl_3$) δ 7.40 – 7.36 (m, 1H), 7.36 – 7.29 (m, 2H), 7.29 – 7.25 (m, 1H), 6.05 (dd, J = 17.1, 10.7 Hz, 1H), 5.34 (dd, J = 17.1, 0.5 Hz, 1H), 5.26 (dd, J = 10.7, 0.5 Hz, 1H), 2.69 – 2.44 (m, 4H).

^{13}C NMR spectrum ($CDCl_3$)

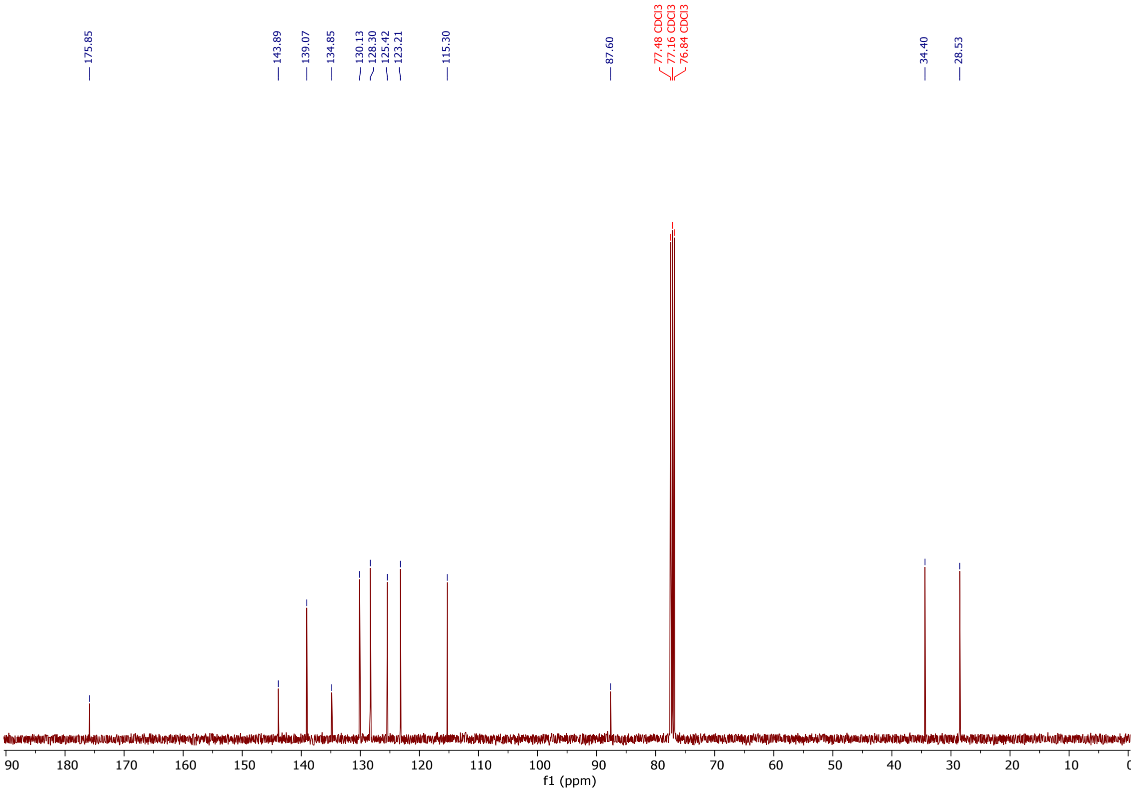

^{13}C NMR (101 MHz, $CDCl_3$) δ 175.85, 143.89, 139.07, 134.85, 130.13, 128.30, 125.42, 123.21, 115.30, 87.60, 34.40, 28.53.

IR spectrum (neat)

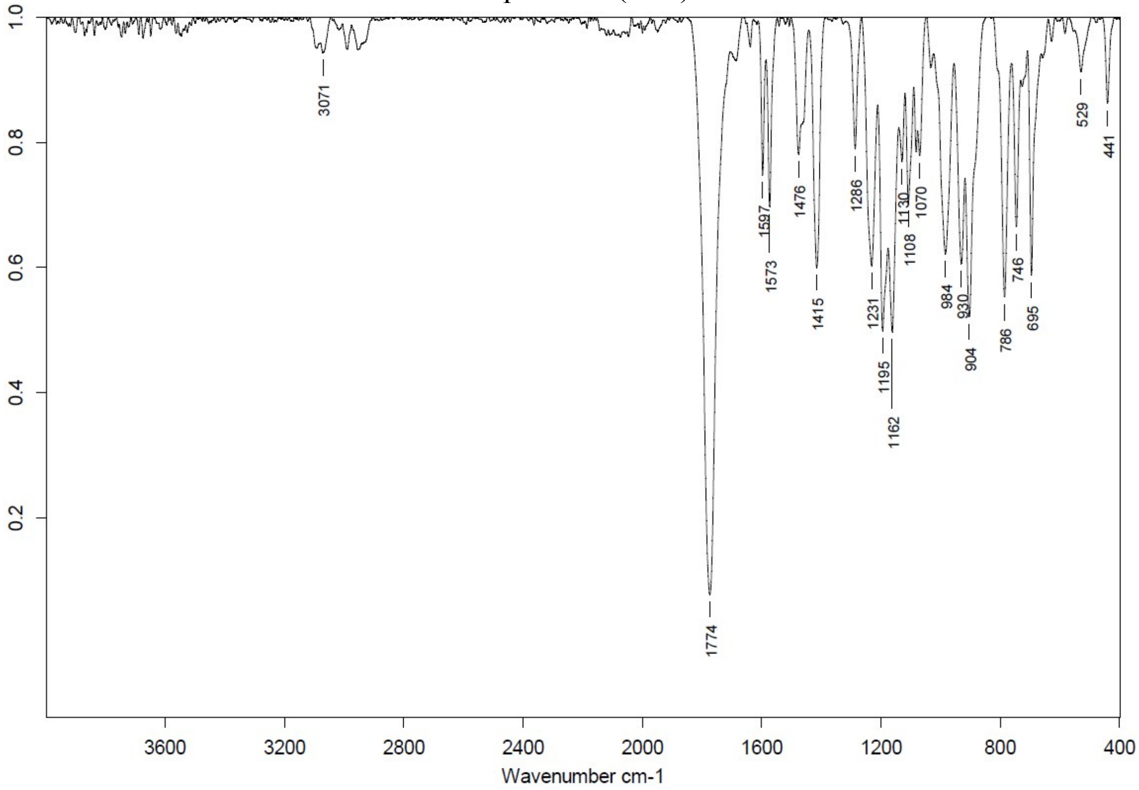

HRMS (ESI$^+$, MeOH): *m/z* calcd. 245.0340 $(M + Na)^+$, found: 245.0344.

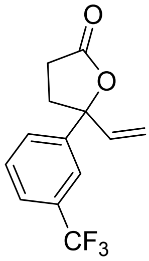

General procedure **A**: Yellow oil; Column conditions: Hexane : EA = 20 : 1, R_f = 0.15

^{1}H NMR spectrum ($CDCl_3$)

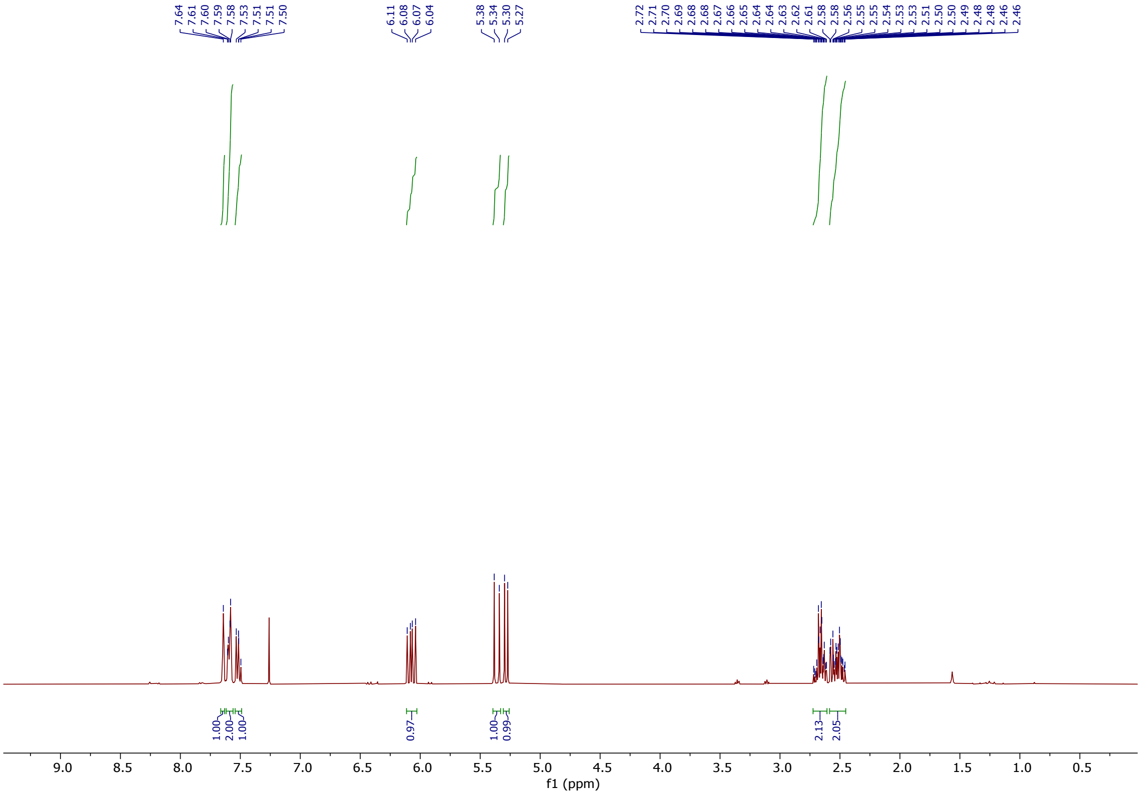

^{1}H NMR (400 MHz, $CDCl_3$) δ 7.64 (s, 1H), 7.61 – 7.55 (m, 2H), 7.53 – 7.41 (m, 1H), 6.07 (dd, J = 17.2, 10.7 Hz, 1H), 5.36 (d, J = 17.1 Hz, 1H), 5.28 (d, J = 10.7 Hz, 1H), 2.72 – 2.61 (m, 2H), 2.59 – 2.45 (m, 2H).

^{13}C NMR spectrum ($CDCl_3$)

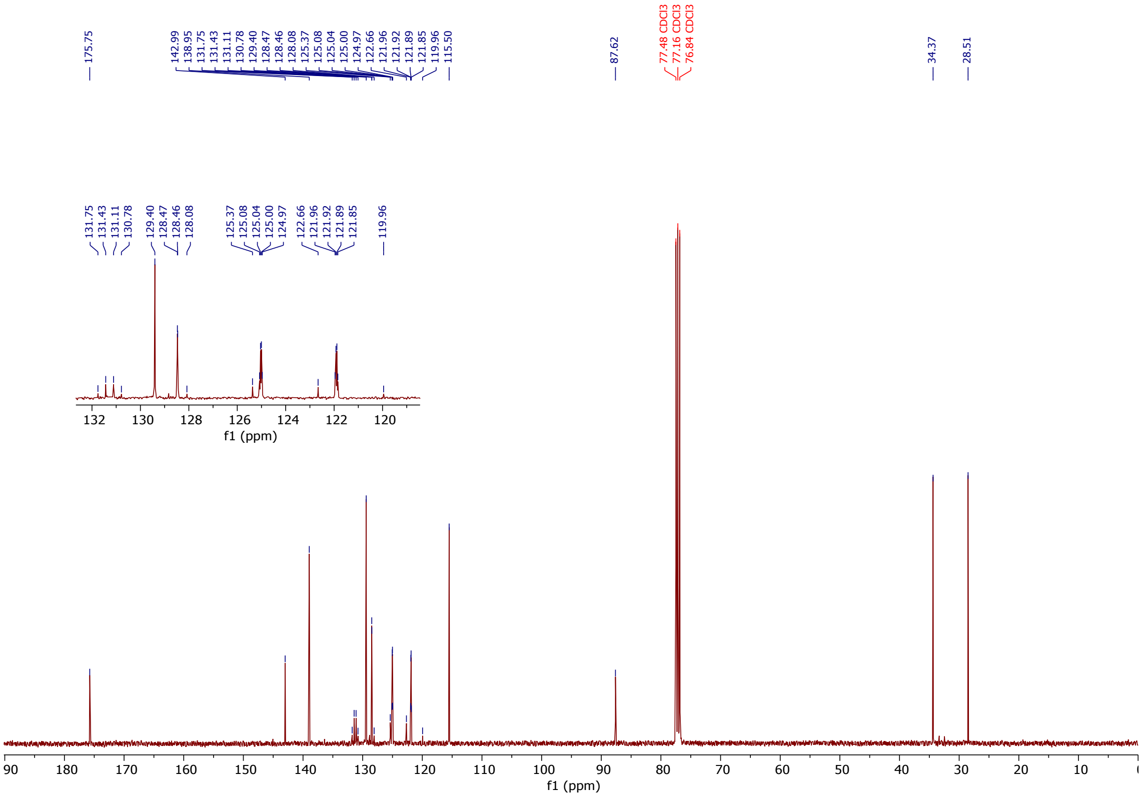

^{13}C NMR (101 MHz, $CDCl_3$) δ 175.75, 142.99, 138.95, 131.27 (q, J = 32.5 Hz), 129.40, 128.47, 128.46, 125.02 (q, J = 3.8 Hz), 124.02 (q, J = 272.4 Hz), 121.90 (q, J = 3.9 Hz), 115.50, 87.62, 34.37, 28.51.

^{19}F NMR spectrum ($CDCl_3$)

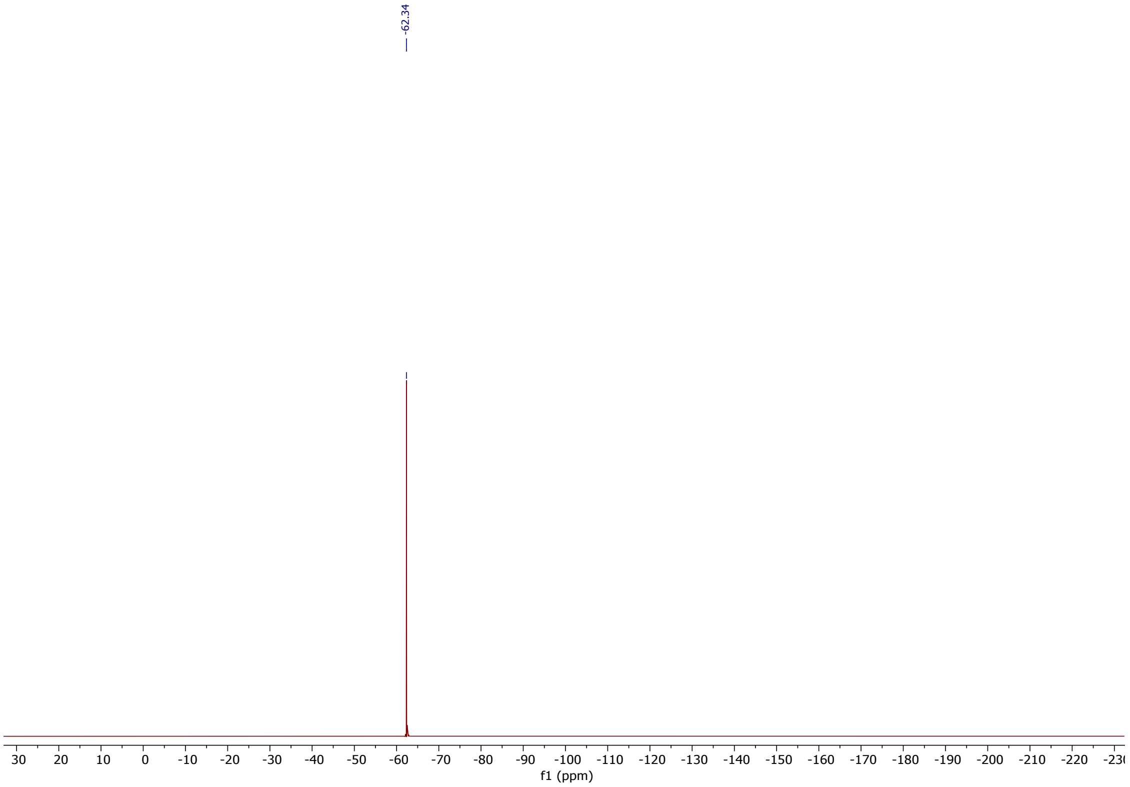

^{19}F NMR (376 MHz, $CDCl_3$) δ -62.34.

IR spectrum (neat)

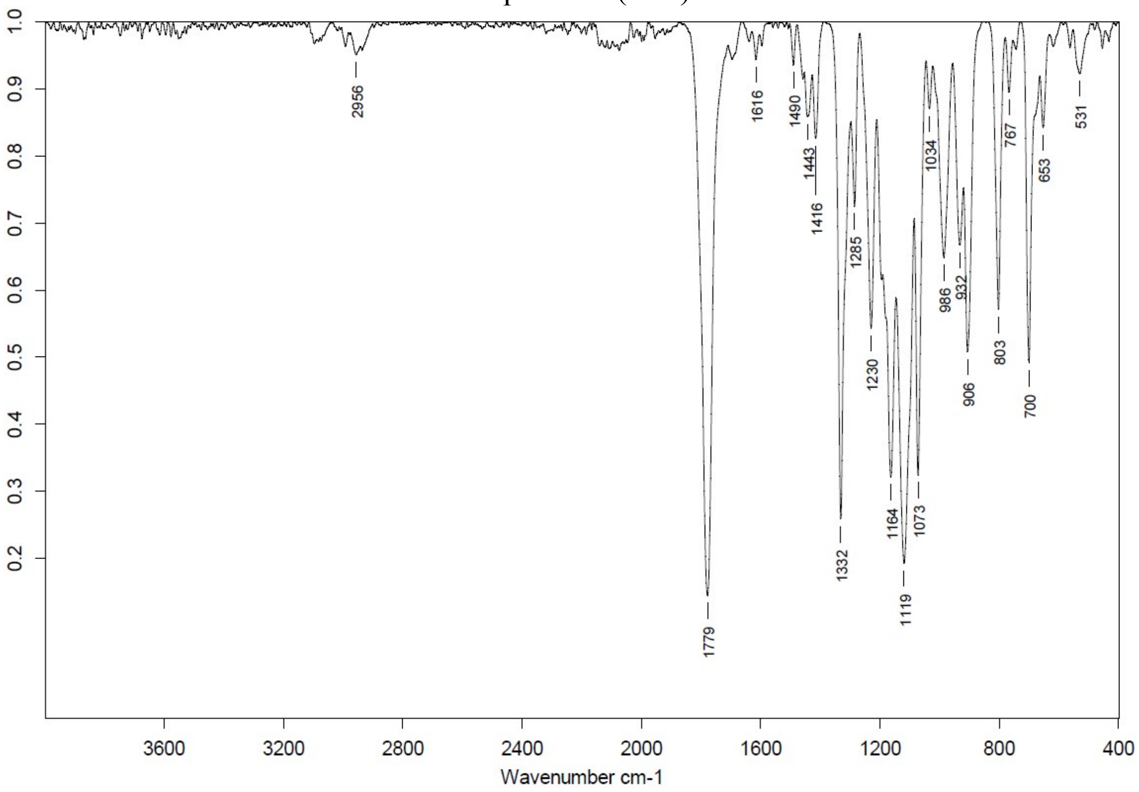

HRMS (ESI$^+$, MeOH): *m/z* calcd. 279.0603 (M + Na)$^+$, found: 279.0609.

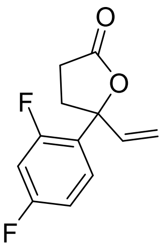

General procedure **A**: Yellow oil; Column conditions: Hexane : DCM = 1 : 1, R_f = 0.15

^{1}H NMR spectrum ($CDCl_3$)

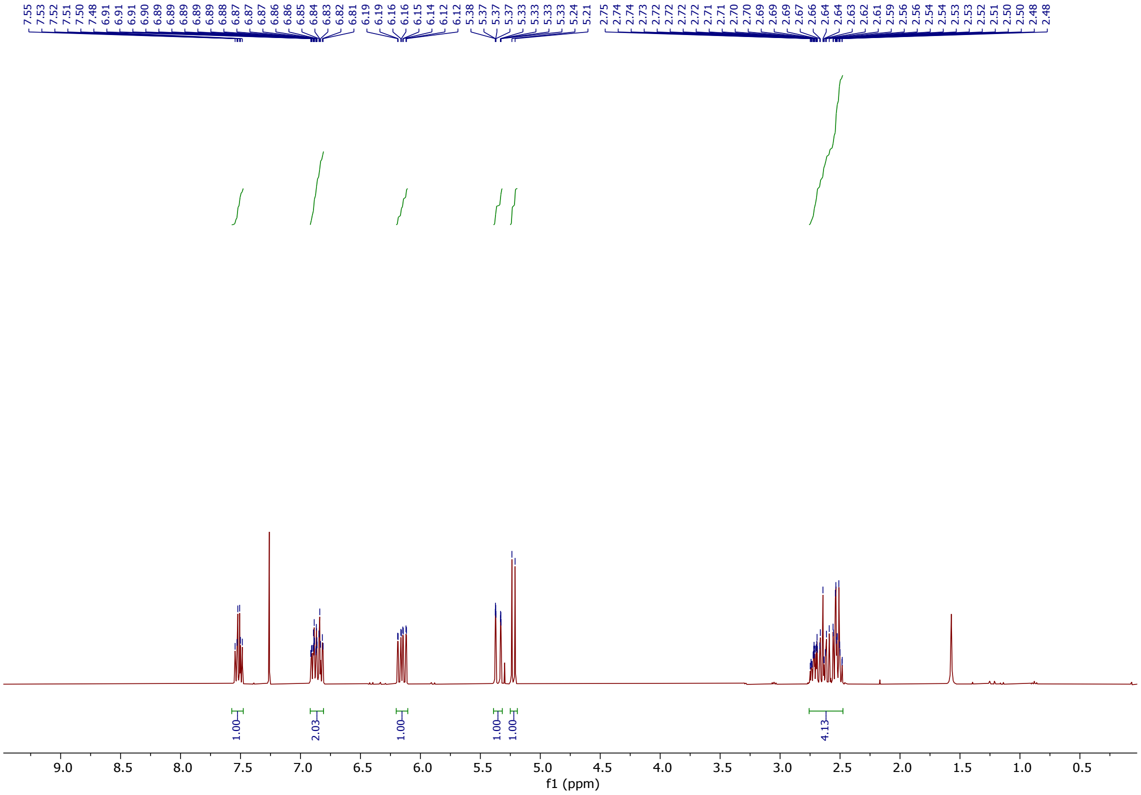

^{1}H NMR (400 MHz, $CDCl_3$) δ 7.51 (td, *J* = 8.9, 6.4 Hz, 1H), 6.92 – 6.81 (m, 2H), 6.15 (ddd, *J* = 17.1, 10.7, 1.6 Hz, 1H), 5.35 (ddd, *J* = 17.1, 1.5, 0.6 Hz, 1H), 5.22 (d, *J* = 10.7 Hz, 1H), 2.76 – 2.48 (m, 4H).

^{13}C NMR spectrum ($CDCl_3$)

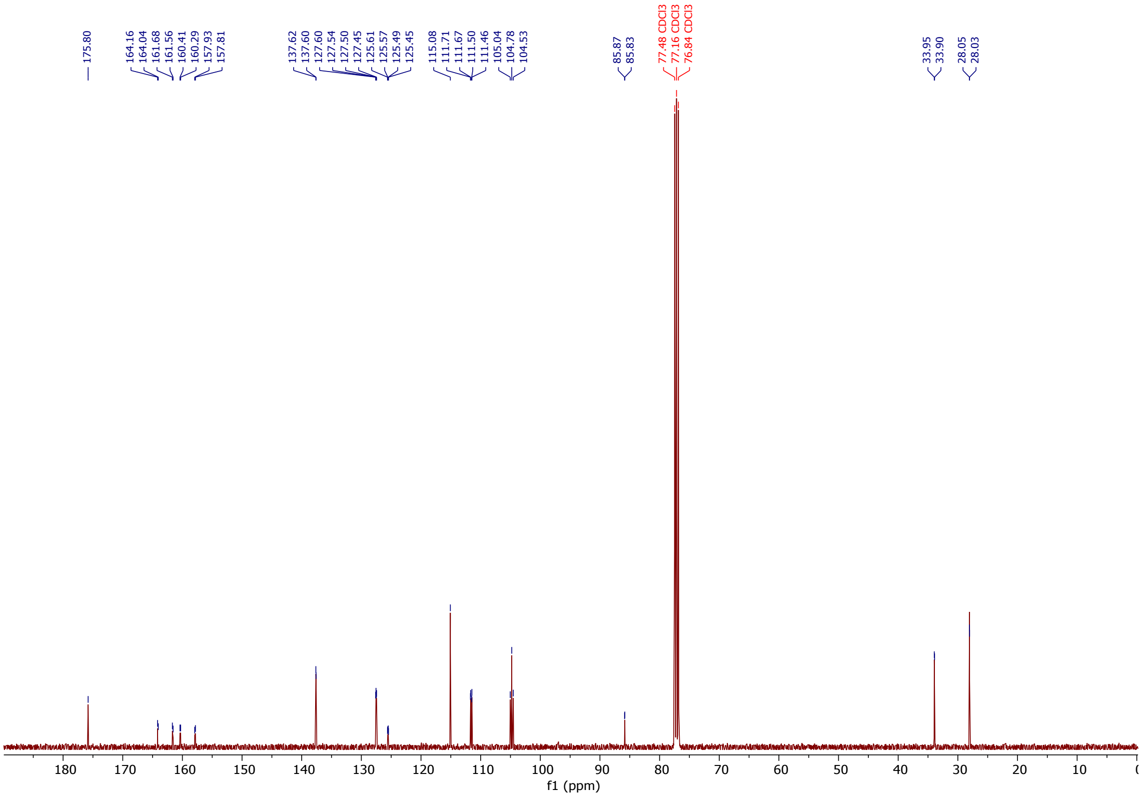

^{13}C NMR (101 MHz, $CDCl_3$) δ 175.80, 162.86 (dd, J = 249.8, 12.1 Hz), 159.11 (dd, J = 249.1, 11.7 Hz), 137.61 (d, J = 2.6 Hz), 127.52 (dd, J = 9.5, 5.5 Hz), 125.53 (dd, J = 12.5, 4.0 Hz), 115.08, 111.59 (dd, J = 20.9, 3.7 Hz), 104.78 (t, J = 25.7 Hz), 85.85 (d, J = 3.7 Hz), 33.93 (d, J = 5.1 Hz), 28.04 (d, J = 1.8 Hz).

^{19}F NMR spectrum ($CDCl_3$)

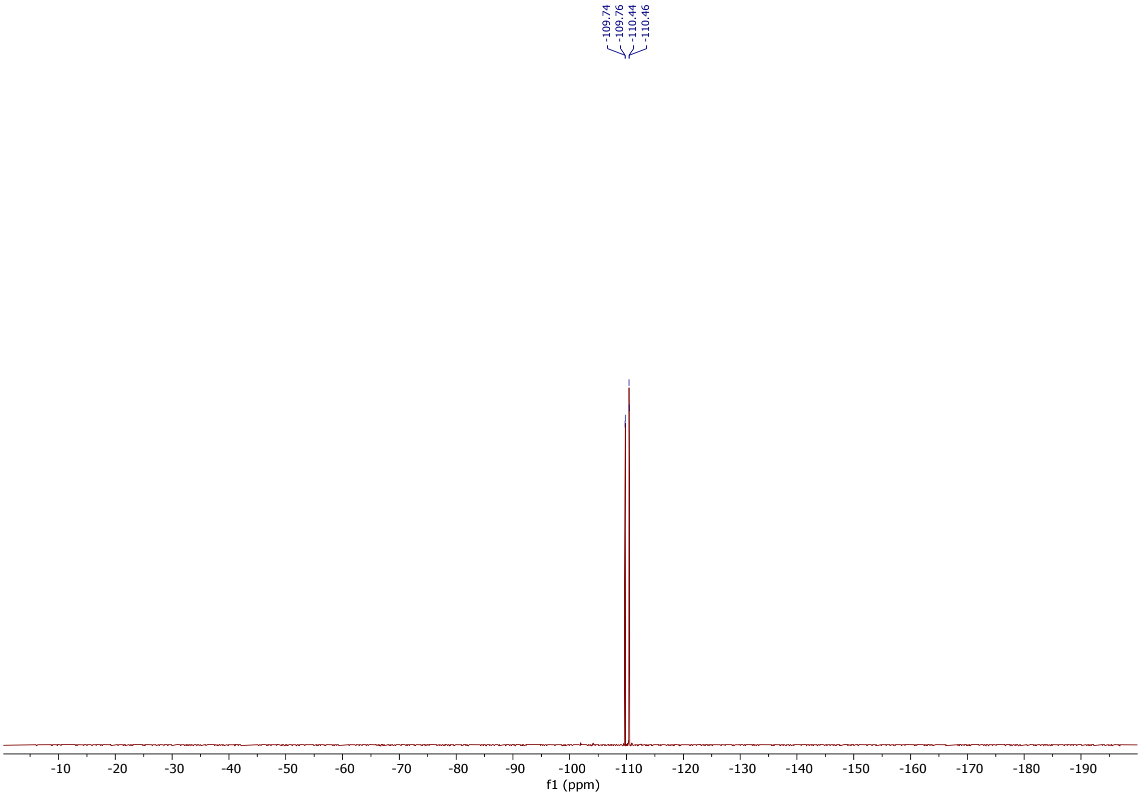

^{19}F NMR (376 MHz, $CDCl_3$) δ -109.75 (d, J = 8.0 Hz), -110.45 (d, J = 8.0 Hz).

IR spectrum (neat)

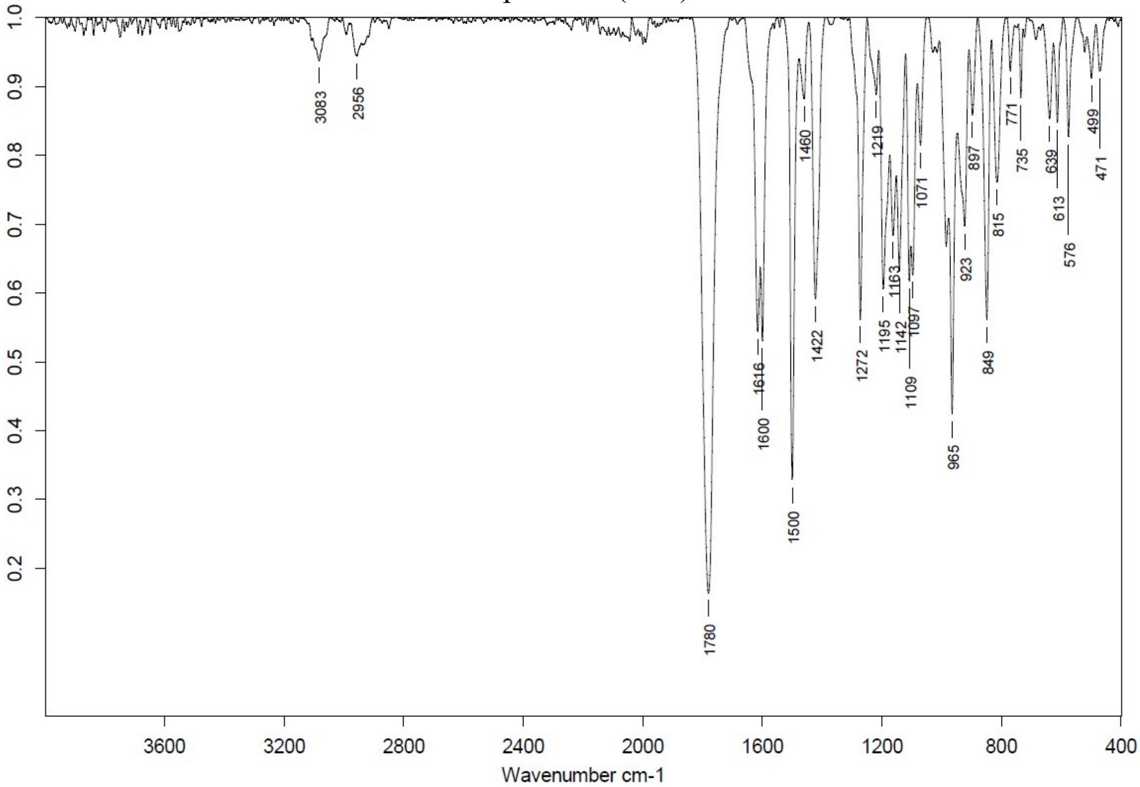

HRMS (ESI^+, MeOH): *m/z* calcd. 247.0541 $(M + Na)^+$, found: 247.0544.

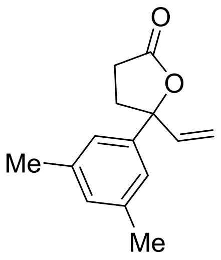

General procedure **A**: Colorless oil; Column conditions: Hexane : EA = 20 : 1, R_f = 0.20

^{1}H NMR spectrum ($CDCl_3$)

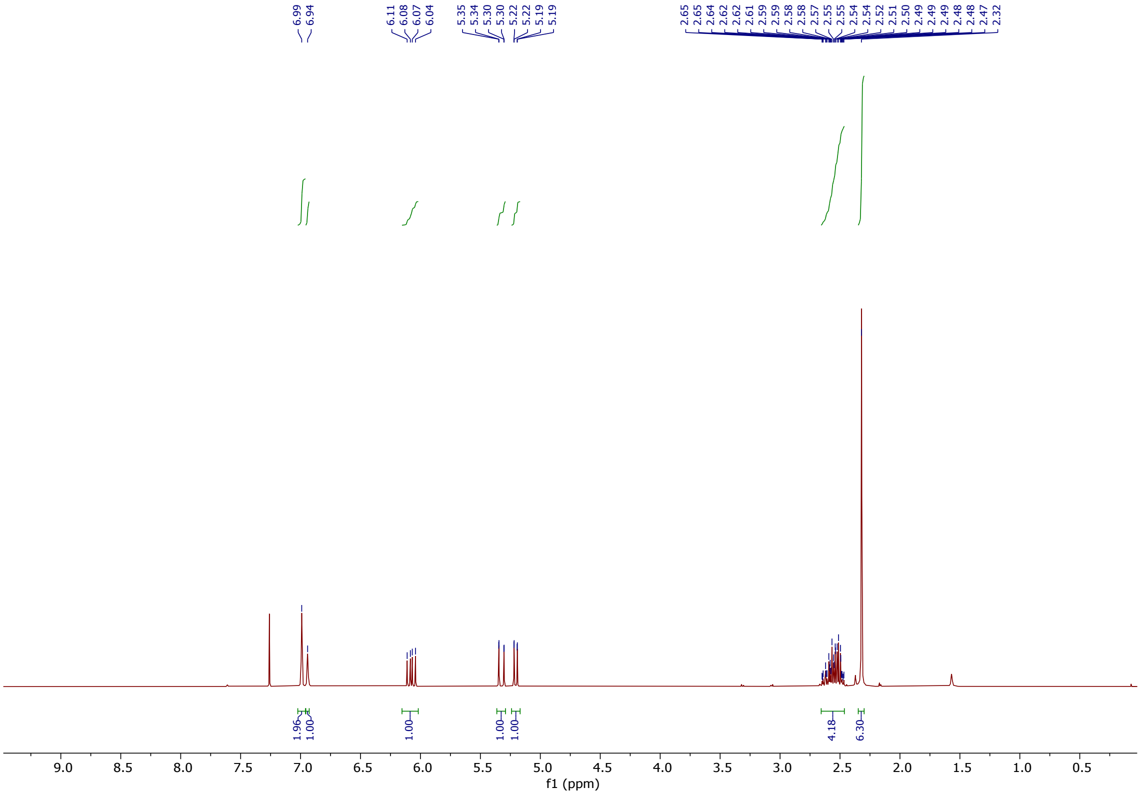

^{1}H NMR (400 MHz, $CDCl_3$) δ 6.99 (s, 2H), 6.94 (s, 1H), 6.08 (dd, J = 17.2, 10.7 Hz, 1H), 5.32 (dd, J = 17.2, 0.8 Hz, 1H), 5.21 (dd, J = 10.7, 0.7 Hz, 1H), 2.66 – 2.46 (m, 4H), 2.32 (s, 6H).

^{13}C NMR spectrum ($CDCl_3$)

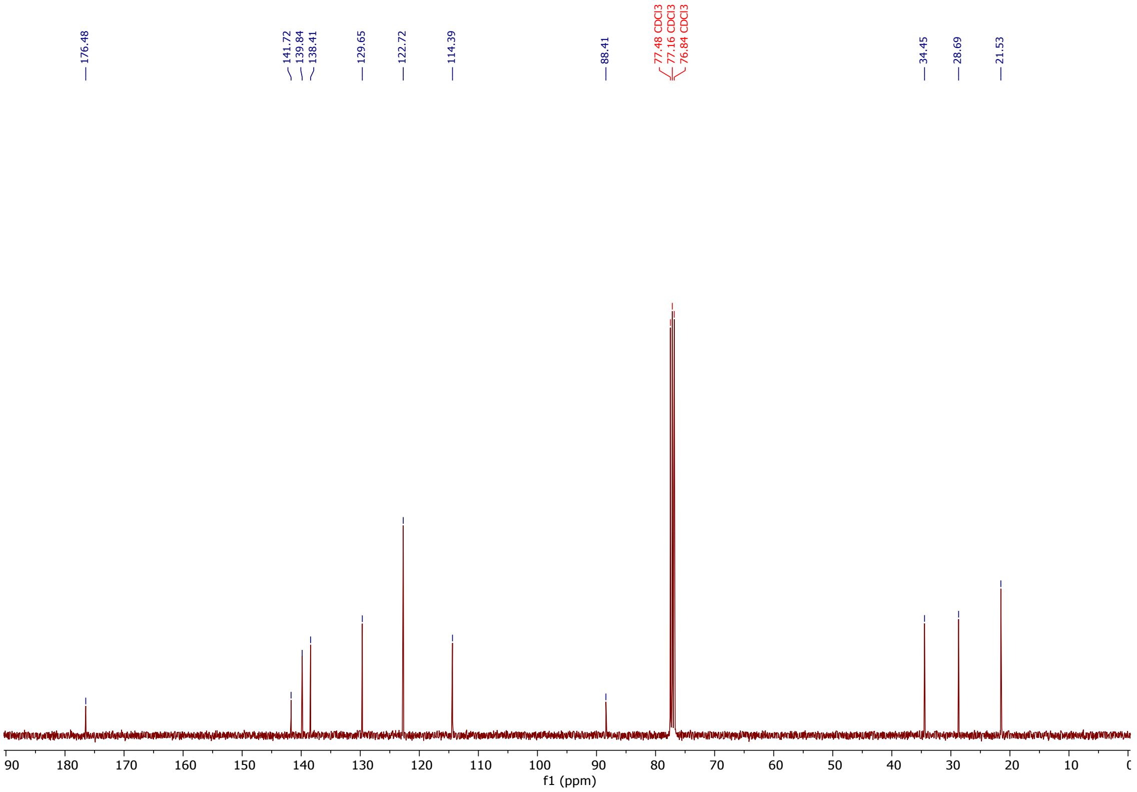

^{13}C NMR (101 MHz, $CDCl_3$) δ 176.48, 141.72, 139.84, 138.41, 129.65, 122.72, 114.39, 88.41, 34.45, 28.69, 21.53.

IR spectrum (neat)

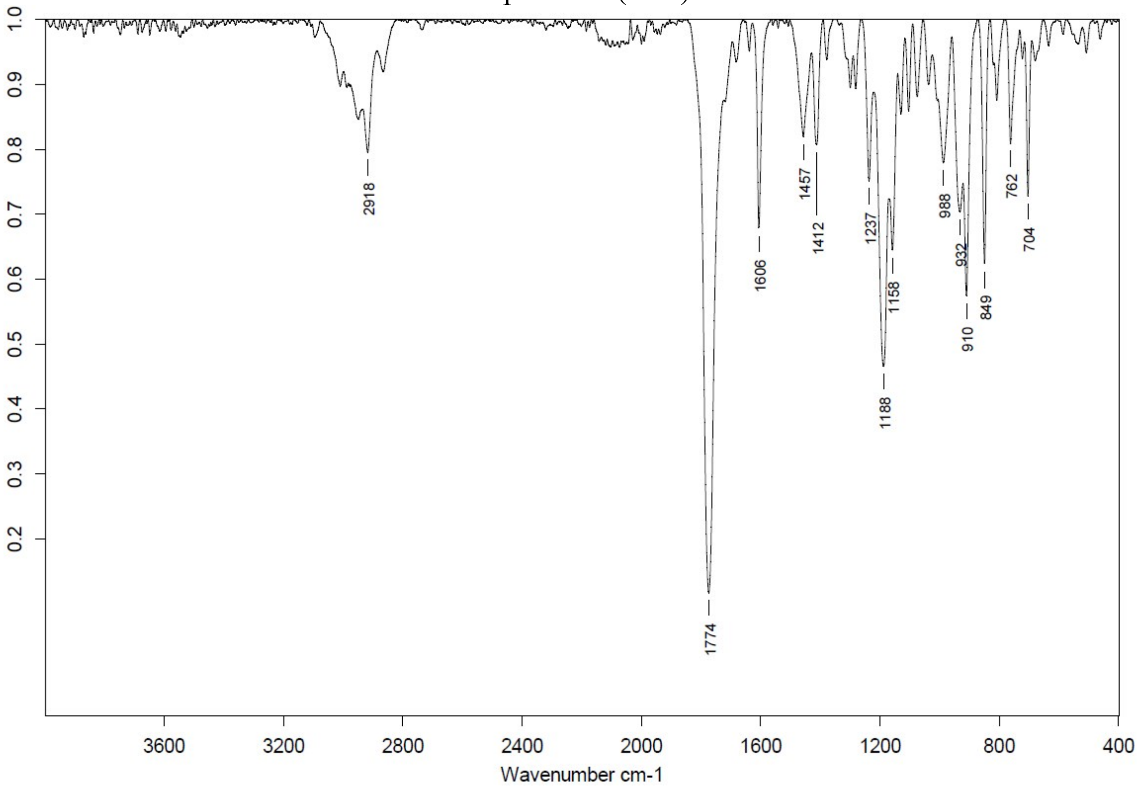

HRMS (ESI$^+$, MeOH): *m/z* calcd. 239.1043 $(M + Na)^+$, found: 239.1032.

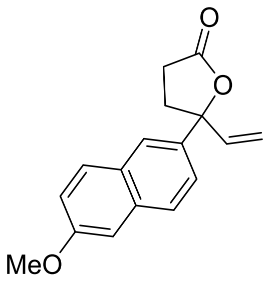

General procedure **A**: White solid; Column conditions: Hexane : EA = 10 : 1, R_f = 0.11

^{1}H NMR spectrum ($CDCl_3$)

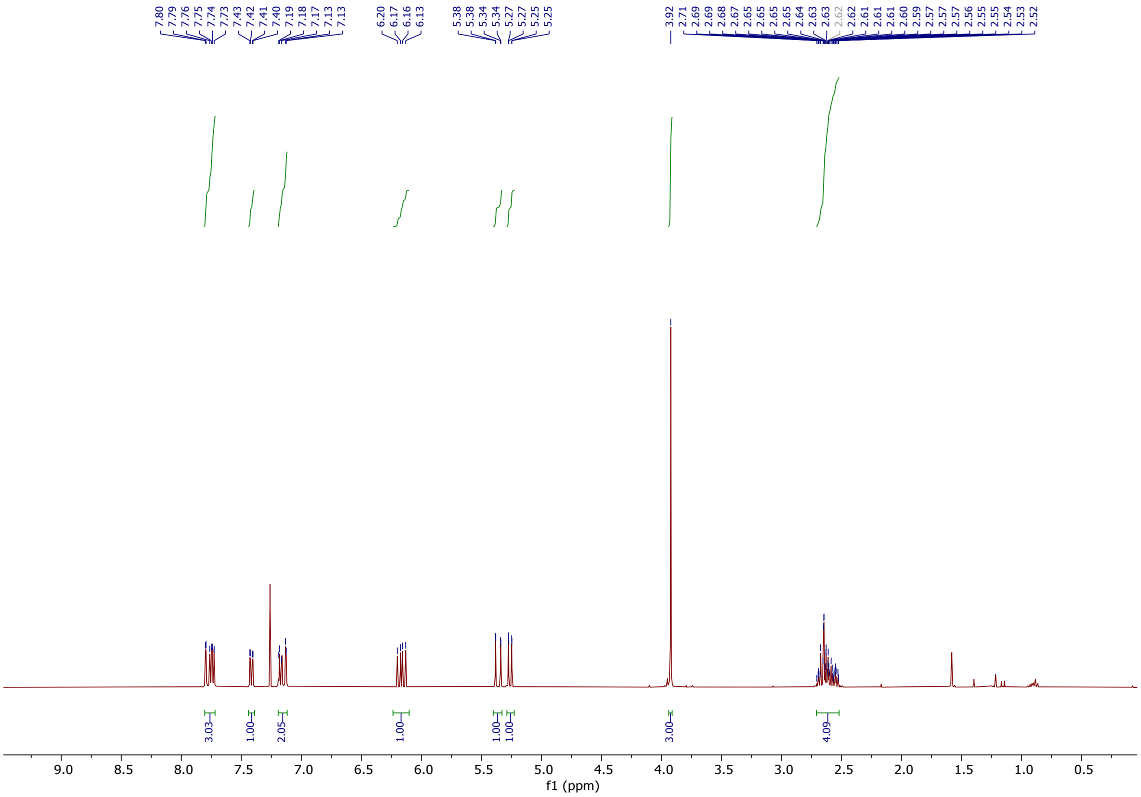

^{1}H NMR (400 MHz, $CDCl_3$) δ 7.80 – 7.72 (m, 3H), 7.42 (dd, J = 8.6, 1.9 Hz, 1H), 7.19 – 7.12 (m, 2H), 6.17 (dd, J = 17.2, 10.7 Hz, 1H), 5.36 (dd, J = 17.2, 0.7 Hz, 1H), 5.26 (dd, J = 10.7, 0.7 Hz, 1H), 3.92 (s, 3H), 2.71 – 2.52 (m, 4H).

^{13}C NMR spectrum ($CDCl_3$)

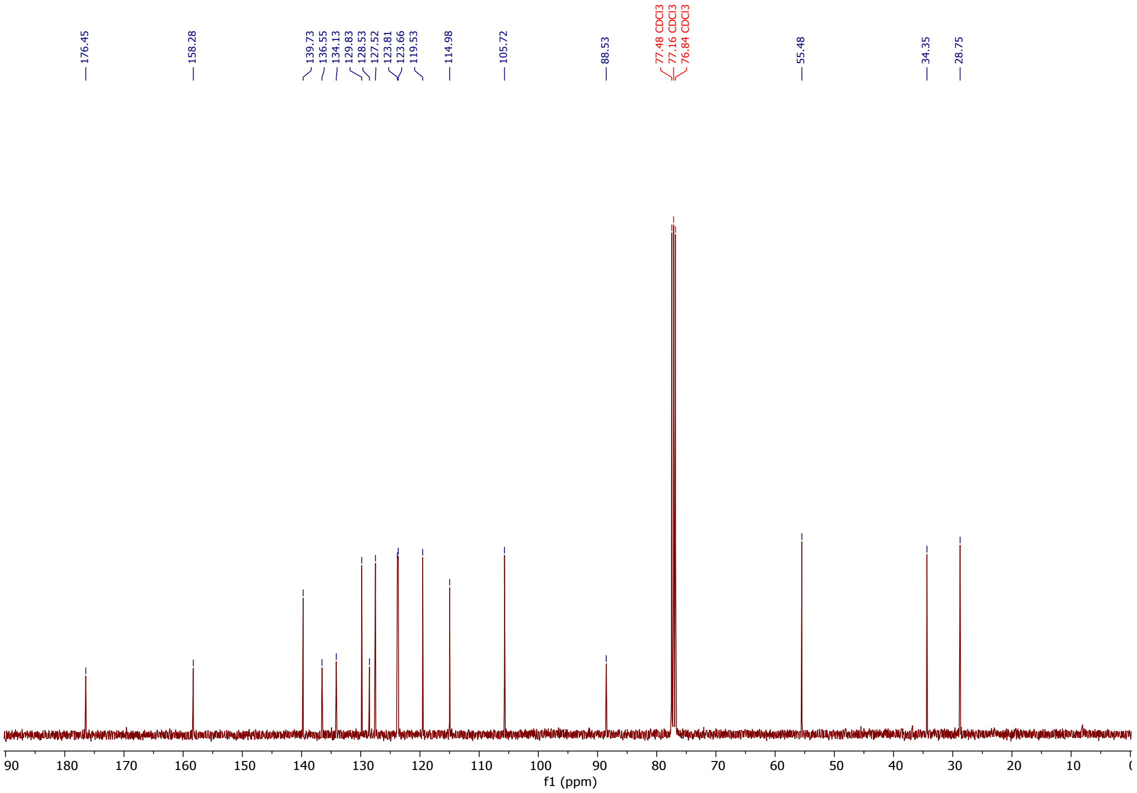

^{13}C NMR (101 MHz, $CDCl_3$) δ 176.45, 158.28, 139.73, 136.55, 134.13, 129.83, 128.53, 127.52, 123.81, 123.66, 119.53, 114.98, 105.72, 88.53, 55.48, 34.35, 28.75.

IR spectrum (neat)

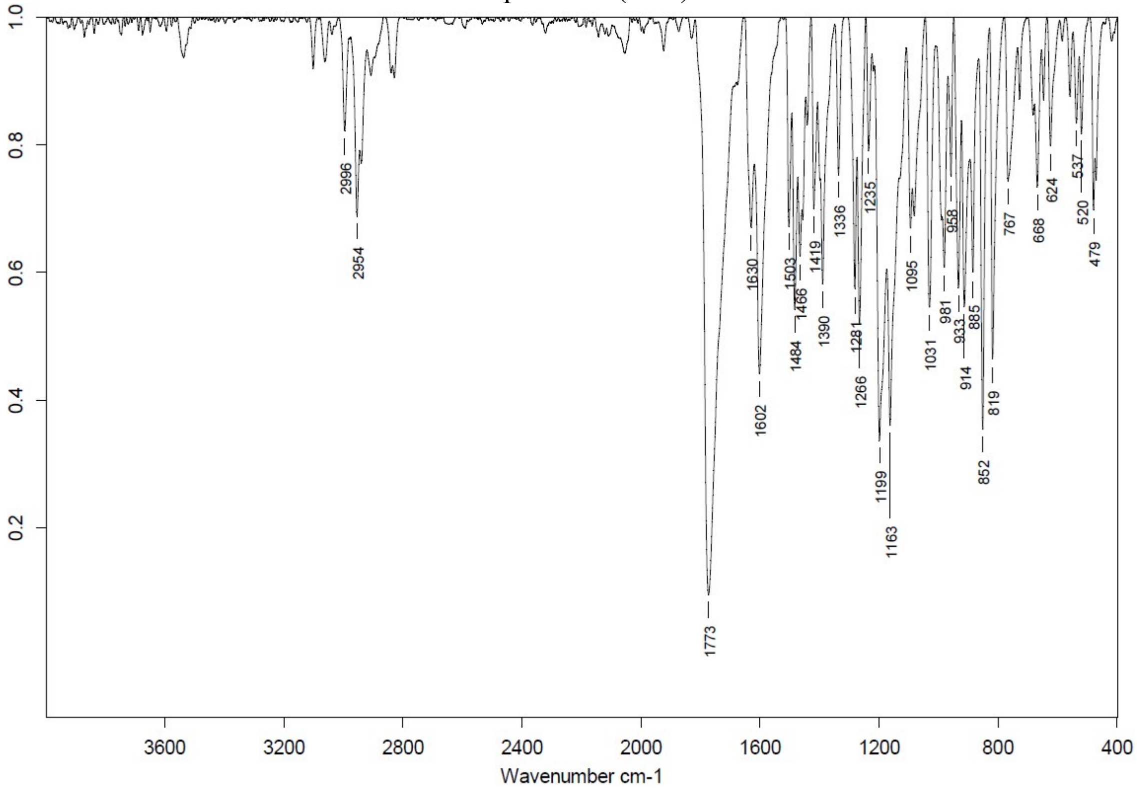

HRMS (ESI^+, MeOH): *m/z* calcd. 291.0992 $(M + Na)^+$, found: 291.0989.

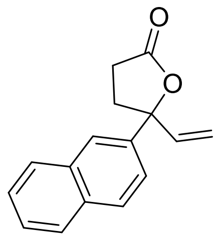

General procedure **A**: White solid; Column conditions: Hexane : EA = 10 : 1, R_f = 0.13

^{1}H NMR spectrum ($CDCl_3$)

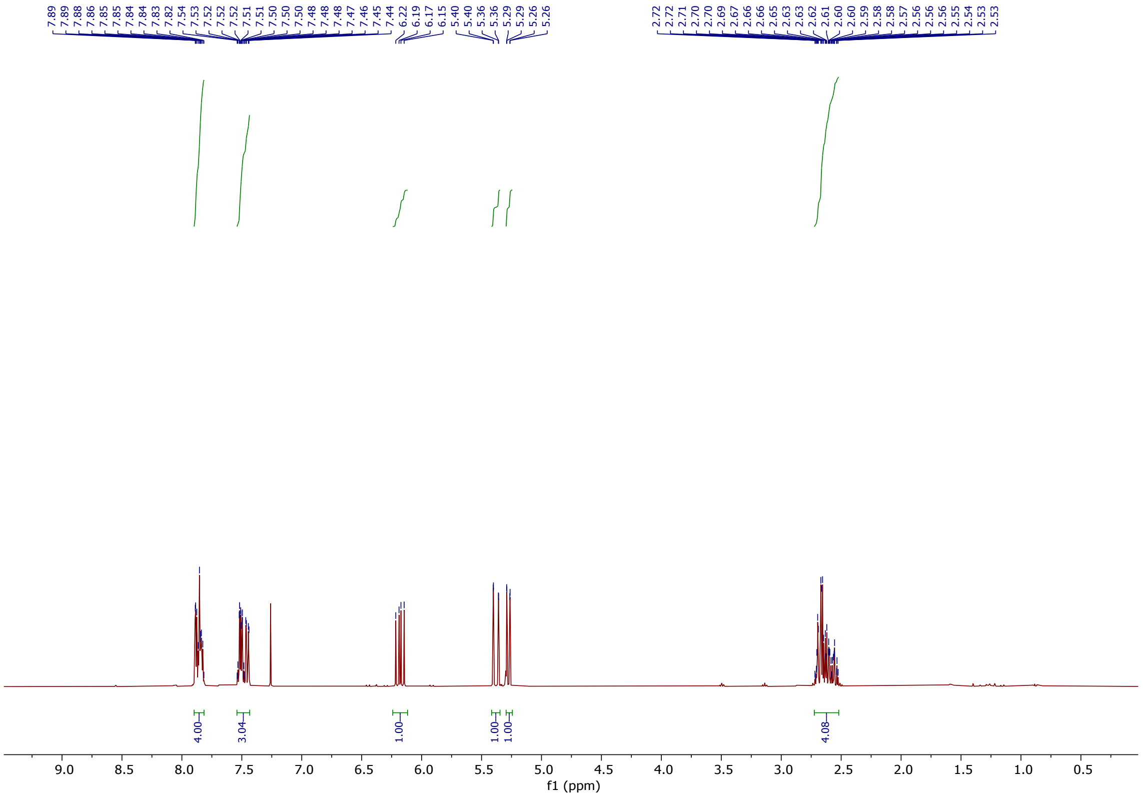

^{1}H NMR (400 MHz, $CDCl_3$) δ 7.90 – 7.82 (m, 4H), 7.54 – 7.43 (m, 3H), 6.18 (dd, J = 17.1, 10.7 Hz, 1H), 5.38 (dd, J = 17.2, 0.7 Hz, 1H), 5.28 (dd, J = 10.7, 0.7 Hz, 1H), 2.72 – 2.52 (m, 4H).

^{13}C NMR spectrum ($CDCl_3$)

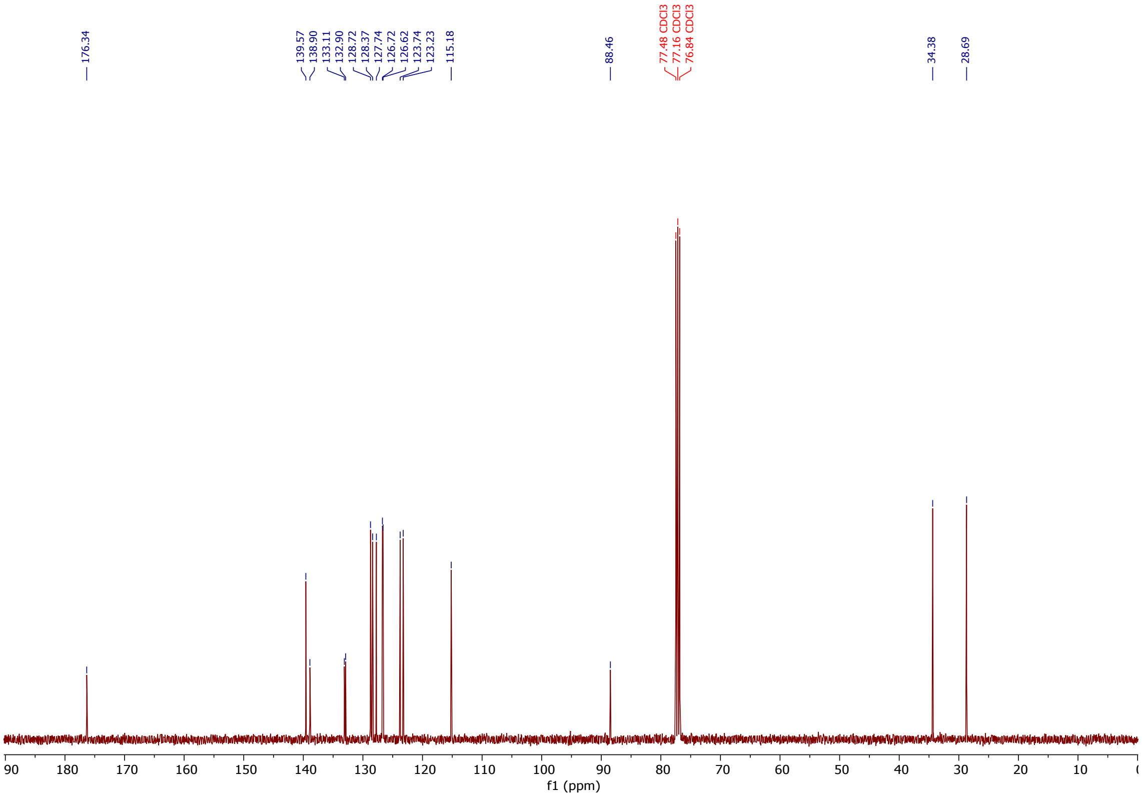

^{13}C NMR (101 MHz, $CDCl_3$) δ 176.34, 139.57, 138.90, 133.11, 132.90, 128.72, 128.37, 127.74, 126.72, 126.62, 123.74, 123.23, 115.18, 88.46, 34.38, 28.69.

IR spectrum (neat)

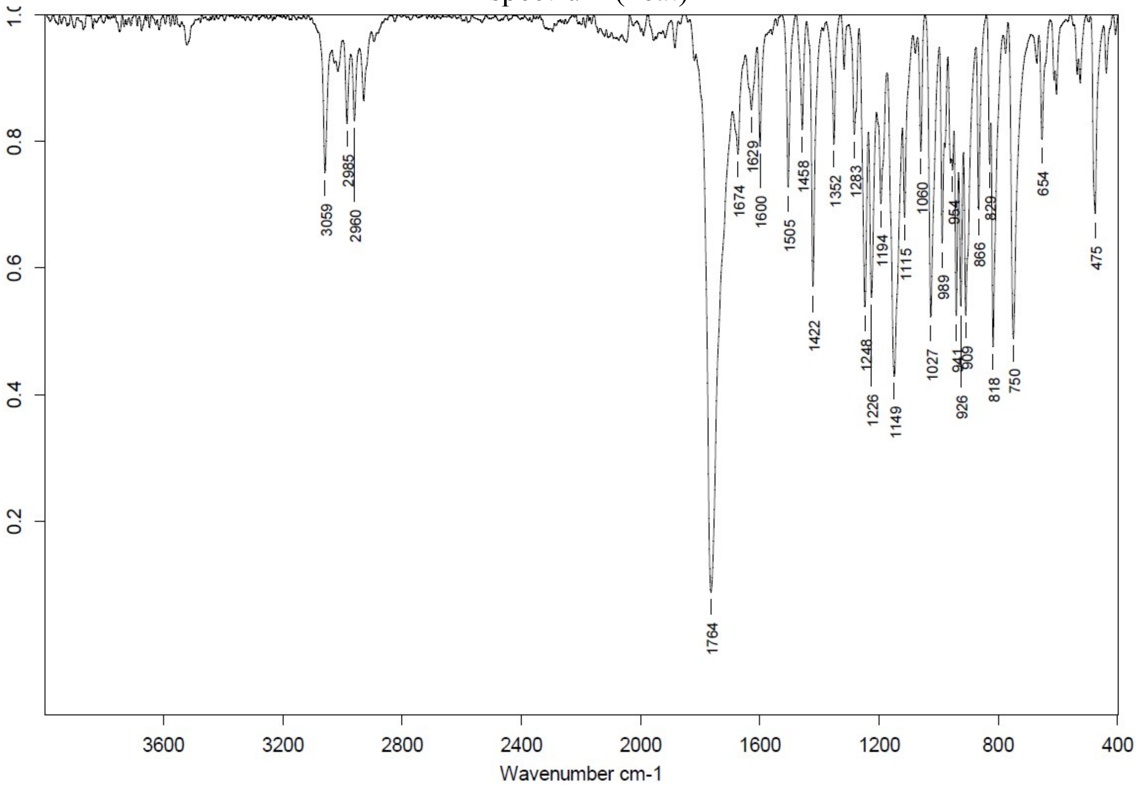

HRMS (ESI^+, MeOH): *m/z* calcd. 239.1067 $(M + H)^+$, found: 239.1059.

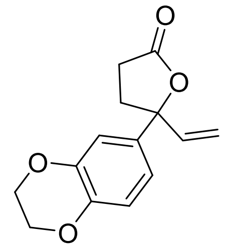

General procedure **A**: Colorless oil; Column conditions: Hexane : EA = 20 : 1, R_f = 0.15

^{1}H NMR spectrum ($CDCl_3$)

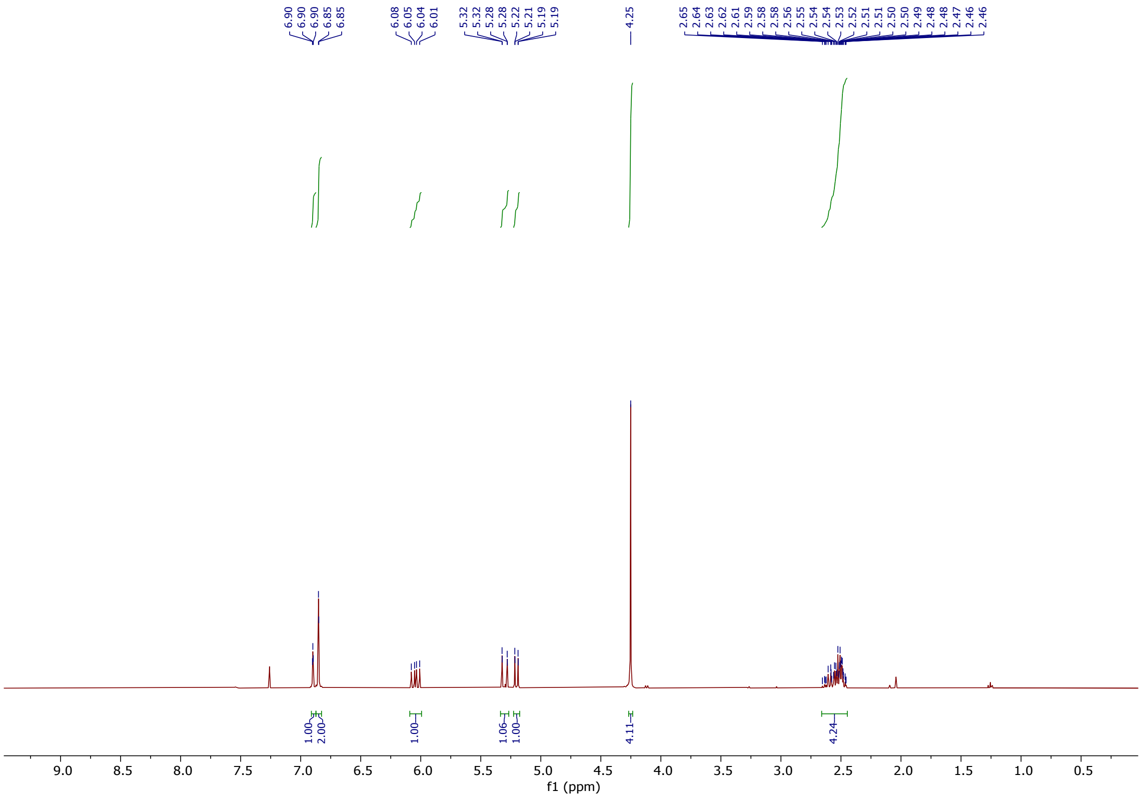

^{1}H NMR (400 MHz, $CDCl_3$) δ 6.91 – 6.87 (m, 1H), 6.86 – 6.81 (m, 2H), 6.04 (dd, J = 17.2, 10.7 Hz, 1H), 5.30 (dd, J = 17.2, 0.6 Hz, 1H), 5.20 (dd, J = 10.7, 0.6 Hz, 1H), 4.25 (s, 4H), 2.66 – 2.45 (m, 4H).

^{13}C NMR spectrum ($CDCl_3$)

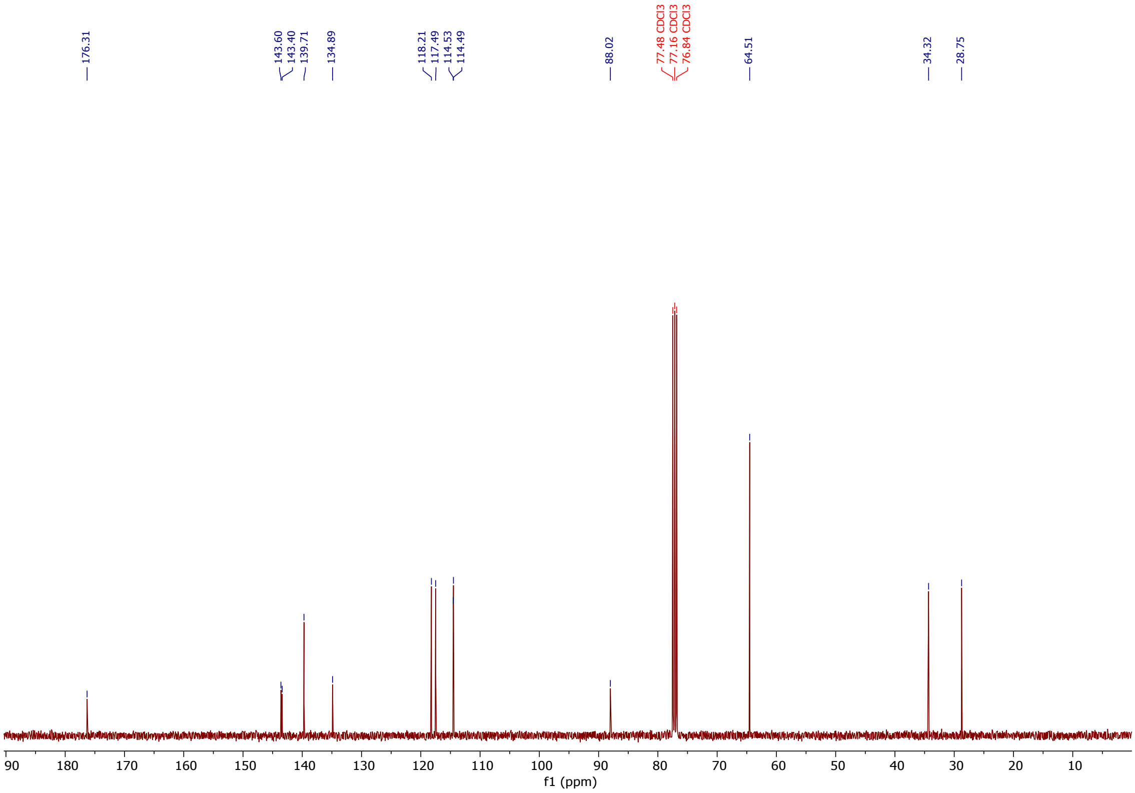

^{13}C NMR (101 MHz, $CDCl_3$) δ 176.31, 143.60, 143.40, 139.71, 134.89, 118.21, 117.49, 114.53, 114.49, 88.02, 64.51, 34.32, 28.75.

IR spectrum (neat)

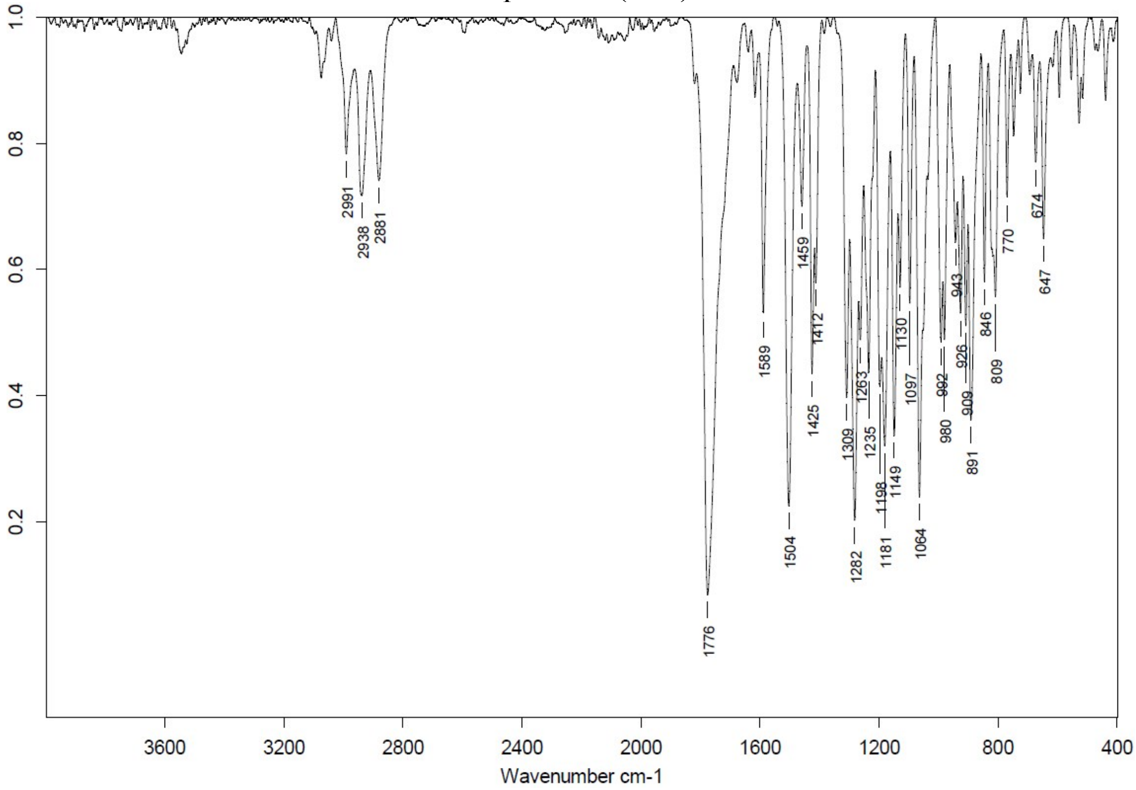

HRMS (ESI$^+$, MeOH): *m/z* calcd. 247.0965 $(M + H)^+$, found: 247.0955.

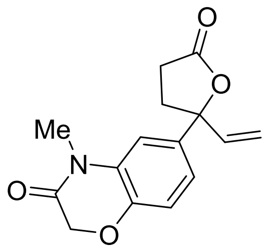

General procedure **A**: Colorless solid; Column conditions: Hexane : EA = 5 : 1, R_f = 0.10

^{1}H NMR spectrum ($CDCl_3$)

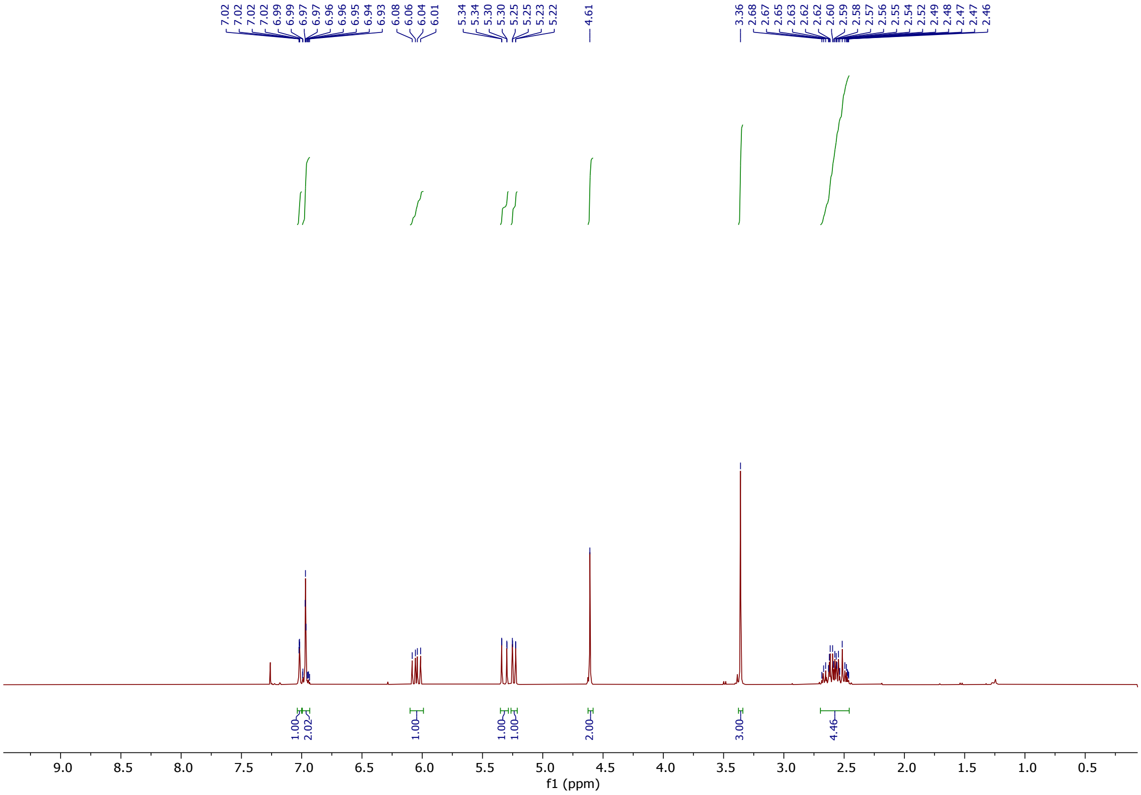

^{1}H NMR (400 MHz, $CDCl_3$) δ 7.02 (dd, J = 1.7, 0.8 Hz, 1H), 6.99 – 6.93 (m, 2H), 6.05 (dd, J = 17.2, 10.7 Hz, 1H), 5.32 (dd, J = 17.1, 0.6 Hz, 1H), 5.24 (dd, J = 10.7, 0.7 Hz, 1H), 4.61 (s, 2H), 3.36 (s, 3H), 2.70 – 2.46 (m, 4H).

^{13}C NMR spectrum ($CDCl_3$)

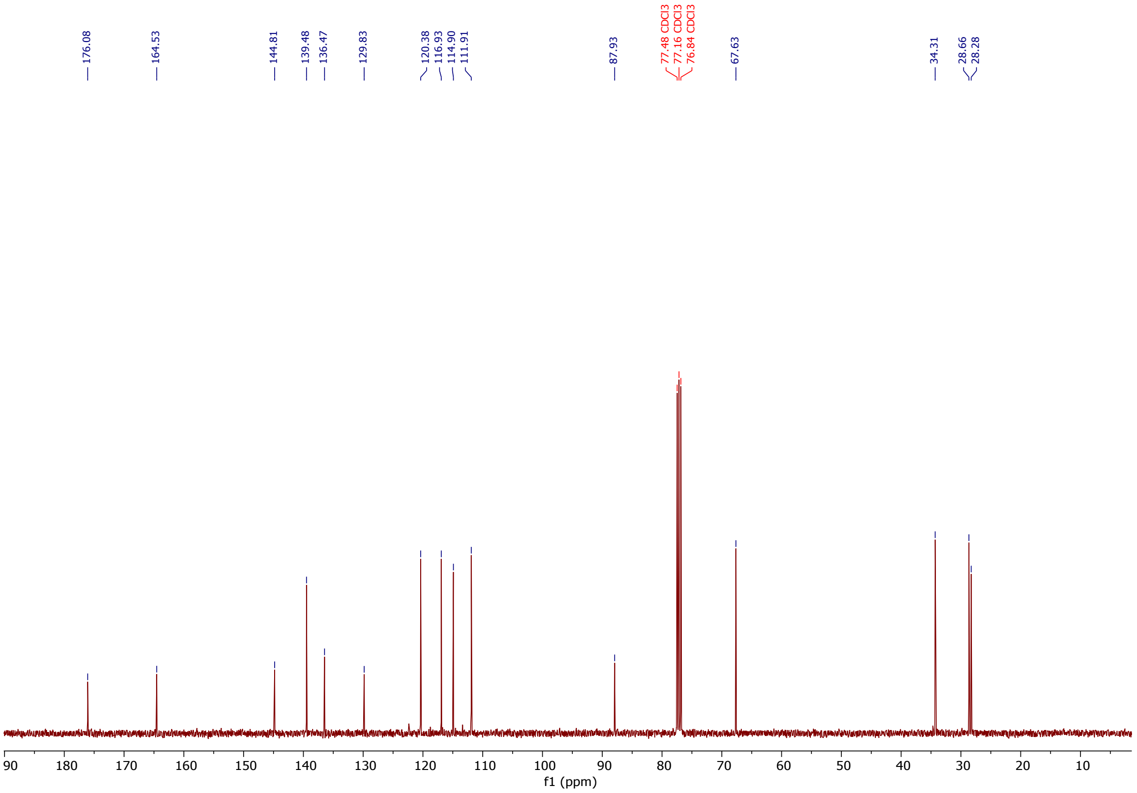

^{13}C NMR (101 MHz, $CDCl_3$) δ 176.08, 164.53, 144.81, 139.48, 136.47, 129.83, 120.38, 116.93, 114.90, 111.91, 87.93, 67.63, 34.31, 28.66, 28.28.

IR spectrum (neat)

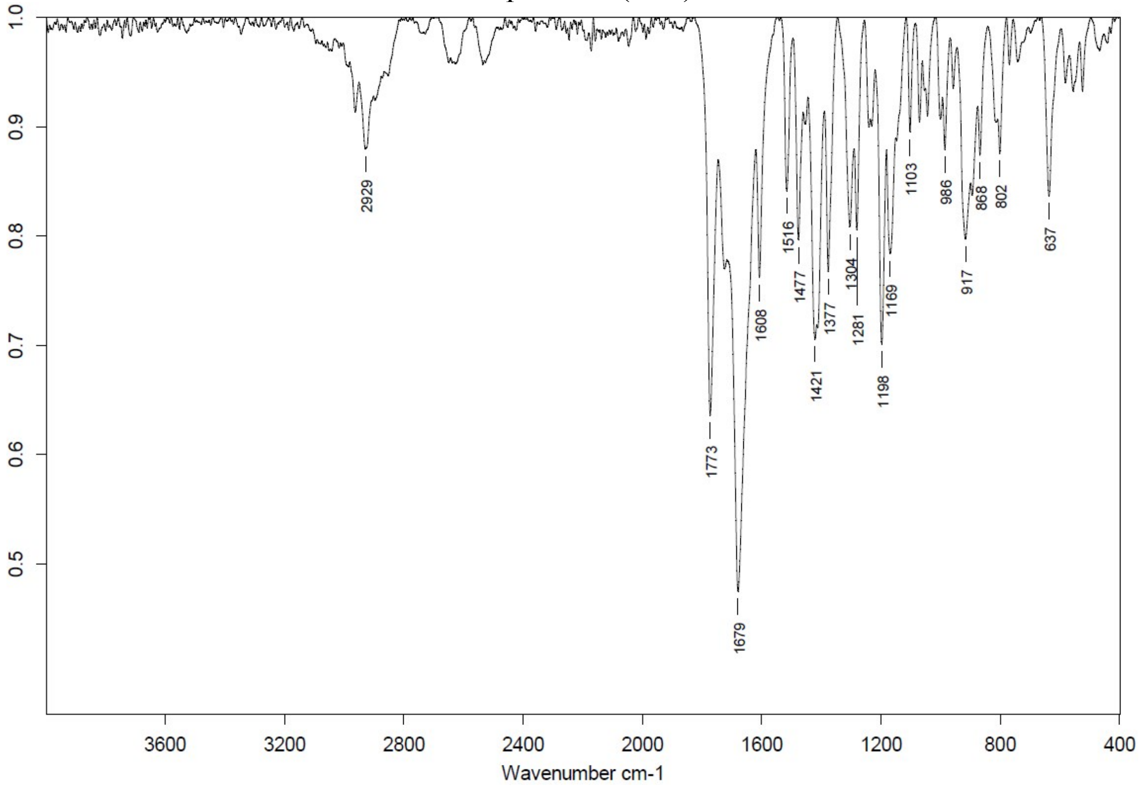

HRMS (ESI$^+$, MeOH): *m/z* calcd. 274.1074 $(M + H)^+$, found: 274.1075.

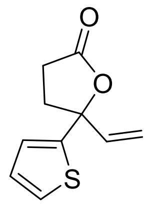

General procedure **B**: Yellow oil; Column conditions: Hexane : EA = 20 : 1, R_f = 0.15

^{1}H NMR spectrum ($CDCl_3$)

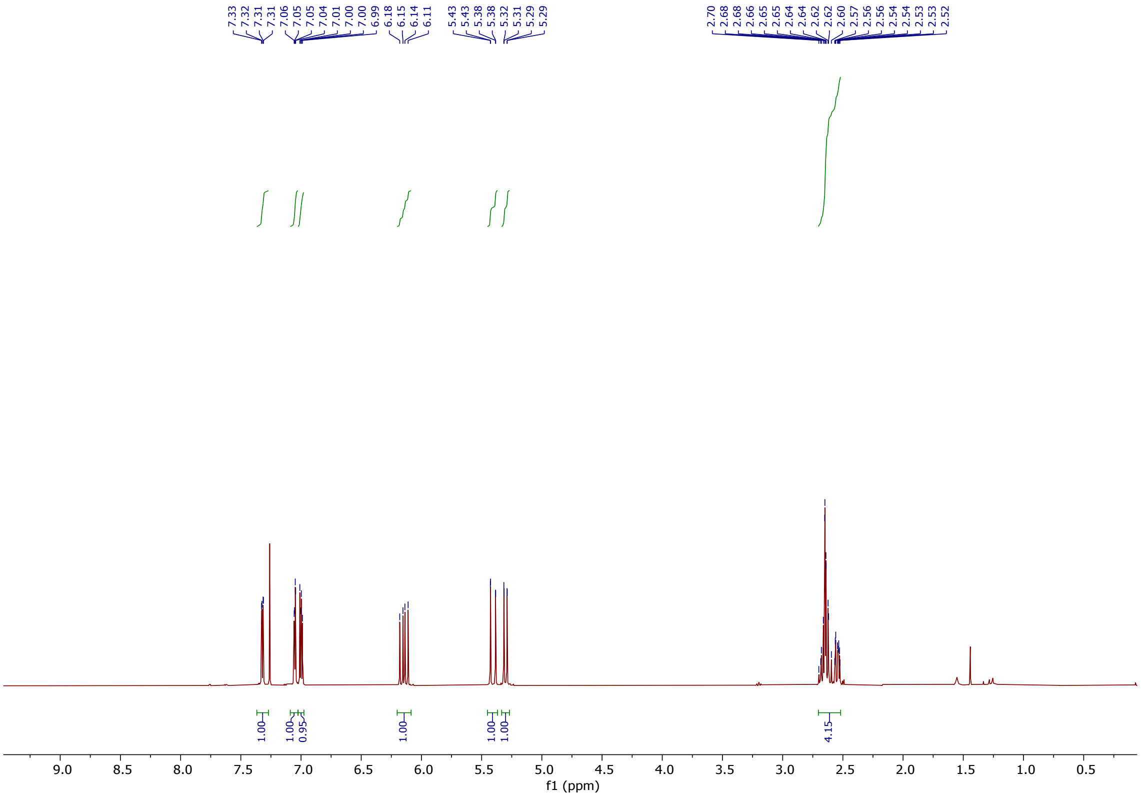

^{1}H NMR (400 MHz, $CDCl_3$) δ 7.32 (dd, J = 5.1, 1.3 Hz, 1H), 7.08 – 7.02 (m, 1H), 7.00 (dd, J = 5.0, 3.6 Hz, 1H), 6.15 (dd, J = 17.1, 10.7 Hz, 1H), 5.41 (dd, J = 17.1, 0.6 Hz, 1H), 5.30 (dd, J = 10.6, 0.6 Hz, 1H), 2.70 – 2.52 (m, 4H).

^{13}C NMR spectrum ($CDCl_3$)

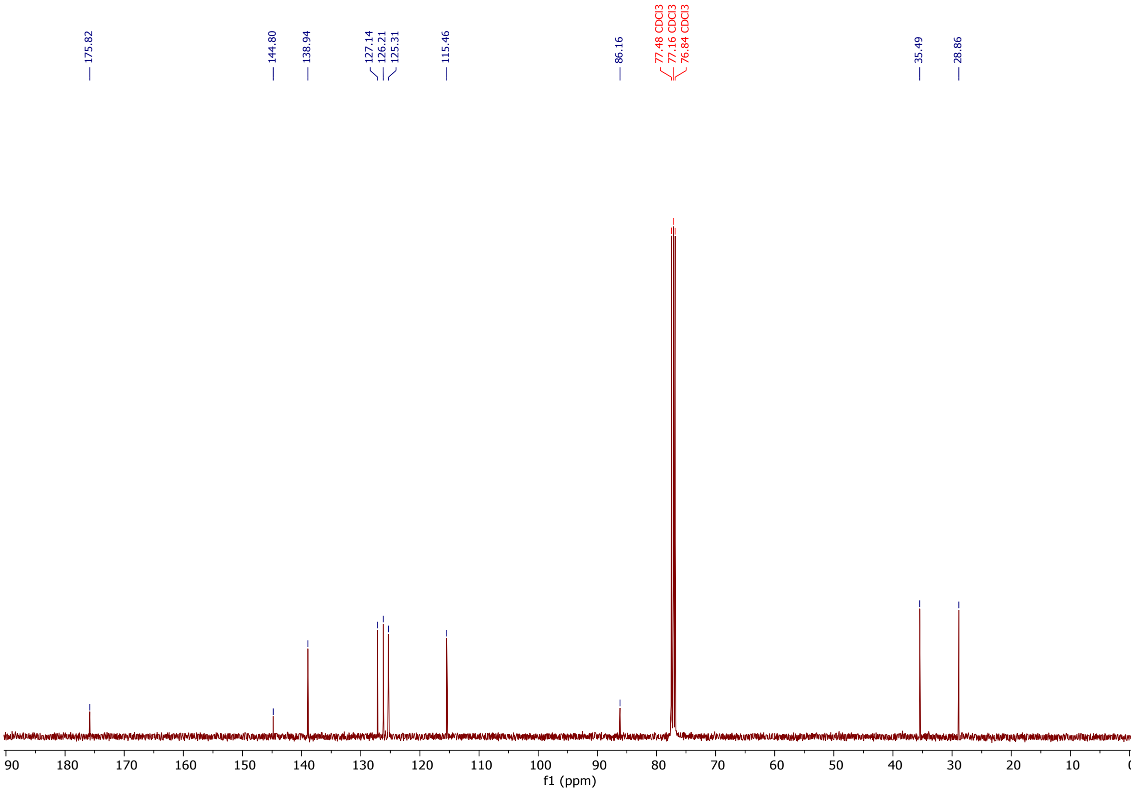

^{13}C NMR (101 MHz, $CDCl_3$) δ 175.82, 144.80, 138.94, 127.14, 126.21, 125.31, 115.46, 86.16, 35.49, 28.86.

IR spectrum (neat)

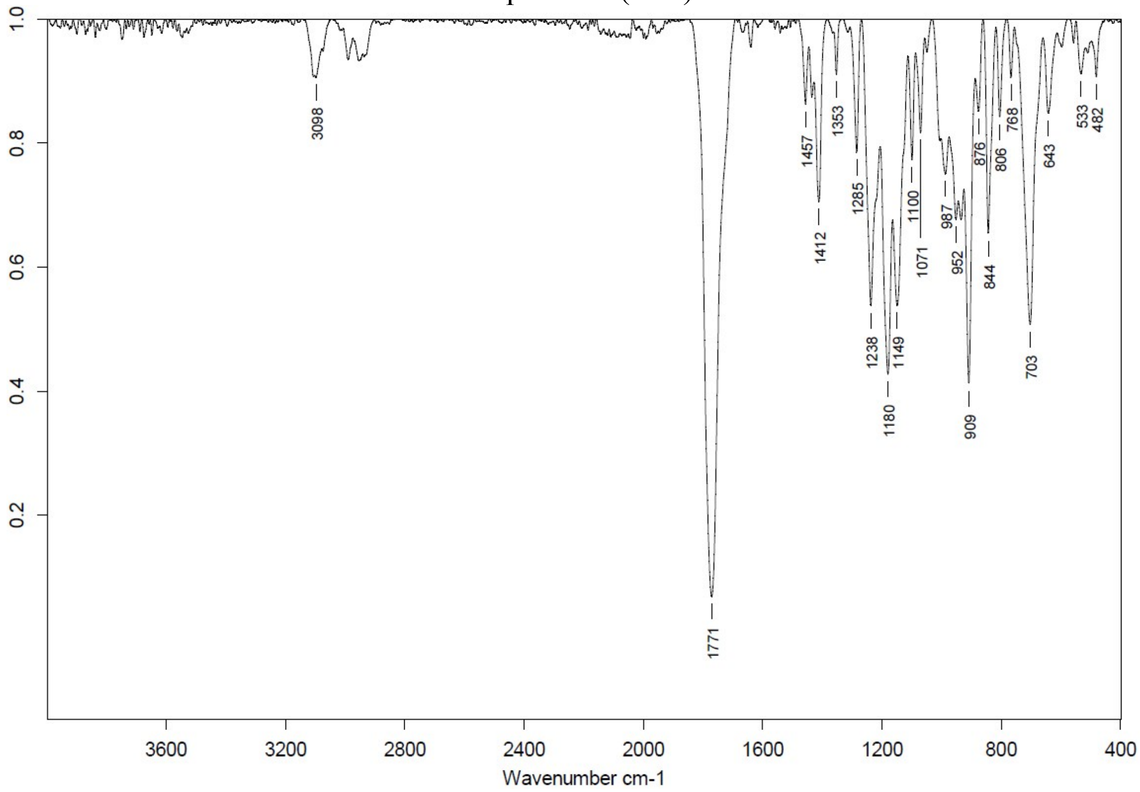

HRMS (ESI$^+$, MeOH): *m/z* calcd. 195.0474 $(M + H)^+$, found: 195.0471.

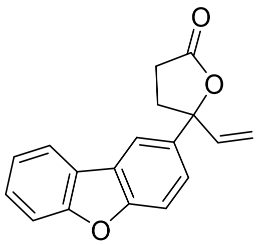

General procedure **A**: White solid; Column conditions: Hexane : EA = 10 : 1, R_f = 0.10

^{1}H NMR spectrum ($CDCl_3$)

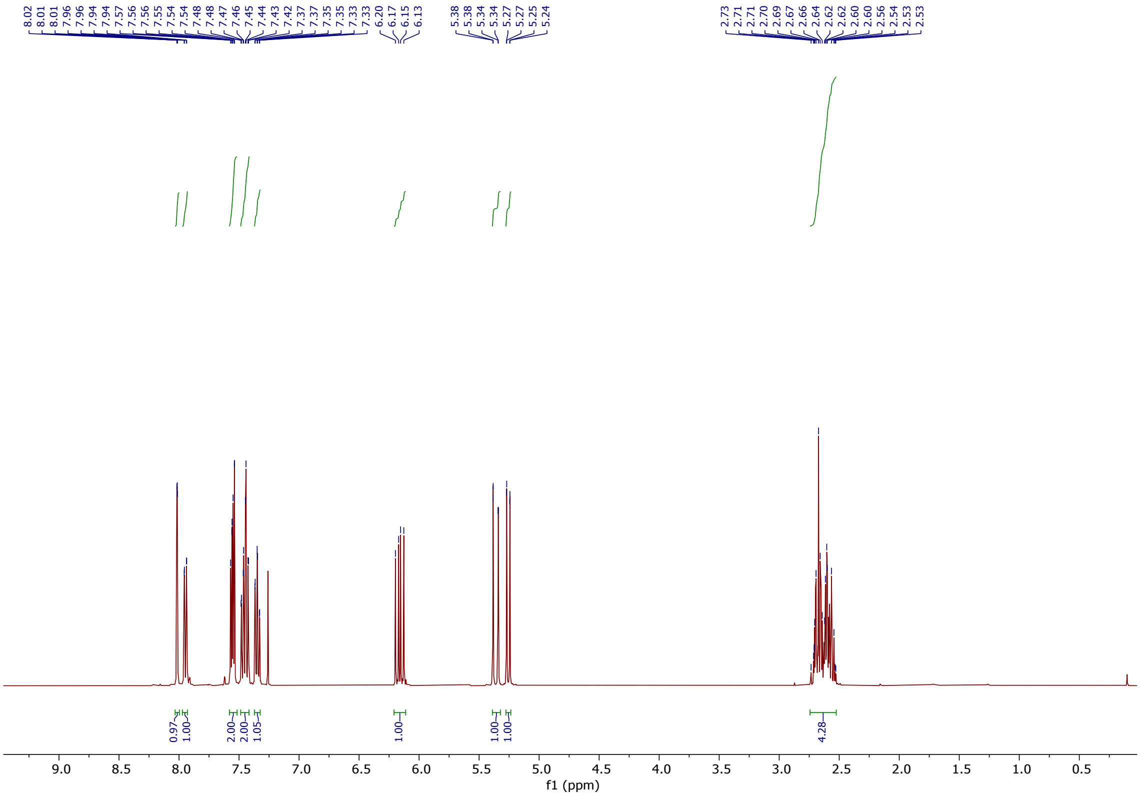

^{1}H NMR (400 MHz, $CDCl_3$) δ 8.03 – 8.00 (m, 1H), 7.98 – 7.92 (m, 1H), 7.58 – 7.52 (m, 2H), 7.49 – 7.42 (m, 2H), 7.37 – 7.32 (m, 1H), 6.16 (dd, *J* = 17.2, 10.7 Hz, 1H), 5.36 (dd, *J* = 17.2, 0.7 Hz, 1H), 5.26 (dd, *J* = 10.7, 0.7 Hz, 1H), 2.74 – 2.52 (m, 4H).

^{13}C NMR spectrum ($CDCl_3$)

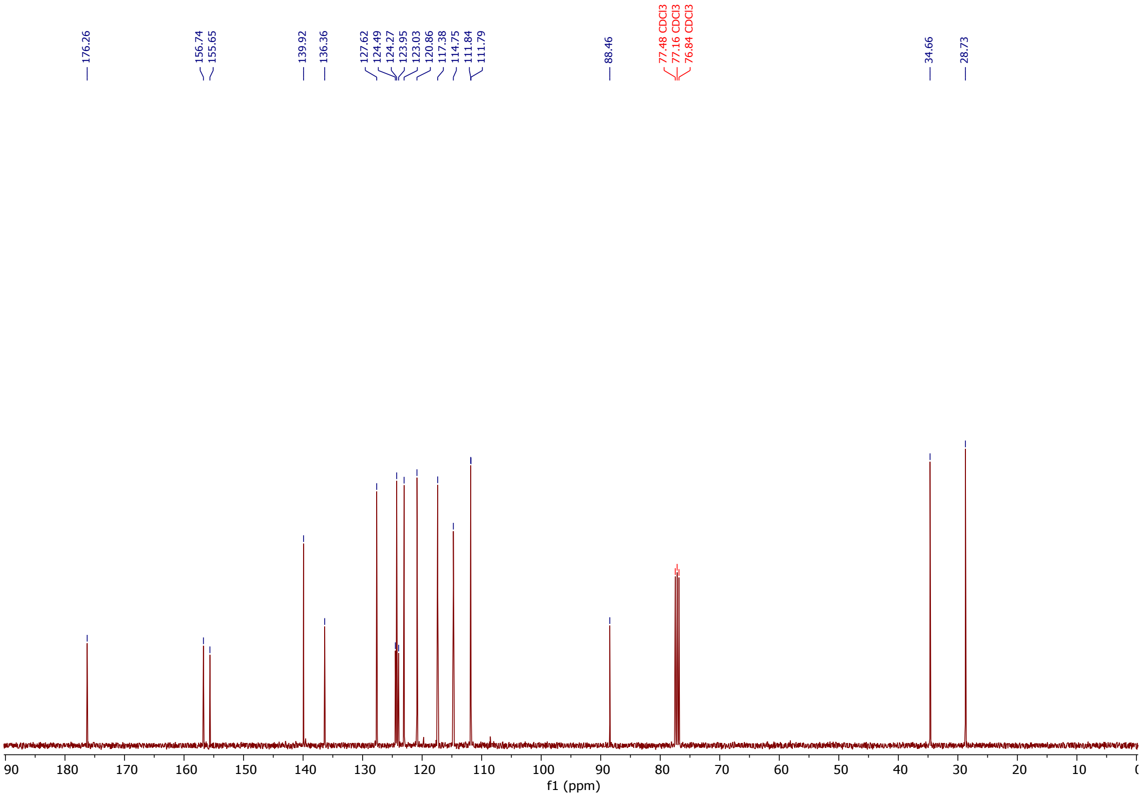

^{13}C NMR (101 MHz, $CDCl_3$) δ 176.26, 156.74, 155.65, 139.92, 136.36, 127.62, 124.49, 124.27, 123.95, 123.03, 120.86, 117.38, 114.75, 111.84, 111.79, 88.46, 34.66, 28.73.

IR spectrum (neat)

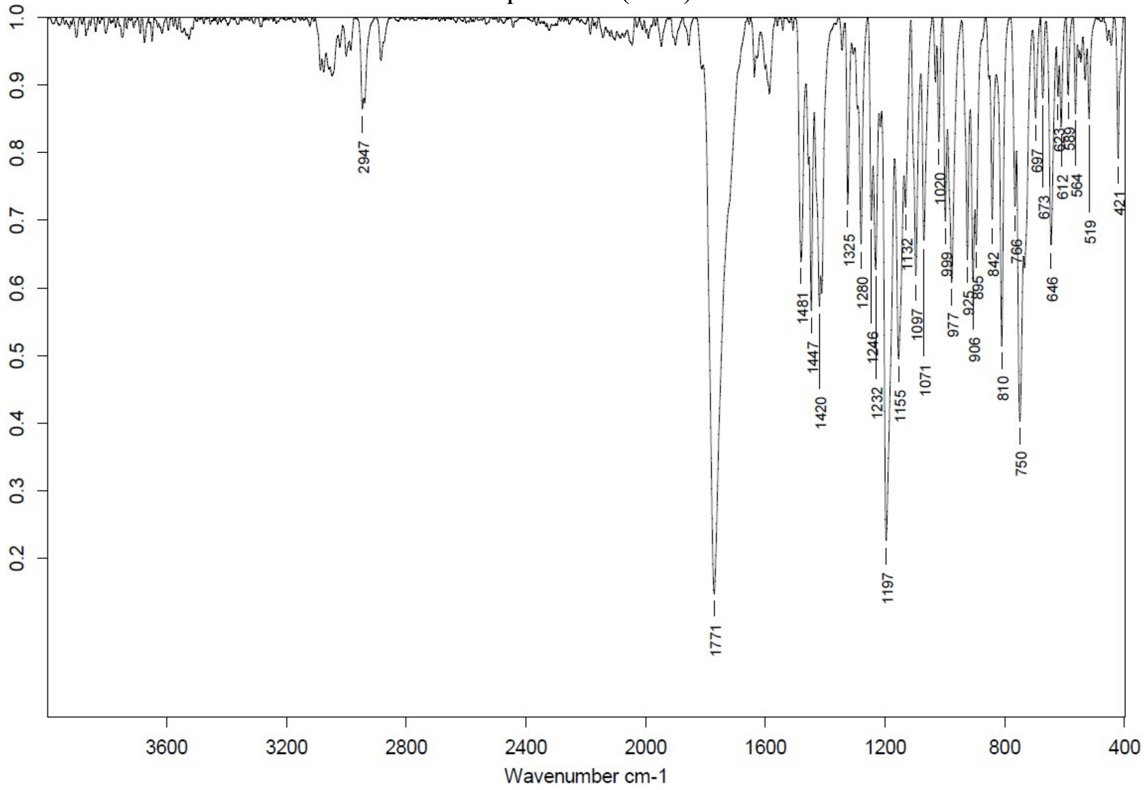

HRMS (ESI$^+$, MeOH): *m/z* calcd. 301.0835 $(M + Na)^+$, found: 301.0841.

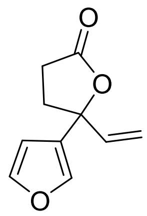

General procedure **B**: Colorless oil; Column conditions: Hexane : DCM = 1 : 1, R_f = 0.13

^{1}H NMR spectrum ($CDCl_3$)

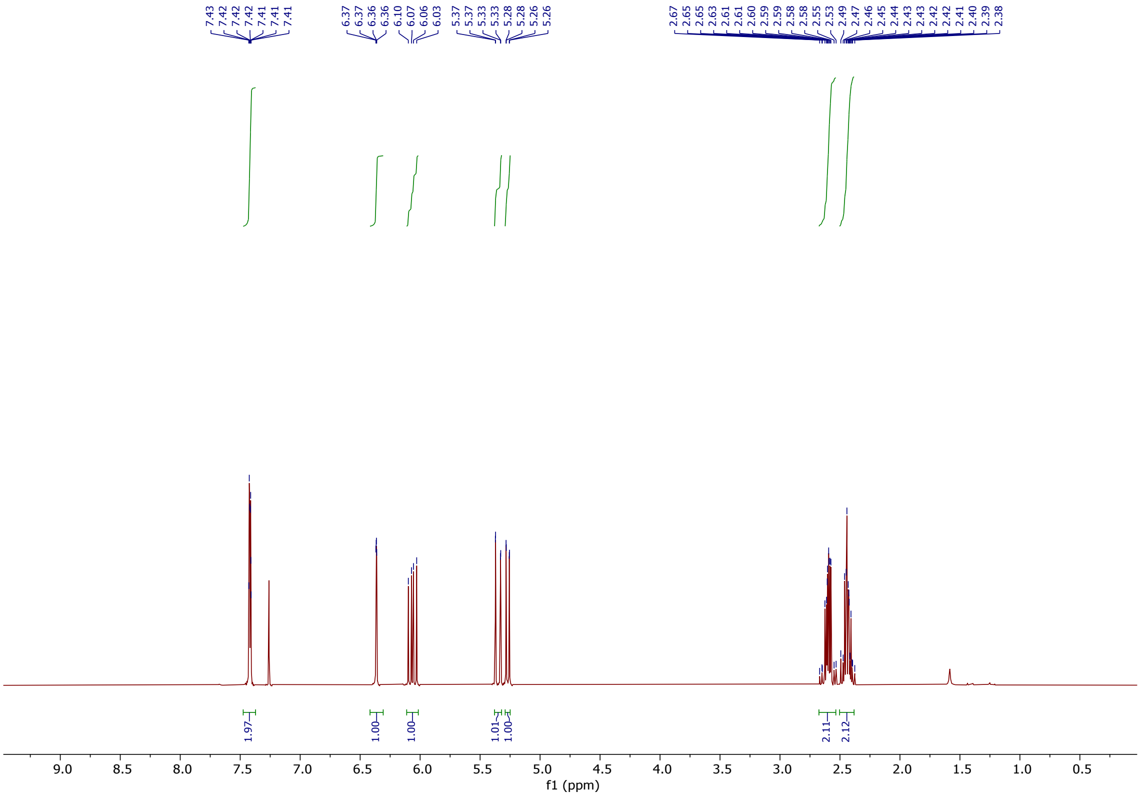

^{1}H NMR (400 MHz, $CDCl_3$) δ 7.48 – 7.37 (m, 2H), 6.37 (dd, J = 1.9, 1.0 Hz, 1H), 6.06 (dd, J = 17.2, 10.7 Hz, 1H), 5.35 (dd, J = 17.1, 0.7 Hz, 1H), 5.27 (dd, J = 10.7, 0.7 Hz, 1H), 2.68 – 2.53 (m, 2H), 2.50 – 2.38 (m, 2H).

^{13}C NMR spectrum (CDCl$_3$)

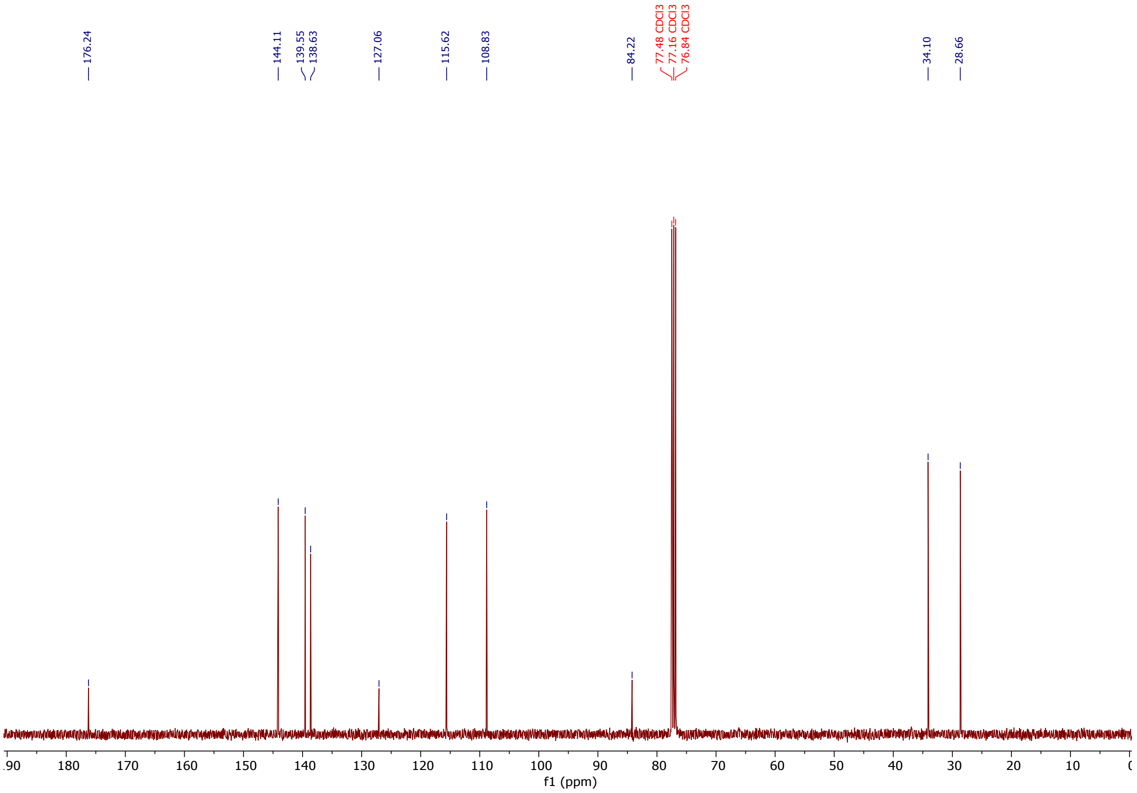

^{13}C NMR (101 MHz, CDCl$_3$) δ 176.24, 144.11, 139.55, 138.63, 127.06, 115.62, 108.83, 84.22, 34.10, 28.66.

IR spectrum (neat)

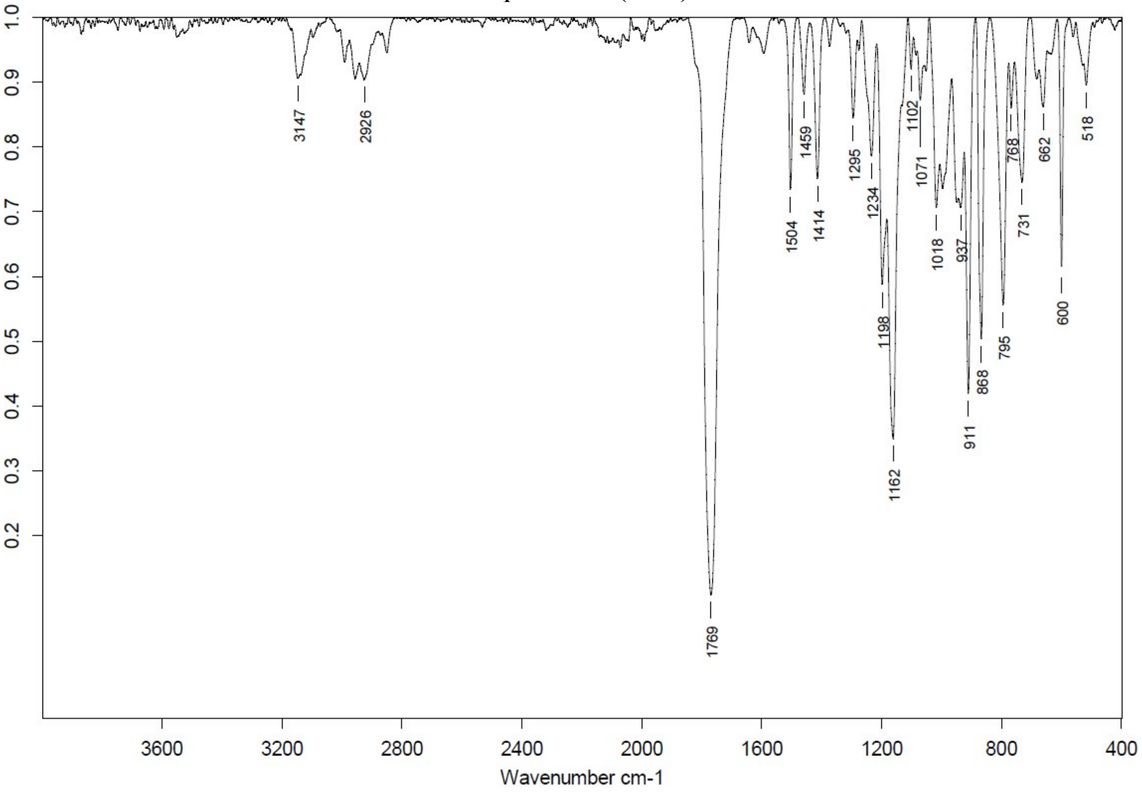

HRMS (ESI$^+$, MeOH): *m/z* calcd. 179.0703 (M + H)$^+$, found: 179.0700.

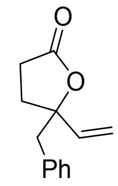

General procedure **B**: Yellow oil; Column conditions: Hexane : EA = 20 : 1, R_f = 0.18

^{1}H NMR spectrum ($CDCl_3$)

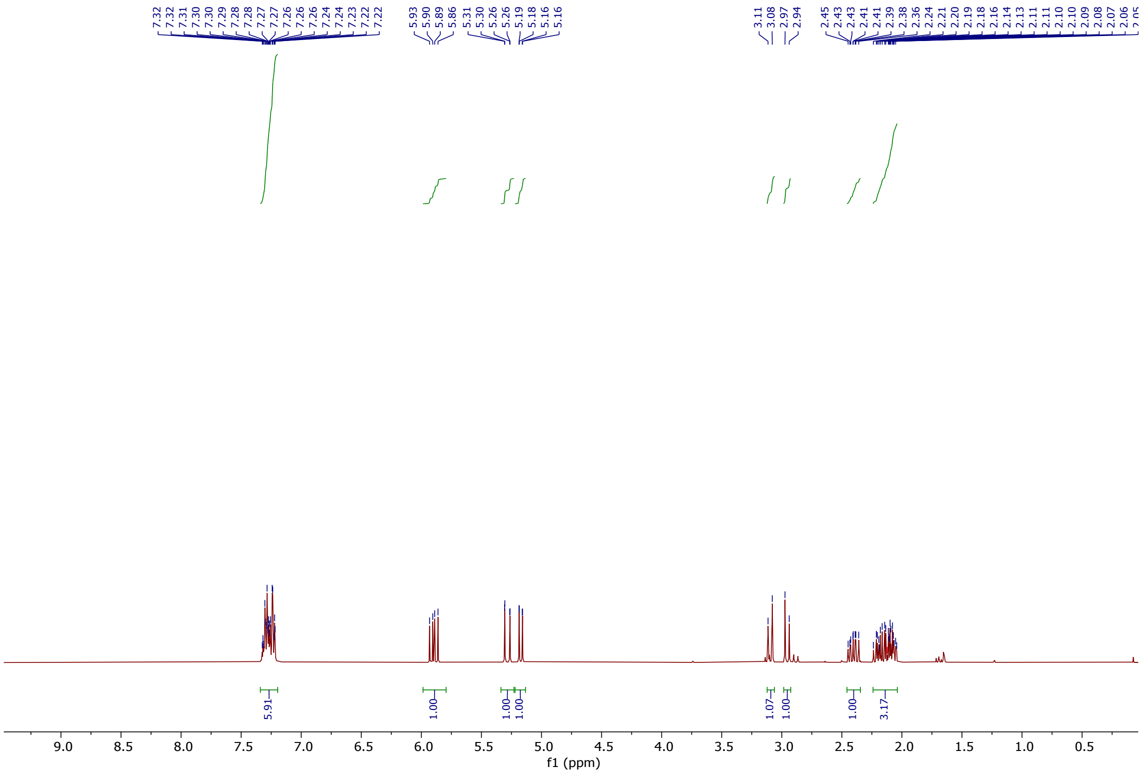

^{1}H NMR (400 MHz, $CDCl_3$) δ 7.34 – 7.20 (m, 5H), 5.90 (dd, J = 17.2, 10.9 Hz, 1H), 5.28 (dd, J = 17.2, 0.9 Hz, 1H), 5.17 (dd, J = 10.9, 0.9 Hz, 1H), 3.10 (d, J = 14.0 Hz, 1H), 2.96 (d, J = 14.0 Hz, 1H), 2.46 – 2.35 (m, 1H), 2.24 – 2.04 (m, 3H).

^{13}C NMR spectrum ($CDCl_3$)

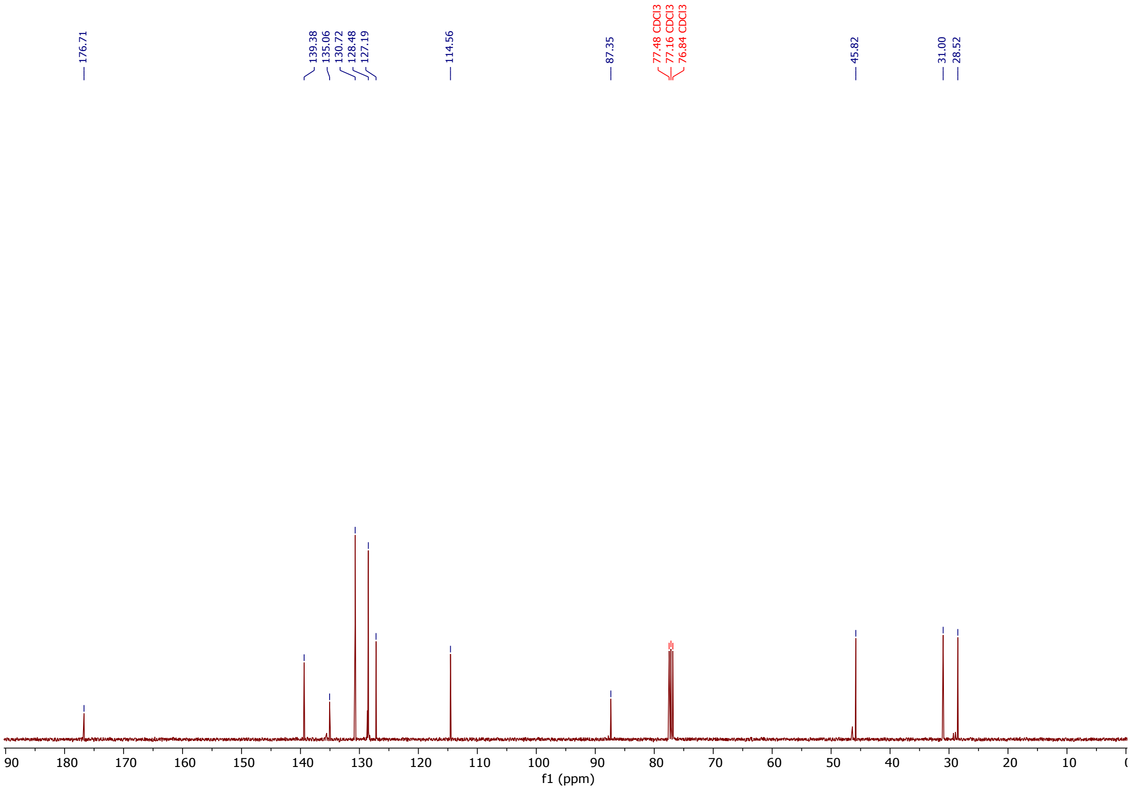

^{13}C NMR (101 MHz, $CDCl_3$) δ 176.71, 139.38, 135.06, 130.72, 128.48, 127.19, 114.56, 87.35, 45.82, 31.00, 28.52.

IR spectrum (neat)

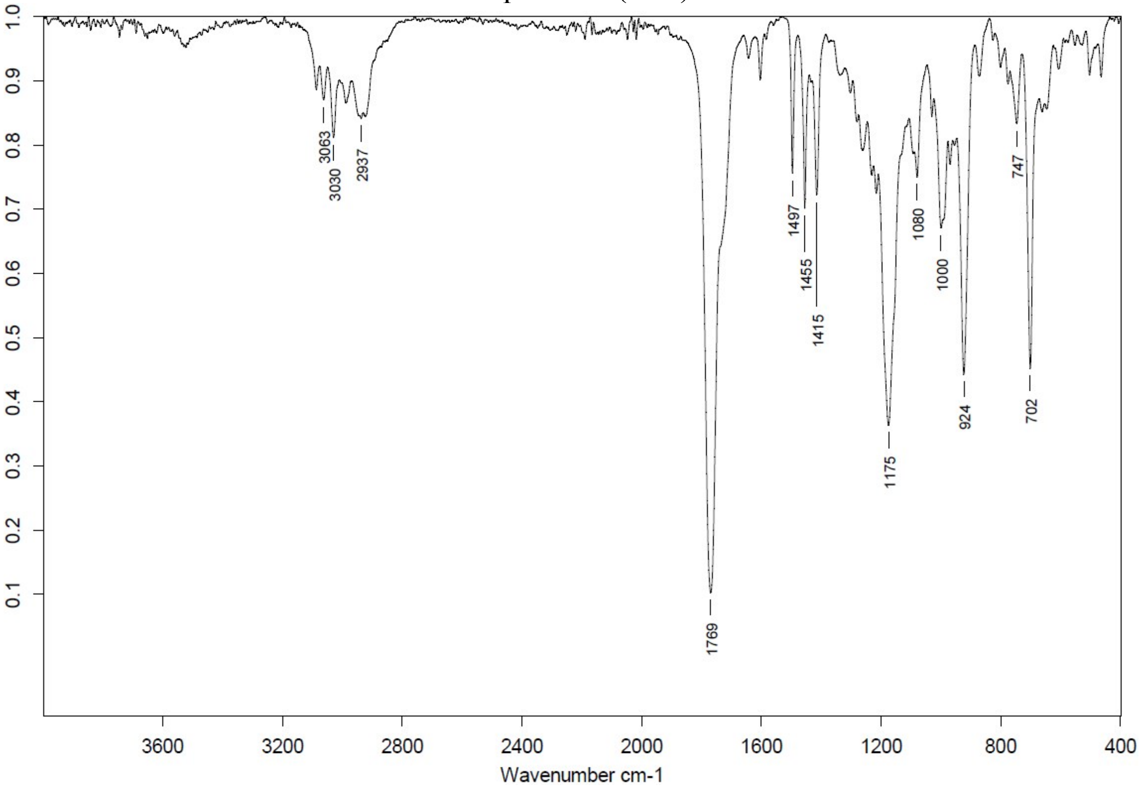

HRMS (ESI$^+$, MeOH): *m/z* calcd. 225.0886 $(M + Na)^+$, found: 225.0887.

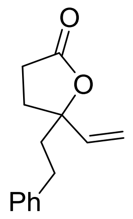

General procedure **B**: Light yellow oil; Column conditions: Hexane : EA = 20 : 1, R_f = 0.15

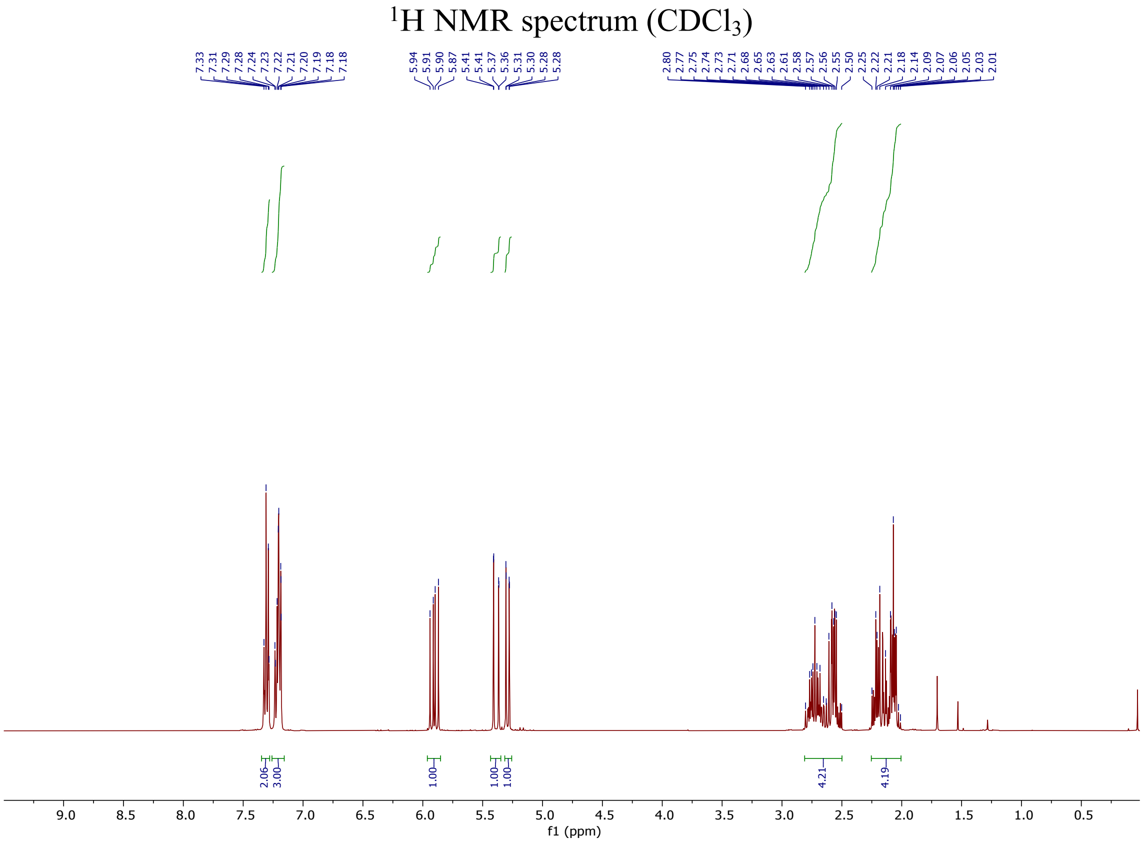

^{1}H NMR (400 MHz, $CDCl_3$) δ 7.35 – 7.28 (m, 2H), 7.26 – 7.16 (m, 3H), 5.90 (dd, J = 17.3, 10.9 Hz, 1H), 5.39 (dd, J = 17.2, 0.9 Hz, 1H), 5.29 (dd, J = 10.9, 0.9 Hz, 1H), 2.81 – 2.50 (m, 4H), 2.25 – 2.01 (m, 4H).

^{13}C NMR spectrum ($CDCl_3$)

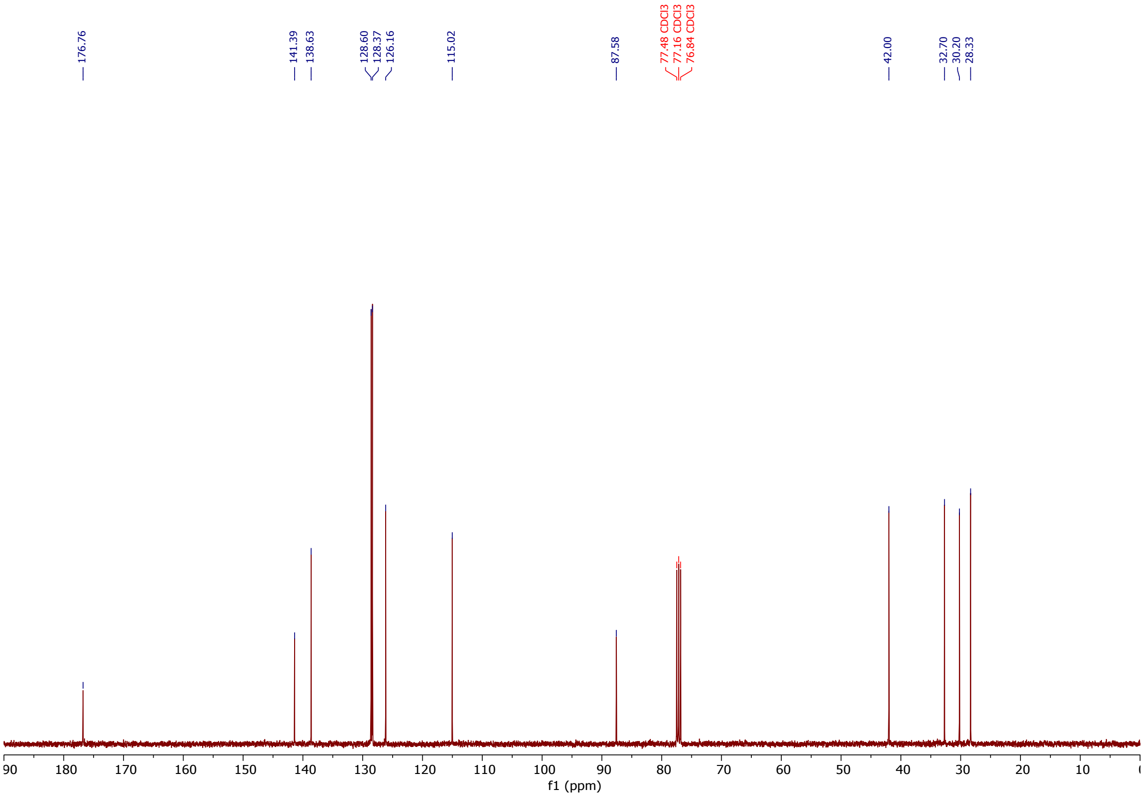

^{13}C NMR (101 MHz, $CDCl_3$) δ 176.76, 141.39, 138.63, 128.60, 128.37, 126.16, 115.02, 87.58, 42.00, 32.70, 30.20, 28.33.

IR spectrum (neat)

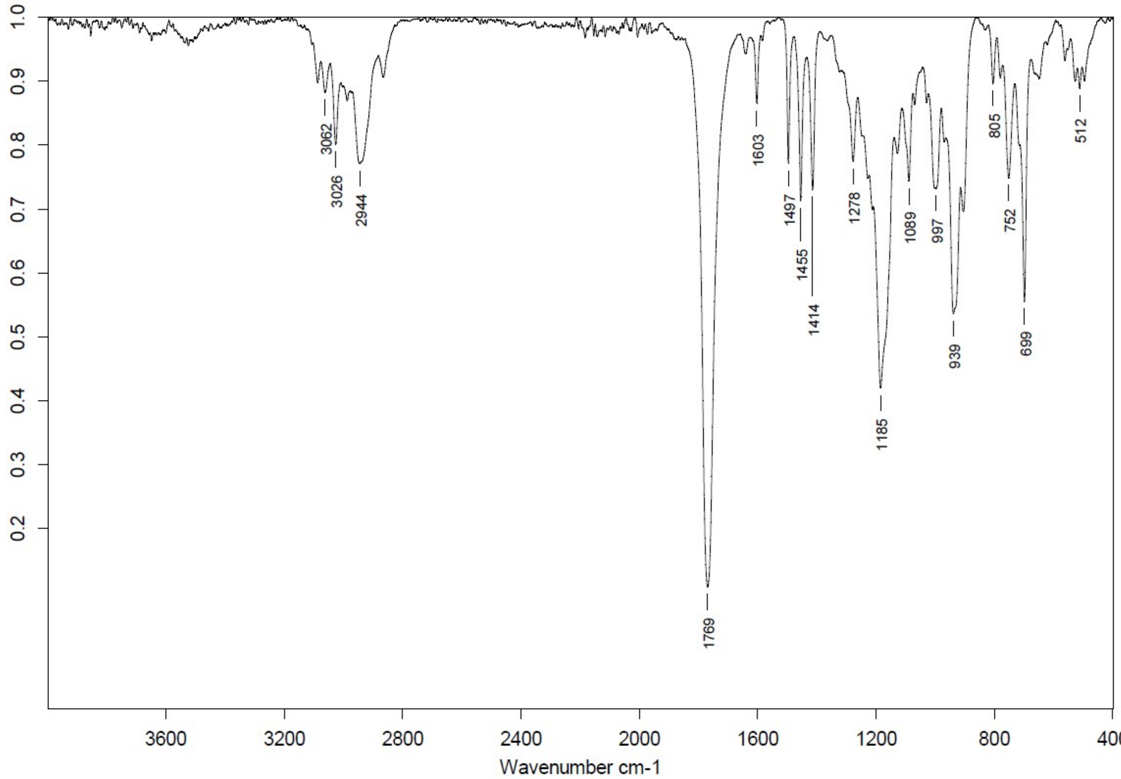

HRMS (ESI^+, MeOH): *m/z* calcd. 239.1043 $(M + Na)^+$, found: 239.1052.

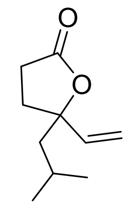

General procedure **A**: Light yellow oil; Column conditions: Hexane : EA = 20 : 1, R_f = 0.15

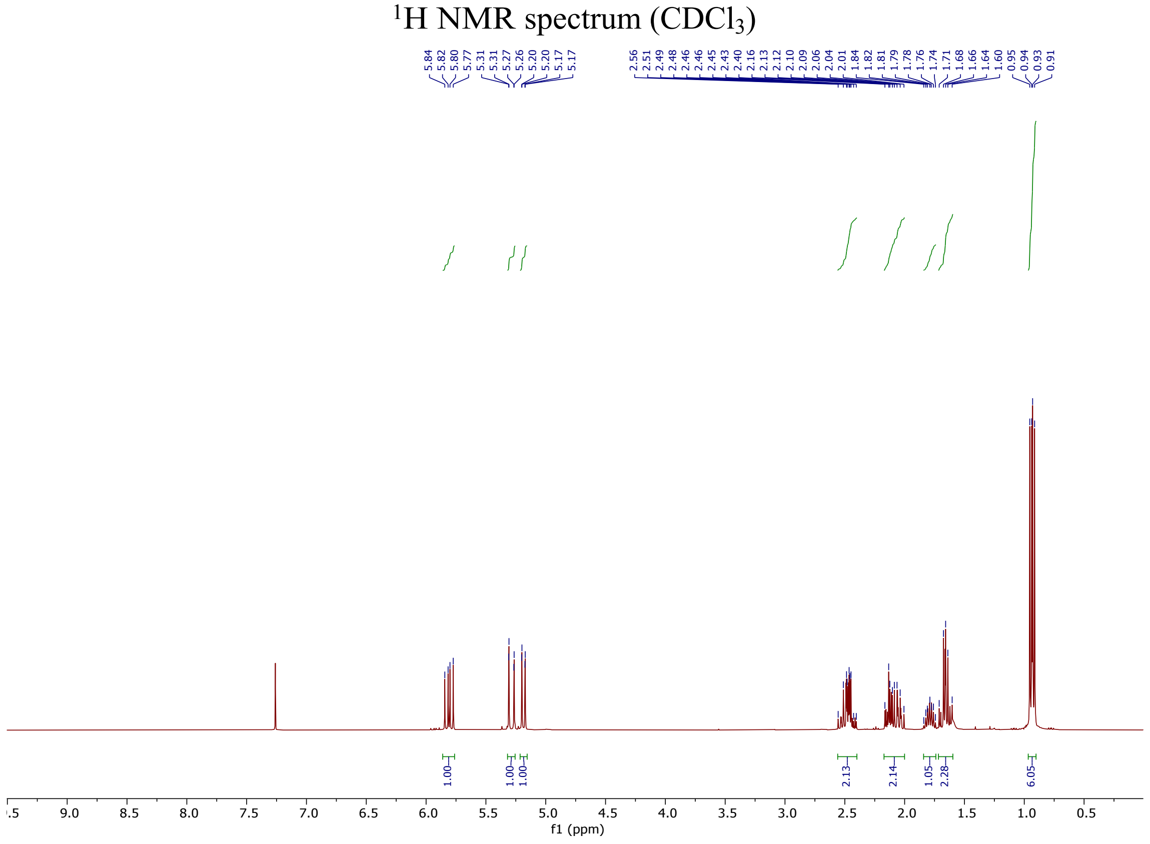

^{1}H NMR (400 MHz, $CDCl_3$) δ 5.81 (dd, J = 17.2, 10.9 Hz, 1H), 5.29 (dd, J = 17.2, 0.9 Hz, 1H), 5.19 (dd, J = 10.9, 0.9 Hz, 1H), 2.56 – 2.40 (m, 2H), 2.17 – 2.00 (m, 2H), 1.84 – 1.74 (m, 1H), 1.72 – 1.60 (m, 2H), 0.93 (dd, J = 9.6, 6.6 Hz, 6H).

^{13}C NMR spectrum ($CDCl_3$)

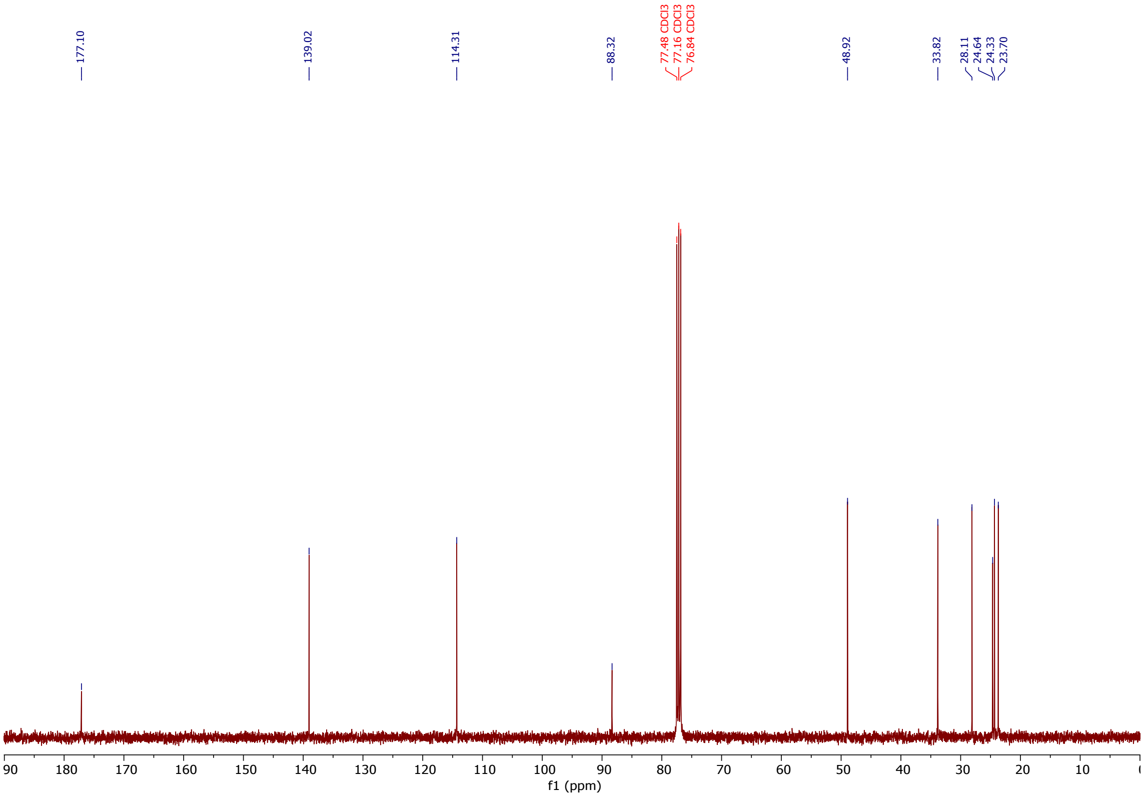

^{13}C NMR (101 MHz, $CDCl_3$) δ 177.10, 139.02, 114.31, 88.32, 48.92, 33.82, 28.11, 24.64, 24.33, 23.70.

IR spectrum (neat)

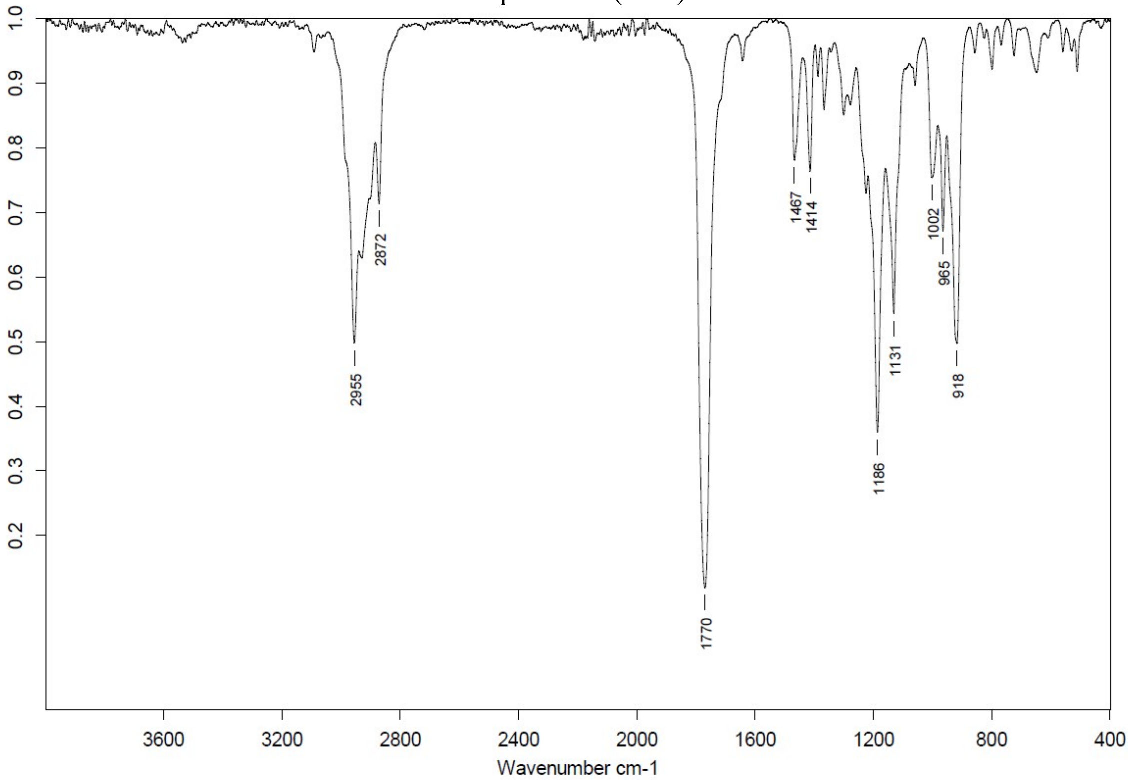

HRMS (ESI$^+$, MeOH): *m/z* calcd. 191.1043 (M + Na)$^+$, found: 191.1036.

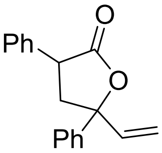

General procedure **B**: Slight yellow oil (39:61 *dr)*; Column conditions: Hexane : DCM = 1 : 1, R_f = 0.20

^{1}H NMR spectrum ($CDCl_3$)

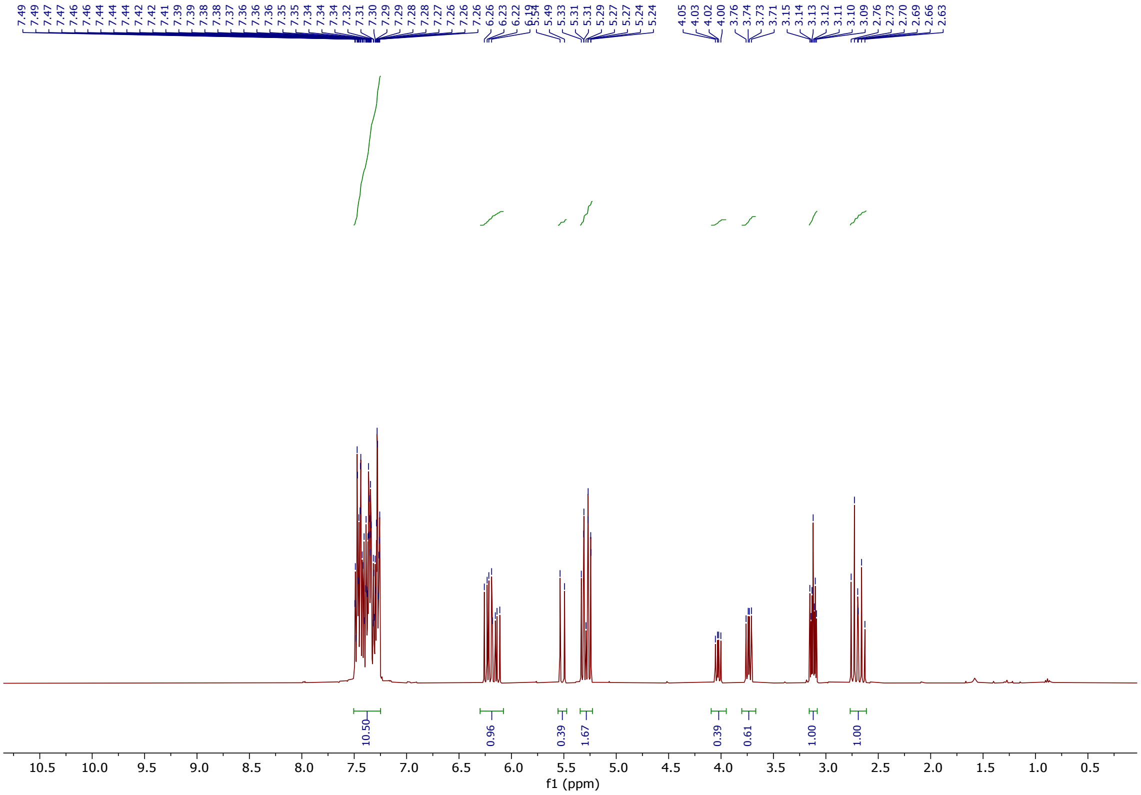

^{1}H NMR (400 MHz, $CDCl_3$) δ 7.50 – 7.25 (m, 10H), 6.29 – 6.07 (m, 1H), 5.51 (d, *J* = 17.1 Hz, 0.39H), 5.34 – 5.23 (m, 2H), 4.03 (dd, *J* = 12.6, 8.3 Hz, 0.39H), 3.74 (dd, *J* = 12.5, 8.3 Hz, 0.61H), 3.17 – 3.08 (m, 1H), 2.78 – 2.62 (m, 1H).

^{13}C NMR spectrum ($CDCl_3$)

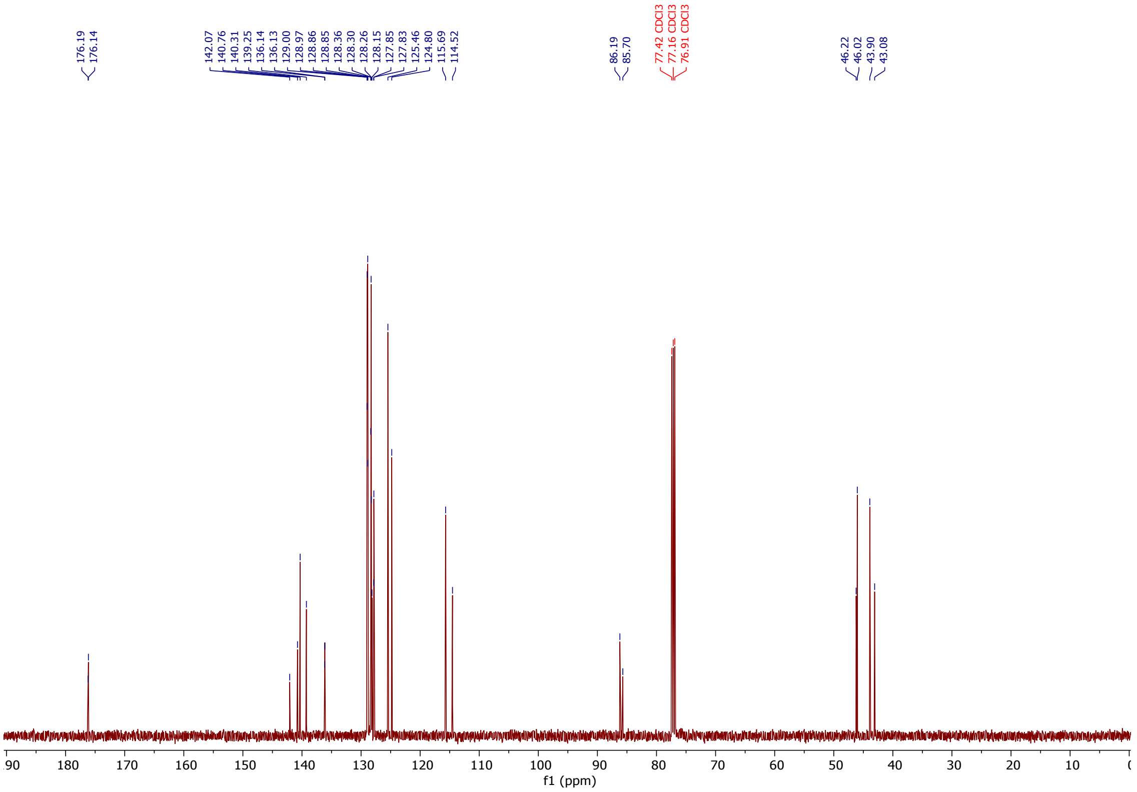

^{13}C NMR (126 MHz, $CDCl_3$) δ 176.19, 176.14, 142.07, 140.76, 140.31, 139.25, 136.14, 136.13, 129.00, 128.97, 128.86, 128.85, 128.36, 128.30, 128.26, 128.15, 127.85, 127.83, 125.46, 124.80, 115.69, 114.52, 86.19, 85.70, 46.22, 46.02, 43.90, 43.08.

IR spectrum (neat)

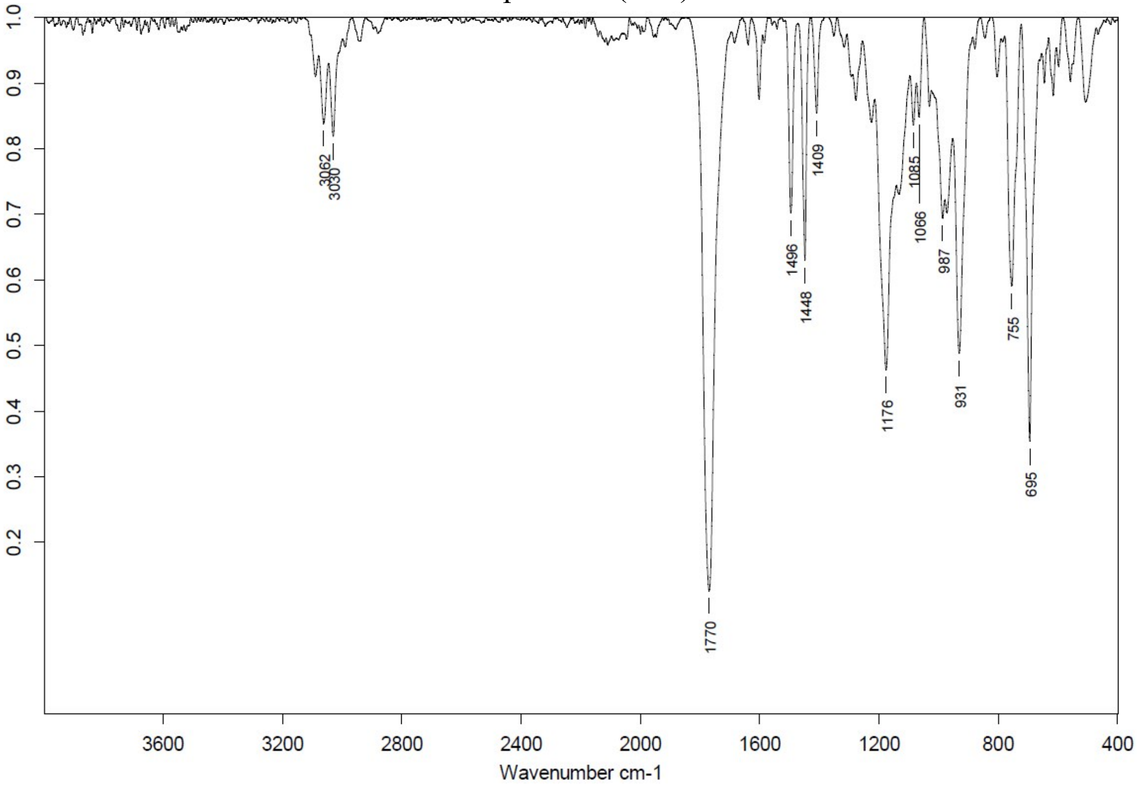

HRMS (ESI$^+$, MeOH): *m/z* calcd. 287.1043 $(M + Na)^+$, found: 287.1044.

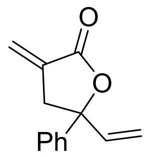

General procedure **A**: Yellow oil; Column conditions: Hexane : EA = 20 : 1, R_f = 0.15

^{1}H NMR spectrum ($CDCl_3$)

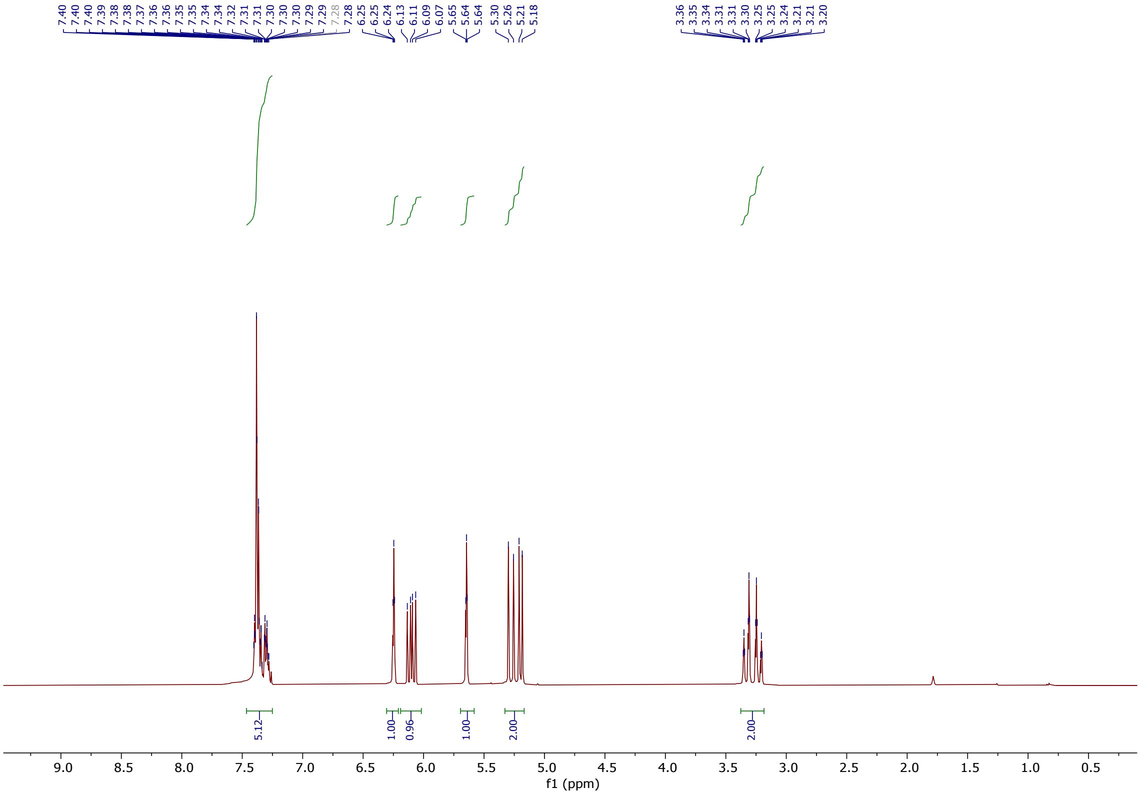

^{1}H NMR (400 MHz, $CDCl_3$) δ 7.46 – 7.25 (m, 5H), 6.25 (t, *J* = 2.7 Hz, 1H), 6.10 (dd, *J* = 17.1, 10.7 Hz, 1H), 5.64 (t, *J* = 2.4 Hz, 1H), 5.33 – 5.17 (m, 2H), 3.38 – 3.18 (m, 2H).

^{13}C NMR spectrum ($CDCl_3$)

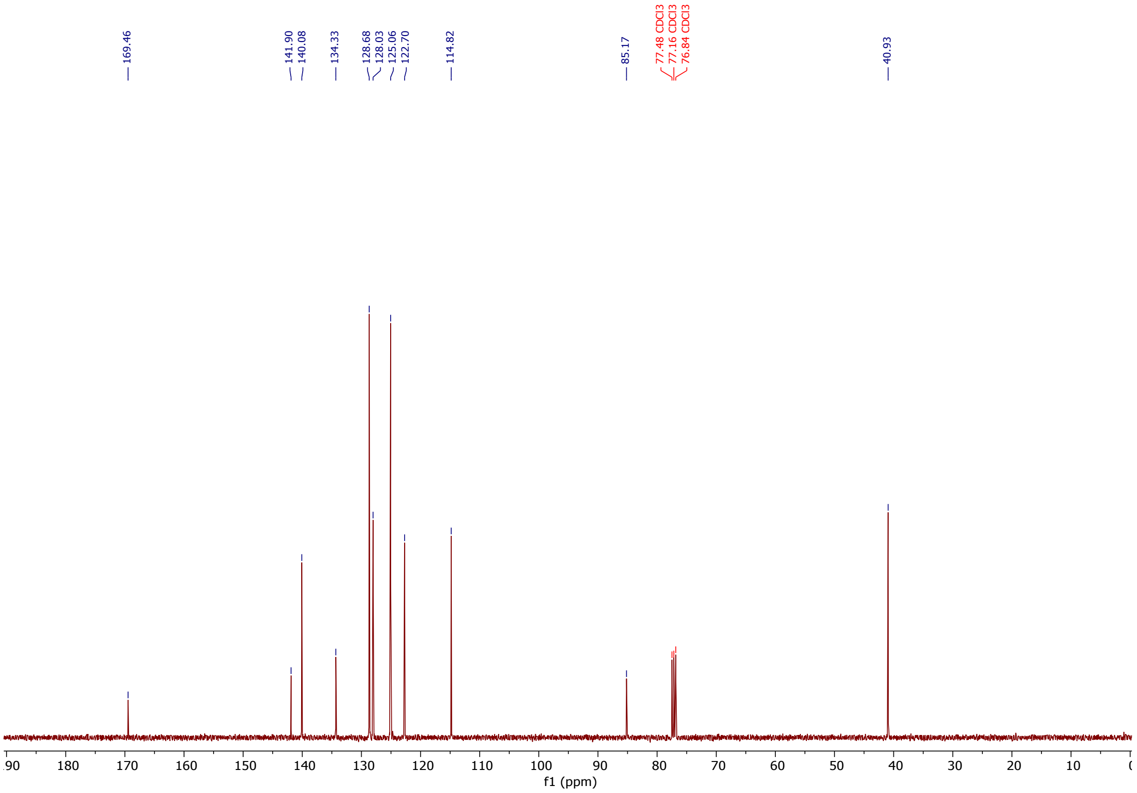

^{13}C NMR (101 MHz, $CDCl_3$) δ 169.46, 141.90, 140.08, 134.33, 128.68, 128.03, 125.06, 122.70, 114.82, 85.17, 40.93.

IR spectrum (neat)

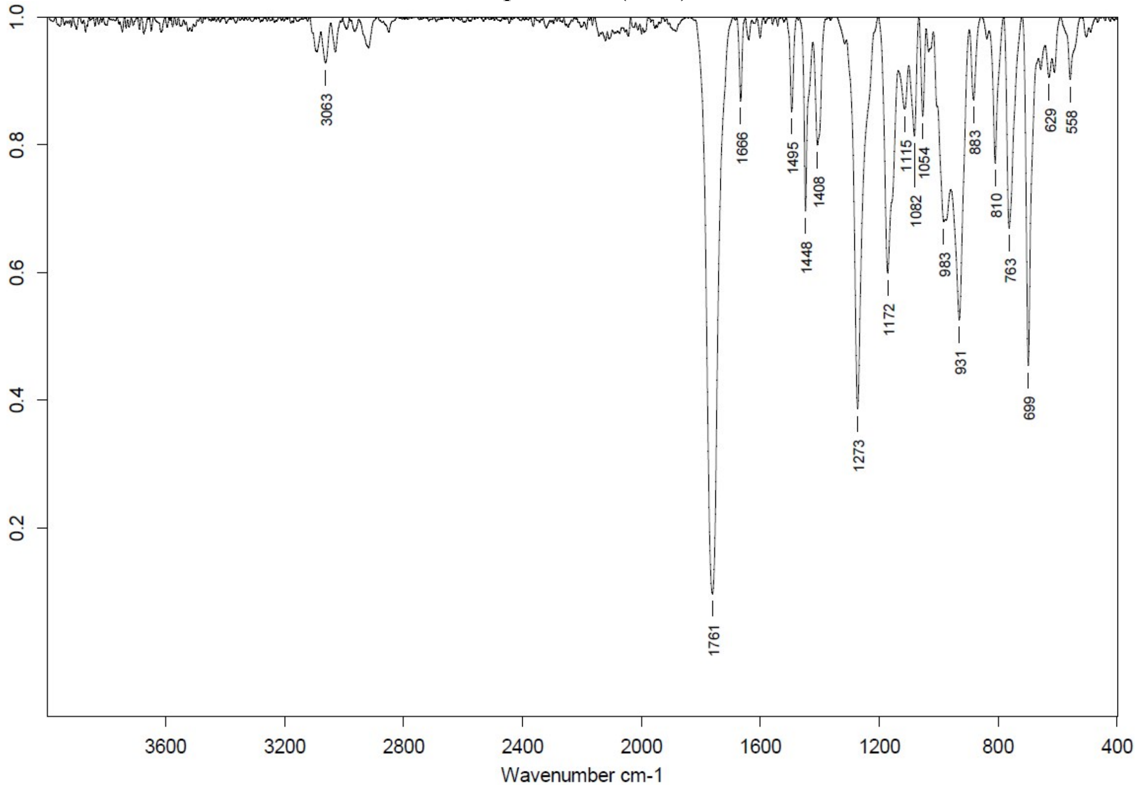

HRMS (ESI^+, MeOH): *m/z* calcd. 223.0730 $(M + Na)^+$, found: 223.0731.

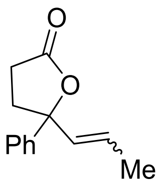

General procedure **B** (from 1-propenylmagnesium bromide): Yellow oil; Column conditions: Hexane : EA = 20 : 1, R_f = 0.13

^{1}H NMR spectrum ($CDCl_3$)

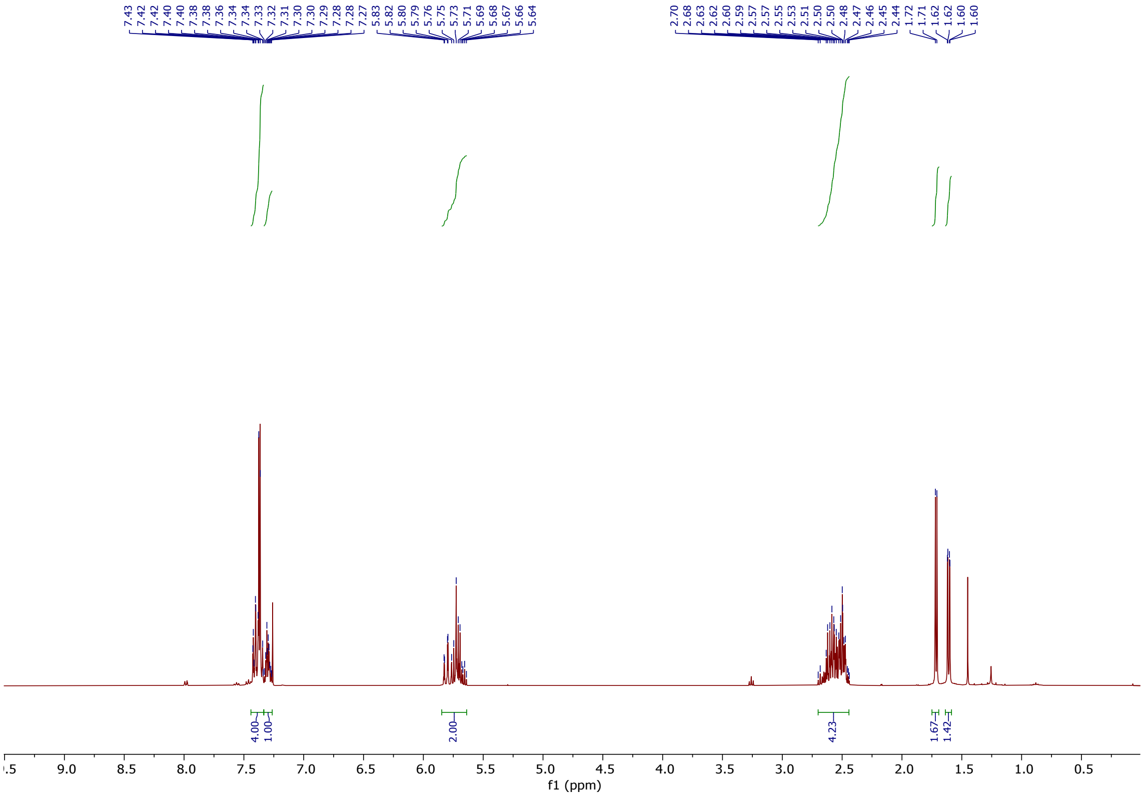

^{1}H NMR (400 MHz, $CDCl_3$) δ 7.44 – 7.33 (m, 4H), 7.33 – 7.26 (m, 1H), 5.85 – 5.64 (m, 2H), 2.70 – 2.44 (m, 4H), 1.72 – 1.60 (m, 3H). The lactone is a mixture of two stereoisomers with a ratio of *E/Z* = 54:46.

^{13}C NMR spectrum ($CDCl_3$)

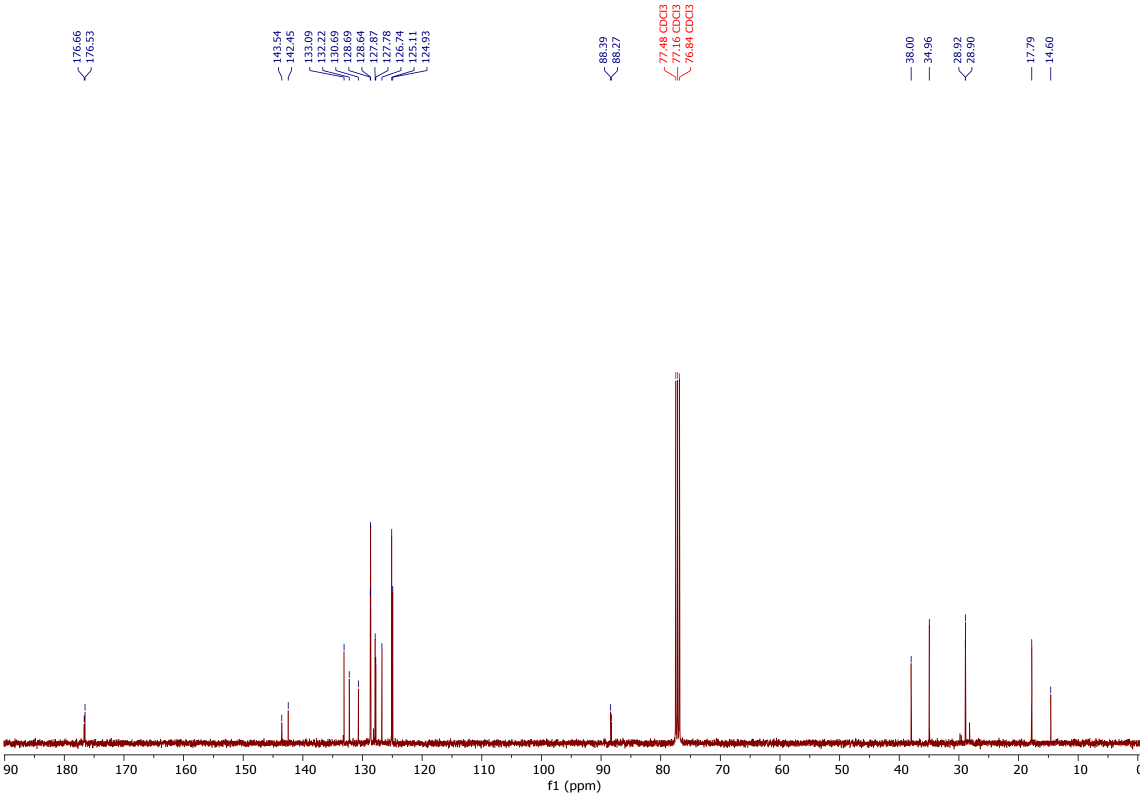

^{13}C NMR (101 MHz, $CDCl_3$) δ 176.66, 176.53, 143.54, 142.45, 133.09, 132.22, 130.69, 128.69, 128.64, 127.87, 127.78, 126.74, 125.11, 124.93, 88.39, 88.27, 38.00, 34.96, 28.92, 28.90, 17.79, 14.60.

IR spectrum (neat)

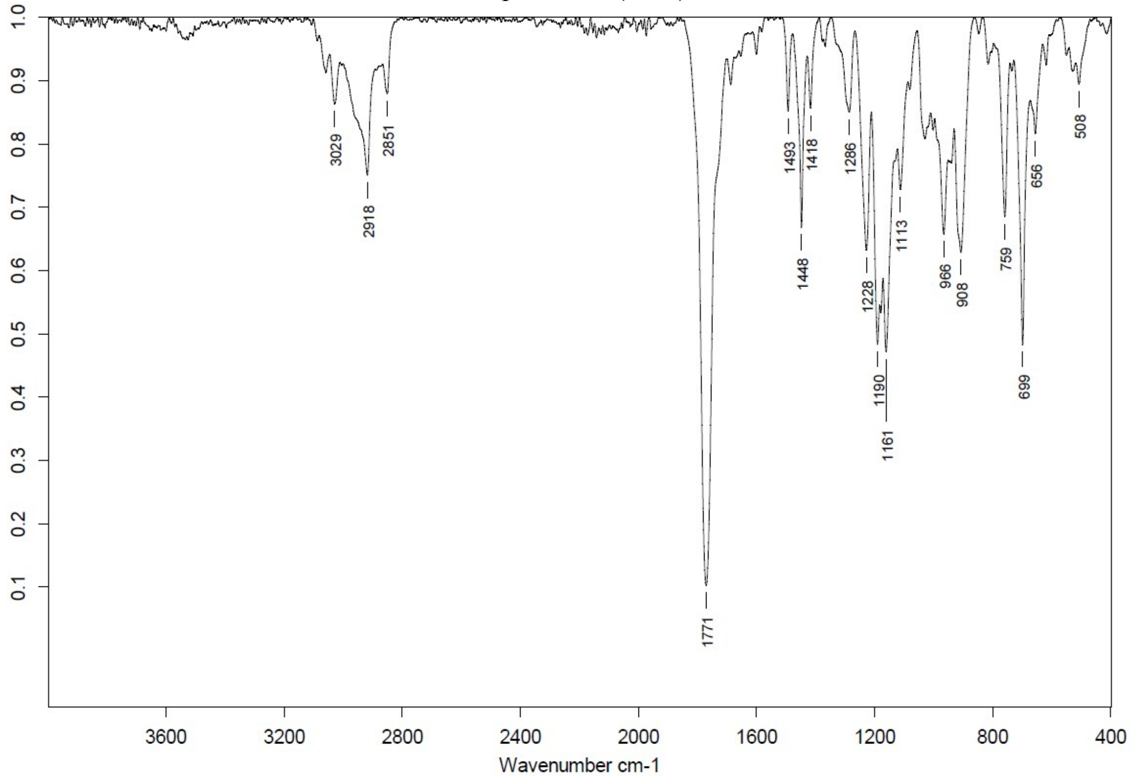

HRMS (ESI^+, MeOH): *m/z* calcd. 225.0886 $(M + Na)^+$, found: 225.0877.

S87. Characterization data for the amino acid intermediate *E*-1

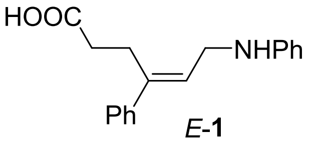

E-1

^{1}H NMR spectrum ($CDCl_3$)

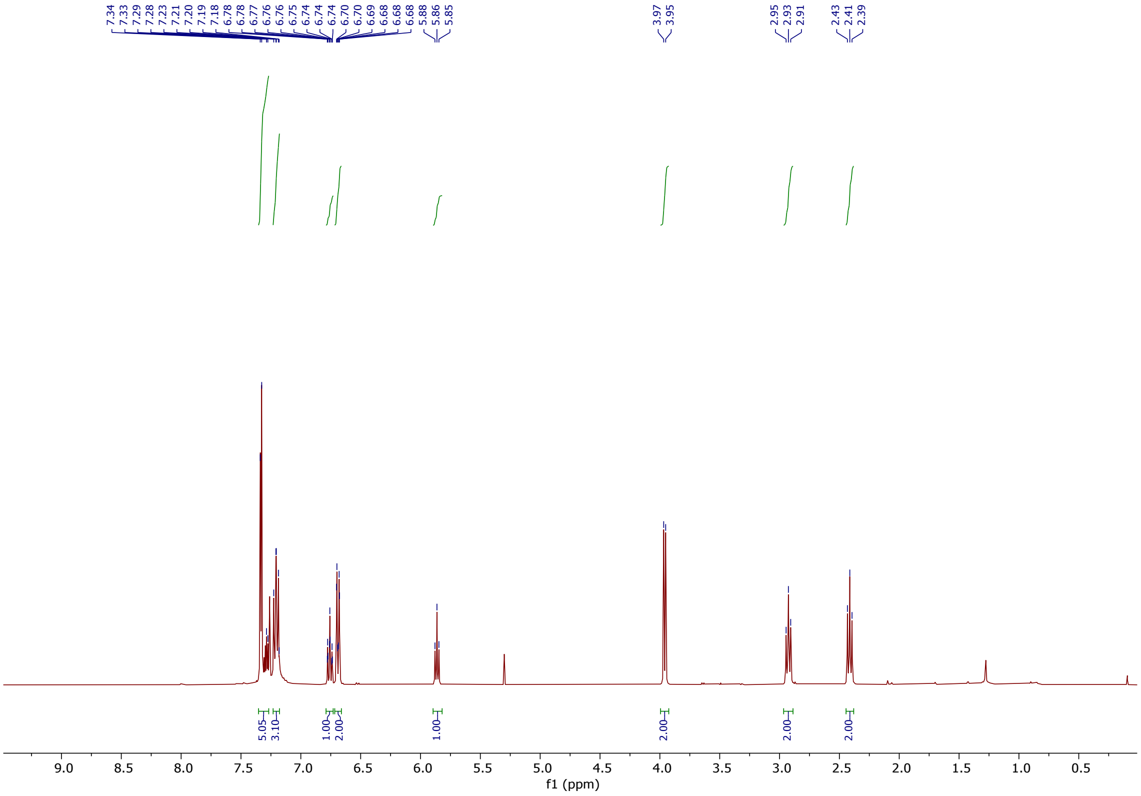

^{1}H NMR (400 MHz, $CDCl_3$) δ 7.35 – 7.27 (m, 5H), 7.23 – 7.17 (m, 3H), 6.76 (tt, J = 7.4, 1.1 Hz, 1H), 6.72 – 6.66 (m, 2H), 5.86 (t, J = 6.6 Hz, 1H), 3.96 (d, J = 6.7 Hz, 2H), 2.93 (t, J = 7.7 Hz, 2H), 2.44 – 2.38 (m, 2H).

^{13}C NMR spectrum ($CDCl_3$)

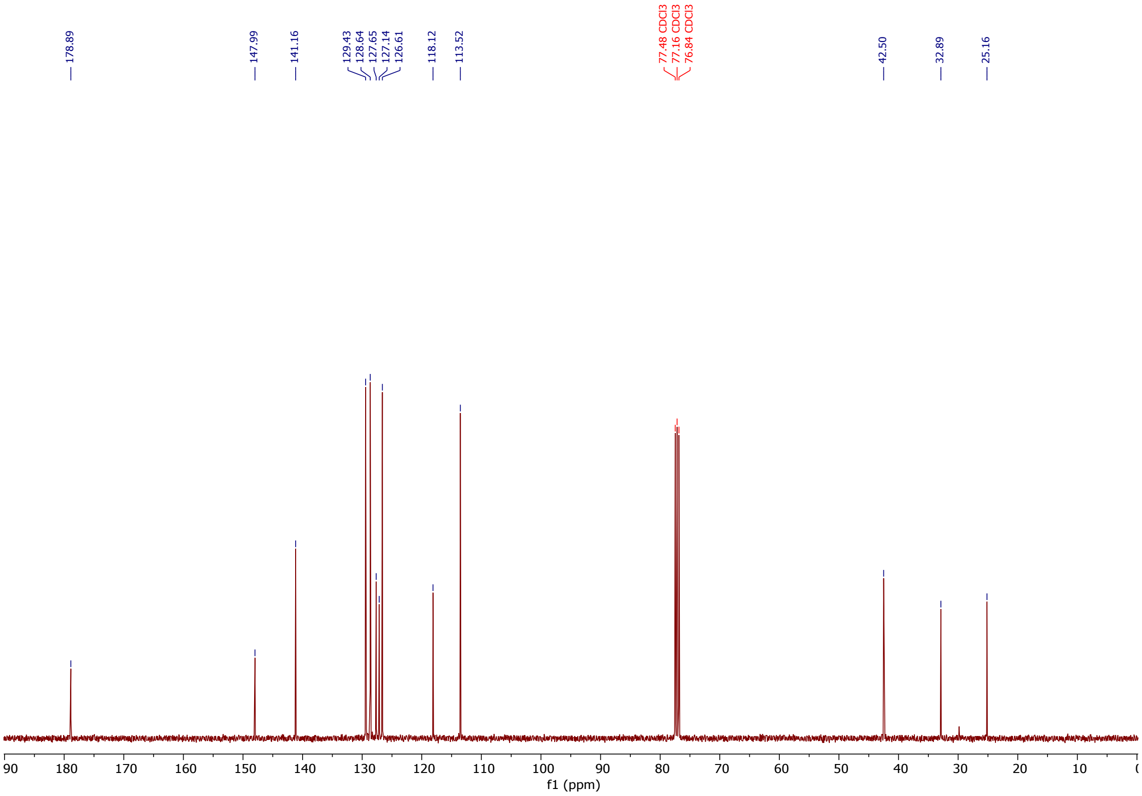

^{13}C NMR (101 MHz, $CDCl_3$) δ 178.89, 147.99, 141.16, 129.43, 128.64, 127.65, 127.14, 126.61, 118.12, 113.52, 42.50, 32.89, 25.16.

IR spectrum (neat)

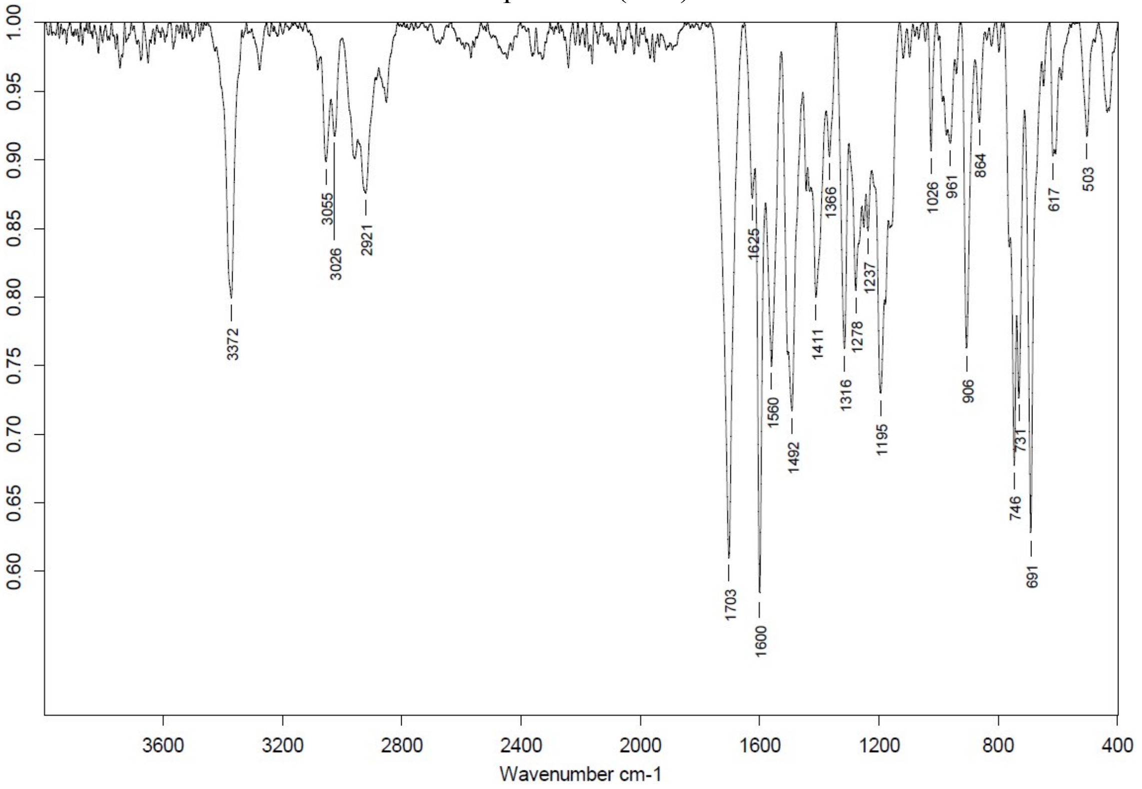

HRMS (ESI^-, MeOH): *m/z* calcd. 280.1343 $(M - H)^-$, found: 280.1349.

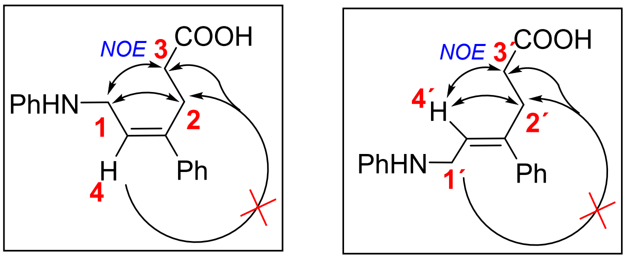

(*E*-**1**) (*Z*-**1**)

2D ^{1}H-^{1}H NOESY NMR spectrum ($CDCl_3$) for the mixture of *Z*-**1** and *E*-**1**

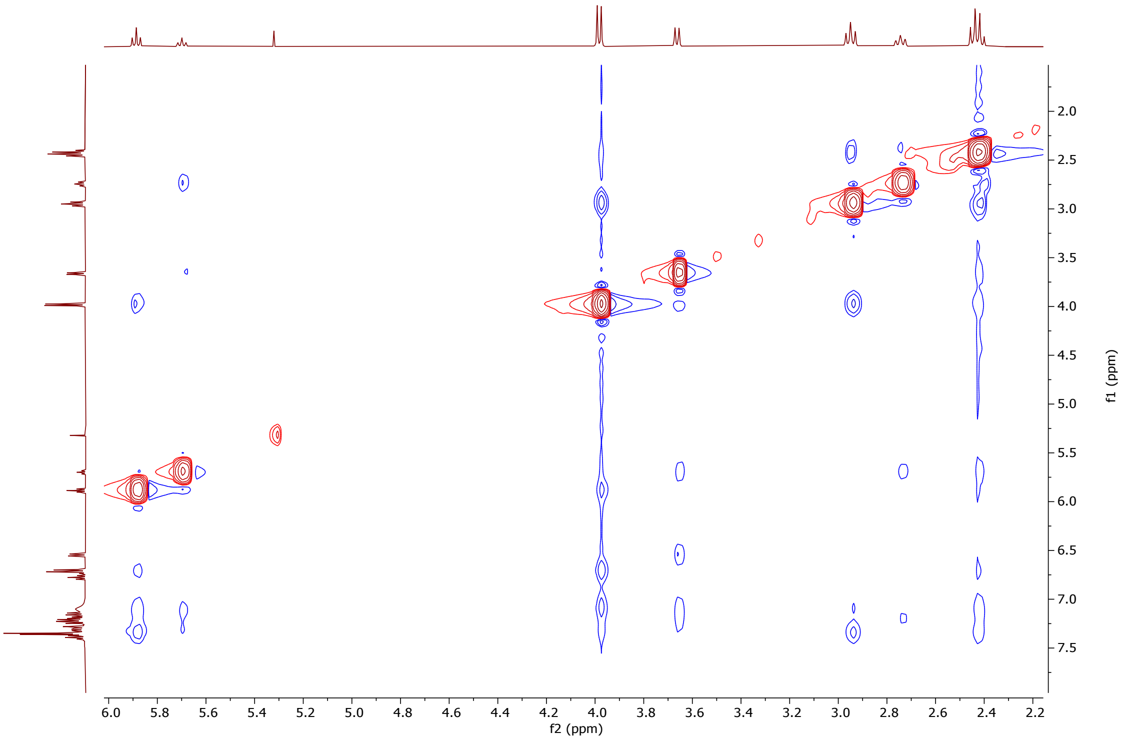

S90. IR, NMR spectra and HRMS data for caprolactam products

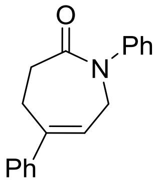

Compound 3

Scale: 0.2 mmol; *E/Z* = 98:2, isolated 43.2 mg (82% yield), slight yellow solid, Hexane : EA = 2 : 1, R_f = 0.25

^{1}H NMR spectrum ($CDCl_3$)

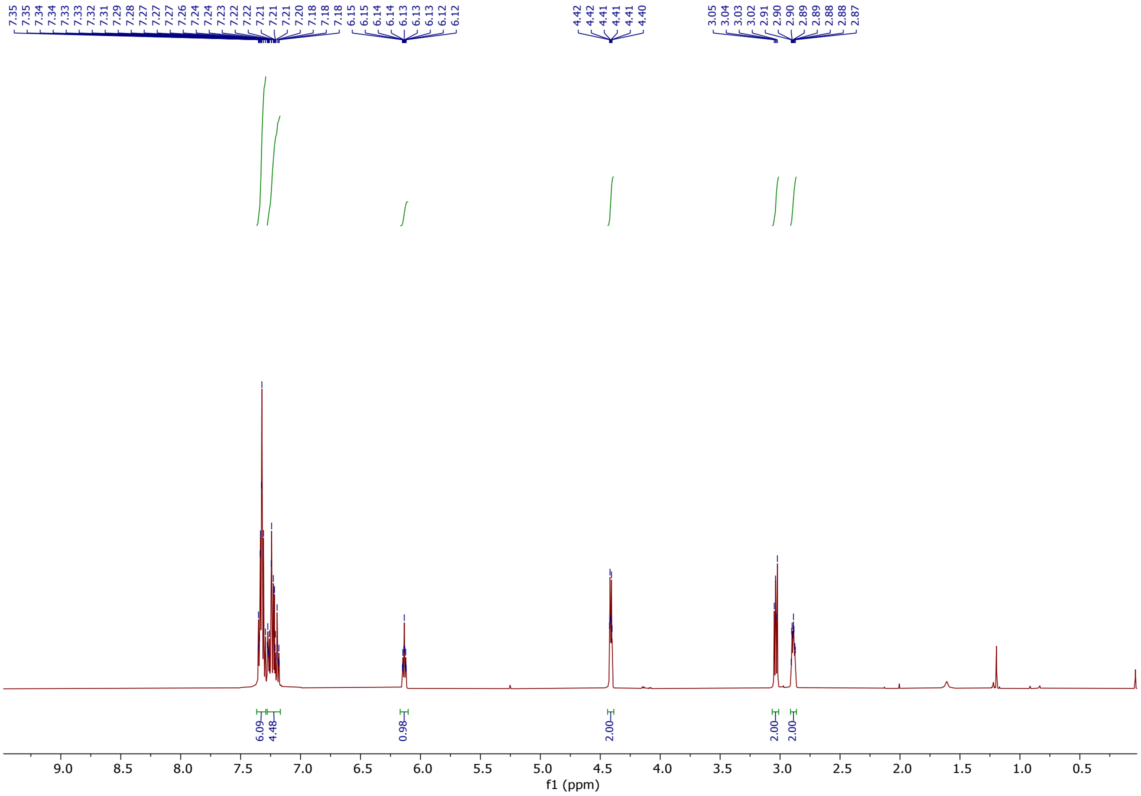

^{1}H NMR (500 MHz, $CDCl_3$) δ 7.37 – 7.29 (m, 6H), 7.28 – 7.17 (m, 4H), 6.13 (tt, *J* = 6.1, 1.9 Hz, 1H), 4.41 (dt, *J* = 6.1, 2.2 Hz, 2H), 3.07 – 3.01 (m, 2H), 2.91– 2.85 (m, 2H).

^{13}C NMR spectrum ($CDCl_3$)

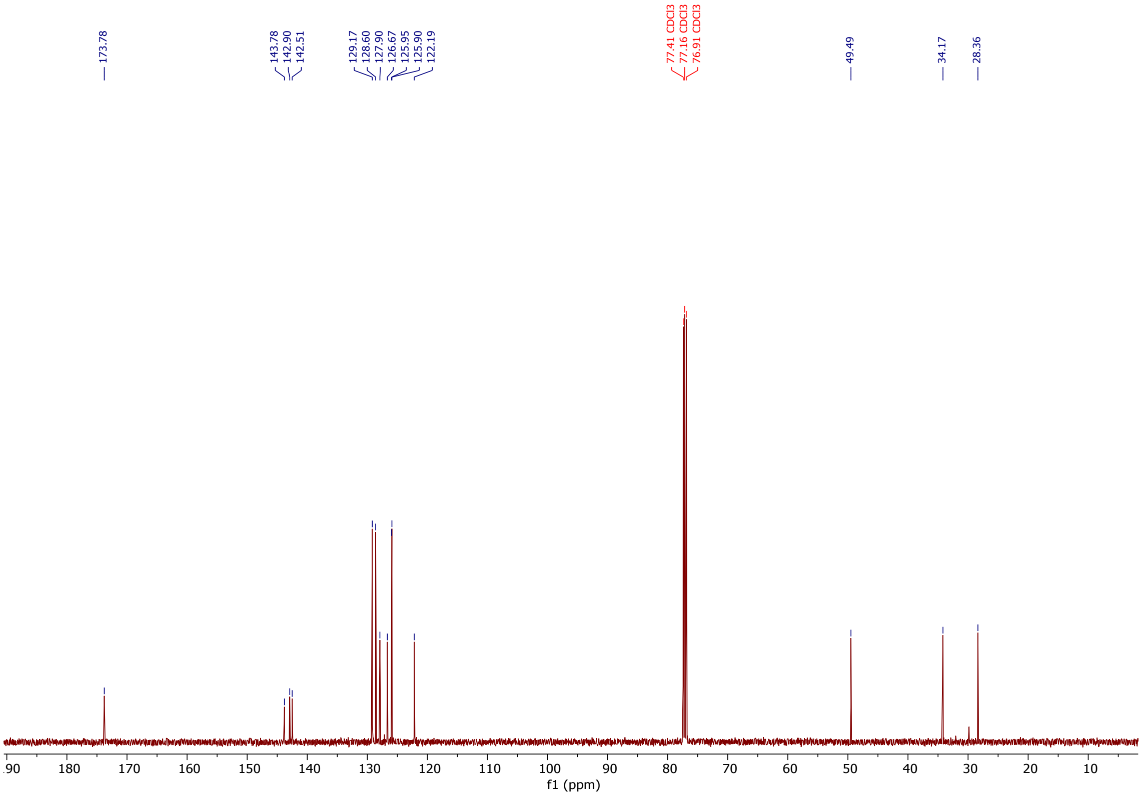

^{13}C NMR (126 MHz, $CDCl_3$) δ 173.78, 143.78, 142.90, 142.51, 129.17, 128.60, 127.90, 126.67, 125.95, 125.90, 122.19, 49.49, 34.17, 28.36.

IR spectrum (neat)

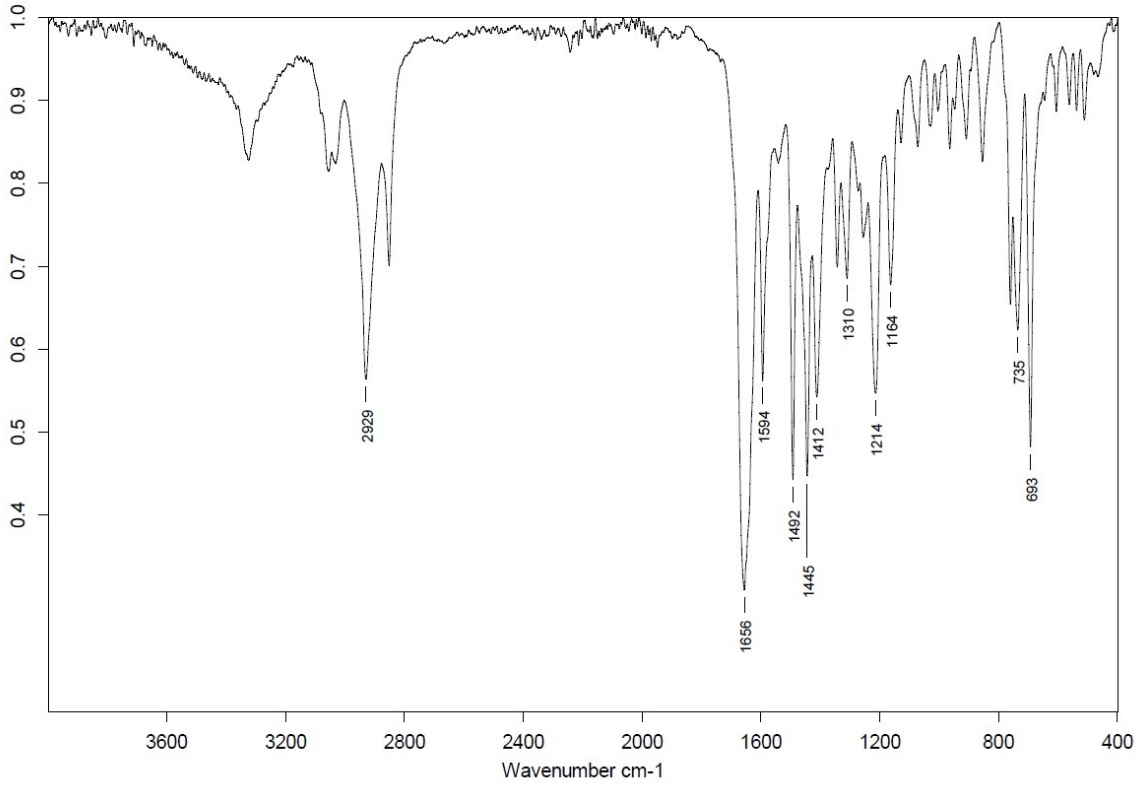

HRMS (ESI$^+$, MeOH): *m/z* calcd. 264.1383 $(M + H)^+$, found: 264.1379.

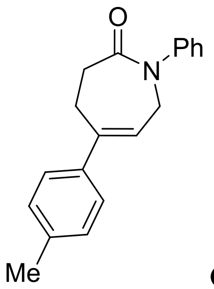

Compound 4

Scale: 0.2 mmol; E/Z = 96:4, isolated 44.9 mg (81% yield), yellow solid, Hexane : EA = 2 : 1, R_f = 0.25

^{1}H NMR spectrum ($CDCl_3$)

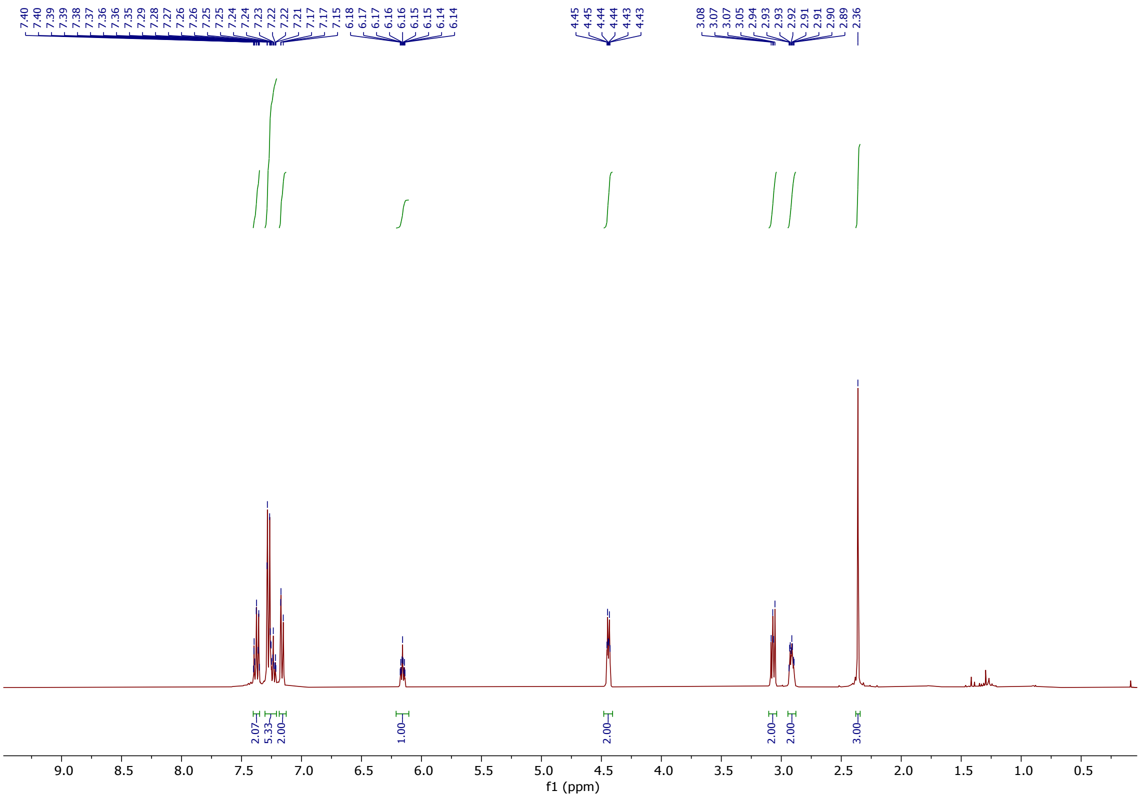

^{1}H NMR (400 MHz, $CDCl_3$) δ 7.40 – 7.35 (m, 2H), 7.30 – 7.21 (m, 5H), 7.19 – 7.13 (m, 2H), 6.16 (tt, J = 6.1, 1.8 Hz, 1H), 4.44 (dt, J = 6.1, 2.1 Hz, 2H), 3.10 – 3.03 (m, 2H), 2.94 – 2.87 (m, 2H), 2.36 (s, 3H).

^{13}C NMR spectrum ($CDCl_3$)

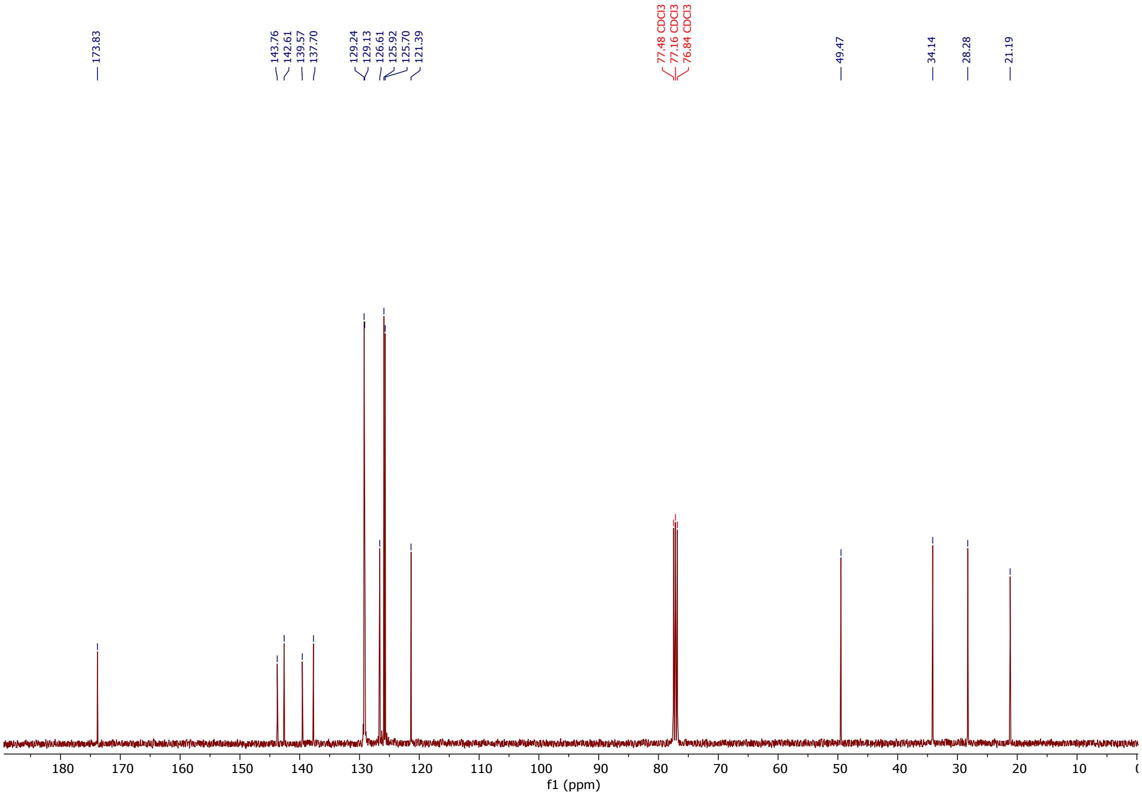

^{13}C NMR (101 MHz, $CDCl_3$) δ 173.83, 143.76, 142.61, 139.57, 137.70, 129.24, 129.13, 126.61, 125.92, 125.70, 121.39, 49.47, 34.14, 28.28, 21.19.

IR spectrum (neat)

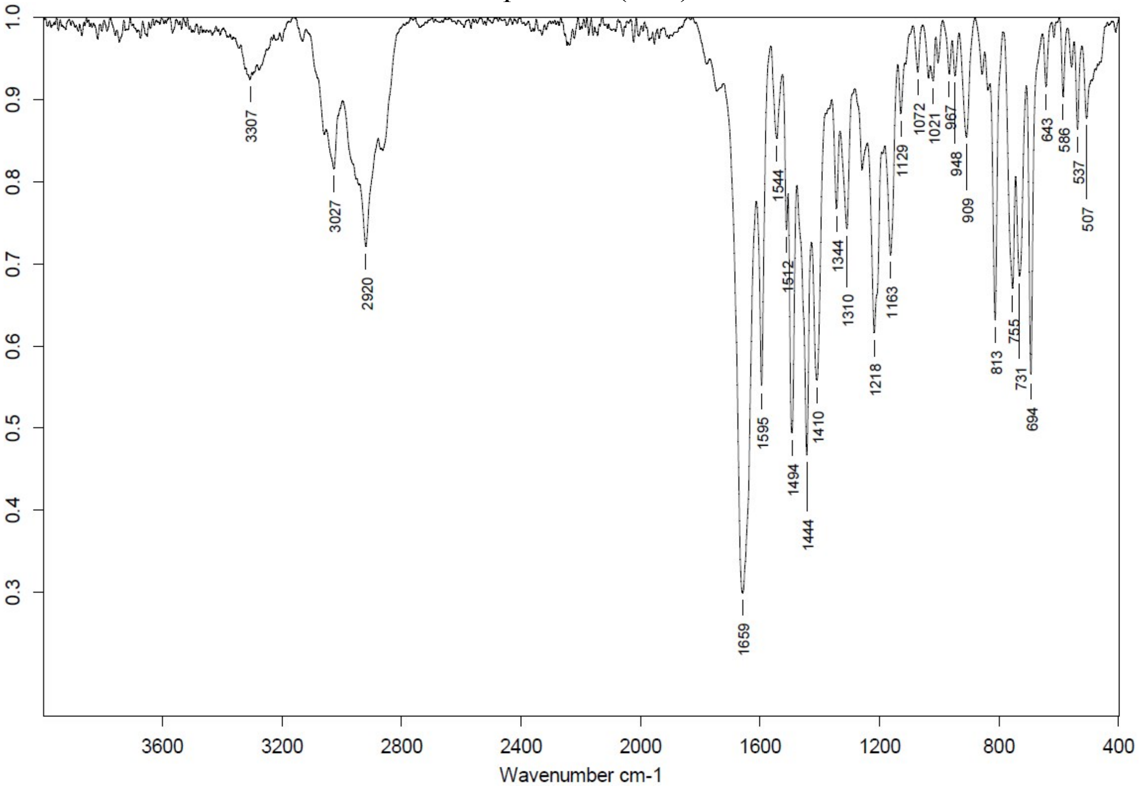

HRMS (ESI^+, MeOH): *m/z* calcd. 300.1359 $(M + Na)^+$, found: 300.1360.

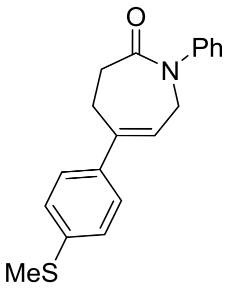

Compound 5

Scale: 0.2 mmol; E/Z = 90:10, isolated 50.7 mg (82% yield), yellow solid, Hexane : EA = 2 : 1, R_f = 0.25

^{1}H NMR spectrum ($CDCl_3$)

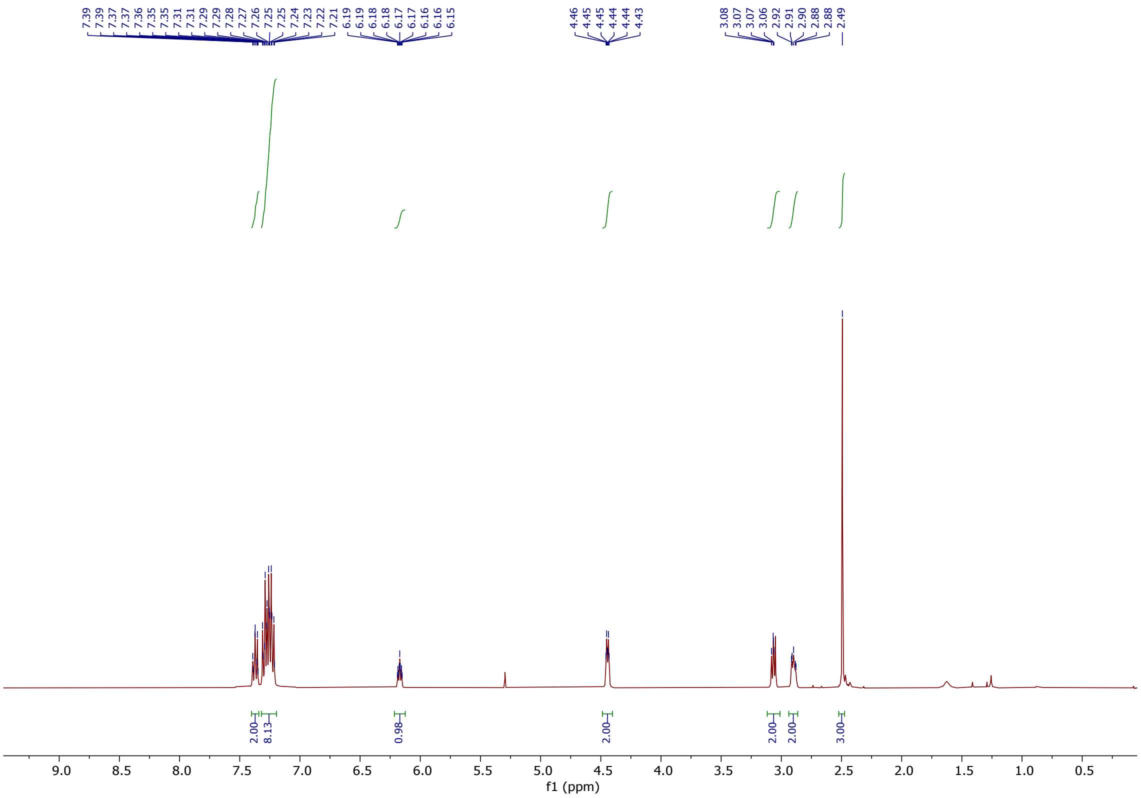

^{1}H NMR (400 MHz, $CDCl_3$) δ 7.40 – 7.34 (m, 2H), 7.32 – 7.19 (m, 8H), 6.17 (tt, J = 6.1, 1.9 Hz, 1H), 4.44 (dt, J = 6.1, 2.1 Hz, 2H), 3.12 – 3.01 (m, 2H), 2.94 – 2.86 (m, 2H), 2.49 (s, 3H).

^{13}C NMR spectrum ($CDCl_3$)

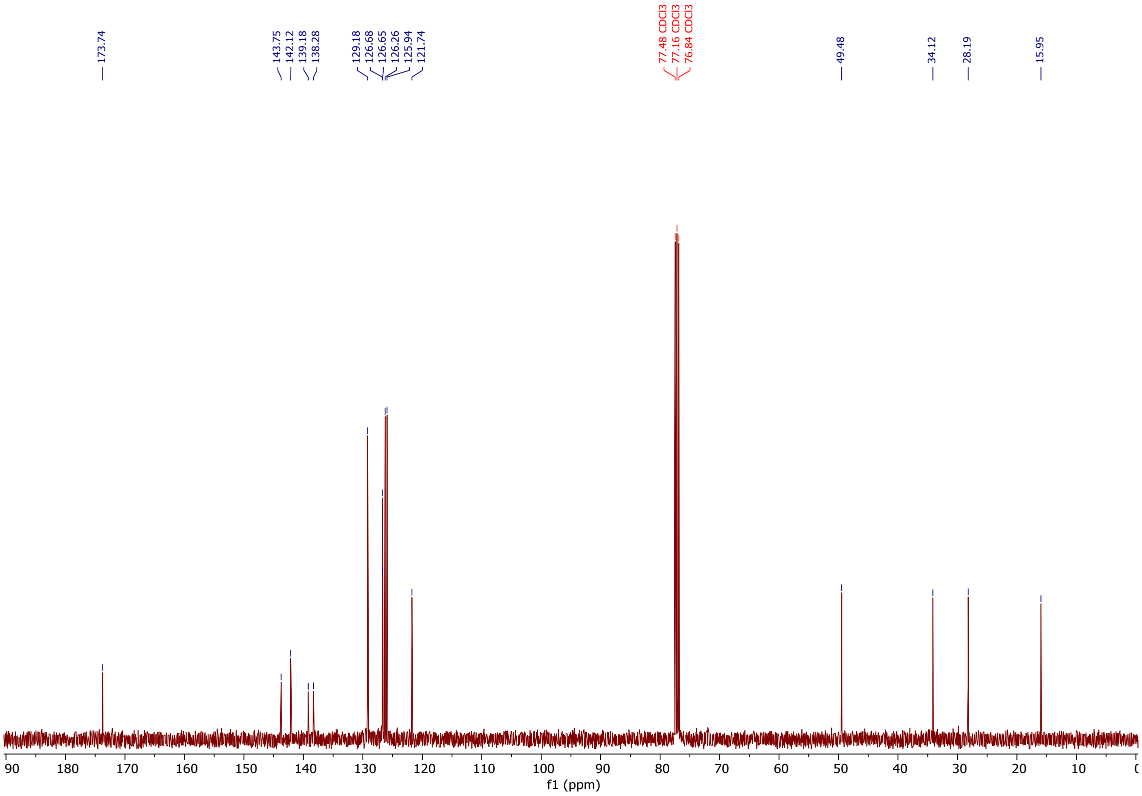

^{13}C NMR (101 MHz, $CDCl_3$) δ 173.74, 143.75, 142.12, 139.18, 138.28, 129.18, 126.68, 126.65, 126.26, 125.94, 121.74, 49.48, 34.12, 28.19, 15.95.

IR spectrum (neat)

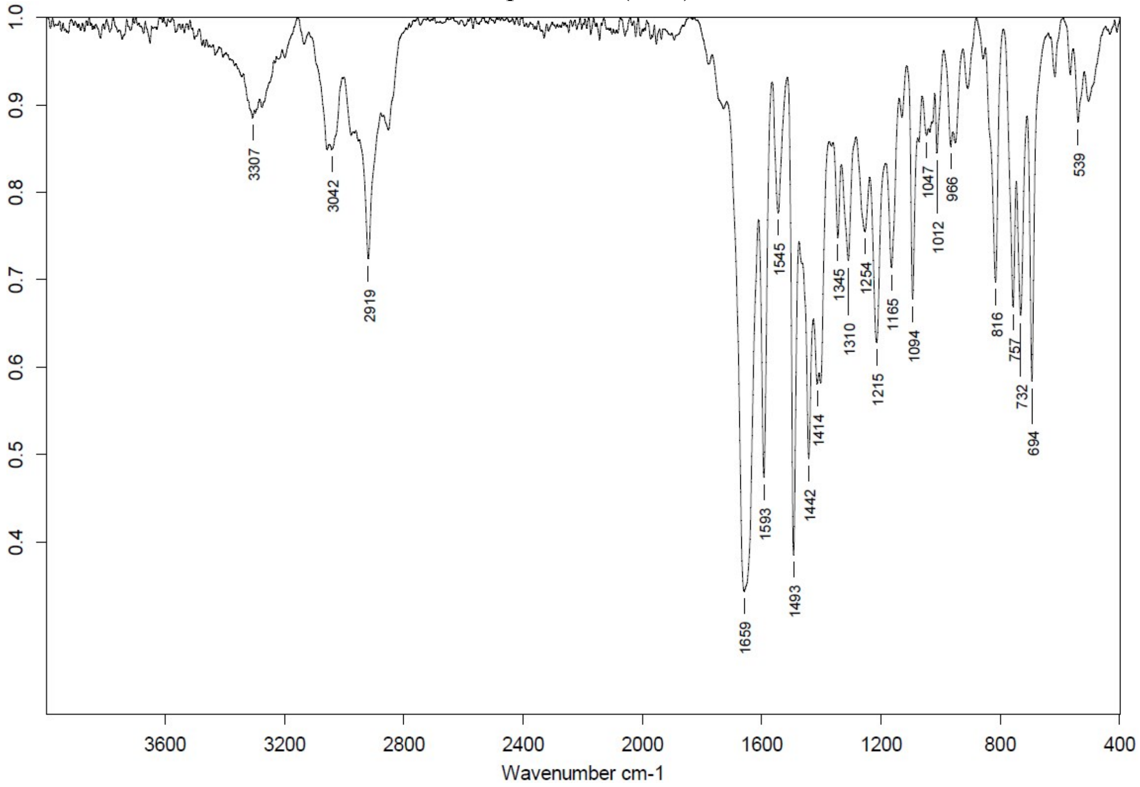

HRMS (ESI$^+$, MeOH): *m/z* calcd. 310.1260 $(M + H)^+$, found: 310.1267.

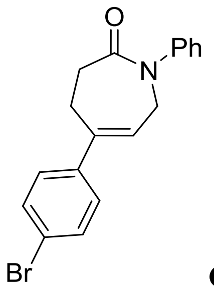

Compound 6

Scale: 0.2 mmol; *E/Z* = 97:3, isolated 57.5 mg (84% yield), yellow solid, Hexane : EA = 2 : 1, R_f = 0.25

^{1}H NMR spectrum ($CDCl_3$)

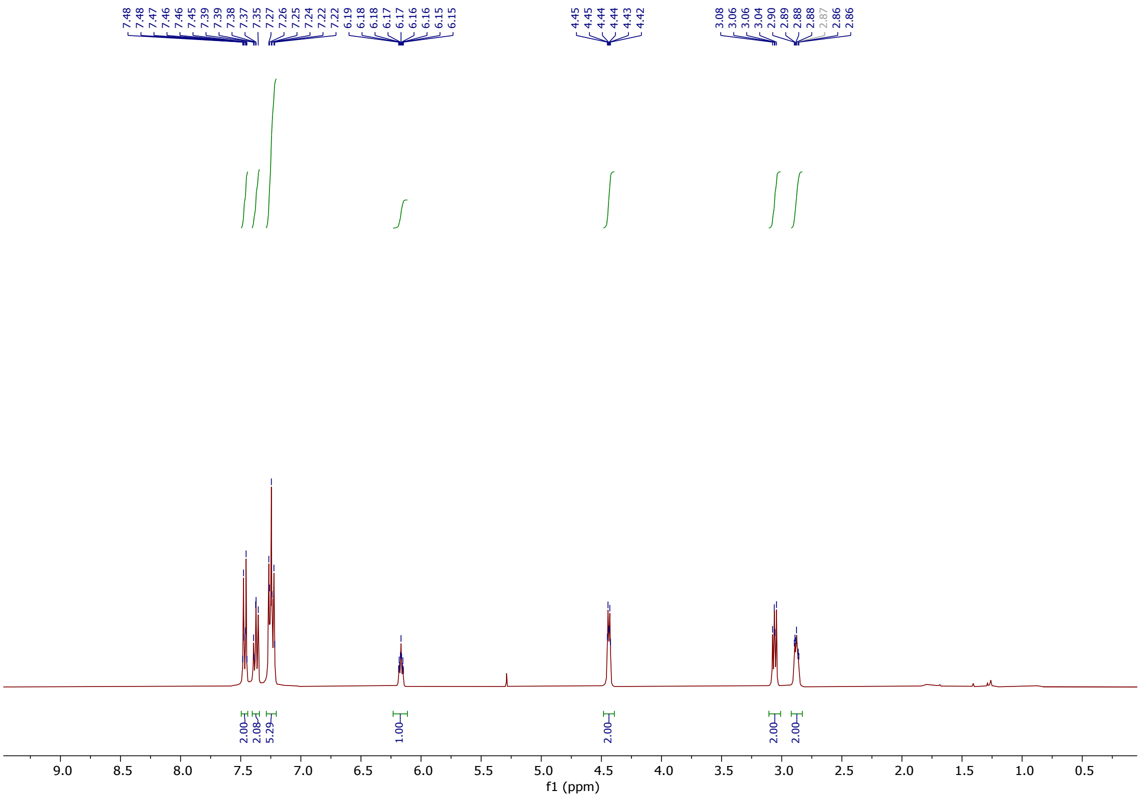

^{1}H NMR (400 MHz, $CDCl_3$) δ 7.50 – 7.44 (m, 2H), 7.40 – 7.34 (m, 2H), 7.28 – 7.19 (m, 5H), 6.17 (tt, *J* = 6.1, 1.9 Hz, 1H), 4.44 (dt, *J* = 6.1, 2.2 Hz, 2H), 3.08 – 3.04 (m, 2H), 2.90 – 2.86 (m, 2H).

^{13}C NMR spectrum ($CDCl_3$)

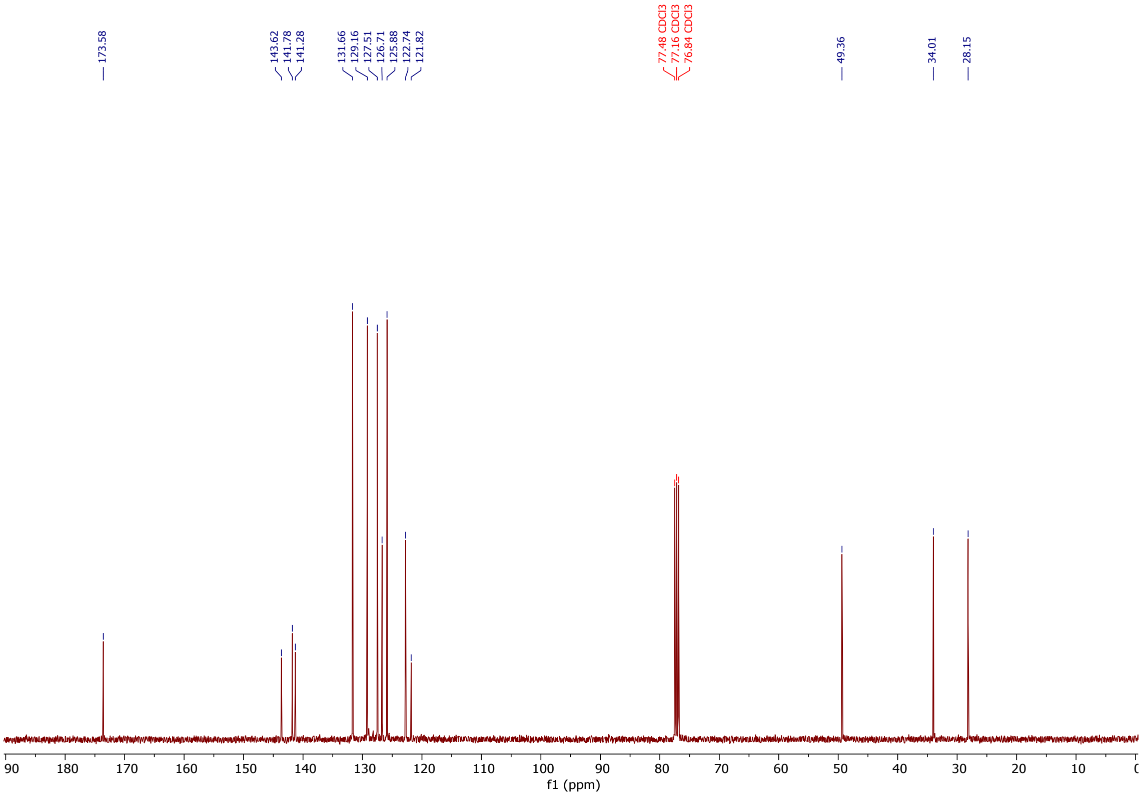

^{13}C NMR (101 MHz, $CDCl_3$) δ 173.58, 143.62, 141.78, 141.28, 131.66, 129.16, 127.51, 126.71, 125.88, 122.74, 121.82, 49.36, 34.01, 28.15.

IR spectrum (neat)

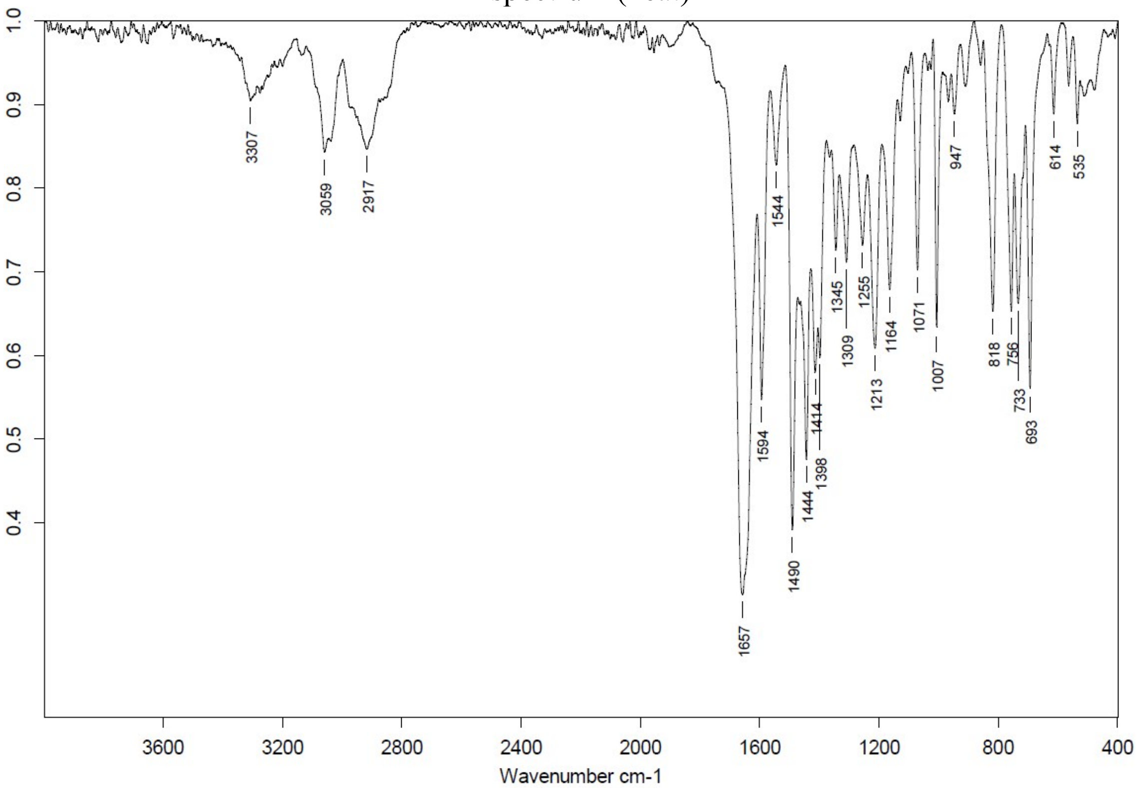

HRMS (ESI$^+$, MeOH): *m/z* calcd. 342.0488 $(M + H)^+$, found: 342.0499.

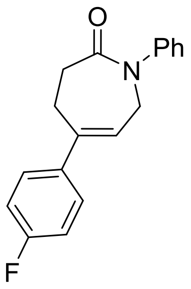

Compound 7

Scale: 0.2 mmol; *E/Z* = 97:3, isolated 50.6 mg (90% yield), yellow solid, Hexane : EA = 2 : 1, R_f = 0.25

^{1}H NMR spectrum (CDCl$_3$)

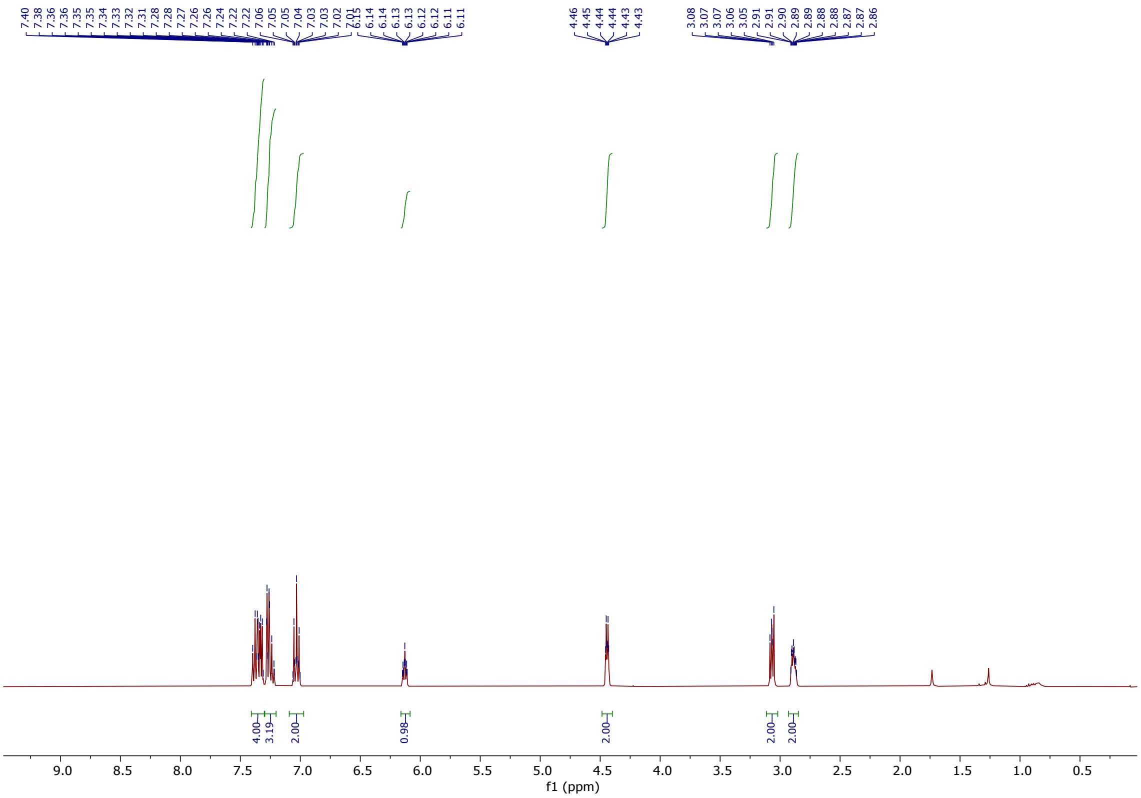

^{1}H NMR (400 MHz, CDCl$_3$) δ 7.41 – 7.30 (m, 4H), 7.30 – 7.20 (m, 3H), 7.09 – 6.97 (m, 2H), 6.13 (tt, *J* = 6.1, 1.8 Hz, 1H), 4.44 (dt, *J* = 6.1, 2.1 Hz, 2H), 3.11 – 3.02 (m, 2H), 2.92 – 2.83 (m, 2H).

^{13}C NMR spectrum ($CDCl_3$)

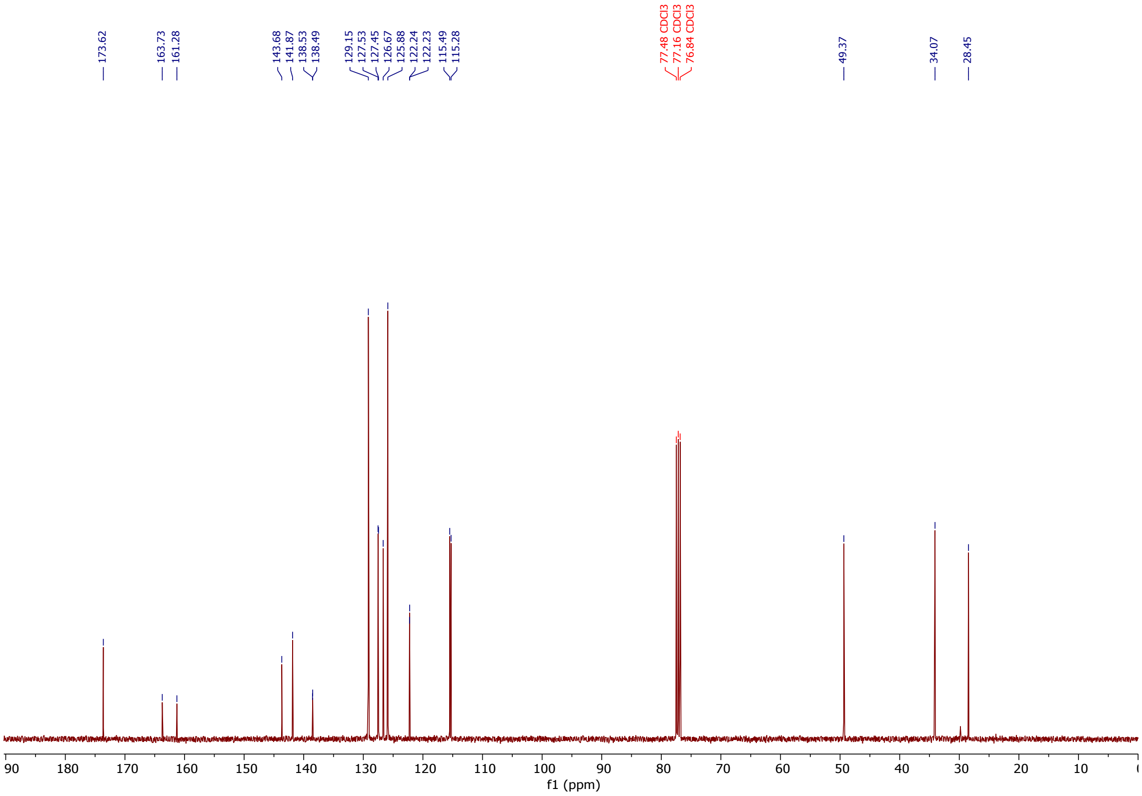

^{13}C NMR (101 MHz, $CDCl_3$) δ 173.62, 162.51 (d, J = 246.6 Hz), 143.68, 141.87, 138.51 (d, J = 3.3 Hz), 129.15, 127.49 (d, J = 8.1 Hz), 126.67, 125.88, 122.23 (d, J = 1.5 Hz), 115.39 (d, J = 21.6 Hz), 49.37, 34.07, 28.45.

^{19}F NMR spectrum ($CDCl_3$)

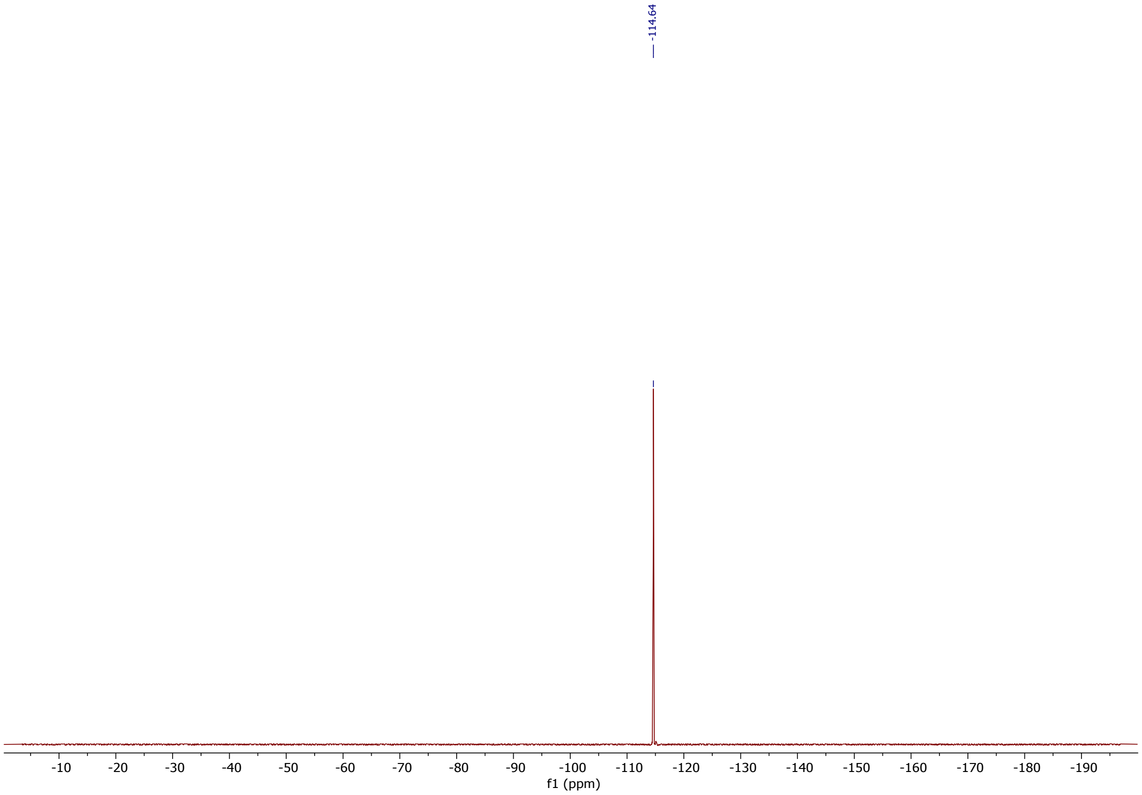

^{19}F NMR (376 MHz, $CDCl_3$) δ -114.64.

IR spectrum (neat)

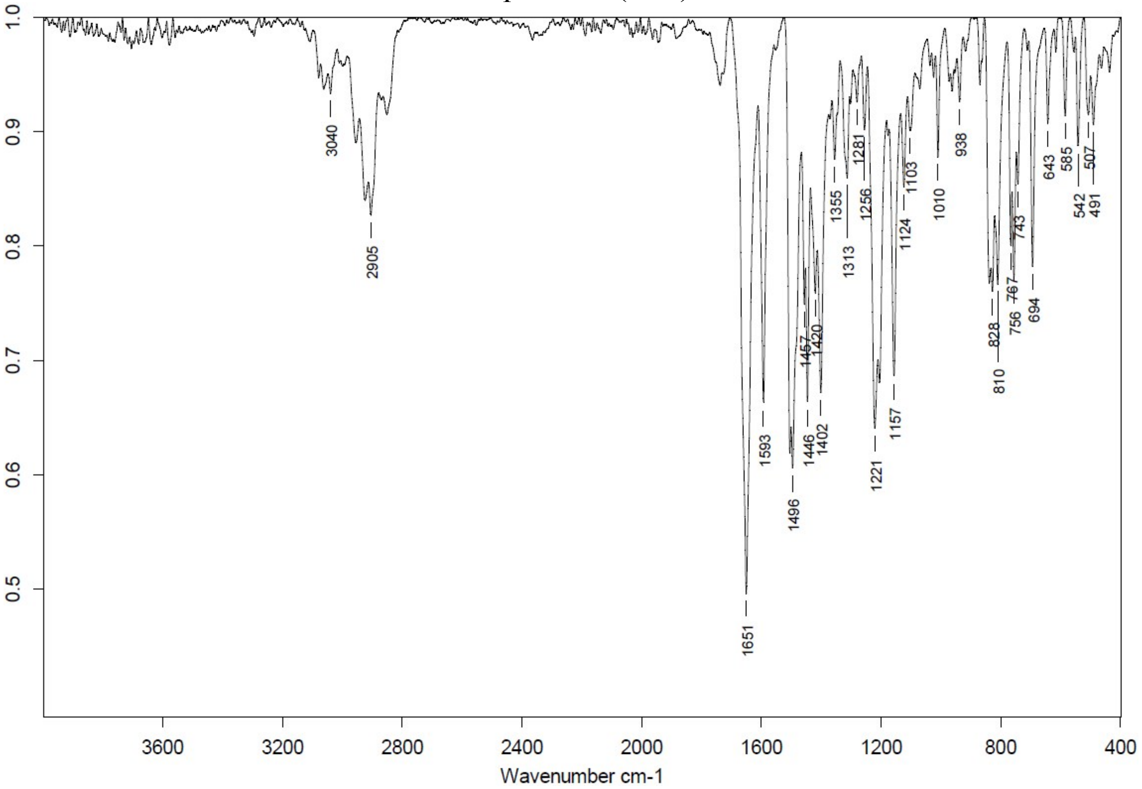

HRMS (ESI$^+$, MeOH): *m/z* calcd. 304.1108 (M + Na)$^+$, found: 304.1105.

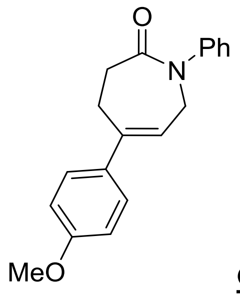

Compound 8

Scale: 0.2 mmol; *E/Z* = 93:7, isolated 41.1 mg (70% yield), yellow solid, Hexane : EA = 2 : 1, R_f = 0.25

^{1}H NMR spectrum ($CDCl_3$)

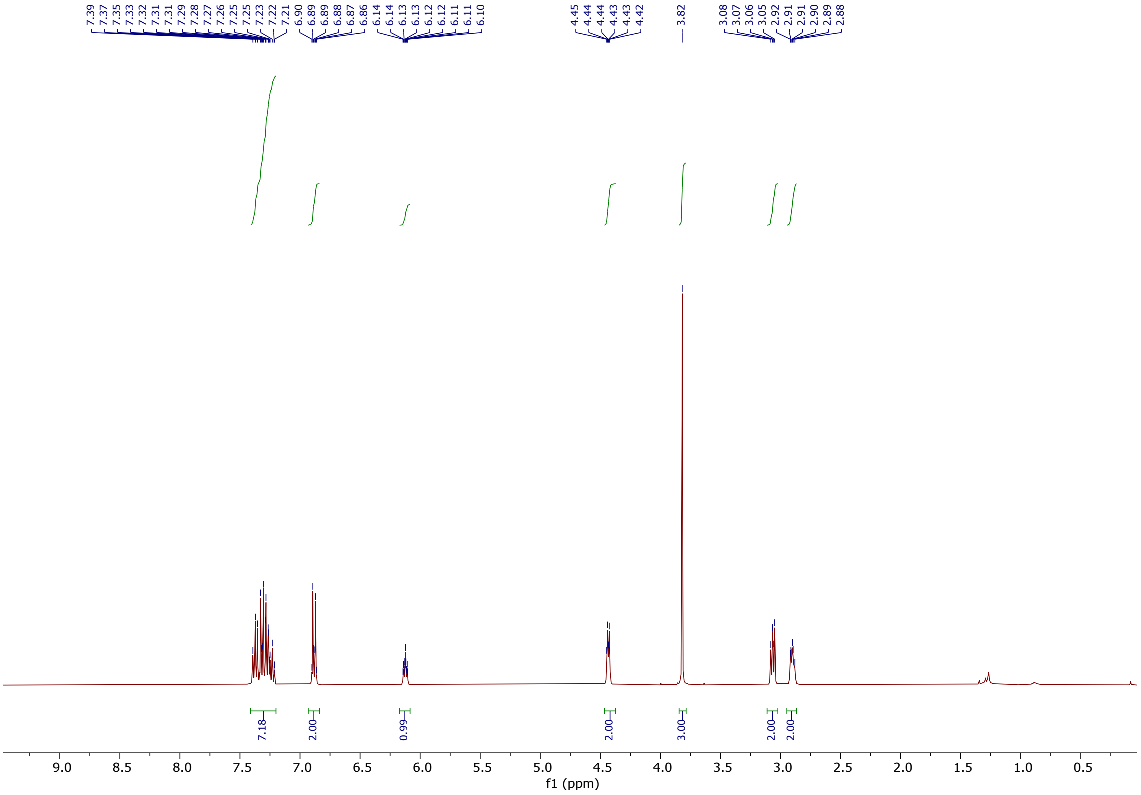

^{1}H NMR (400 MHz, $CDCl_3$) δ 7.41 – 7.20 (m, 7H), 6.93 – 6.84 (m, 2H), 6.12 (tt, *J* = 6.1, 1.8 Hz, 1H), 4.43 (dt, *J* = 6.1, 2.1 Hz, 2H), 3.82 (s, 3H), 3.08 – 3.04 (m, 2H), 2.95 – 2.87 (m, 2H).

^{13}C NMR spectrum ($CDCl_3$)

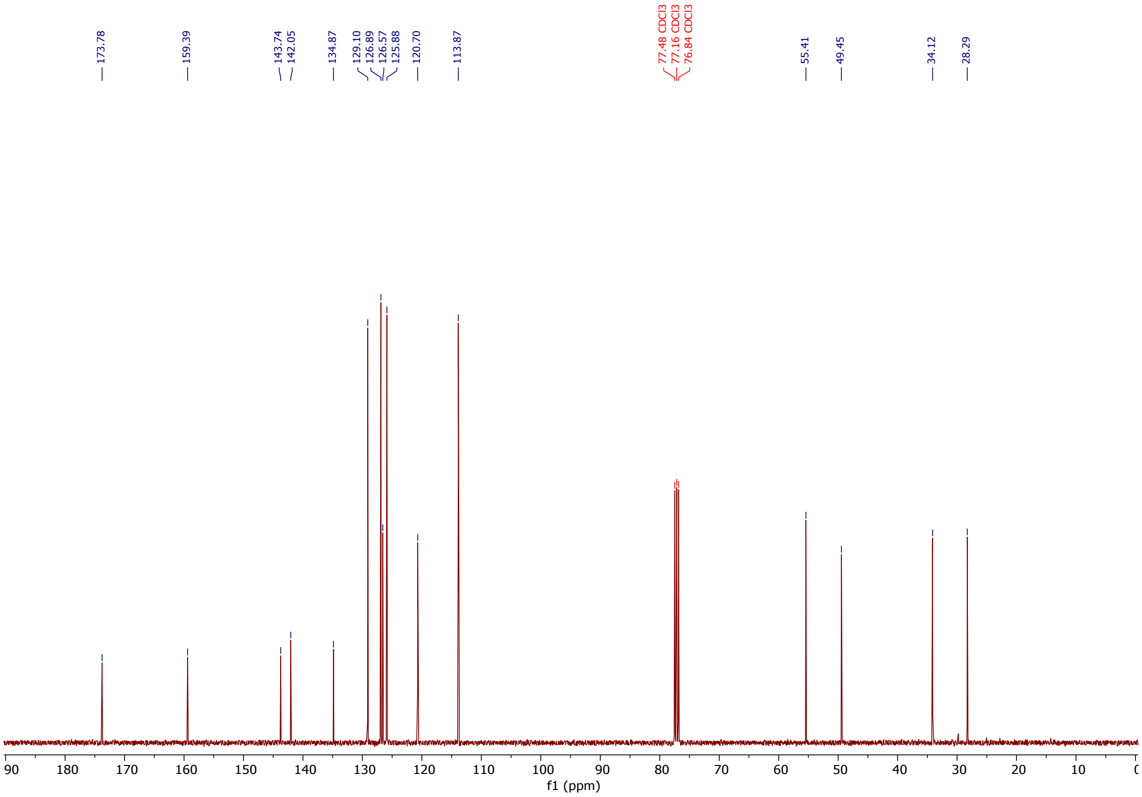

^{13}C NMR (101 MHz, $CDCl_3$) δ 173.78, 159.39, 143.74, 142.05, 134.87, 129.10, 126.89, 126.57, 125.88, 120.70, 113.87, 55.41, 49.45, 34.12, 28.29.

IR spectrum (neat)

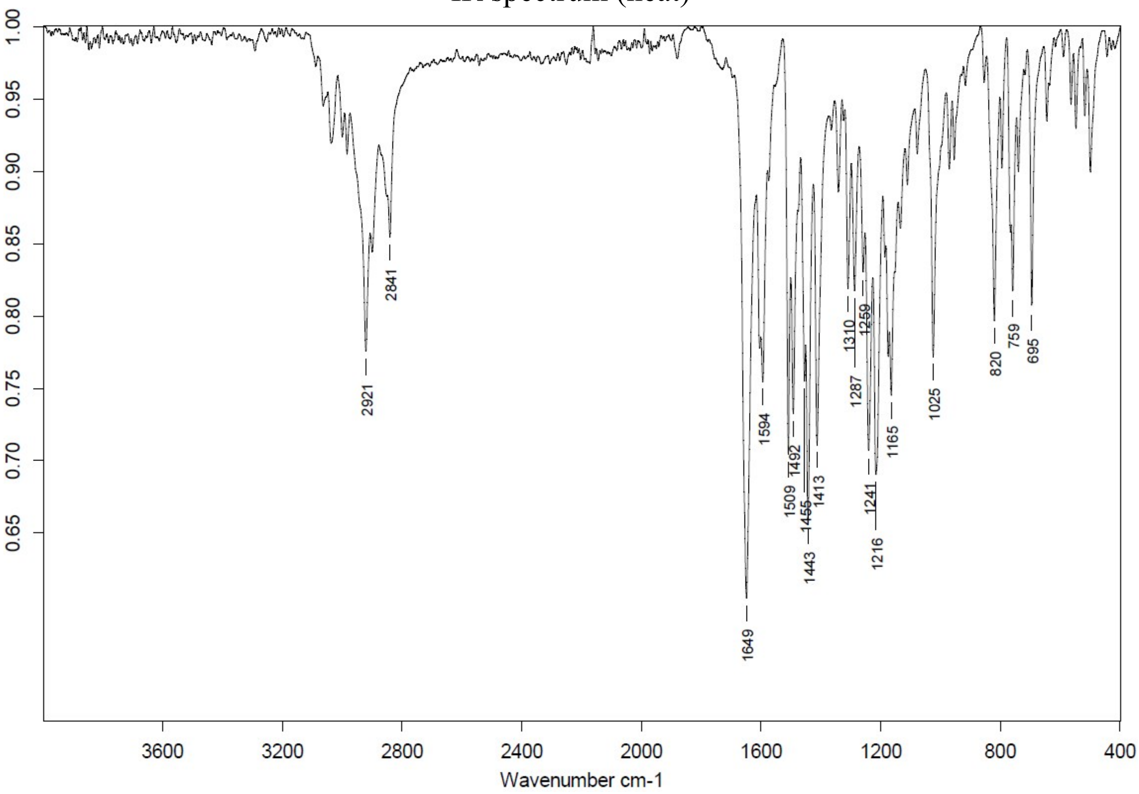

HRMS (ESI$^+$, MeOH): *m/z* calcd. 294.1489 $(M + H)^+$, found: 294.1502.

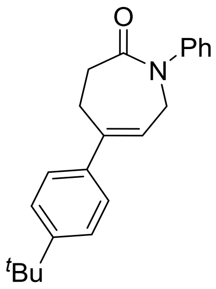

Compound 9

Scale: 0.2 mmol; *E/Z*>99:1, isolated 58.1 mg (91% yield), white solid, Hexane : EA = 2 : 1, R_f = 0.25

^{1}H NMR spectrum ($CDCl_3$)

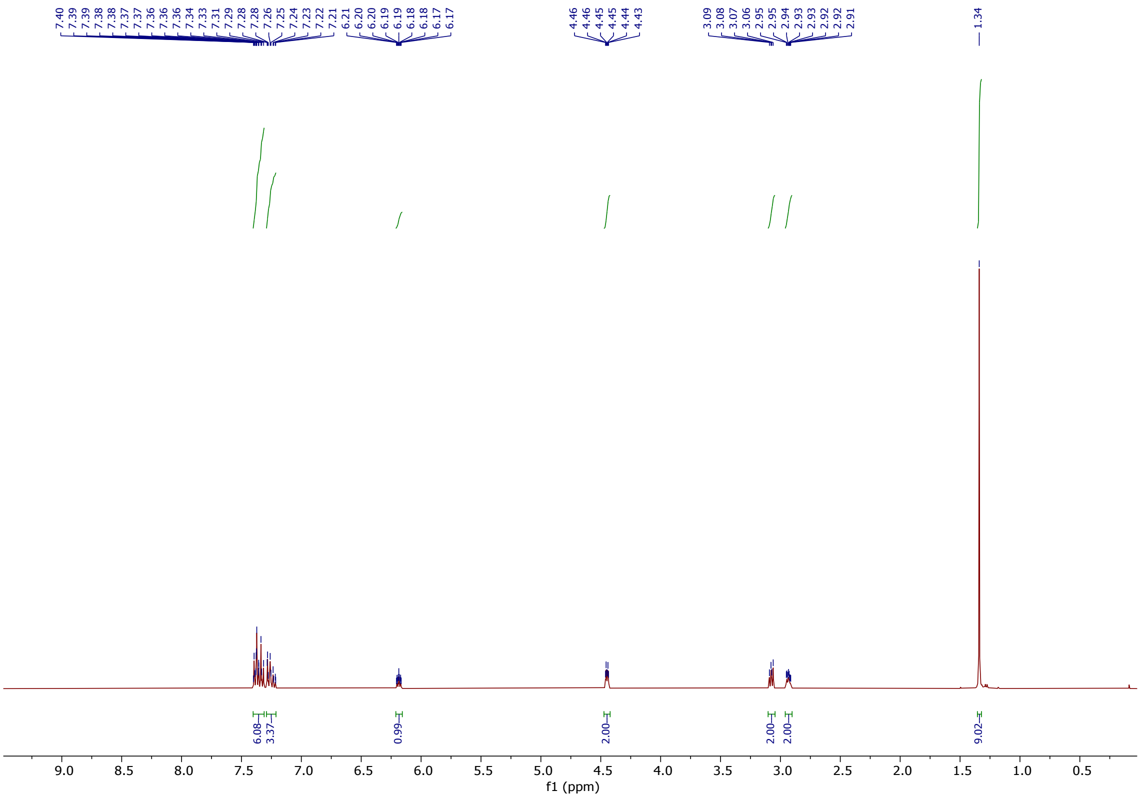

^{1}H NMR (400 MHz, $CDCl_3$) δ 7.40 – 7.31 (m, 6H), 7.29 – 7.21 (m, 3H), 6.19 (tt, *J* = 6.1, 1.8 Hz, 1H), 4.45 (dt, *J* = 6.1, 2.1 Hz, 2H), 3.10 – 3.03 (m, 2H), 2.96 – 2.90 (m, 2H), 1.34 (s, 9H).

^{13}C NMR spectrum ($CDCl_3$)

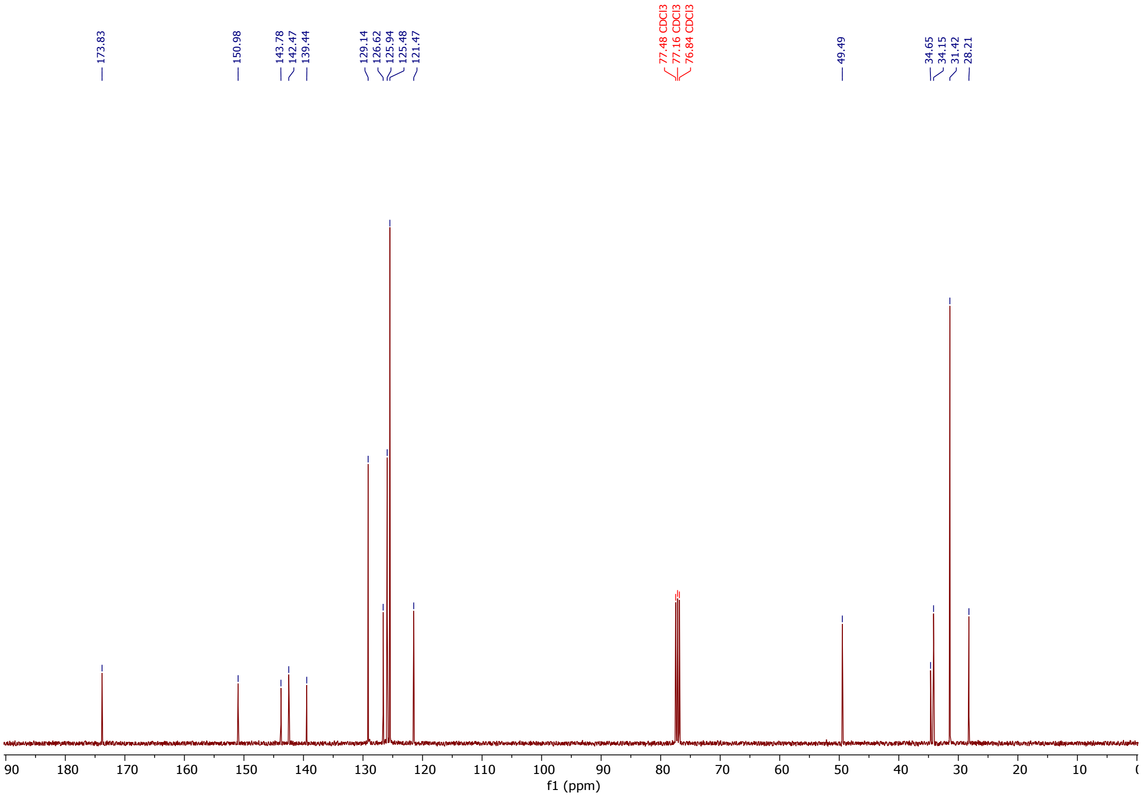

^{13}C NMR (101 MHz, $CDCl_3$) δ 173.83, 150.98, 143.78, 142.47, 139.44, 129.14, 126.62, 125.94, 125.48, 121.47, 49.49, 34.65, 34.15, 31.42, 28.21.

IR spectrum (neat)

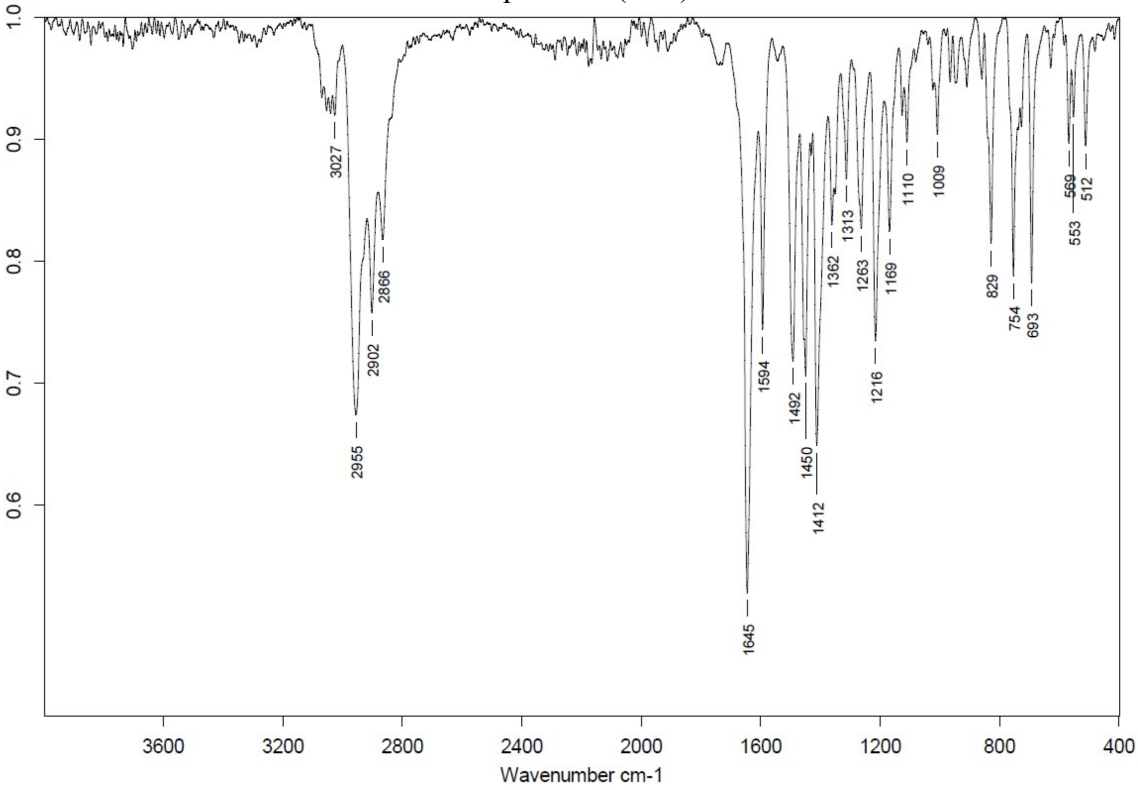

HRMS (ESI^+, MeOH): *m/z* calcd. 320.2009 $(M + H)^+$, found: 320.2008.

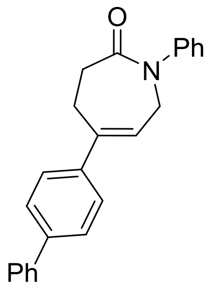

Compound 10

Scale: 0.2 mmol; E/Z = 97:3, isolated 54.3 mg (80% yield), white solid, Hexane : EA = 2 : 1, R_f = 0.20

^{1}H NMR spectrum ($CDCl_3$)

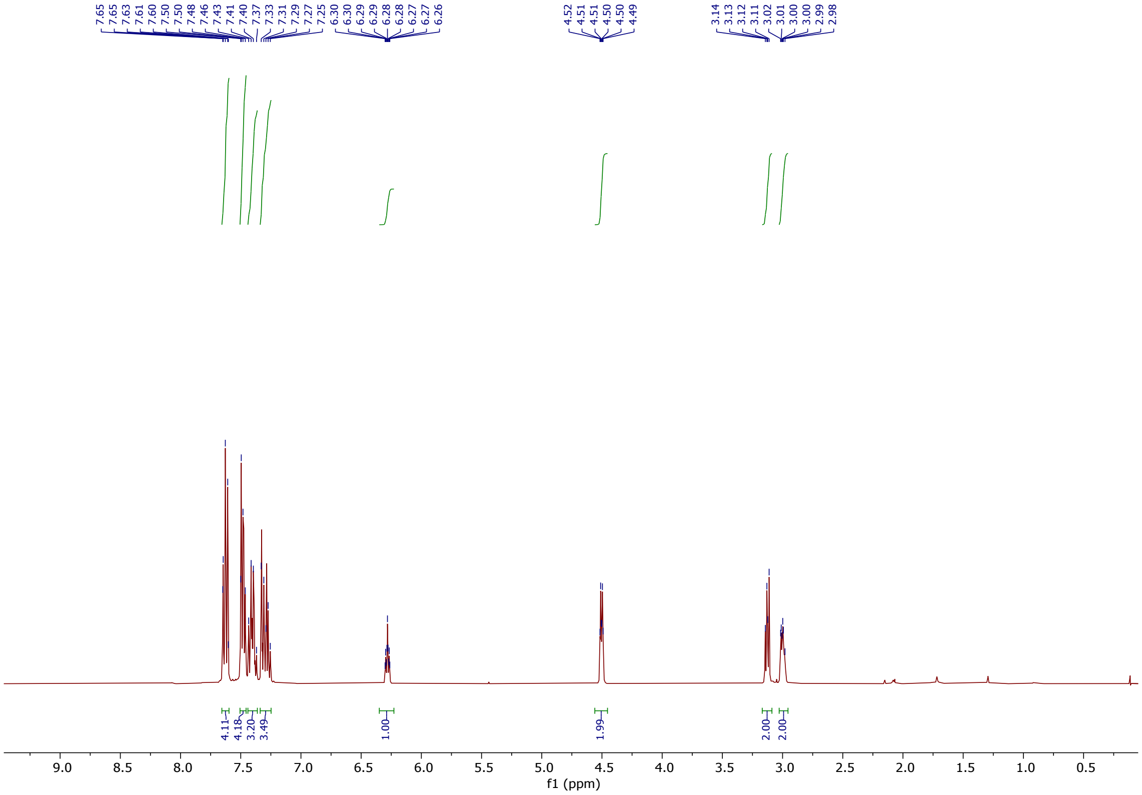

^{1}H NMR (400 MHz, $CDCl_3$) δ 7.66 – 7.60 (m, 4H), 7.51 – 7.46 (m, 4H), 7.44 – 7.36 (m, 3H), 7.34 – 7.25 (m, 3H), 6.28 (tt, J = 6.1, 1.8 Hz, 1H), 4.50 (dt, J = 6.1, 2.1 Hz, 2H), 3.18 – 3.09 (m, 2H), 3.03 – 2.95 (m, 2H).

^{13}C NMR spectrum ($CDCl_3$)

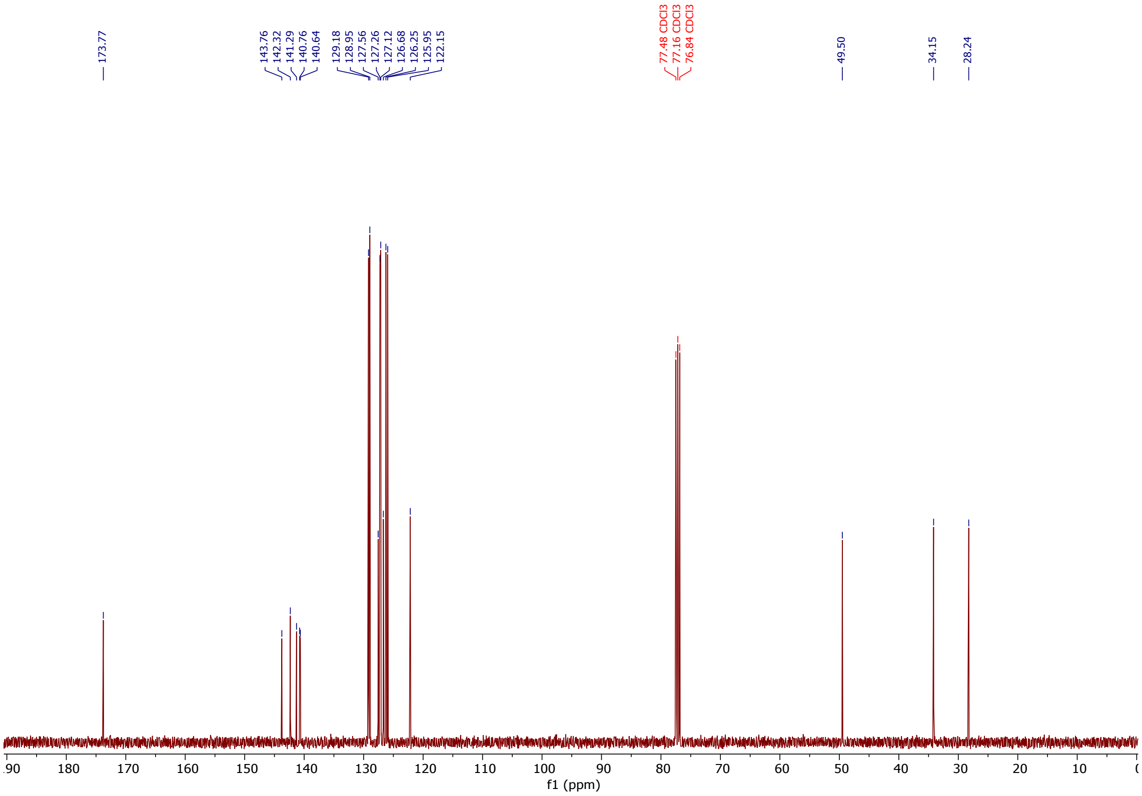

^{13}C NMR (101 MHz, $CDCl_3$) δ 173.77, 143.76, 142.32, 141.29, 140.76, 140.64, 129.18, 128.95, 127.56, 127.26, 127.12, 126.68, 126.25, 125.95, 122.15, 49.50, 34.15, 28.24.

IR spectrum (neat)

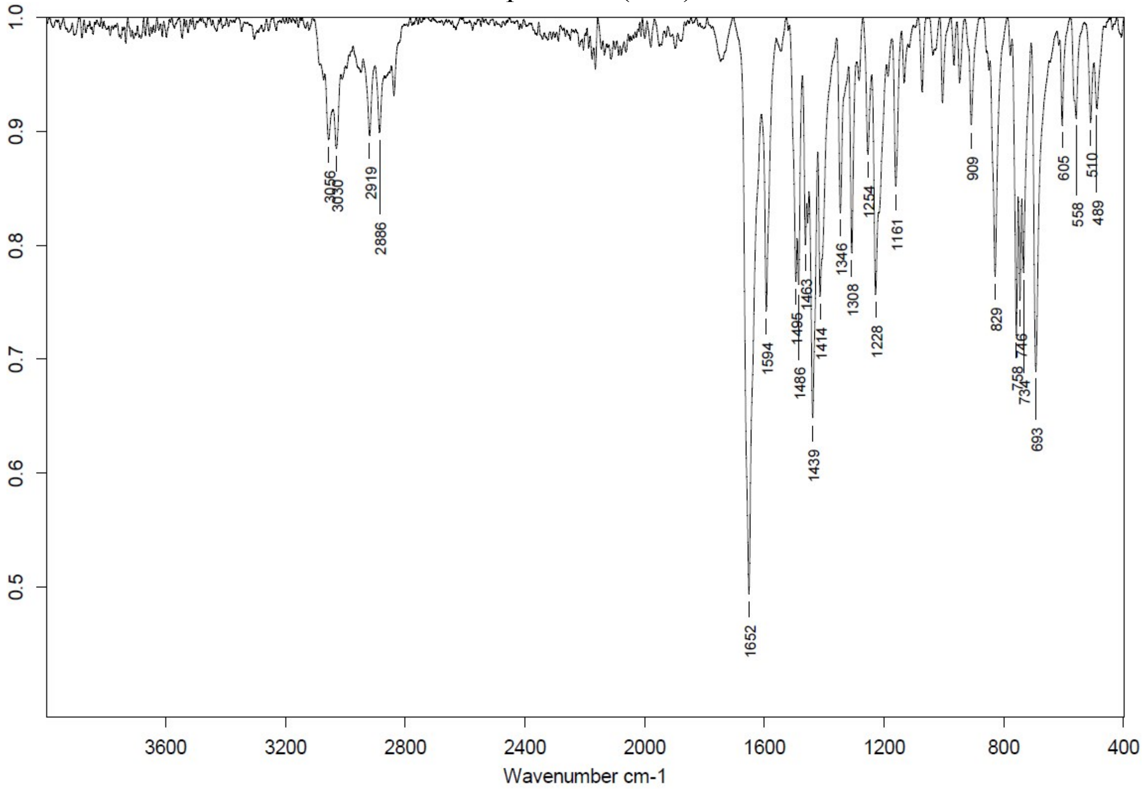

HRMS (ESI^+, MeOH): *m/z* calcd. 340.1696 $(M + H)^+$, found: 340.1696.

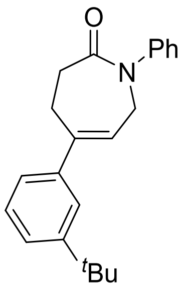

Compound 11

Scale: 0.2 mmol; *E/Z* = 97:3, isolated 47.3 mg (74% yield), white solid, Hexane : EA = 2 : 1, R_f = 0.25

^{1}H NMR spectrum ($CDCl_3$)

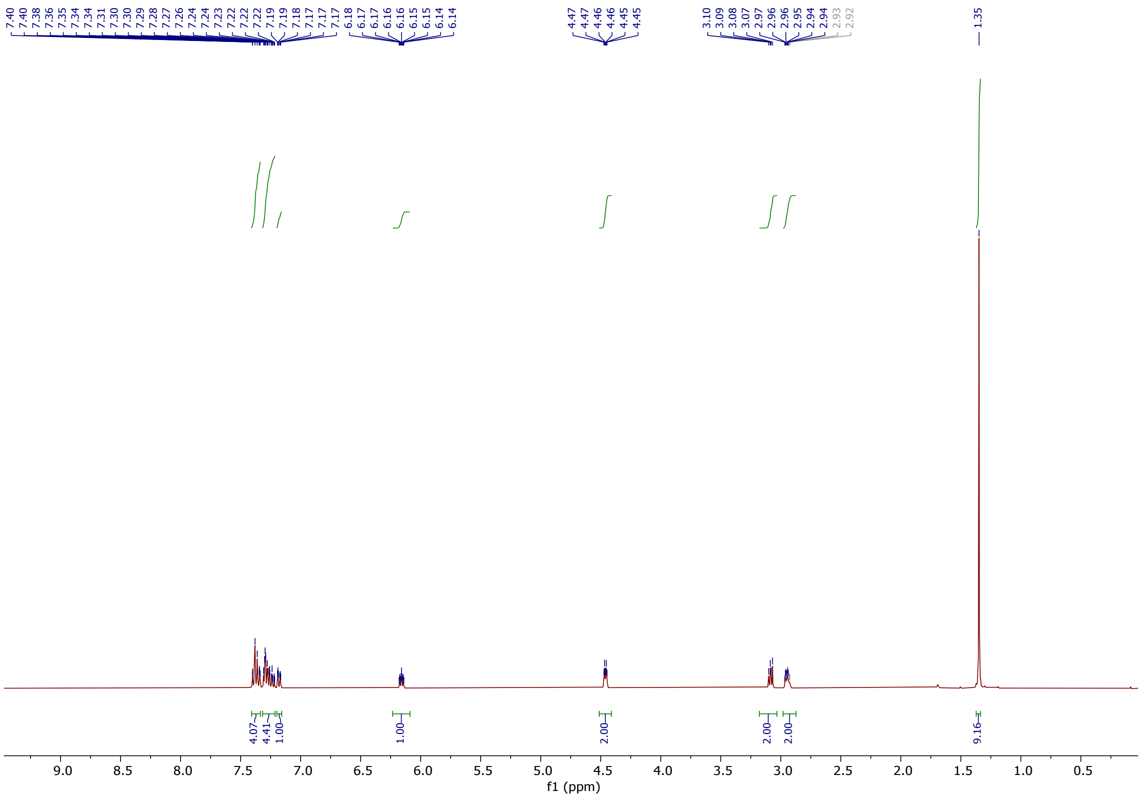

^{1}H NMR (400 MHz, $CDCl_3$) δ 7.41 – 7.33 (m, 4H), 7.32 – 7.21 (m, 4H), 7.20 – 7.16 (m, 1H), 6.16 (tt, *J* = 6.1, 1.9 Hz, 1H), 4.46 (dt, *J* = 6.1, 2.1 Hz, 2H), 3.18 – 3.03 (m, 2H), 2.97 – 2.87 (m, 2H), 1.35 (s, 9H).

^{13}C NMR spectrum ($CDCl_3$)

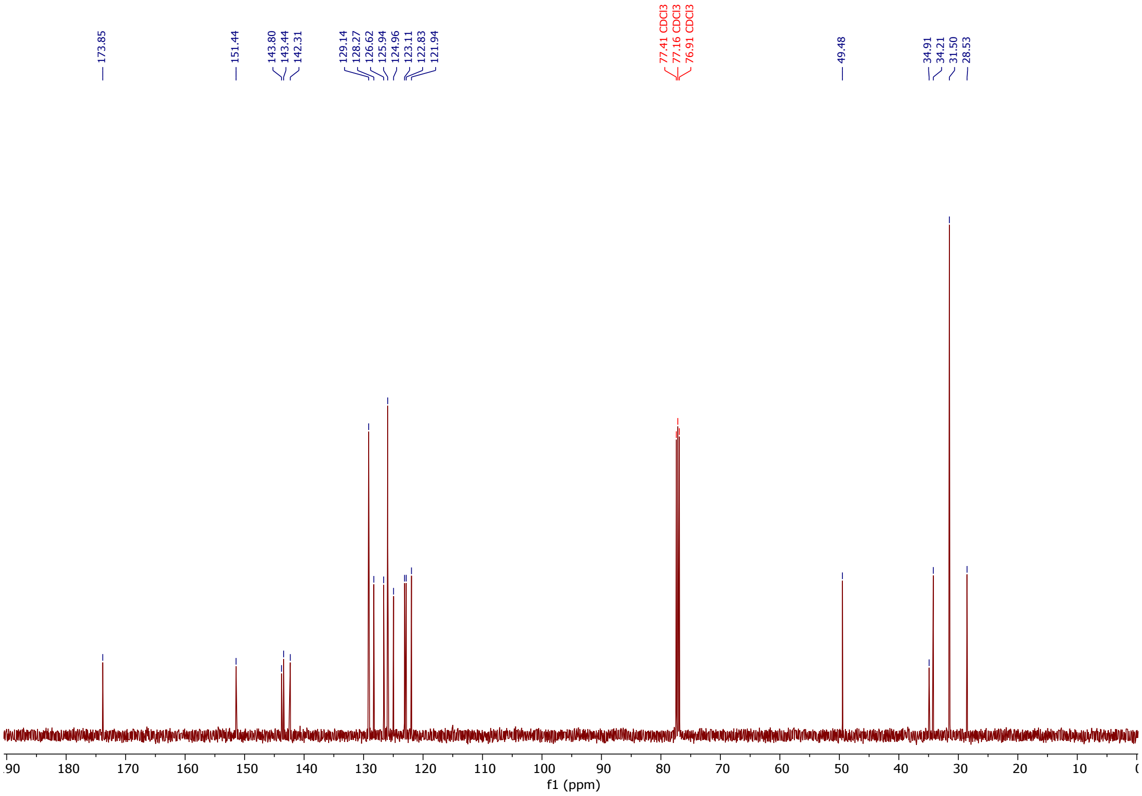

^{13}C NMR (126 MHz, $CDCl_3$) δ 173.85, 151.44, 143.80, 143.44, 142.31, 129.14, 128.27, 126.62, 125.94, 124.96, 123.11, 122.83, 121.94, 49.48, 34.91, 34.21, 31.50, 28.53.

IR spectrum (neat)

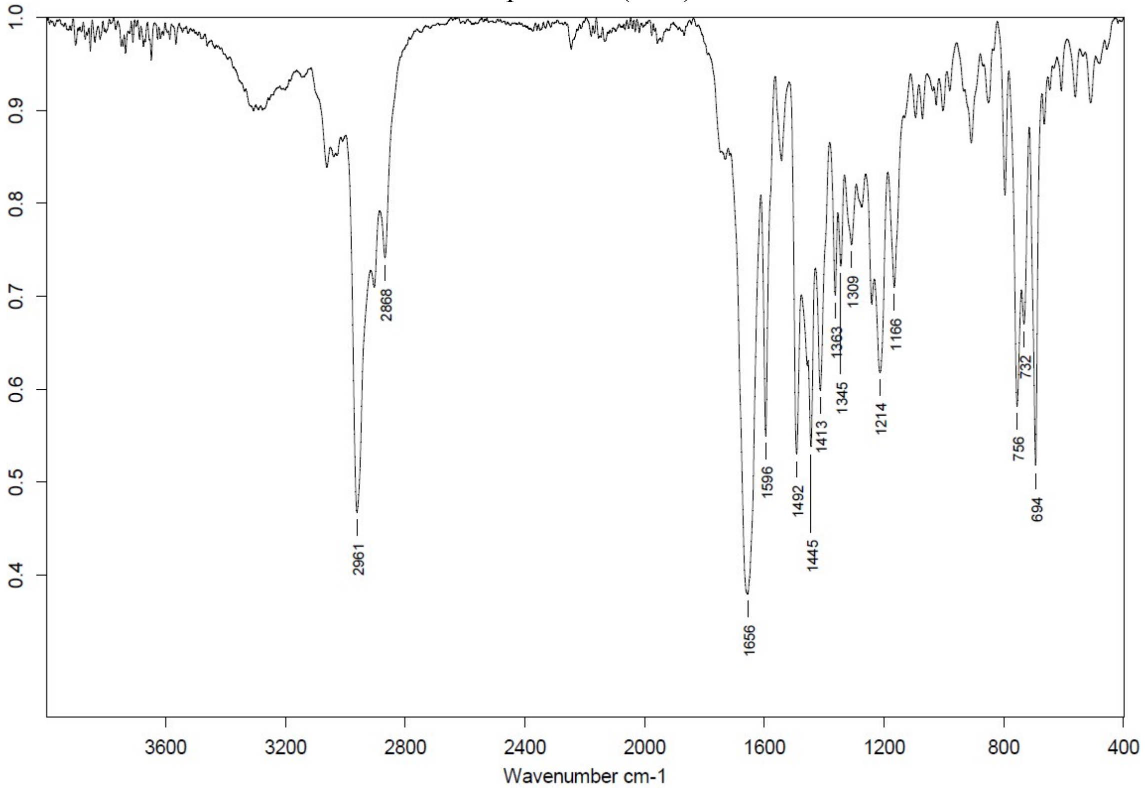

HRMS (ESI$^+$, MeOH): *m/z* calcd. 320.2009 (M + H)$^+$, found: 320.2013.

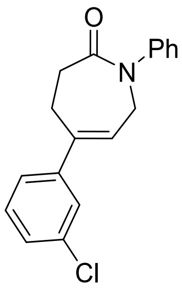

Compound 12

Scale: 0.2 mmol; *E/Z* = 96:4, isolated 53.6 mg (90% yield), yellow solid, Hexane : EA = 2 : 1, R_f = 0.25

^{1}H NMR spectrum ($CDCl_3$)

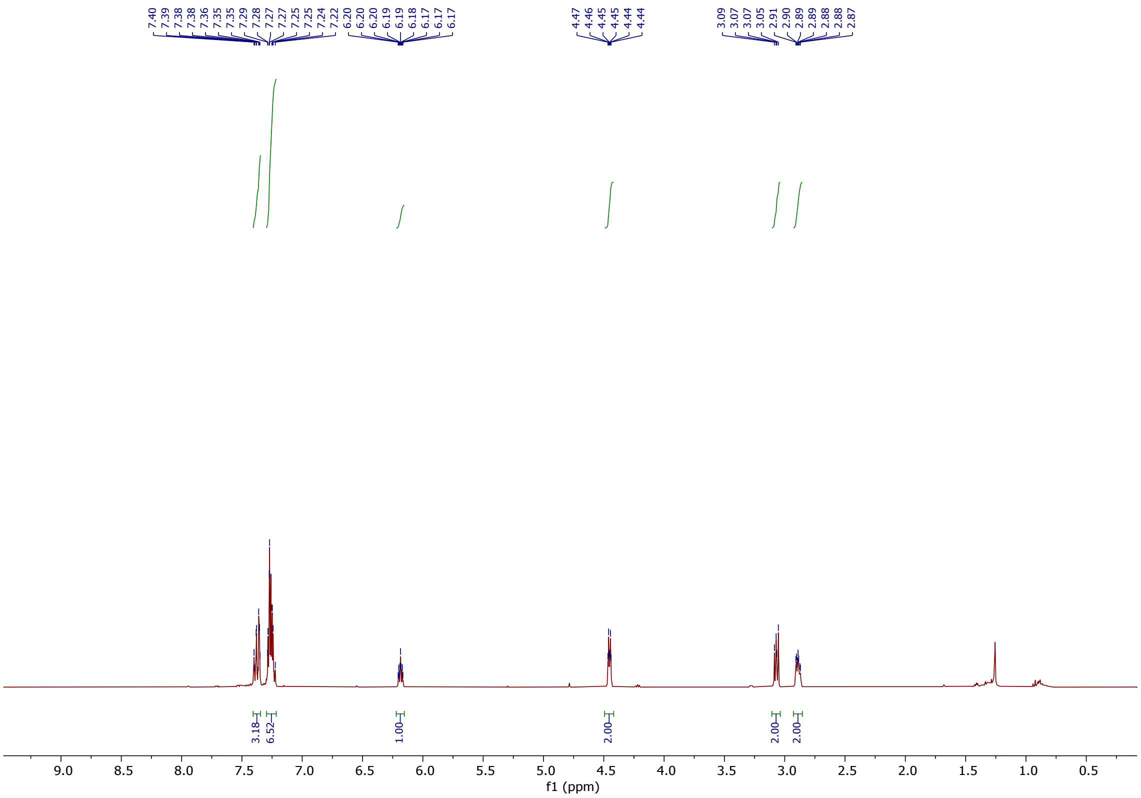

^{1}H NMR (400 MHz, $CDCl_3$) δ 7.41 – 7.35 (m, 3H), 7.30 – 7.22 (m, 6H), 6.18 (tt, *J* = 6.0, 1.9 Hz, 1H), 4.45 (dt, *J* = 6.1, 2.2 Hz, 2H), 3.11 – 3.04 (m, 2H), 2.93 – 2.85 (m, 2H).

^{13}C NMR spectrum ($CDCl_3$)

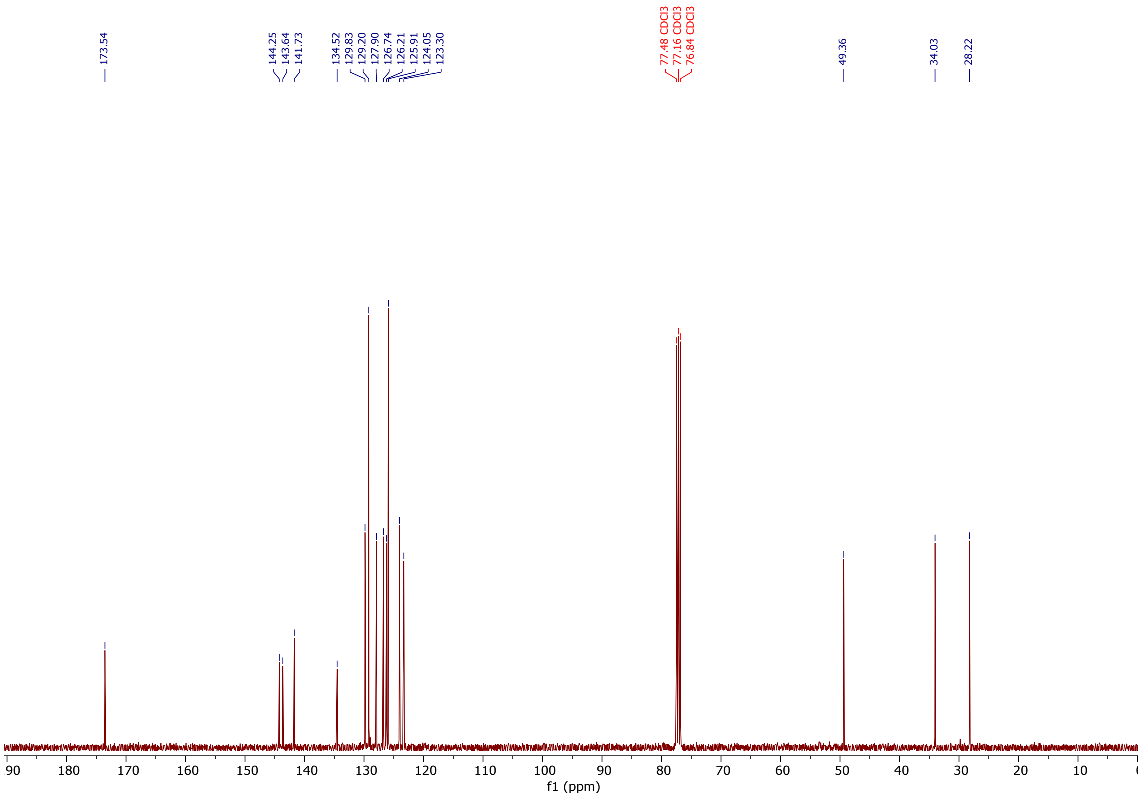

^{13}C NMR (101 MHz, $CDCl_3$) δ 173.54, 144.25, 143.64, 141.73, 134.52, 129.83, 129.20, 127.90, 126.74, 126.21, 125.91, 124.05, 123.30, 49.36, 34.03, 28.22.

IR spectrum (neat)

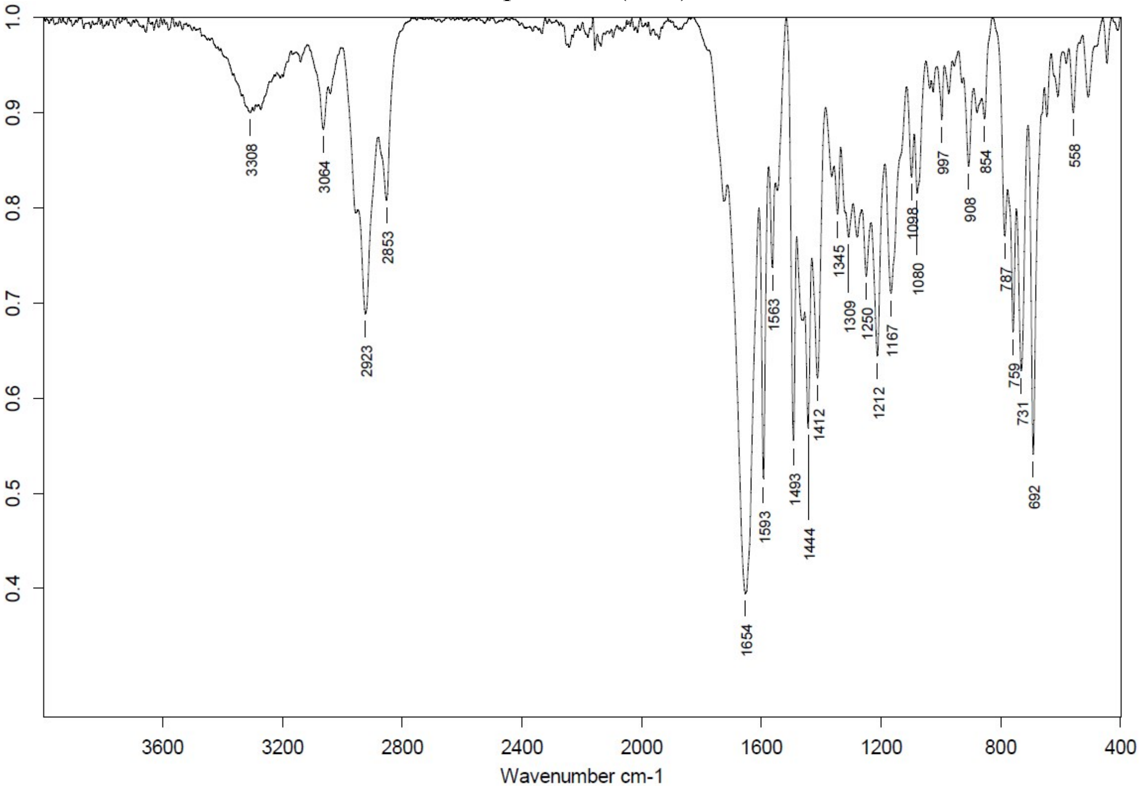

HRMS (ESI^+, MeOH): *m/z* calcd. 298.0993 $(M + H)^+$, found: 298.0993.

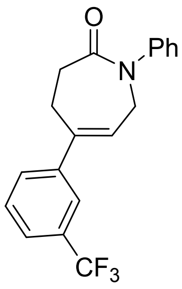

Compound 13

Scale: 0.2 mmol; *E/Z* = 97:3, isolated 56.3 mg (85% yield), yellow solid, Hexane : EA = 2 : 1, R_f = 0.15

^{1}H NMR spectrum ($CDCl_3$)

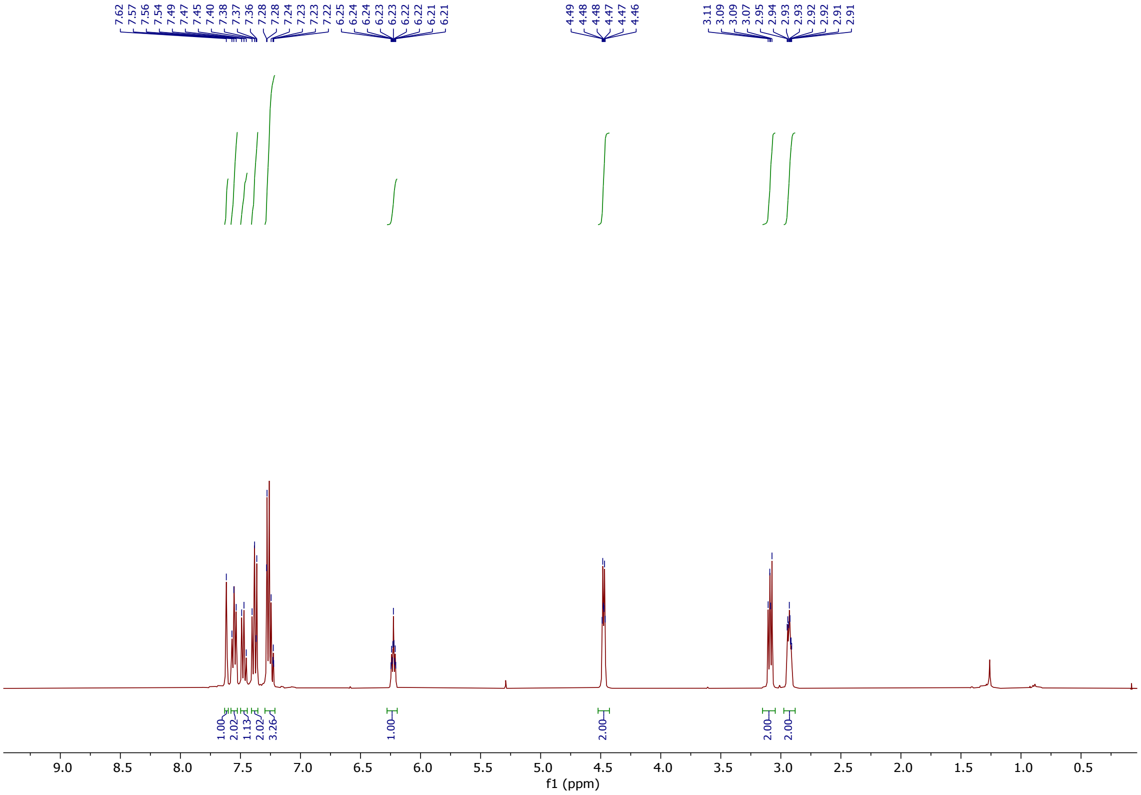

^{1}H NMR (400 MHz, $CDCl_3$) δ 7.62 (s, 1H), 7.58 – 7.53 (m, 2H), 7.49 – 7.44 (m, 1H), 7.41 – 7.35 (m, 2H), 7.30 – 7.21 (m, 3H), 6.23 (tt, *J* = 6.0, 1.8 Hz, 1H), 4.48 (dt, *J* = 6.1, 2.1 Hz, 2H), 3.15 – 3.04 (m, 2H), 2.97 – 2.87 (m, 2H).

^{13}C NMR spectrum ($CDCl_3$)

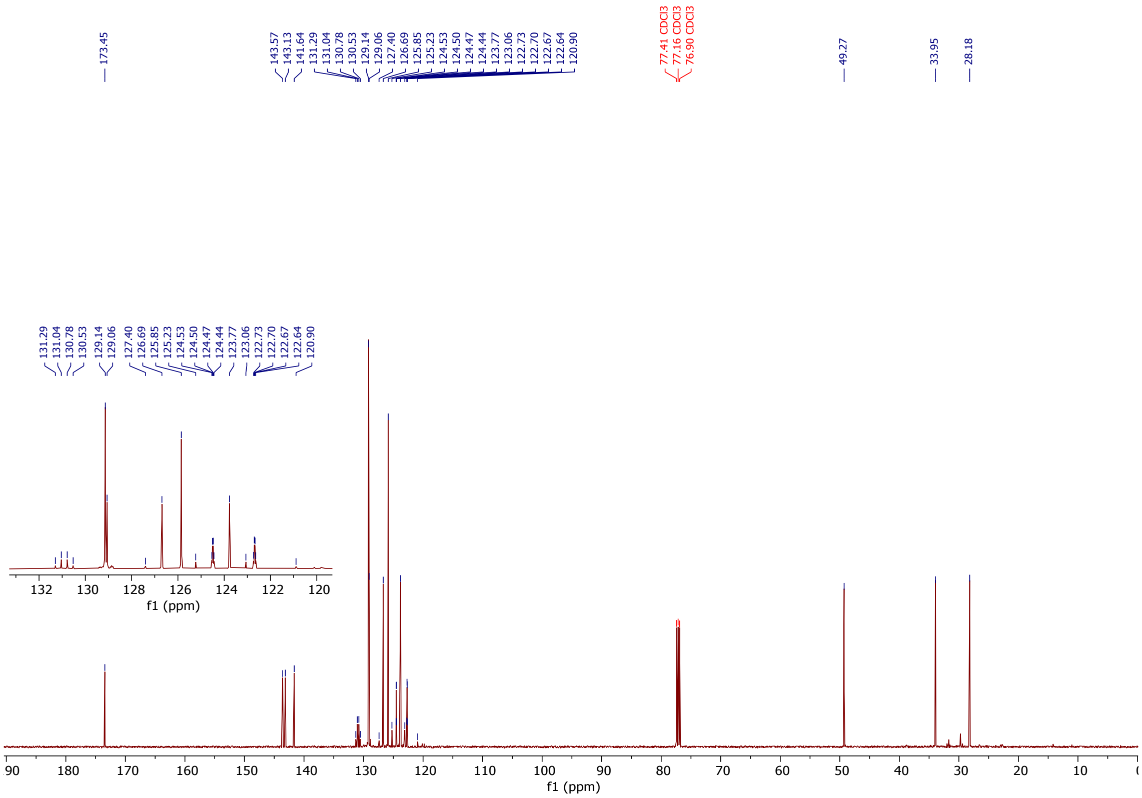

^{13}C NMR (126 MHz, $CDCl_3$) δ 173.45, 143.57, 143.13, 141.64, 130.91 (q, J = 32.3 Hz), 129.14, 129.06, 126.69, 125.85, 124.49 (q, J = 3.8 Hz), 124.15 (q, J = 272.4 Hz), 123.77, 122.68 (q, J = 3.9 Hz), 49.27, 33.95, 28.18.

^{19}F NMR spectrum ($CDCl_3$)

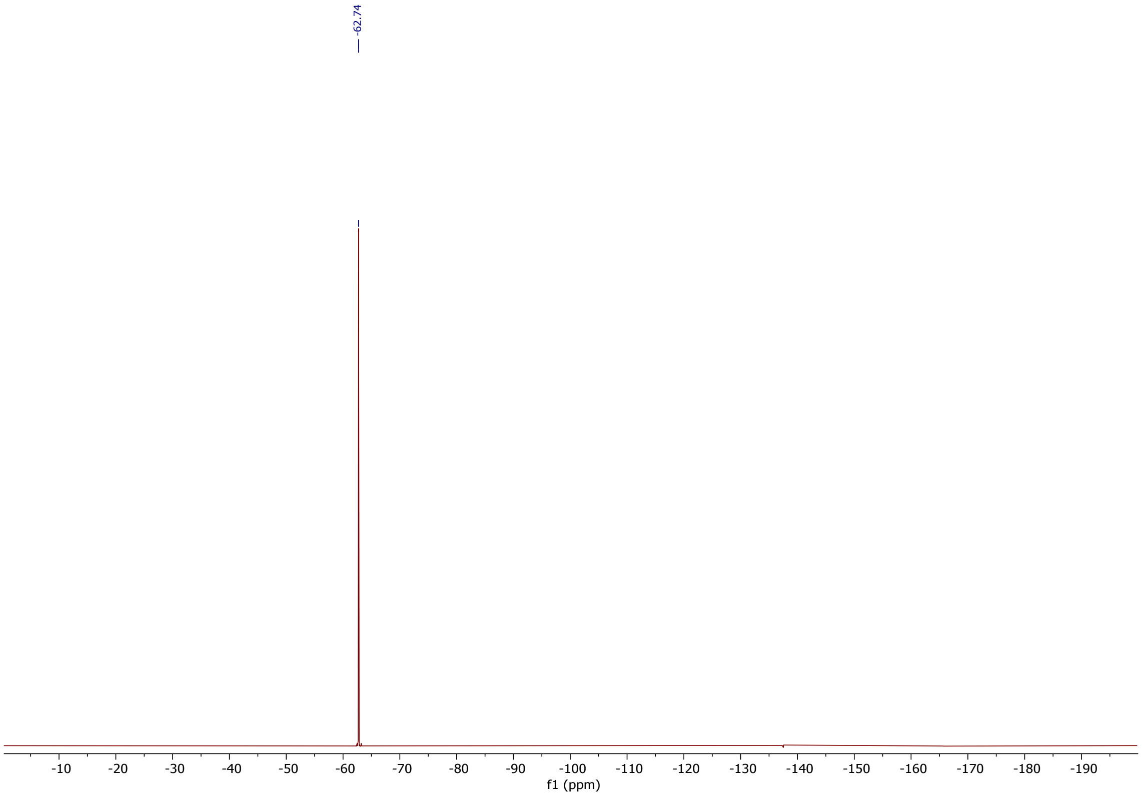

^{19}F NMR (376 MHz, $CDCl_3$) δ -62.74.

IR spectrum (neat)

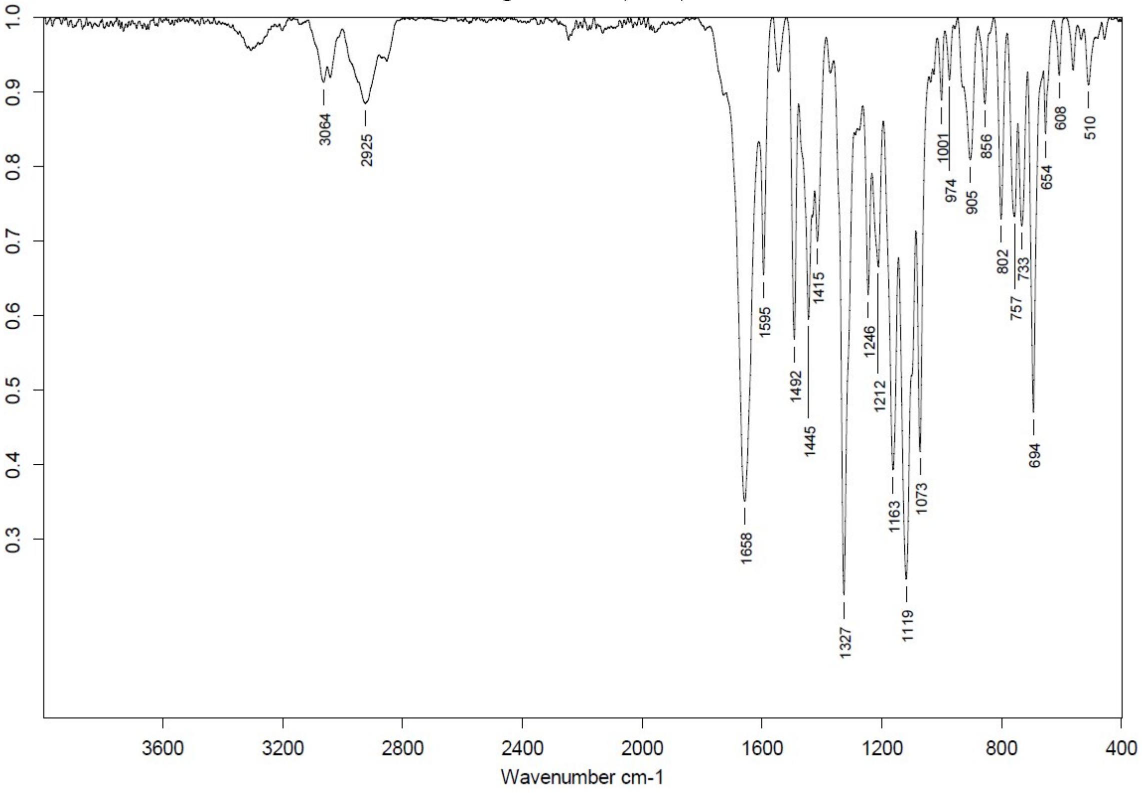

HRMS (ESI$^+$, MeOH): *m/z* calcd. 332.1257 (M + H)$^+$, found: 332.1259.

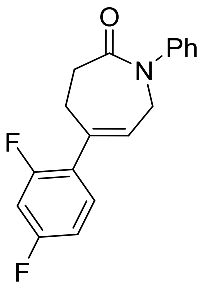

Compound 14

Scale: 0.2 mmol; *E/Z* = 99:1, isolated 46.1 mg (77% yield), yellow solid, Hexane : EA = 2 : 1, R_f = 0.25

^{1}H NMR spectrum ($CDCl_3$)

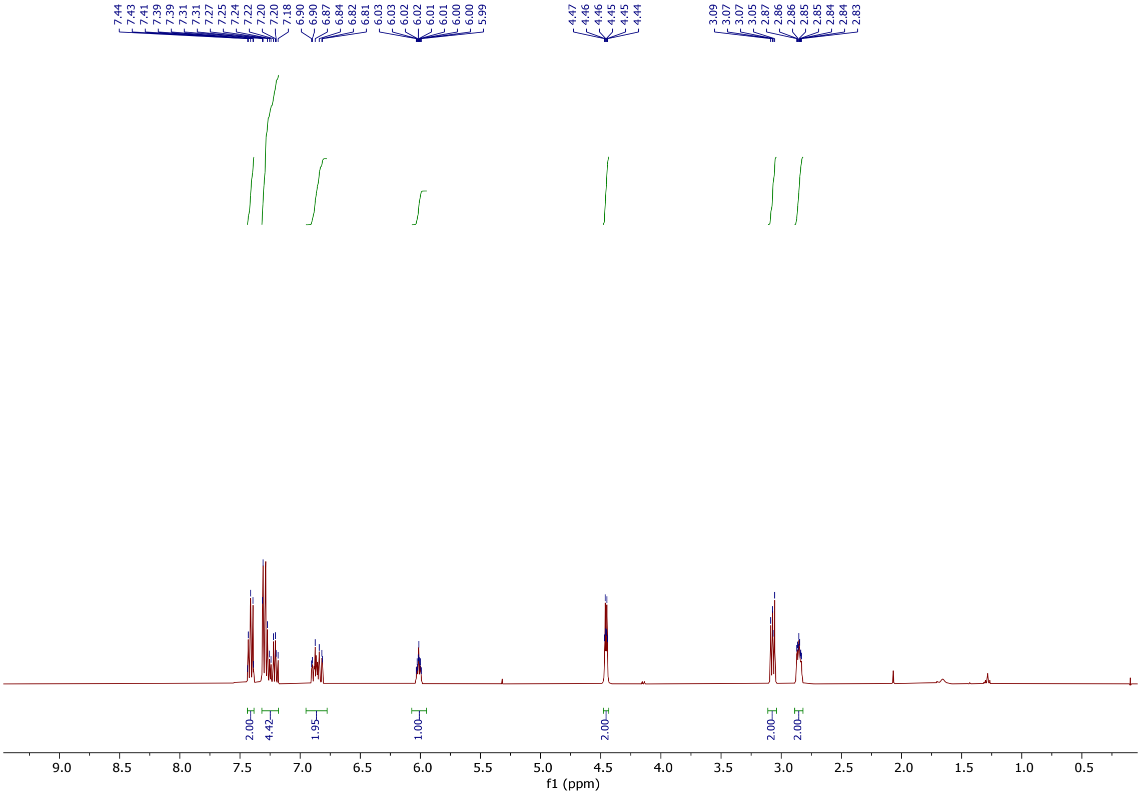

^{1}H NMR (400 MHz, $CDCl_3$) δ 7.44 – 7.38 (m, 2H), 7.32 – 7.18 (m, 4H), 6.95 – 6.78 (m, 2H), 6.01 (tt, *J* = 5.9, 2.2 Hz, 1H), 4.46 (dt, *J* = 5.9, 2.2 Hz, 2H), 3.11 – 3.04 (m, 2H), 2.89 – 2.81 (m, 2H).

^{13}C NMR spectrum ($CDCl_3$)

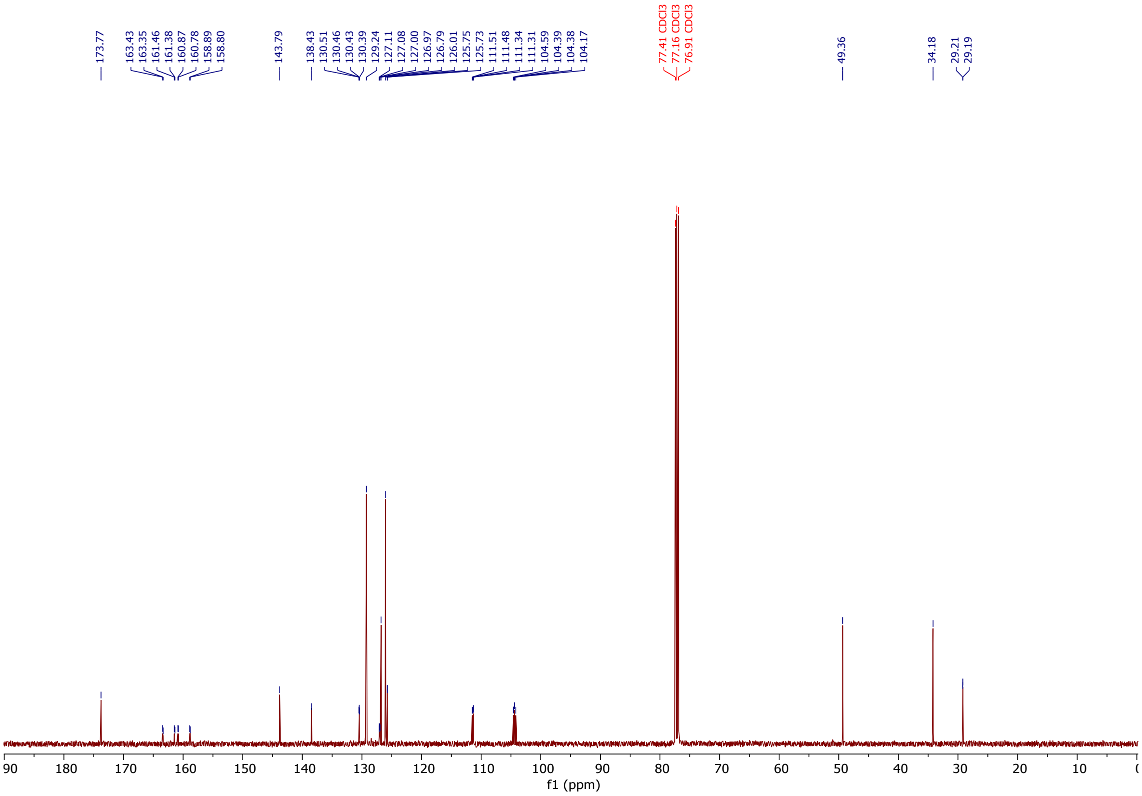

^{13}C NMR (126 MHz, $CDCl_3$) δ 173.77, 162.41 (dd, J = 248.1, 10.9 Hz), 159.84 (dd, J = 249.2, 11.6 Hz), 143.79, 138.43, 130.45 (dd, J = 9.7, 5.5 Hz), 129.24, 127.04 (dd, J = 14.8, 3.7 Hz), 126.79, 126.01, 125.74 (d, J = 2.3 Hz), 111.41 (dd, J = 21.3, 3.7 Hz), 104.38 (dd, J = 26.7, 25.1 Hz), 49.36, 34.18, 29.20 (d, J = 3.2 Hz).

^{19}F NMR spectrum ($CDCl_3$)

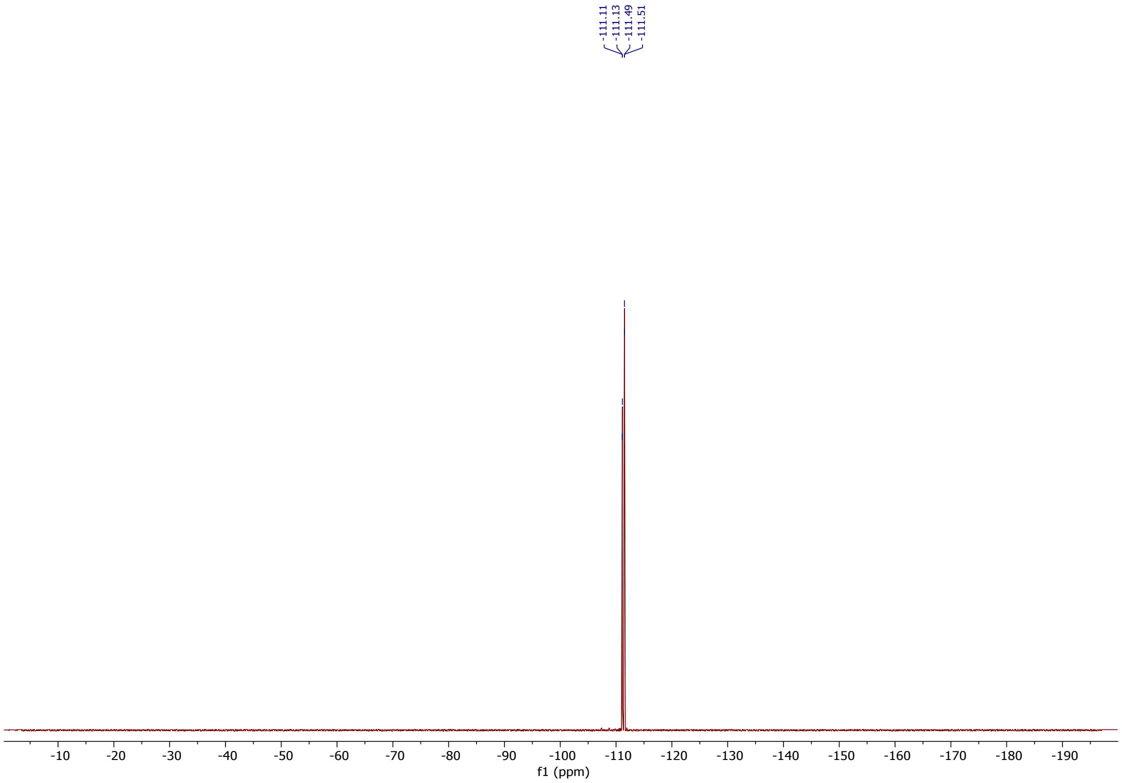

^{19}F NMR (376 MHz, $CDCl_3$) δ -111.12 (d, J = 7.5 Hz), -111.50 (d, J = 7.5 Hz).

IR spectrum (neat)

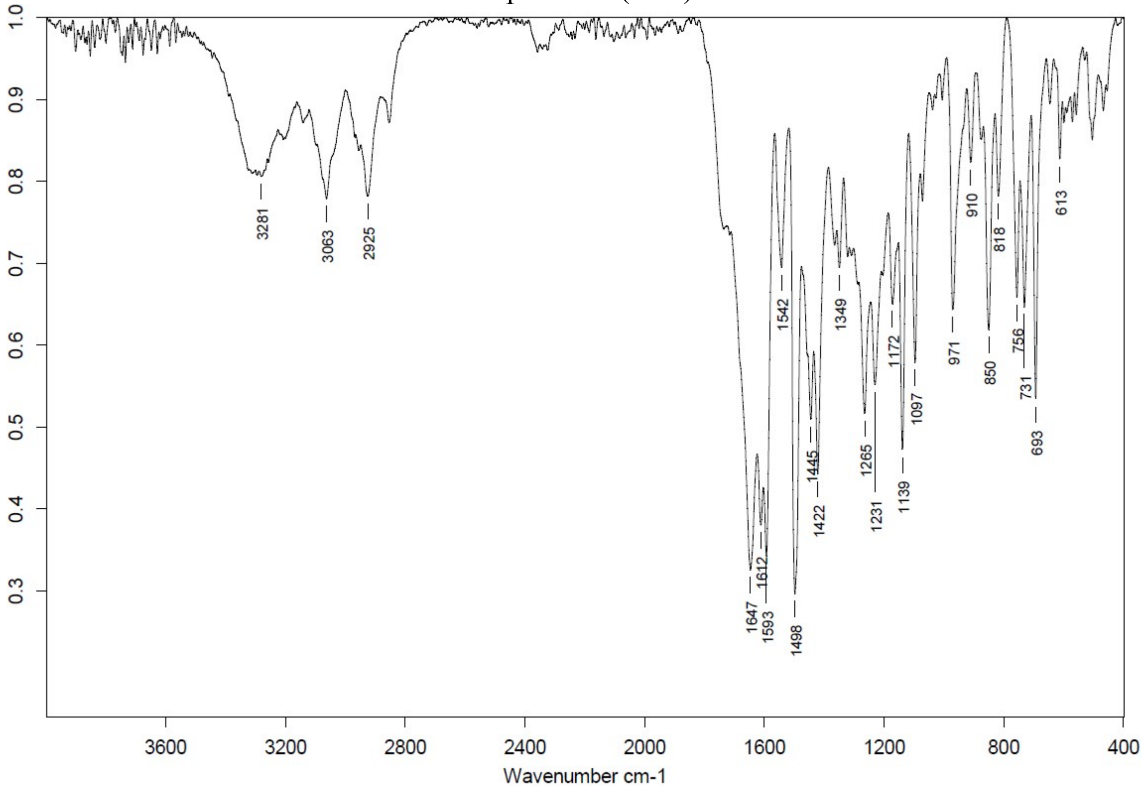

HRMS (ESI$^+$, MeOH): *m/z* calcd. 322.1013 (M + Na)$^+$, found: 322.1014.

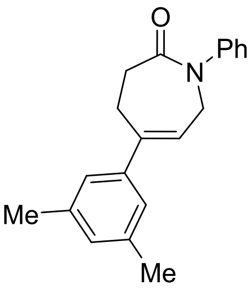

Compound 15

Scale: 0.2 mmol; *E/Z*>99:1, isolated 41.4 mg (71% yield), yellow solid, Hexane : EA = 2 : 1, R_f = 0.25

^{1}H NMR spectrum (CDCl$_3$)

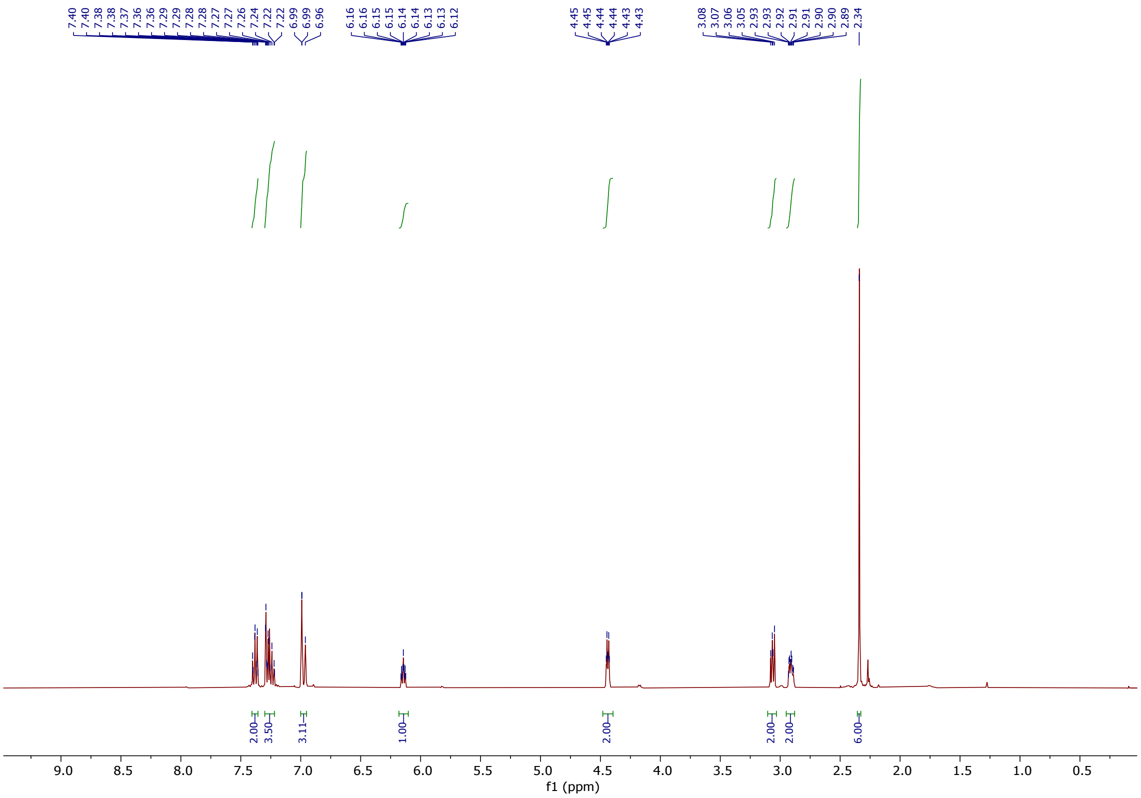

^{1}H NMR (400 MHz, CDCl$_3$) δ 7.41 – 7.36 (m, 2H), 7.30 – 7.22 (m, 3H), 7.00 – 6.95 (m, 3H), 6.14 (tt, J = 6.1, 1.9 Hz, 1H), 4.44 (dt, J = 6.1, 2.1 Hz, 2H), 3.11– 3.02 (m, 2H), 2.95 – 2.87 (m, 2H), 2.34 (s, 6H).

^{13}C NMR spectrum ($CDCl_3$)

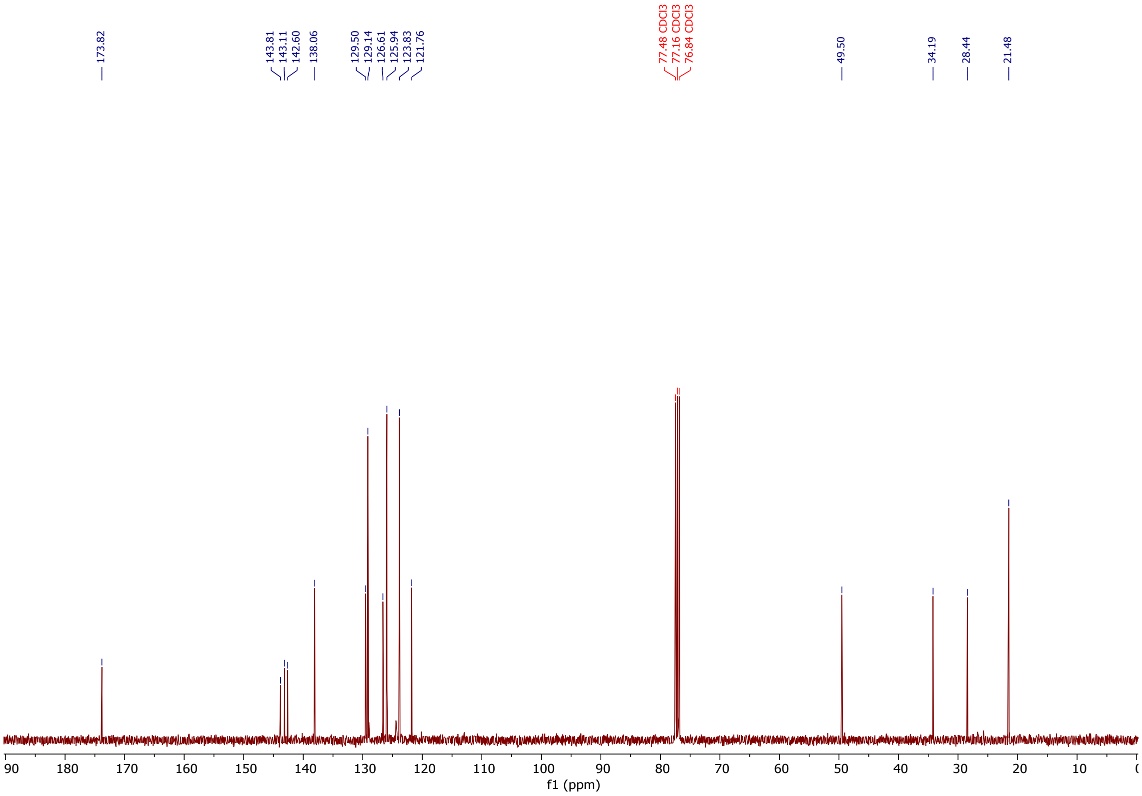

^{13}C NMR (101 MHz, $CDCl_3$) δ 173.82, 143.81, 143.11, 142.60, 138.06, 129.50, 129.14, 126.61, 125.94, 123.83, 121.76, 49.50, 34.19, 28.44, 21.48.

IR spectrum (neat)

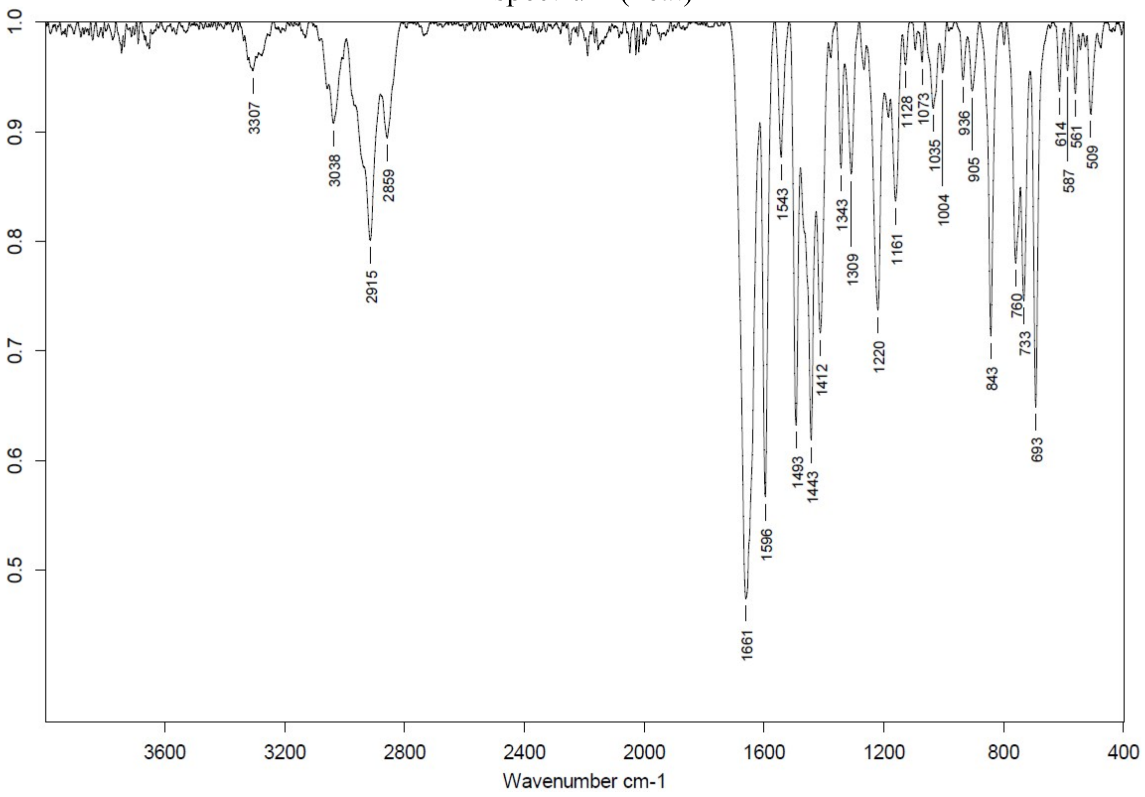

HRMS (ESI$^+$, MeOH): *m/z* calcd. 292.1698 $(M + H)^+$, found: 292.1696.

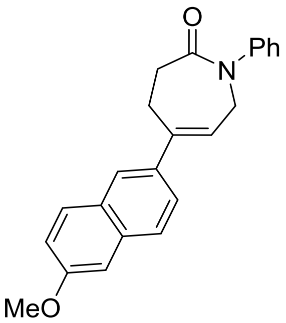

Compound 16

Scale: 0.2 mmol; *E/Z*>99:1, isolated 44.6 mg (65% yield), white solid, Hexane : EA = 2 : 1, R_f = 0.15

^{1}H NMR spectrum ($CDCl_3$)

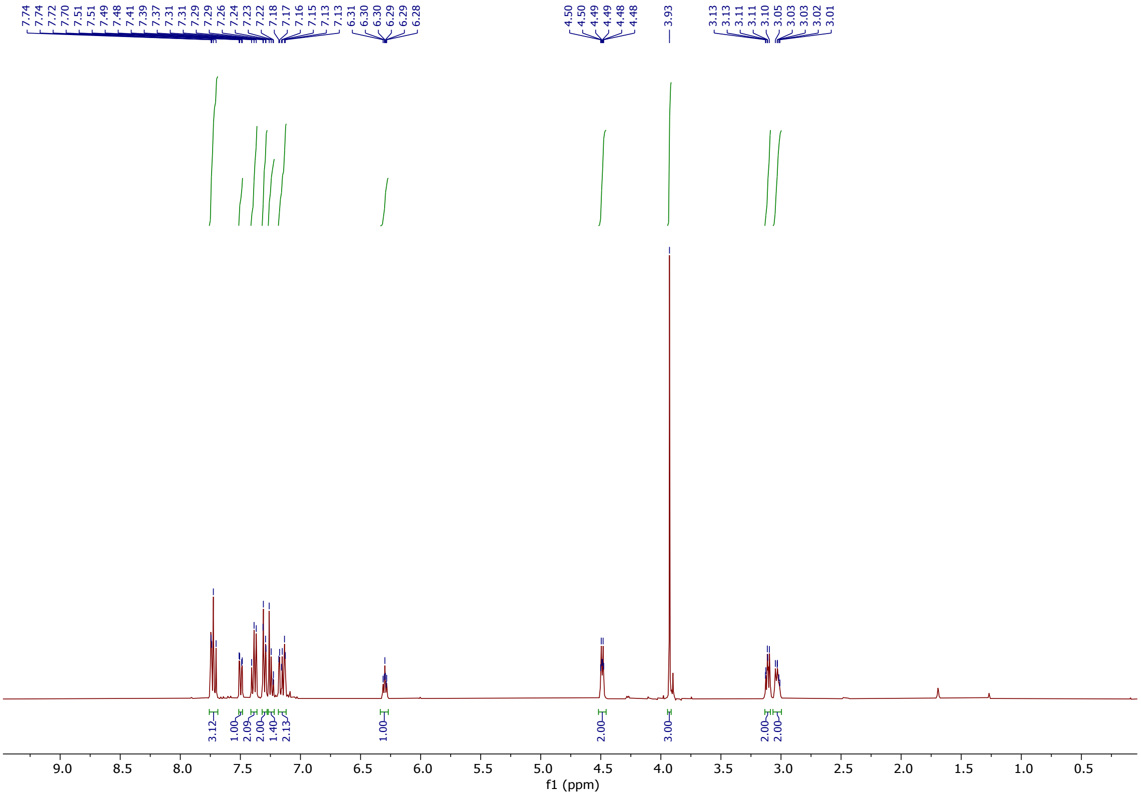

^{1}H NMR (400 MHz, $CDCl_3$) δ 7.76 – 7.69 (m, 3H), 7.50 (dd, J = 8.6, 2.0 Hz, 1H), 7.41 – 7.36 (m, 2H), 7.32 – 7.27 (m, 2H), 7.27 – 7.22 (m, 1H), 7.18 – 7.12 (m, 2H), 6.30 (tt, J = 6.1, 1.8 Hz, 1H), 4.49 (dt, J = 6.1, 2.0 Hz, 2H), 3.93 (s, 3H), 3.13 – 3.09 (m, 2H), 3.07 – 3.00 (m, 2H).

^{13}C NMR spectrum ($CDCl_3$)

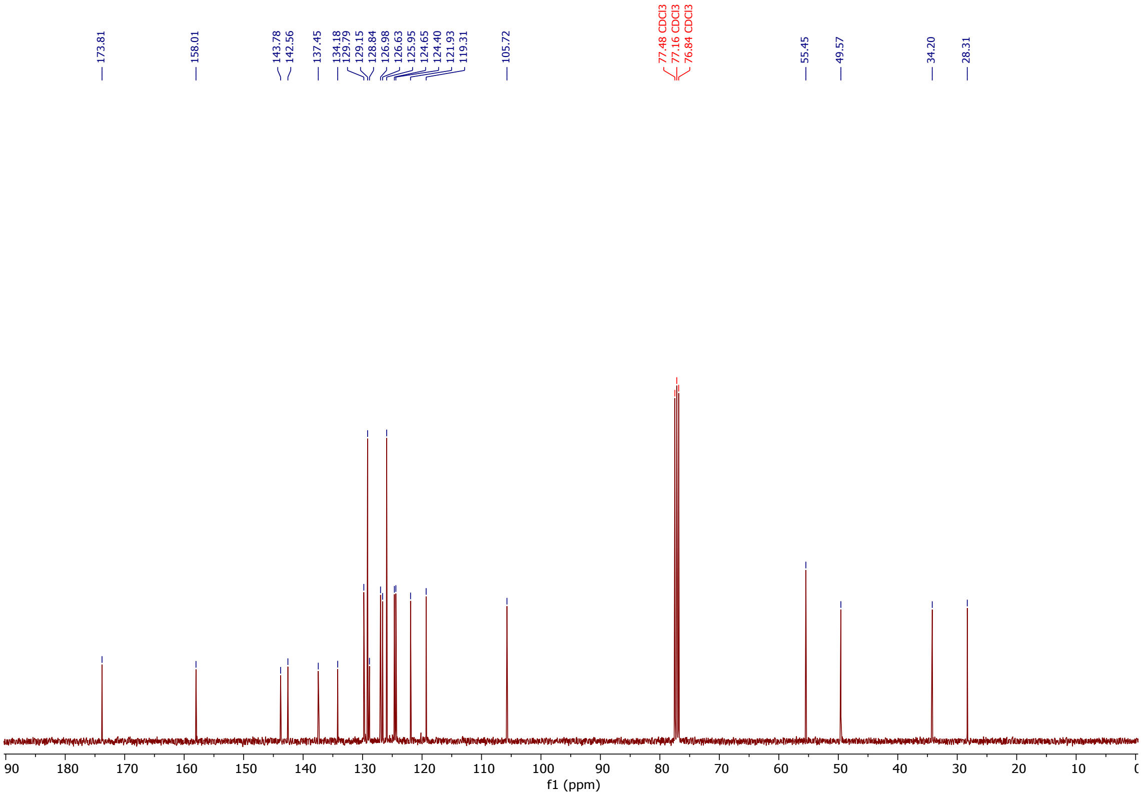

^{13}C NMR (101 MHz, $CDCl_3$) δ 173.81, 158.01, 143.78, 142.56, 137.45, 134.18, 129.79, 129.15, 128.84, 126.98, 126.63, 125.95, 124.65, 124.40, 121.93, 119.31, 105.72, 55.45, 49.57, 34.20, 28.31.

IR spectrum (neat)

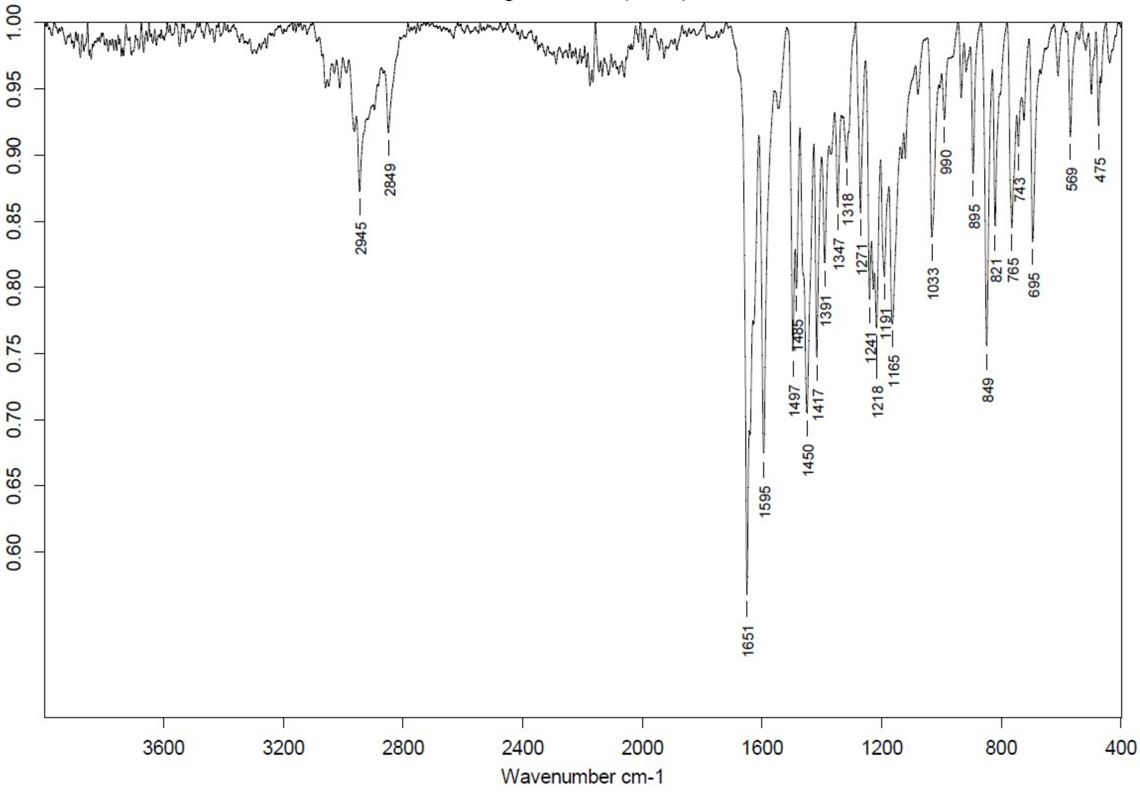

HRMS (ESI$^+$, MeOH): *m/z* calcd. 344.1645 $(M + H)^+$, found: 344.1646.

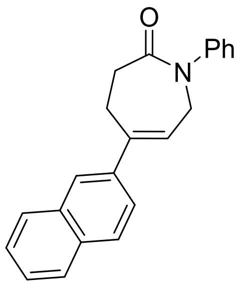

Compound 17

Scale: 0.2 mmol; *E/Z* = 99:1, isolated 48.3 mg (77% yield), white solid, Hexane : EA = 2 : 1, R_f = 0.18

^{1}H NMR spectrum ($CDCl_3$)

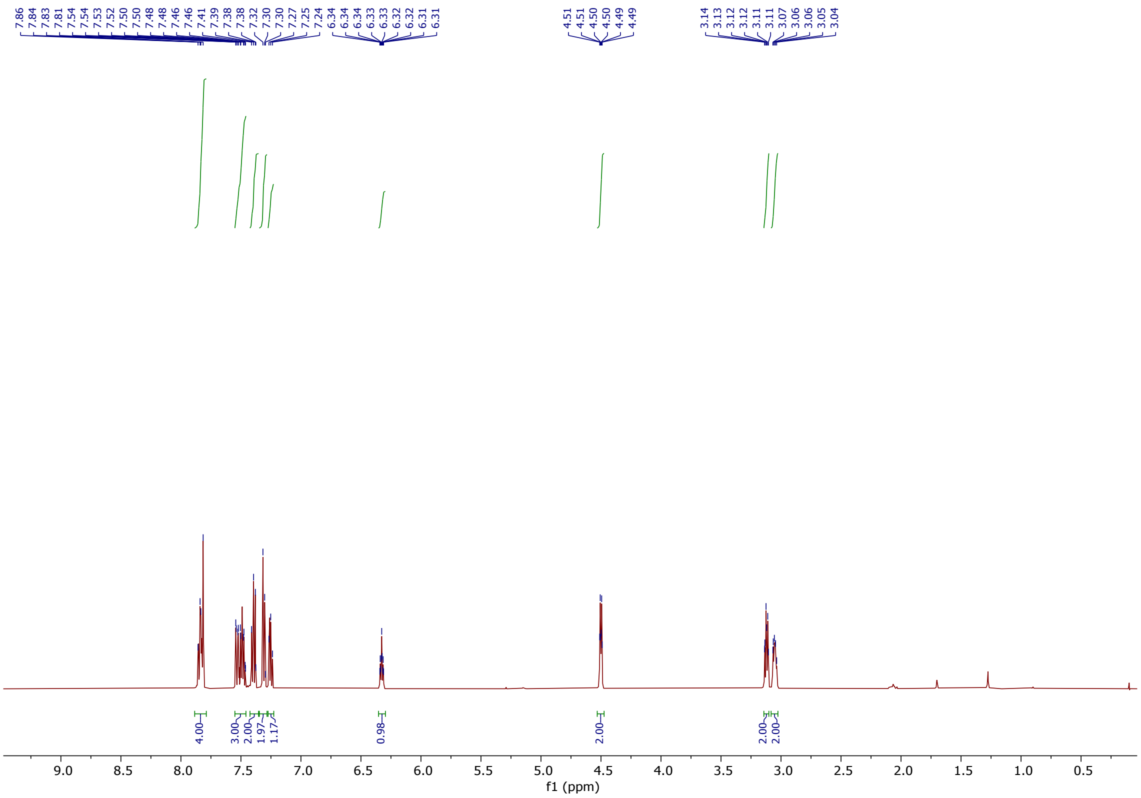

^{1}H NMR (500 MHz, $CDCl_3$) δ 7.89 – 7.79 (m, 4H), 7.55 – 7.46 (m, 3H), 7.42 – 7.35 (m, 2H), 7.31 (d, J = 7.3 Hz, 2H), 7.27 – 7.22 (m, 1H), 6.33 (tt, J = 6.1, 1.7 Hz, 1H), 4.50 (dt, J = 6.1, 2.1 Hz, 2H), 3.14 – 3.10 (m, 2H), 3.08 – 3.03 (m, 2H).

^{13}C NMR spectrum ($CDCl_3$)

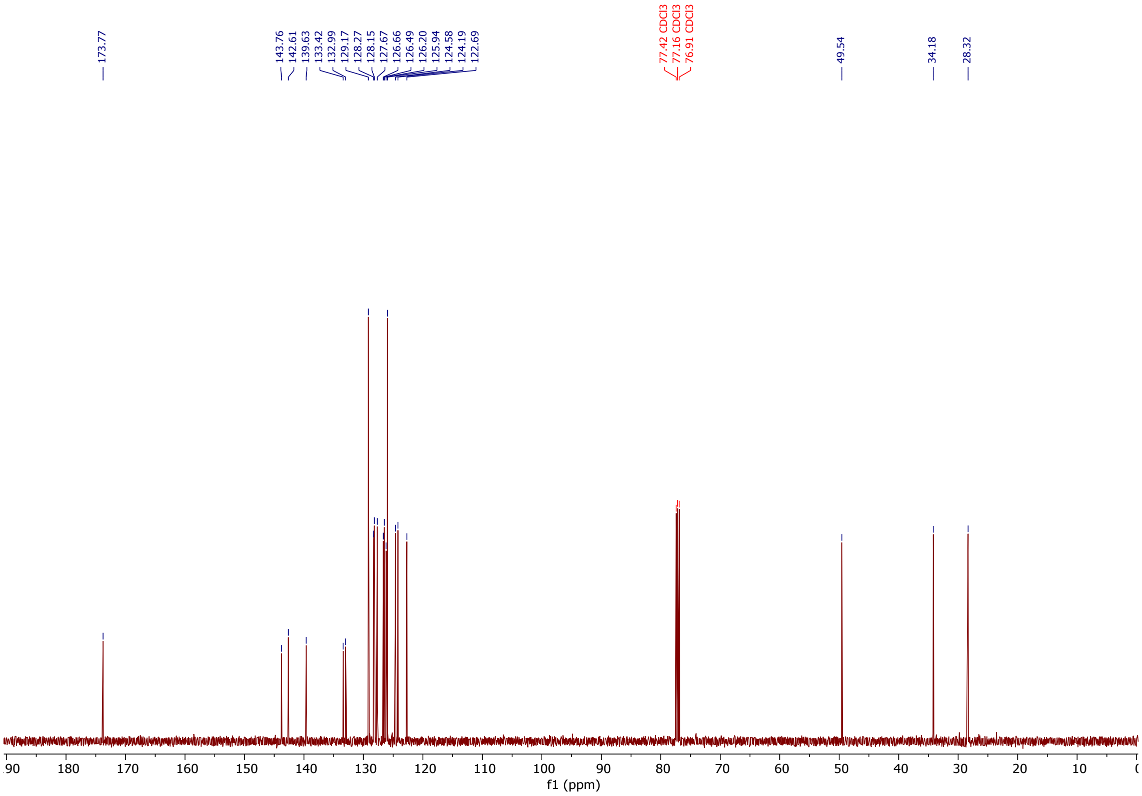

^{13}C NMR (126 MHz, $CDCl_3$) δ 173.77, 143.76, 142.61, 139.63, 133.42, 132.99, 129.17, 128.27, 128.15, 127.67, 126.66, 126.49, 126.20, 125.94, 124.58, 124.19, 122.69, 49.54, 34.18, 28.32.

IR spectrum (neat)

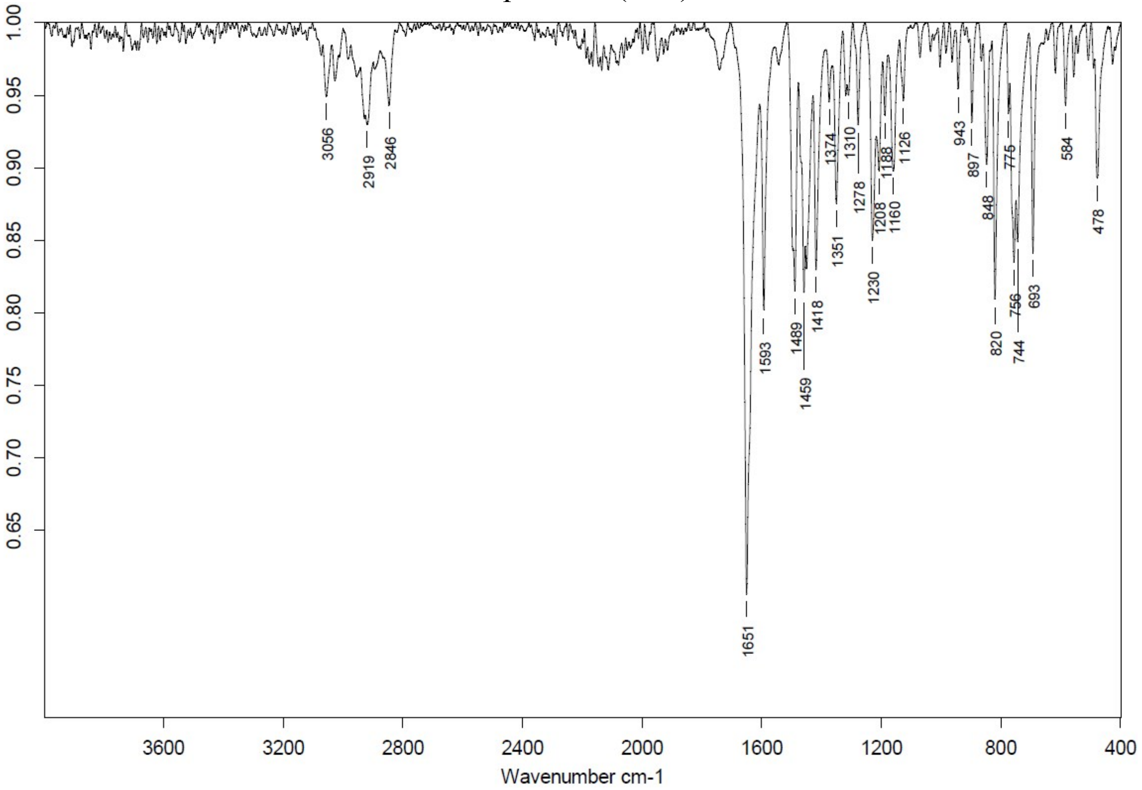

HRMS (ESI$^+$, MeOH): *m/z* calcd. 314.1539 $(M + H)^+$, found: 314.1545.

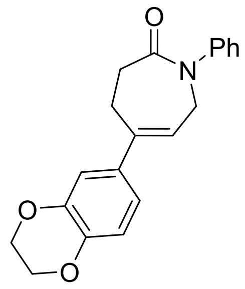

Compound 18

Scale: 0.2 mmol; *E/Z* = 98:2, isolated 54.6 mg (85% yield), white solid, Hexane : EA = 2 : 1, R_f = 0.18

^{1}H NMR spectrum ($CDCl_3$)

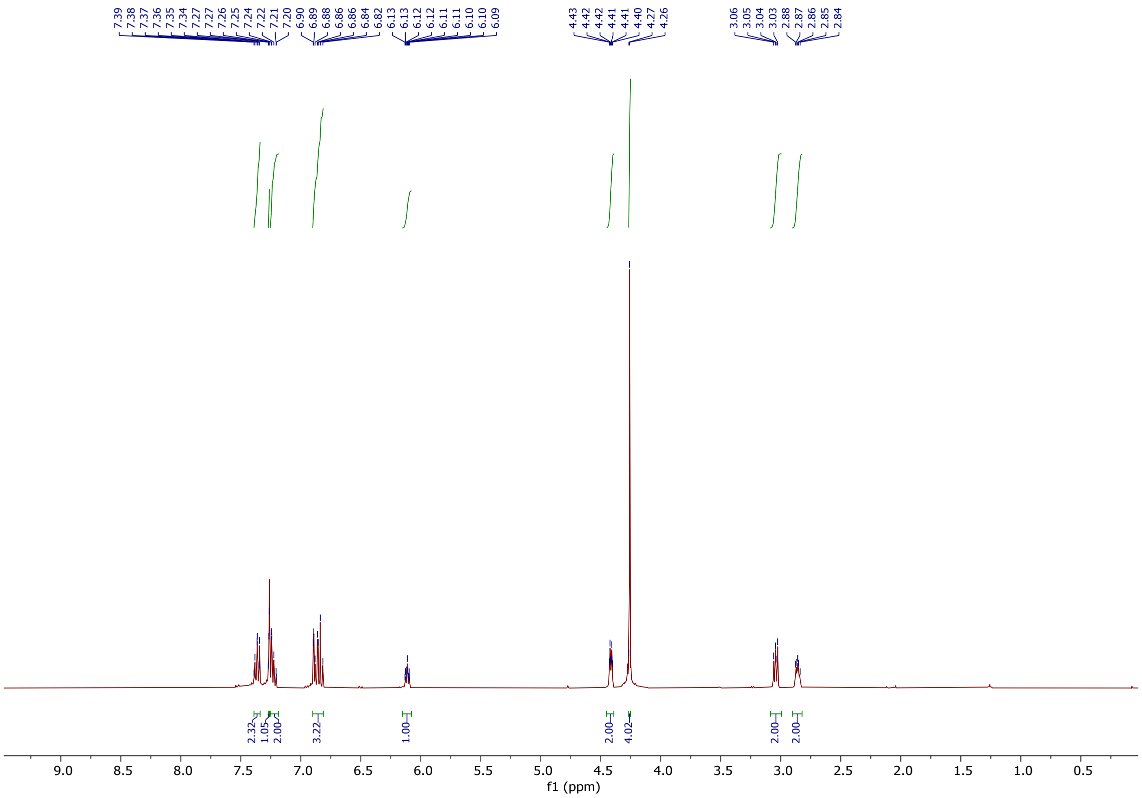

^{1}H NMR (400 MHz, $CDCl_3$) δ 7.39 – 7.34 (m, 2H), 7.27 – 7.26 (m, 1H), 7.26 – 7.19 (m, 2H), 6.90 – 6.81 (m, 3H), 6.11 (tt, *J* = 6.1, 1.9 Hz, 1H), 4.42 (dt, *J* = 6.1, 2.1 Hz, 2H), 4.26 (s, 4H), 3.08 – 3.00 (m, 2H), 2.90 – 2.82 (m, 2H).

^{13}C NMR spectrum ($CDCl_3$)

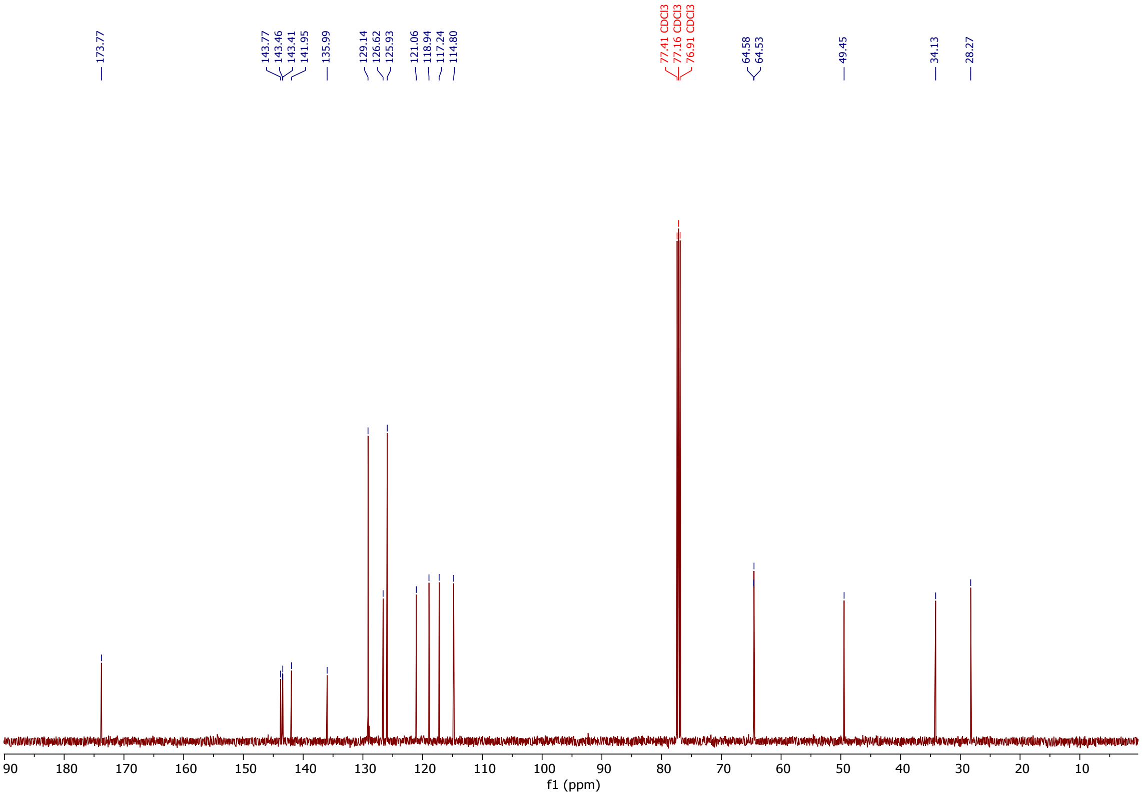

^{13}C NMR (126 MHz, $CDCl_3$) δ 173.77, 143.77, 143.46, 143.41, 141.95, 135.99, 129.14, 126.62, 125.93, 121.06, 118.94, 117.24, 114.80, 64.58, 64.53, 49.45, 34.13, 28.27.

IR spectrum (neat)

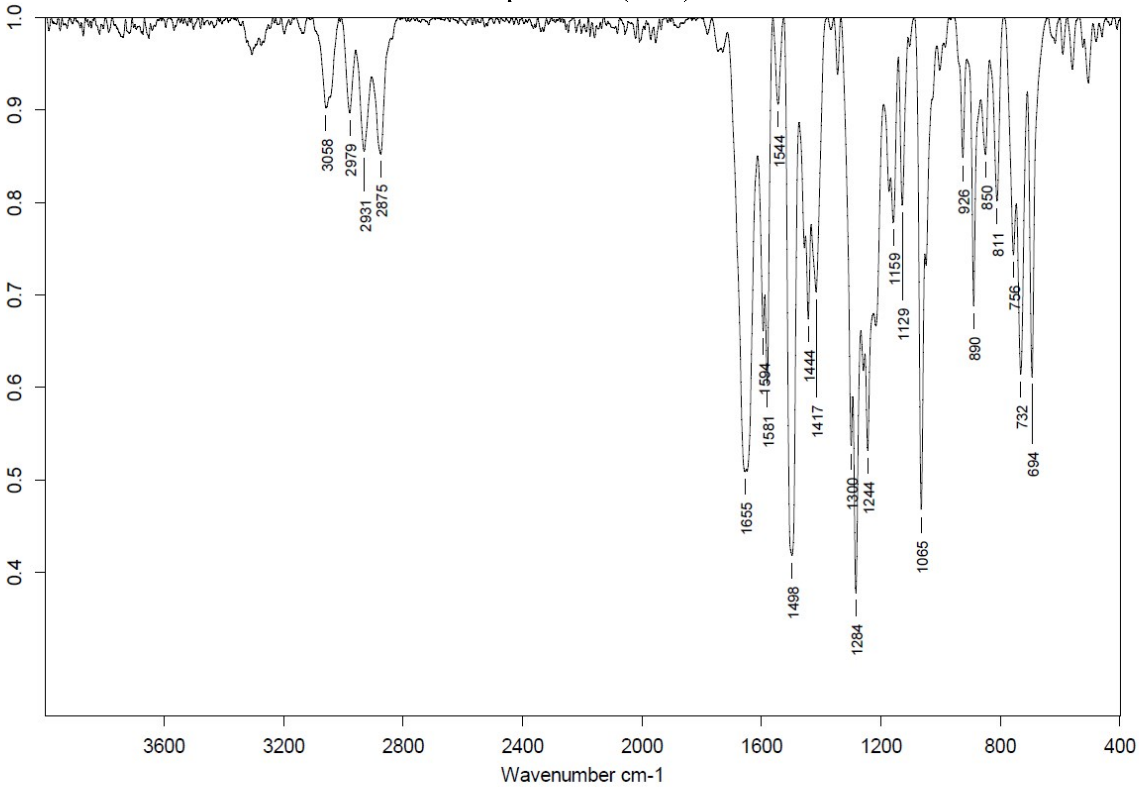

HRMS (ESI^+, MeOH): *m/z* calcd. 322.1438 $(M + H)^+$, found: 322.1443.

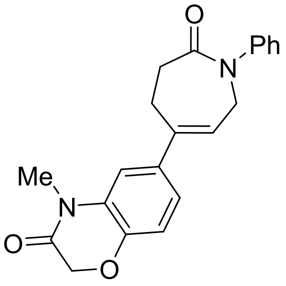

Compound 19

Scale: 0.2 mmol; *E/Z* = 93:7, isolated 50.9 mg (73% yield), white solid, Hexane : EA = 1 : 1, R_f = 0.10

^{1}H NMR spectrum ($CDCl_3$)

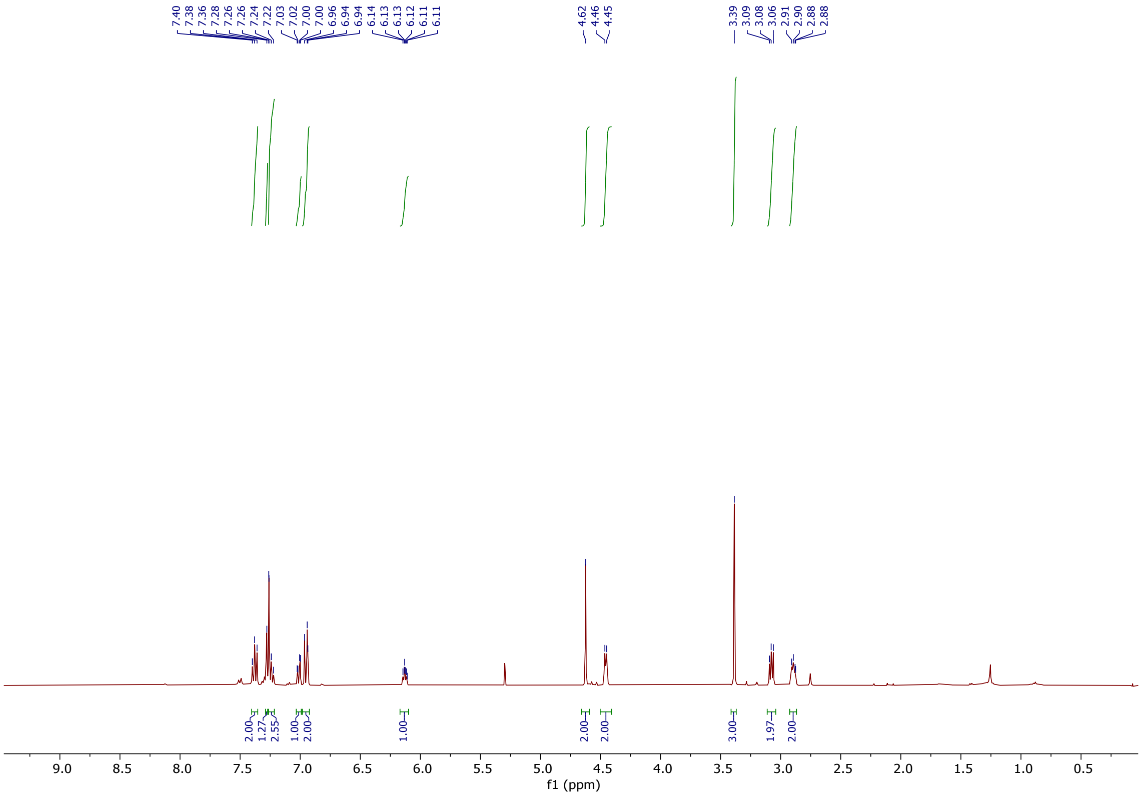

^{1}H NMR (400 MHz, $CDCl_3$) δ 7.41 – 7.34 (m, 2H), 7.28 (s, 1H), 7.26 – 7.21 (m, 2H), 7.04 – 6.99 (m, 1H), 6.98 – 6.92 (m, 2H), 6.16 – 6.09 (m, 1H), 4.62 (s, 2H), 4.46 (d, J = 6.1 Hz, 2H), 3.39 (s, 3H), 3.11 – 3.04 (m, 2H), 2.92 – 2.87 (m, 2H).

^{13}C NMR spectrum ($CDCl_3$)

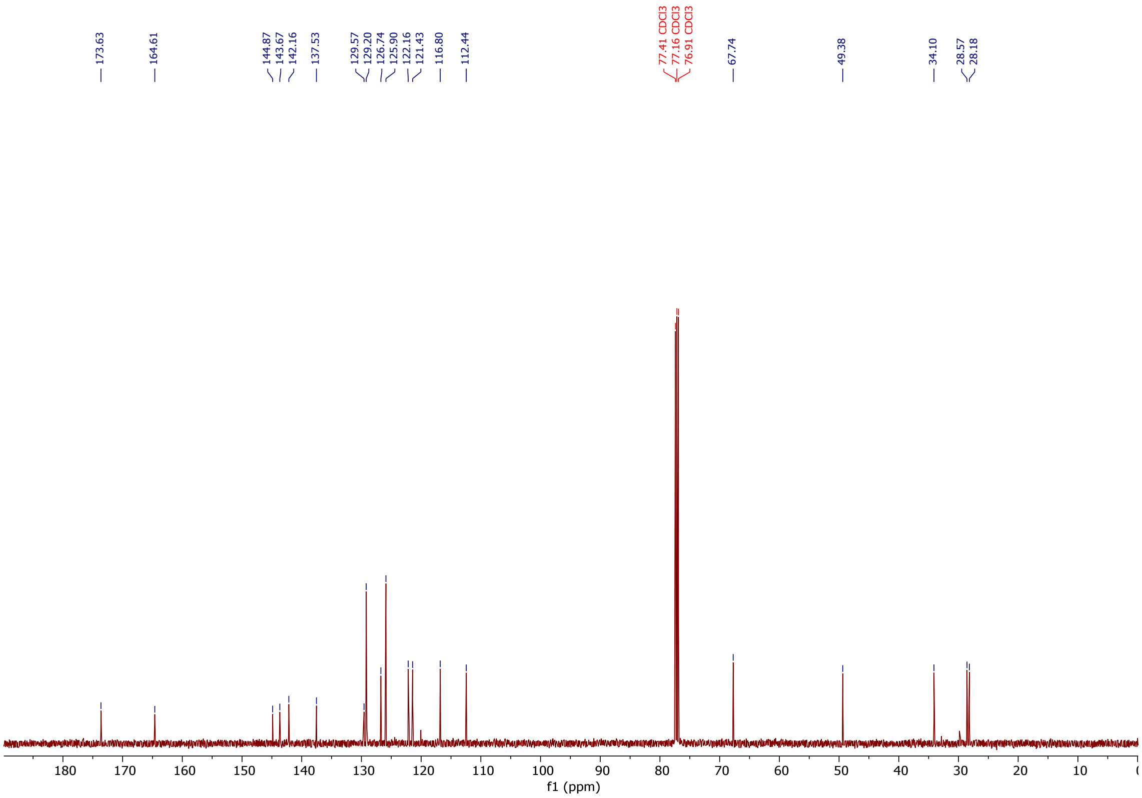

^{13}C NMR (126 MHz, $CDCl_3$) δ 173.63, 164.61, 144.87, 143.67, 142.16, 137.53, 129.57, 129.20, 126.74, 125.90, 122.16, 121.43, 116.80, 112.44, 67.74, 49.38, 34.10, 28.57, 28.18.

IR spectrum (neat)

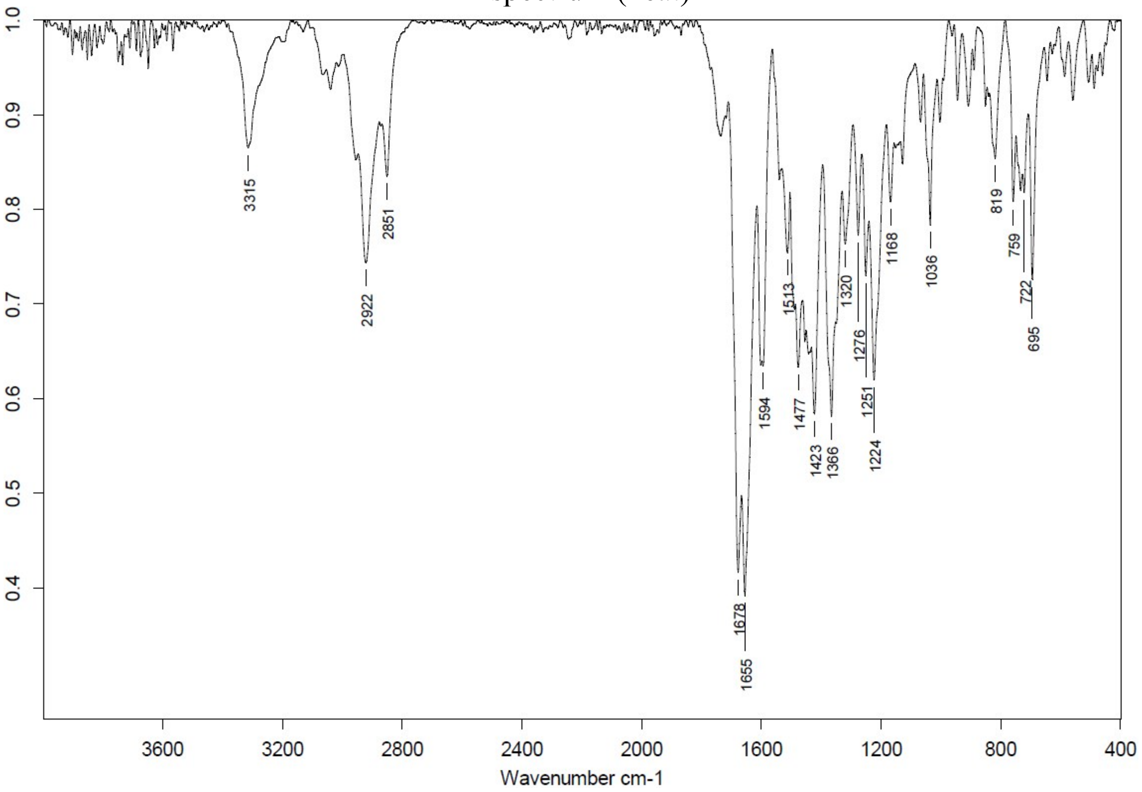

HRMS (ESI^+, MeOH): *m/z* calcd. 349.1547 $(M + H)^+$, found: 349.1549.

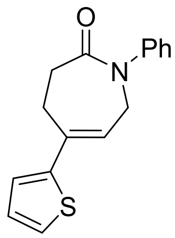

Compound 20

Scale: 0.2 mmol; *E/Z* = 78:22, isolated 36.1 mg (67% yield), yellow solid, Hexane : EA = 3 : 1, R_f = 0.25

^{1}H NMR spectrum ($CDCl_3$)

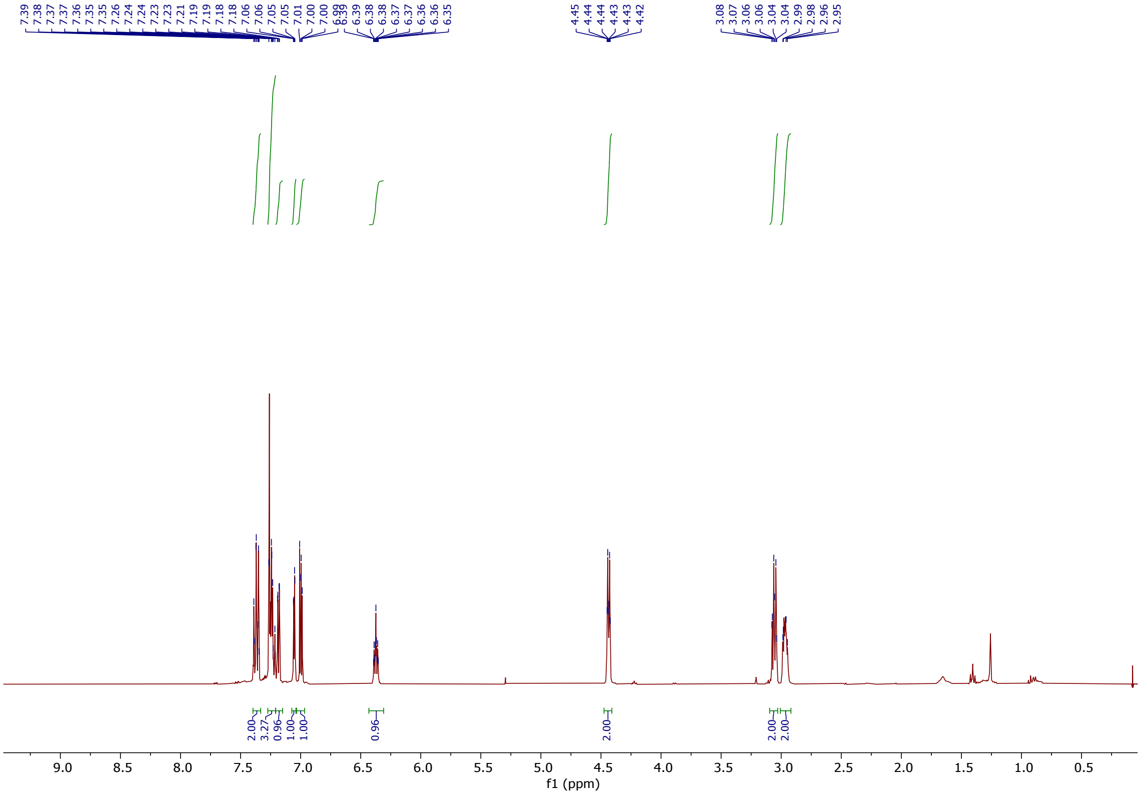

^{1}H NMR (400 MHz, $CDCl_3$) δ 7.40 – 7.33 (m, 2H), 7.27 – 7.21 (m, 3H), 7.20 – 7.15 (m, 1H), 7.07 – 7.04 (m, 1H), 7.03 – 6.96 (m, 1H), 6.37 (tt, *J* = 6.1, 1.8 Hz, 1H), 4.44 (dt, *J* = 6.1, 2.1 Hz, 2H), 3.10 – 3.03 (m, 2H), 3.01 – 2.92 (m, 2H).

^{13}C NMR spectrum ($CDCl_3$)

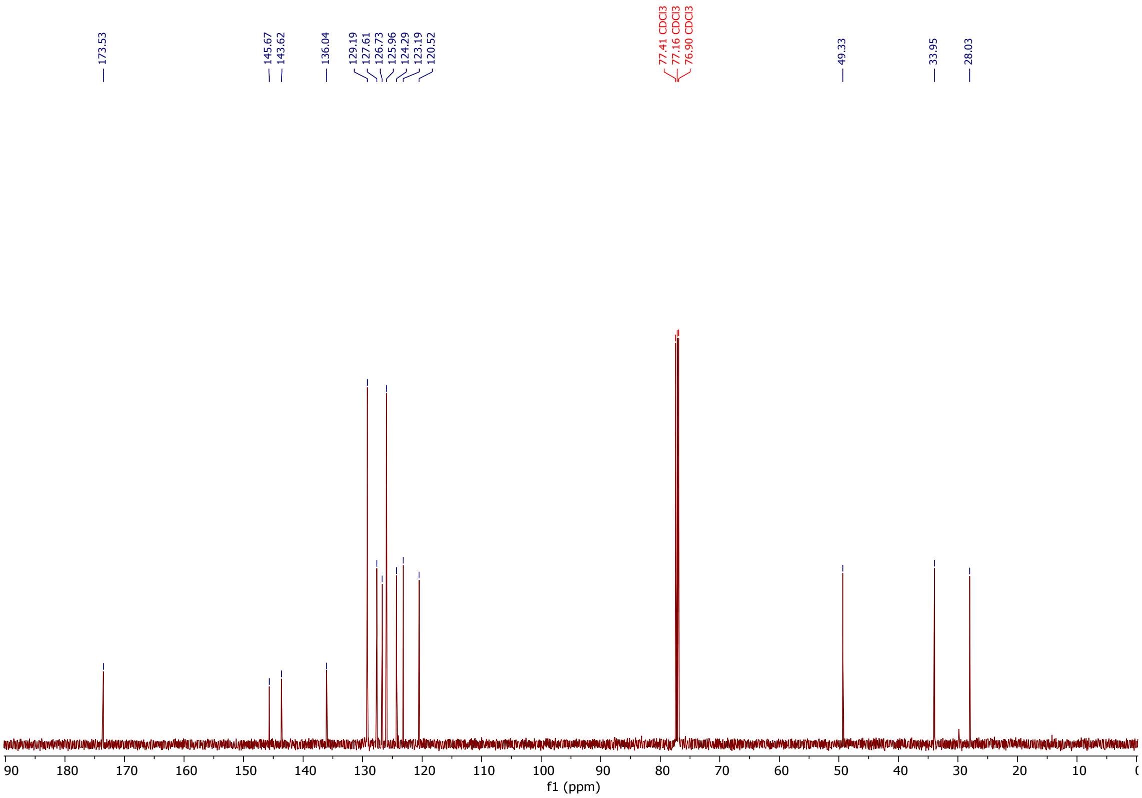

^{13}C NMR (126 MHz, $CDCl_3$) δ 173.53, 145.67, 143.62, 136.04, 129.19, 127.61, 126.73, 125.96, 124.29, 123.19, 120.52, 49.33, 33.95, 28.03.

IR spectrum (neat)

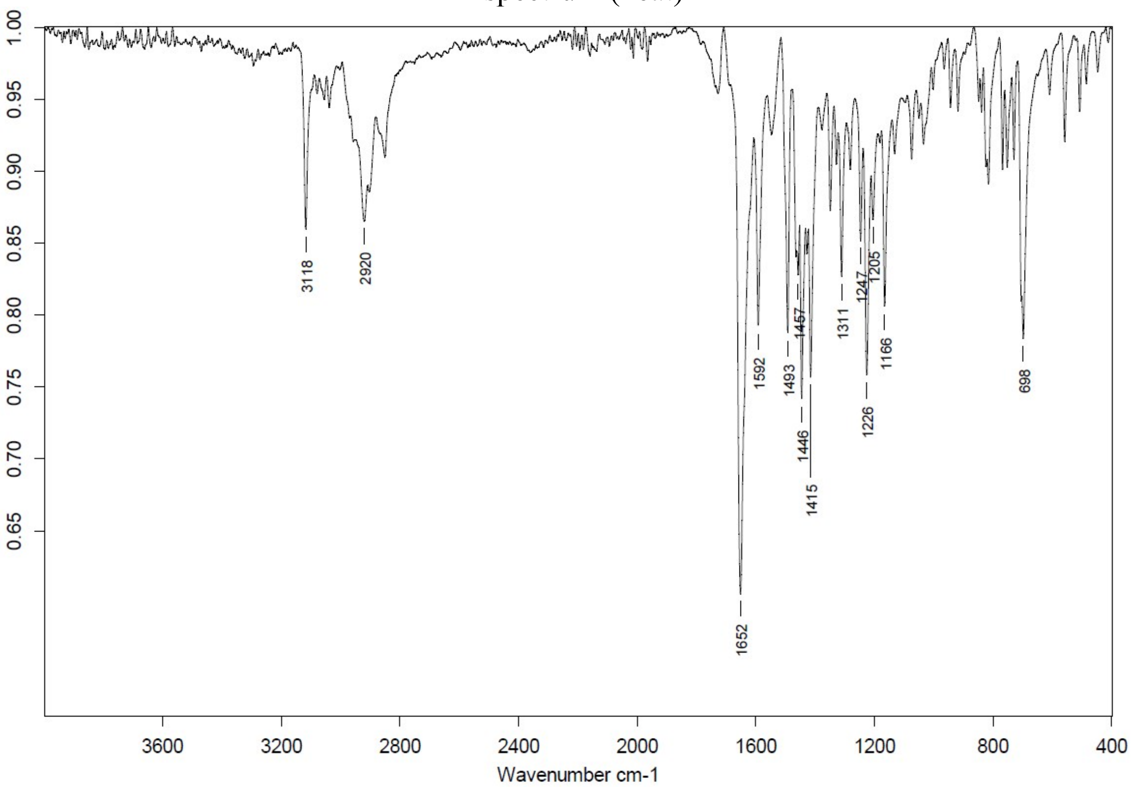

HRMS (ESI$^+$, MeOH): *m/z* calcd. 270.0947 $(M + H)^+$, found: 270.0943.

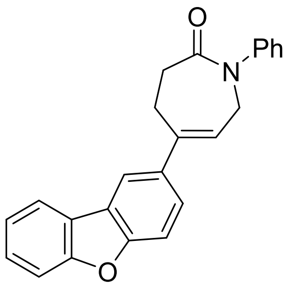

Compound 21

Scale: 0.2 mmol; *E/Z* = 99:1, isolated 56.5 mg (80% yield), yellow solid, Hexane : EA = 2 : 1, R_f = 0.15

^{1}H NMR spectrum ($CDCl_3$)

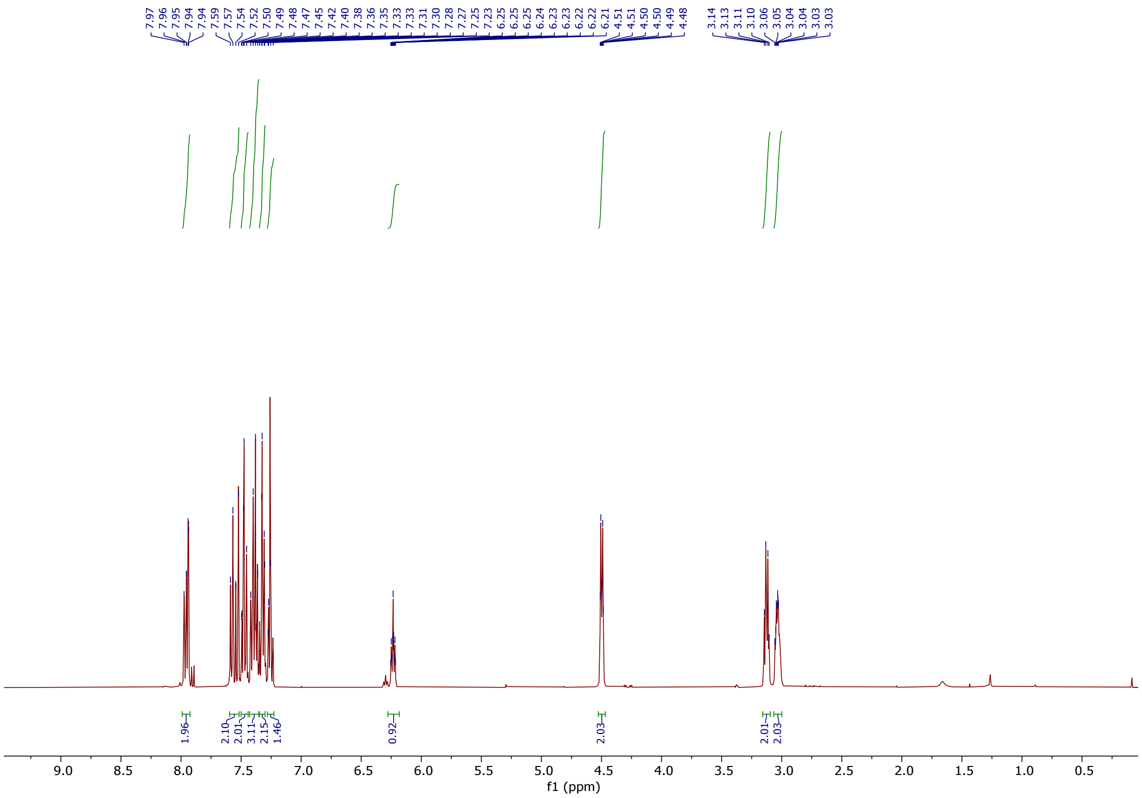

^{1}H NMR (400 MHz, $CDCl_3$) δ 7.99 – 7.93 (m, 2H), 7.60 – 7.52 (m, 2H), 7.50 – 7.44 (m, 2H), 7.43 – 7.35 (m, 3H), 7.35 – 7.30 (m, 2H), 7.28 – 7.23 (m, 1H), 6.23 (tt, *J* = 6.1, 1.9 Hz, 1H), 4.50 (dt, *J* = 6.1, 2.2 Hz, 2H), 3.16 – 3.09 (m, 2H), 3.06 – 2.98 (m, 2H).

^{13}C NMR spectrum ($CDCl_3$)

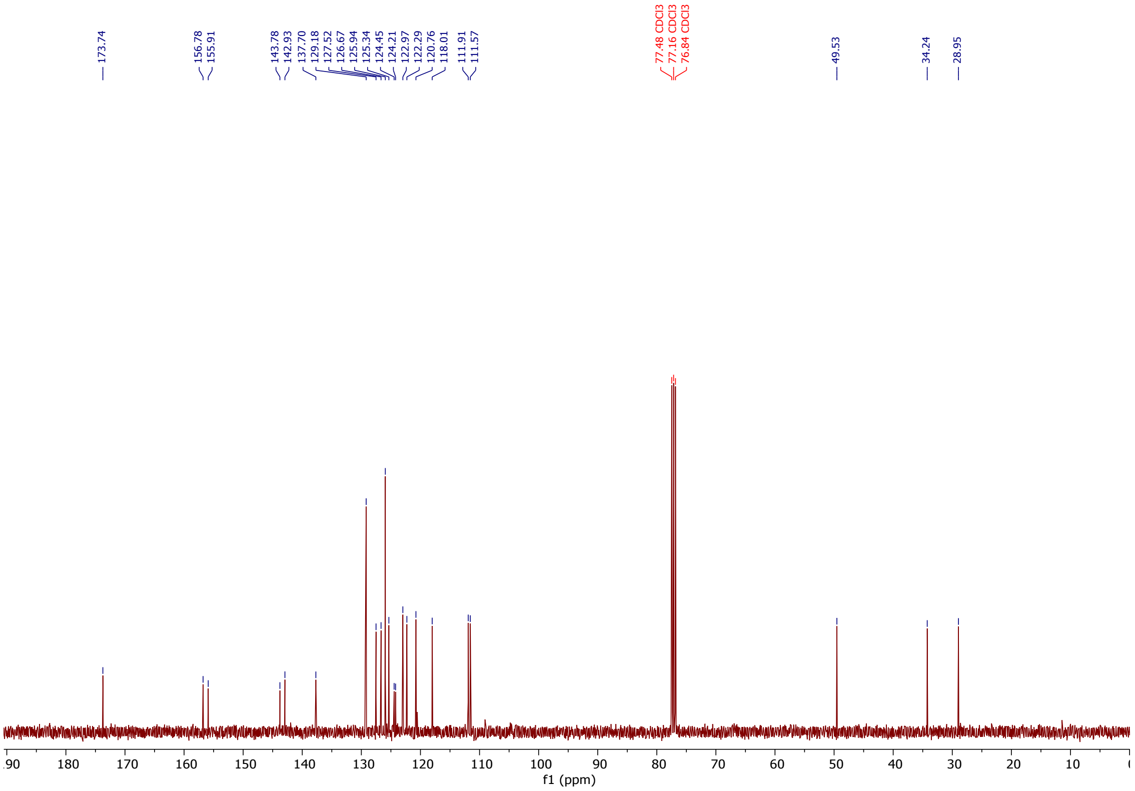

^{13}C NMR (101 MHz, $CDCl_3$) δ 173.74, 156.78, 155.91, 143.78, 142.93, 137.70, 129.18, 127.52, 126.67, 125.94, 125.34, 124.45, 124.21, 122.97, 122.29, 120.76, 118.01, 111.91, 111.57, 49.53, 34.24, 28.95.

IR spectrum (neat)

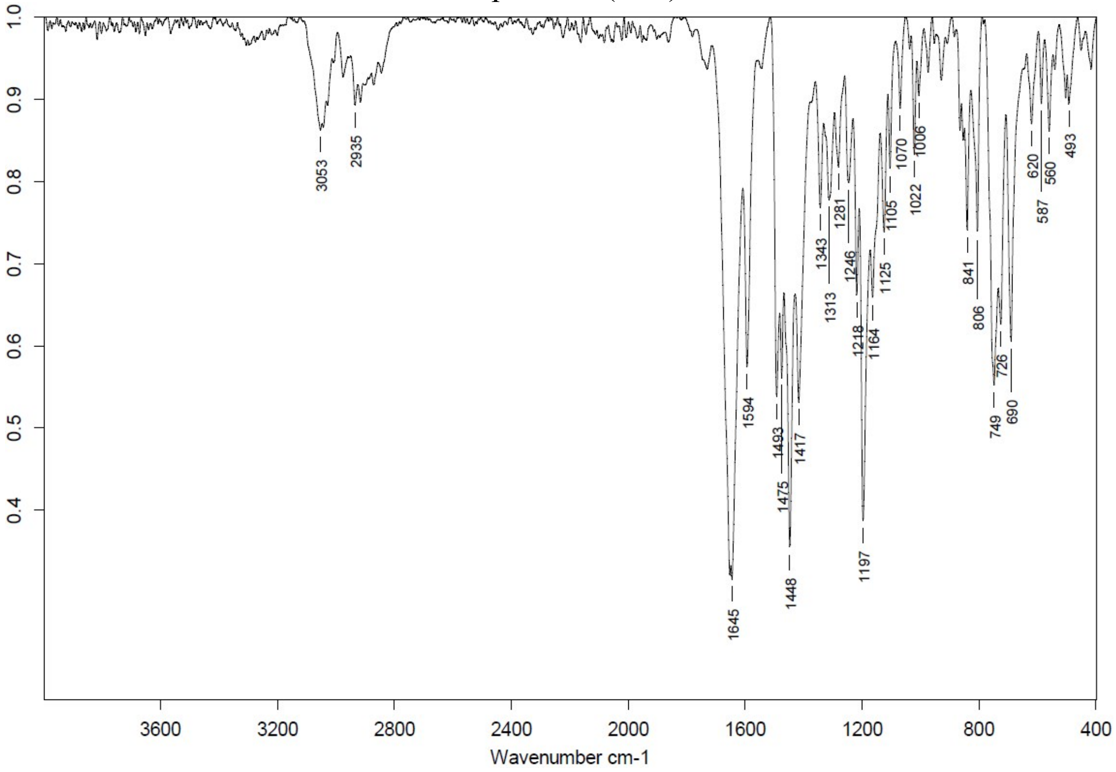

HRMS (ESI$^+$, MeOH): *m/z* calcd. 376.1308 (M + Na)$^+$, found: 376.1305.

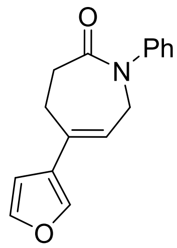

Compound 22

Scale: 0.2 mmol; *E/Z* = 87:13, isolated 36.5 mg (72% yield), yellow oil, Hexane : EA = 4 : 1, R_f = 0.15

^{1}H NMR spectrum ($CDCl_3$)

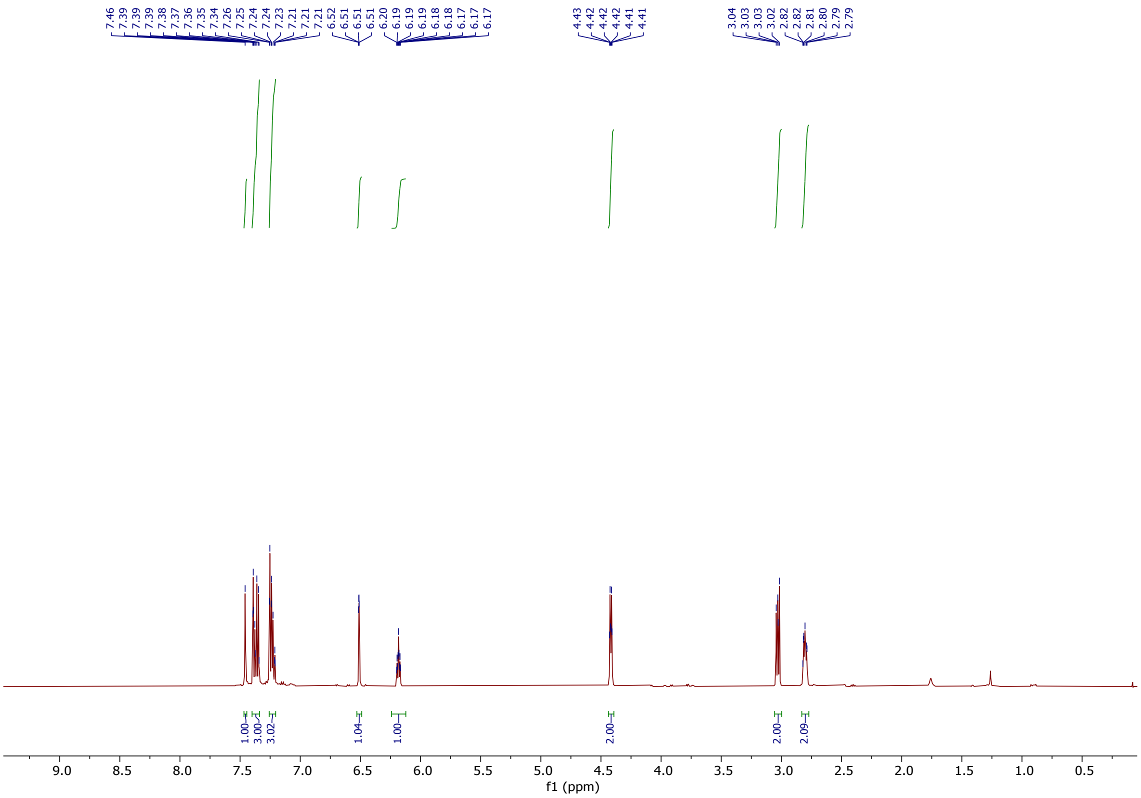

^{1}H NMR (500 MHz, $CDCl_3$) δ 7.46 (s, 1H), 7.40 – 7.34 (m, 3H), 7.26 – 7.20 (m, 3H), 6.53 – 6.48 (m, 1H), 6.18 (tt, *J* = 6.1, 1.8 Hz, 1H), 4.42 (dt, *J* = 6.1, 2.0 Hz, 2H), 3.06 – 3.00 (m, 2H), 2.83 – 2.77 (m, 2H).

^{13}C NMR spectrum ($CDCl_3$)

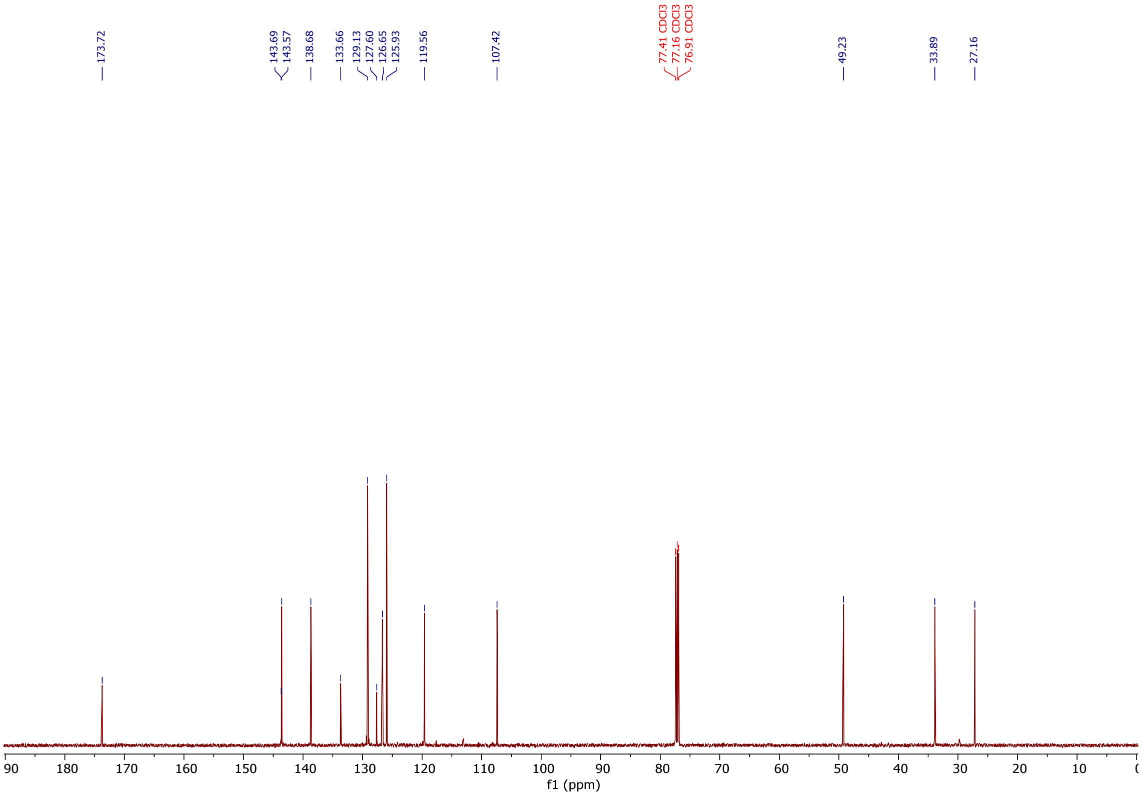

^{13}C NMR (126 MHz, $CDCl_3$) δ 173.72, 143.69, 143.57, 138.68, 133.66, 129.13, 127.60, 126.65, 125.93, 119.56, 107.42, 49.23, 33.89, 27.16.

IR spectrum (neat)

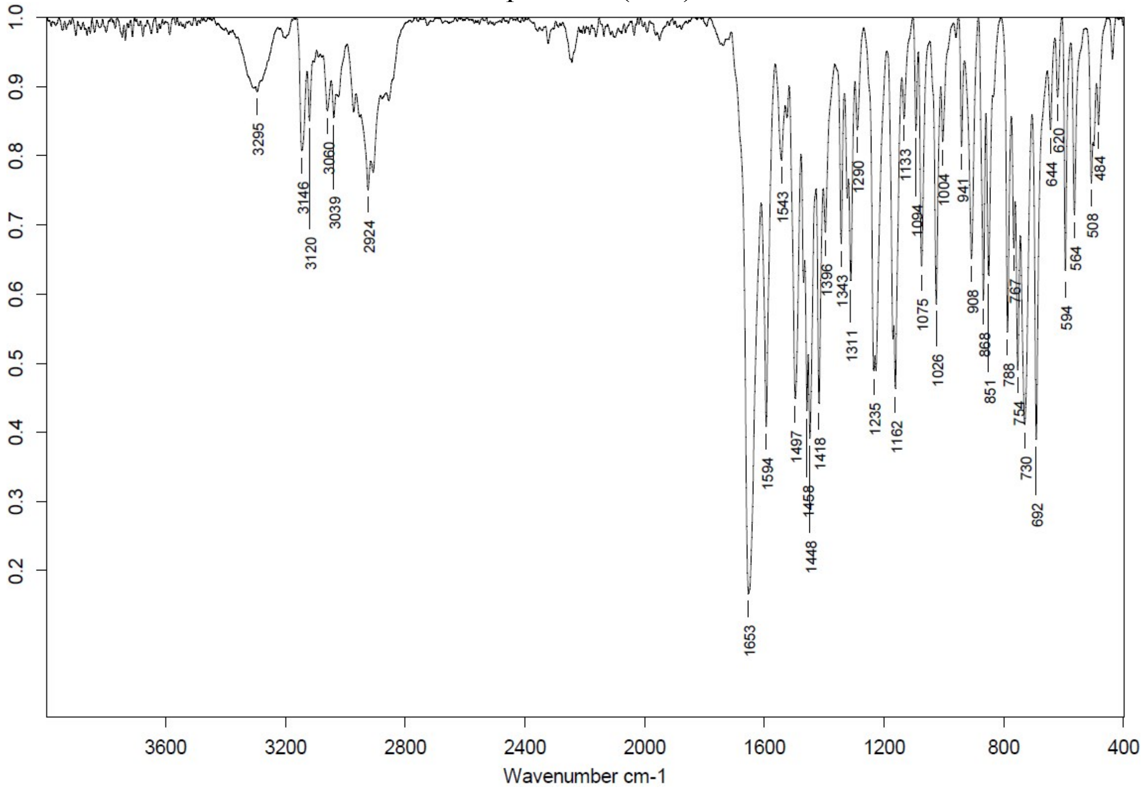

HRMS (ESI$^+$, MeOH): *m/z* calcd. 276.0995 $(M + Na)^+$, found: 276.0996.

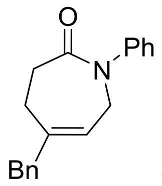

Compound 23

Scale: 0.2 mmol; *E/Z*>99:1, isolated 41.6 mg (75% yield), slight yellow solid, Hexane : EA = 2 : 1, R_f = 0.2

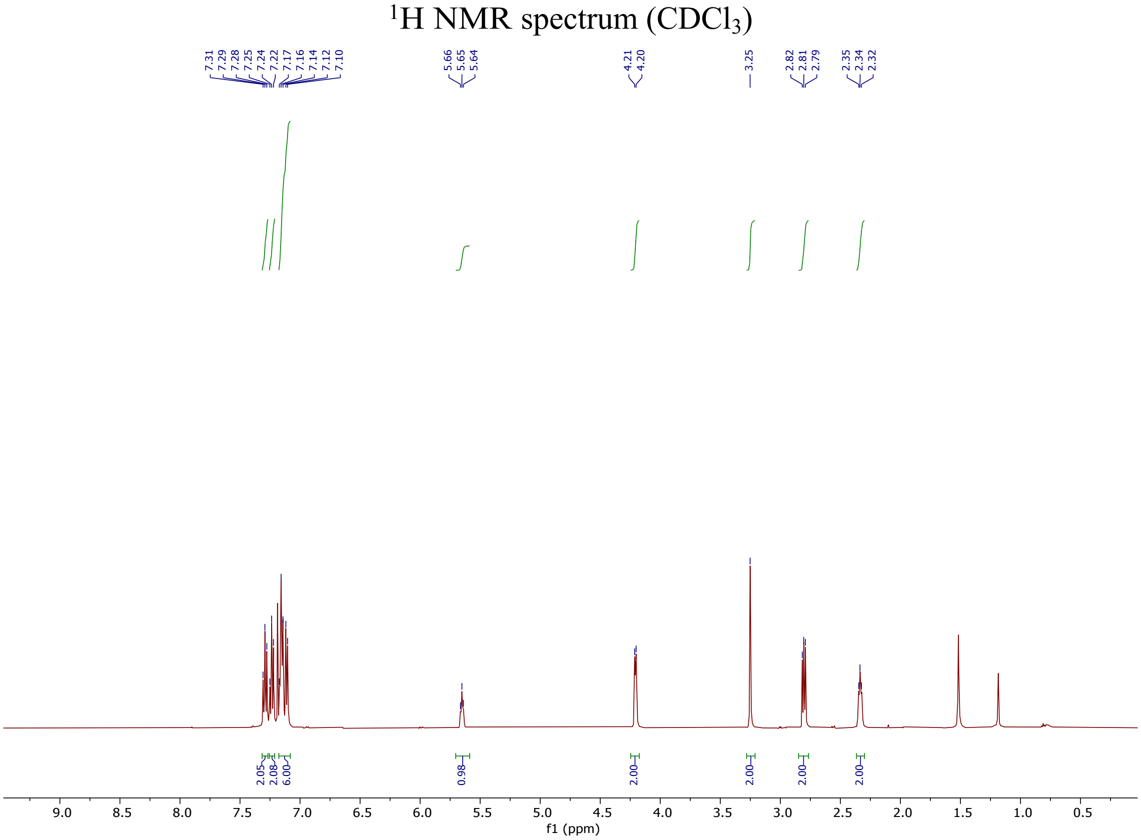

^{1}H NMR (500 MHz, $CDCl_3$) δ 7.32 – 7.27 (m, 2H), 7.25 – 7.21 (m, 2H), 7.18 – 7.08 (m, 6H), 5.65 (t, *J* = 6.1 Hz, 1H), 4.21 (d, *J* = 5.9 Hz, 2H), 3.25 (s, 2H), 2.85 – 2.76 (m, 2H), 2.36 – 2.29 (m, 2H).

^{13}C NMR spectrum ($CDCl_3$)

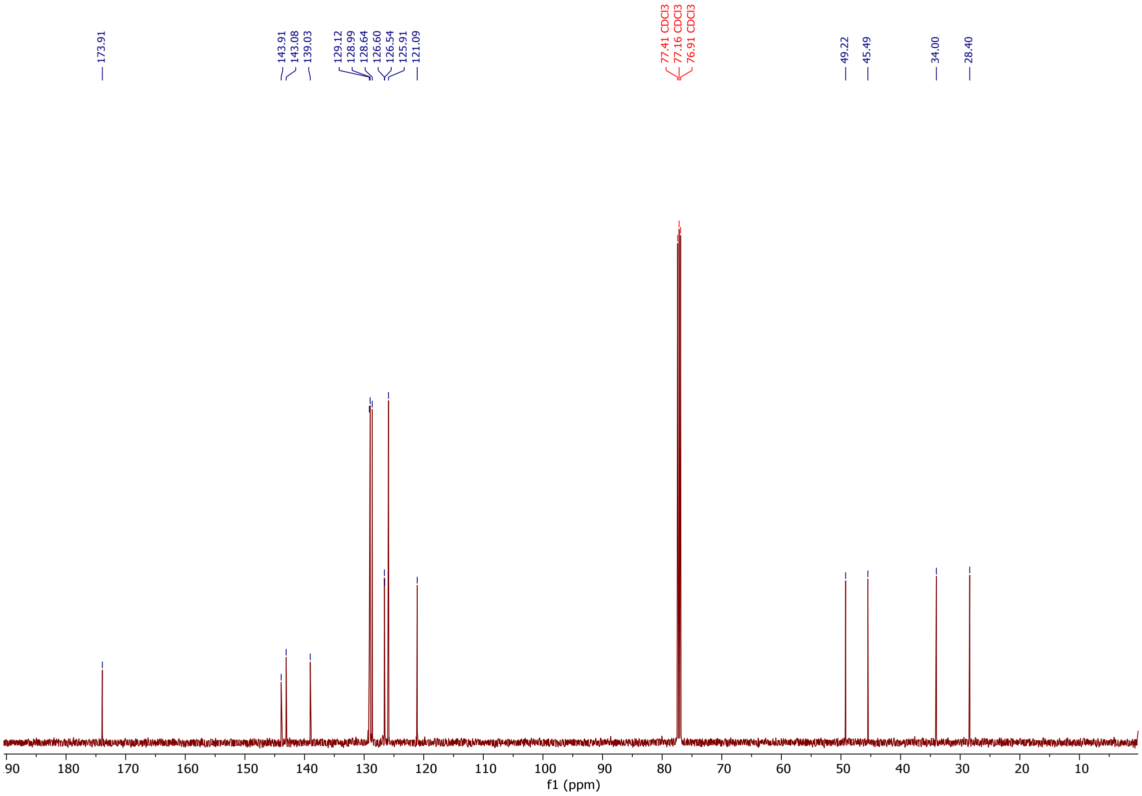

^{13}C NMR (126 MHz, $CDCl_3$) δ 173.91, 143.91, 143.08, 139.03, 129.12, 128.99, 128.64, 126.60, 126.54, 125.91, 121.09, 49.22, 45.49, 34.00, 28.40.

IR spectrum (neat)

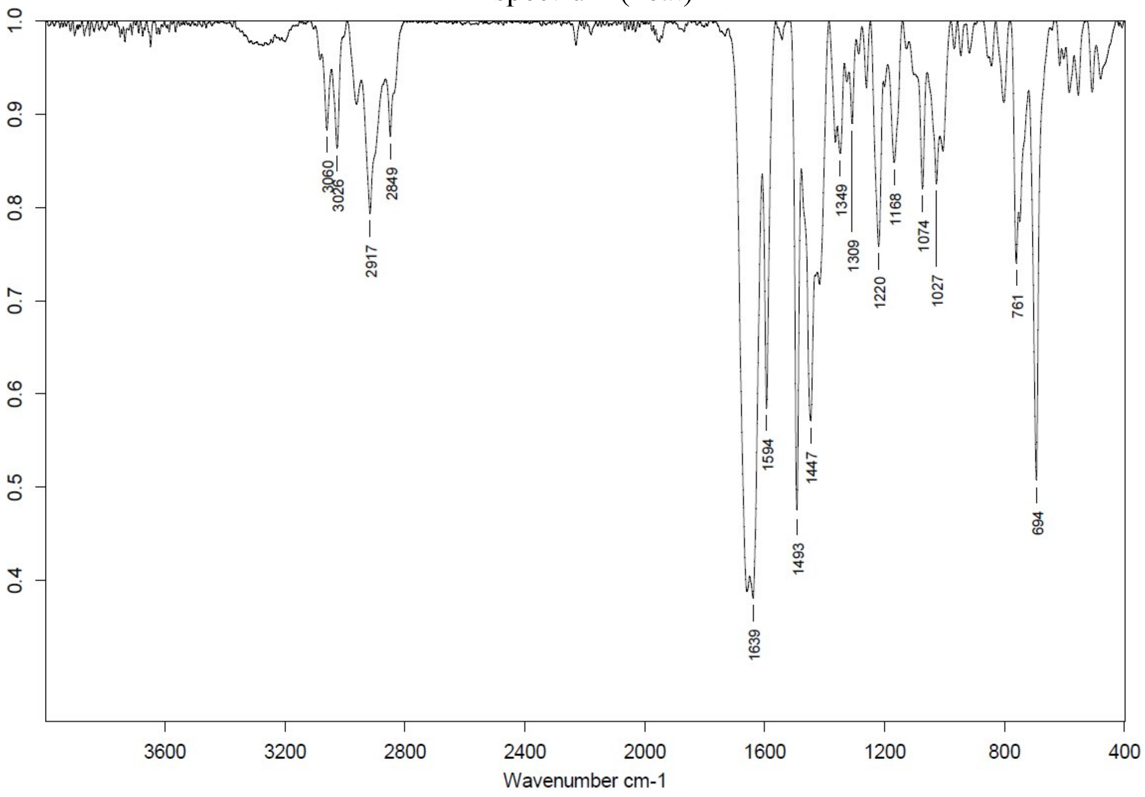

HRMS (ESI$^+$, MeOH): *m/z* calcd. 278.1539 $(M + H)^+$, found: 278.1542.

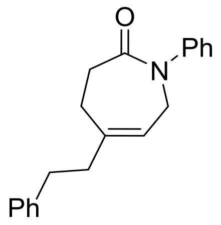

Compound 24

Scale: 0.2 mmol; *E/Z* = 26:74, isolated 29.7 mg (51% yield), yellow oil, Hexane : EA = 2 : 1, R_f = 0.20

^{1}H NMR spectrum ($CDCl_3$)

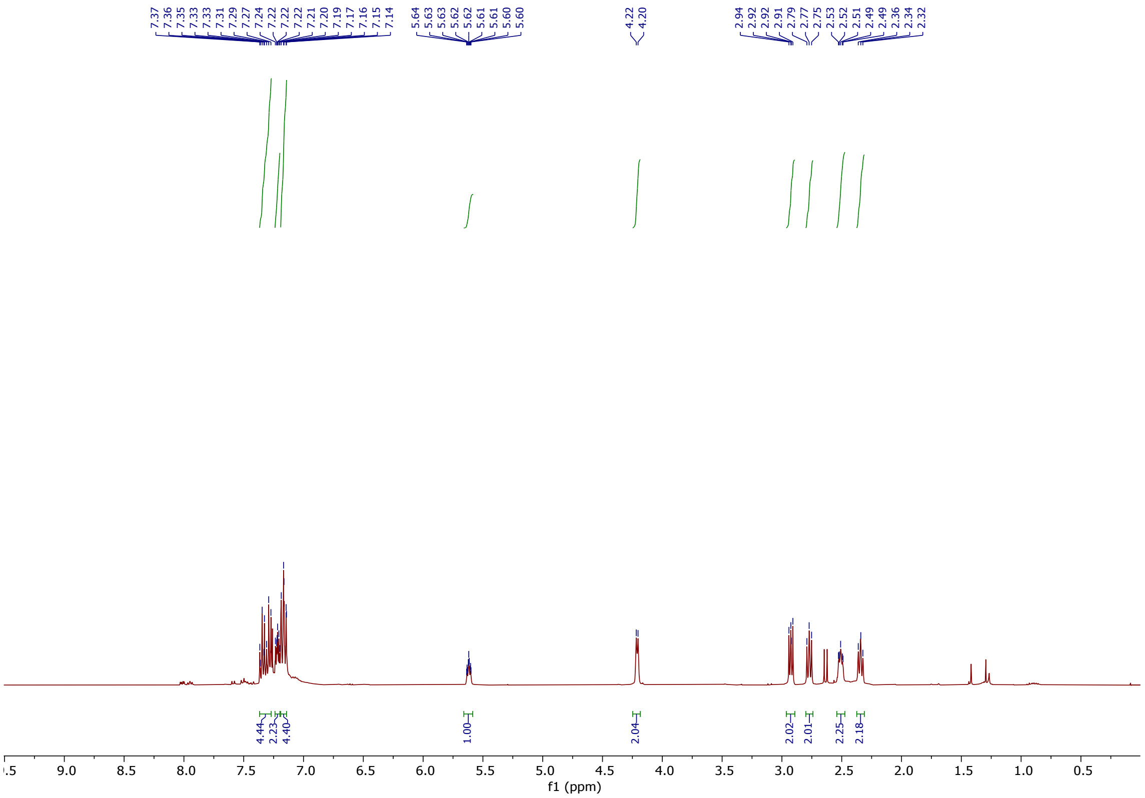

^{1}H NMR (400 MHz, $CDCl_3$) δ 7.37 – 7.27 (m, 4H), 7.24 – 7.20 (m, 2H), 7.19 – 7.14 (m, 4H), 5.62 (tt, J = 6.0, 1.5 Hz, 1H), 4.21 (d, J = 5.9 Hz, 2H), 2.96 – 2.89 (m, 2H), 2.80 – 2.74 (m, 2H), 2.54 – 2.47 (m, 2H), 2.37 – 2.31 (m, 2H).

^{13}C NMR spectrum ($CDCl_3$)

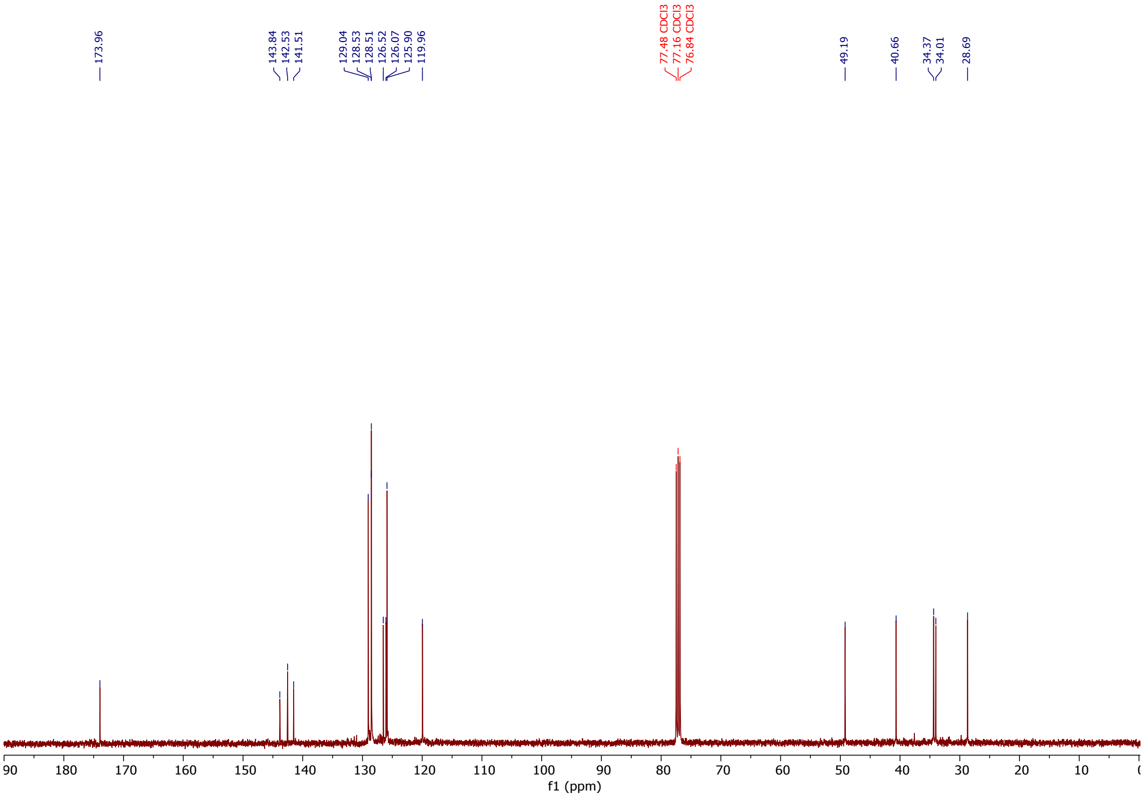

^{13}C NMR (101 MHz, $CDCl_3$) δ 173.96, 143.84, 142.53, 141.51, 129.04, 128.53, 128.51, 126.52, 126.07, 125.90, 119.96, 49.19, 40.66, 34.37, 34.01, 28.69.

IR spectrum (neat)

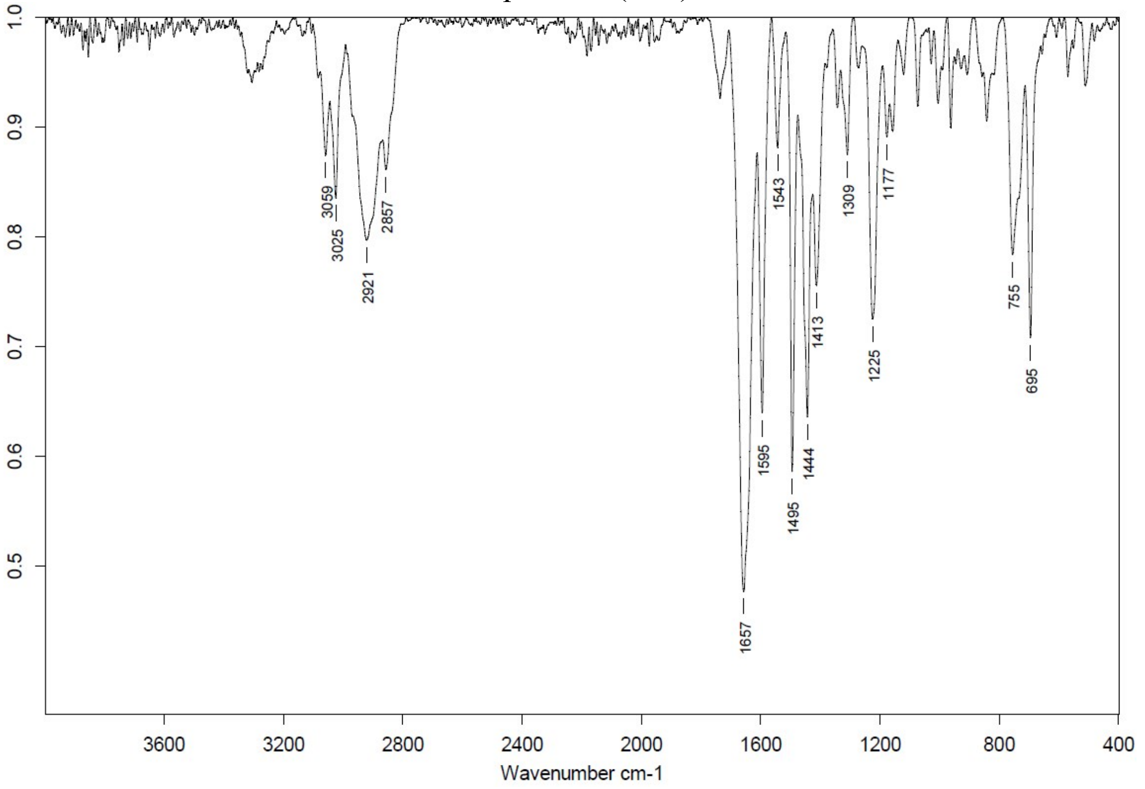

HRMS (ESI+, MeOH): *m/z* calcd. 292.1696 (M + H)+, found: 292.1693.

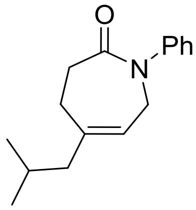

Compound 25

Scale: 0.2 mmol; *E/Z* = 62:38, isolated 26.3 mg (54% yield), yellow oil, Hexane : EA = 2 : 1, R_f = 0.25

^{1}H NMR spectrum ($CDCl_3$)

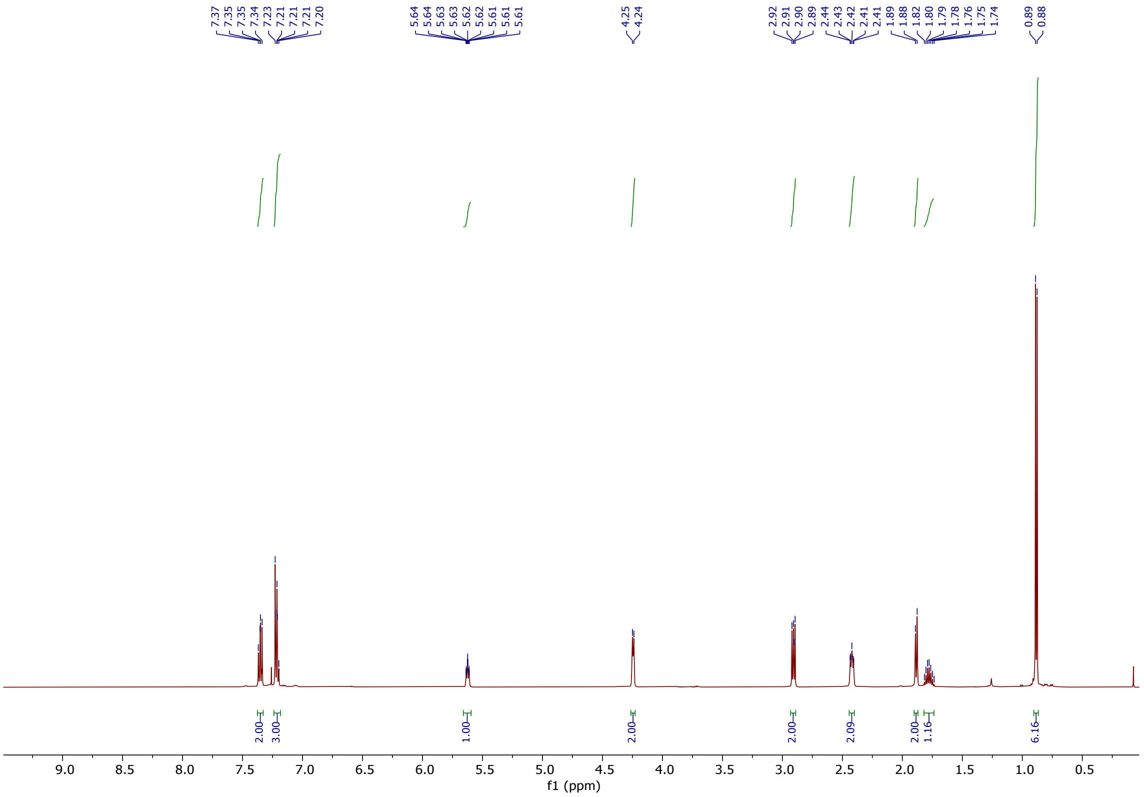

^{1}H NMR (500 MHz, $CDCl_3$) δ 7.38 – 7.33 (m, 2H), 7.24 – 7.18 (m, 3H), 5.62 (tt, *J* = 6.0, 1.0 Hz, 1H), 4.24 (d, *J* = 5.9 Hz, 2H), 2.93 – 2.89 (m, 2H), 2.44 – 2.40 (m, 2H), 1.88 (d, *J* = 7.4 Hz, 2H), 1.82 – 1.74 (m, 1H), 0.88 (d, *J* = 6.5 Hz, 6H).

^{13}C NMR spectrum ($CDCl_3$)

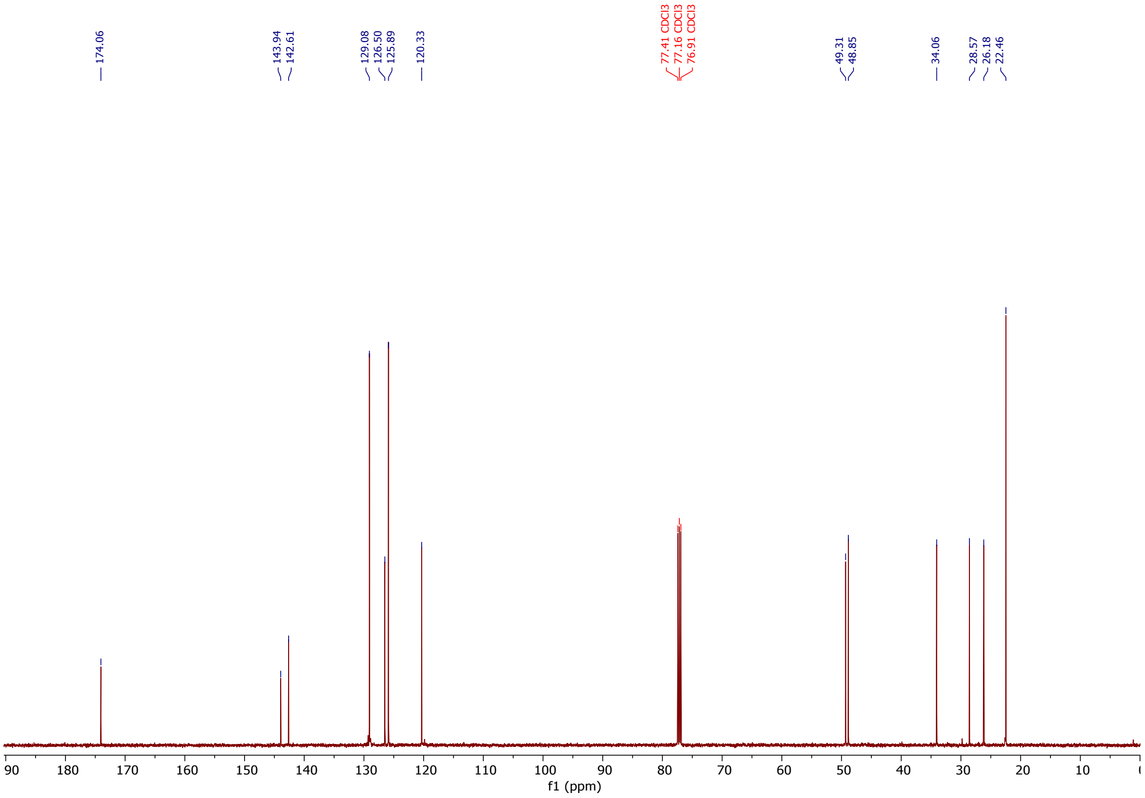

^{13}C NMR (126 MHz, $CDCl_3$) δ 174.06, 143.94, 142.61, 129.08, 126.50, 125.89, 120.33, 49.31, 48.85, 34.06, 28.57, 26.18, 22.46.

IR spectrum (neat)

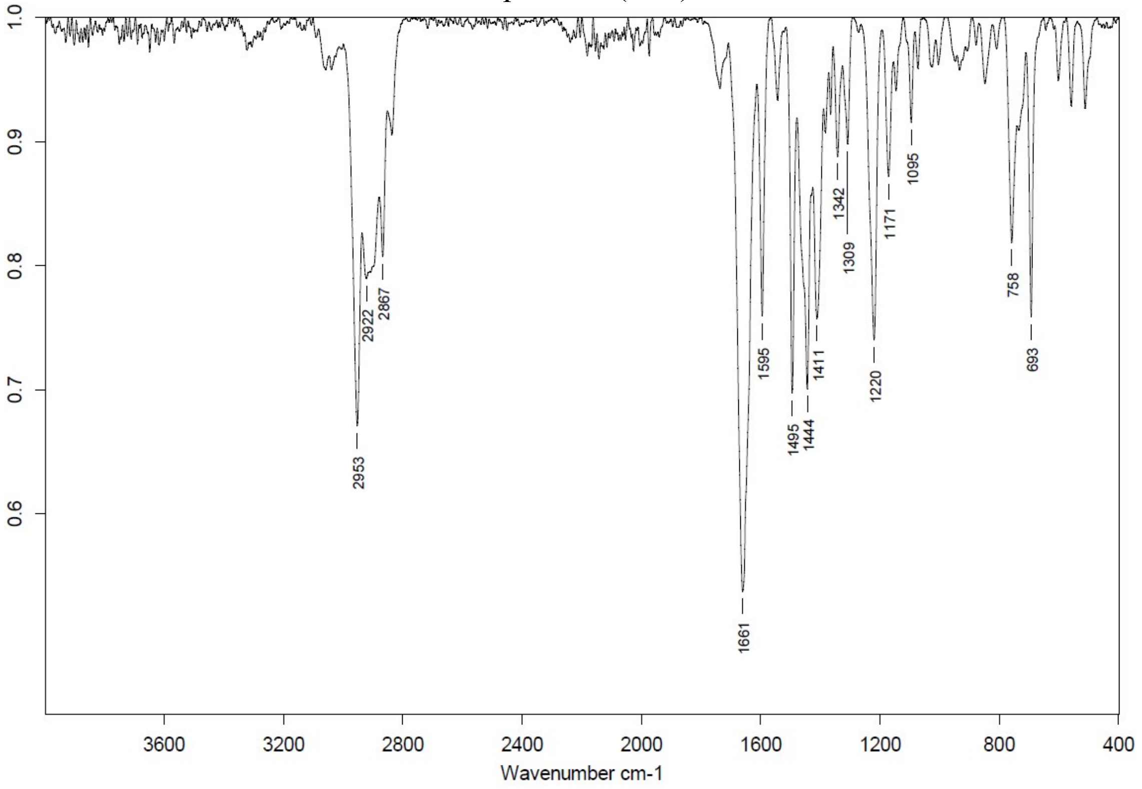

HRMS (ESI$^+$, MeOH): *m/z* calcd. 266.1515 (M + Na)$^+$, found: 266.1519.

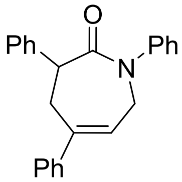

Compound 26

Scale: 0.2 mmol; *E/Z* = 97:3, isolated 55.0 mg (81% yield), yellow oil, Hexane : EA = 6 : 1, R_f = 0.15

^{1}H NMR spectrum ($CDCl_3$)

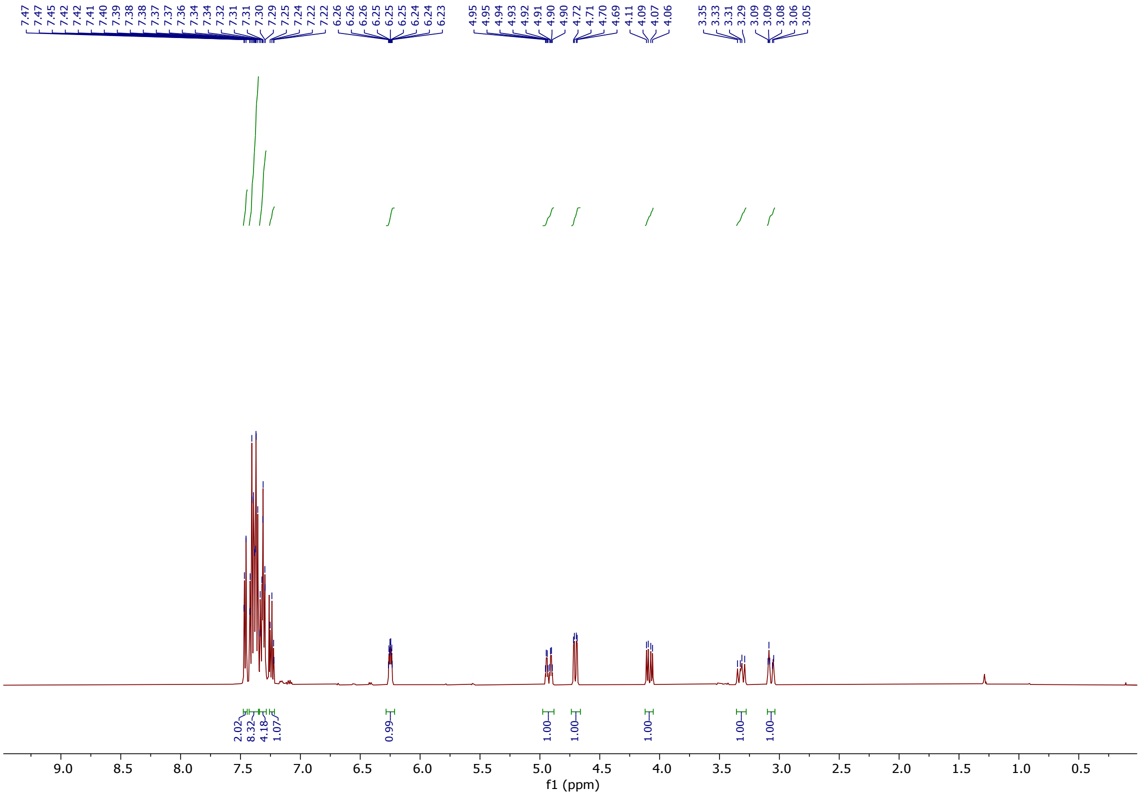

^{1}H NMR (500 MHz, $CDCl_3$) δ 7.48 – 7.44 (m, 2H), 7.43 – 7.35 (m, 8H), 7.34 – 7.28 (m, 4H), 7.24 (t, *J* = 7.3 Hz, 1H), 6.25 (ddt, *J* = 7.8, 4.1, 1.9 Hz, 1H), 4.98 – 4.88 (m, 1H), 4.70 (dd, *J* = 11.7, 3.6 Hz, 1H), 4.08 (dd, *J* = 18.0, 7.5 Hz, 1H), 3.35 – 3.27 (m, 1H), 3.10 – 3.04 (m, 1H).

^{13}C NMR spectrum ($CDCl_3$)

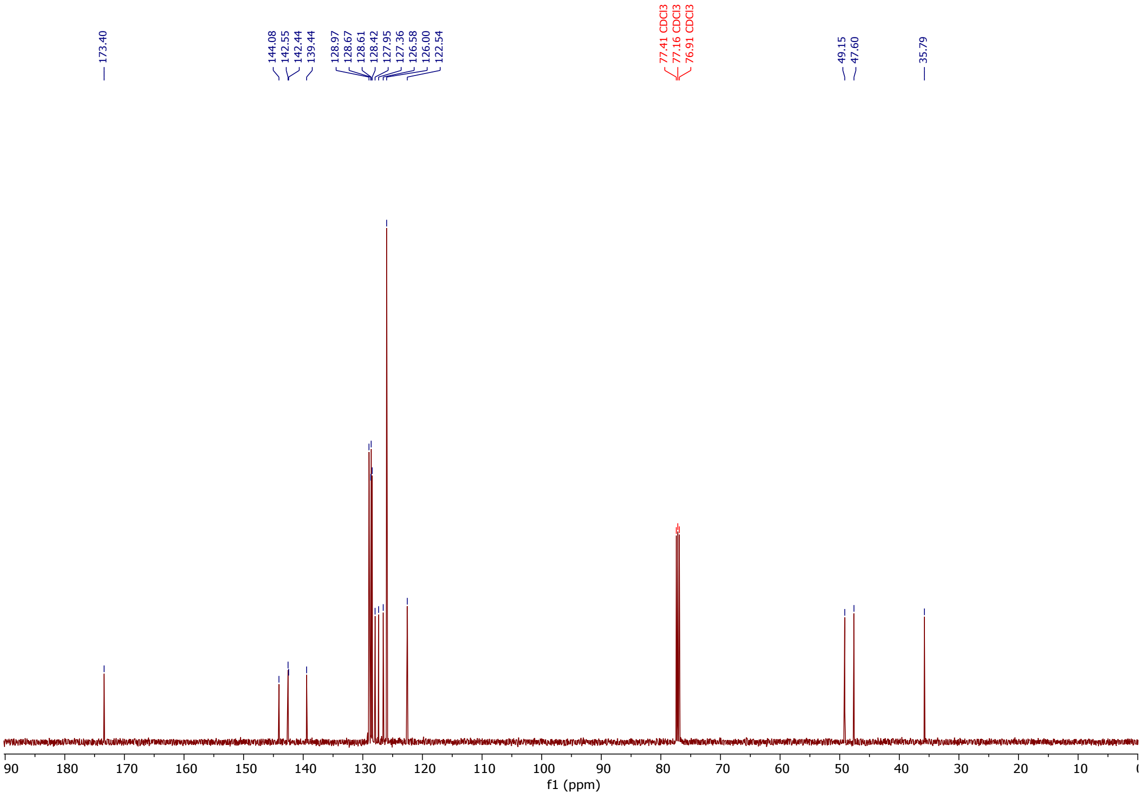

^{13}C NMR (126 MHz, $CDCl_3$) δ 173.40, 144.08, 142.55, 142.44, 139.44, 128.97, 128.67, 128.61, 128.42, 127.95, 127.36, 126.58, 126.00, 122.54, 49.15, 47.60, 35.79.

IR spectrum (neat)

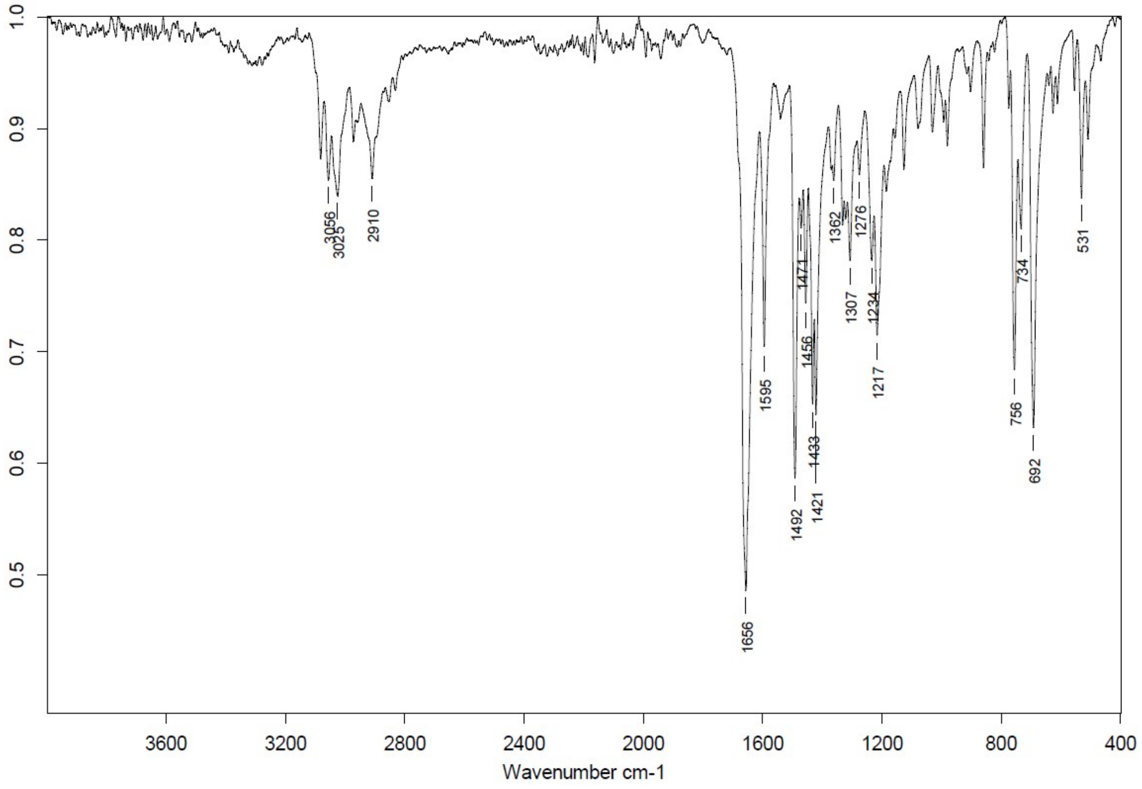

HRMS (ESI$^+$, MeOH): *m/z* calcd. 340.1696 $(M + H)^+$, found: 340.1699.

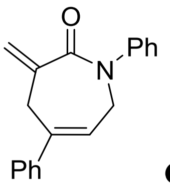

Compound 27

Scale: 0.2 mmol; *E/Z*>99:1, isolated 28.6 mg (52% yield), yellow oil, Hexane : EA = 5 : 1, R_f = 0.15

^{1}H NMR spectrum (CDCl$_3$)

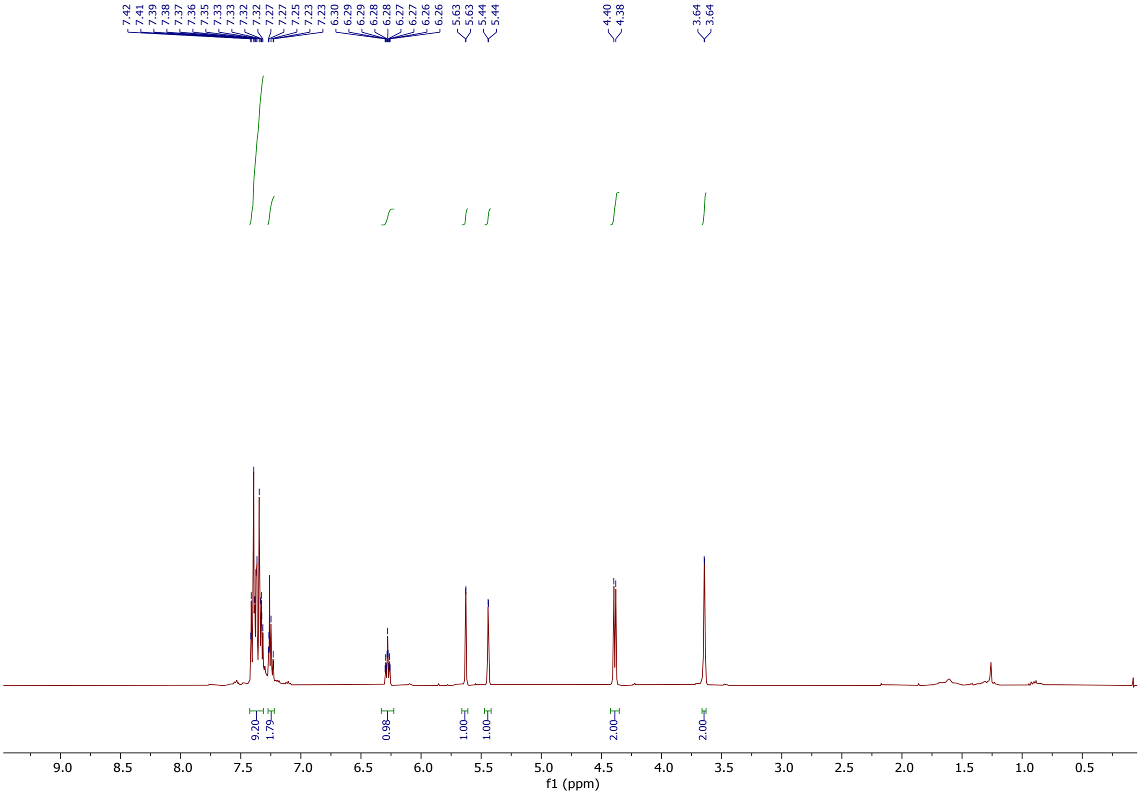

^{1}H NMR (400 MHz, CDCl$_3$) δ 7.43 – 7.31 (m, 9H), 7.27 – 7.22 (m, 1H), 6.28 (tt, J = 6.5, 1.8 Hz, 1H), 5.63 (d, J = 1.2 Hz, 1H), 5.44 (d, J = 1.3 Hz, 1H), 4.39 (d, J = 6.5 Hz, 2H), 3.64 (d, J = 1.5 Hz, 2H).

^{13}C NMR spectrum ($CDCl_3$)

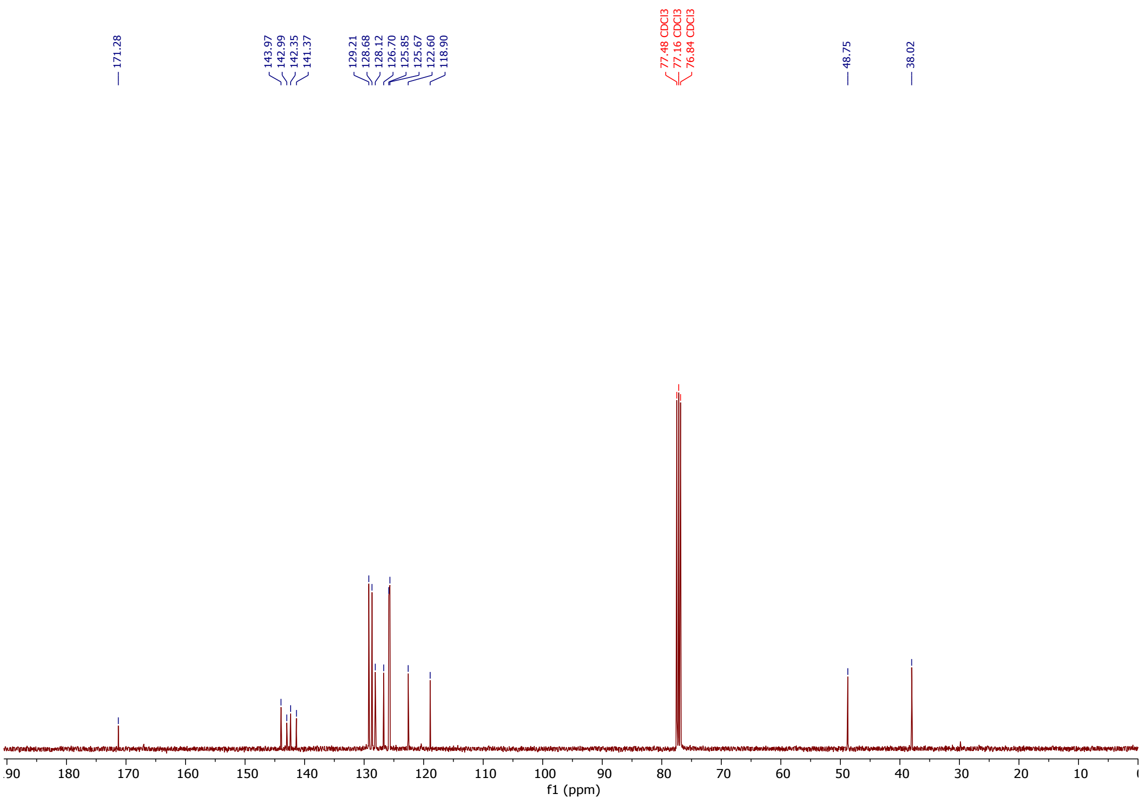

^{13}C NMR (101 MHz, $CDCl_3$) δ 171.28, 143.97, 142.99, 142.35, 141.37, 129.21, 128.68, 128.12, 126.70, 125.85, 125.67, 122.60, 118.90, 48.75, 38.02.

IR spectrum (neat)

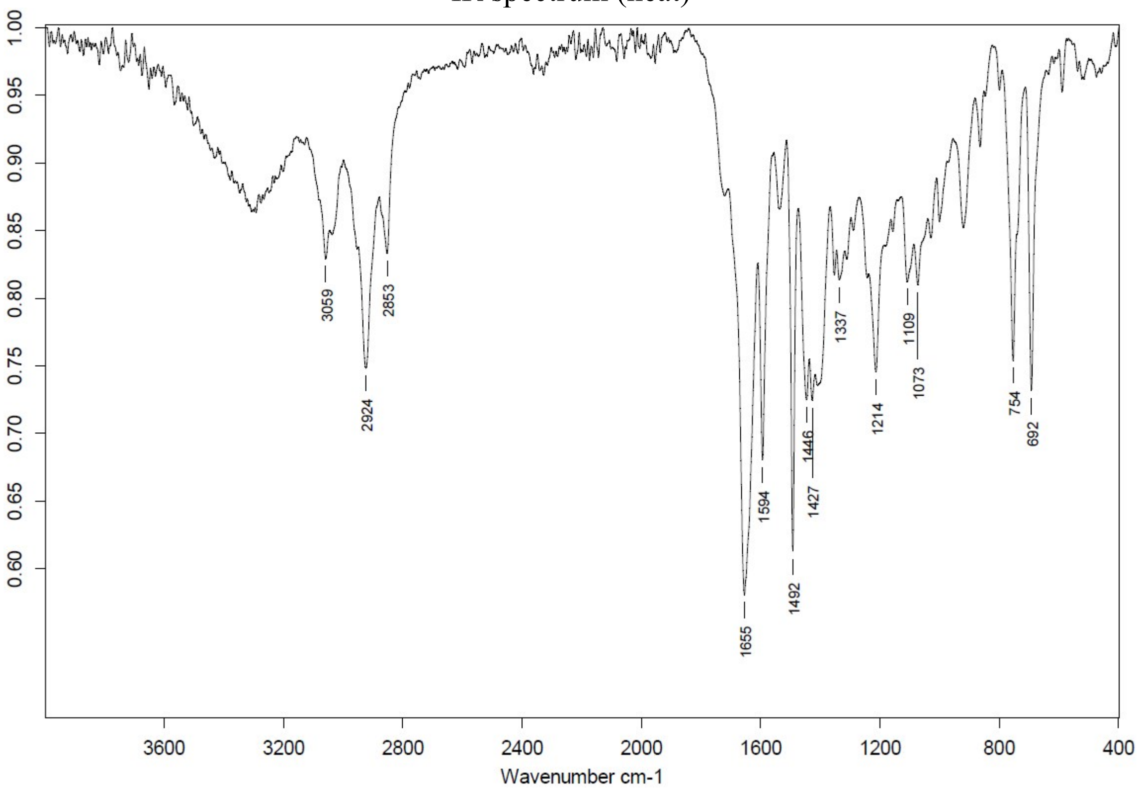

HRMS (ESI$^+$, MeOH): *m/z* calcd. 276.1383 $(M + H)^+$, found: 276.1380.

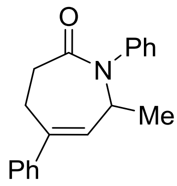

Compound 28

Scale: 0.2 mmol; *E/Z*>99:1, isolated 34.9 mg (63% yield), colorless oil, Hexane : EA = 1 : 1, R_f = 0.25

^{1}H NMR spectrum ($CDCl_3$)

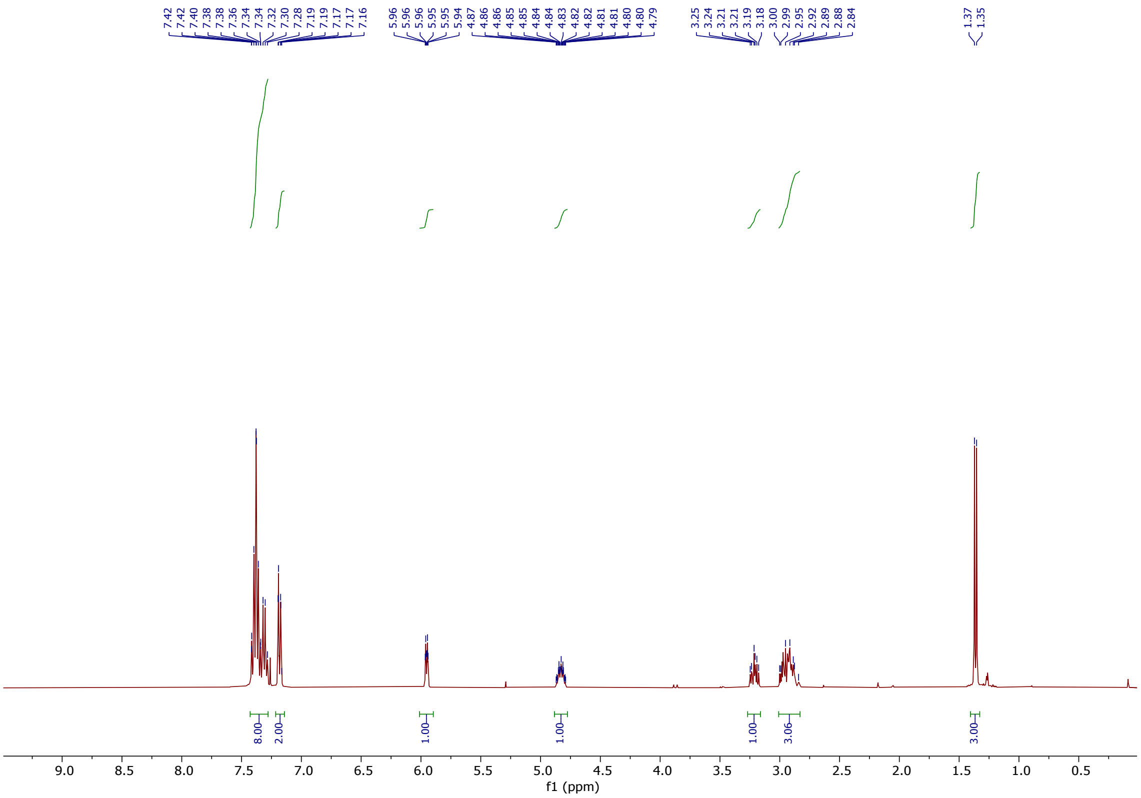

^{1}H NMR (400 MHz, $CDCl_3$) δ 7.43 – 7.28 (m, 8H), 7.21 – 7.14 (m, 2H), 5.95 (dt, J = 6.0, 1.7 Hz, 1H), 4.88 – 4.77 (m, 1H), 3.21 (td, J = 12.4, 11.8, 5.2 Hz, 1H), 3.01 – 2.83 (m, 3H), 1.36 (d, J = 7.2 Hz, 3H).

^{13}C NMR spectrum ($CDCl_3$)

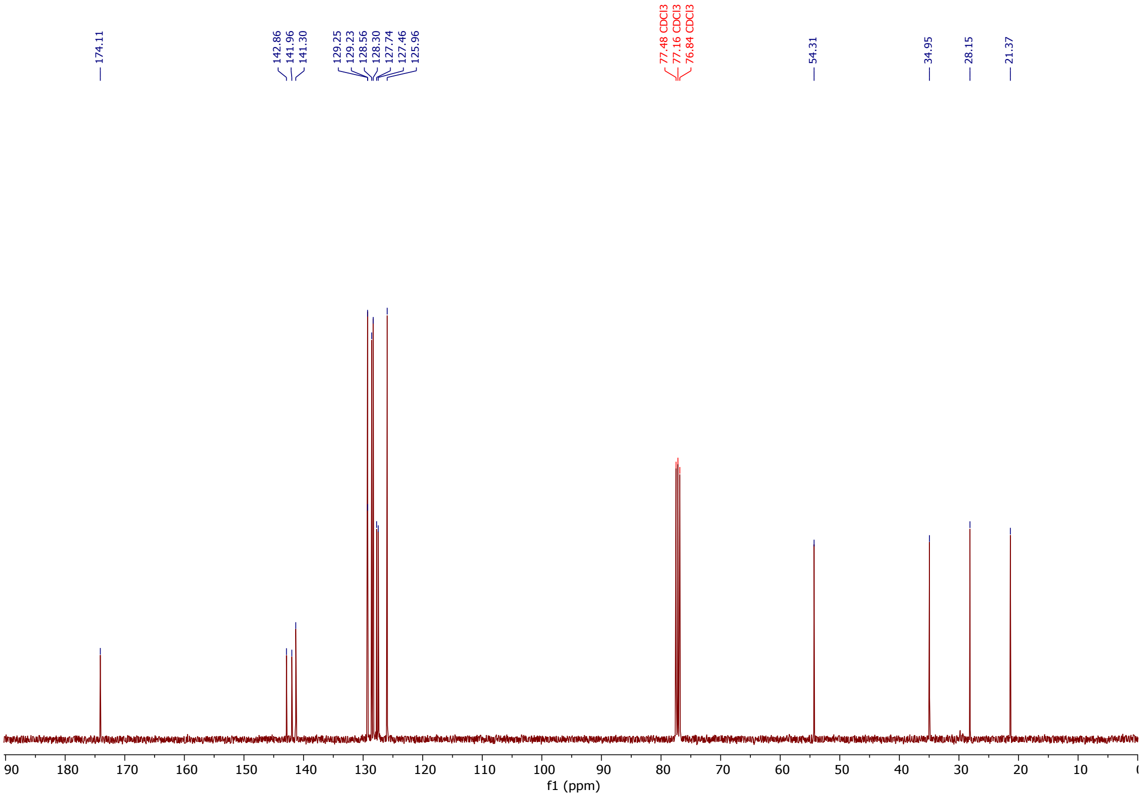

^{13}C NMR (101 MHz, $CDCl_3$) δ 174.11, 142.86, 141.96, 141.30, 129.25, 129.23, 128.56, 128.30, 127.74, 127.46, 125.96, 54.31, 34.95, 28.15, 21.37.

IR spectrum (neat)

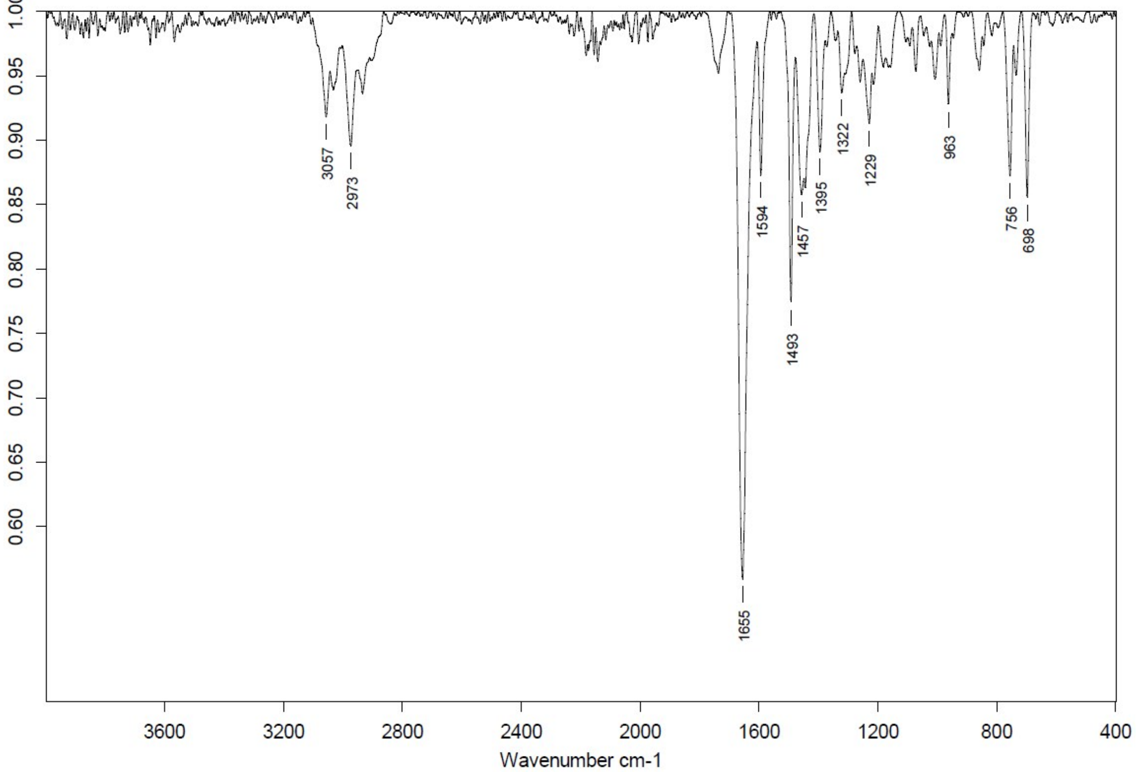

HRMS (ESI$^+$, MeOH): *m/z* calcd. 278.1539 $(M + H)^+$, found: 278.1542.

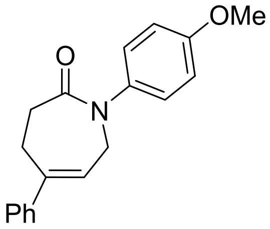

Compound 29

Scale: 0.2 mmol; *E/Z* = 97:3, isolated 46.9 mg (80% yield), slight yellow solid, Hexane : EA = 2 : 1, R_f = 0.25

^{1}H NMR spectrum ($CDCl_3$)

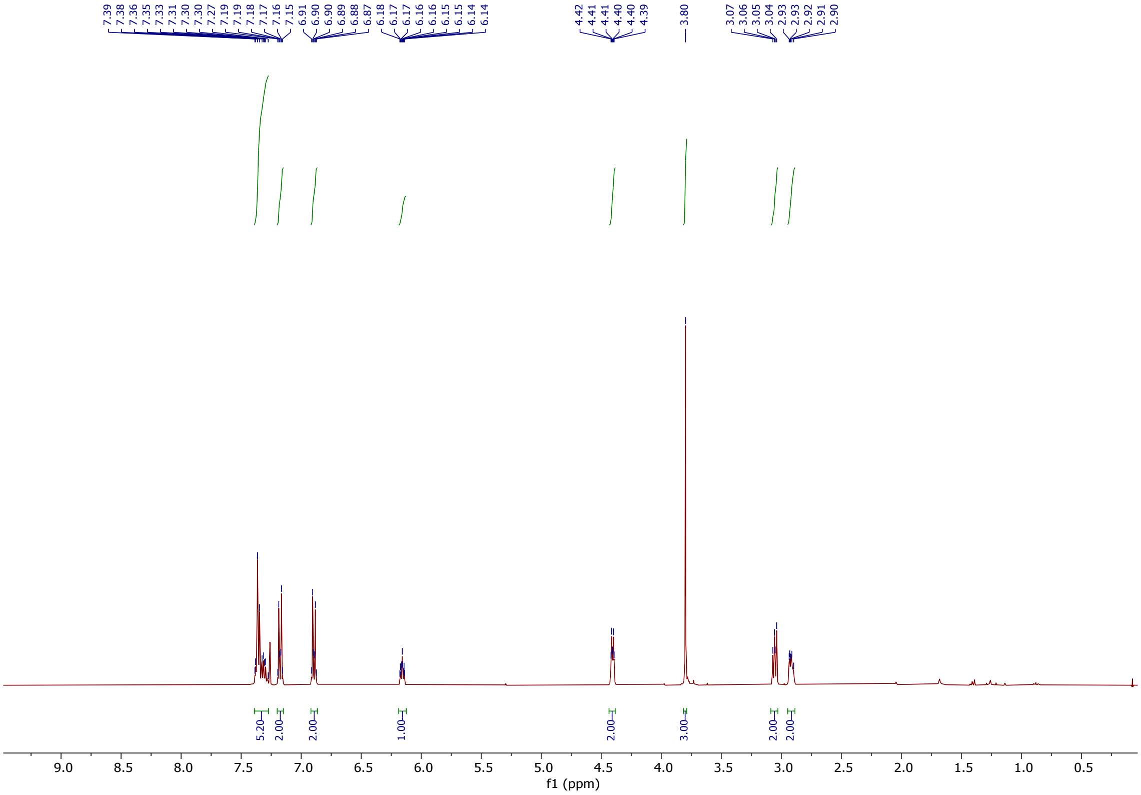

^{1}H NMR (400 MHz, $CDCl_3$) δ 7.39 – 7.27 (m, 5H), 7.20 – 7.15 (m, 2H), 6.92 – 6.86 (m, 2H), 6.16 (tt, *J* = 6.1, 1.9 Hz, 1H), 4.41 (dt, *J* = 6.1, 2.2 Hz, 2H), 3.80 (s, 3H), 3.08 – 3.02 (m, 2H), 2.95 – 2.89 (m, 2H).

^{13}C NMR spectrum ($CDCl_3$)

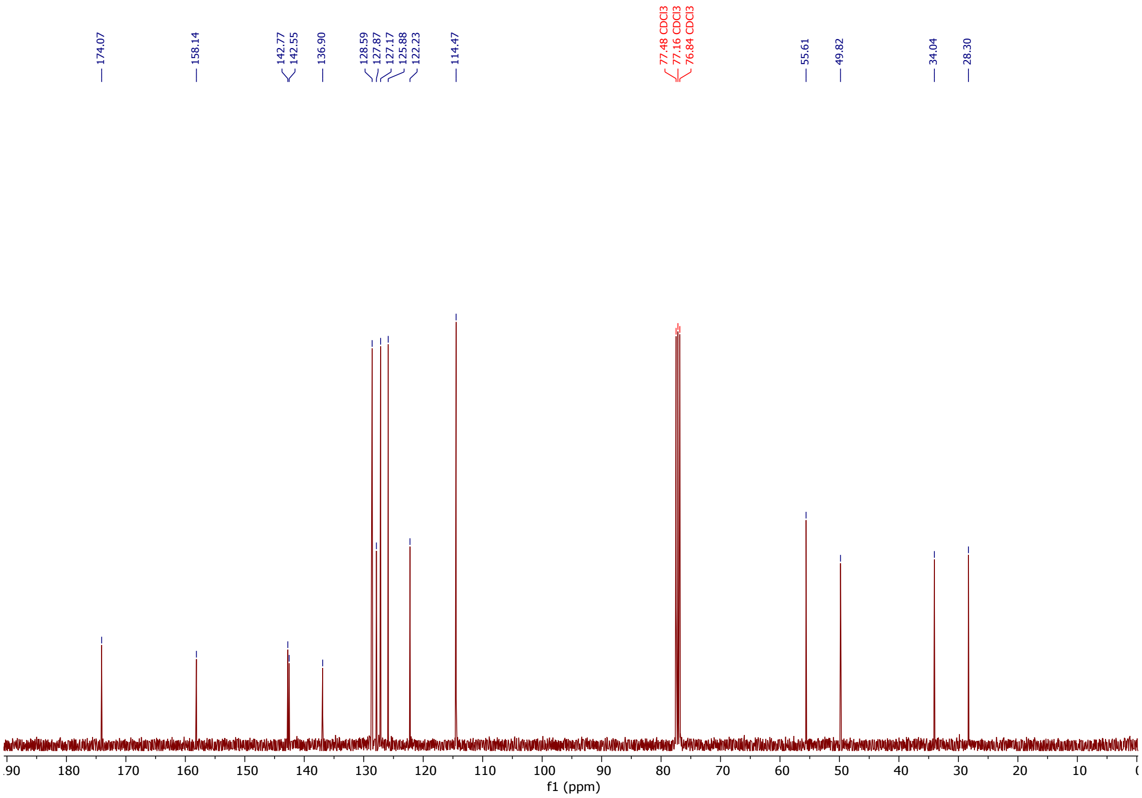

^{13}C NMR (101 MHz, $CDCl_3$) δ 174.07, 158.14, 142.77, 142.55, 136.90, 128.59, 127.87, 127.17, 125.88, 122.23, 114.47, 55.61, 49.82, 34.04, 28.30.

IR spectrum (neat)

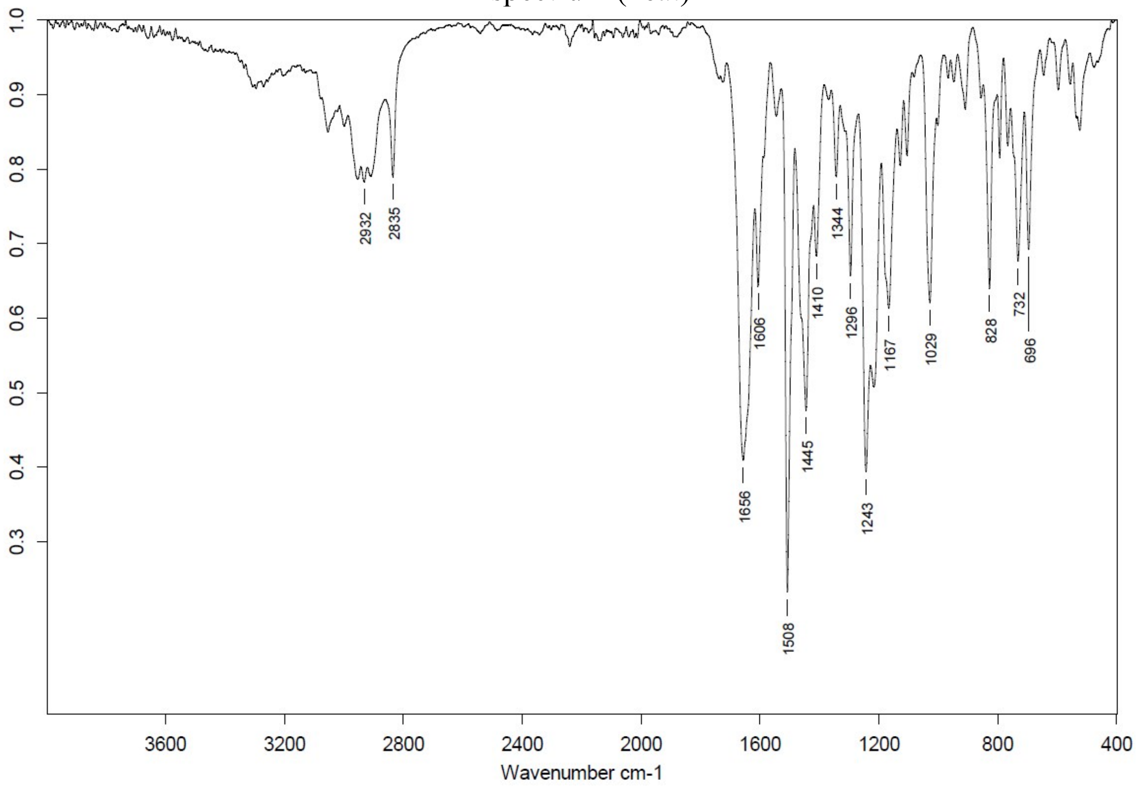

HRMS (ESI$^+$, MeOH): *m/z* calcd. 294.1489 $(M + H)^+$, found: 294.1489.

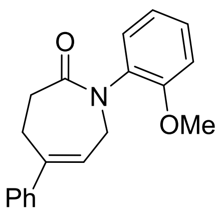

Compound 30

Scale: 0.2 mmol; *E/Z*>99:1, isolated 53.4 mg (91% yield), slight yellow solid, Hexane : EA = 2 : 1, $R_f = 0.25$

^{1}H NMR spectrum ($CDCl_3$)

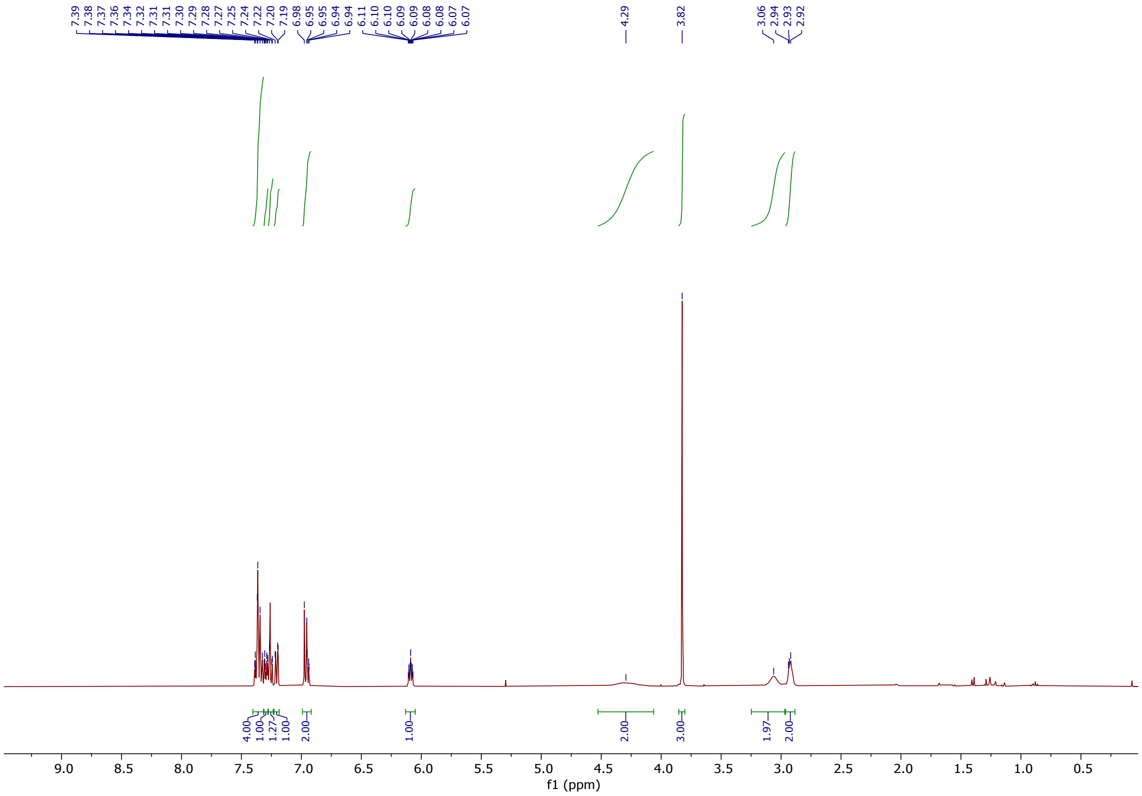

^{1}H NMR (400 MHz, $CDCl_3$) δ 7.40 – 7.32 (m, 4H), 7.31 – 7.28 (m, 1H), 7.27 – 7.23 (m, 1H), 7.23 – 7.18 (m, 1H), 6.99 – 6.92 (m, 2H), 6.09 (tt, *J* = 5.9, 1.8 Hz, 1H), 4.29 (s, 2H), 3.82 (s, 3H), 2.94 (s, 2H), 2.96 – 2.87 (m, 2H).

^{13}C NMR spectrum ($CDCl_3$)

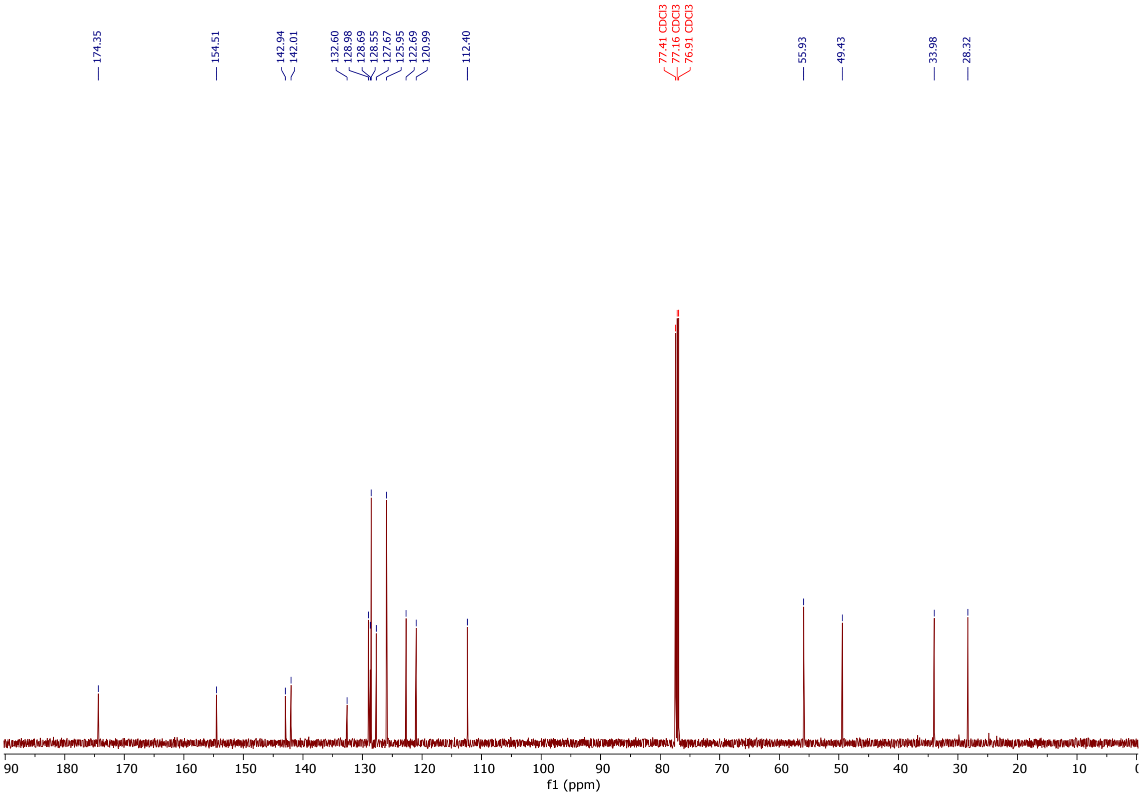

^{13}C NMR (126 MHz, $CDCl_3$) δ 174.35, 154.51, 142.94, 142.01, 132.60, 128.98, 128.69, 128.55, 127.67, 125.95, 122.69, 120.99, 112.40, 55.93, 49.43, 33.98, 28.32.

IR spectrum (neat)

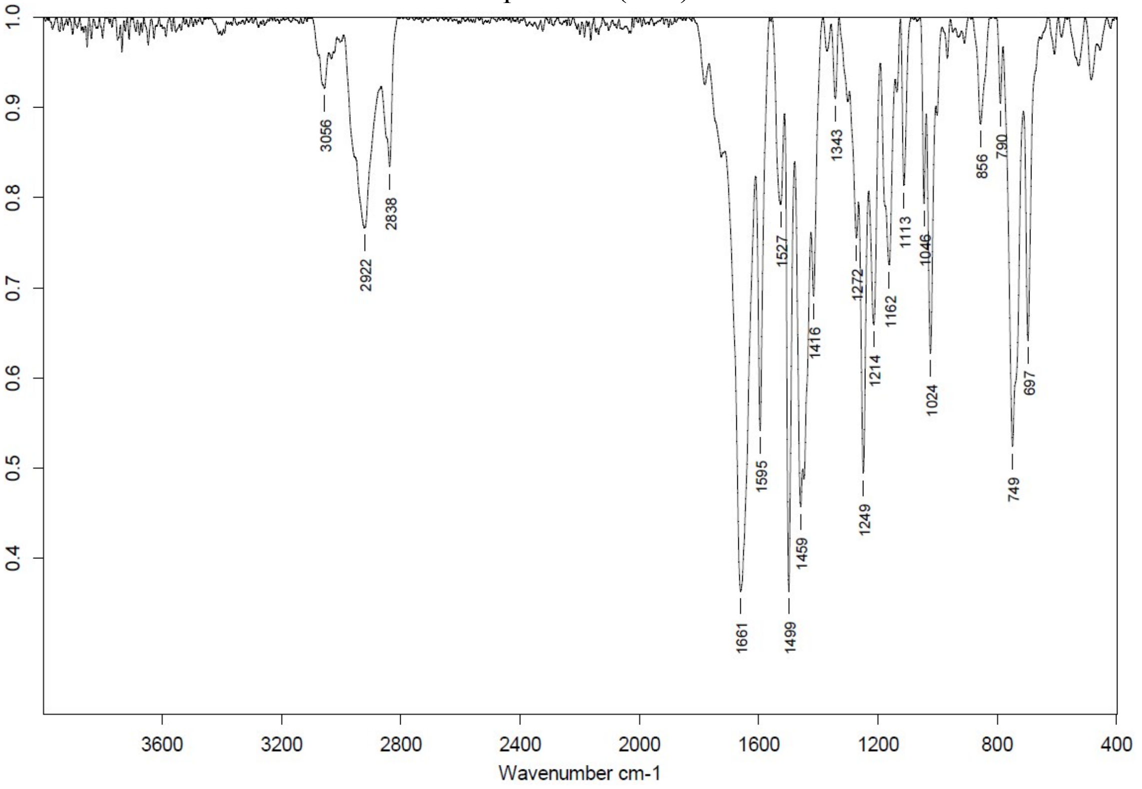

HRMS (ESI$^+$, MeOH): *m/z* calcd. 294.1489 $(M + H)^+$, found: 294.1490.

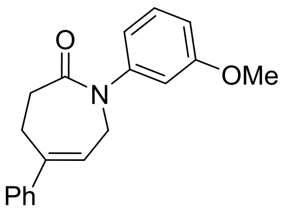

Compound 31

Scale: 0.2 mmol; *E/Z* = 99:1, isolated 50.5 mg (86% yield), yellow solid, Hexane : EA = 2 : 1, R_f = 0.25

^{1}H NMR spectrum ($CDCl_3$)

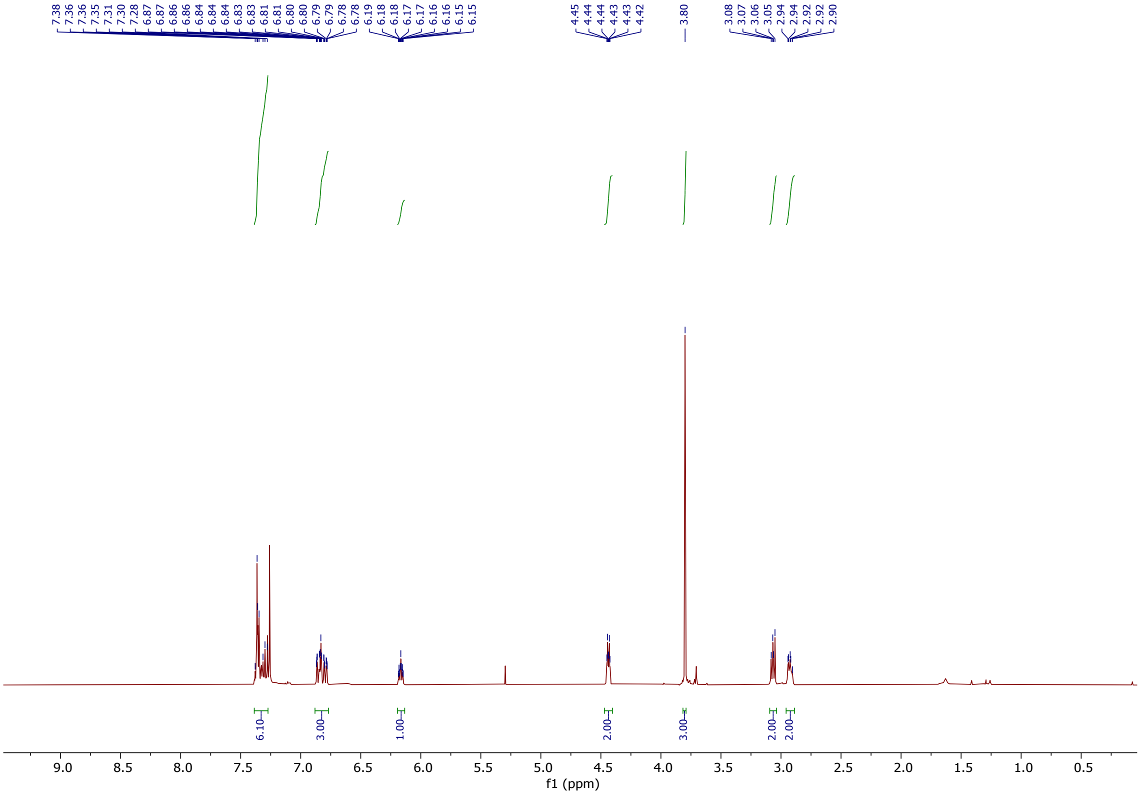

^{1}H NMR (400 MHz, $CDCl_3$) δ 7.39 – 7.27 (m, 6H), 6.88 – 6.77 (m, 3H), 6.17 (tt, *J* = 6.1, 1.8 Hz, 1H), 4.44 (dt, *J* = 6.1, 2.1 Hz, 2H), 3.80 (s, 3H), 3.10 – 3.04 (m, 2H), 2.96 – 2.89 (m, 2H).

^{13}C NMR spectrum ($CDCl_3$)

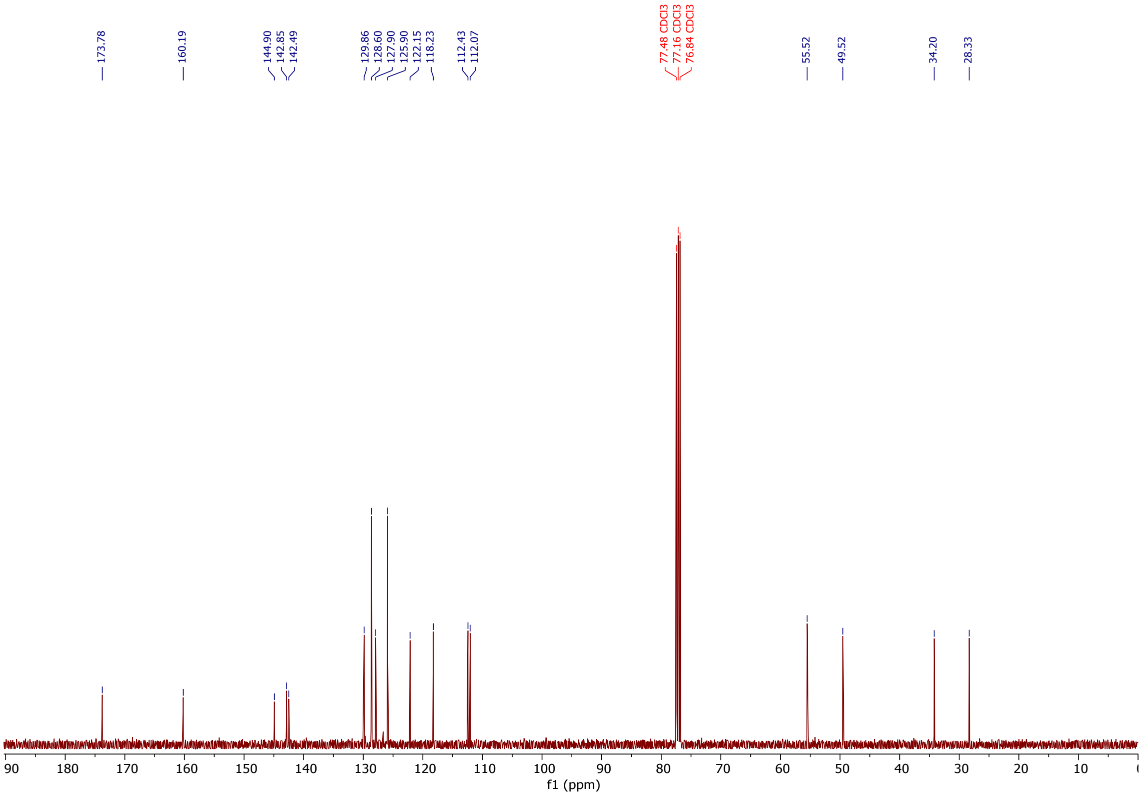

NMR (101 MHz, $CDCl_3$) δ 173.78, 160.19, 144.90, 142.85, 142.49, 129.86, 128.60, 127.90, 125.90, 122.15, 118.23, 112.43, 112.07, 55.52, 49.52, 34.20, 28.33.

IR spectrum (neat)

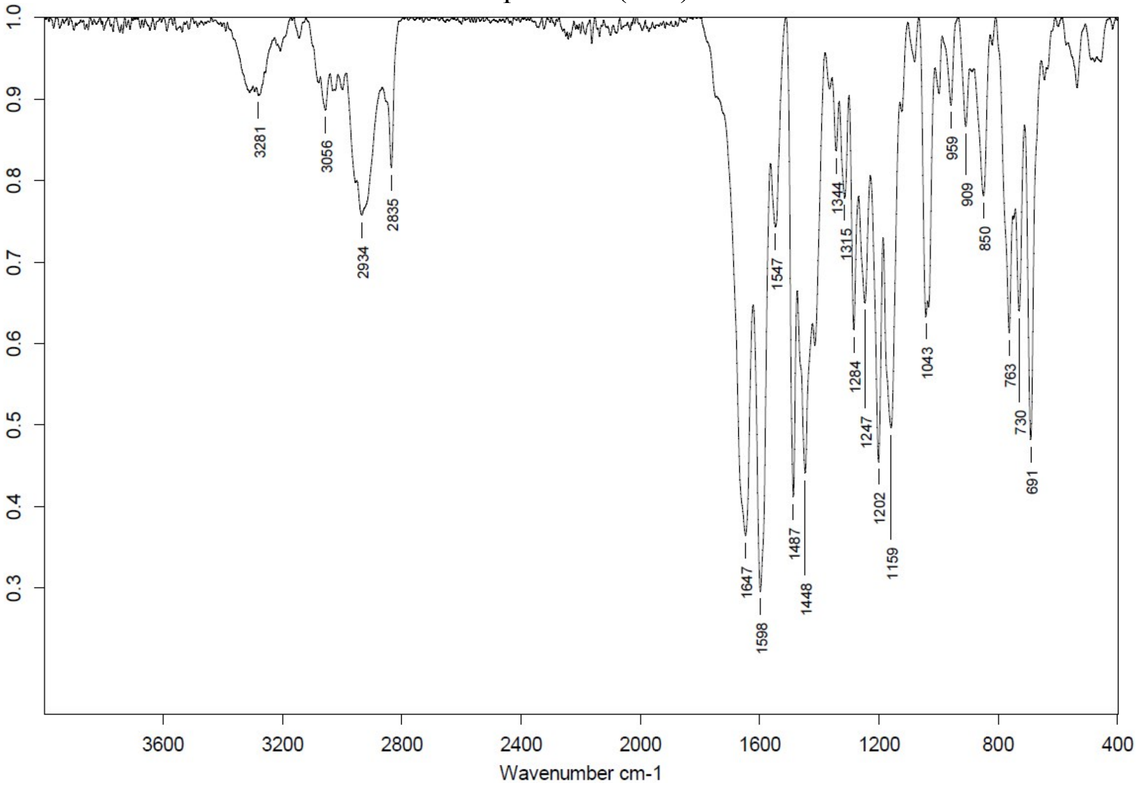

HRMS (ESI$^+$, MeOH): *m/z* calcd. 294.1489 $(M + H)^+$, found: 294.1489.

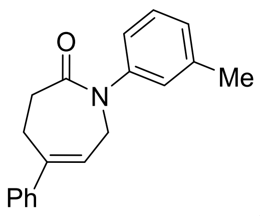

Compound 32

Scale: 0.2 mmol; *E/Z* = 92:8, isolated 41.1 mg (74% yield), yellow solid, Hexane : EA = 2 : 1, R_f = 0.25

^{1}H NMR spectrum ($CDCl_3$)

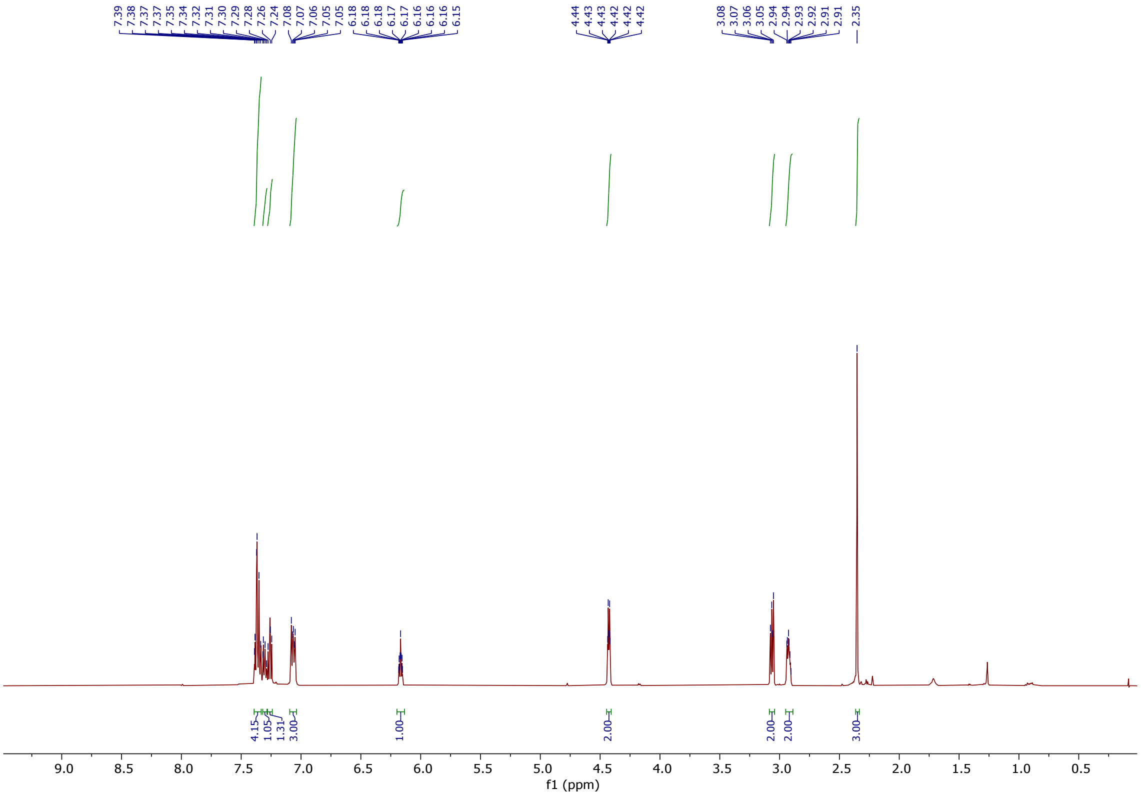

^{1}H NMR (500 MHz, $CDCl_3$) δ 7.39 – 7.33 (m, 4H), 7.32 – 7.28 (m, 1H), 7.28 – 7.24 (m, 1H), 7.09 – 7.04 (m, 3H), 6.17 (tt, *J* = 6.1, 1.8 Hz, 1H), 4.43 (dt, *J* = 6.1, 2.1 Hz, 2H), 3.09 – 3.03 (m, 2H), 2.95 – 2.89 (m, 2H), 2.35 (s, 3H).

^{13}C NMR spectrum ($CDCl_3$)

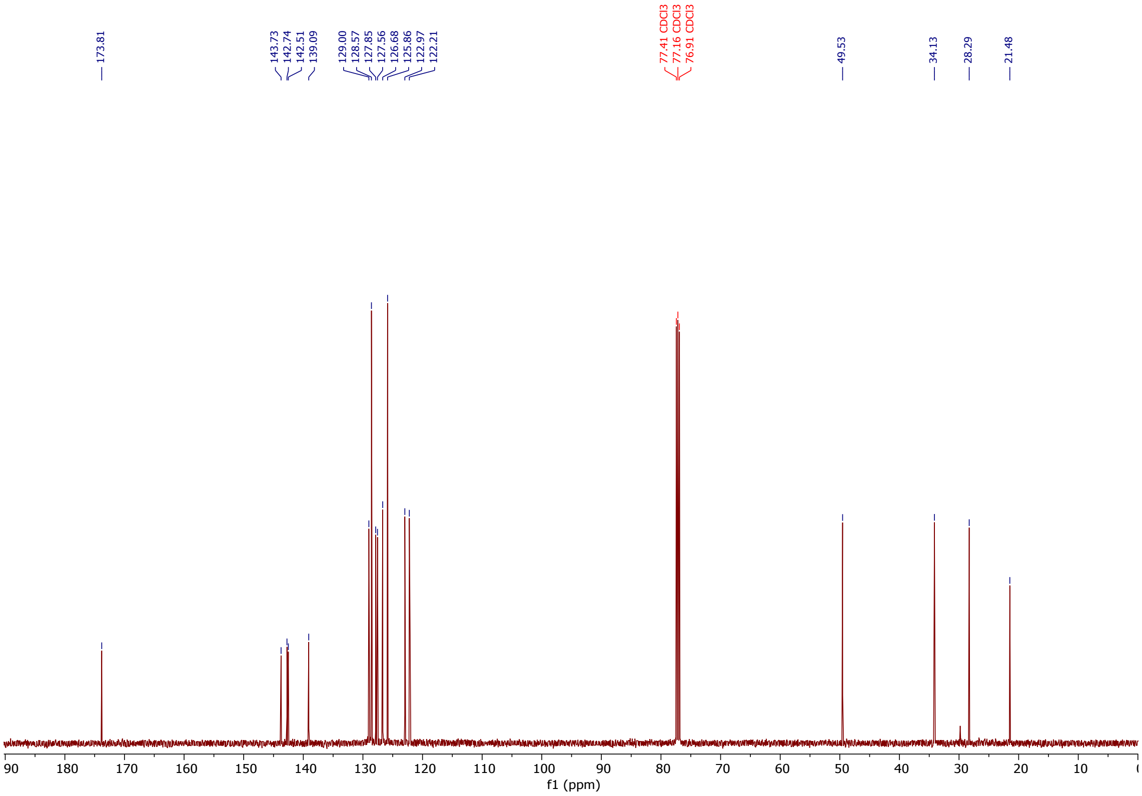

^{13}C NMR (126 MHz, $CDCl_3$) δ 173.81, 143.73, 142.74, 142.51, 139.09, 129.00, 128.57, 127.85, 127.56, 126.68, 125.86, 122.97, 122.21, 49.53, 34.13, 28.29, 21.48.

IR spectrum (neat)

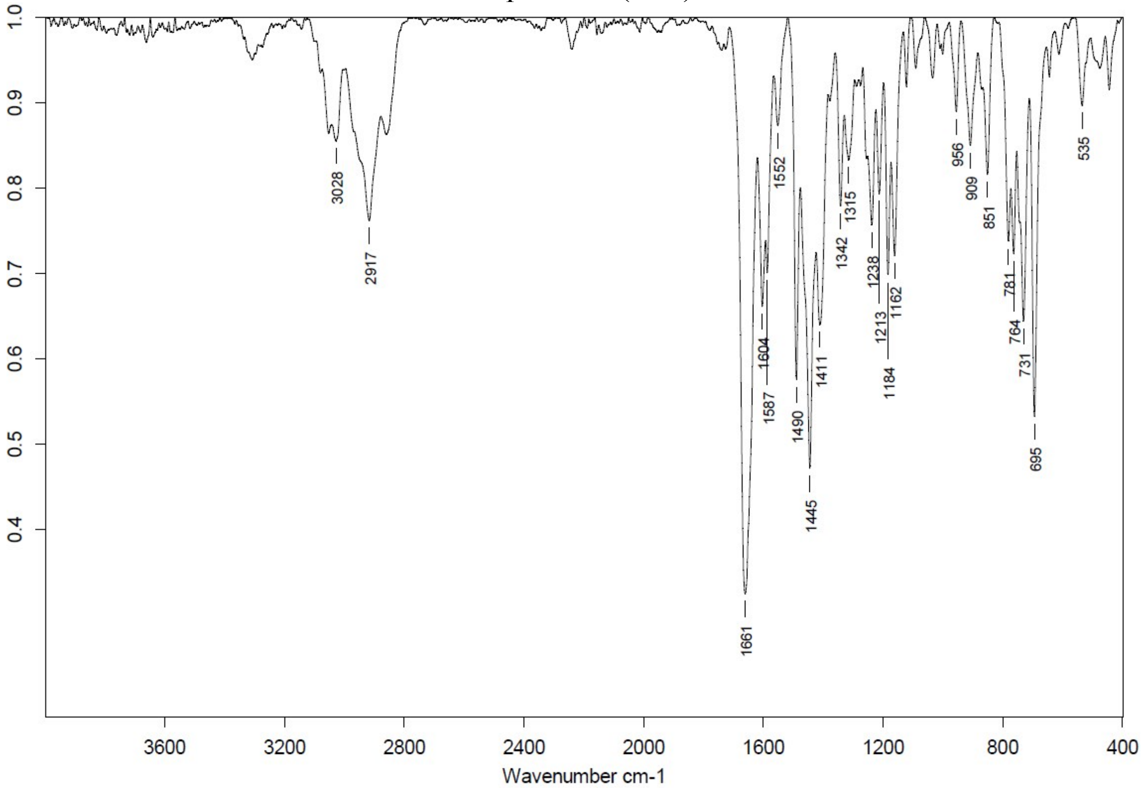

HRMS (ESI$^+$, MeOH): *m/z* calcd. 278.1539 $(M + H)^+$, found: 278.1546.

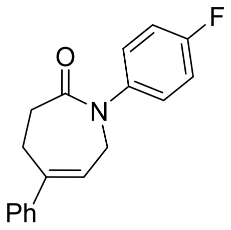

Compound 33

Scale: 0.2 mmol; *E/Z* = 98:2, isolated 52.3 mg (93% yield), yellow solid, Hexane : EA = 2 : 1, R_f = 0.25

^{1}H NMR spectrum ($CDCl_3$)

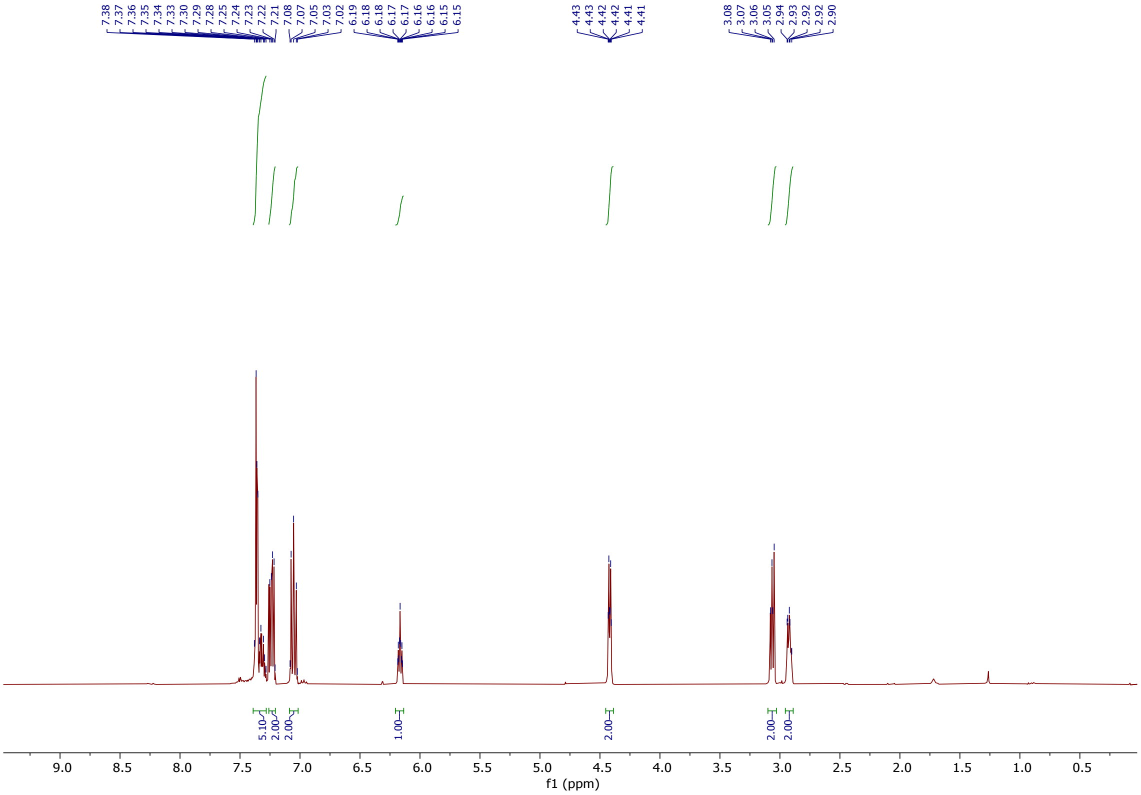

^{1}H NMR (400 MHz, $CDCl_3$) δ 7.39 – 7.28 (m, 5H), 7.26 – 7.20 (m, 2H), 7.09 – 7.02 (m, 2H), 6.17 (tt, *J* = 6.0, 1.8 Hz, 1H), 4.42 (dt, *J* = 6.1, 2.1 Hz, 2H), 3.09 – 3.03 (m, 2H), 2.95 – 2.89 (m, 2H).

^{13}C NMR spectrum ($CDCl_3$)

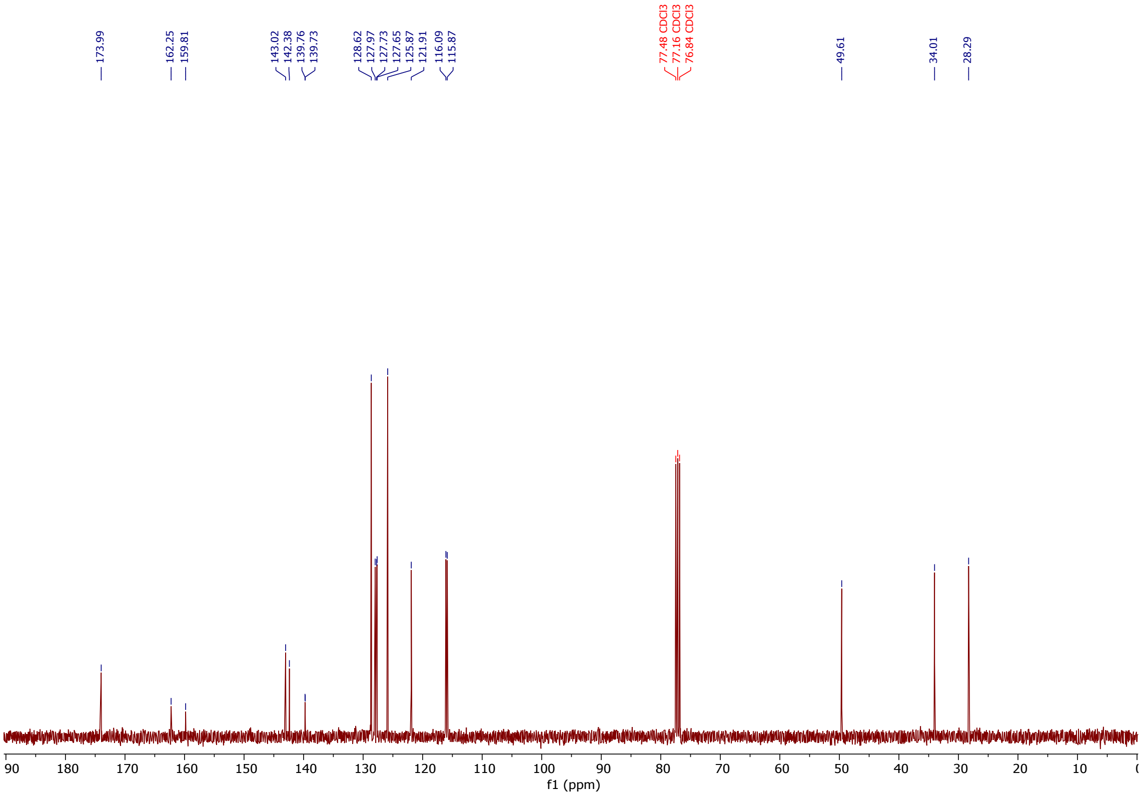

^{13}C NMR (101 MHz, $CDCl_3$) δ 173.99, 161.03 (d, J = 245.9 Hz), 143.02, 142.38, 139.74 (d, J = 3.3 Hz), 128.62, 127.97, 127.69 (d, J = 8.4 Hz), 125.87, 121.91, 115.98 (d, J = 22.7 Hz), 49.61, 34.01, 28.29.

^{19}F NMR spectrum ($CDCl_3$)

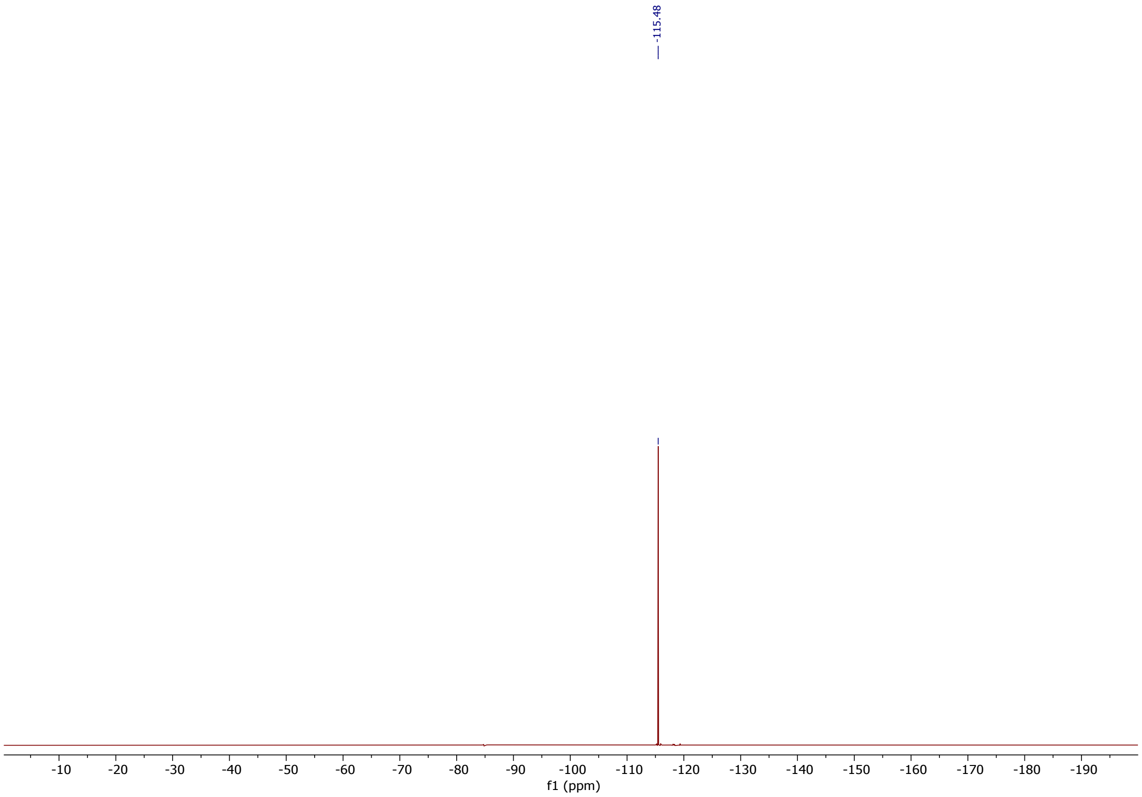

^{19}F NMR (376 MHz, $CDCl_3$) δ -115.48.

IR spectrum (neat)

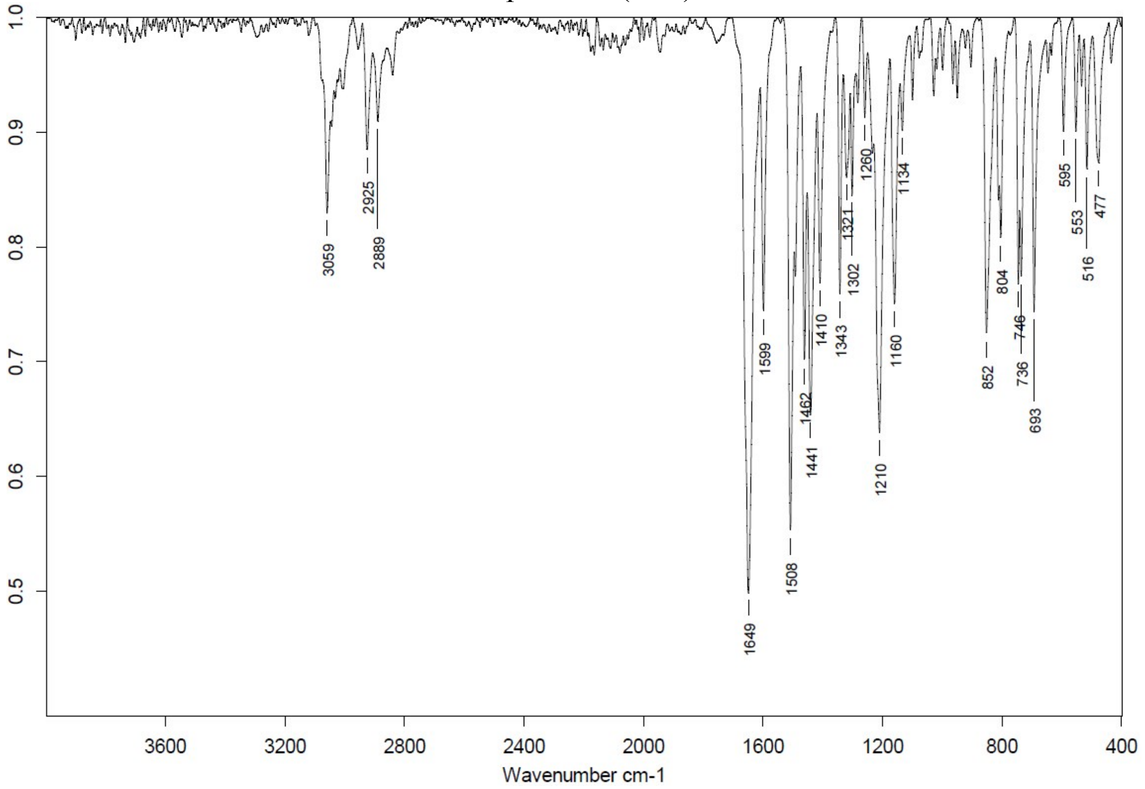

HRMS (ESI$^+$, MeOH): *m/z* calcd. 282.1289 (M + H)$^+$, found: 282.1295.

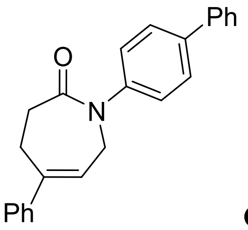

Compound 34

Scale: 0.2 mmol; *E/Z* = 97:3, isolated 50.9 mg (75% yield), white solid, Hexane : EA = 2 : 1, R_f = 0.15

^{1}H NMR spectrum ($CDCl_3$)

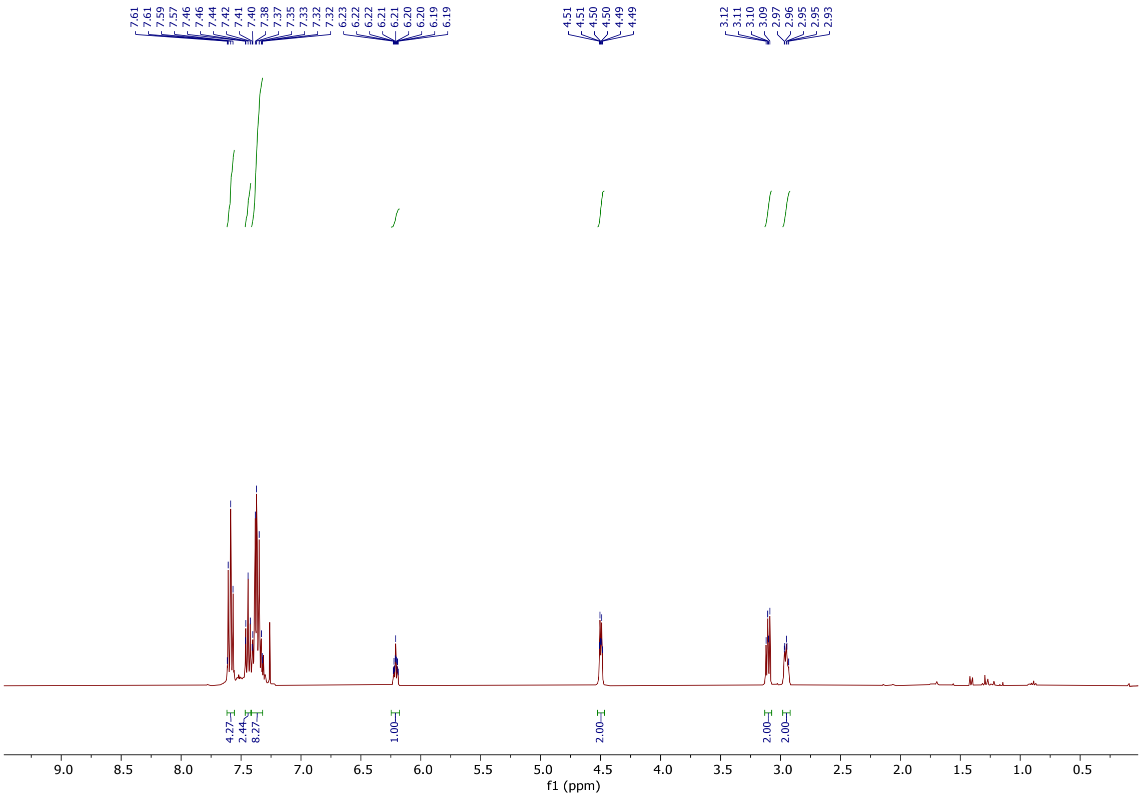

^{1}H NMR (400 MHz, $CDCl_3$) δ 7.62 – 7.56 (m, 4H), 7.47 – 7.42 (m, 2H), 7.41 – 7.32 (m, 8H), 6.21 (tt, *J* = 6.1, 1.8 Hz, 1H), 4.50 (dt, *J* = 6.1, 2.1 Hz, 2H), 3.13 – 3.07 (m, 2H), 2.98 – 2.91 (m, 2H).

^{13}C NMR spectrum ($CDCl_3$)

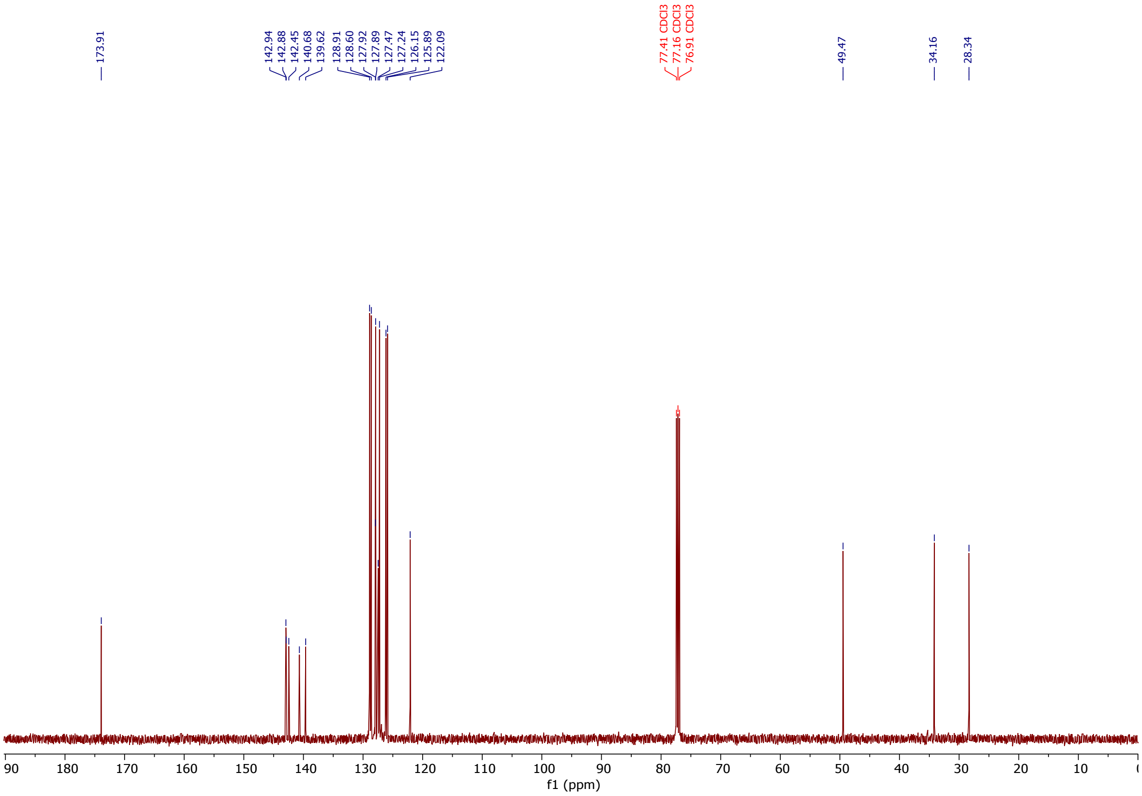

^{13}C NMR (126 MHz, $CDCl_3$) δ 173.91, 142.94, 142.88, 142.45, 140.68, 139.62, 128.91, 128.60, 127.92, 127.89, 127.47, 127.24, 126.15, 125.89, 122.09, 49.47, 34.16, 28.34.

IR spectrum (neat)

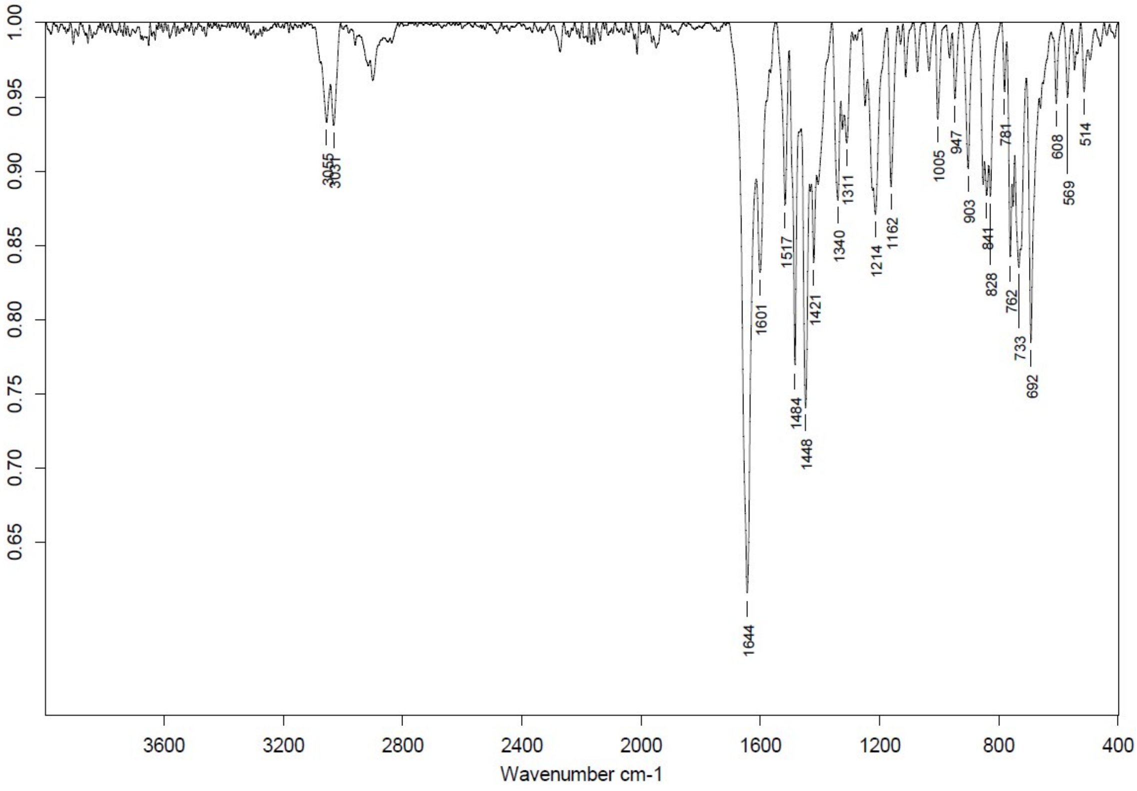

HRMS (ESI^+, MeOH): *m/z* calcd. 340.1696 $(M + H)^+$, found: 340.1705.

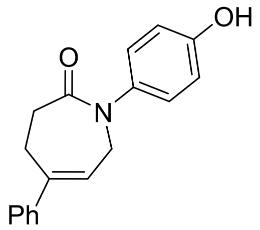

Compound 35

Scale: 0.2 mmol; *E/Z*>99:1, isolated 32.4 mg (58% yield), white solid, DCM : MeOH = 50 : 1, $R_f = 0.13$

^{1}H NMR spectrum ($CDCl_3$)

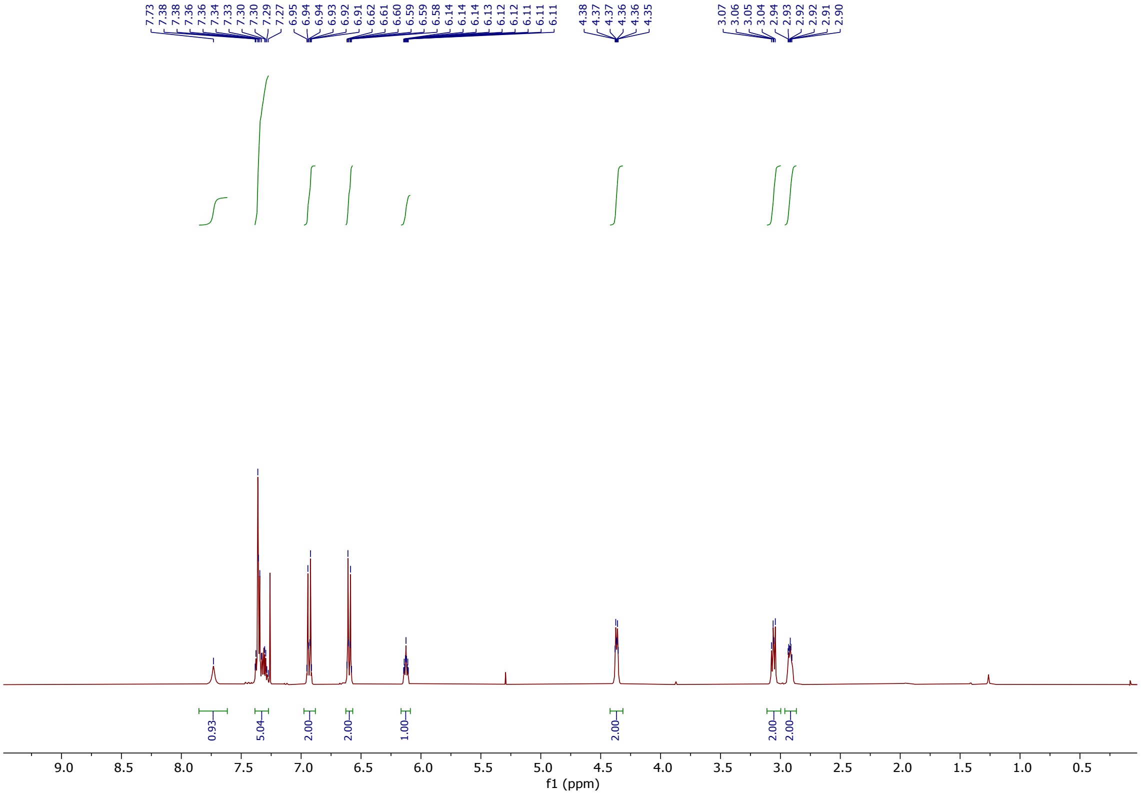

^{1}H NMR (400 MHz, $CDCl_3$) δ 7.73 (s, 1H), 7.39 – 7.27 (m, 5H), 6.98 – 6.88 (m, 2H), 6.63 – 6.57 (m, 2H), 6.12 (tt, *J* = 6.0, 1.8 Hz, 1H), 4.37 (dt, *J* = 6.1, 2.2 Hz, 2H), 3.12 – 3.00 (m, 2H), 2.96 – 2.86 (m, 2H).

^{13}C NMR spectrum ($CDCl_3$)

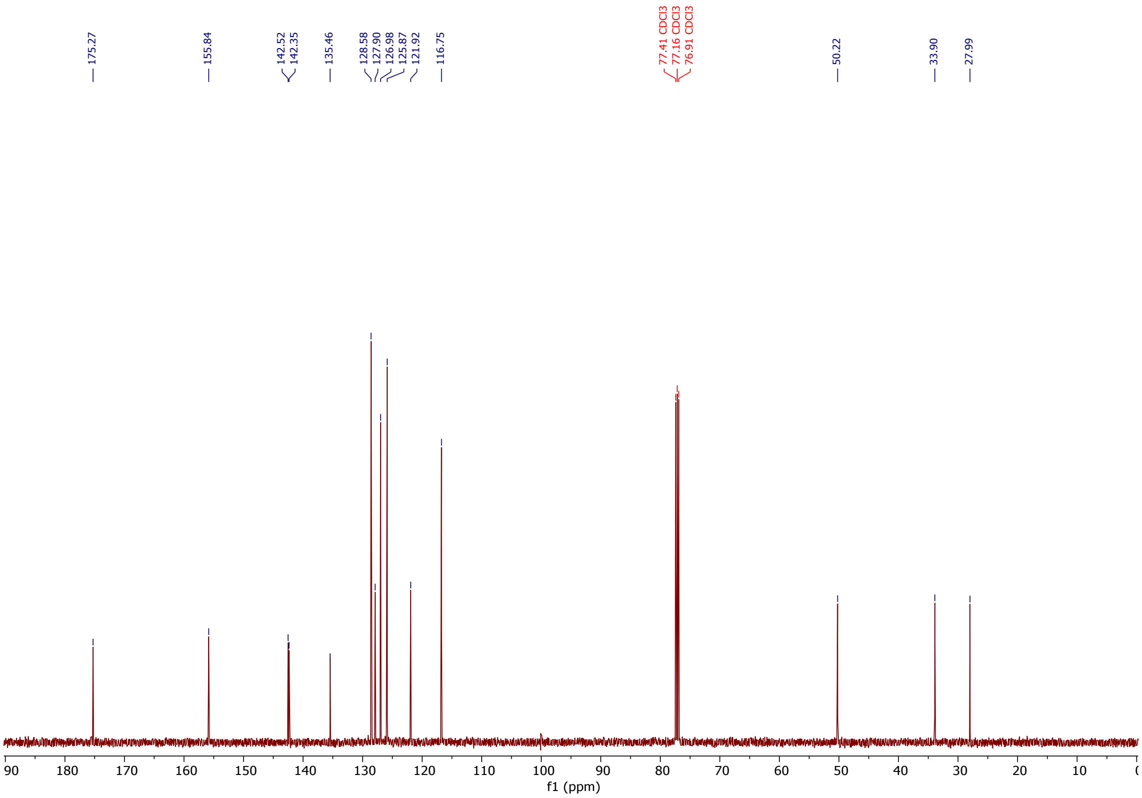

^{13}C NMR (126 MHz, $CDCl_3$) δ 175.27, 155.84, 142.52, 142.35, 135.46, 128.58, 127.90, 126.98, 125.87, 121.92, 116.75, 50.22, 33.90, 27.99.

IR spectrum (neat)

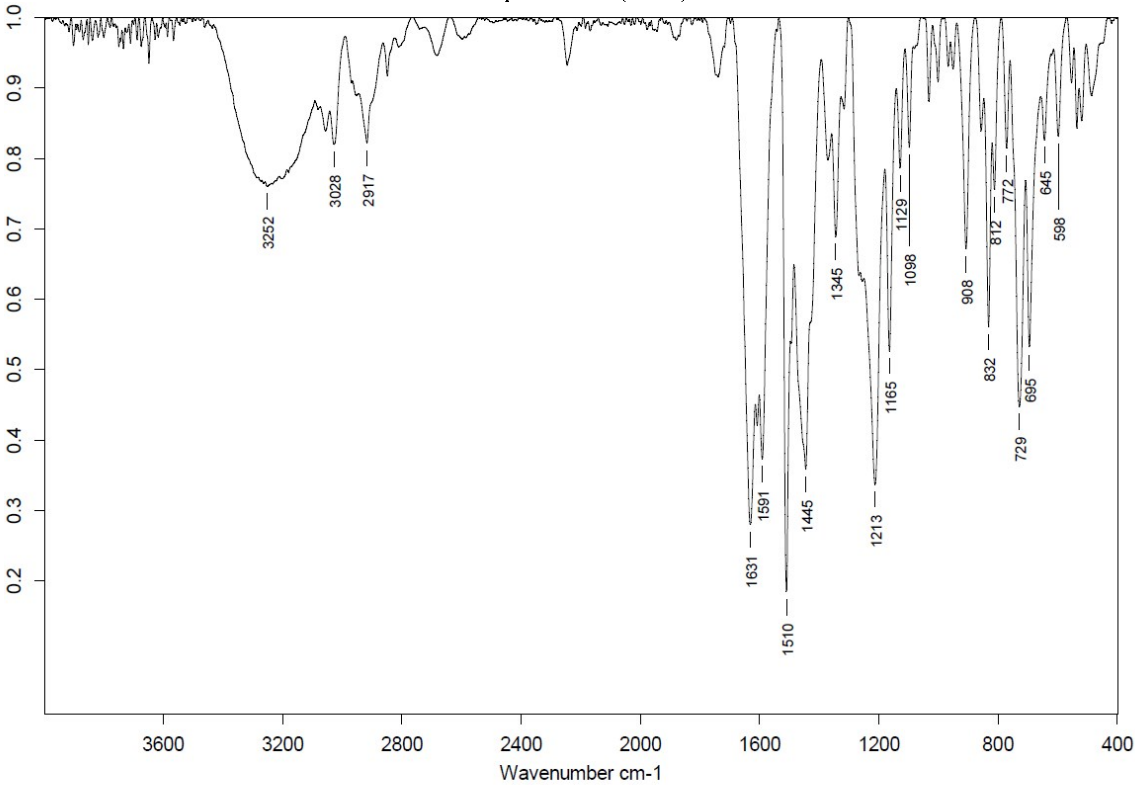

HRMS (ESI^+, MeOH): *m/z* calcd. 280.1332 $(M + H)^+$, found: 280.1330.

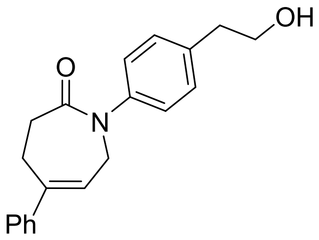

Compound 36

Scale: 0.2 mmol; *E/Z* = 94:6, isolated 38.1 mg (62% yield), yellow solid, Hexane : EA = 1 : 3, R_f = 0.2

^{1}H NMR spectrum ($CDCl_3$)

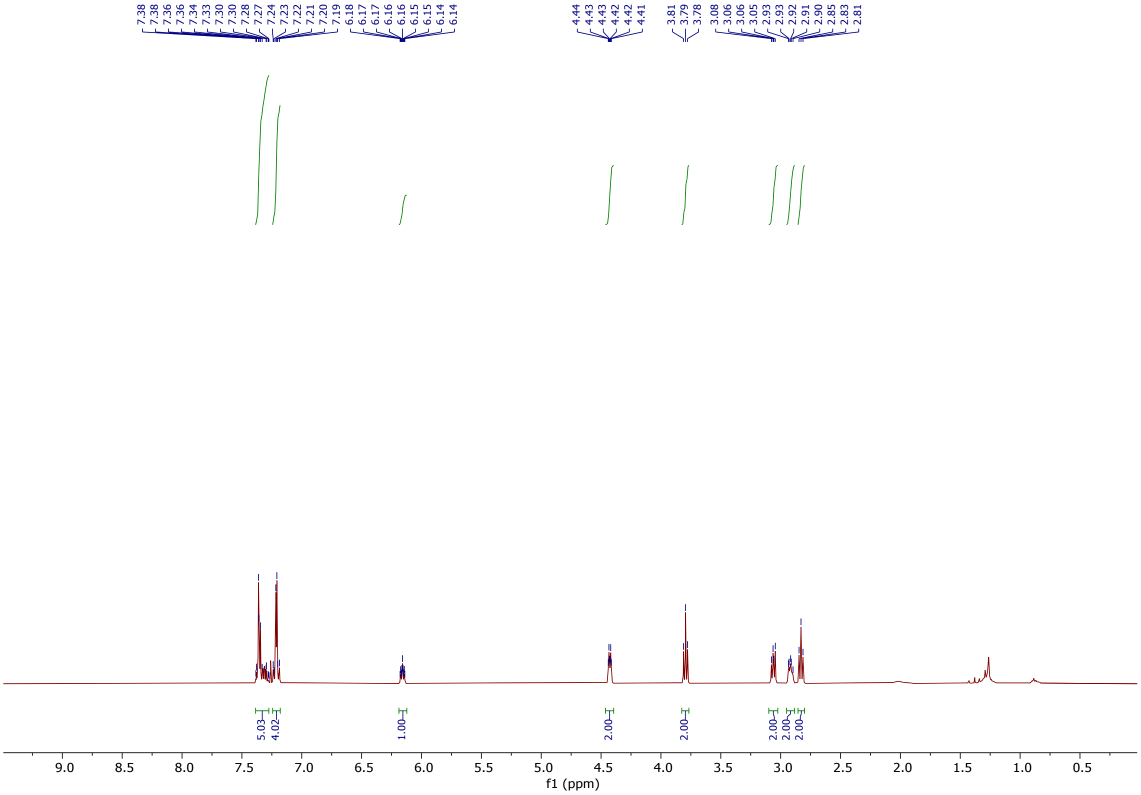

^{1}H NMR (400 MHz, $CDCl_3$) δ 7.39 – 7.27 (m, 5H), 7.24 – 7.18 (m, 4H), 6.16 (tt, J = 6.1, 1.8 Hz, 1H), 4.43 (dt, J = 6.1, 2.2 Hz, 2H), 3.79 (t, J = 6.7 Hz, 2H), 3.10 – 3.02 (m, 2H), 2.95 – 2.88 (m, 2H), 2.83 (t, J = 6.6 Hz, 2H).

^{13}C NMR spectrum ($CDCl_3$)

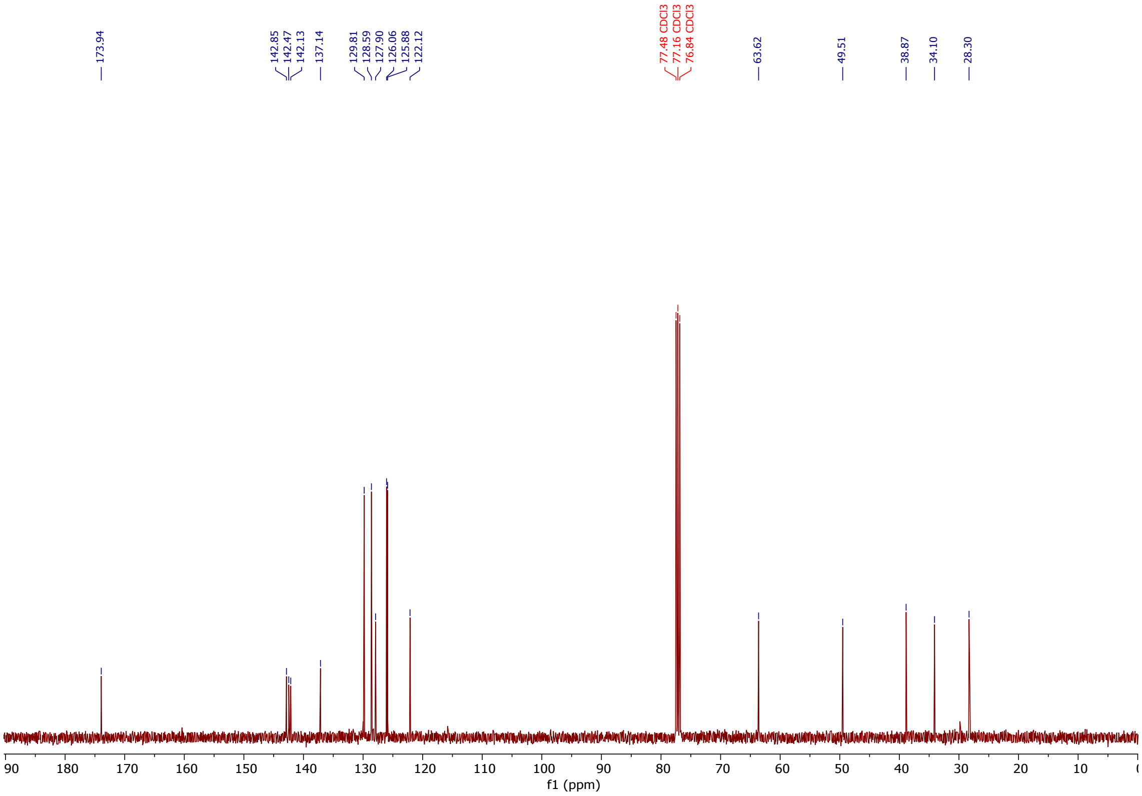

^{13}C NMR (101 MHz, $CDCl_3$) δ 173.94, 142.85, 142.47, 142.13, 137.14, 129.81, 128.59, 127.90, 126.06, 125.88, 122.12, 63.62, 49.51, 38.87, 34.10, 28.30.

IR spectrum (neat)

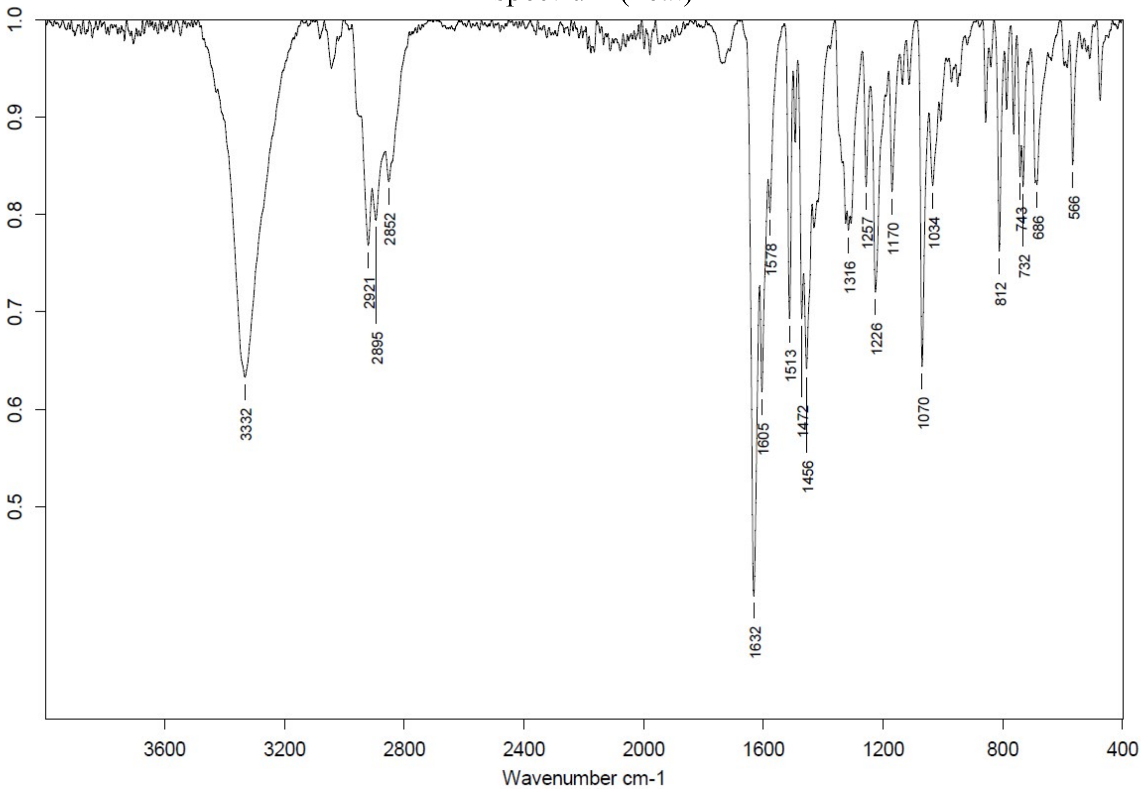

HRMS (ESI$^+$, MeOH): *m/z* calcd. 308.1645 $(M + H)^+$, found: 308.1652.

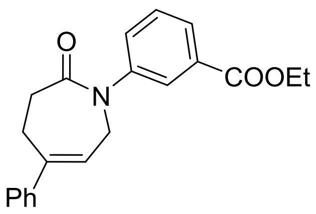

Compound 37

Scale: 0.2 mmol; *E/Z* = 96:4, isolated 45.6 mg (68% yield), yellow solid, Hexane : EA = 1 : 1, R_f = 0.15

^{1}H NMR spectrum ($CDCl_3$)

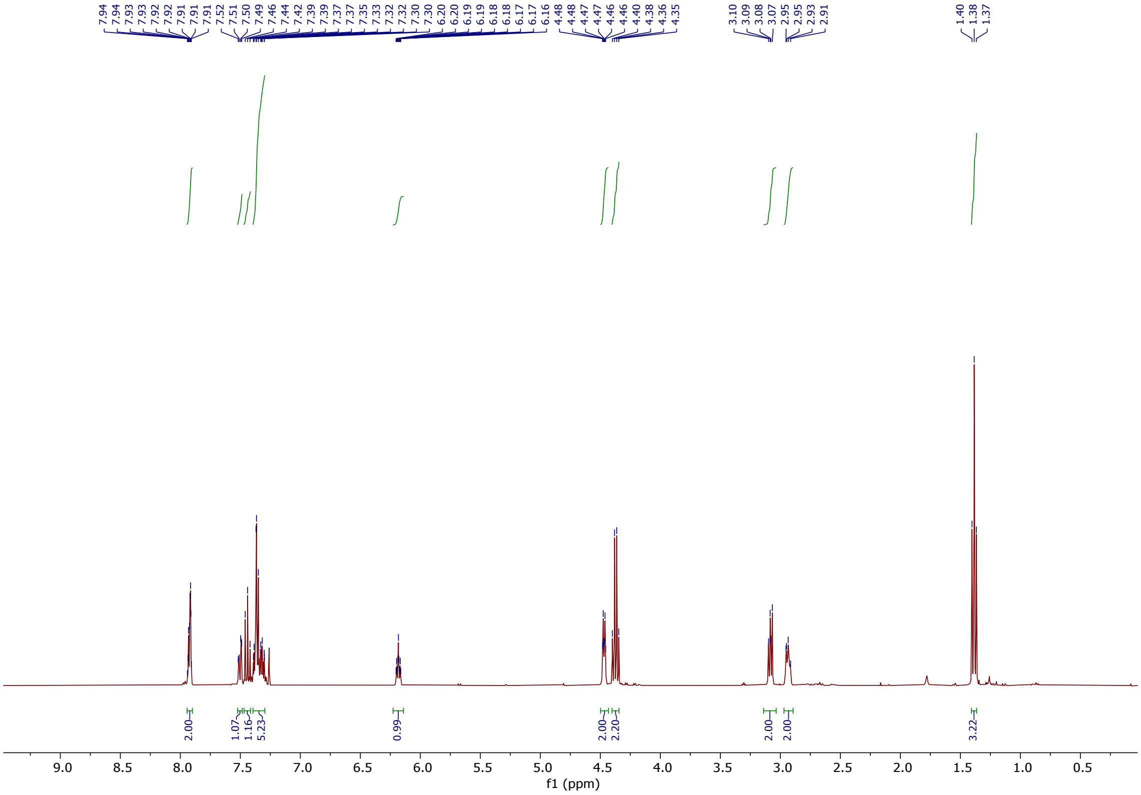

^{1}H NMR (400 MHz, $CDCl_3$) δ 7.94 – 7.90 (m, 2H), 7.52 – 7.46 (m, 1H), 7.47 – 7.40 (m, 1H), 7.40 – 7.30 (m, 5H), 6.18 (tt, J = 6.1, 1.8 Hz, 1H), 4.47 (dt, J = 6.1, 2.1 Hz, 2H), 4.37 (q, J = 7.1 Hz, 2H), 3.14 – 3.03 (m, 2H), 2.97 – 2.89 (m, 2H), 1.38 (t, J = 7.2 Hz, 3H).

^{13}C NMR spectrum ($CDCl_3$)

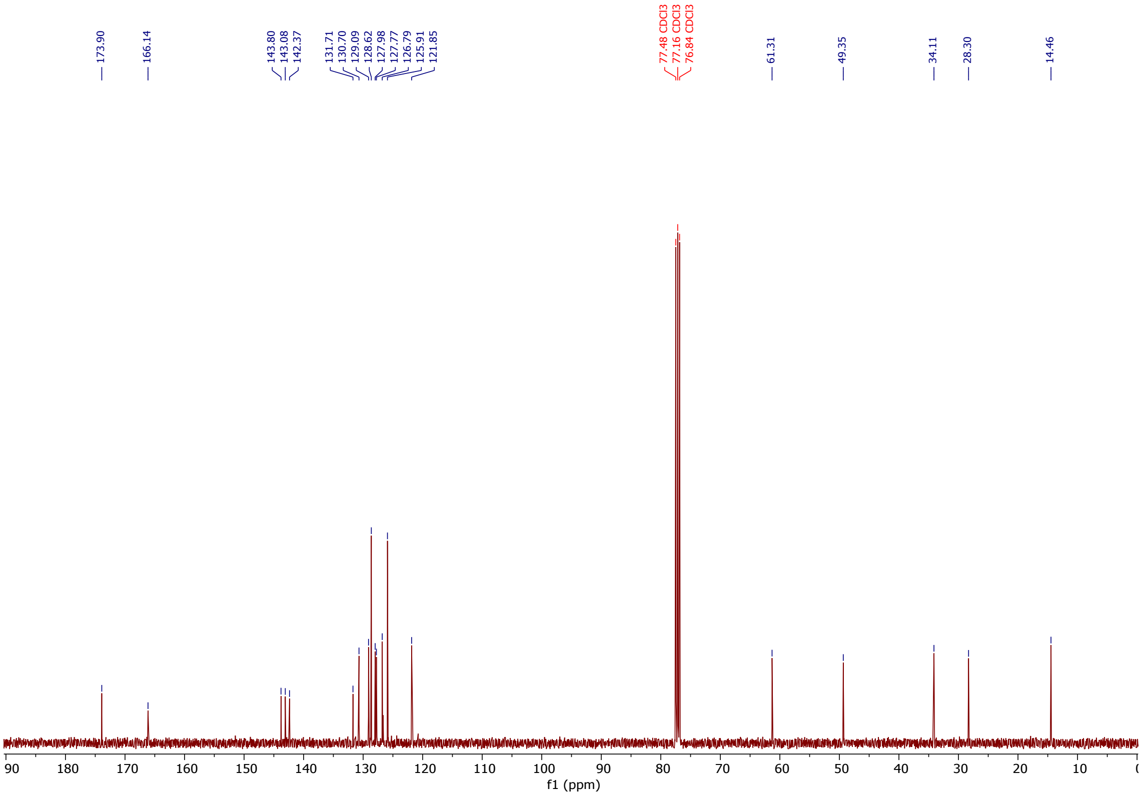

^{13}C NMR (101 MHz, $CDCl_3$) δ 173.90, 166.14, 143.80, 143.08, 142.37, 131.71, 130.70, 129.09, 128.62, 127.98, 127.77, 126.79, 125.91, 121.85, 61.31, 49.35, 34.11, 28.30, 14.46.

IR spectrum (neat)

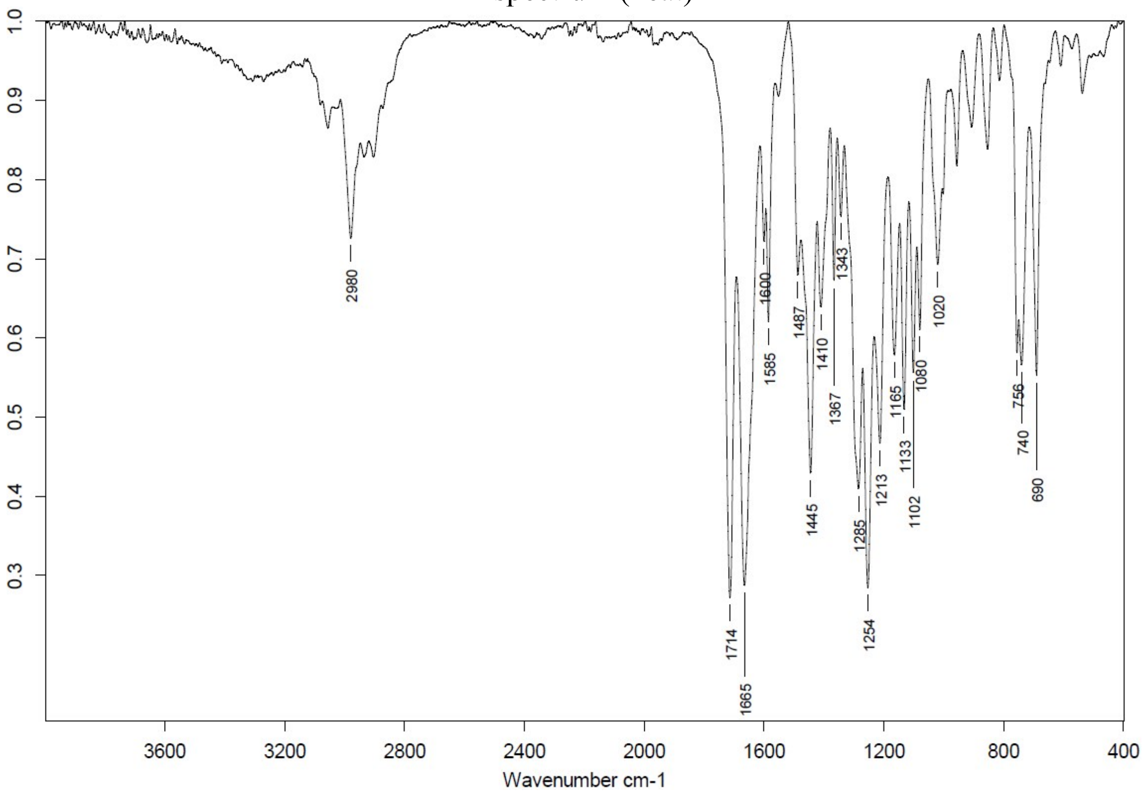

HRMS (ESI^+, MeOH): *m/z* calcd. 336.1594 $(M + H)^+$, found: 336.1595.

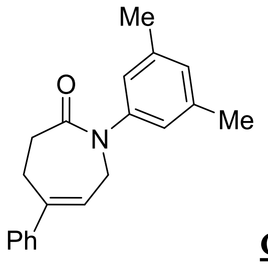

Compound 38

Scale: 0.2 mmol; *E/Z* = 94:6, isolated 48.4 mg (83% yield), yellow solid, Hexane : EA = 2 : 1, R_f = 0.25

^{1}H NMR spectrum ($CDCl_3$)

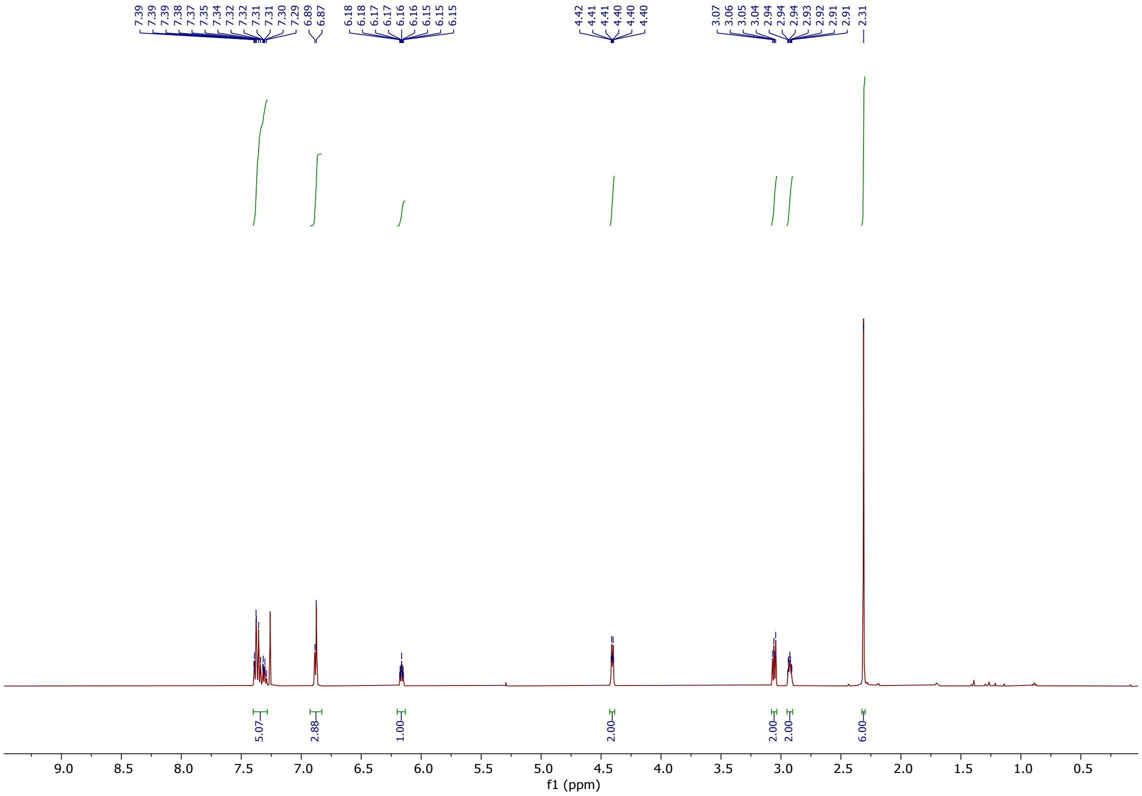

^{1}H NMR (500 MHz, $CDCl_3$) δ 7.40 – 7.28 (m, 5H), 6.92 – 6.82 (m, 3H), 6.16 (tt, *J* = 6.1, 1.8 Hz, 1H), 4.41 (dt, *J* = 6.1, 2.1 Hz, 2H), 3.08 – 3.04 (m, 2H), 2.95 – 2.90 (m, 2H), 2.31 (s, 6H).

^{13}C NMR spectrum ($CDCl_3$)

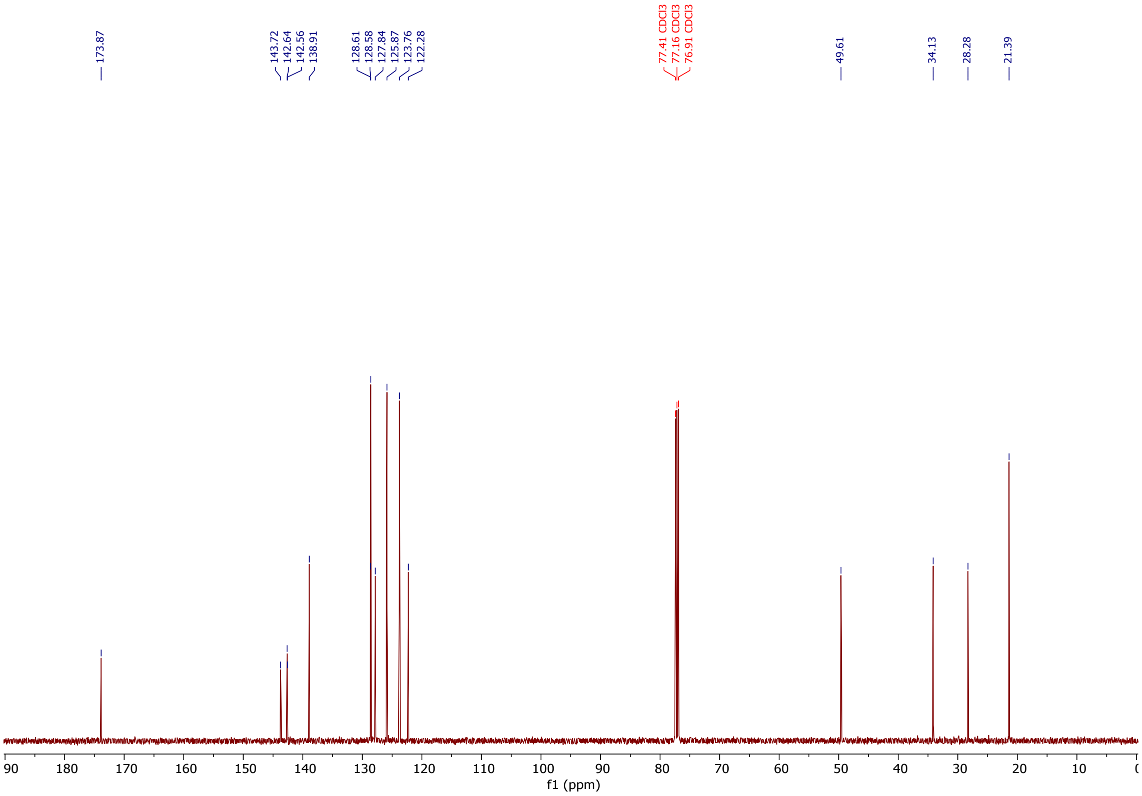

^{13}C NMR (126 MHz, $CDCl_3$) δ 173.87, 143.72, 142.64, 142.56, 138.91, 128.61, 128.58, 127.84, 125.87, 123.76, 122.28, 49.61, 34.13, 28.28, 21.39.

IR spectrum (neat)

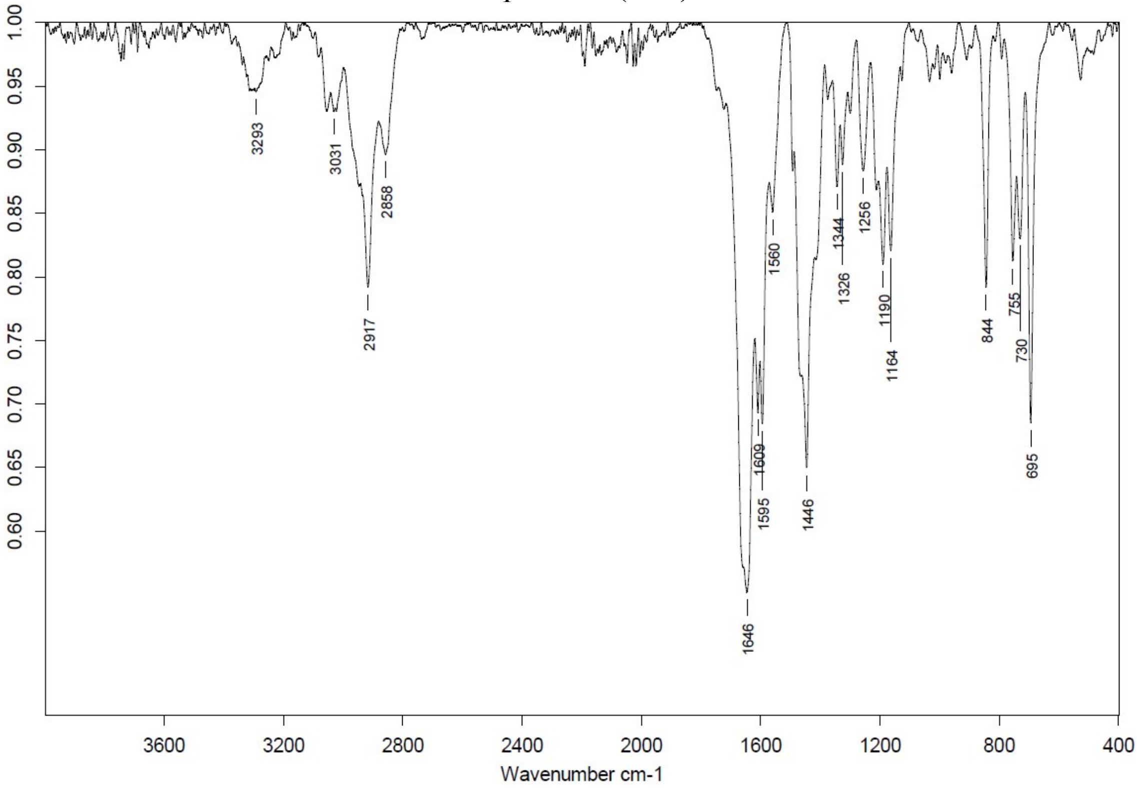

HRMS (ESI$^+$, MeOH): *m/z* calcd. 292.1696 $(M + H)^+$, found: 292.1704.

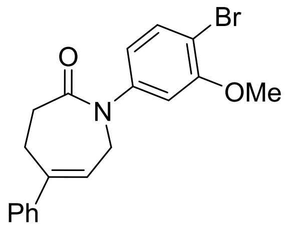

Compound 39

Scale: 0.2 mmol; *E/Z* = 99:1, isolated 54.4 mg (73% yield), yellow solid, Hexane : EA = 2 : 1, R_f = 0.18

^{1}H NMR spectrum ($CDCl_3$)

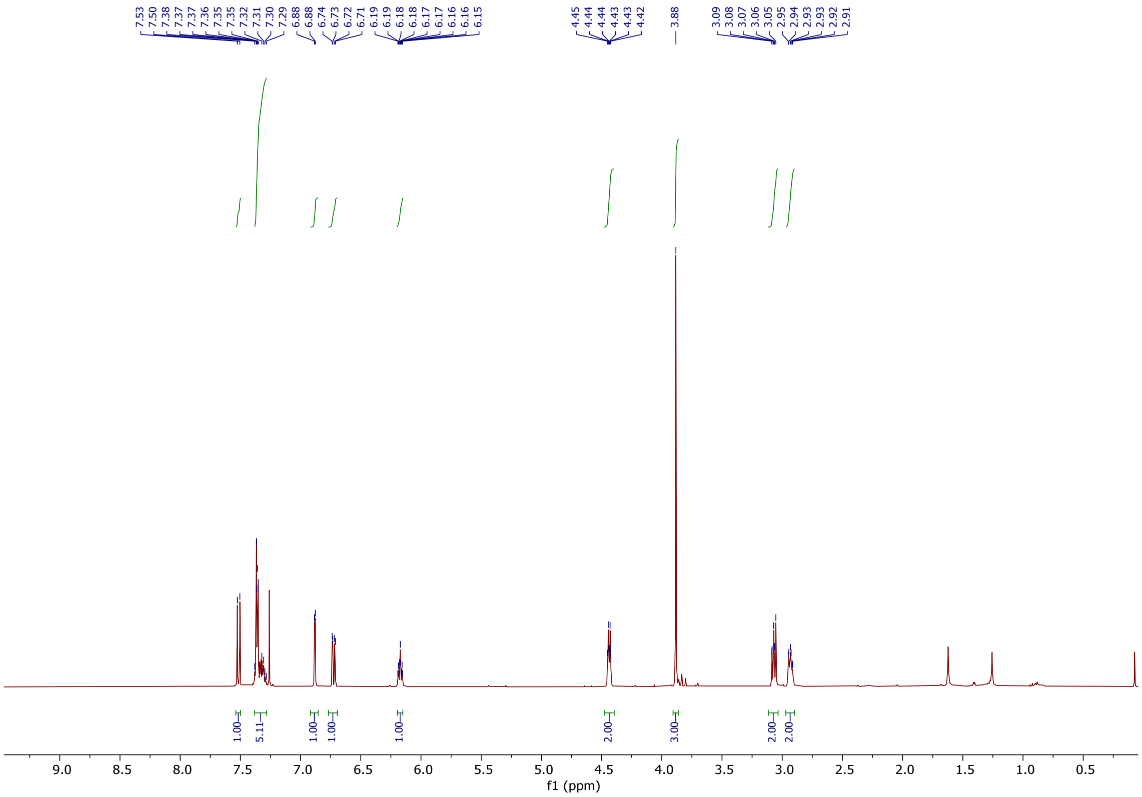

^{1}H NMR (400 MHz, $CDCl_3$) δ 7.51 (d, J = 8.4 Hz, 1H), 7.38 – 7.28 (m, 5H), 6.88 (d, J = 2.3 Hz, 1H), 6.72 (dd, J = 8.4, 2.3 Hz, 1H), 6.17 (tt, J = 6.0, 1.8 Hz, 1H), 4.44 (dt, J = 6.1, 2.1 Hz, 2H), 3.88 (s, 3H), 3.12 – 3.03 (m, 2H), 2.97 – 2.89 (m, 2H).

^{13}C NMR spectrum ($CDCl_3$)

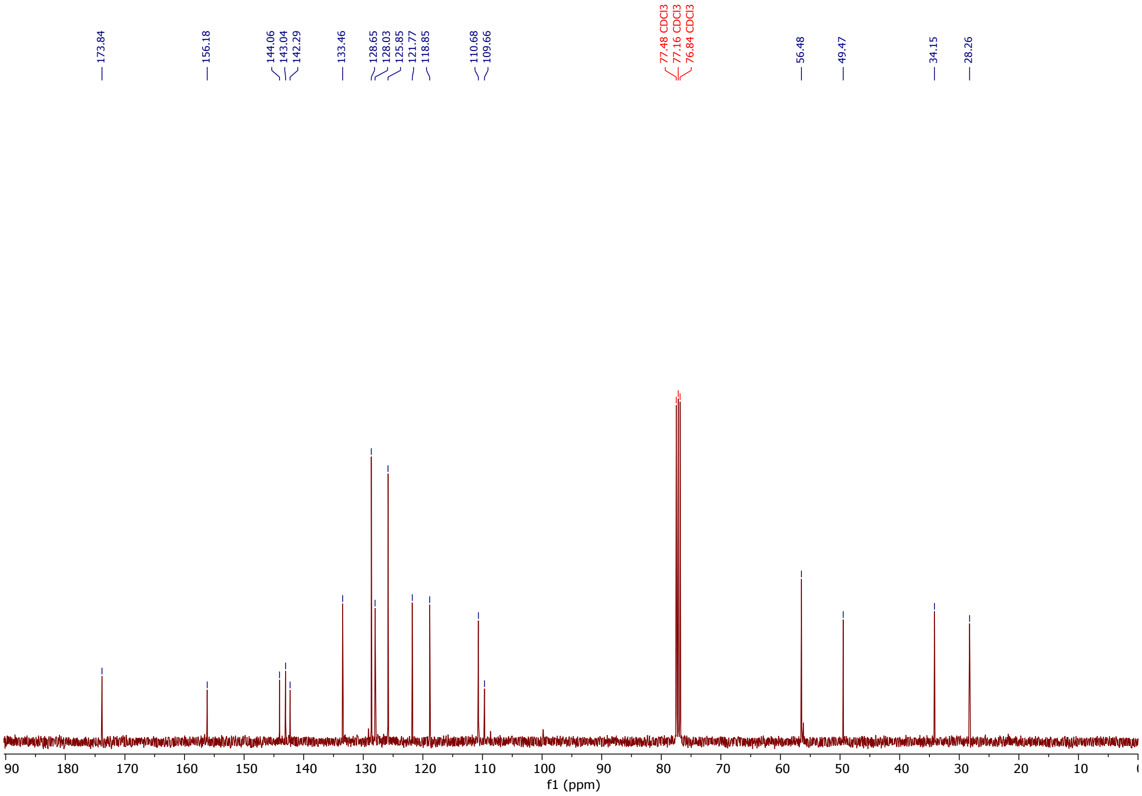

^{13}C NMR (101 MHz, $CDCl_3$) δ 173.84, 156.18, 144.06, 143.04, 142.29, 133.46, 128.65, 128.03, 125.85, 121.77, 118.85, 110.68, 109.66, 56.48, 49.47, 34.15, 28.26.

IR spectrum (neat)

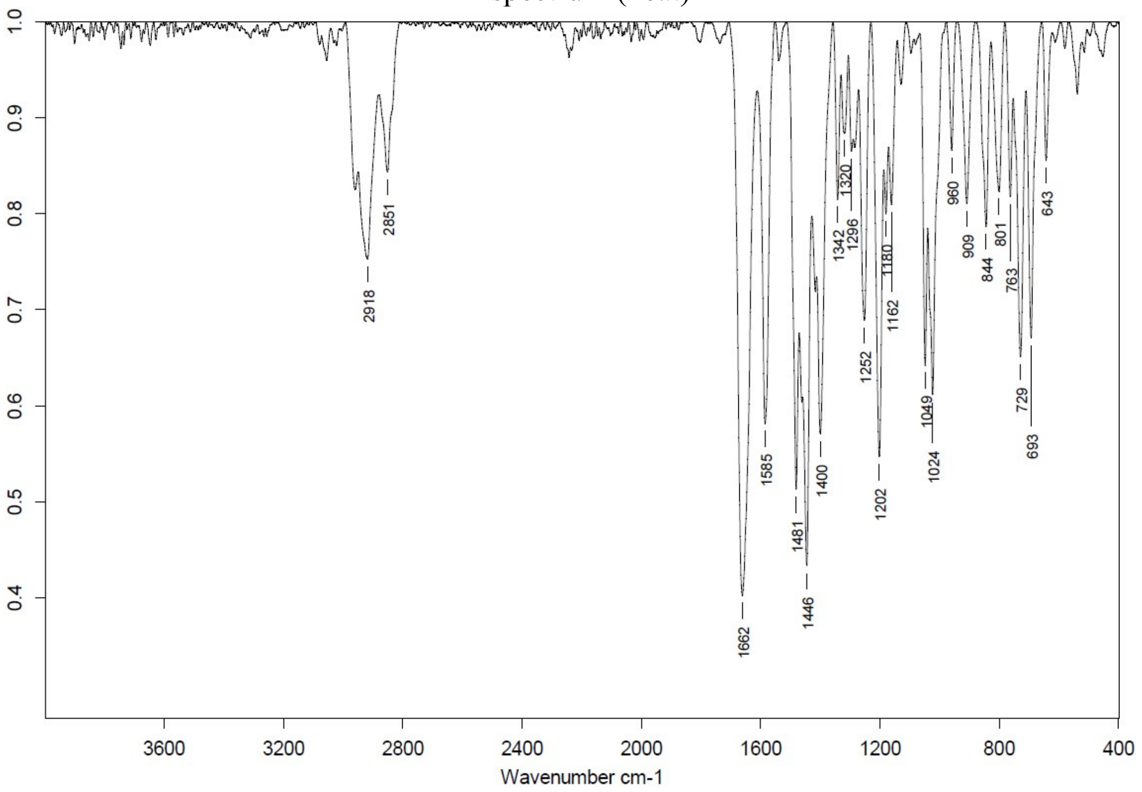

HRMS (ESI$^+$, MeOH): *m/z* calcd. 372.0594 $(M + H)^+$, found: 372.0599.

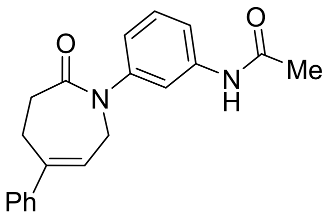

Compound 40

Scale: 0.2 mmol; *E/Z*>99:1, isolated 45.5 mg (71% yield), yellow solid, Hexane : EA = 1 : 1, R_f = 0.15

^{1}H NMR spectrum ($CDCl_3$)

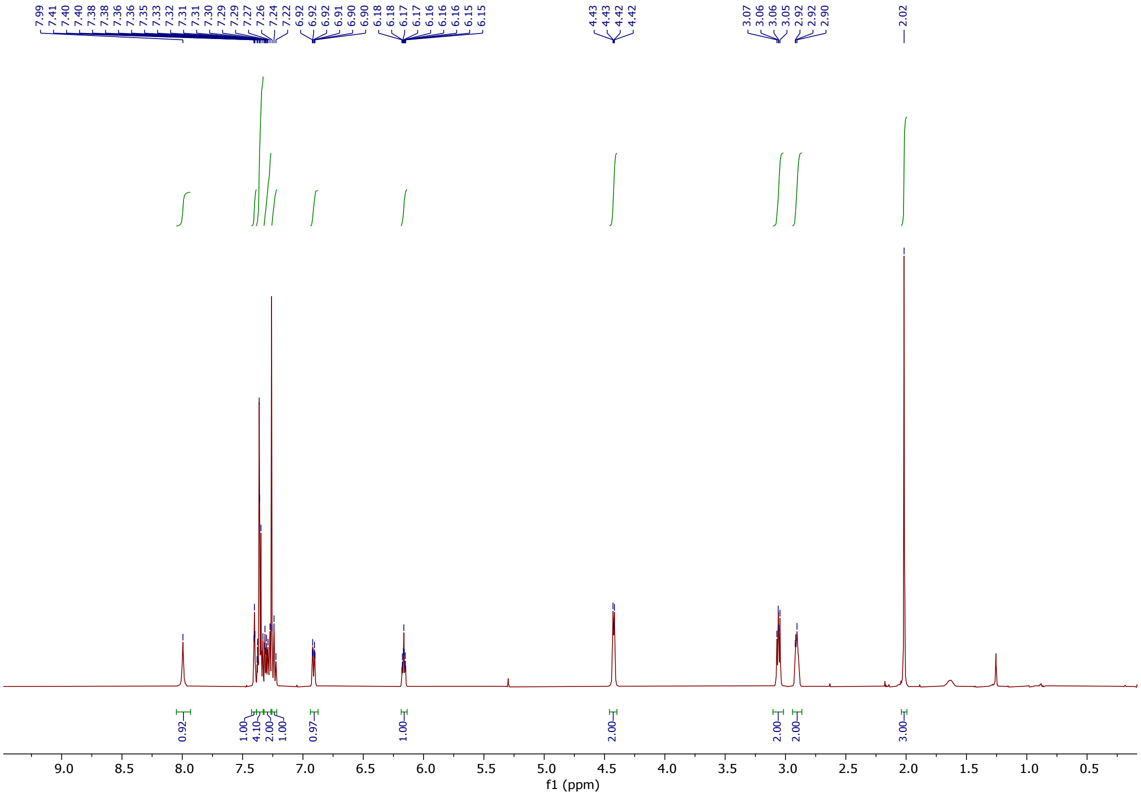

^{1}H NMR (500 MHz, $CDCl_3$) δ 7.99 (s, 1H), 7.40 (t, *J* = 2.1 Hz, 1H), 7.39 – 7.33 (m, 4H), 7.32 – 7.27 (m, 2H), 7.24 (t, *J* = 7.9 Hz, 1H), 6.91 (dt, *J* = 7.6, 1.6 Hz, 1H), 6.16 (tt, *J* = 6.1, 2.0 Hz, 1H), 4.46 – 4.40 (m, 2H), 3.09 – 3.01 (m, 2H), 2.94 – 2.86 (m, 2H), 2.02 (s, 3H).

^{13}C NMR spectrum ($CDCl_3$)

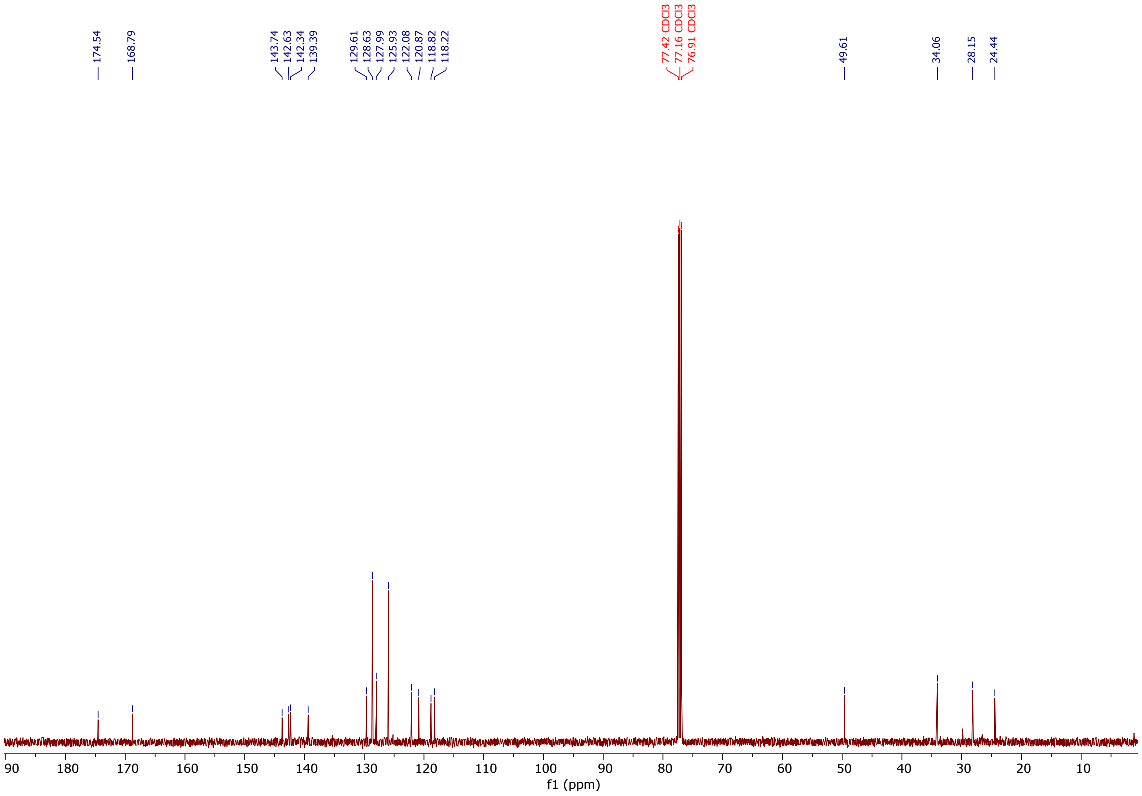

^{13}C NMR (126 MHz, $CDCl_3$) δ 174.54, 168.79, 143.74, 142.63, 142.34, 139.39, 129.61, 128.63, 127.99, 125.93, 122.08, 120.87, 118.82, 118.22, 49.61, 34.06, 28.15, 24.44.

IR spectrum (neat)

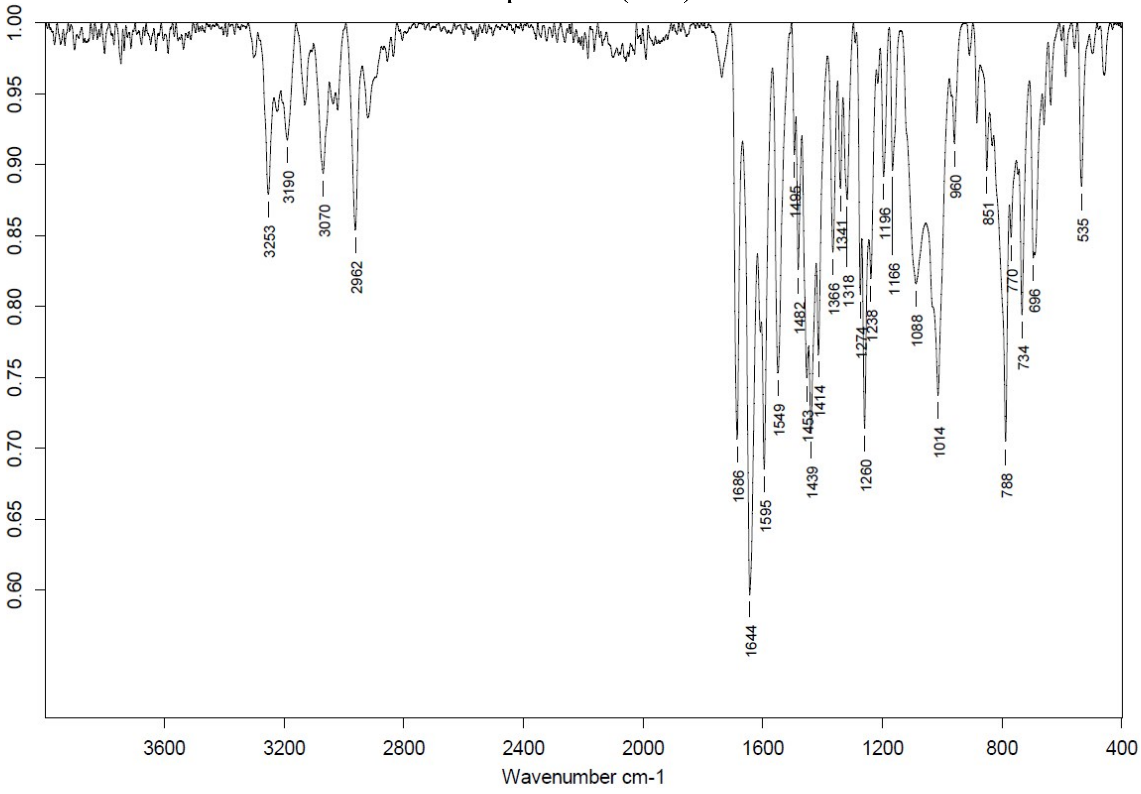

HRMS (ESI$^+$, MeOH): *m/z* calcd. 321.1598 $(M + H)^+$, found: 321.1604.

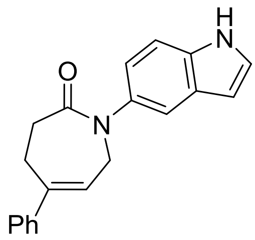

Compound 41

Scale: 0.2 mmol; *E/Z*>99:1, isolated 36.3 mg (60% yield), yellow solid, Hexane : EA = 1 : 2, R_f = 0.15

^{1}H NMR spectrum ($CDCl_3$)

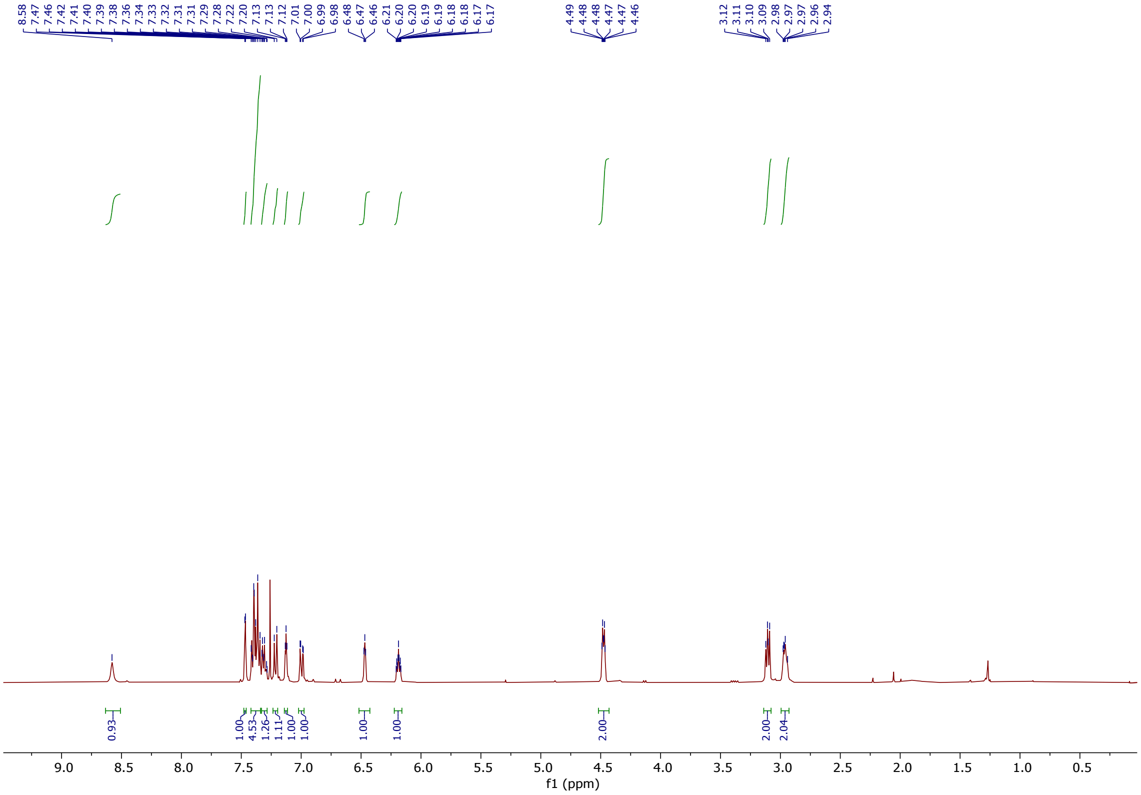

^{1}H NMR (400 MHz, $CDCl_3$) δ 8.58 (s, 1H), 7.47 (d, *J* = 2.1 Hz, 1H), 7.42 – 7.34 (m, 4H), 7.33 – 7.28 (m, 1H), 7.24 – 7.19 (m, 1H), 7.13 (t, *J* = 2.8 Hz, 1H), 7.02 – 6.96 (m, 1H), 6.47 (t, *J* = 2.8 Hz, 1H), 6.19 (tt, *J* = 6.0, 1.9 Hz, 1H), 4.48 (dt, *J* = 6.1, 2.1 Hz, 2H), 3.14 – 3.07 (m, 2H), 3.00 – 2.92 (m, 2H).

^{13}C NMR spectrum ($CDCl_3$)

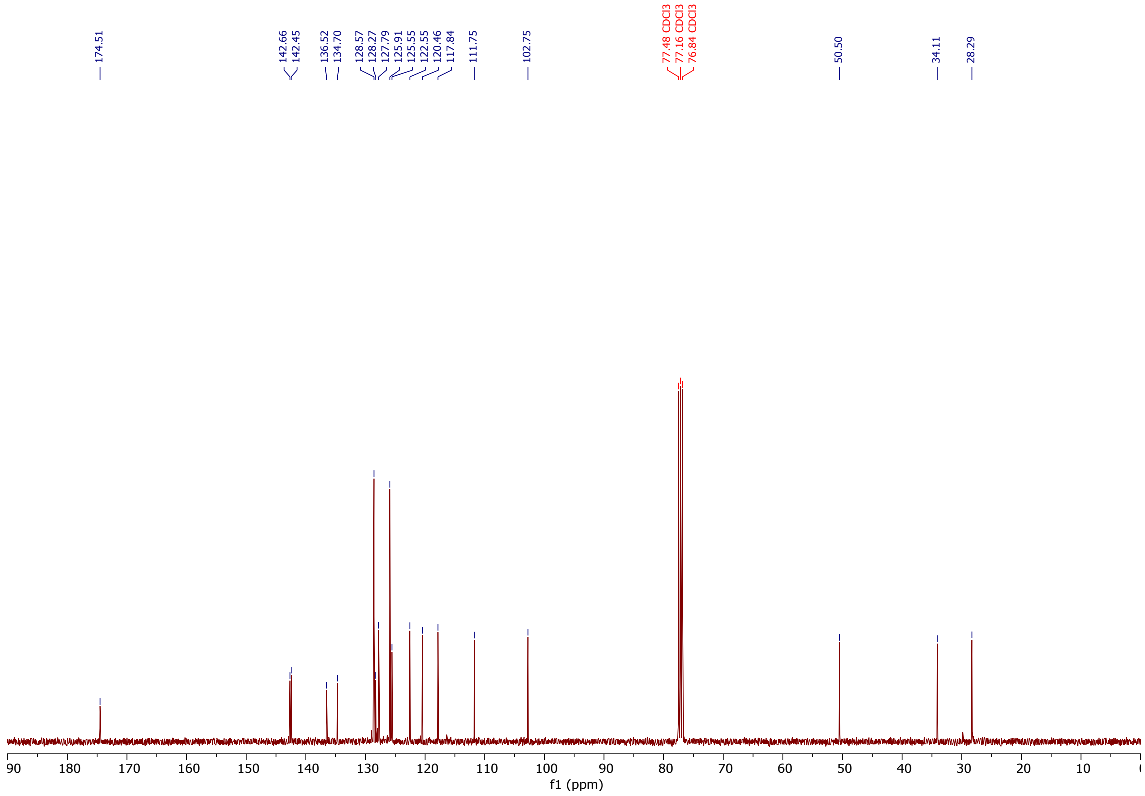

^{13}C NMR (101 MHz, $CDCl_3$) δ 174.51, 142.66, 142.45, 136.52, 134.70, 128.57, 128.27, 127.79, 125.91, 125.55, 122.55, 120.46, 117.84, 111.75, 102.75, 50.50, 34.11, 28.29.

IR spectrum (neat)

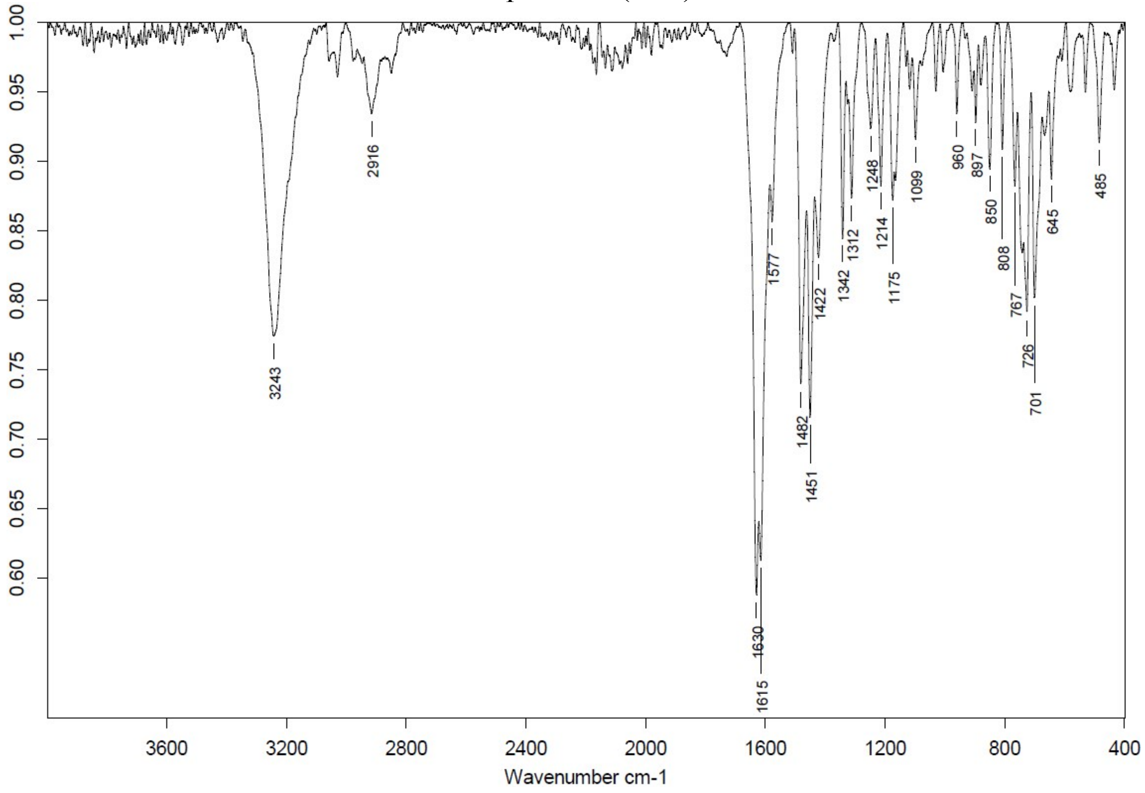

HRMS (ESI$^+$, MeOH): *m/z* calcd. 303.1492 $(M + H)^+$, found: 303.1494.

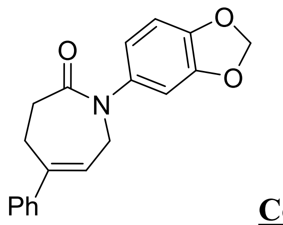

Compound 42

Scale: 0.2 mmol; *E/Z* = 95:5, isolated 45.5 mg (74% yield), yellow solid, Hexane : EA = 1 : 1, R_f = 0.15

^{1}H NMR spectrum ($CDCl_3$)

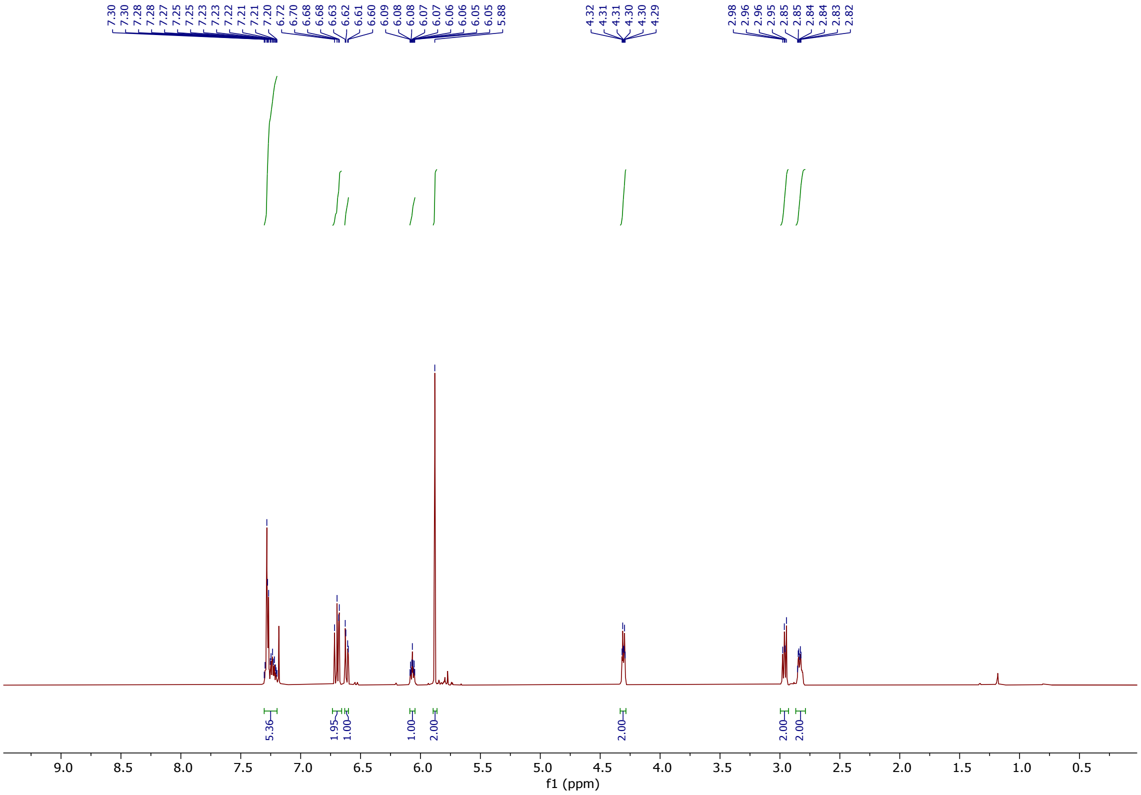

^{1}H NMR (400 MHz, $CDCl_3$) δ 7.30 – 7.20 (m, 5H), 6.73 – 6.66 (m, 2H), 6.63 – 6.60 (m, 1H), 6.07 (tt, J = 6.0, 1.8 Hz, 1H), 5.88 (s, 2H), 4.30 (dt, J = 6.1, 2.1 Hz, 2H), 2.99 – 2.92 (m, 2H), 2.86 – 2.78 (m, 2H).

^{13}C NMR spectrum ($CDCl_3$)

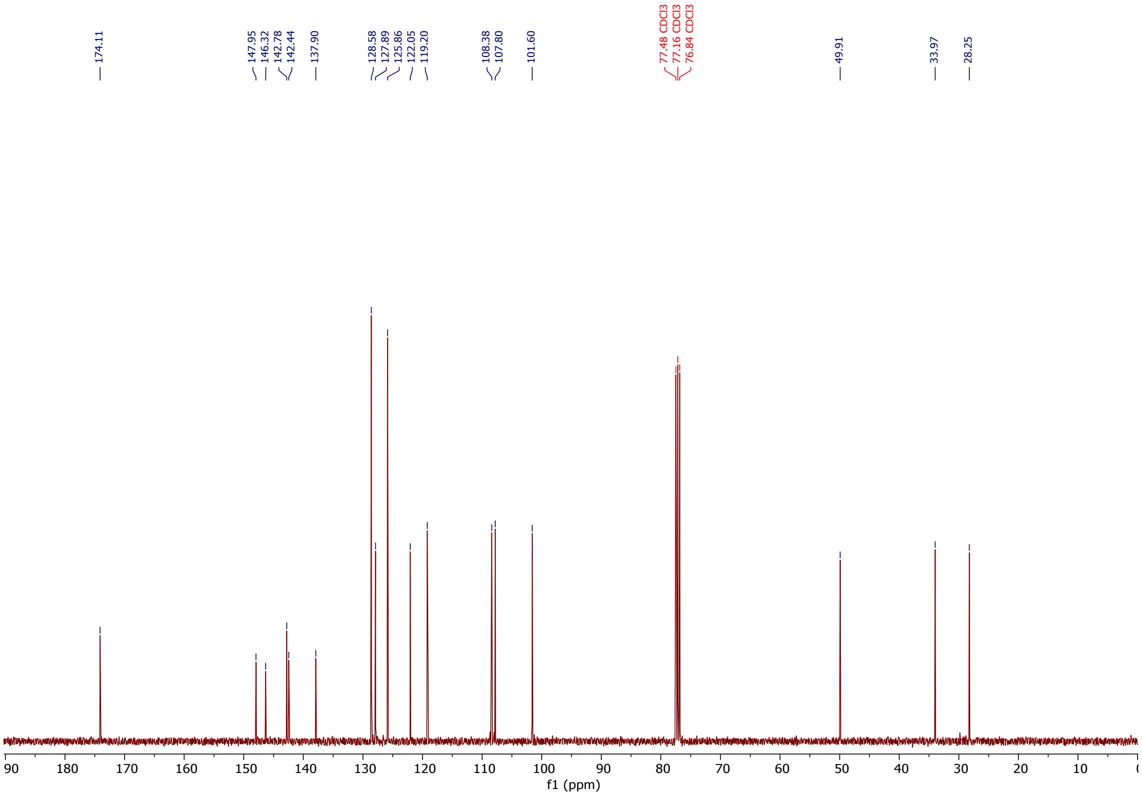

^{13}C NMR (101 MHz, $CDCl_3$) δ 174.11, 147.95, 146.32, 142.78, 142.44, 137.90, 128.58, 127.89, 125.86, 122.05, 119.20, 108.38, 107.80, 101.60, 49.91, 33.97, 28.25.

IR spectrum (neat)

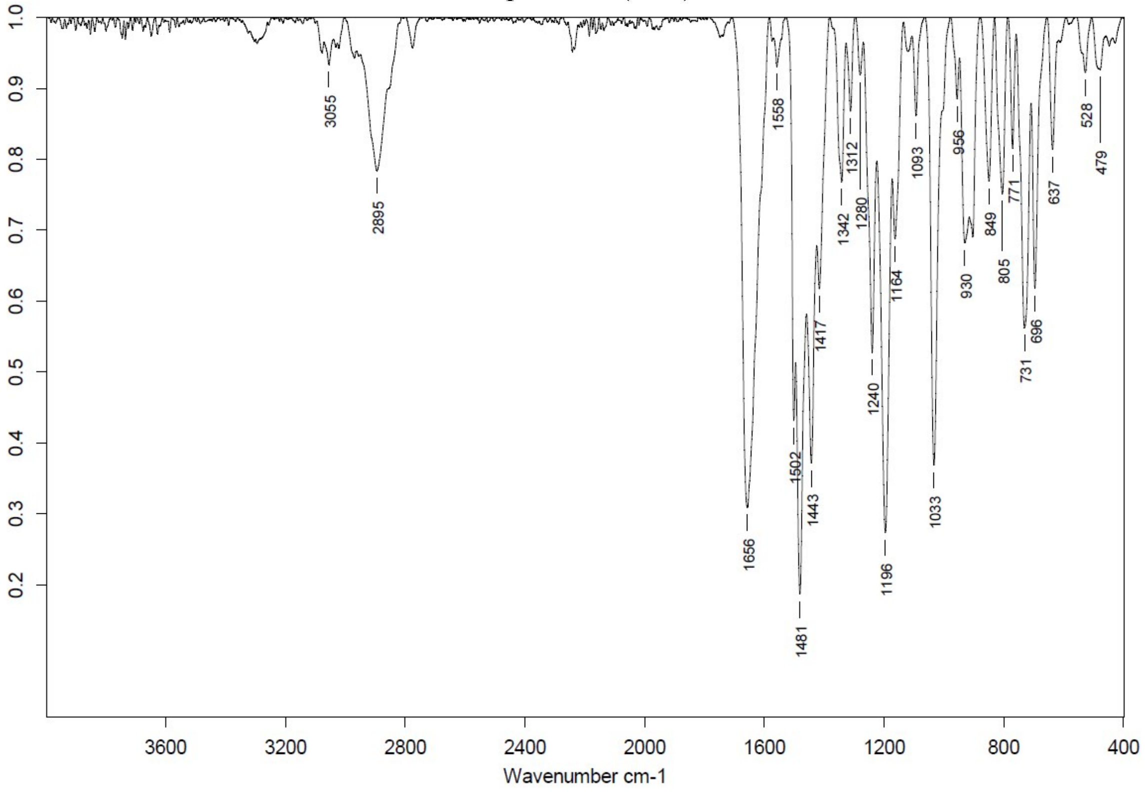

HRMS (ESI$^+$, MeOH): *m/z* calcd. 308.1281 $(M + H)^+$, found: 308.1273.

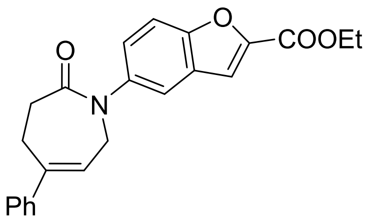

Compound 43

Scale: 0.2 mmol; *E/Z* = 95:5, isolated 60.8 mg (81% yield), yellow solid, Hexane : EA = 1 : 1, R_f = 0.15

^{1}H NMR spectrum ($CDCl_3$)

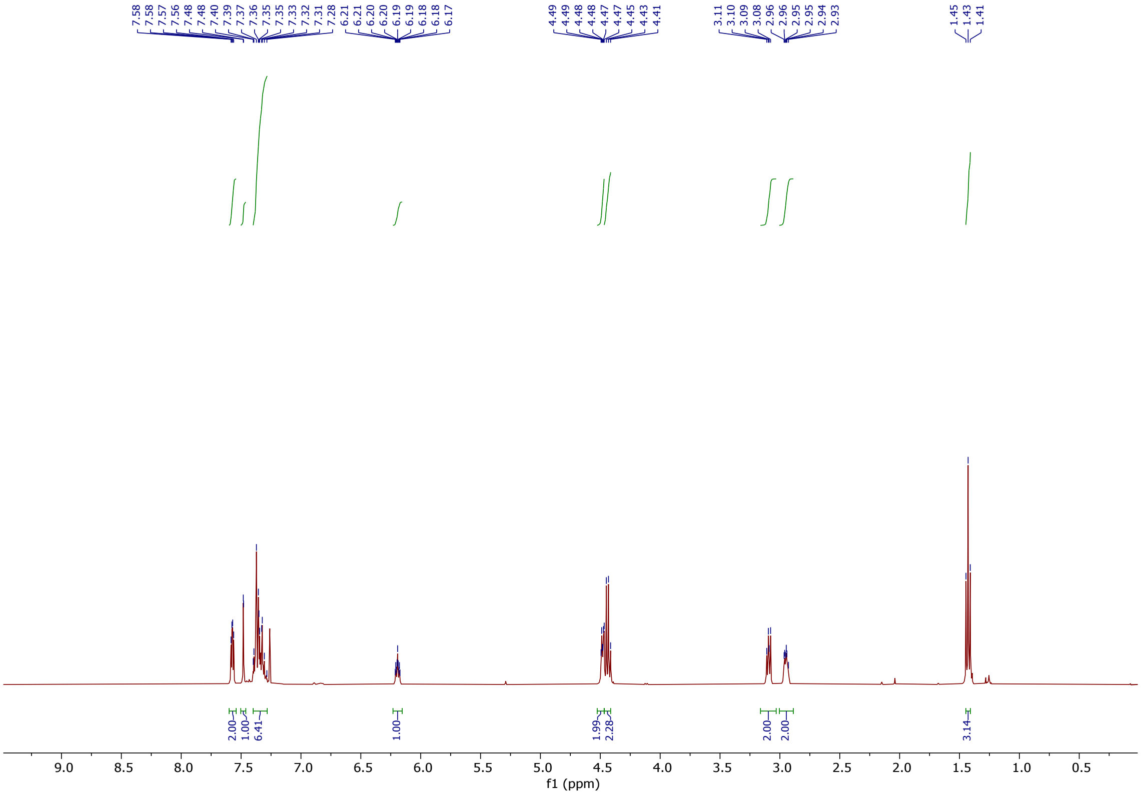

^{1}H NMR (400 MHz, $CDCl_3$) δ 7.59 – 7.54 (m, 2H), 7.48 (d, *J* = 0.9 Hz, 1H), 7.40 – 7.28 (m, 6H), 6.19 (tt, *J* = 6.1, 1.8 Hz, 1H), 4.53 – 4.47 (m, 2H), 4.44 (q, *J* = 7.1 Hz, 2H), 3.16 – 3.02 (m, 2H), 3.00 – 2.88 (m, 2H), 1.43 (t, *J* = 7.1 Hz, 3H).

^{13}C NMR spectrum ($CDCl_3$)

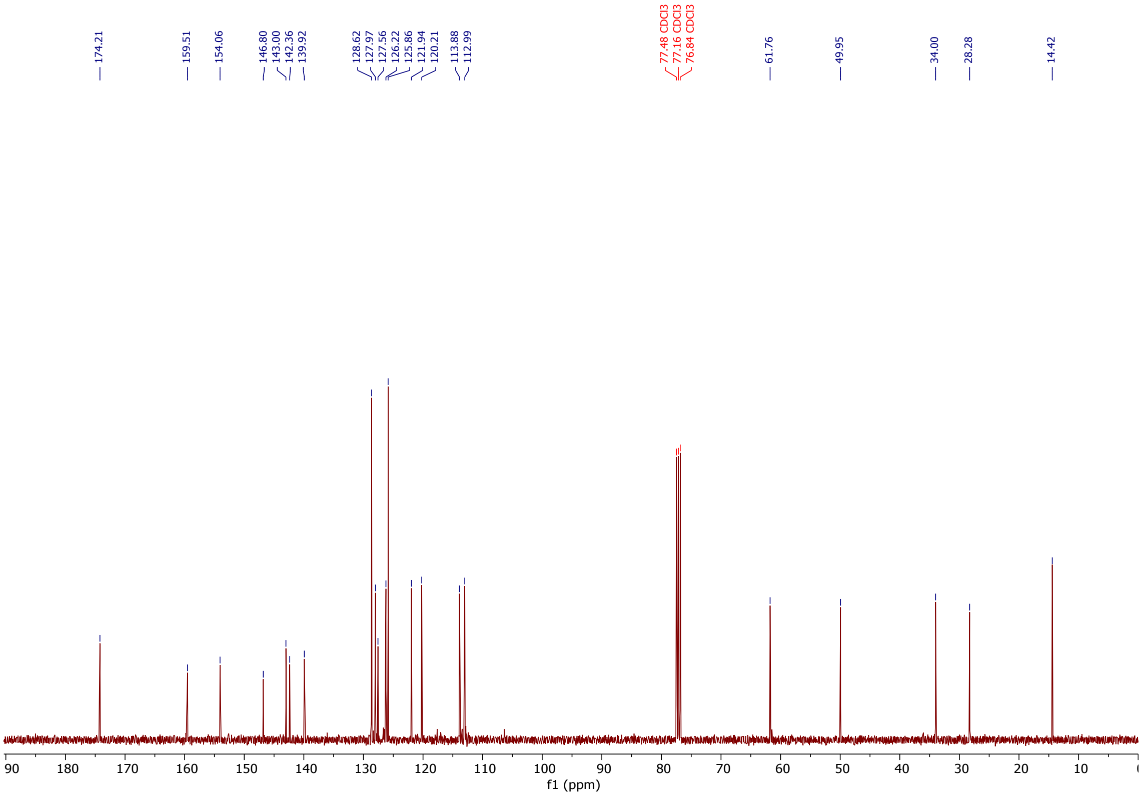

^{13}C NMR (101 MHz, $CDCl_3$) δ 174.21, 159.51, 154.06, 146.80, 143.00, 142.36, 139.92, 128.62, 127.97, 127.56, 126.22, 125.86, 121.94, 120.21, 113.88, 112.99, 61.76, 49.95, 34.00, 28.28, 14.42.

IR spectrum (neat)

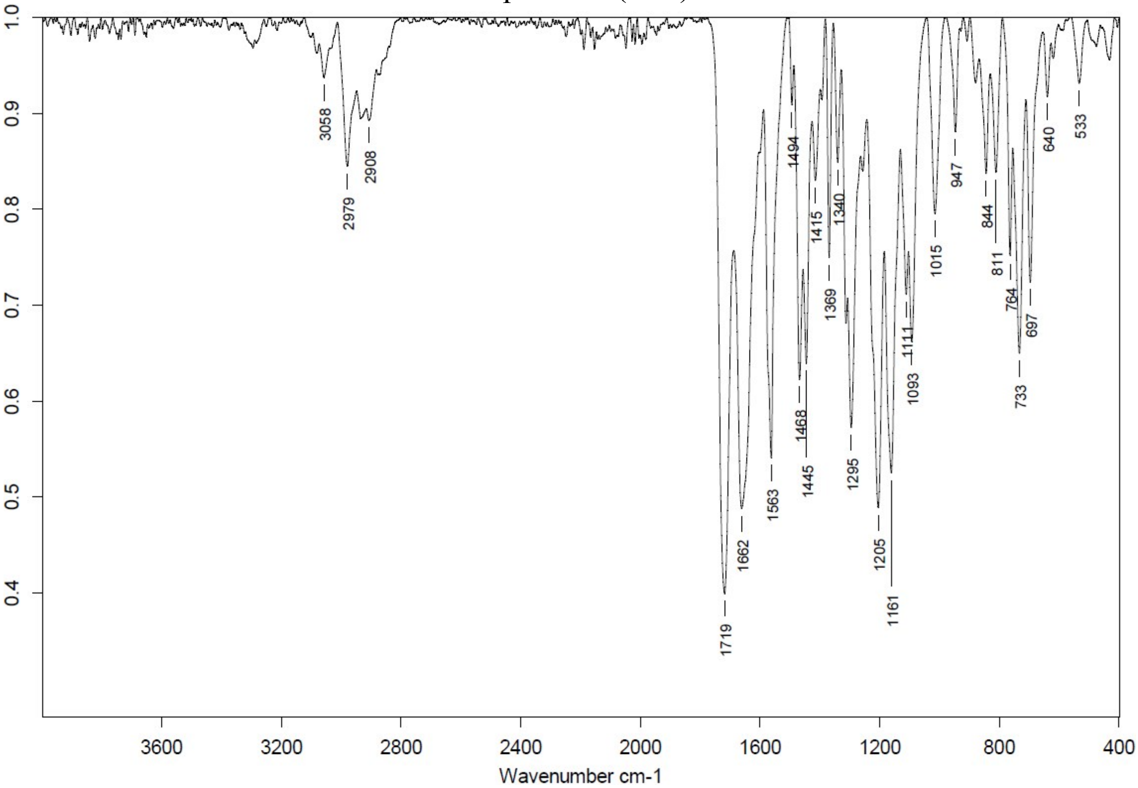

HRMS (ESI$^+$, MeOH): *m/z* calcd. 398.1363 (M + Na)$^+$, found: 398.1363.

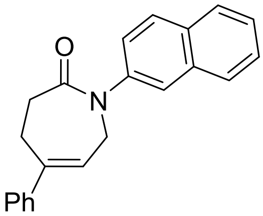

Compound 44

Scale: 0.2 mmol; *E/Z*>99:1, isolated 44.5 mg (71% yield), yellow solid, Hexane : EA = 2 : 1, R_f = 0.20

^{1}H NMR spectrum ($CDCl_3$)

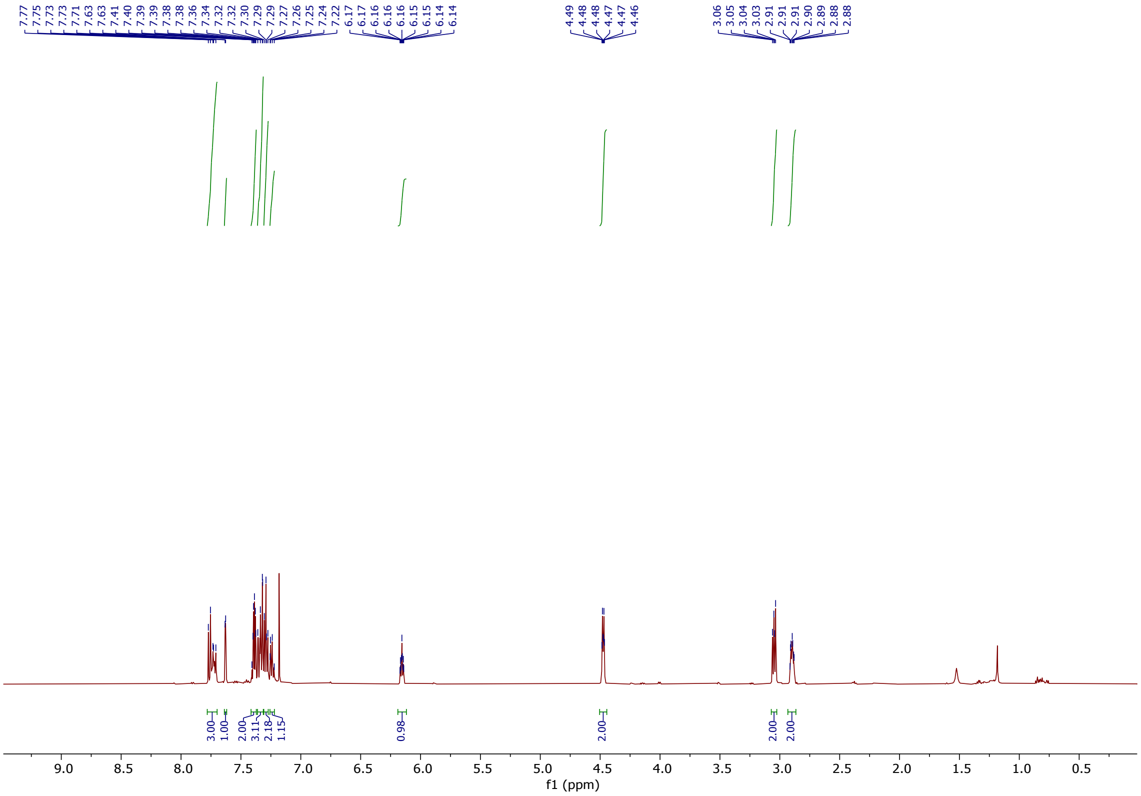

^{1}H NMR (500 MHz, $CDCl_3$) δ 7.78 – 7.70 (m, 3H), 7.63 (d, J = 2.4 Hz, 1H), 7.42 – 7.37 (m, 2H), 7.36 – 7.31 (m, 3H), 7.30 – 7.27 (m, 2H), 7.26 – 7.22 (m, 1H), 6.16 (tt, J = 6.1, 1.8 Hz, 1H), 4.47 (dt, J = 6.1, 2.1 Hz, 2H), 3.07 – 3.02 (m, 2H), 2.93 – 2.86 (m, 2H).

^{13}C NMR spectrum (CDCl$_3$)

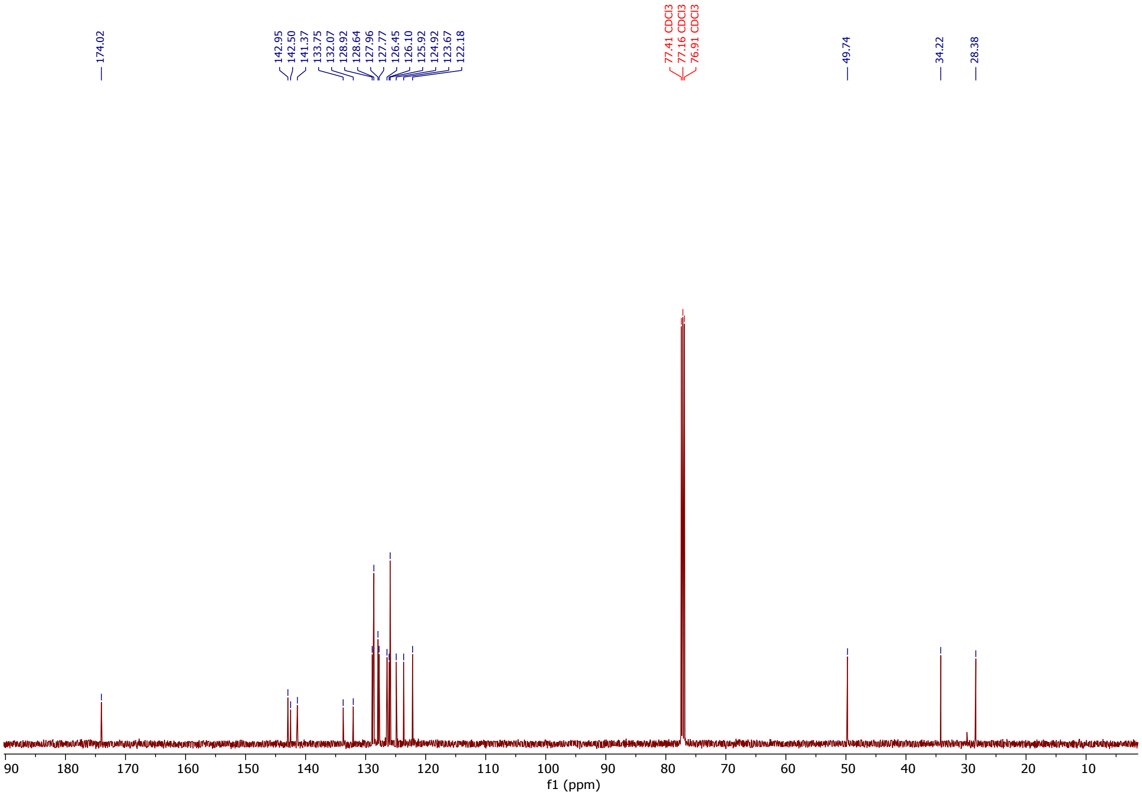

^{13}C NMR (126 MHz, CDCl$_3$) δ 174.02, 142.95, 142.50, 141.37, 133.75, 132.07, 128.92, 128.64, 127.96, 127.77, 126.45, 126.10, 125.92, 124.92, 123.67, 122.18, 49.74, 34.22, 28.38.

IR spectrum (neat)

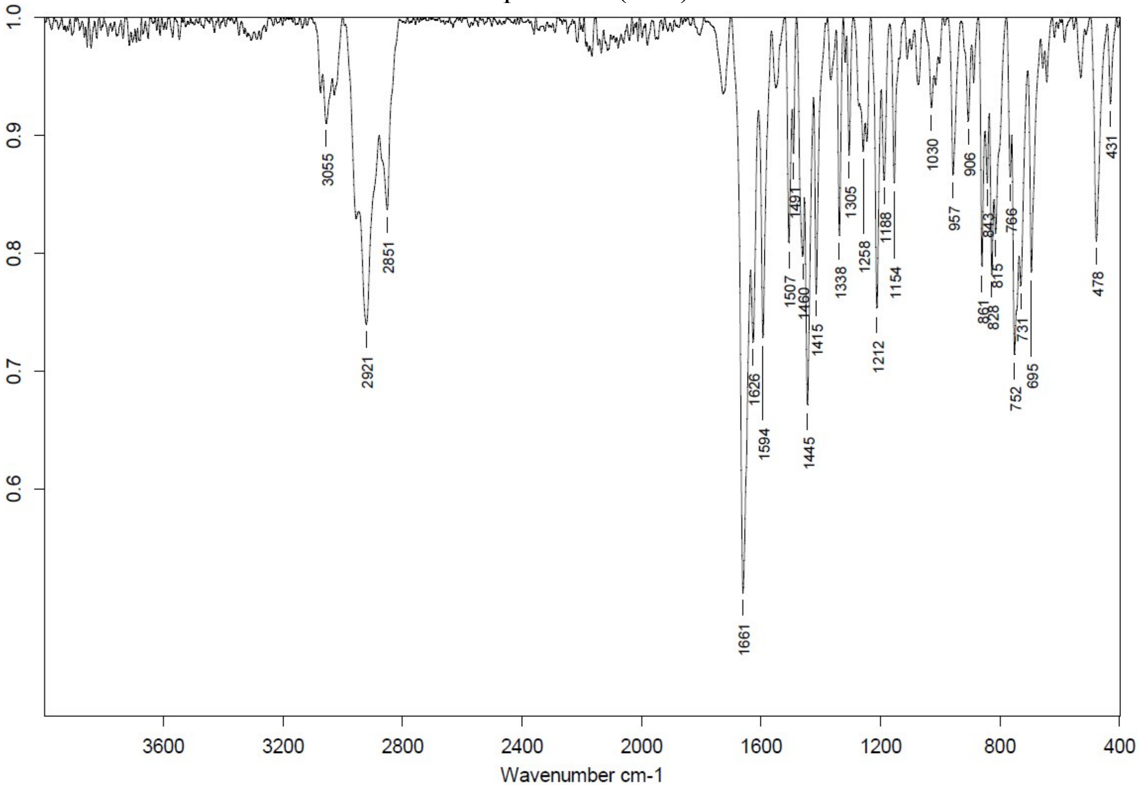

HRMS (ESI$^+$, MeOH): *m/z* calcd. 314.1539 (M + H)$^+$, found: 314.1531.

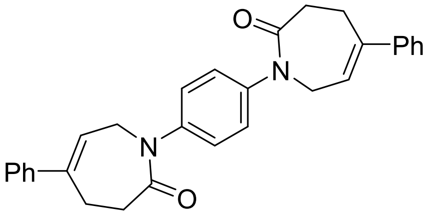

Compound 45

Scale: 0.1 mmol *p*-phenylenediamine, 0.2 mmol vinyl lactone **A**; isolated 31.0 mg (69% yield), yellow solid, DCM : MeOH = 50 : 1, R_f = 0.10

^{1}H NMR spectrum ($CDCl_3$)

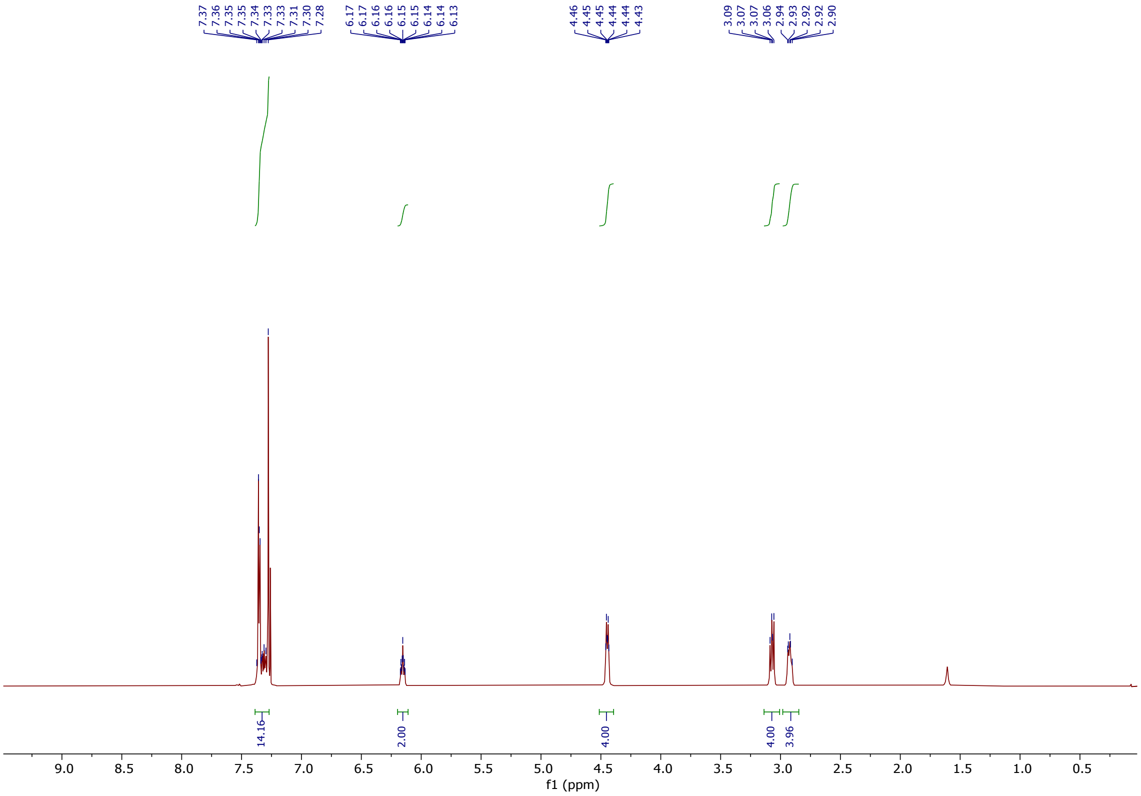

^{1}H NMR (400 MHz, $CDCl_3$) δ 7.39 – 7.27 (m, 14H), 6.15 (tt, *J* = 6.1, 1.8 Hz, 2H), 4.45 (dt, *J* = 6.1, 2.1 Hz, 4H), 3.13 – 3.00 (m, 4H), 2.97 – 2.84 (m, 4H).

^{13}C NMR spectrum ($CDCl_3$)

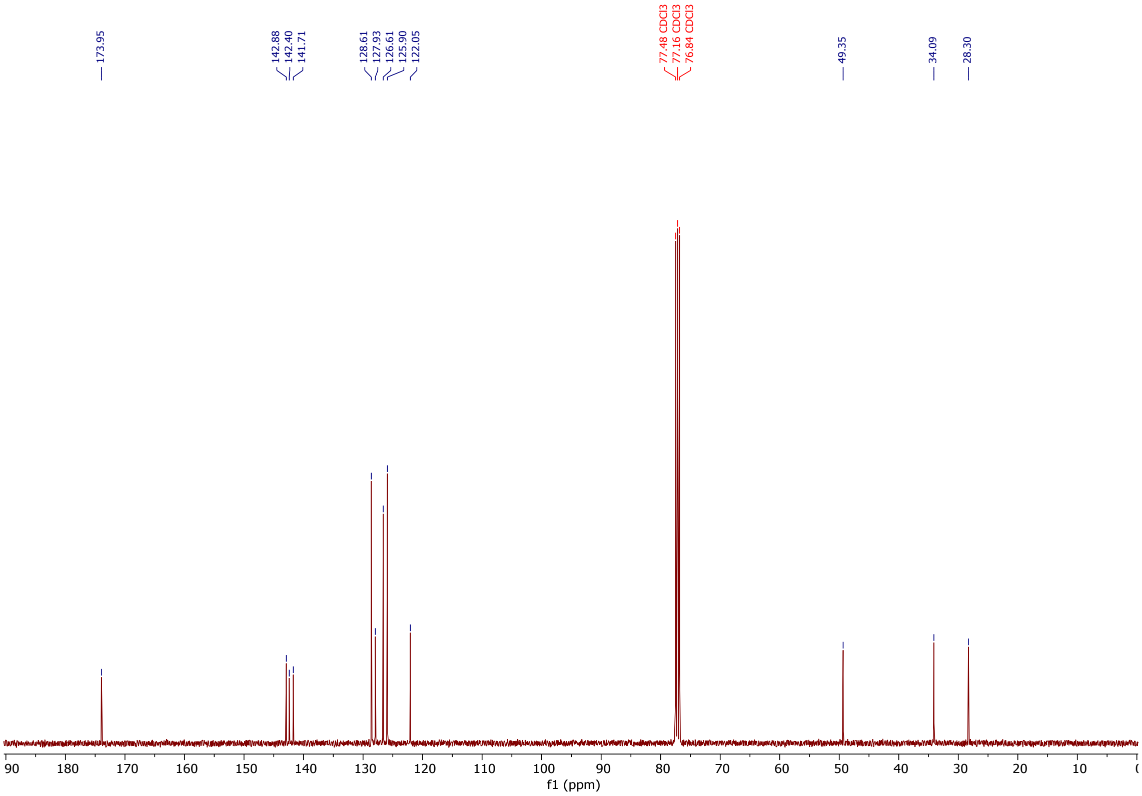

^{13}C NMR (101 MHz, $CDCl_3$) δ 173.95, 142.88, 142.40, 141.71, 128.61, 127.93, 126.61, 125.90, 122.05, 49.35, 34.09, 28.30.

IR spectrum (neat)

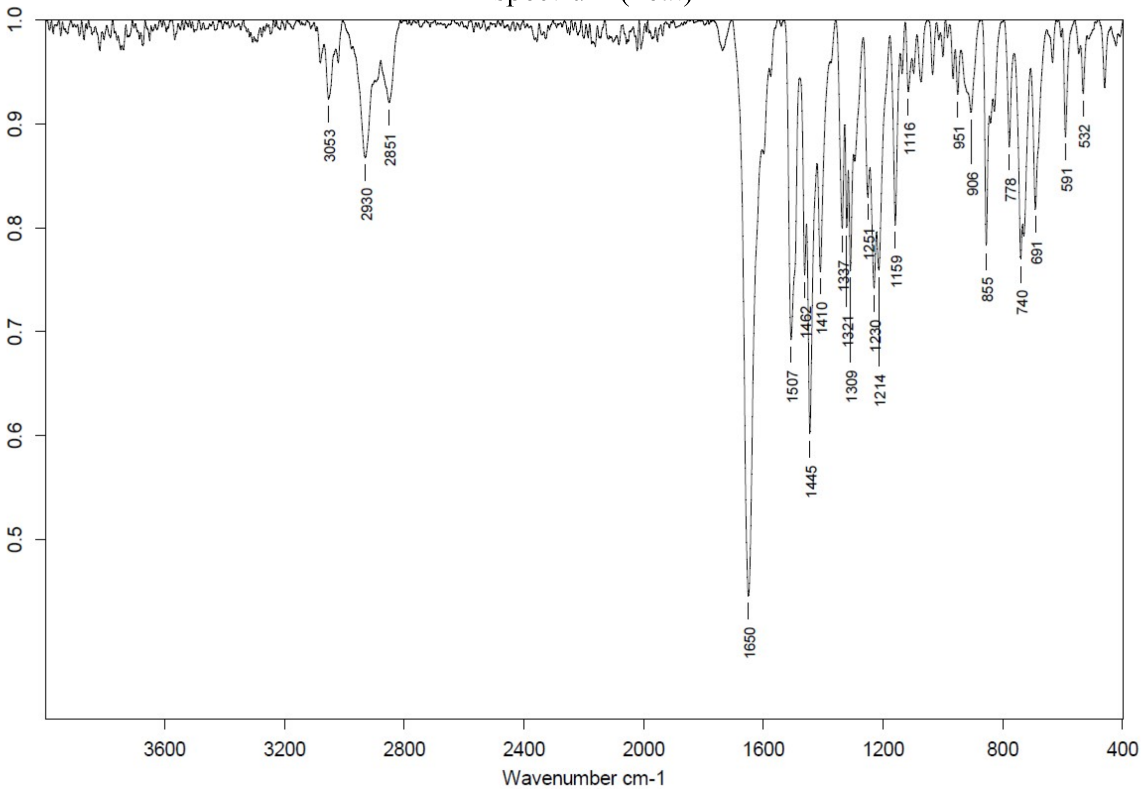

HRMS (ESI$^+$, MeOH): *m/z* calcd. 449.2224 $(M + H)^+$, found: 449.2225.

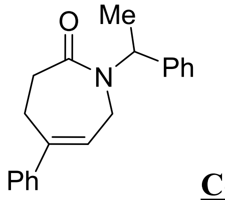

Compound 46

Scale: 0.2 mmol; *E/Z*>99:1, isolated 32.1 mg (55% yield), yellow solid, Hexane : EA = 2 : 1, R_f = 0.25

^{1}H NMR spectrum ($CDCl_3$)

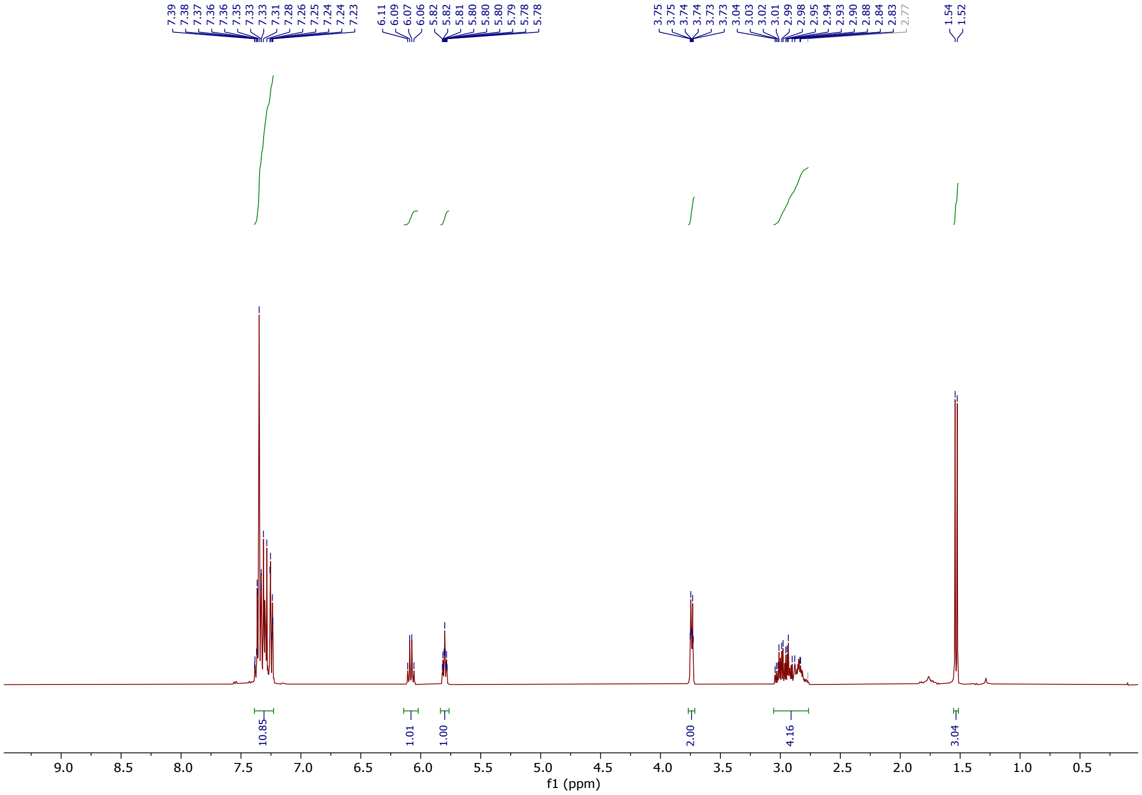

^{1}H NMR (400 MHz, $CDCl_3$) δ 7.39 – 7.23 (m, 10H), 6.08 (q, J = 7.0 Hz, 1H), 5.80 (tt, J = 6.0, 1.8 Hz, 1H), 3.74 (dt, J = 6.0, 2.2 Hz, 2H), 3.06 – 2.76 (m, 4H), 1.53 (d, J = 7.0 Hz, 3H).

^{13}C NMR spectrum ($CDCl_3$)

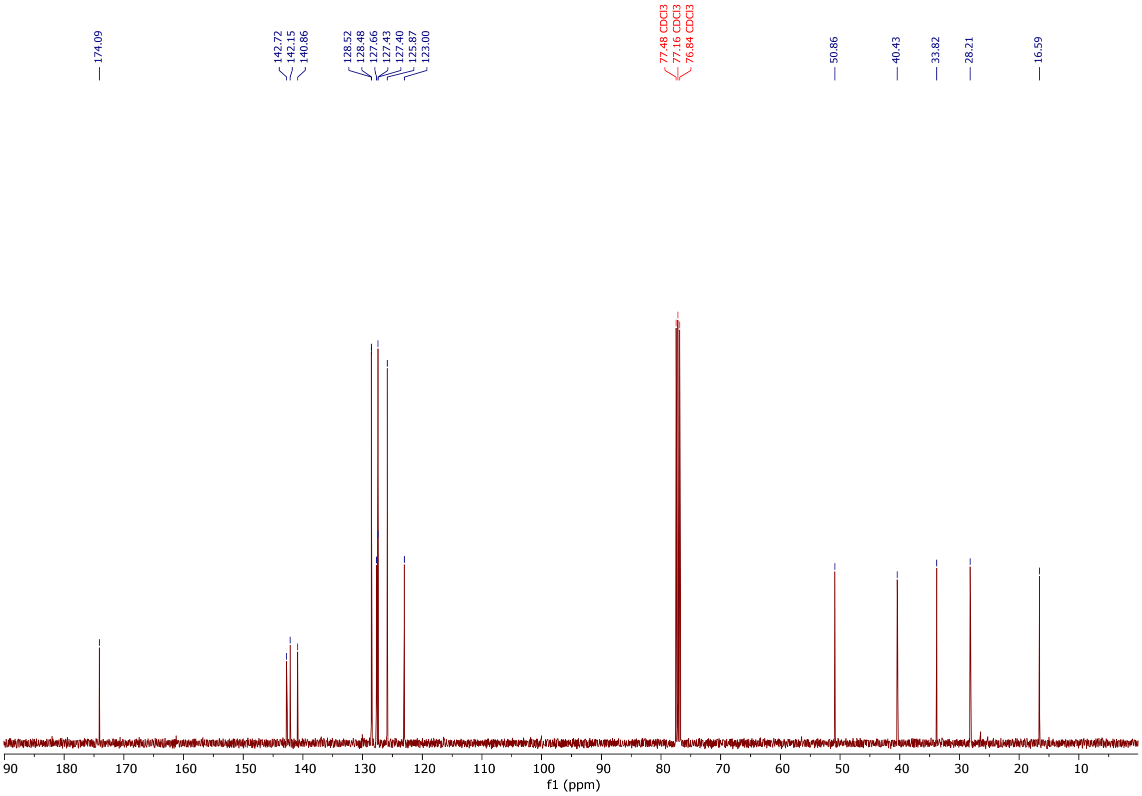

^{13}C NMR (101 MHz, $CDCl_3$) δ 174.09, 142.72, 142.15, 140.86, 128.52, 128.48, 127.66, 127.43, 127.40, 125.87, 123.00, 50.86, 40.43, 33.82, 28.21, 16.59.

IR spectrum (neat)

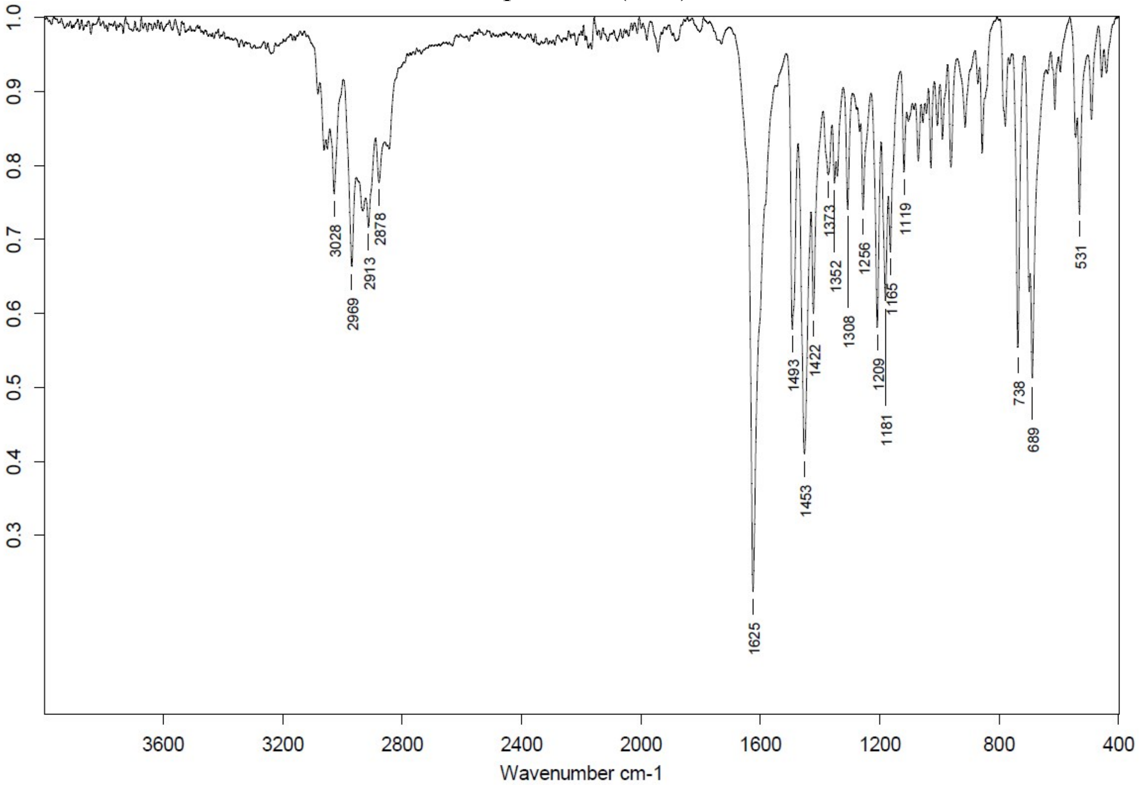

HRMS (ESI^+, MeOH): *m/z* calcd. 292.1696 $(M + H)^+$, found: 292.1700.

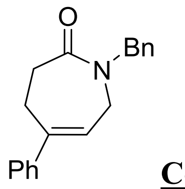

Compound 47

Scale: 0.2 mmol; *E/Z*>99:1, isolated 26.6 mg (48% yield), yellow solid, Hexane : EA = 2 : 1, R_f = 0.25

^{1}H NMR spectrum ($CDCl_3$)

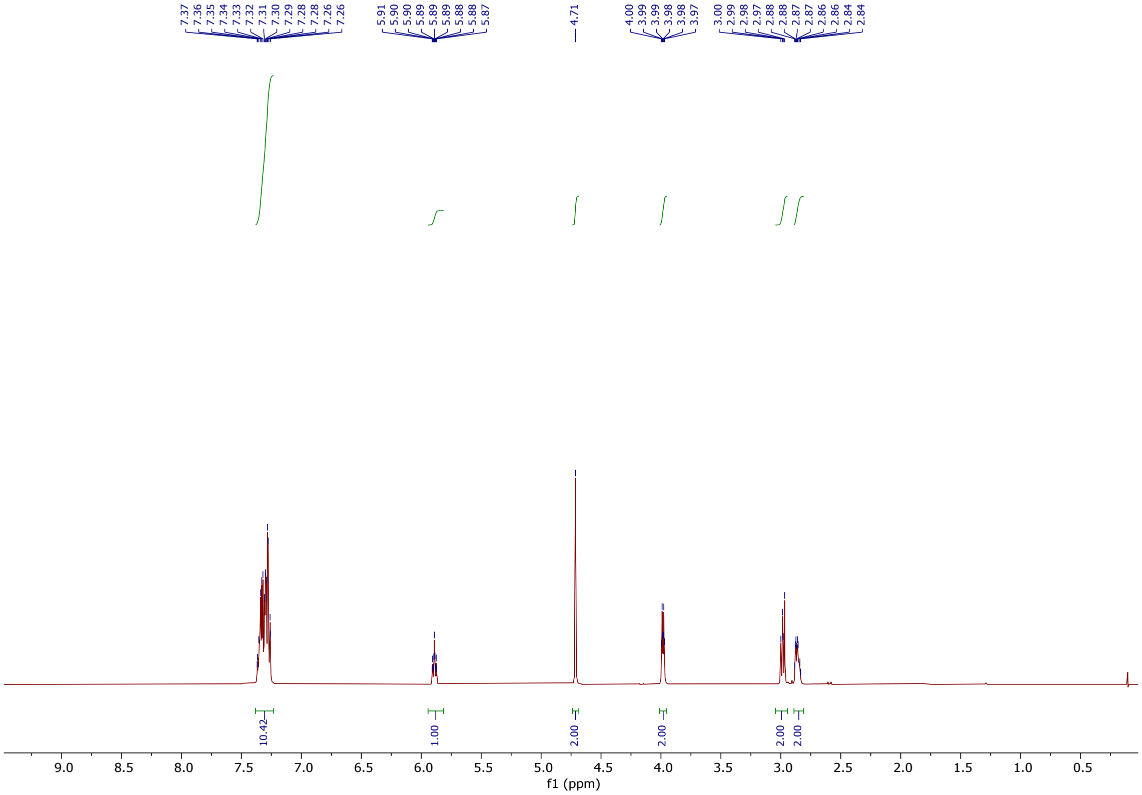

^{1}H NMR (400 MHz, $CDCl_3$) δ 7.38 – 7.23 (m, 10H), 5.89 (tt, J = 5.9, 1.8 Hz, 1H), 4.71 (s, 2H), 3.98 (dt, J = 5.9, 2.2 Hz, 2H), 3.04 – 2.94 (m, 2H), 2.89 – 2.81 (m, 2H).

^{13}C NMR spectrum ($CDCl_3$)

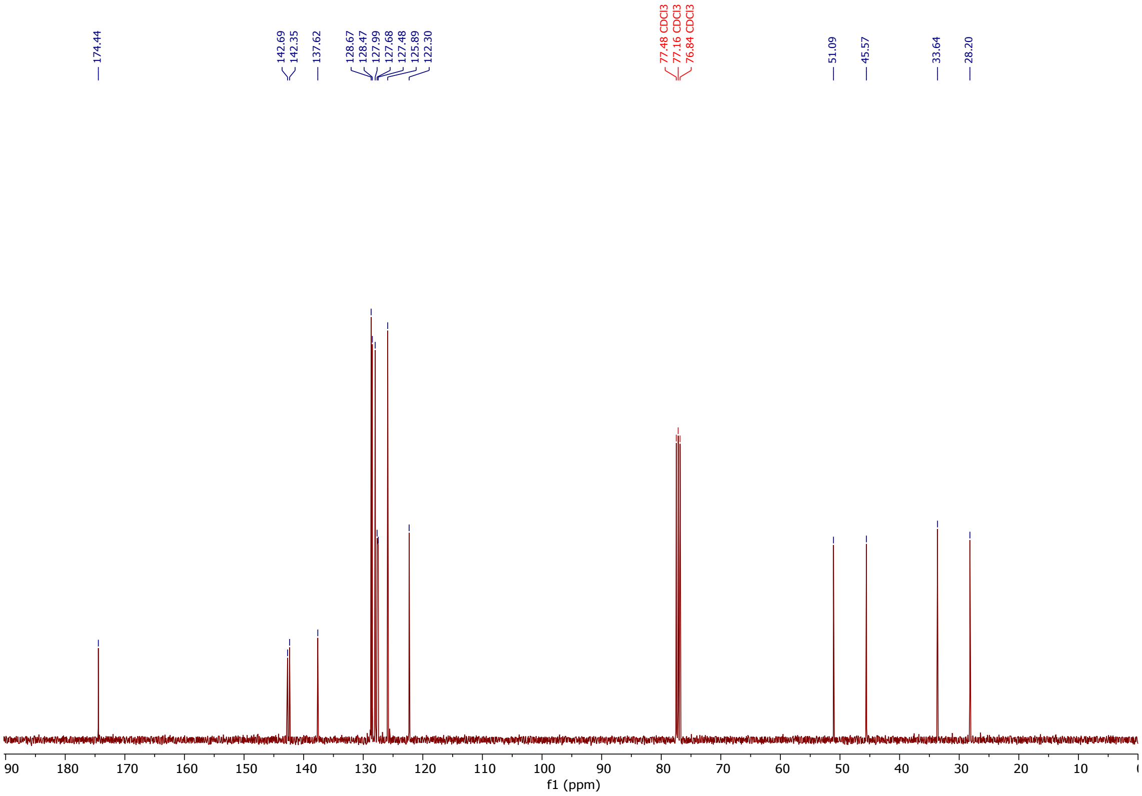

^{13}C NMR (101 MHz, $CDCl_3$) δ 174.44, 142.69, 142.35, 137.62, 128.67, 128.47, 127.99, 127.68, 127.48, 125.89, 122.30, 51.09, 45.57, 33.64, 28.20.

IR spectrum (neat)

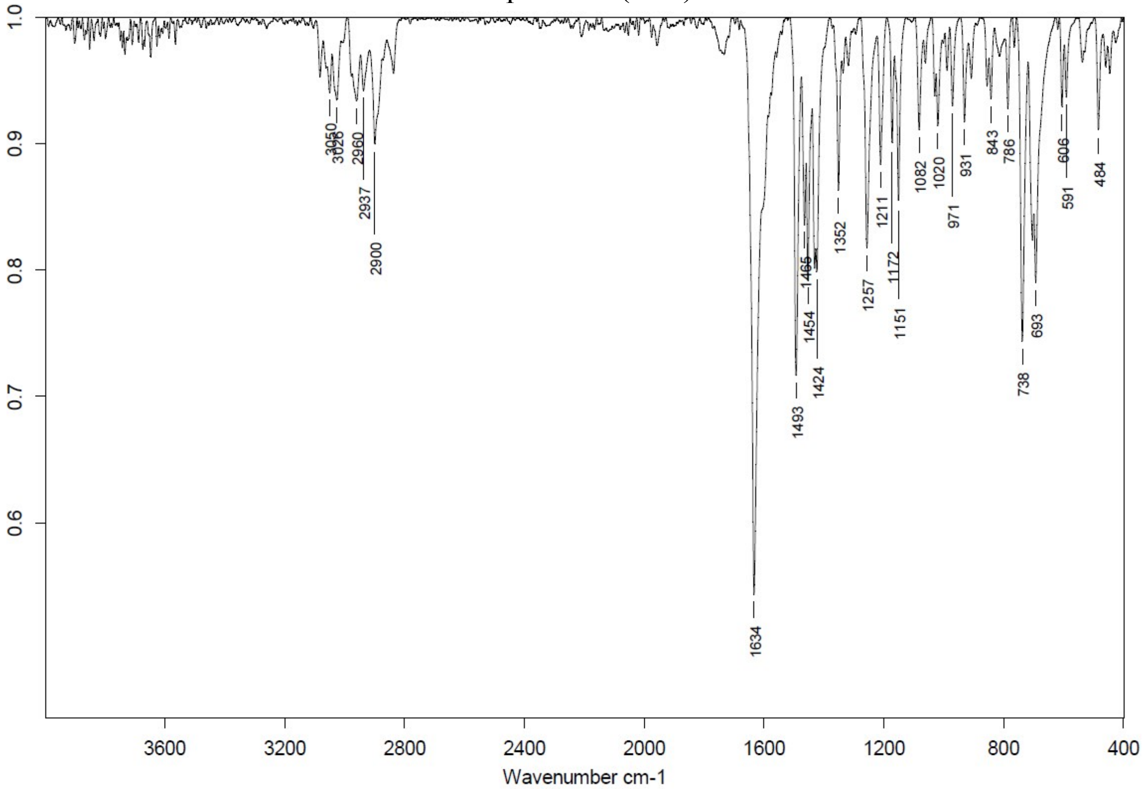

HRMS (ESI$^+$, MeOH): *m/z* calcd. 278.1539 $(M + H)^+$, found: 278.1544.

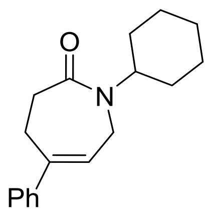

Compound 48

Scale: 0.2 mmol; *E/Z*>99:1, isolated 19.9 mg (37% yield), yellow solid, Hexane : EA = 2 : 1, R_f = 0.25

^{1}H NMR spectrum ($CDCl_3$)

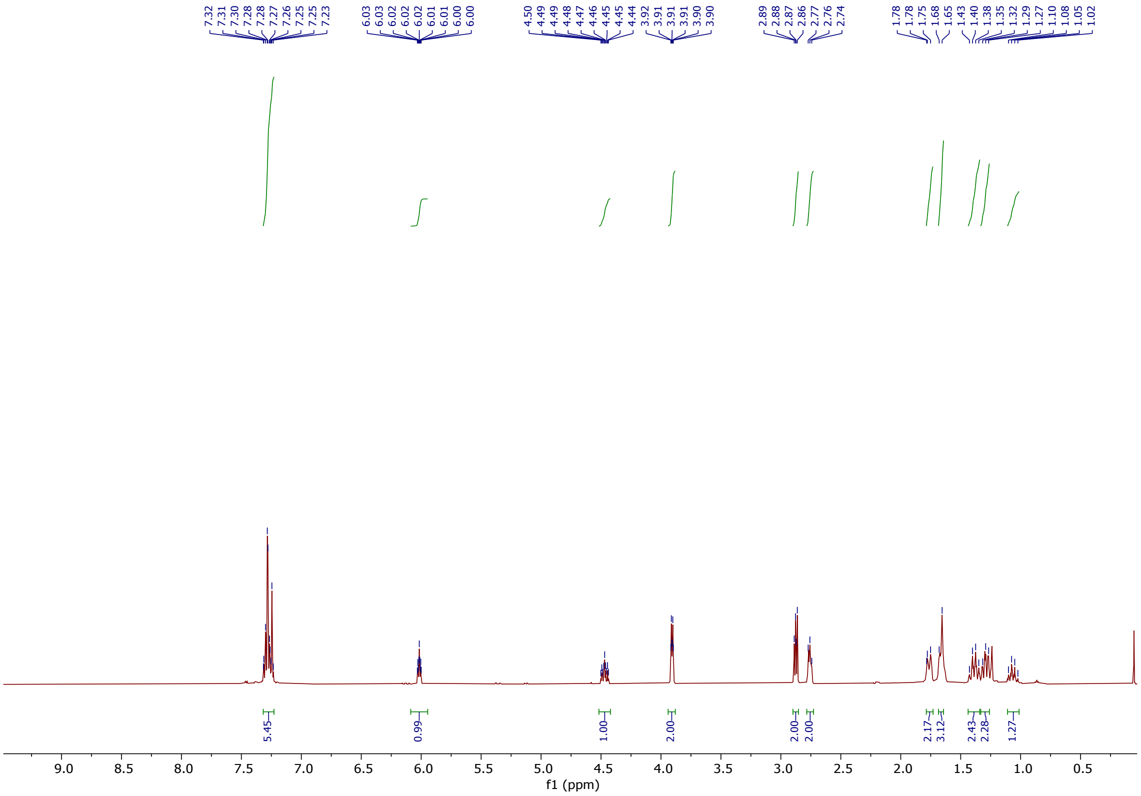

^{1}H NMR (500 MHz, $CDCl_3$) δ 7.32 – 7.23 (m, 5H), 6.02 (tt, J = 6.1, 1.9 Hz, 1H), 4.47 (tt, J = 11.8, 3.8 Hz, 1H), 3.91 (dt, J = 6.1, 2.1 Hz, 2H), 2.89 – 2.85 (m, 2H), 2.78 – 2.73 (m, 2H), 1.79 – 1.73 (m, 2H), 1.68 – 1.64 (m, 3H), 1.43 – 1.34 (m, 2H), 1.33 – 1.25 (m, 2H), 1.10 – 1.01 (m, 1H).

^{13}C NMR spectrum ($CDCl_3$)

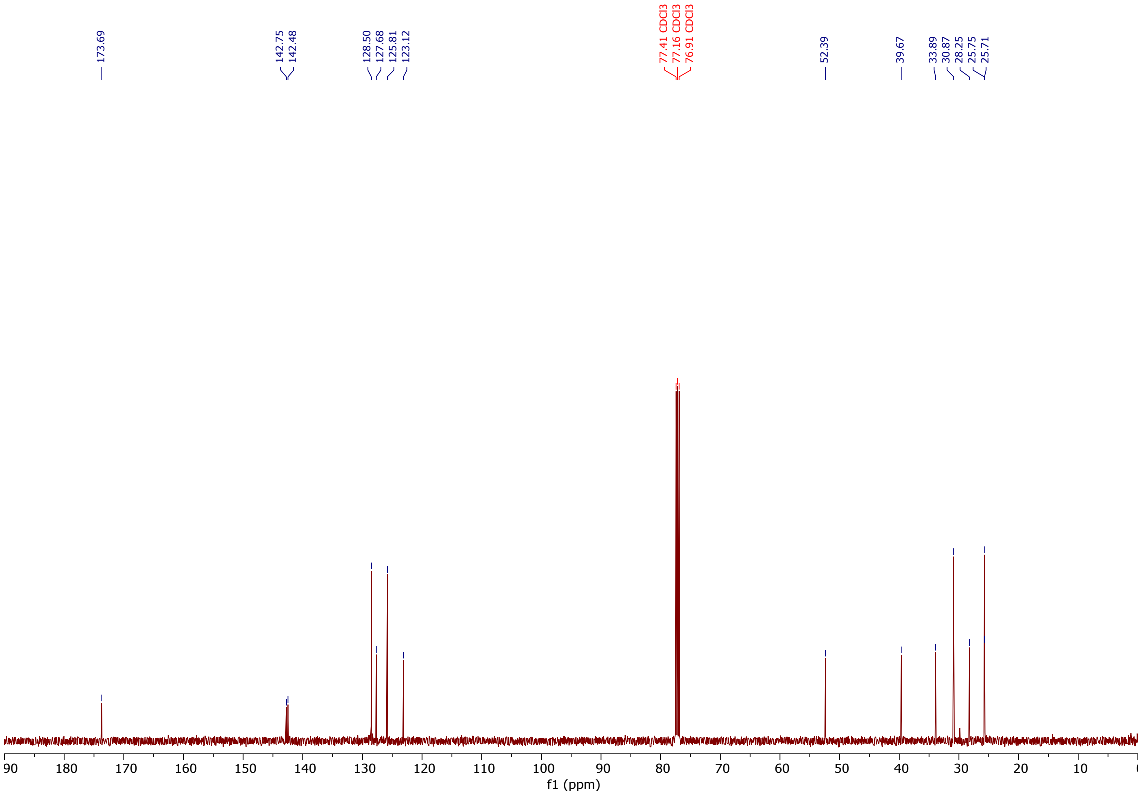

^{13}C NMR (126 MHz, $CDCl_3$) δ 173.69, 142.75, 142.48, 128.50, 127.68, 125.81, 123.12, 52.39, 39.67, 33.89, 30.87, 28.25, 25.75, 25.71.

IR spectrum (neat)

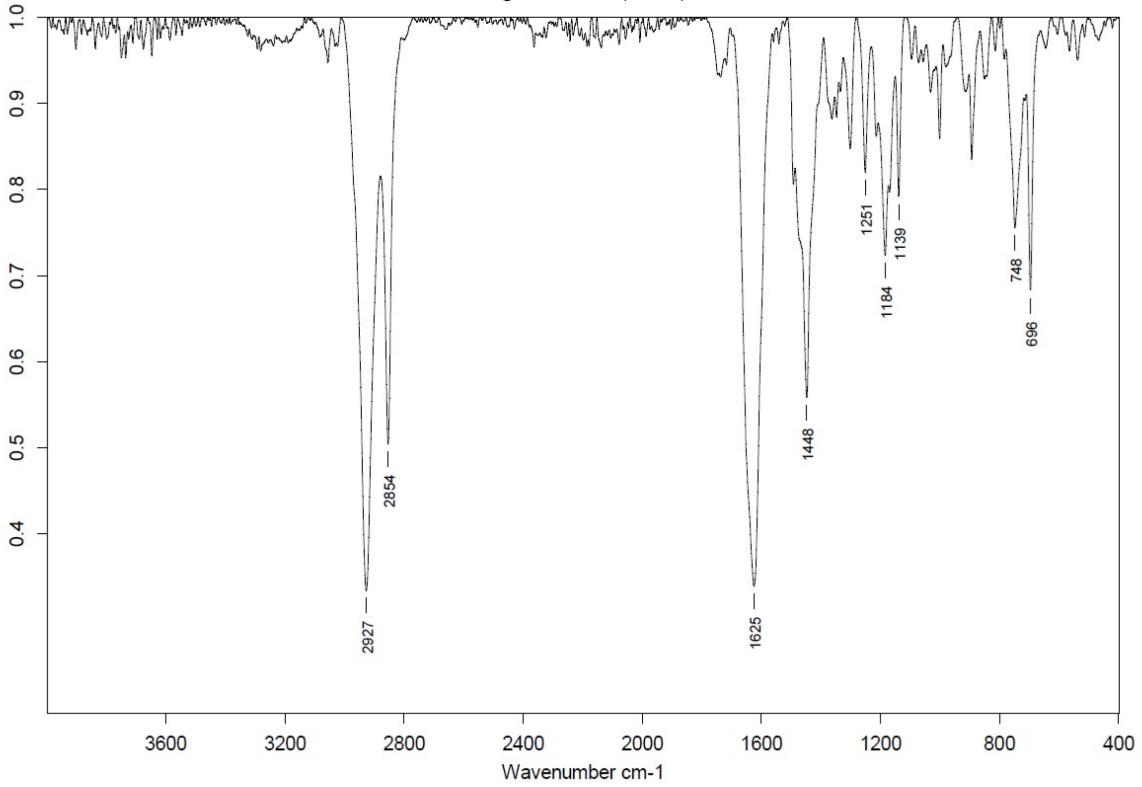

HRMS (ESI$^+$, MeOH): *m/z* calcd. 270.1852 (M + H)$^+$, found: 270.1855.

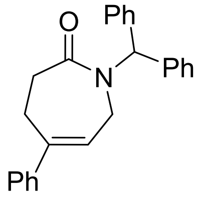

Compound 49

Scale: 0.2 mmol; *E/Z*>99:1, isolated 41.0 mg (58% yield), yellow solid, Hexane : EA = 2 : 1, R_f = 0.15

^{1}H NMR spectrum ($CDCl_3$)

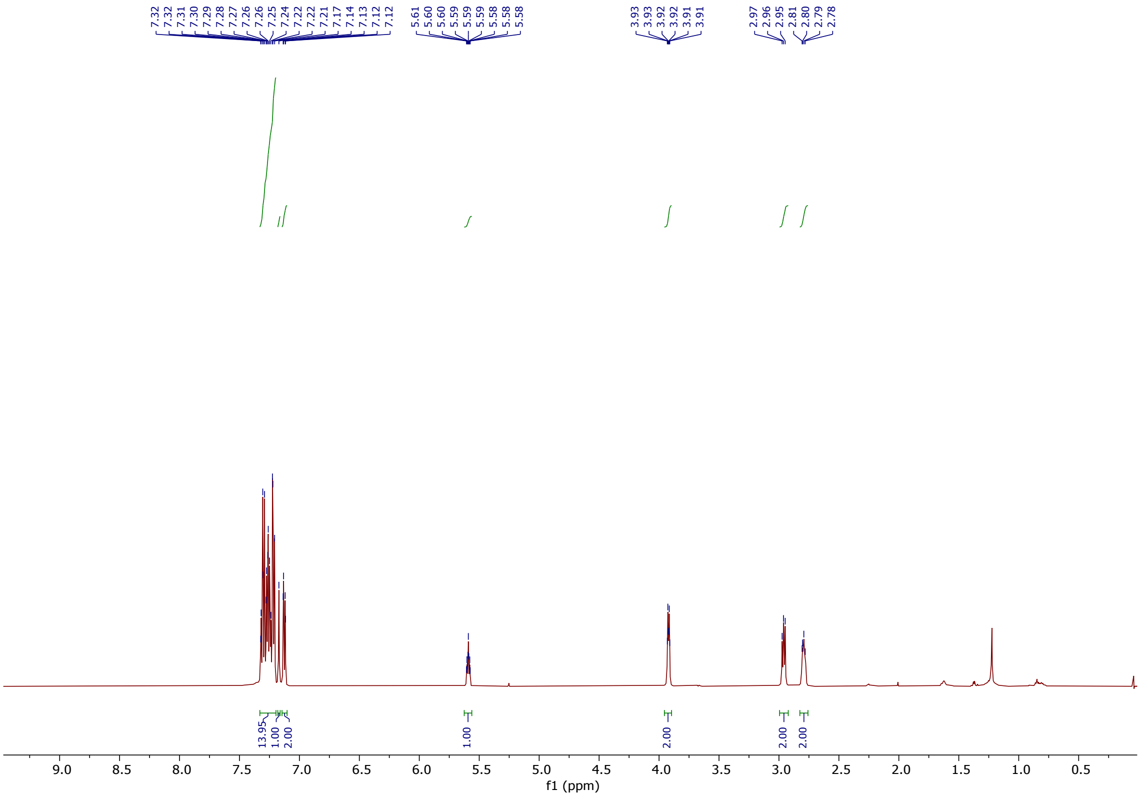

^{1}H NMR (500 MHz, $CDCl_3$) δ 7.33 – 7.20 (m, 13H), 7.17 (s, 1H), 7.14 – 7.10 (m, 2H), 5.59 (tt, J = 6.0, 1.8 Hz, 1H), 3.92 (dt, J = 6.0, 2.2 Hz, 2H), 2.99 – 2.92 (m, 2H), 2.83 – 2.75 (m, 2H).

^{13}C NMR spectrum ($CDCl_3$)

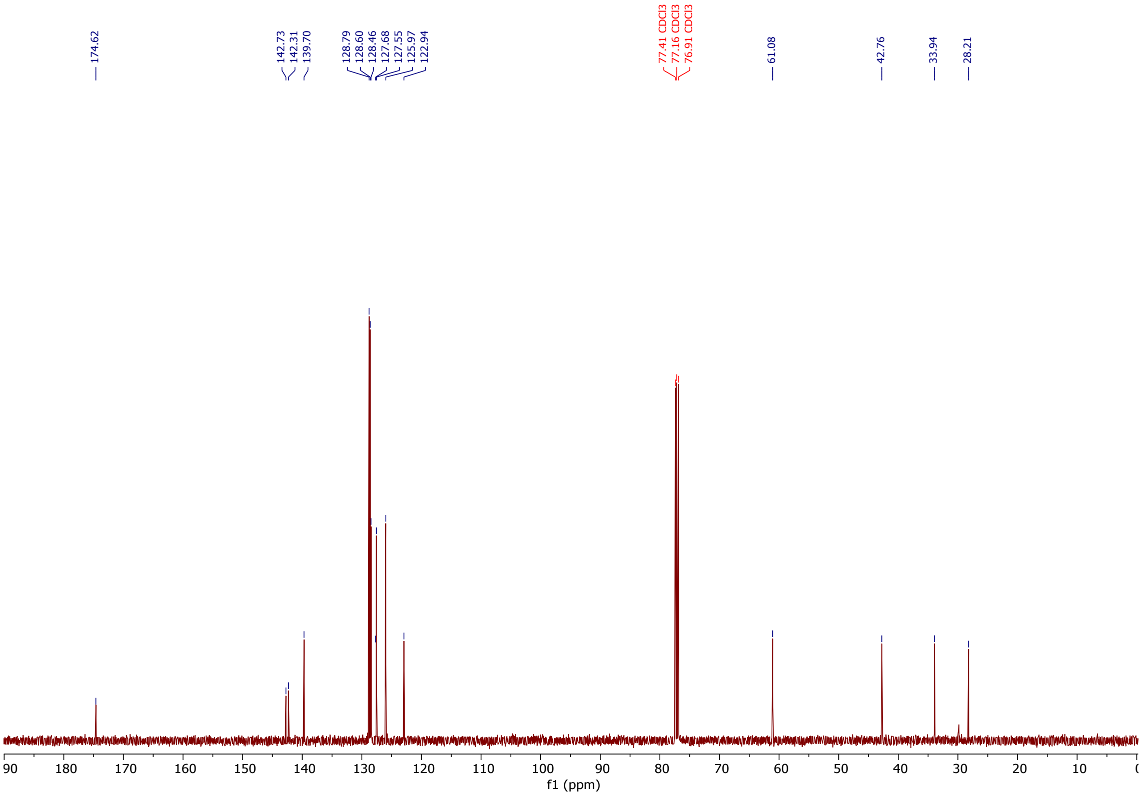

^{13}C NMR (126 MHz, $CDCl_3$) δ 174.62, 142.73, 142.31, 139.70, 128.79, 128.60, 128.46, 127.68, 127.55, 125.97, 122.94, 61.08, 42.76, 33.94, 28.21.

IR spectrum (neat)

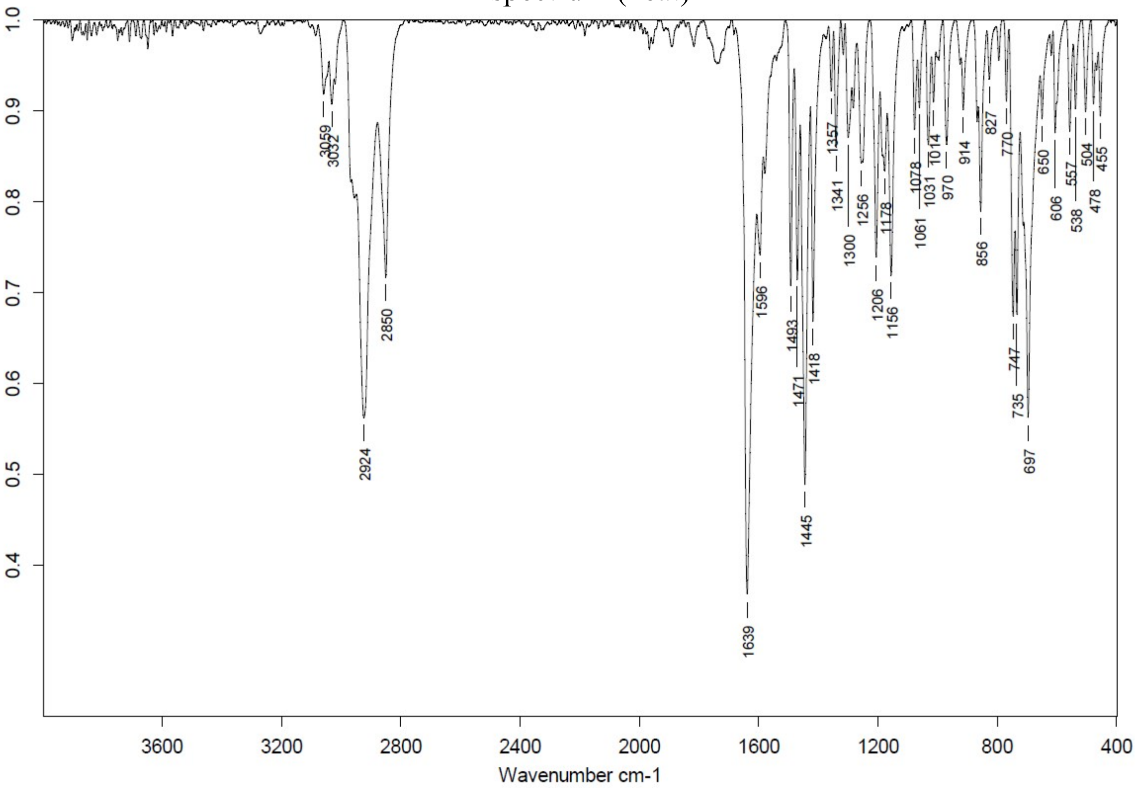

HRMS (ESI$^+$, MeOH): *m/z* calcd. 376.1672 $(M + Na)^+$, found: 376.1669.

S188. Product diversification based on compounds 3 and 29

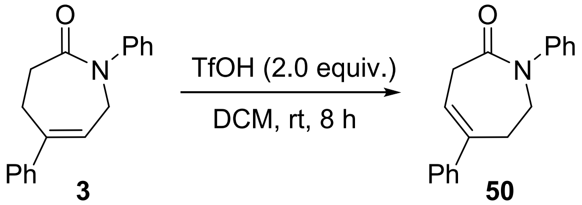

3 50

In a vial equipped with a magnetic stirring bar, caprolactam **3** (52.7 mg, 0.20 mmol, 1.0 equiv.) was dissolved into DCM (0.20 mL), and then trifluoromethanesulfonic acid (0.035 mL, 0.40 mmol, 2.0 equiv.) was added with a syringe. The resultant solution was stirred for 8 h at room temperature. Hereafter, saturated aqueous $NaHCO_3$ was slowly added and the organic components were extracted with DCM (3 × 10 mL). Then, the combined organic layers were dried over Na_2SO_4, filtered, and concentrated under vacuum. The crude product was purified by flash chromatography (Hexane/EtOAc = 2:1) to afford the isomerized product **50** (40.6 mg, 77%) as a light yellow solid.

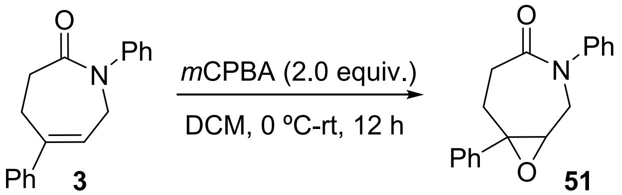

3 51

To a solution of caprolactam **3** (52.7 mg, 0.20 mmol, 1.0 equiv.) in DCM (0.40 mL) was added *m*CPBA (0.40 mmol, 69.0 mg, 2.0 equiv.) at 0 °C under air. Then the reaction mixture was allowed to warm to room temperature, and stirred for 12 h. The reaction mixture was filtered through Celite. The solvent in the filtrate was evaporated under reduced pressure, and the crude product was purified by flash chromatography (Hexane/EtOAc = 1:1) to afford the pure epoxide product **51** (47.5 mg, 85%, >99:1 *dr*) as a yellow solid.

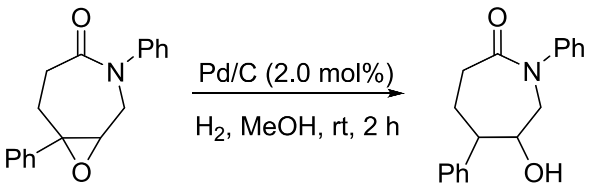

51 52

A Schlenk tube charged with **51** (83.8 mg, 0.30 mmol, 1.0 equiv) and Pd/C catalyst (6.4 mg, 10.0 wt% palladium on carbon) was evacuated and filled with H_2 (balloon) for three times. After that, MeOH (1.0 mL) was added and the reaction mixture was stirred under a H_2 atmosphere (balloon) for 2 h at room temperature and monitored by NMR. The reaction mixture was filtered through Celite. The solvent in the filtrate was evaporated under reduced pressure. The residue was purified by flash chromatography (Hexane/EtOAc = 1:2) to afford the pure product **52** (77.7 mg, 92%, >99:1 *dr*) as a white solid.

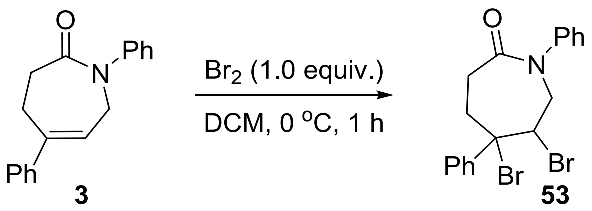

To an oven-dried Schlenk tube was added caprolactam **3** (52.7 mg, 0.20 mmol, 1.0 equiv) and anhydrous DCM (0.40 mL), and the solution was cooled to 0 °C. A solution of Br_2 (10.2 µL, 0.40 mmol 1.0 equiv) in DCM (0.20 mL) was added dropwise under N_2 and the reaction mixture stirred at 0 ºC. After completion (about 1 h, followed by TLC), the reaction mixture was diluted with DCM and washed with 5% $NaHCO_3$ solution. The organic layer was then dried over $MgSO_4$, filtered, and then concentrated. The residue was purified by flash chromatography (Hexane/EtOAc = 5:1) to afford the dibrominated product **53** (70.2 mg, 83%, >99:1 *dr*) as a colorless solid. The X-ray molecular structure was also determined.

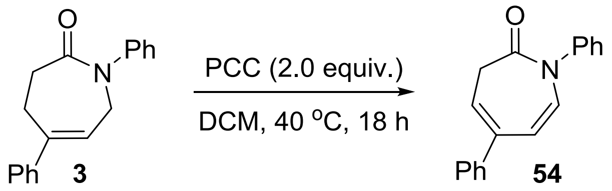

Under N_2, to a magnetically stirred solution of caprolactam **3** (52.7 mg, 0.20 mmol, 1.0 equiv) in DCM (1.0 mL) was added pyridinium chlorochromate (PCC, 86.2 mg, 0.40 mmol, 2.0 equiv.). The mixture was stirred at 40 °C for 18 h, then cooled, diluted with water (10 mL), and extracted with EtOAc (3 × 10 mL). The combined organic layers were washed with 5% aqueous $NaHCO_3$ (10 mL), and dried with sodium sulfate, filtered, and then concentrated. Purification of the residue by flash chromatography (Hexane/EtOAc = 10:1) to afford the desired product **54** (29.3 mg, 56%) as a colorless oil.

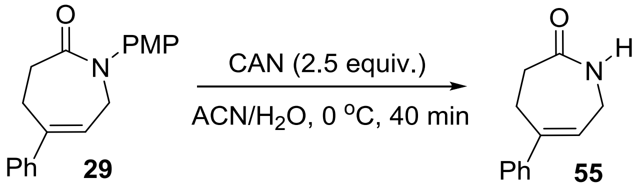

In a vial equipped with a magnetic stirring bar, caprolactam **29** (44.0 mg, 0.15 mmol, 1.0 equiv.) was dissolved into ACN (0.9 mL) and stirred at 0 °C. A solution of CAN (205.6 mg, 0.375 mmol, 2.5 equiv.) in H_2O (1.3 mL) was added dropwise. The resultant solution was stirred at 0 °C until no starting materials could be detected by TLC (about 40 min). Hereafter, saturated aqueous $NaHCO_3$ was added and the organic components were extracted with DCM (3 × 10 mL). The combined organic layers were then dried over $MgSO_4$, filtered, and then concentrated, the crude product was purified by flash chromatography (DCM/MeOH = 30:1) to afford the deprotected lactam **55** (11.2 mg, 40%) as a light yellow solid.

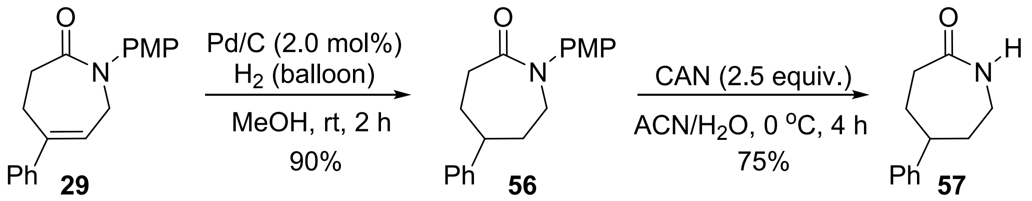

A Schlenk tube was charged with **29** (176.0 mg, 0.6 mmol, 1.0 equiv) and Pd/C as catalyst (12.8 mg, 10.0 wt % palladium on carbon), and then the reactor was evacuated/filled with H_2 (balloon) for three times. After that, MeOH (2.0 mL) was added and the reaction mixture was stirred under H_2 atmosphere (balloon) for 2 h at room temperature, and monitored by NMR. The reaction mixture was filtered through Celite. The solvent in the filtrate was evaporated under reduced pressure. The residue was purified by flash chromatography (Hex/EtOAc = 3:1) to afford the pure saturated lactam **56** (159.5 mg, 90%) as a yellow solid.

In a vial equipped with a magnetic stirring bar, caprolactam **56** (44.3 mg, 0.15 mmol, 1.0 equiv.) was dissolved into ACN (0.9 mL) and stirred at 0 °C. A solution of CAN (205.6 mg, 0.375 mmol, 2.5 equiv.) in H_2O (1.3 mL) was added dropwise. The resultant solution was stirred at 0 °C for 4 h. Hereafter, saturated aqueous $NaHCO_3$ was added and the organic components were extracted with DCM (3 × 10 mL). The combined organic layers were then dried over $MgSO_4$, filtered, and then concentrated, the crude product was purified by flash chromatography (DCM/MeOH = 30:1) to afford the deprotected lactam **57** (21.3 mg, 75%) as a light brown solid.

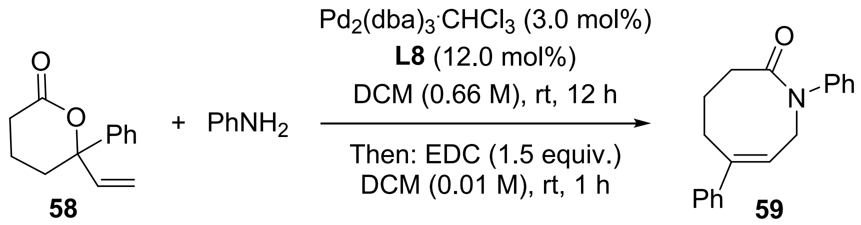

Six-membered vinyl lactone **58** (40.5 mg, 0.20 mmol, 1.0 equiv) was combined with $Pd_2(dba)_3$·$CHCl_3$ (6.0 mg, 3.0 mol%), **L8** (9.6 mg, 12.0 mol%) and aniline (38.0 mg, 0.40 mmol) in DCM (0.30 mL) at room temperature in air. The reaction mixture was stirred at room temperature for 12 h, then an aliquot of the mixture was taken for NMR analysis, which showed the unsaturated amino acid intermediate to have an *E*/*Z* ratio of >99:1. Hereafter, the mixture was diluted to 0.01 M and EDC (57.5 mg, 3.0 mmol) was added and the reaction mixture stirred for another 1 h. The desired eight-membered lactam **59** (30.0 mg, 54%) was isolated by flash chromatography (Hexane/EtOAc = 2:1).

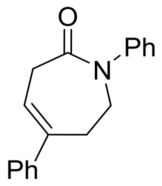

Compound 50

Scale: 0.2 mmol; isolated 40.6 mg (77% yield), light yellow solid, Hexane : EA = 2 : 1, R_f = 0.25

^{1}H NMR spectrum ($CDCl_3$)

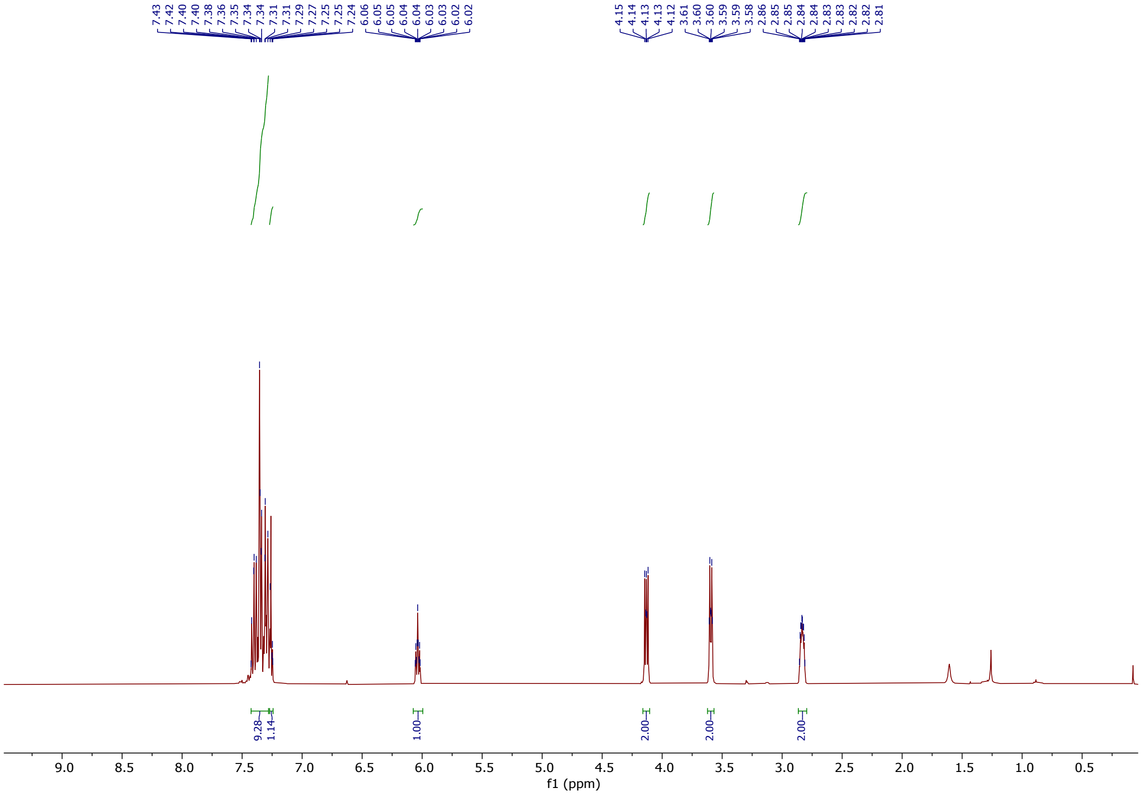

^{1}H NMR (400 MHz, $CDCl_3$) δ 7.43 – 7.28 (m, 9H), 7.27 – 7.24 (m, 1H), 6.04 (tt, J = 6.2, 1.8 Hz, 1H), 4.16 – 4.10 (m, 2H), 3.59 (dt, J = 6.2, 2.3 Hz, 2H), 2.83 (ddq, J = 8.1, 4.2, 2.2 Hz, 2H).

^{13}C NMR spectrum ($CDCl_3$)

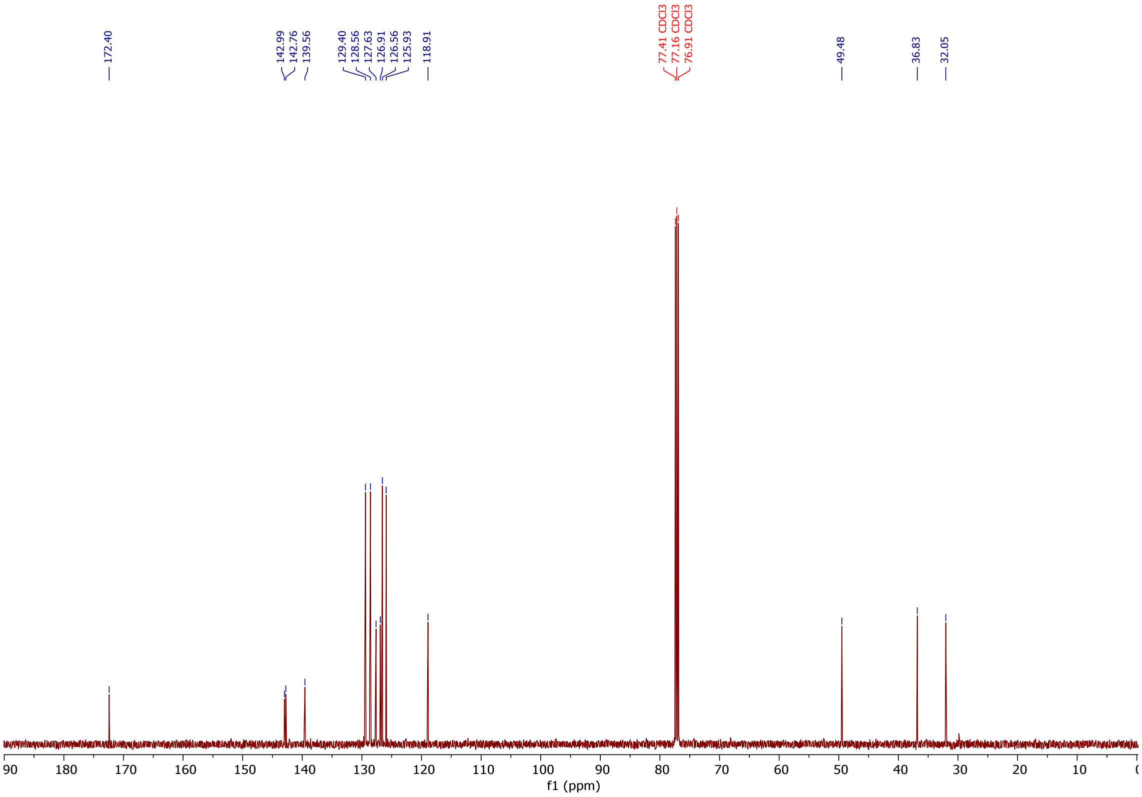

^{13}C NMR (126 MHz, $CDCl_3$) δ 172.40, 142.99, 142.76, 139.56, 129.40, 128.56, 127.63, 126.91, 126.56, 125.93, 118.91, 49.48, 36.83, 32.05.

IR spectrum (neat)

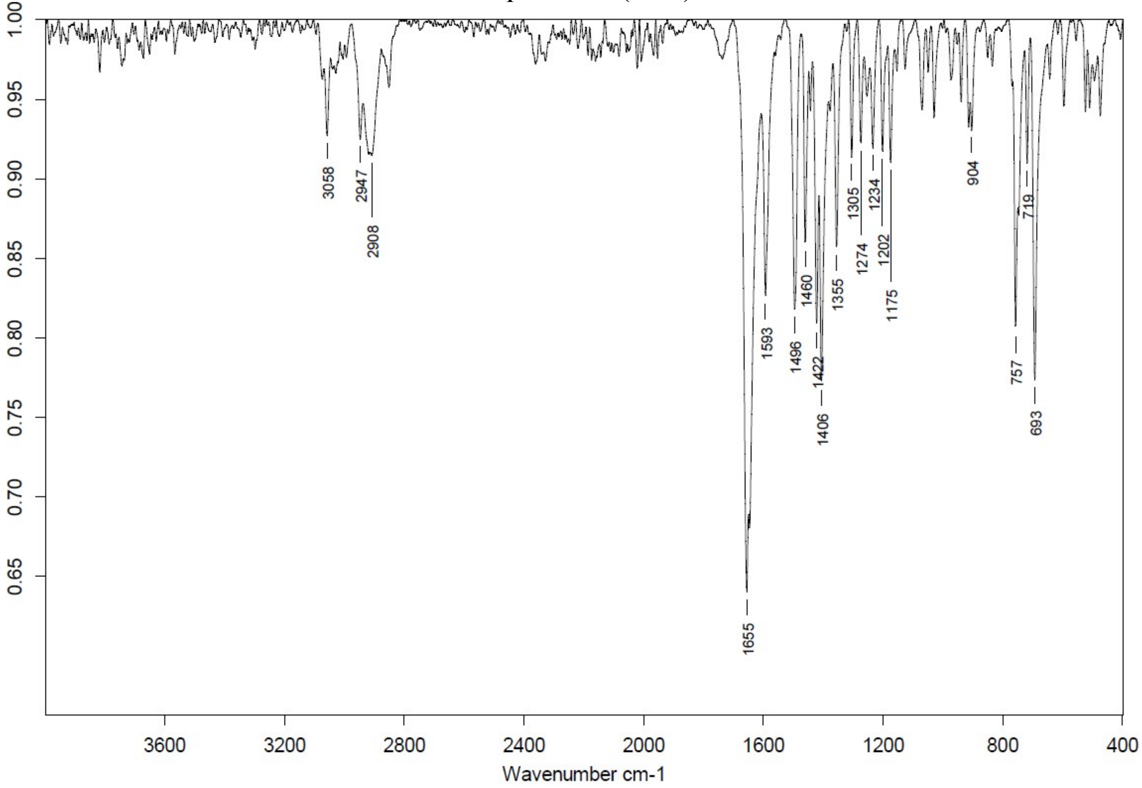

HRMS (ESI$^+$, MeOH): *m/z* calcd. 264.1383 $(M + H)^+$, found: 264.1386.

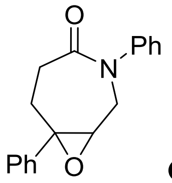

Compound 51

Scale: 0.2 mmol; isolated 47.5 mg (85% yield, >99:1 *dr*), yellow solid, Hexane : EA = 1 : 1, R_f = 0.15

^{1}H NMR spectrum ($CDCl_3$)

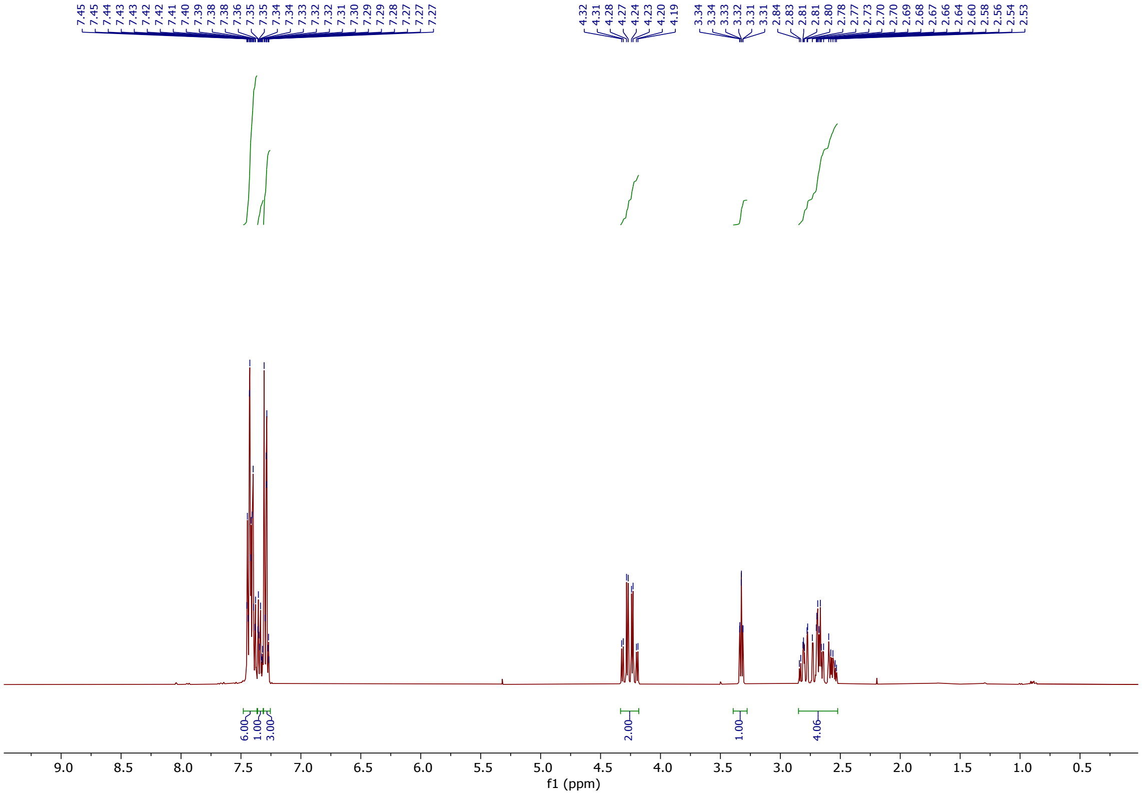

^{1}H NMR (400 MHz, $CDCl_3$) δ 7.48 – 7.37 (m, 6H), 7.36 – 7.31 (m, 1H), 7.31 – 7.25 (m, 3H), 4.26 (qd, J = 16.4, 5.3 Hz, 2H), 3.33 (td, J = 5.3, 0.8 Hz, 1H), 2.85 – 2.52 (m, 4H).

^{13}C NMR spectrum (CDCl$_3$)

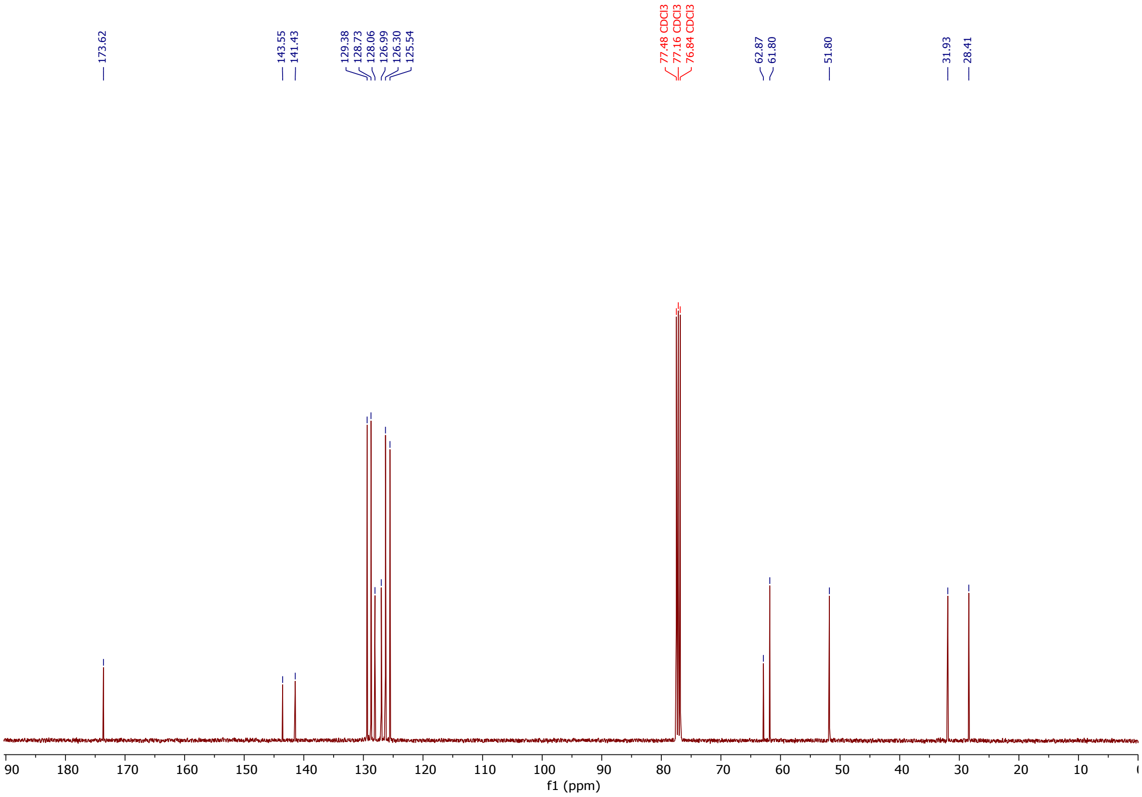

^{13}C NMR (101 MHz, CDCl$_3$) δ 173.62, 143.55, 141.43, 129.38, 128.73, 128.06, 126.99, 126.30, 125.54, 62.87, 61.80, 51.80, 31.93, 28.41.

IR spectrum (neat)

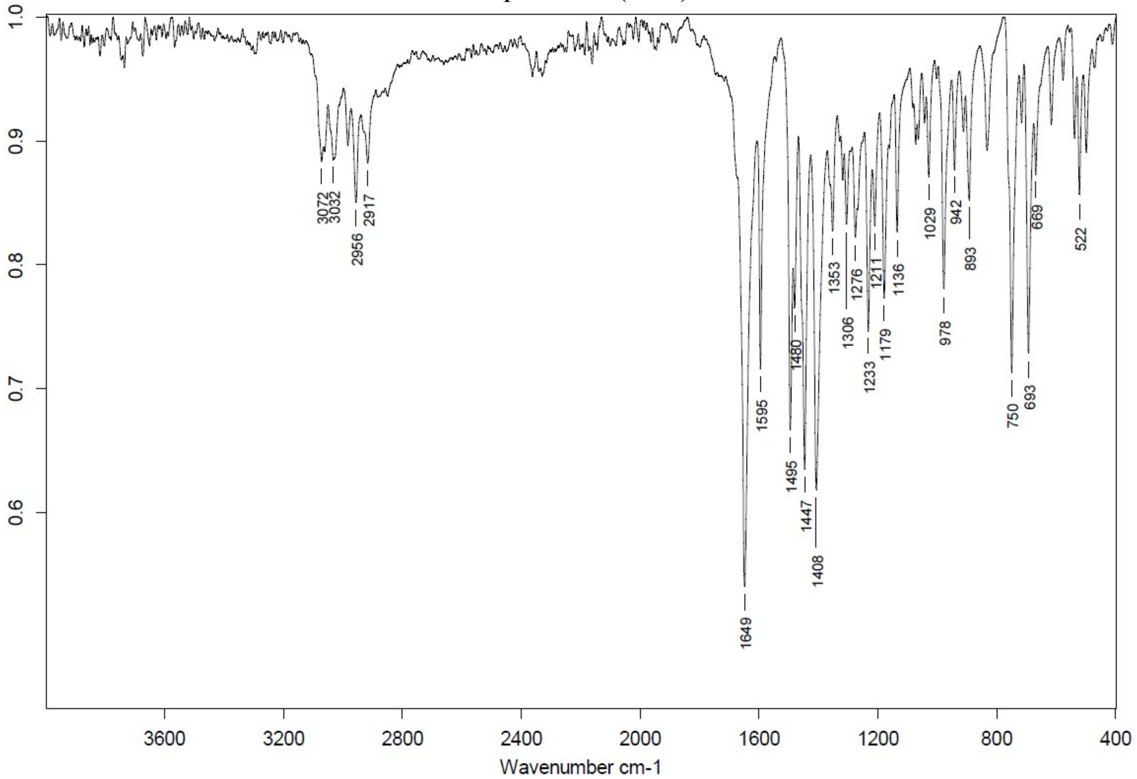

HRMS (ESI$^+$, MeOH): *m/z* calcd. 280.1332 $(M + H)^+$, found: 280.1328.

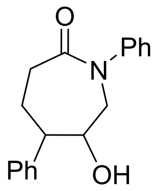

Compound 52

Scale: 0.3 mmol; isolated 77.7 mg (92% yield, >99:1 *dr*), white solid, Hexane : EA = 1 : 2, $R_f = 0.15$

^{1}H NMR spectrum ($CDCl_3$)

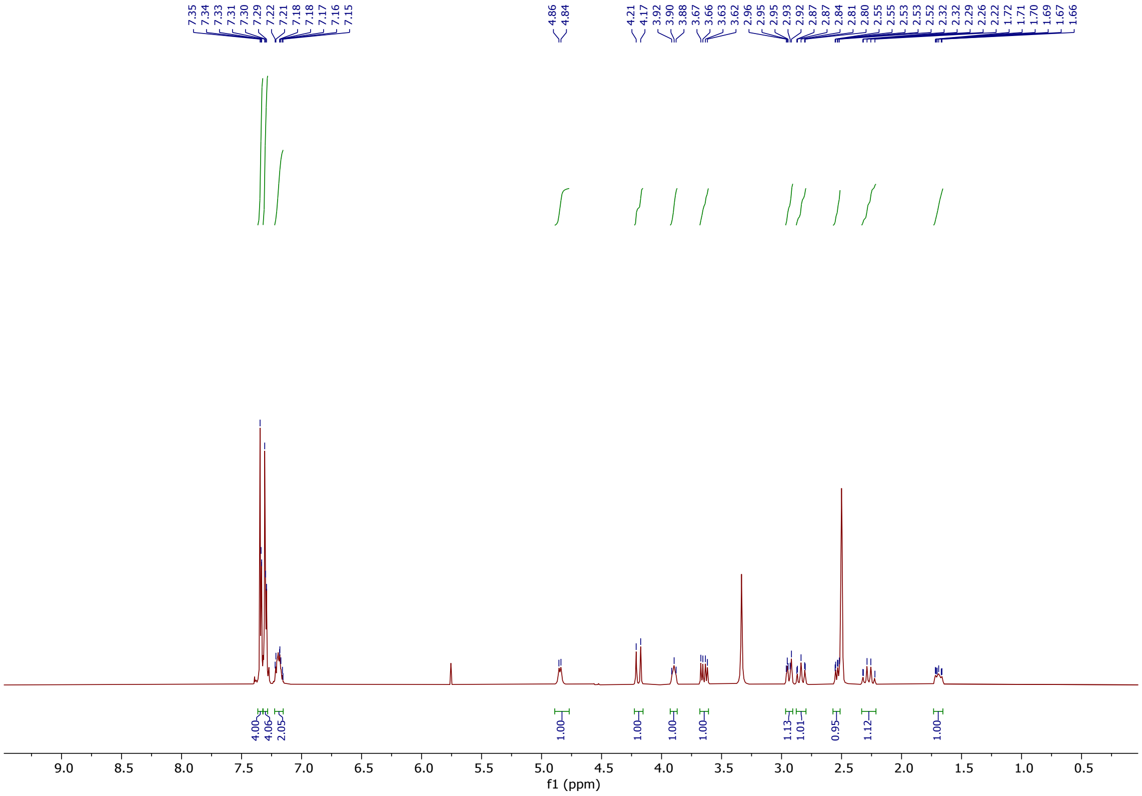

^{1}H NMR (400 MHz, DMSO) δ 7.37 – 7.32 (m, 4H), 7.32 – 7.26 (m, 4H), 7.22 – 7.15 (m, 2H), 4.85 (d, J = 6.8 Hz, 1H), 4.19 (d, J = 15.1 Hz, 1H), 3.90 (t, J = 7.3 Hz, 1H), 3.65 (dd, J = 15.2, 6.7 Hz, 1H), 2.97 – 2.91 (m, 1H), 2.88 – 2.80 (m, 1H), 2.57 – 2.52 (m, 1H), 2.33 – 2.21 (m, 1H), 1.73 – 1.66 (m, 1H).

^{13}C NMR spectrum ($CDCl_3$)

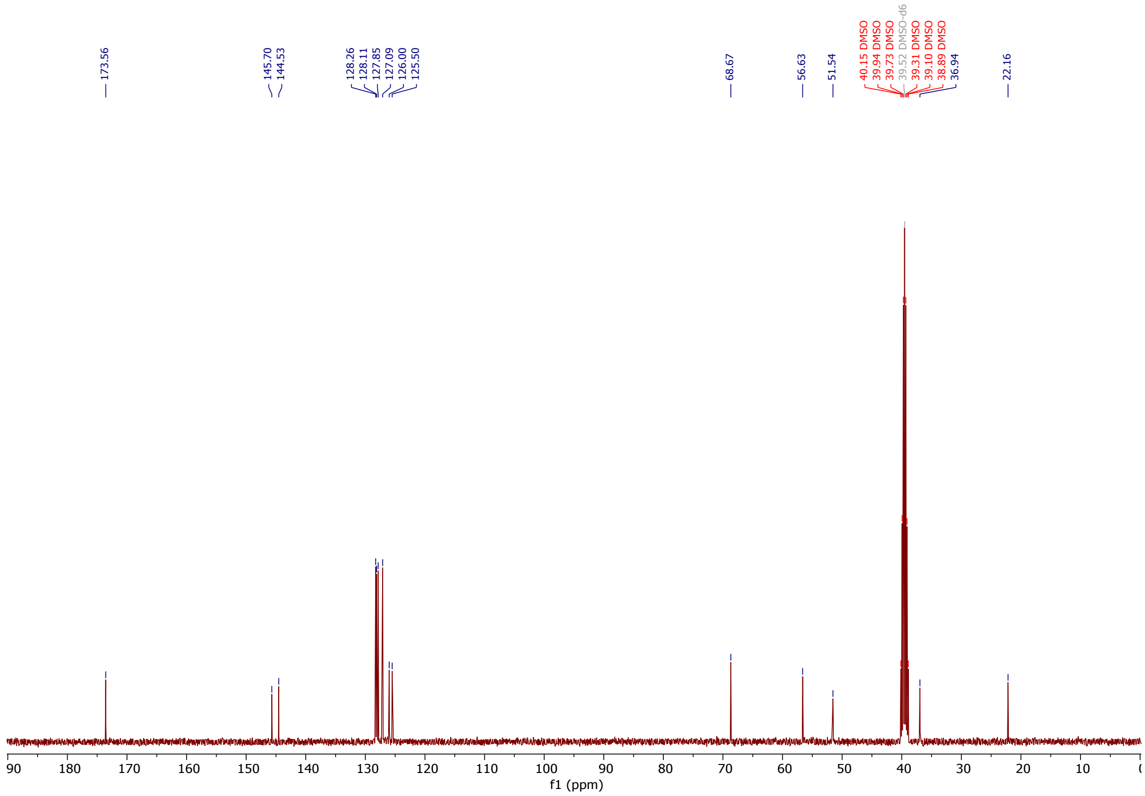

^{13}C NMR (101 MHz, DMSO) δ 173.56, 145.70, 144.53, 128.26, 128.11, 127.85, 127.09, 126.00, 125.50, 68.67, 56.63, 51.54, 36.94, 22.16.

IR spectrum (neat)

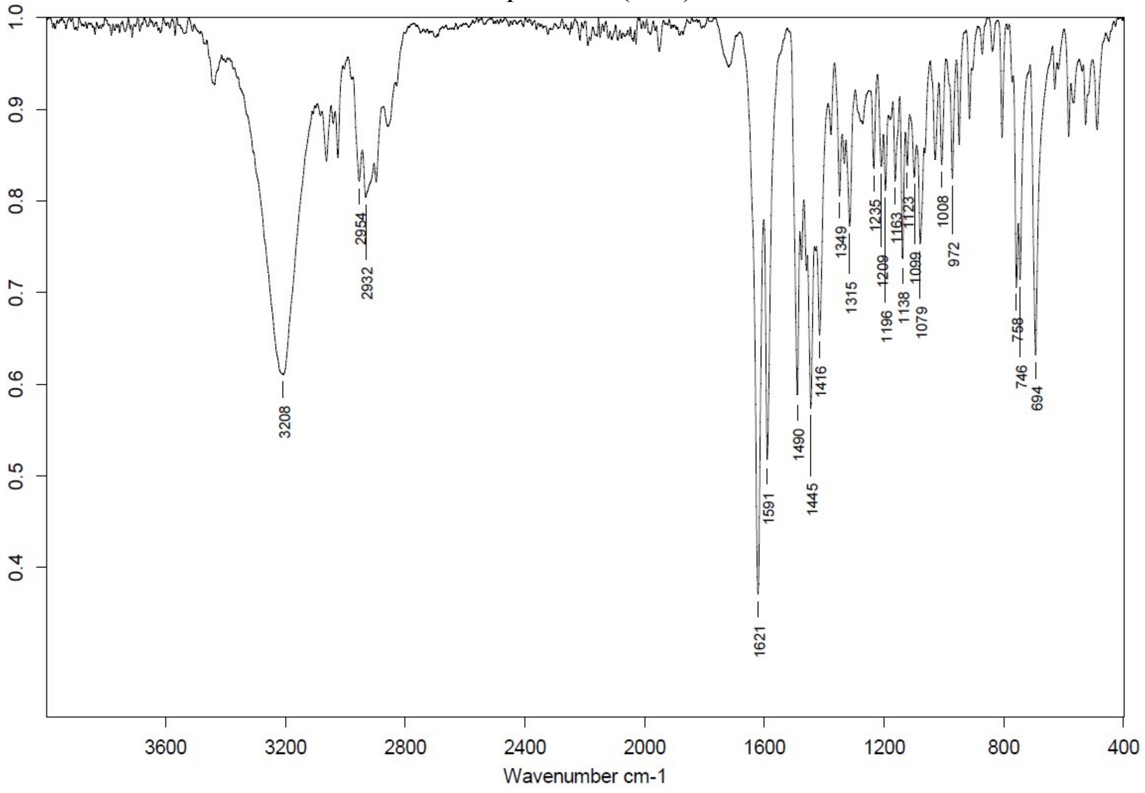

HRMS (ESI$^+$, MeOH): *m/z* calcd. 282.1489 $(M + H)^+$, found: 282.1495.

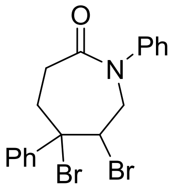

Compound 53

Scale: 0.2 mmol; isolated 70.2 mg (83% yield, >99:1 *dr*), colorless solid, Hexane : EA = 5 : 1, $R_f = 0.10$

^{1}H NMR spectrum ($CDCl_3$)

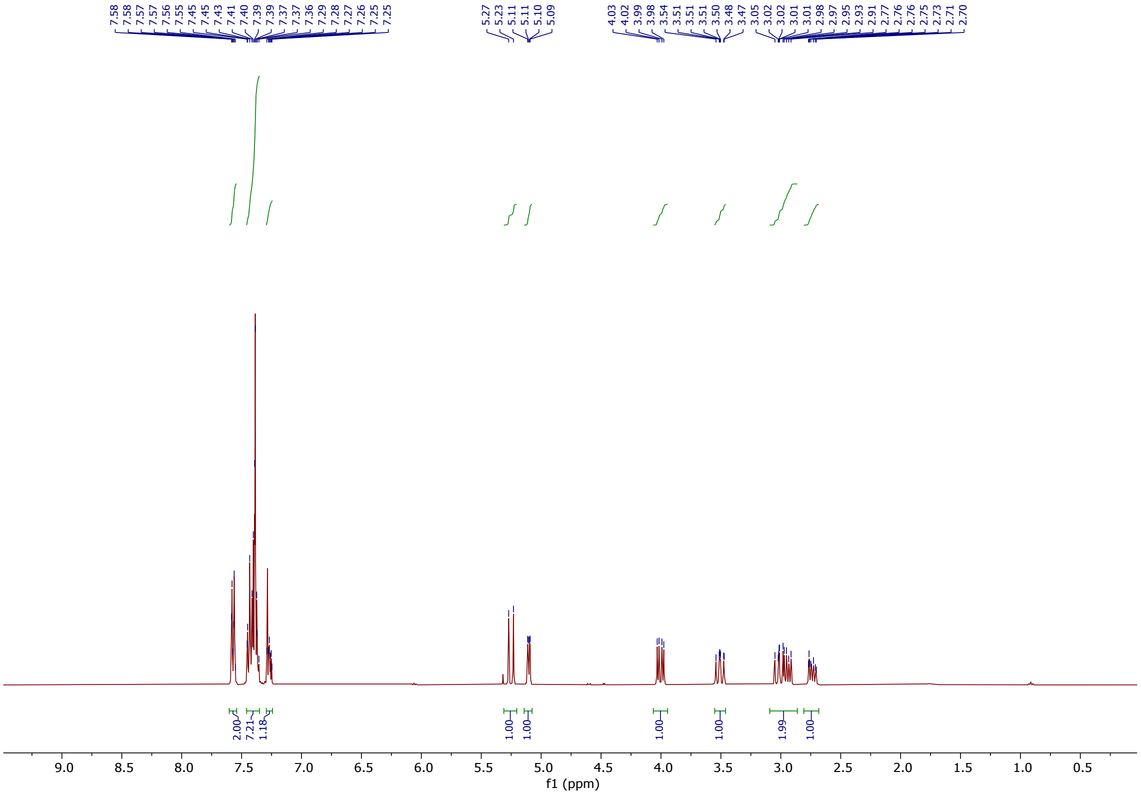

^{1}H NMR (400 MHz, $CDCl_3$) δ 7.60 – 7.54 (m, 2H), 7.46 – 7.35 (m, 7H), 7.29 – 7.24 (m, 1H), 5.25 (d, J = 15.9 Hz, 1H), 5.10 (dd, J = 5.8, 2.1 Hz, 1H), 4.00 (dd, J = 15.9, 6.1 Hz, 1H), 3.55 – 3.46 (m, 1H), 3.09 – 2.86 (m, 2H), 2.81 – 2.68 (m, 1H).

^{13}C NMR spectrum ($CDCl_3$)

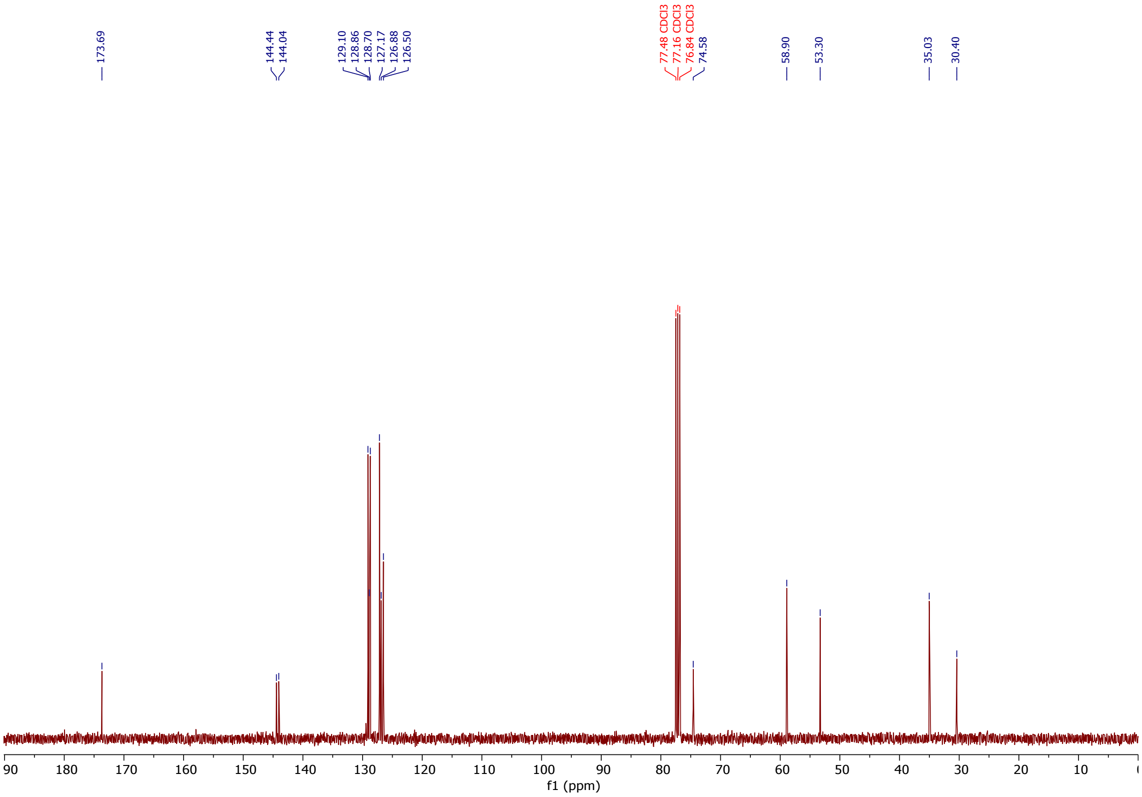

^{13}C NMR (101 MHz, $CDCl_3$) δ 173.69, 144.44, 144.04, 129.10, 128.86, 128.70, 127.17, 126.88, 126.50, 74.58, 58.90, 53.30, 35.03, 30.40.

IR spectrum (neat)

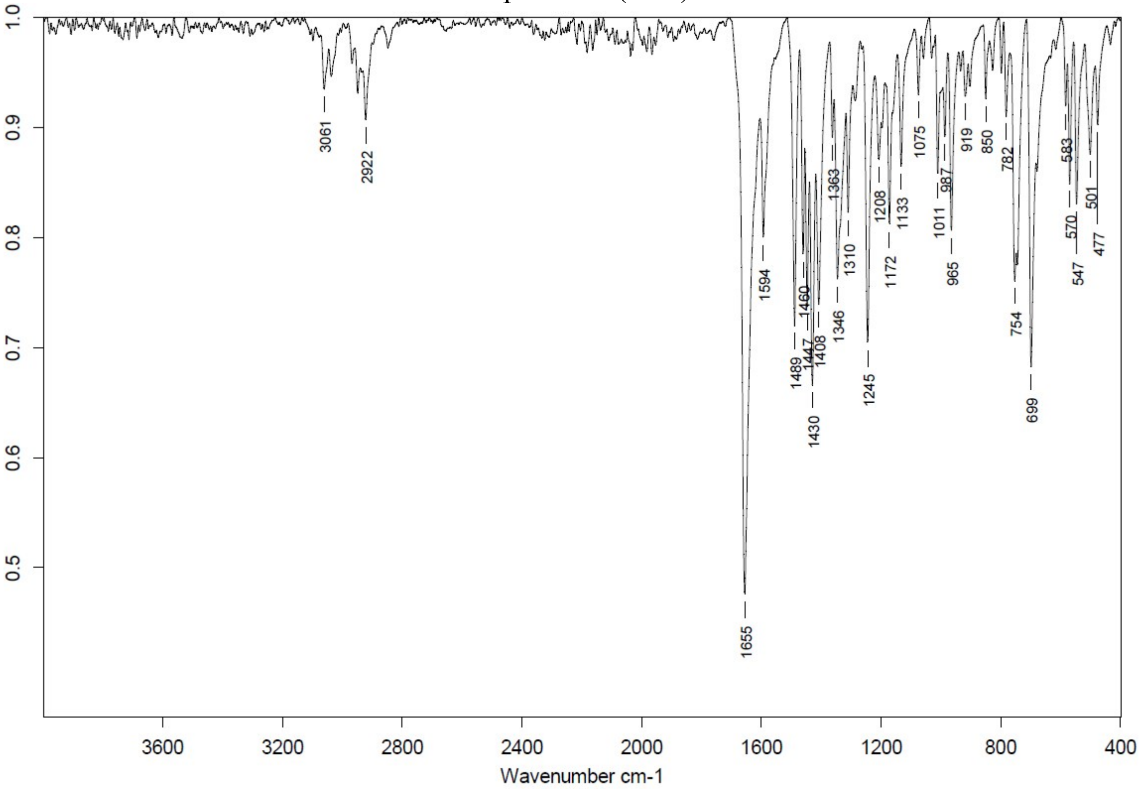

HRMS (ESI^+, MeOH): *m/z* calcd. 421.9750 $(M + H)^+$, found: 421.9755.

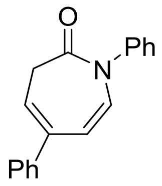

Compound 54

Scale: 0.2 mmol; isolated 29.3 mg (56% yield), colorless oil, Hexane : EA = 10 : 1, R_f = 0.15

^{1}H NMR spectrum ($CDCl_3$)

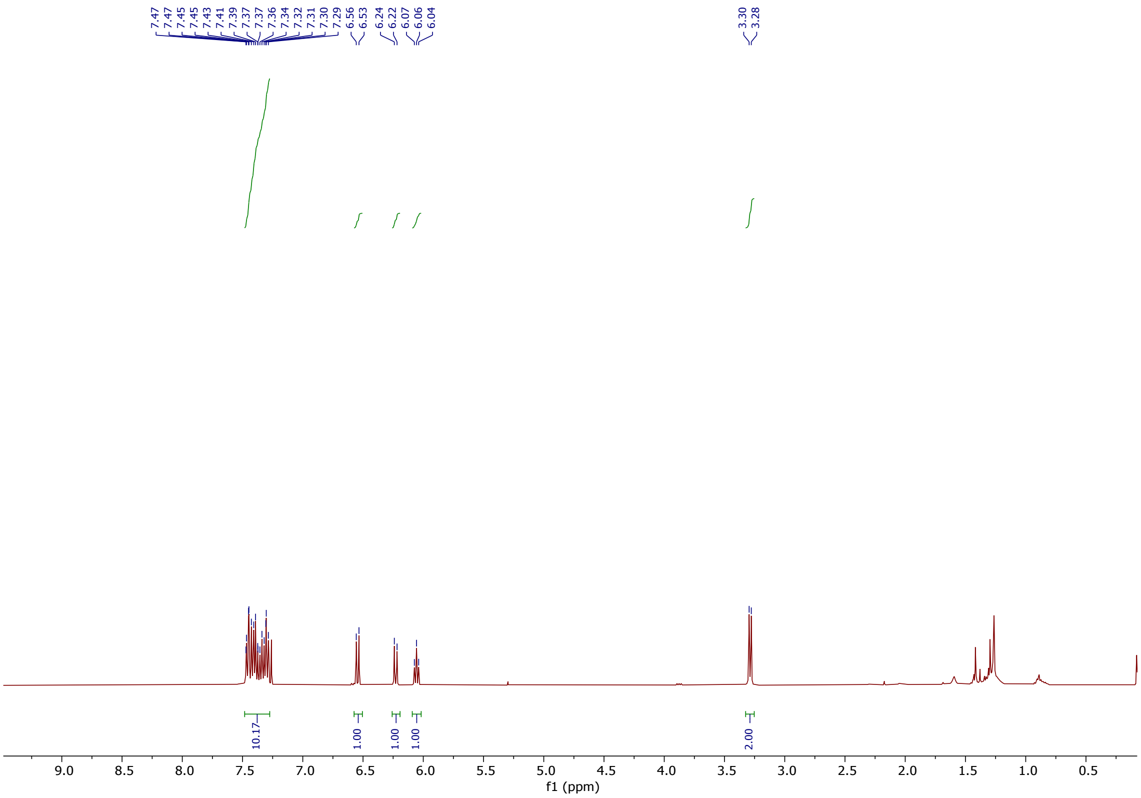

^{1}H NMR (400 MHz, $CDCl_3$) δ 7.48 – 7.27 (m, 10H), 6.54 (d, J = 9.0 Hz, 1H), 6.23 (d, J = 9.1 Hz, 1H), 6.06 (t, J = 7.2 Hz, 1H), 3.29 (d, J = 7.2 Hz, 2H).

^{13}C NMR spectrum ($CDCl_3$)

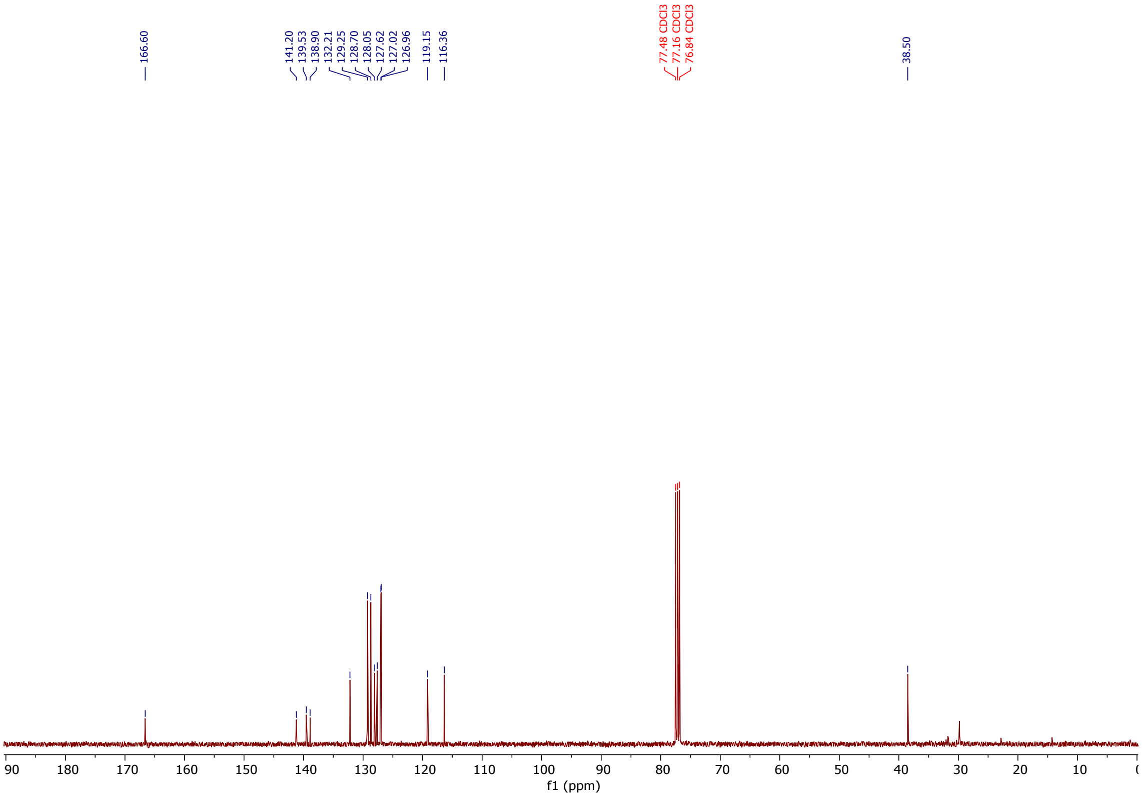

^{13}C NMR (101 MHz, $CDCl_3$) δ 166.60, 141.20, 139.53, 138.90, 132.21, 129.25, 128.70, 128.05, 127.62, 127.02, 126.96, 119.15, 116.36, 38.50.

IR spectrum (neat)

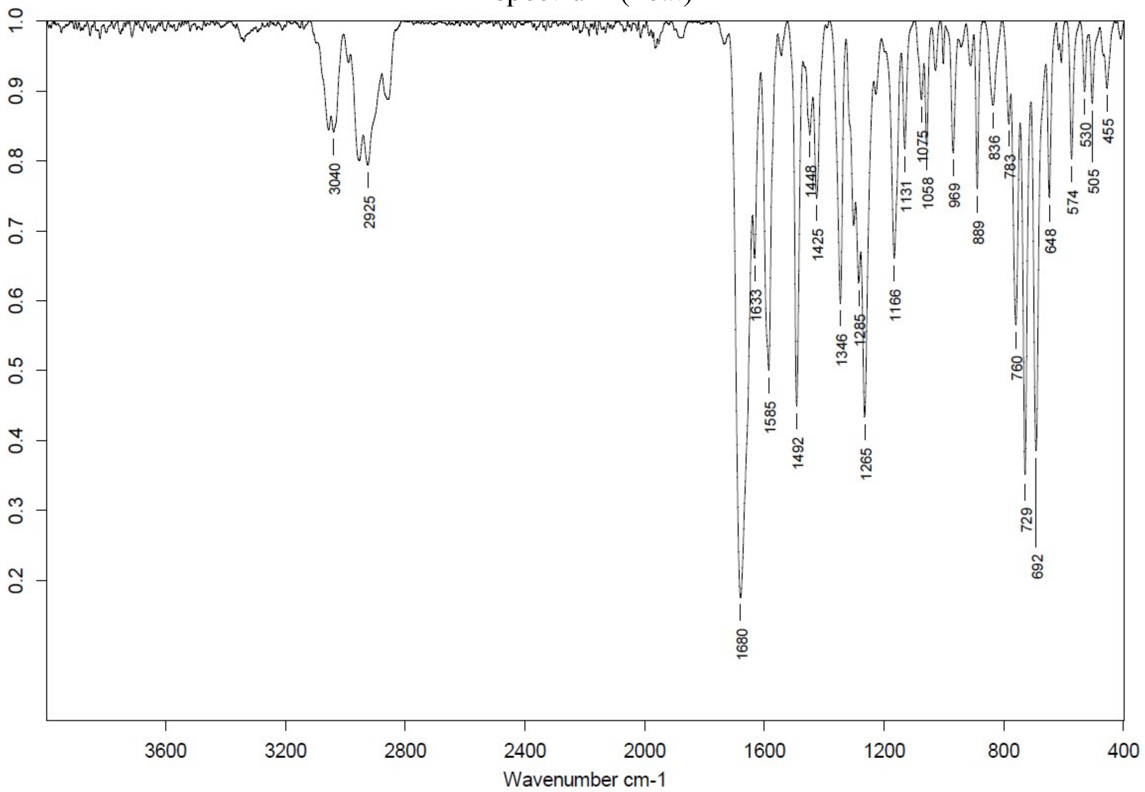

HRMS (ESI$^+$, MeOH): *m/z* calcd. 262.1226 $(M + H)^+$, found: 262.1225.

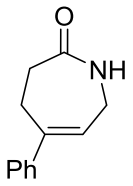

Compound 55

Scale: 0.15 mmol; isolated 11.2 mg (40% yield), light yellow solid, DCM : MeOH = 30 : 1, $R_f = 0.15$

^{1}H NMR spectrum ($CDCl_3$)

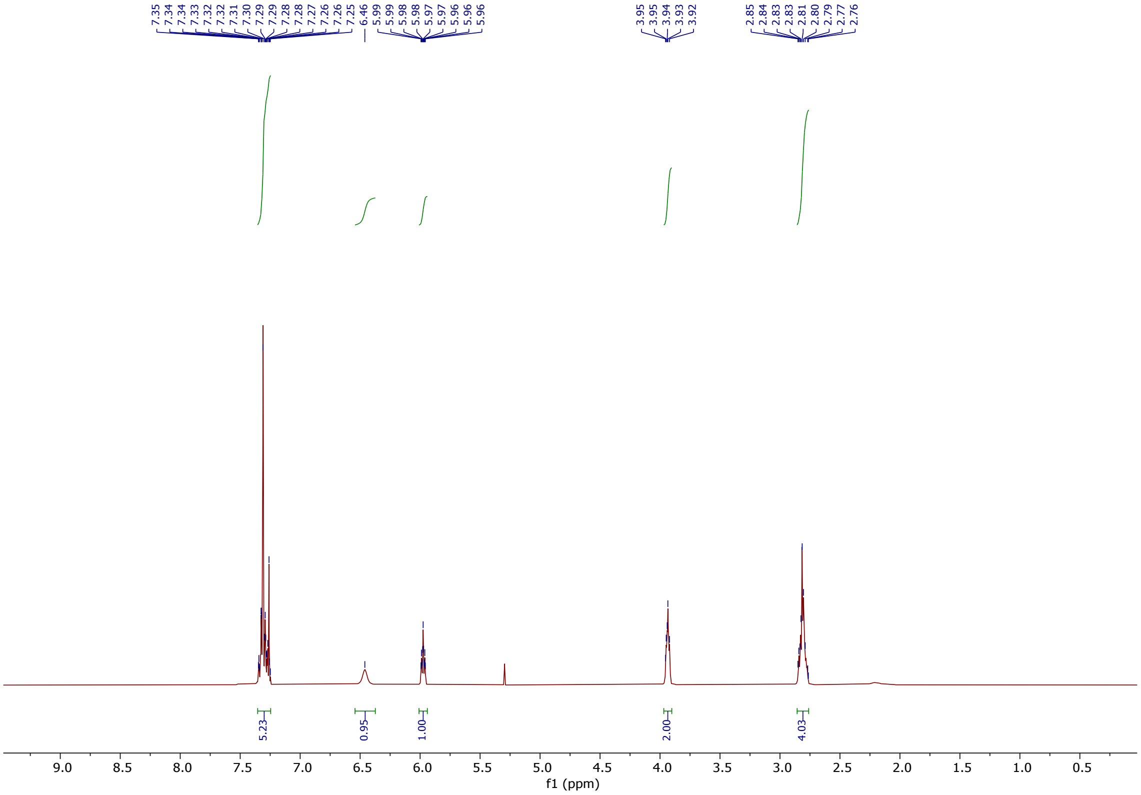

^{1}H NMR (400 MHz, $CDCl_3$) δ 7.35 – 7.25 (m, 5H), 6.46 (s, 1H), 5.97 (tt, J = 5.6, 1.6 Hz, 1H), 3.96 – 3.90 (q, J = 5.5, 3.9 Hz, 2H), 2.86 – 2.76 (m, 4H).

^{13}C NMR spectrum ($CDCl_3$)

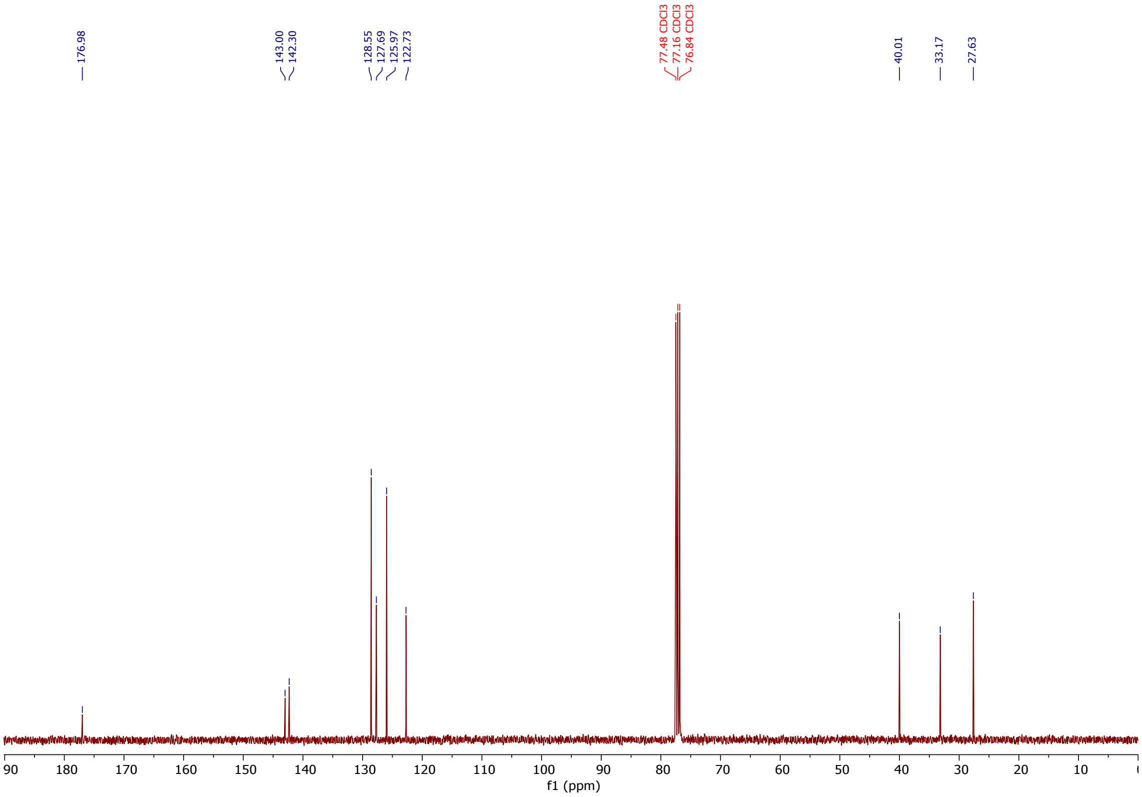

^{13}C NMR (101 MHz, $CDCl_3$) δ 176.98, 143.00, 142.30, 128.55, 127.69, 125.97, 122.73, 40.01, 33.17, 27.63.

IR spectrum (neat)

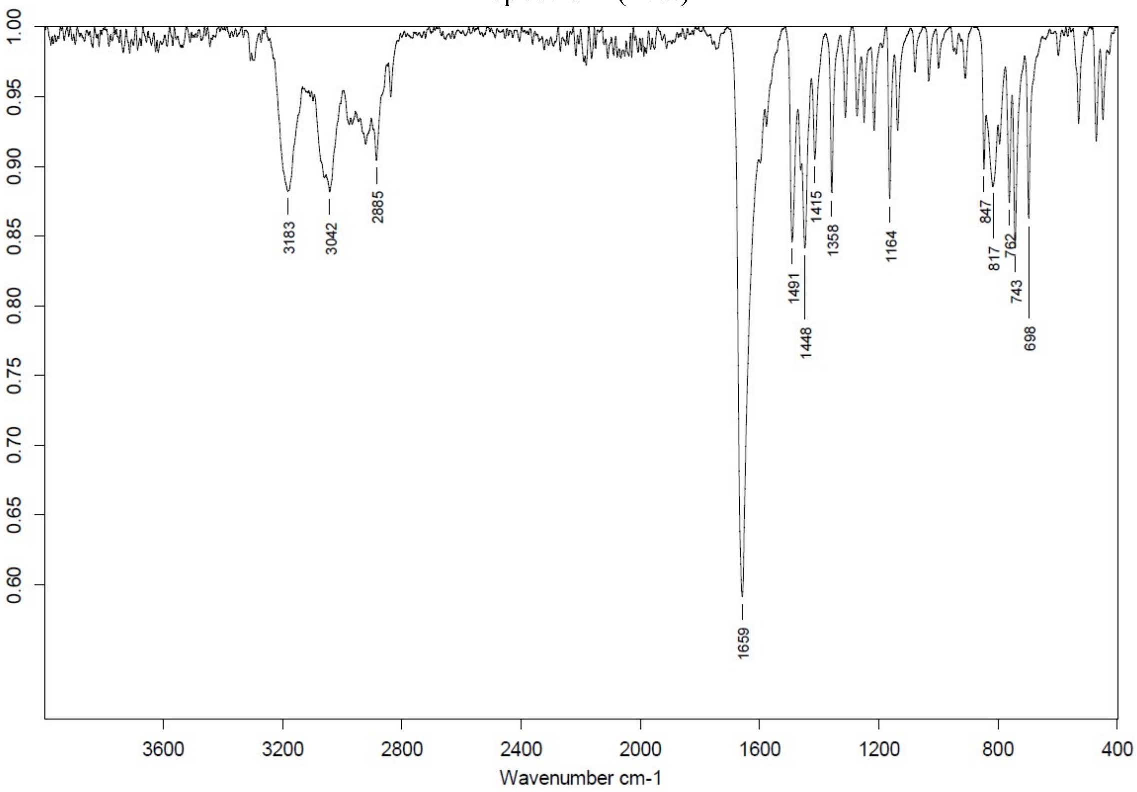

HRMS (ESI$^+$, MeOH): *m/z* calcd. 210.0889 $(M + Na)^+$, found: 210.0884.

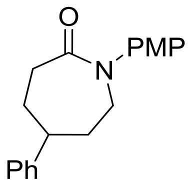

Compound 56 (from 29)

Scale: 0.6 mmol; isolated 159.5 mg (90% yield), yellow solid, Hexane : EA = 3 : 1, R_f = 0.18

^{1}H NMR spectrum ($CDCl_3$)

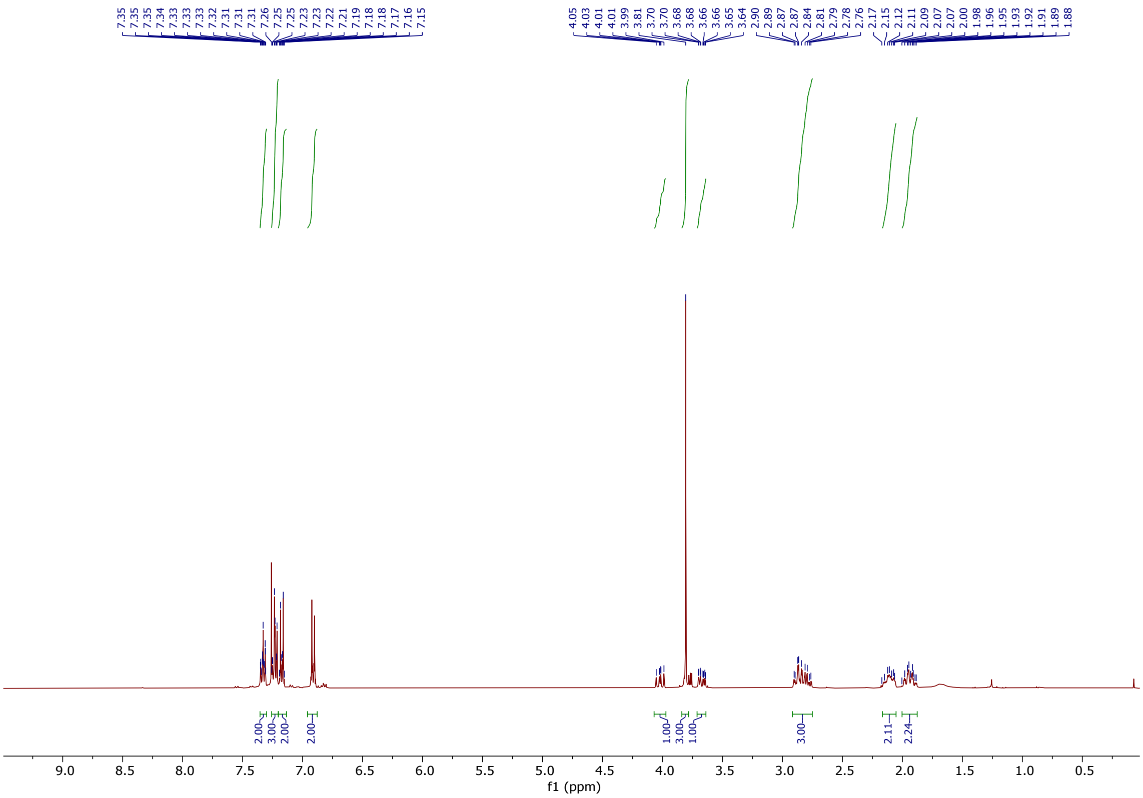

^{1}H NMR (400 MHz, $CDCl_3$) δ 7.36 – 7.30 (m, 2H), 7.26 – 7.20 (m, 3H), 7.20 – 7.13 (m, 2H), 6.96 – 6.88 (m, 2H), 4.02 (dd, J = 15.2, 10.7 Hz, 1H), 3.81 (s, 3H), 3.67 (ddd, J = 15.4, 6.4, 1.7 Hz, 1H), 2.92 – 2.75 (m, 3H), 2.17 – 2.05 (m, 2H), 2.00 – 1.88 (m, 2H).

^{13}C NMR spectrum ($CDCl_3$)

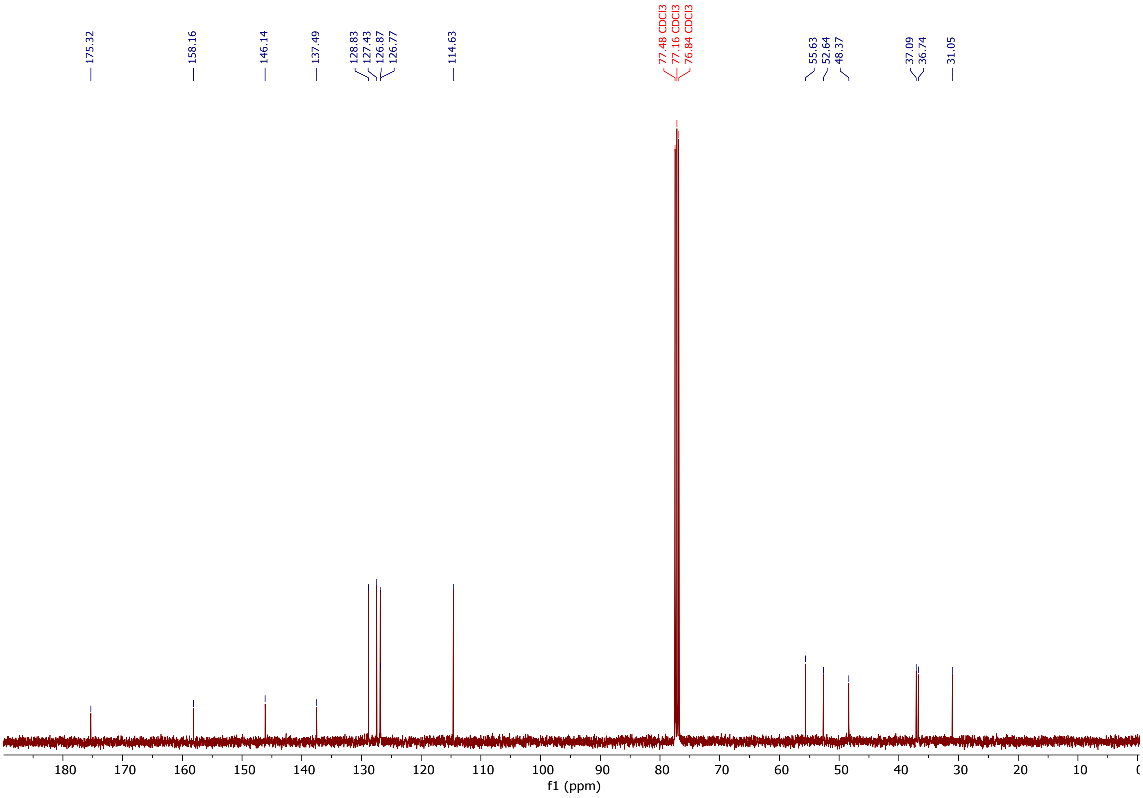

^{13}C NMR (101 MHz, $CDCl_3$) δ 175.32, 158.16, 146.14, 137.49, 128.83, 127.43, 126.87, 126.77, 114.63, 55.63, 52.64, 48.37, 37.09, 36.74, 31.05.

IR spectrum (neat)

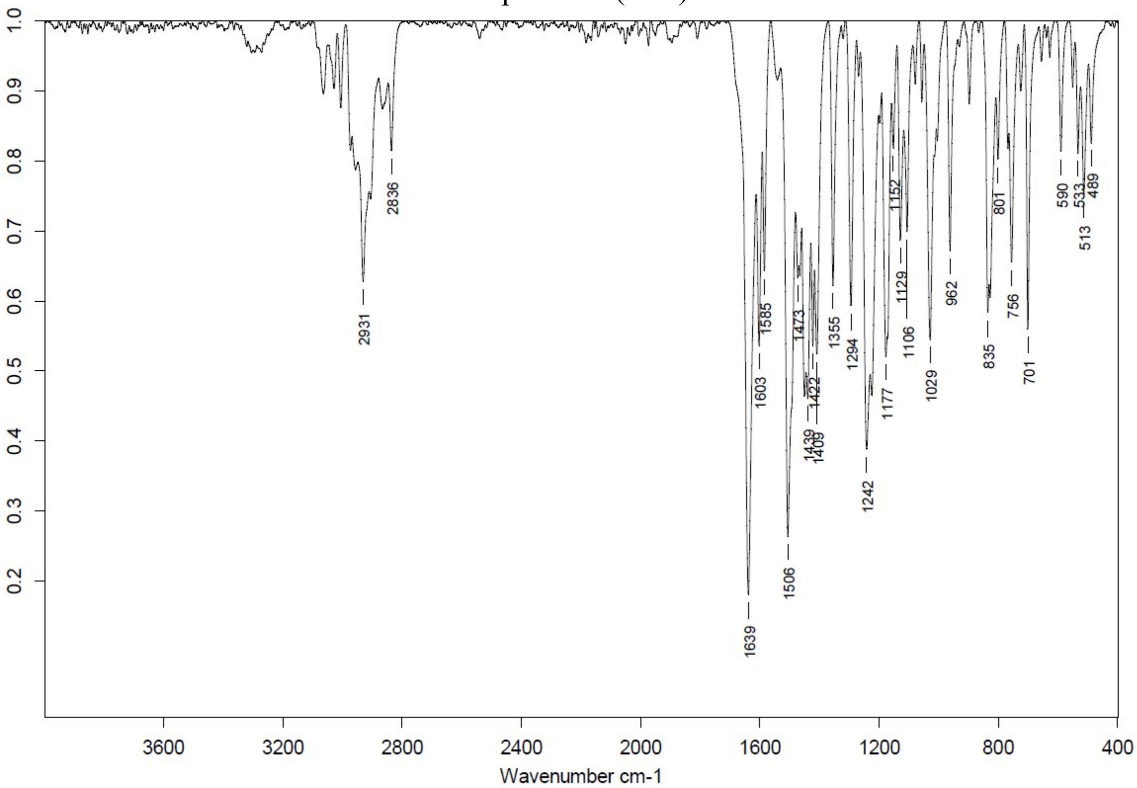

HRMS (ESI$^+$, MeOH): *m/z* calcd. 296.1645 $(M + H)^+$, found: 296.1640.

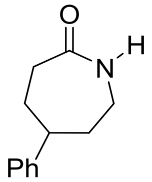

Compound 57 (from 56)

Scale: 0.15 mmol; isolated 21.3 mg (75% yield), light brown solid, DCM : MeOH = 30 : 1, $R_f = 0.14$

^{1}H NMR spectrum ($CDCl_3$)

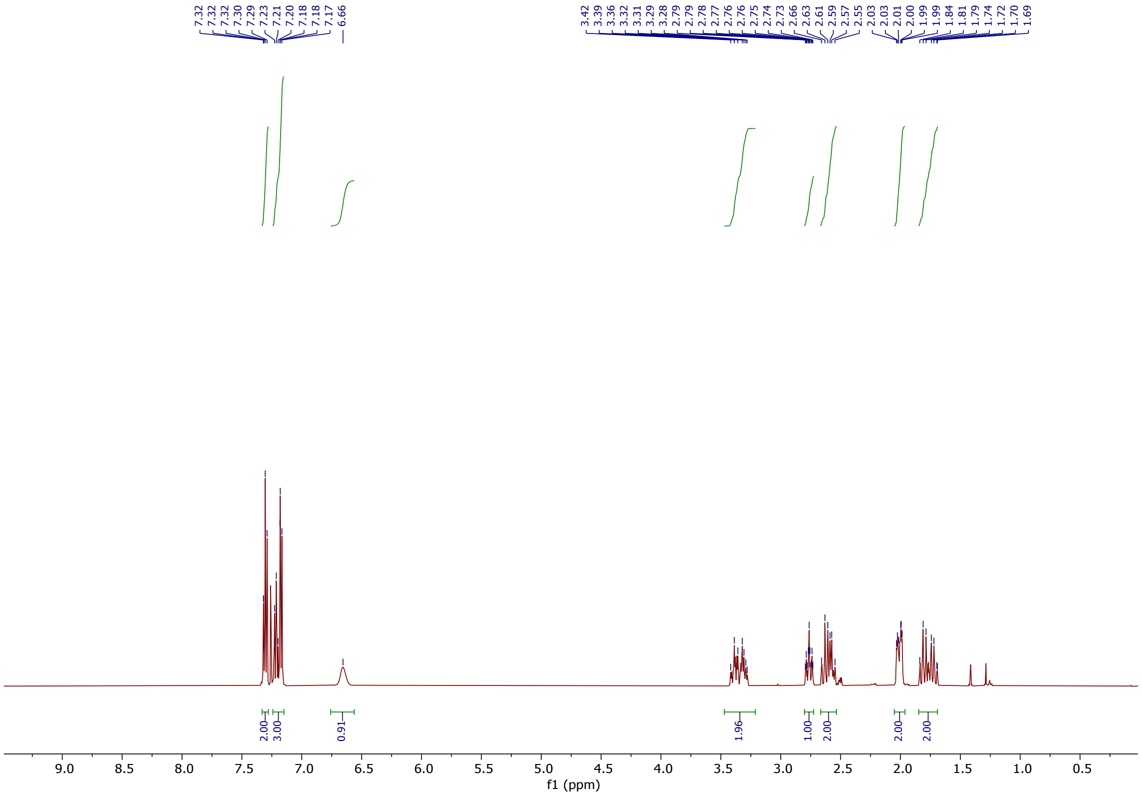

^{1}H NMR (500 MHz, $CDCl_3$) δ 7.33 – 7.28 (m, 2H), 7.24 – 7.15 (m, 3H), 6.66 (s, 1H), 3.47 – 3.21 (m, 2H), 2.76 (tt, J = 12.1, 3.4 Hz, 1H), 2.67 – 2.53 (m, 2H), 2.05 – 1.96 (m, 2H), 1.85 – 1.69 (m, 2H).

^{13}C NMR spectrum ($CDCl_3$)

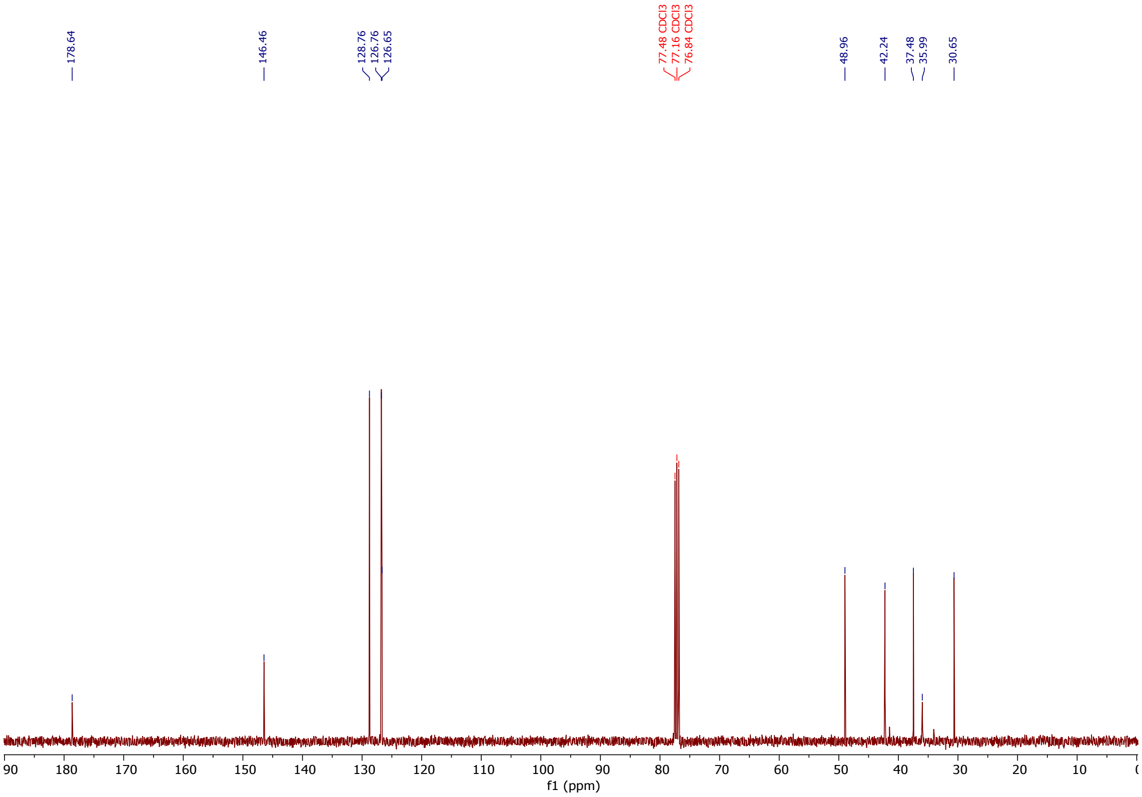

^{13}C NMR (101 MHz, $CDCl_3$) δ 178.64, 146.46, 128.76, 126.76, 126.65, 48.96, 42.24, 37.48, 35.99, 30.65.[6]

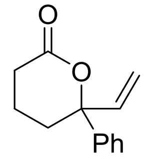

Compound 58

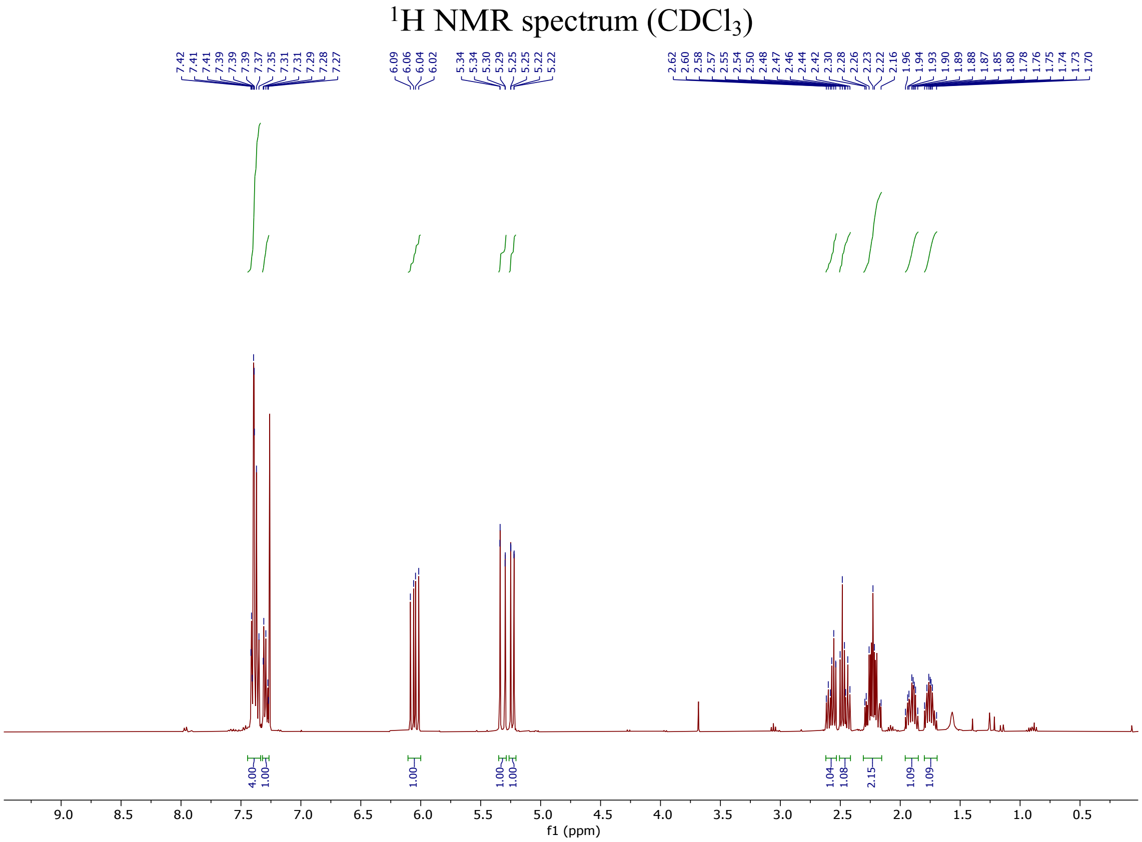

^{1}H NMR (400 MHz, $CDCl_3$) δ 7.44 – 7.34 (m, 4H), 7.32 – 7.27 (m, 1H), 6.05 (dd, J = 17.2, 10.7 Hz, 1H), 5.32 (dd, J = 17.2, 0.8 Hz, 1H), 5.24 (dd, J = 10.8, 0.8 Hz, 1H), 2.62 – 2.53 (m, 1H), 2.51 – 2.42 (m, 1H), 2.31 – 2.15 (m, 2H), 1.96 – 1.85 (m, 1H), 1.80 – 1.69 (m, 1H).

^{13}C NMR spectrum ($CDCl_3$)

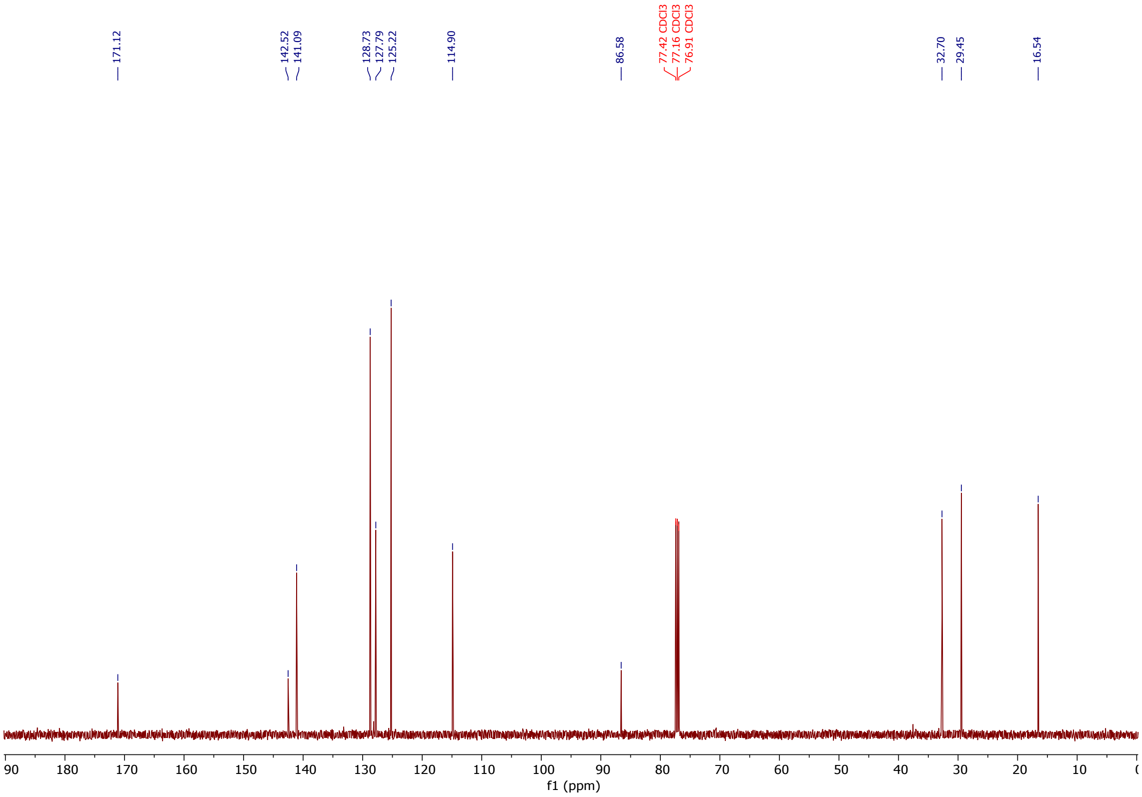

^{13}C NMR (126 MHz, $CDCl_3$) δ 171.12, 142.52, 141.09, 128.73, 127.79, 125.22, 114.90, 86.58, 32.70, 29.45, 16.54.[5]

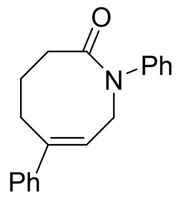

Compound 59

Scale: 0.2 mmol; isolated 30.0 mg (54% yield), slight yellow solid, Hexane : EA = 2 : 1, R_f = 0.25

^{1}H NMR spectrum ($CDCl_3$)

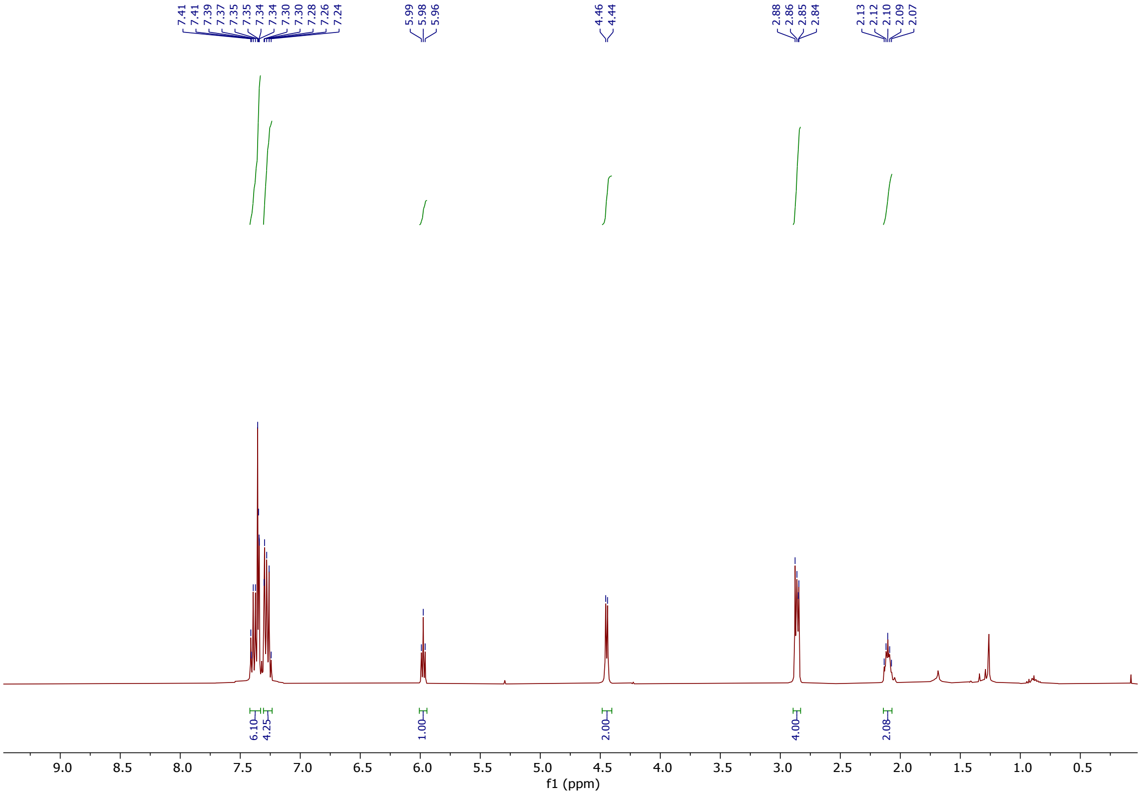

^{1}H NMR (400 MHz, $CDCl_3$) δ 7.42 – 7.33 (m, 6H), 7.31 – 7.23 (m, 4H), 5.98 (t, J = 6.2 Hz, 1H), 4.45 (d, J = 6.2 Hz, 2H), 2.89 – 2.83 (m, 4H), 2.14 – 2.07 (m, 2H).

^{13}C NMR spectrum ($CDCl_3$)

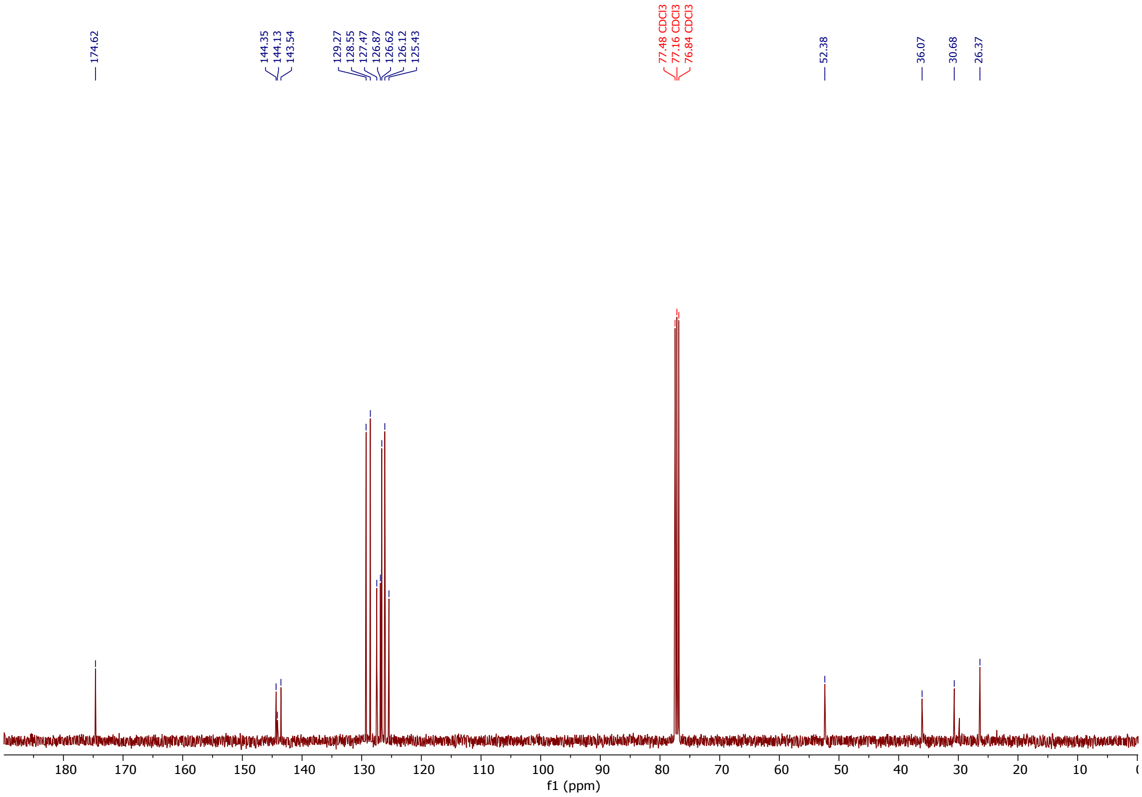

^{13}C NMR (101 MHz, $CDCl_3$) δ 174.62, 144.35, 144.13, 143.54, 129.27, 128.55, 127.47, 126.87, 126.62, 126.12, 125.43, 52.38, 36.07, 30.68, 26.37.

IR spectrum (neat)

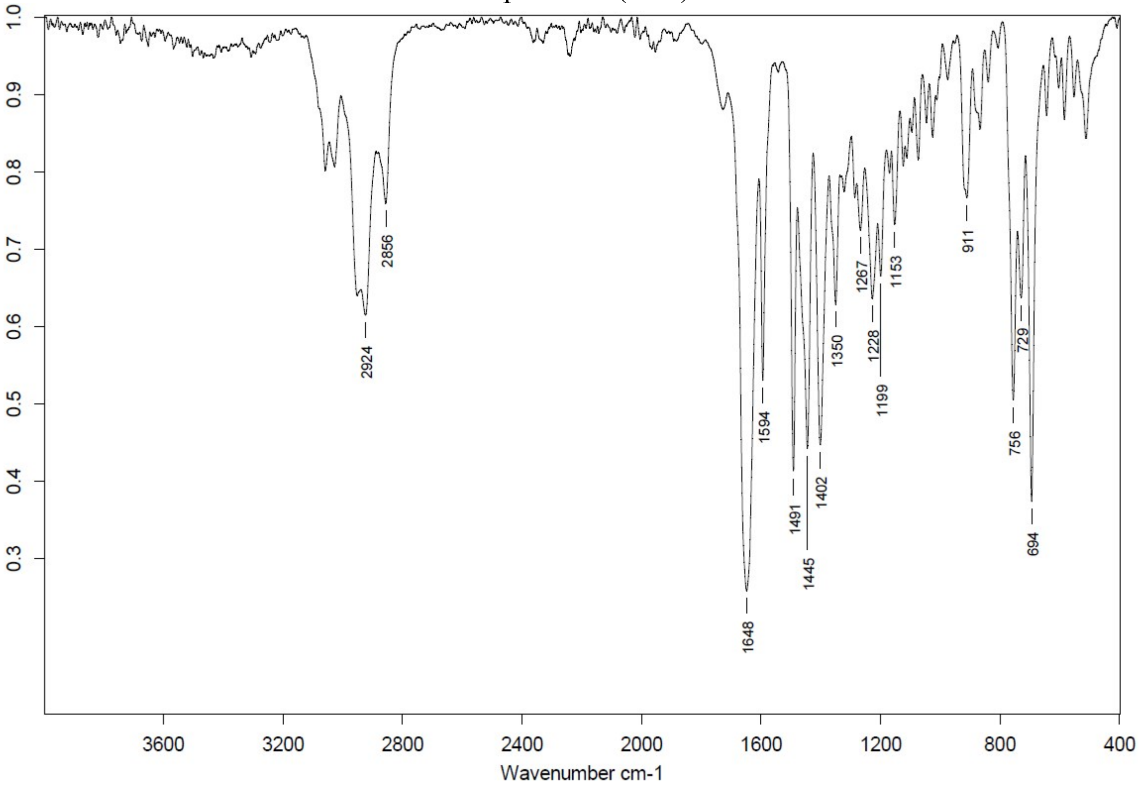

HRMS (ESI^+, MeOH): *m/z* calcd. 300.1359 $(M + Na)^+$, found: 300.1347.

S211. Control reactions

The nucleophilic attack at the less hindered, terminal carbon is typically favored in Pd-catalyzed allylic substitution reactions. However, this preference can be modulated by the sterics and electronics of the π-allylmetals. For electronically related π-acceptor ligands (see: *B. L. Feringa, J. F. Teichert, Angew. Chem. Int. Ed.* ***2010****, 49, 2486*) such as phosphoramidites and phosphites can increase the cationic character of the allyl unit, with an increased cationic character being more stable at the internal carbon center of the allyl group and thus directs (electronically) the nucleophilic addition to that position (see also: *B. M. Trost, M. R. Machacek, A. Aponick, Acc. Chem. Res.* ***2006****, 39, 747*). According to our previous research based on Pd/phosphoramidite mediated regioselective allylic substitution (see refs. 14a and 17 of the main text), it showed significant potential for "branched amination" and thus providing a possible regio-selectivity issue when using phosphoramidite ligands (such as **L8**) in the formation of caprolactams in our tandem process, and potentially lowering the yield of the target caprolactam. Indeed, when we used vinyl cyclic carbonate as an allylic surrogate instead of a γ-vinyl lactone under the standard conditions, a mixture of regio-isomers were detected with a ratio of branched/(*Z*)-linear of 15:85 as determined by ^{1}H NMR integration. **Note**: branched allylic amine product (double doublet at 6.45 ppm), linear allylic amine product with a triplet at 6.18 ppm.

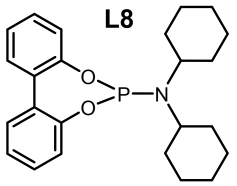

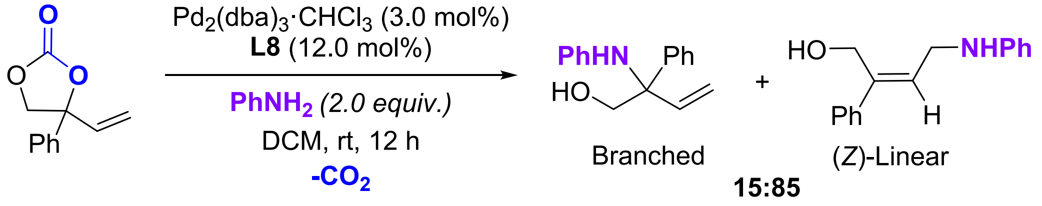

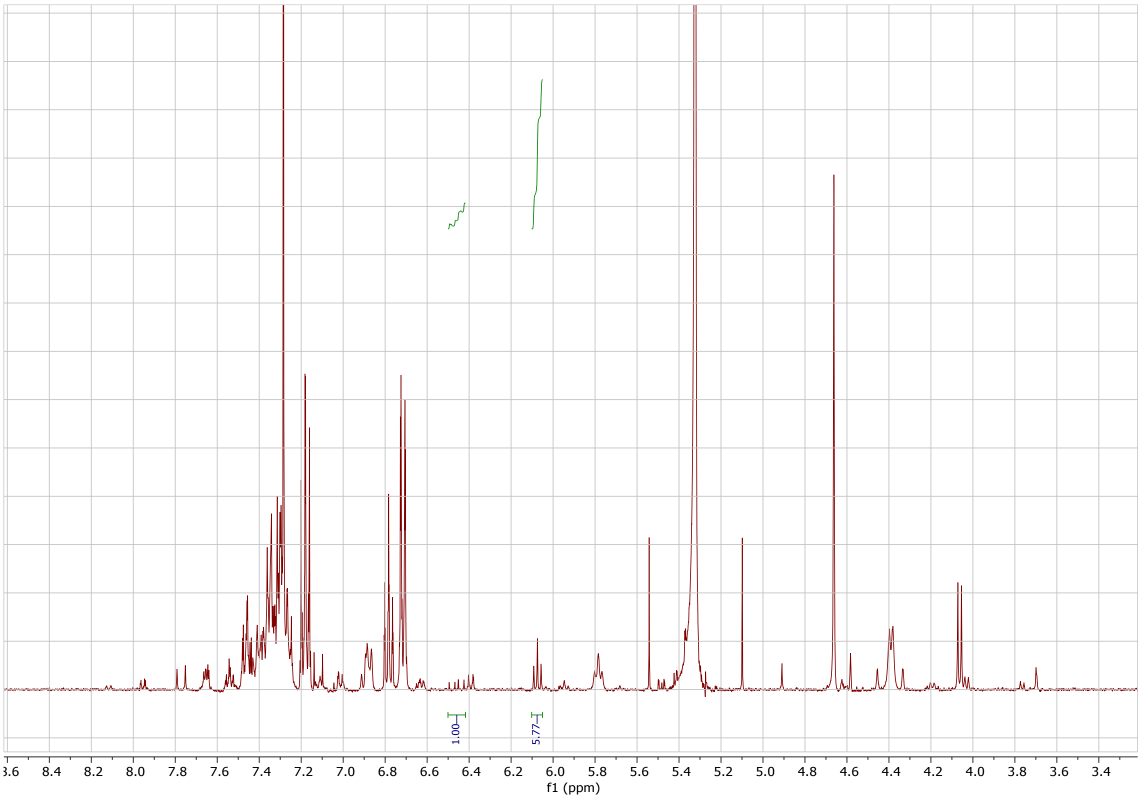

The use of a vinyl lactone having a secondary carbon center (R^1 = H, see below: this is a known compound) was also attempted after its preparation through the following procedure[7]:

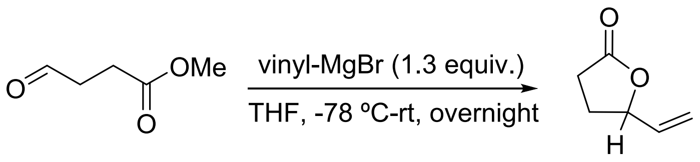

Amination (after ring-opening) of this vinyl γ-lactone substrate (with R^1 = H) proceeded efficiently under standard conditions (main text, Table 1, entry 20) with 100% conversion and producing a 71% NMR yield of the *ε*-amino acid intermediate. However, the stereocontrol was significantly decreased with a *Z/E* ratio of 11:89 (**note**: for this compound the *E/Z* priority assignment is different and here the "Z" isomer is warranted for cyclization). As a result, only trace amount of the desired caprolactam could be achieved. This reasoning follows the general observation that preparation of (*Z*)-configured 1,2-disubstituted alkenes by allylic substitution is very challenging because of steric effects.

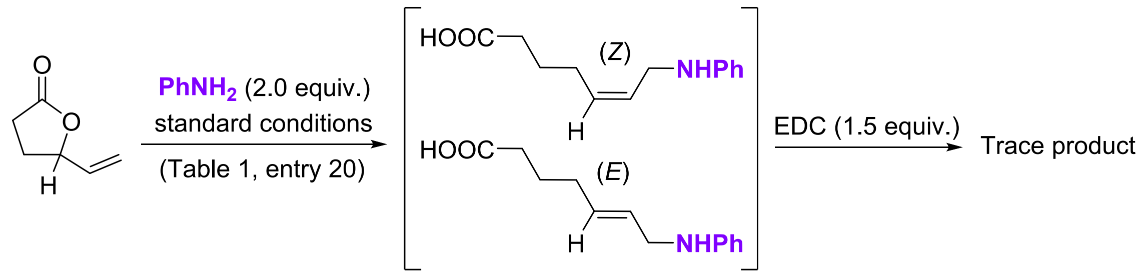

100% conversion
71% NMR yield
***Z/E* = 11:89**

Analytical data for this unsubstituted vinyl lactone:

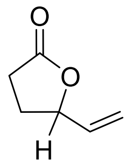

Using general procedure **B**: Light yellow oil; Column conditions: Hexane : EA = 10 : 1, R_f = 0.15

^{1}H NMR spectrum ($CDCl_3$)

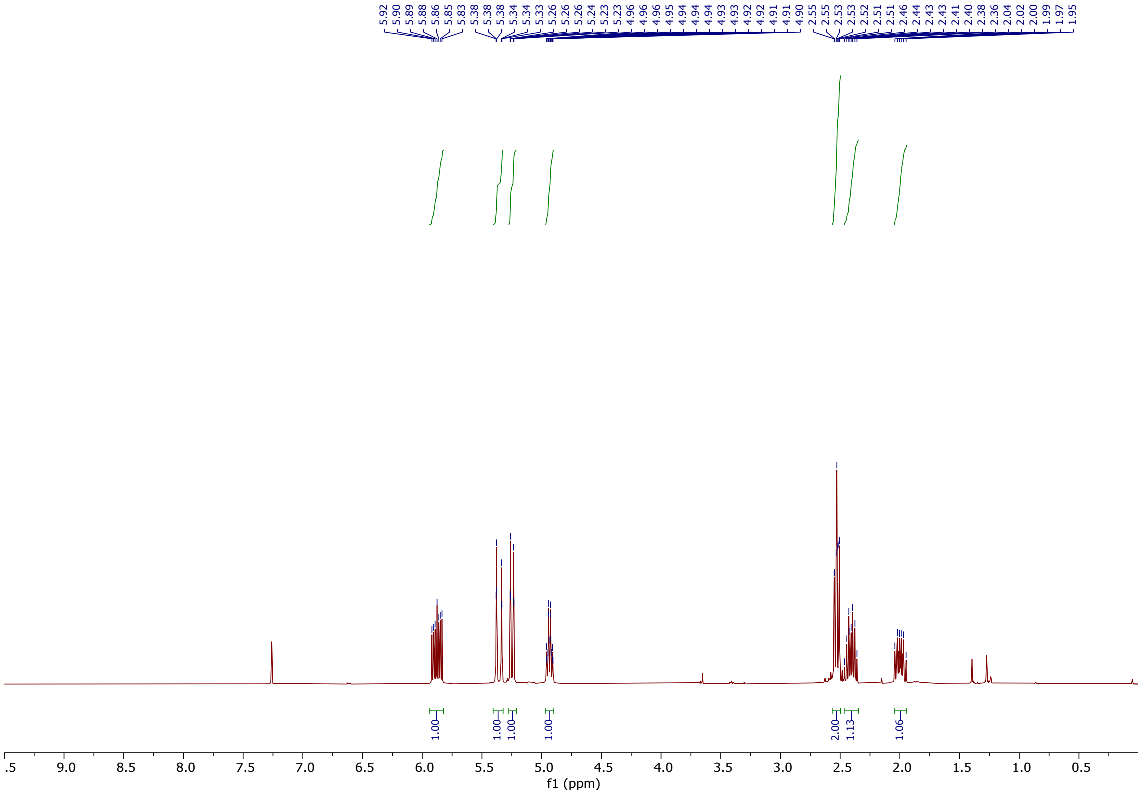

^{1}H NMR (400 MHz, $CDCl_3$) δ 5.94 – 5.82 (m, 1H), 5.36 (dt, J = 17.2, 1.2 Hz, 1H), 5.25 (dt, J = 10.5, 1.1 Hz, 1H), 4.96 – 4.89 (m, 1H), 2.53 (ddd, J = 8.6, 7.1, 1.9 Hz, 2H), 2.47 – 2.34 (m, 1H), 2.05 – 1.94 (m, 1H).

^{13}C NMR spectrum ($CDCl_3$)

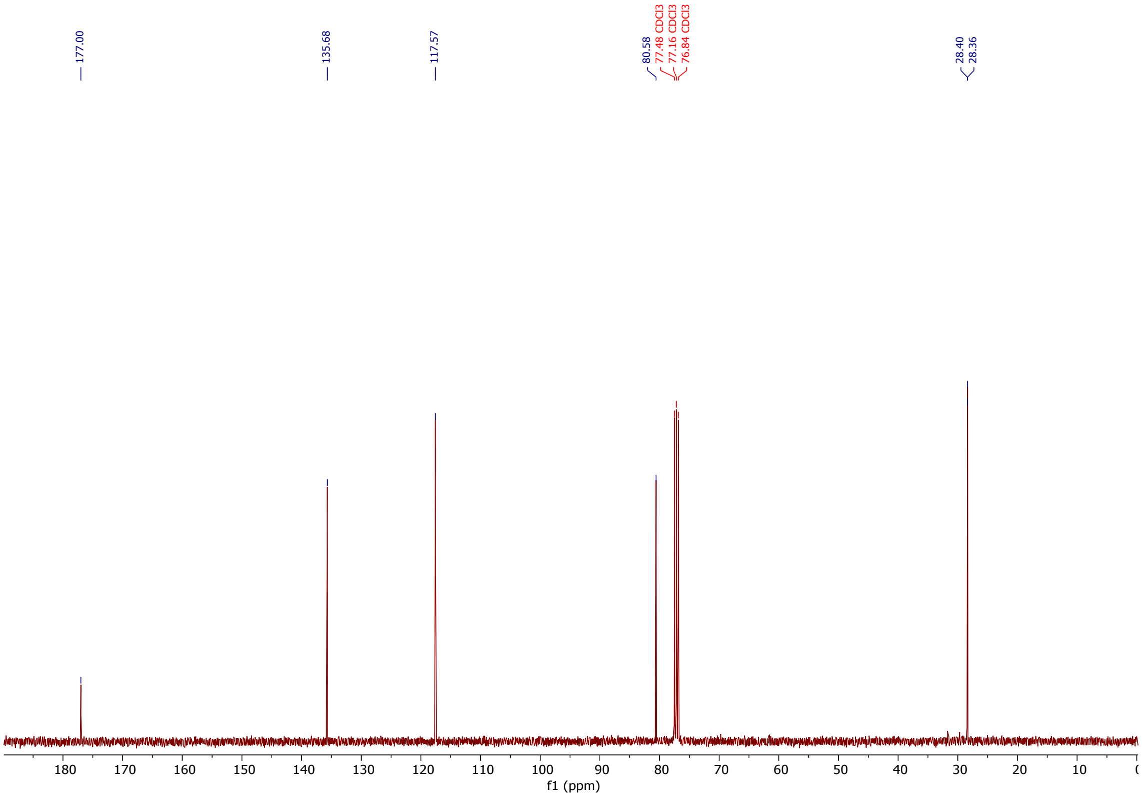

^{13}C NMR (101 MHz, $CDCl_3$) δ 177.00, 135.68, 117.57, 80.58, 28.40, 28.36.[8]

The preparation of a vinyl ε-lactone substrate failed by using the standard method used to get access to the *γ*-lactones. After some optimization, it could be prepared though a two-step method from 5-benzoyl pentanoic acid:

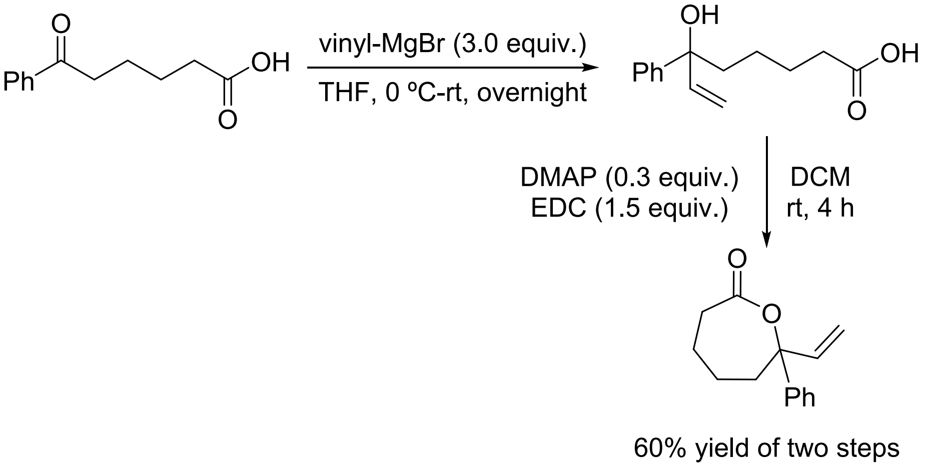

Procedure: under a N_2 atmosphere, to a separate flame-dried round-bottom flask equipped with a stirring bar was added the respective 5-benzoyl pentanoic acid (1.03 g, 5.0 mmol) and anhydrous THF (15 mL). The solution was cooled down to 0 ºC (ice/water), followed by dropwise addition of vinyl magnesium bromide in THF (1.0 M, 15.0 mL, 15.0 mmol). The reaction mixture was allowed to warm to room temperature and stirred for 12 h. The reaction mixture was quenched with saturated aqueous NH_4Cl, then treated with HCl (4 M) until the pH was 3. The organic components were extracted with EtOAc (3 × 20 mL). Hereafter, the combined organic layers were washed with brine, dried over anhydrous Na_2SO_4, filtered, and then concentrated under reduced pressure. The residue could be used directly for next step. To a solution of the 5-hydroxyl acid (1.0 equiv.) in DCM (15 mL) was added DMAP (0.1833, 0.3 equiv) and EDC (1.4375 g, 1.5 equiv). The reaction was stirred at room temperature for 4 h. The reaction mixture was then concentrated under reduced pressure. The residue was purified by flash chromatography on silica to afford the vinyl *ε*-lactone substrate.

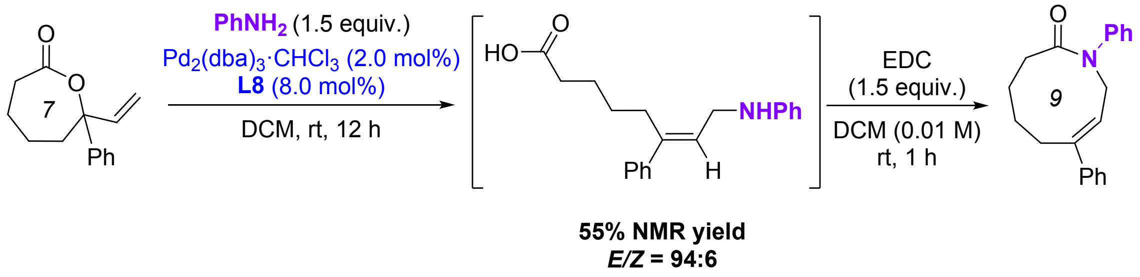

The allylic amination step could be realized using modified conditions (55% NMR yield, *E/Z* = 94:6), while low yield (< 10%) of nine-membered lactam was detected from the crude reaction mixture by ^1H NMR after attempted cyclization.

Procedure: the vinyl *ε*-lactone (43.3 mg, 0.20 mmol, 1.0 equiv) was combined with $Pd_2(dba)_3 \cdot CHCl_3$ (4.0 mg, 2.0 mol%), **L8** (6.4 mg, 8.0 mol%) and aniline (28.5 mg, 0.30

mmol) in DCM (0.30 mL) at rt in air. The reaction mixture was stirred at rt for 12 h, then an aliquot of the mixture was taken for NMR analysis, which showed the unsaturated amino acid intermediate (55% NMR yield) to have an *E*/*Z* ratio of 96:4. Hereafter, the mixture was diluted to 0.01 M and EDC (57.5 mg, 3.0 mmol) was added, and the reaction mixture stirred for another 1 h. Hereafter, only a trace amount of target product could be detected by ^{1}H NMR of the crude mixture.

The lower yield of the nine-membered lactam can be explained by the increasing entropic cost to pre-organize the substrate for intramolecular cyclization versus intermolecular “amide” bond formation.

Analytical data for the seven-membered vinyl lactone:

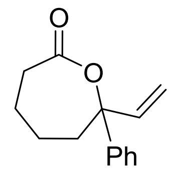

^{1}H NMR spectrum ($CDCl_3$)

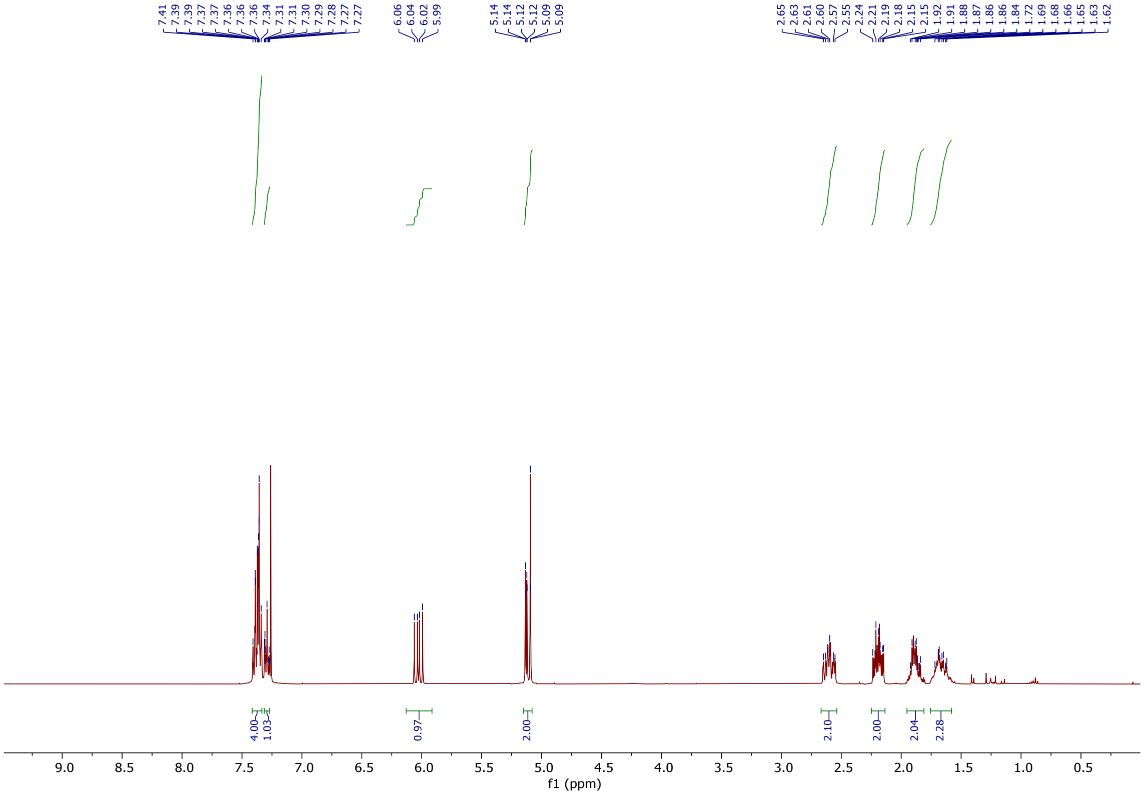

^{1}H NMR (400 MHz, $CDCl_3$) δ 7.41 – 7.33 (m, 4H), 7.31 – 7.27 (m, 1H), 6.03 (dd, J = 17.3, 10.6 Hz, 1H), 5.15 – 5.08 (m, 2H), 2.67 – 2.54 (m, 2H), 2.25 – 2.13 (m, 2H), 1.95 – 1.81 (m, 2H), 1.76 – 1.58 (m, 2H).

^{13}C NMR spectrum ($CDCl_3$)

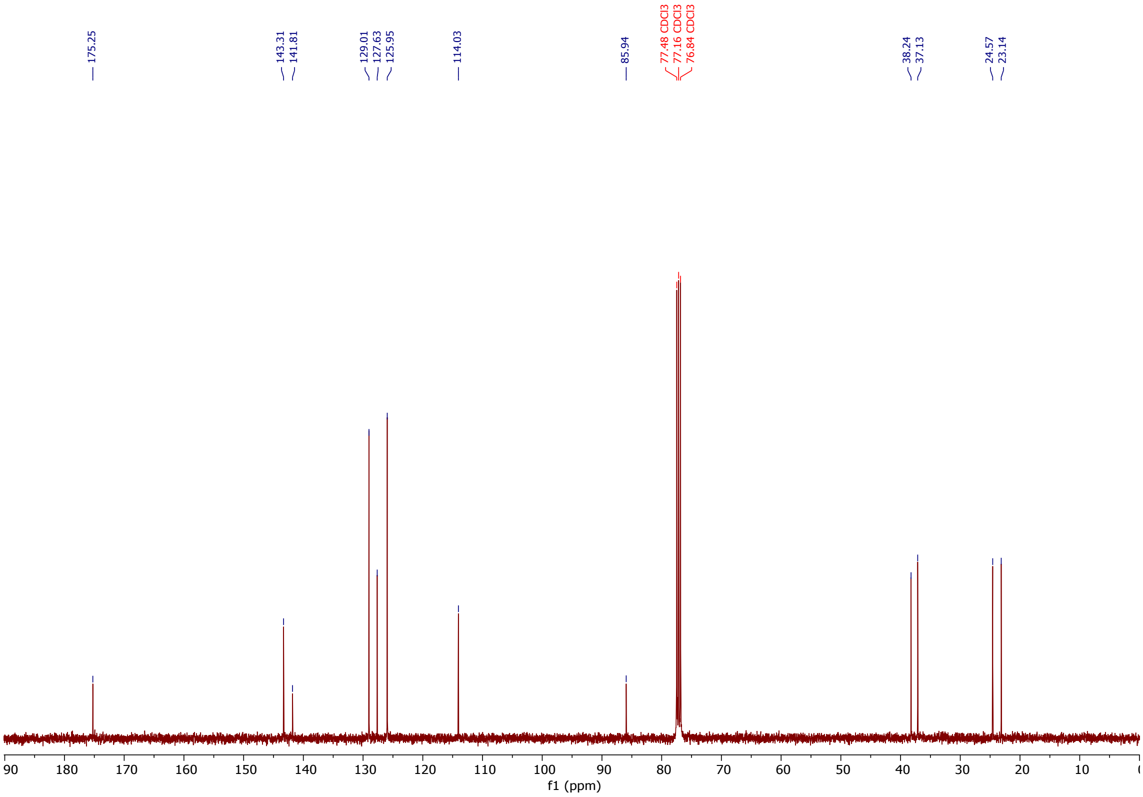

^{13}C NMR (101 MHz, $CDCl_3$) δ 175.25, 143.31, 141.81, 129.01, 127.63, 125.95, 114.03, 85.94, 38.24, 37.13, 24.57, 23.14.

IR spectrum (neat)

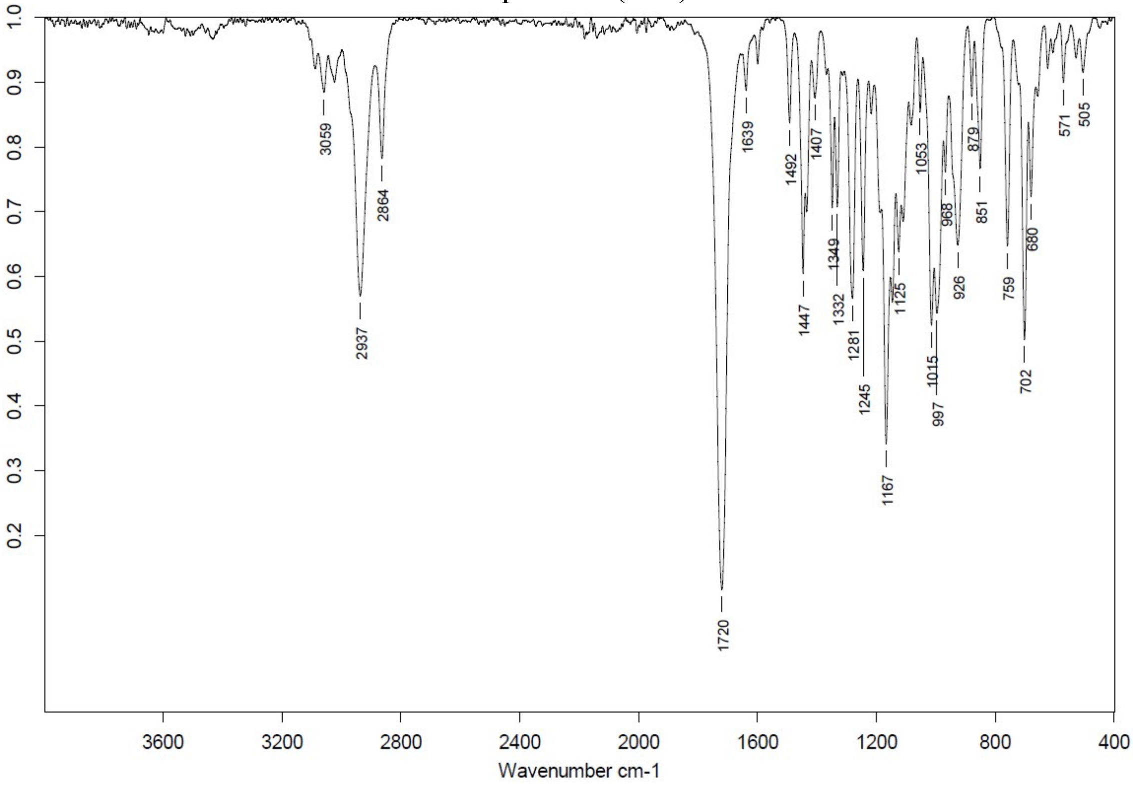

HRMS (ESI$^+$, MeOH): *m/z* calcd. 239.1043 (M + Na)$^+$, found: 239.1036.

S219. Characterization of byproduct 2 from bis-allylation of aniline

The isolation of completely pure byproduct **2** was unsuccessful. The characterization by ^{1}H NMR, IR and MS data of the purest sample of **2** is here provided to support its proposed structure.

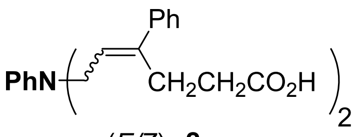

(*E*/*Z*)$_2$-**2**

Yellow solid, Hexane : EA = 1 : 3, R_f = 0.1, or DCM:MeOH = 30 :1, R_f = 0.12

^{1}H NMR spectrum ($CDCl_3$)

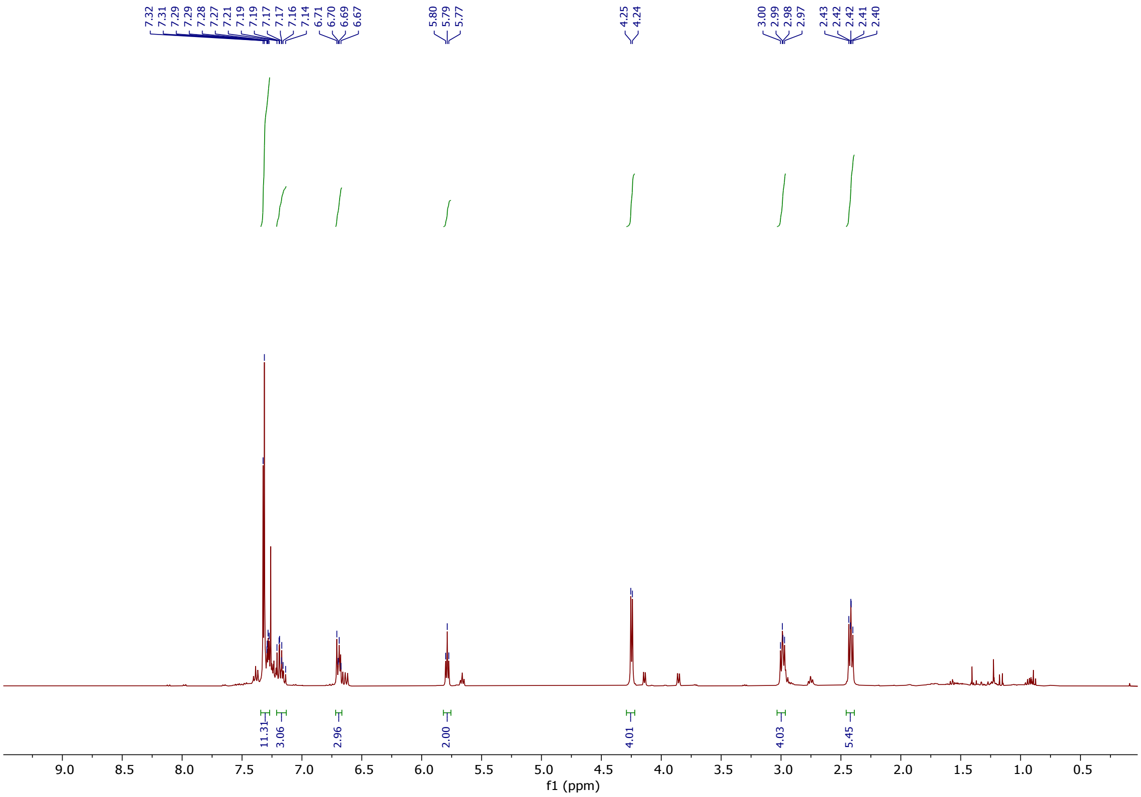

IR spectrum (neat)

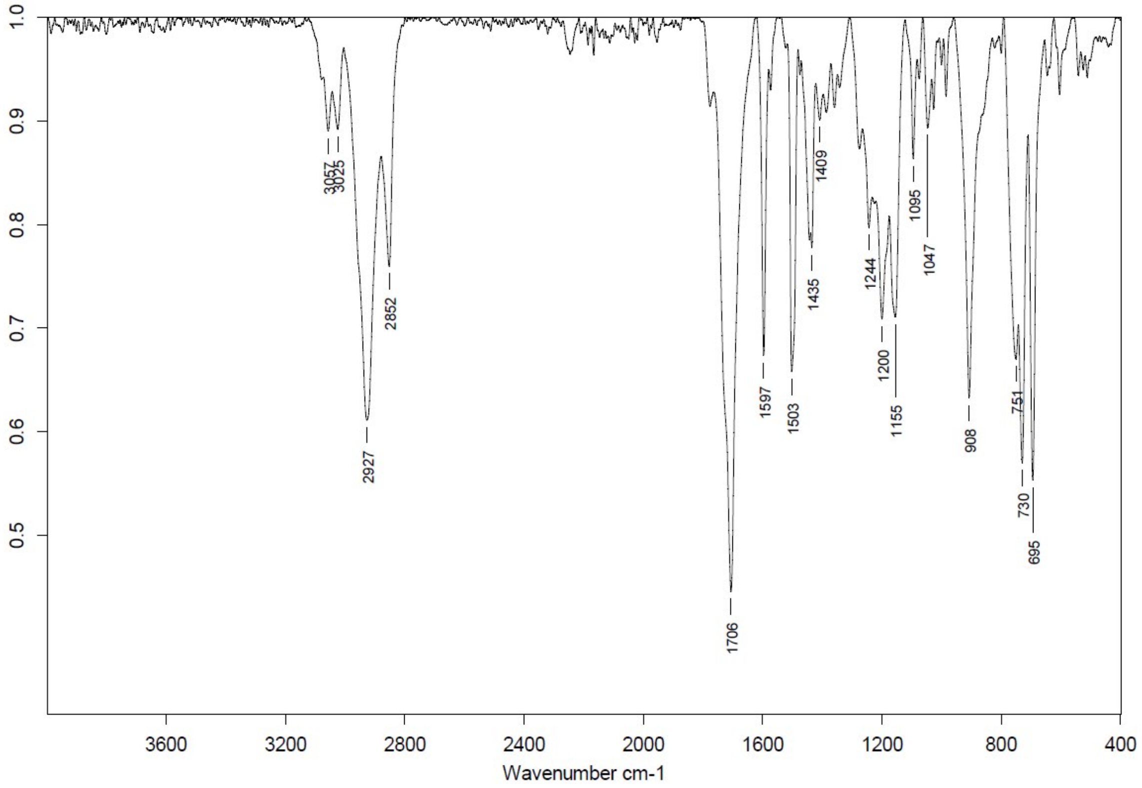

HPLC-MS conditions: Zorbax C18 100 × 4.6mm, 3.5µm, H_2O/MeOH = 30 : 70. 70% MeOH up to 100% in 10'; hold 5', 1 mL/min. APCI +/-; Sample: 1 mg/ml in MeOH.

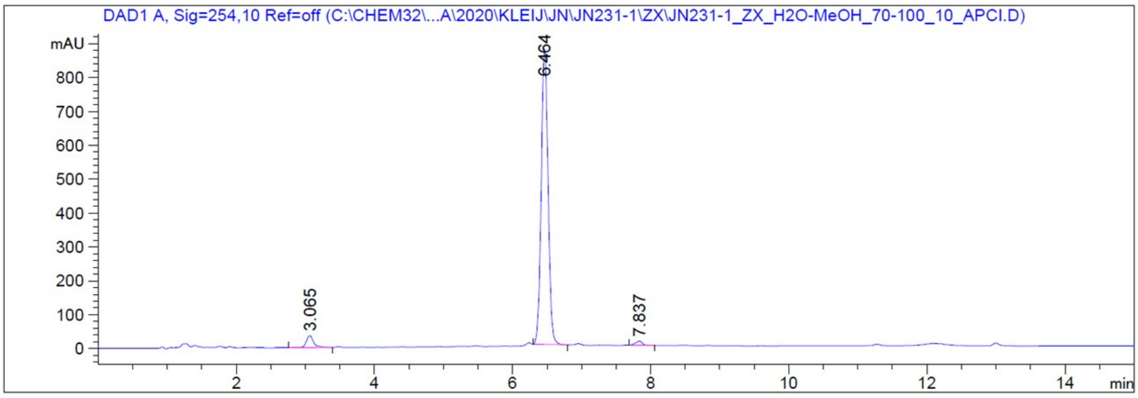

Peak #	RetTime [min]	Type	Width [min]	Area [mAU*s]	Height [mAU]	Area %
1	3.065	BB	0.1106	253.47015	34.93452	4.0079
2	6.464	BB	0.1059	5992.86816	874.79449	94.7589
3	7.837	BB	0.0957	77.99753	12.01399	1.2333

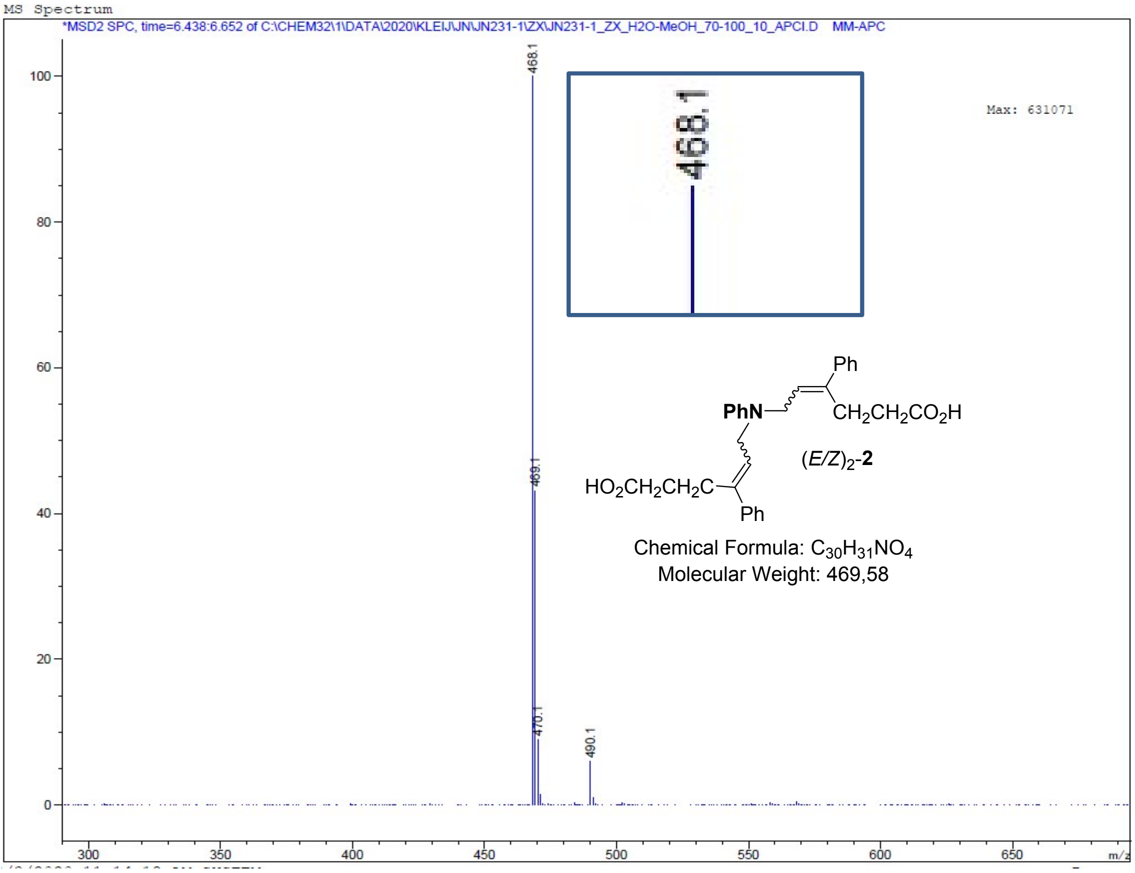

S222. X-ray structures of 3 and 53

Molecular structure for **3** (CCDC-1998235):

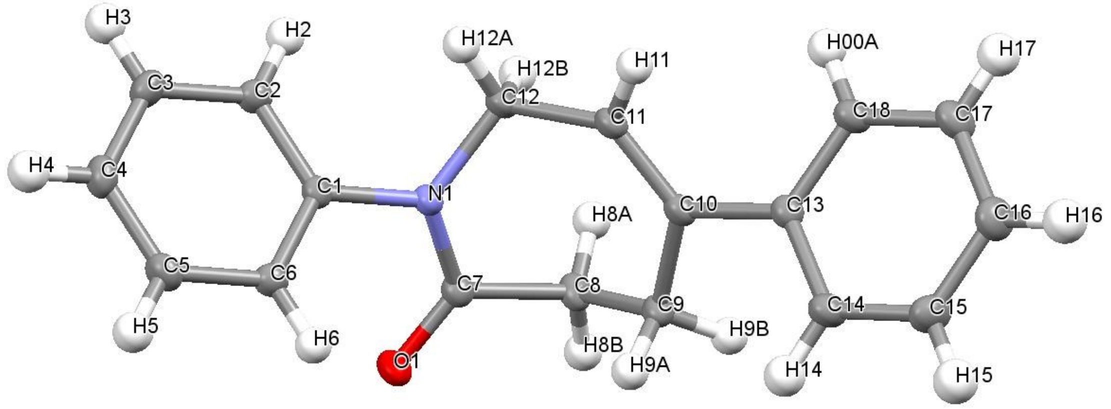

Molecular structure for **53** (CCDC-2001102):

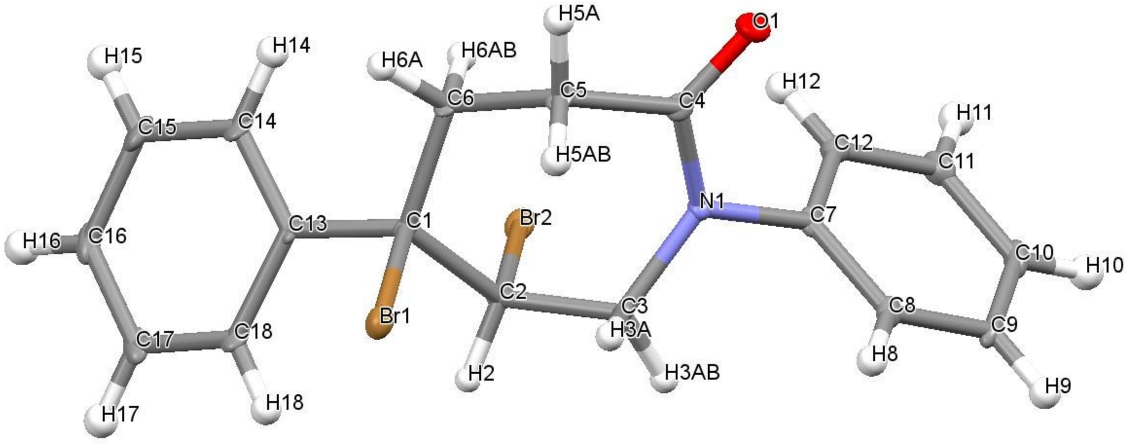

S223. References

1 (*a*) A. Z. González and F. D. Toste, *Org. Lett.*, 2010, **12**, 200; (*b*) N. Mršić, A. J. Minnaard, B. L. Feringa and J. G. de Vries, *J. Am. Chem. Soc.*, 2009, **131**, 8358; (*c*) B. Zhao, H. Du, R. Fu and Y. Shi, *Org. Synth.*, 2010, **87**, 263; (*d*) A. Mercier, W. C. Yeo, J. Chou, P. D. Chaudhuri, G. Bernardinelli, and E. P. Kündig, *Chem. Commun.*, 2009, 5227.

2 (*a*) N. Sakai, T. Kobayashi and Y. Ogiwara, *Chem. Lett.*, 2015, **44**, 1503; (*b*) P. Saravanan and P. Anbarasan, *Adv. Synth. Catal.*, 2018, **360**, 2894; (*c*) P. B. Cranwell, A. T. Russell and C. D. Smith, *Synlett.*, 2016, **27**, 131; (*d*) C. Chen, Z. Zhang, S. Jin, X. Fan, M. Geng, Y. Zhou, S. Wen, X. Wang, L. W. Chung, X.-Q. Dong and X. Zhang, *Angew. Chem. Int. Ed.*, 2017, **56**, 6808; (*e*) S. Cuadros, L. DellAmico and P. Melchiorre, *Angew. Chem. Int. Ed.*, 2017, **56**, 11875; (*f*) T. Song, S. Arseniyadis and J. Cossy, *Org. Lett.*, 2019, **21**, 603; (*g*) J. Koyanagi, K. Yamamoto, K. Nakayama and A. Tanaka, *J. Nat. Prod.*, 1995, **58**, 1955; (*h*) S. Li, J. Zhang, H. Li, L. Feng and P. Jiao, *J. Org. Chem.*, 2019, **84**, 9460; (*i*) S. Lee, H. Y. Bae and B. List, *Angew. Chem. Int. Ed.*, 2018, **57**, 12162.

3 J. E. Gómez, W. Guo, S. Gaspa and A. W. Kleij, *Angew. Chem. Int. Ed.*, 2017, **56**, 15035.

4 (*a*) Y. Odabachian, Q. Wang and J. Zhu, *Chem. Eur. J.*, 2013, **19**, 12229; (*b*) L. F. R. Gomes, L. F. Veiros, N. Maulide and C. A. M. Afonso, *Chem. Eur. J.*, 2015, **21**, 1449.

5 X.-L. Xu and Z. Li, *Org. Lett.*, 2019, **21**, 5078.

6 D. Kong, M. Li, G. Zi and G. Hou, *Org. Biomol. Chem.*, 2016, **14**, 4046.

7 R. L. Sutar, S. Sen, O. Eivgi, G. Segalovich, I. Schapiro, O. Reany and N. G. Lemcoff, *Chem. Sci.*, 2018, **9**, 1368.

8 Y. Suzuki, T. Seki, S. Tanaka and M. Kitamura, *J. Am. Chem. Soc.*, 2015, **137**, 9539.
